# *Capsicum chinense MYB* Transcription Factor Genes: Identification, Expression Analysis, and Their Conservation and Diversification With Other Solanaceae Genomes

**DOI:** 10.3389/fpls.2021.721265

**Published:** 2021-10-13

**Authors:** Khushbu Islam, Abdul Rawoof, Ilyas Ahmad, Meenakshi Dubey, John Momo, Nirala Ramchiary

**Affiliations:** ^1^School of Life Sciences, Jawaharlal Nehru University, New Delhi, India; ^2^Department of Biotechnology, Delhi Technological University, New Delhi, India

**Keywords:** *Capsicum chinense*, *baccatum*, *MYB*, Solanaceae, fruit development, transcription factors

## Abstract

Myeloblastosis (*MYB*) genes are important transcriptional regulators of plant growth, development, and secondary metabolic biosynthesis pathways, such as capsaicinoid biosynthesis in *Capsicum*. Although *MYB* genes have been identified in *Capsicum annuum*, no comprehensive study has been conducted on other *Capsicum* species. We identified a total of 251 and 240 *MYB* encoding genes in *Capsicum chinense MYBs* (*CcMYBs*) and *Capsicum baccatum MYBs* (*CbMYBs*). The observation of twenty tandem and 41 segmental duplication events indicated expansion of the *MYB* gene family in the *C. chinense* genome. Five *CcMYB* genes, i.e., *CcMYB101, CcMYB46, CcMYB6, CcPHR8*, and *CcRVE5*, and two *CaMYBs*, i.e., *CaMYB3* and *CaHHO1*, were found within the previously reported capsaicinoid biosynthesis quantitative trait loci. Based on phylogenetic analysis with tomato *MYB* proteins, the *Capsicum* MYBs were classified into 24 subgroups supported by conserved amino acid motifs and gene structures. Also, a total of 241 *CcMYBs* were homologous with 225 *C. annuum*, 213 *C. baccatum*, 125 potato, 79 tomato, and 23 Arabidopsis *MYBs*. Synteny analysis showed that all 251 *CcMYBs* were collinear with *C. annuum, C. baccatum*, tomato, potato, and Arabidopsis *MYBs* spanning over 717 conserved syntenic segments. Using transcriptome data from three fruit developmental stages, a total of 54 *CcMYBs* and 81 *CaMYBs* showed significant differential expression patterns. Furthermore, the expression of 24 *CcMYBs* from the transcriptome data was validated by quantitative real-time (qRT) PCR analysis. Eight out of the 24 *CcMYBs* validated by the qRT-PCR were highly expressed in fiery hot *C. chinense* than in the lowly pungent *C. annuum*. Furthermore, the co-expression analysis revealed several *MYB* genes clustered with genes from the capsaicinoid, anthocyanin, phenylpropanoid, carotenoid, and flavonoids biosynthesis pathways, and related to determining fruit shape and size. The homology modeling of 126 R2R3 CcMYBs showed high similarity with that of the Arabidopsis R2R3 *MYB* domain template, suggesting their potential functional similarity at the proteome level. Furthermore, we have identified simple sequence repeat (SSR) motifs in the *CcMYB* genes, which could be used in *Capsicum* breeding programs. The functional roles of the identified *CcMYBs* could be studied further so that they can be manipulated for *Capsicum* trait improvement.

## Introduction

The myeloblastosis (*MYB*) gene family is one of the largest transcription factor (TF) families in plants (Romero et al., 1998; Riechmann et al., 2000). MYB TFs have one or more imperfect repeats of the characteristic DNA-binding domain (DBD) in the basic region of a protein (Klempnauer and Sippel, 1987). Each repeat comprises about 53 amino acids with three regularly placed tryptophan residues forming a helix-turn-helix structure (Ogata et al., 1994; König et al., 1996). *MYB* TFs with only one repeat are called “MYB1R” or MYB-related, while those with two, three, or four repeats are called “R2R3-MYB,” “MYB3R,” or “MYB4R,” respectively. In plants, most MYBs belong to the R2R3 class, unlike in animals, and exhibit plant-specific responses (Martin and Paz-Ares, 1997). MYB TFs play key roles in the regulation of phenylpropanoid and flavonoid metabolism in plants; for instance, in Arabidopsis, *MYB123* partly determines the accumulation of proanthocyanidin (PA) in the coat of seeds (Nesi et al., 2001), *MYB11, MYB12*, and *MYB111* are involved in the transcriptional regulation of the *chalcone synthase* and *flavonol synthase* genes (Mehrtens et al., 2005), while *MYB14* and *MYB15*, along with WRKY53, are reported to regulate stilbene synthesis in Chinese wild grapes (Wang et al., 2020b). *MYB88* and *MYB124* were reported to have diverse roles (Lei et al., 2015), such as in the regulation of mitotic divisions of the stomatal guard mother cell (Lai et al., 2005; Lee et al., 2013) and direct transcriptional regulation of auxin transporter PIN-FORMED proteins in roots of *Arabidopsis thaliana* (Xie et al., 2010; Wang et al., 2015; Geng et al., 2018). Reports also suggest their role in female reproductive development (Makkena et al., 2012) and conditional repression of non-stomatal epidermal cells in Arabidopsis cotyledons (Yang, 2016). *MYB75* and *MYB90* are known to activate phenylpropanoid biosynthetic genes and the accumulation of purple anthocyanins in Arabidopsis (Kranz et al., 1998; Borevitz et al., 2000). Furthermore, several *MYBs* have been reported to induce anthocyanin production in different organs, including fruits in tomatoes (Kiferle et al., 2015; Jian et al., 2019; Yan et al., 2020), potato (Li et al., 2021), and in other plants (Quattrocchio et al., 2006; Takos et al., 2006; Cutanda-Perez et al., 2009; Czemmel et al., 2009; Kortstee et al., 2011; Wang et al., 2017; Yan et al., 2019). In *Capsicum, MYB*_*A*_ and *CaAN2* control anthocyanin pigmentation in flower and fruit tissues (Aguilar-Barragán and Ochoa-Alejo, 2014; Jung et al., 2019). However, MYB TFs have been scarcely studied for their protein structures; for example, a MYB-related motif in Arabidopsis recognizes the major groove of target DNA *via* the amino acid residues present in three alpha helices while binding to the minor groove using an N-terminal arm (Hosoda et al., 2002). Another report characterized the crystal structure of the MYB domain from an *Antirrhinum majus* single MYB repeat RADIALIS (RAD) TF, which functions in the development of floral asymmetry (Stevenson et al., 2006).

The genus *Capsicum* comprises several species grown worldwide mostly for vegetables and spices, which are of high economic and nutritional value. The *Capsicum* fruit is known for its unique attribute of pungency owing to alkaloids, known as capsaicinoids complex, mainly capsaicin and dihydrocapsaicin (Antonious and Jarret, 2006), which have pharmaceutical applications (Fattori et al., 2016). There exists a wide variation in capsaicin content in *Capsicum* genotypes, with the highest being reported in Bhut jolokia (*C. chinense*; Sarpras et al., 2016; Chhapekar et al., 2020). *R2R3-MYB31* was reported as a transcriptional regulator of capsaicinoid biosynthetic genes (CBGs; Arce-Rodríguez and Ochoa-Alejo, 2017). The same *MYB* was reported to be encoded by a pungency-controlling locus *Pun3* (Han et al., 2019), and its gene promoter showed natural variations between high and low pungent *C. annuum* species (Zhu et al., 2019). *CaMYB108* confers a pungent flavor to *Capsicum* genotypes and controls stamen development, and it is found to be induced by methyl jasmonate (Sun et al., 2019). Recently, *CaMYB48* was discovered to directly control the expression of *CBGs acyl transferase* (*AT3a*) and *ketoacyl-ACP synthase* (*KasIa*) and the accumulation of capsaicinoids in *C. annuum* (Sun et al., 2020).

*Myeloblastosis* genes have been identified only in *C. annuum* (Wang et al., 2020c; Arce-Rodríguez et al., 2021), and no comprehensive study has been reported in other *Capsicum* species, such as *C. chinense, C. baccatum*, and *C. frutescens*. In this study, we identified *MYB* genes in the *C. chinense, C. baccatum*, and *C. annuum* genomes, and the analysis of their expression was performed using transcriptome data and validated by quantitative real-time (qRT) PCR in the early green (EG), mature green (MG), and breaker (Br) fruit developmental stages of highly pungent *C. chinense* and lowly pungent *C. annuum*. Seven *MYB* genes were found within the previously reported capsaicinoids quantitative trait loci (QTLs) (Han et al., 2018; Park et al., 2019). The co-expression analysis revealed several *MYB* genes that clustered with capsaicinoid, anthocyanin, phenylpropanoid, flavonoid, fruit shape and size, carotenoid, and vitamin C biosynthesis pathway genes. Furthermore, we analyzed duplications of *MYB* genes in *C. chinense*, and comparative analysis with *C. baccatum, C. annuum*, tomato, potato, eggplant, and Arabidopsis showed conserved syntenic segments (CSSs) and collinear *MYB* genes, and diversification among them. In addition, we performed homology modeling of R2R3 CcMYB proteins and developed simple sequence repeat (SSR) markers in genic and promoter regions of *Capsicum MYBs*, which can be used to manipulate pungency levels in future *Capsicum* breeding programs. The identified *MYB* genes in this study could be used in the future to understand their regulatory roles in diverse biological functions including the capsaicinoid biosynthetic pathway.

## Materials and Methods

### Identification of *MYB* Genes in *Capsicum* Spp

For the identification of *MYB* genes, a local blastp analysis (e-value <1e-05) was performed against 34,974, 35,853, and 31,353 full-length protein sequences of *C. chinense* (GCA_002271895.2; Kim et al., 2017), *C. annuum* (GCA_000710875.1; Qin et al., 2014), and *C. baccatum* (GCA_002271885.2; Kim et al., 2017), respectively, using the R2R3-MYB family protein sequences of Arabidopsis (Stracke et al., 2001), tomato (Li et al., 2016), potato (Li et al., 2021), and *C. annuum* (Arce-Rodríguez et al., 2021) as a query. For the Hidden Markov Model (HMM) analysis, the sequences of the *Capsicum* proteins were queried against the protein sequences of MYB DNA-binding domains (PF00249 and PF13921) using HMMER (v3.2.1; http://hmmer.janelia.org/). The duplicate sequences were removed manually, and the remaining sequences were cross-checked for the presence of the MYB domain using the Conserved Domains Database (CDD) of the National Centre for Biotechnology Information (NCBI) (Lu et al., 2020) and the Simple Modular Architecture Research Tool (SMART); http://smart.embl-heidelberg.de/) database. The resultant *C. chinense* and *C. baccatum MYB* encoding genes were given acronyms according to their amino acid sequence homology with *C. annuum MYB* genes (Arce-Rodríguez et al., 2021). Physicochemical features, such as molecular weight, theoretical isoelectric point (pI), instability index, aliphatic index, and grand average of hydrophobicity (GRAVY) of the MYB protein sequences were estimated using ProtParam (https://web.expasy.org/protparam/; Gasteiger et al., 2005). The subcellular localization of *Capsicum* MYBs was determined using Cello (v.2.5 http://cello.life.nctu.edu.tw/).

### Chromosomal Distribution, Gene Duplication, and Co-localization With Capsaicinoid QTLs

The chromosomal positions of *Capsicum MYB* genes were obtained from the gene feature file (gff) of their genomes. The physical locations of *Capsicum MYB* genes, along with the capsaicin and dihydrocapsaicin QTLs as reported earlier (Han et al., 2018; Park et al., 2019), were represented across 12 *Capsicum* chromosomes using TBtools (v1.068; Chen et al., 2020). The duplication of *MYB* genes within the *C. chinense* genome was identified based on filter criteria of >75% identity and query coverage of above 75% of the gene length. Gene pairs with a <100-kb (kilobase) distance on the same chromosome were considered as tandem duplicates, while those with >100 kb were considered as segmental duplicates. The rate of non-synonymous (*Ka*) and synonymous substitutions (*Ks*) and their ratio (ω = *Ka/Ks*) for all duplicated gene pairs were estimated using KaKs_Calculator 2.0 (Wang et al., 2010). The value of ω ~ 0 indicates neutral selection, ω <1 indicates purifying selection, and ω > 1 indicates positive selection. The date of duplication (diversion time) was calculated using the formula T = Ks/2λ, assuming a clock-like rate (λ) of 6.96 synonymous substitutions per 10^−9^ years (Moniz de Sá and Drouin, 1996).

### Gene Structure, Motifs, Cis-Elements Analysis, and Homology Modeling

The structure of the *MYBs* genes (exons and introns) was represented using Gene Structure Display Server (GSDS2.0; http://gsds.gao-lab.org/). The conserved motifs in the MYB protein sequences were identified using the Multiple Em for Motif Elicitation (MEME) suite (http://meme-suite.org/tools/meme). The maximum number of motif 40, the minimum width of each motif 6, and the maximum width of 120 were used as parameters. The identified conserved motifs were then confirmed with previously characterized Arabidopsis motifs (Stracke et al., 2001). The *cis*-regulatory elements and motifs in the 1,500 bp (base-pairs) upstream promoter region of *Capsicum MYB* genes were speculated using the PlantCARE website (Lescot et al., 2002). The 126 R2R3 CcMYBs were analyzed for protein tertiary (or 3D; three-dimensional) structure-based homology models using the Phyre2 server (Kelley et al., 2015). The models were predicted based on the alignment coverage, percent identity, and percent confidence score for the individual CcMYB protein sequences.

### Phylogenetic Analysis

The multiple protein sequence alignment of *Capsicum* MYBs was performed using Clustal Omega (Madeira et al., 2019) with default parameters. The phylotree was constructed in MEGAX (v.10.1.8; Kumar et al., 2018) using maximum likelihood (ML) methods with a phylogeny test of 1,000 bootstrap replications and a Jones–Taylor–Thornton (JTT) model with uniform rates among sites applied to infer evolutionary history. The Nearest-Neighbor-Interchange (NNI), as an ML heuristic method, was used for phylogeny tree inference. The combined phylogenetic tree of *C. chinense* and *C. baccatum* R2R3 *MYBs* with already known Arabidopsis *MYBs* (*AtMYBs*) was generated using the above-mentioned parameters. The phylogenetic tree data with bootstrap values were visualized using the Interactive Tree of Life (iTOL) server (https://itol.embl.de/).

### Plant Materials and Growth Conditions

Seeds of *Capsicum* genotypes belonging to highly pungent *C. chinense* (Bhut Jolokia; *Acc-Cc74*; 925084.8 Scoville Heat Unit; SHU) and lowly pungent *C. annuum* (*Acc-Ca18*; 7034.4 SHU) were sown in agro peat and vermiculite (in the proportion 3:1; Sarpras et al., 2019). The seedlings were grown in a glasshouse at 24–26°C temperature with 16-h light and 8-h dark photoperiod and 70% humidity. The 1-month-old plants were transferred into the soil and grown until fruit maturity in the glasshouse at Jawaharlal Nehru University, New Delhi. Fruit tissues of the early green (EG; 5–10 days postanthesis; DPA), mature green (MG; 20–25 DPA), and breaker (Br; 30–45 DPA) stages were harvested and immediately frozen in liquid nitrogen and stored in a deep freezer at −80°C until RNA extraction.

### RNA Sequencing and Differential Gene Expression Analysis

Total RNA from the EG, MG, and Br fruit stages of *C. chinense* (*Acc-Cc74*) and *C. annuum* (*Acc-Ca18*) was extracted using an MN Nucleospin RNA Plant kit (Takara, Mountain View, CA, United States). The integrity of the RNA samples was checked using a bioanalyzer (Agilent Technologies, Santa Clara, CA, United States). The RNA samples from three biological replicates of each fruit stage, with RNA integrity number (RIN) > 8, were used for library preparation using TruSeq RNA Sample Prep Kits (Illumina, San Diego, CA, United States) and sequenced using a HiSeq XTen (Illumina, San Diego, CA, United States) paired-end platform with an average read length of 150 bp. The quality of raw reads was evaluated with FastQC (v0.11.5), and adapter sequences along with low quality reads (phred score <20) were removed using TrimGalore (v0.4.4) as descrbied earlier (Chhapekar et al., 2020; Rawoof et al., 2020). The filtered good quality reads from *C. chinense* and *C. annuum* were mapped to their respective genomes (Qin et al., 2014; Kim et al., 2017) using the Hisat2 (Kim et al., 2019) program. The expression of all genes was estimated using StringTie (v2.0.6; Kim et al., 2019). Read counts of transcripts were quantified using the feature Counts (v1.5.1; Liao et al., 2014). The normalization of raw read counts was performed using TMM methods (Robinson and Oshlack, 2010), and differentially expressed genes (DEGs) between two tissues were identified using the glmQLFit and glmQLFTest functions in the edgeR package (Robinson et al., 2010). Genes with adjusted *p* < 0.01 and fold change (FC) >1.5 were considered as significantly expressed between the two tissues. The normalized expressions and differential expression pattern of *Capsicum MYBs* among fruit tissues were illustrated in the form of a heatmap using gplots (Warnes et al., 2020).

### Expression Analysis of MYB Genes by Quantitative Real-Time (qRT) PCR

A total of 24 *CcMYB* genes showing DE among the fruit developmental stages of *C. chinense* and *C. annuum*, along with *CBG (AT3, KasI, AMT, ACS1, BCAT*, and *COMT*) genes, were validated by qRT-PCR. Gene-specific primers were designed from exonic sequences using standard criteria (Dieffenbach et al., 1993) (Supplementary Table 1). The total RNA was extracted as described above. The quality of RNA was checked on 1% agarose gel, and the quantity was measured using NanoDrop 1000 (Thermo Fisher Scientific, Waltham, MA, United States). The total RNA (1 μg) was then converted into complementary DNA (cDNA) using PrimeScript IV 1st strand cDNA Synthesis Mix (Takara, United States) following the protocol of the manufacturer. The real-time PCR reaction was set up using SYBR Green Mix (Clontech, Mountain View, CA, United States) and run on the CFX96 Real-Time System (Bio-Rad Laboratories, Hercules, CA, United States). The thermal profile included the initial denaturation step (95°C for 2 min) and followed by a 40-cycle amplification step (95°C for 15 s and 60°C for 1 min). For qRT-PCR, three biological replicates of each fruit stage with three technical replicates were used. The relative expression of each gene was calculated using the 2^−Δ*ΔCt*^ method (Livak and Schmittgen, 2001). The *actin* gene was taken as an internal control. The student's *t*-test was performed for calculating significant differences in the expression of *MYB* genes (*p* < 0.05).

### Co-expression Analysis of *MYB* Genes

The co-expression of *Capsicum MYBs* with genes involved in capsaicinoid biosynthesis (*AT3*: *acyltransferase 3*; *Kas*: *ketoacyl-ACP synthase; pAMT: putative aminotransferase; BCKDH: branched-chain a-ketoacid dehydrogenase a-ketoacid decarboxylase; ACL: acyl carrier protein; FatA: acyl-ACP thioesterase*), phenylpropanoid biosynthesis (*PAL: phenylalanine ammonia-lyase; COMT: caffeic acid 3-O-methyltransferase; C4H: cinnamate 4-hydroxylase; 4CL: 4-coumaroyl-CoA ligase; BCAT: branched-chain amino acid aminotransferase*), carotenoid biosynthesis (*CCS: capsanthin-capsorubin synthase; BCH: b-carotene hydroxylase; PSY: phytoene synthase*), anthocyanin biosynthesis genes (*DFR: dihydroflavonol 4-reductase; F3*′*5*′*H: flavonoid 3*′*, 5*′*-hydroxylase; CHS: chalcone synthase*), vitamin C biosynthesis genes (*GLDH: L-galactono-1,4-lactone dehydrogenase; GalDH: L-galactose-1-dehydrogenase; GME: GDP-D-mannose-3*′*,5*′*-epimerase*), and fruit shape and size genes (*WD-40; CLAVATA1; auxin receptor; EAR1; SEC8: Arabidopsis Exocyst Complex; WUSCHEL; TTL3: TPR repeat-containing thioredoxin TTL3; OVATE; SUN*) was represented using a heatmap. The expression of genes was shown as log_2_FPKM, and clustering was performed based on Pearson correlation analysis.

### Synteny and Homologous *MYB* Gene Pairs

Genome-wide CSSs and the collinearity of *C. chinense MYB* genes with *C. annuum, C. baccatum*, tomato, potato, eggplant, and Arabidopsis genomes were identified using the MCScanX toolkit (Wang et al., 2012). Blastp search (e-value <1e-10) results among the total protein sequences of each genome against *C. chinense* total protein sequences were used as input. The whole genome sequences of *C. chinense* (GCA_002271895.2), *C. annuum* (GCA_000710875.1), and *C. baccatum* (GCA_002271885.2) were downloaded from NCBI, and the eggplant genome was downloaded from Solanaceae Genomics Network (https://solgenomics.net/; Barchi et al., 2019). The Arabidopsis, tomato, and potato genome sequences were retrieved from Phytozome v12 (https://phytozome.jgi.doe.gov/pz/portal.html). The CcMYB homologous proteins from the *C. annuum, C. baccatum*, tomato, potato, and Arabidopsis genomes were identified by blastp search with cut-off parameters: e-value <1e-03, percent identity > 75%, and coverage with > 80% of query length. The CSSs were represented using TBtools (v1.068; Chen et al., 2020), and collinear and homologous *MYBs*, along with physical positions on different chromosomes, were presented in a chord diagram using the circlize R package (Gu et al., 2014).

### Simple Sequence Repeat (SSR) Prediction in *Capsicum MYBs*

Full length gene sequences and 1.5-kb upstream of promoter sequences from the Transcription Start Site (TSS) of 251 *C. chinense MYBs* were used to identify simple sequence repeats (SSRs) using the online WebSat tool (Martins et al., 2009) as described previously (Dubey et al., 2019; Jaiswal et al., 2020). The maximum size of an SSR motif was kept at 6 nucleotides, while the minimum number of repeats of the motif was kept at 6. Mononucleotide repeats were excluded from the analysis.

## Results

### Identification of *MYB* Genes in *Capsicum* Spp

After the duplicate sequences were removed from the blastp search results and Hidden Markov Model (HMM) analysis, a total of 301, 433, and 292 potential *MYB-*encoding genes were predicted in the *C. chinense, C. annuum*, and *C. baccatum* genomes, respectively. The remaining sequences were screened using CDD and the SMART database to ascertain the presence of the MYB domain. Ultimately, a total of 251, 245, and 240 *MYB*-encoding genes were identified in *C. chinense, C. annuum*, and *C. baccatum* genomes, respectively. In the *C. chinense* genome, out of the total *CcMYBs*, 126 were R2R3 type, while the remaining 99 were MYB1R type, 25 belonged to MYB with other domains type, and 1 was atypical MYB. A total of 128 *C. annuum MYB* (*CaMYB*) and 123 *C. baccatum MYB* (*CbMYB*) were of R2R3-type. The molecular weight of CcMYBs varied from 11.5 to 114.6 kDa (kilodalton), and that of MYB related from 8.8 to 183.5 kDa. Most of the *CcMYBs* were localized in the nucleus, seven in mitochondria, two in the chloroplast, two in the cytoplasm, and one in extracellular (Supplementary Table 2). The coding (CDS) and protein sequences of *CcMYB* genes are given in Supplementary File 1.

### Genome-Wide Distribution, Duplication, and Co-mapping of *Capsicum MYBs* With Capsaicinoid QTLs

**Figure 1:**
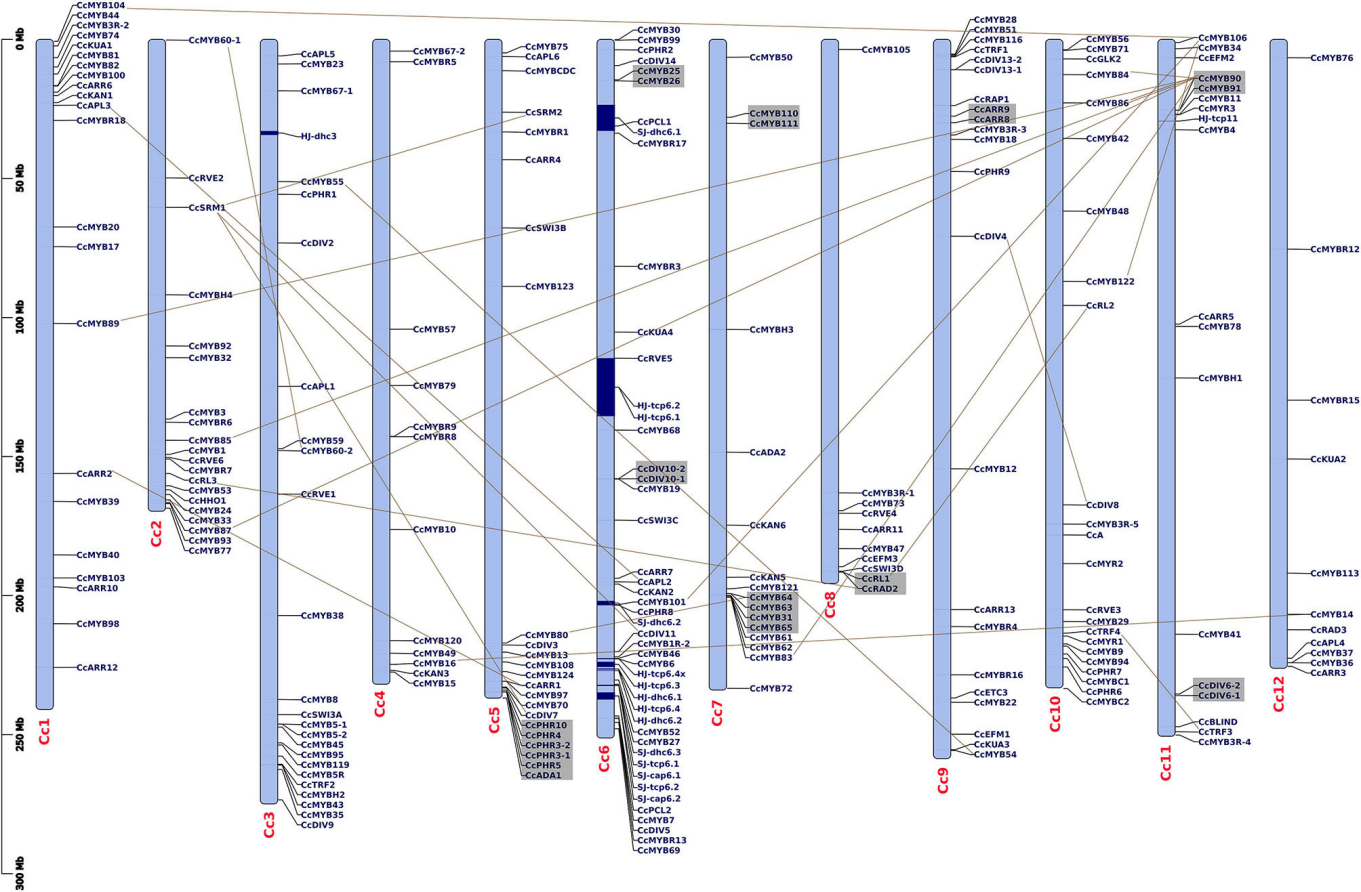
Distribution of *CcMYB* genes on 12 *Capsicum* chromosomes along with the previously reported capsaicinoid QTLs (Han et al., 2018; Park et al., 2019). The QTLs are shown in blue blocks. The tandem duplicates are shown *via* gray blocks, while the segmental duplicates are shown *via* connecting lines. The scale is mentioned on the left of the chromosomes in million base pairs (Mbp).

Of all the *MYB* genes identified, 232 (92.43%) were physically mapped on 12 chromosomes (chrs), and the remaining were mapped on scaffolds of the *C. chinense* genome (Figure 1). In *C. annuum*, 222 (90.61%) and in *C. baccatum* 221 (92.08%) *MYBs* were mapped on their respective chrs (Supplementary Figures 1A,B). Uneven distribution of *MYB* genes on the 12 *Capsicum* chrs (average 11–12/ chr) was observed. In *C. chinense*, the upper end of the arm on chr1 and the lower end of the arm on chrs 2 and 6, respectively, have a greater density of *MYBs*. A similar distribution of *MYBs* was observed for chrs 1 and 2 in both *C. annuum* and *C. baccatum* and for chr 6 in *C. baccatum*.

In the *C. chinense* genome, nine clusters of 20 tandem and 41 segmental duplicated *CcMYB* gene pairs were observed with *Ka/Ks* ratios ranging from 0.001 to 1.0269 (Table 1). Among the duplicate pairs, *CcMYB60-1* and *CcMYB60-2* had the highest *Ka/Ks* ratio (1.0269) followed by *CcPHR10* and *CcPHR3-2* (0.8988), *CcDIV6-2* and *CcDIV6-1* (0.8396), and *CcARR9* and *CcARR8* (0.7781). *Ka/Ks* values of >1 indicate a positive selection, while a *Ka/Ks* ratio of <1 indicates purifying selection for these *MYB* gene pairs in the *C. chinense* genome. The minimum diversion time was 0.2414 MYA for the gene pair duplicated in tandem, *CcGLK1-1* and *CcGLK1-2*, while it was highest between the *CcMYB106* and *CcMYB104* segmental duplicated pair, i.e., 309.78 MYA.

**Table 1:** *MYB* gene duplicates in the *Capsicum chinense* genome.

***C. chinense MYB* name**	**Duplicated *MYB* pair**	**Duplication type**	**Ka**	**Ks**	**Ka/Ks**	**Diversion time (T = Ks/2λ) MYA**
*CcAPL3*	*CcAPL2*	Segmental	0.131	0.526	0.248	37.77
*CcARR1*	*CcARR2*	Segmental	0.167	0.834	0.200	59.92
*CcARR9*	*CcARR8*	Tandem	0.092	0.118	0.778	8.50
*CcDIV10-1*	*CcDIV10-2*	Tandem	0.078	0.117	0.668	8.43
*CcDIV11*	*CcSRM1*	Segmental	0.427	3.501	0.122	251.48
*CcDIV4*	*CcDIV8*	Segmental	0.164	1.008	0.163	72.43
*CcDIV6-2*	*CcDIV6-1*	Tandem	0.091	0.108	0.840	7.77
*CcDIV7*	*CcSRM1*	Segmental	0.456	2.903	0.157	208.54
*CcGLK1-1*	*CcGLK1-2*	Segmental	0.000	0.003	0.001	0.24
	*CcGLK1-3*	Segmental	0.000	0.003	0.001	0.24
	*CcGLK1-4*	Segmental	0.000	0.003	0.001	0.24
*CcGLK1-2*	*CcGLK1-3*	Segmental	NA	NA	NA	–
	*CcGLK1-4*	Segmental	NA	NA	NA	–
*CcGLK1-4*	*CcGLK1-3*	Segmental	NA	NA	NA	–
*CcMYB102*	*CcMYB122*	Segmental	0.235	1.185	0.198	85.10
*CcMYB106*	*CcMYB102*	Segmental	0.254	4.157	0.061	298.60
	*CcMYB101*	Segmental	0.346	3.527	0.098	253.35
	*CcMYB122*	Segmental	0.334	4.018	0.083	288.64
	*CcMYB104*	Segmental	0.336	4.312	0.078	309.78
*CcMYB110*	*CcMYB111*	Tandem	0.014	0.083	0.164	5.94
*CcMYB115*	*CcA*	Segmental	0.369	0.993	0.372	71.32
*CcMYB14*	*CcMYB16*	Segmental	0.396	2.037	0.195	146.36
*CcMYB25*	*CcMYB26*	Tandem	0.035	0.136	0.261	9.74
*CcMYB31*	*CcMYB63*	Tandem	0.137	0.456	0.300	32.76
	*CcMYB65*	Tandem	0.184	0.529	0.349	37.99
*CcMYB32*	*CcMYB33*	Segmental	0.134	0.783	0.172	56.24
*CcMYB55*	*CcMYB54*	Segmental	0.233	2.843	0.082	204.22
*CcMYB60-1*	*CcMYB60-2*	Segmental	0.061	0.060	1.027	4.29
*CcMYB64*	*CcMYB31*	Tandem	0.090	0.334	0.269	23.98
	*CcMYB63*	Tandem	0.115	0.562	0.204	40.35
	*CcMYB80*	Segmental	0.281	3.748	0.075	269.24
*CcMYB90*	*CcMYB91*	Tandem	0.052	0.263	0.198	18.93
	*CcMYB87*	Segmental	0.172	3.742	0.046	268.83
	*CcMYB89*	Segmental	0.156	0.985	0.159	70.74
	*CcMYB86*	Segmental	0.347	2.739	0.127	196.74
	*CcMYB85*	Segmental	0.329	3.449	0.096	247.77
	*CcMYB83*	Segmental	0.359	3.568	0.101	256.29
	*CcMYB84*	Segmental	0.381	2.282	0.167	163.91
*CcMYBC2*	*CcMYBC1*	Segmental	0.158	1.221	0.130	87.75
*CcMYBH1*	*CcMYBH5*	Segmental	0.181	0.534	0.339	38.39
*CcMYBR10*	*CcMYBR11*	Tandem	0.046	0.070	0.655	4.99
*CcMYR1*	*CcMYR2*	Segmental	0.167	0.520	0.322	37.35
*CcPHR10*	*CcPHR3-2*	Tandem	0.196	0.218	0.899	15.69
	*CcPHR4*	Tandem	0.172	0.264	0.651	18.96
*CcPHR10*	*CcPHR3-1*	Tandem	0.240	0.333	0.721	23.94
*CcPHR3-1*	*CcPHR3-2*	Tandem	0.052	0.098	0.528	7.03
*CcPHR4*	*CcPHR3-2*	Tandem	NA	NA	NA	–
	*CcPHR3-1*	Tandem	0.044	0.107	0.415	7.69
	*CcPHR5*	Tandem	0.085	0.138	0.619	9.89
*CcRAD3*	*CcRAD6*	Segmental	0.144	1.636	0.088	117.50
	*CcRAD4*	Segmental	0.288	3.758	0.077	269.94
	*CcRAD5*	Segmental	0.213	2.091	0.102	150.25
*CcRAD4*	*CcRAD6*	Segmental	0.188	3.621	0.052	260.12
	*CcRAD3*	Segmental	0.288	3.758	0.077	269.94
*CcRAD5*	*CcRAD6*	Tandem	0.078	0.143	0.549	10.25
	*CcRAD3*	Segmental	0.213	2.091	0.102	150.25
*CcRL1*	*CcRAD2*	Tandem	0.418	2.159	0.194	155.10
*CcRL2*	*CcRL1*	Segmental	0.210	1.744	0.120	125.30
*CcRL3*	*CcRAD2*	Segmental	0.225	4.043	0.056	290.42
*CcSRM1*	*CcSRM2*	Segmental	0.464	1.053	0.440	75.63
*CcTRF3*	*CcTRF4*	Segmental	0.066	0.107	0.617	7.70
*CcDIV7*	*CcSRM1*	Segmental	0.456	2.903	0.157	208.54
*CcGLK1-1*	*CcGLK1-2*	Segmental	0.000	0.003	0.001	0.24
	*CcGLK1-3*	Segmental	0.000	0.003	0.001	0.24
	*CcGLK1-4*	Segmental	0.000	0.003	0.001	0.24
*CcGLK1-2*	*CcGLK1-3*	Segmental	NA	NA	NA	–
	*CcGLK1-4*	Segmental	NA	NA	NA	–
*CcGLK1-4*	*CcGLK1-3*	Segmental	NA	NA	NA	–
*CcMYB102*	*CcMYB122*	Segmental	0.235	1.185	0.198	85.10
*CcMYB106*	*CcMYB102*	Segmental	0.254	4.157	0.061	298.60
	*CcMYB101*	Segmental	0.346	3.527	0.098	253.35
	*CcMYB122*	Segmental	0.334	4.018	0.083	288.64
	*CcMYB104*	Segmental	0.336	4.312	0.078	309.78
*CcMYB110*	*CcMYB111*	Tandem	0.014	0.083	0.164	5.94
*CcMYB115*	*CcA*	Segmental	0.369	0.993	0.372	71.32
*CcMYB14*	*CcMYB16*	Segmental	0.396	2.037	0.195	146.36
*CcMYB25*	*CcMYB26*	Tandem	0.035	0.136	0.261	9.74
*CcMYB31*	*CcMYB63*	Tandem	0.137	0.456	0.300	32.76
	*CcMYB65*	Tandem	0.184	0.529	0.349	37.99
*CcMYB32*	*CcMYB33*	Segmental	0.134	0.783	0.172	56.24
*CcMYB55*	*CcMYB54*	Segmental	0.233	2.843	0.082	204.22
*CcMYB60-1*	*CcMYB60-2*	Segmental	0.061	0.060	1.027	4.29
*CcMYB64*	*CcMYB31*	Tandem	0.090	0.334	0.269	23.98
	*CcMYB63*	Tandem	0.115	0.562	0.204	40.35
	*CcMYB80*	Segmental	0.281	3.748	0.075	269.24
*CcMYB90*	*CcMYB91*	Tandem	0.052	0.263	0.198	18.93
	*CcMYB87*	Segmental	0.172	3.742	0.046	268.83
	*CcMYB89*	Segmental	0.156	0.985	0.159	70.74
	*CcMYB86*	Segmental	0.347	2.739	0.127	196.74
	*CcMYB85*	Segmental	0.329	3.449	0.096	247.77
	*CcMYB83*	Segmental	0.359	3.568	0.101	256.29
	*CcMYB84*	Segmental	0.381	2.282	0.167	163.91
*CcMYBC2*	*CcMYBC1*	Segmental	0.158	1.221	0.130	87.75
*CcMYBH1*	*CcMYBH5*	Segmental	0.181	0.534	0.339	38.39
*CcMYBR10*	*CcMYBR11*	Tandem	0.046	0.070	0.655	4.99
*CcMYR1*	*CcMYR2*	Segmental	0.167	0.520	0.322	37.35
*CcPHR10*	*CcPHR3-2*	Tandem	0.196	0.218	0.899	15.69
	*CcPHR4*	Tandem	0.172	0.264	0.651	18.96
*CcPHR10*	*CcPHR3-1*	Tandem	0.240	0.333	0.721	23.94
*CcPHR3-1*	*CcPHR3-2*	Tandem	0.052	0.098	0.528	7.03
*CcPHR4*	*CcPHR3-2*	Tandem	NA	NA	NA	–
	*CcPHR3-1*	Tandem	0.044	0.107	0.415	7.69
	*CcPHR5*	Tandem	0.085	0.138	0.619	9.89
*CcRAD3*	*CcRAD6*	Segmental	0.144	1.636	0.088	117.50
	*CcRAD4*	Segmental	0.288	3.758	0.077	269.94
	*CcRAD5*	Segmental	0.213	2.091	0.102	150.25
*CcRAD4*	*CcRAD6*	Segmental	0.188	3.621	0.052	260.12
	*CcRAD3*	Segmental	0.288	3.758	0.077	269.94
*CcRAD5*	*CcRAD6*	Tandem	0.078	0.143	0.549	10.25
	*CcRAD3*	Segmental	0.213	2.091	0.102	150.25
*CcRL1*	*CcRAD2*	Tandem	0.418	2.159	0.194	155.10
*CcRL2*	*CcRL1*	Segmental	0.210	1.744	0.120	125.30
*CcRL3*	*CcRAD2*	Segmental	0.225	4.043	0.056	290.42
*CcSRM1*	*CcSRM2*	Segmental	0.464	1.053	0.440	75.63
*CcTRF3*	*CcTRF4*	Segmental	0.066	0.107	0.617	7.70
	*CcMYB104*	Segmental	0.336	4.312	0.078	309.78
*CcMYB110*	*CcMYB111*	Tandem	0.014	0.083	0.164	5.94
*CcMYB115*	*CcA*	Segmental	0.369	0.993	0.372	71.32
*CcMYB14*	*CcMYB16*	Segmental	0.396	2.037	0.195	146.36
*CcMYB25*	*CcMYB26*	Tandem	0.035	0.136	0.261	9.74
*CcMYB31*	*CcMYB63*	Tandem	0.137	0.456	0.300	32.76
	*CcMYB65*	Tandem	0.184	0.529	0.349	37.99
*CcMYB32*	*CcMYB33*	Segmental	0.134	0.783	0.172	56.24
*CcMYB55*	*CcMYB54*	Segmental	0.233	2.843	0.082	204.22
*CcMYB60-1*	*CcMYB60-2*	Segmental	0.061	0.060	1.027	4.29
*CcMYB64*	*CcMYB31*	Tandem	0.090	0.334	0.269	23.98
	*CcMYB63*	Tandem	0.115	0.562	0.204	40.35
	*CcMYB80*	Segmental	0.281	3.748	0.075	269.24
*CcMYB90*	*CcMYB91*	Tandem	0.052	0.263	0.198	18.93
	*CcMYB87*	Segmental	0.172	3.742	0.046	268.83
	*CcMYB89*	Segmental	0.156	0.985	0.159	70.74
	*CcMYB86*	Segmental	0.347	2.739	0.127	196.74
	*CcMYB85*	Segmental	0.329	3.449	0.096	247.77
	*CcMYB83*	Segmental	0.359	3.568	0.101	256.29
	*CcMYB84*	Segmental	0.381	2.282	0.167	163.91
*CcMYBC2*	*CcMYBC1*	Segmental	0.158	1.221	0.130	87.75
*CcMYBH1*	*CcMYBH5*	Segmental	0.181	0.534	0.339	38.39
*CcMYBR10*	*CcMYBR11*	Tandem	0.046	0.070	0.655	4.99
*CcMYR1*	*CcMYR2*	Segmental	0.167	0.520	0.322	37.35
*CcPHR10*	*CcPHR3-2*	Tandem	0.196	0.218	0.899	15.69
	*CcPHR4*	Tandem	0.172	0.264	0.651	18.96
*CcPHR10*	*CcPHR3-1*	Tandem	0.240	0.333	0.721	23.94
*CcPHR3-1*	*CcPHR3-2*	Tandem	0.052	0.098	0.528	7.03
*CcPHR4*	*CcPHR3-2*	Tandem	NA	NA	NA	–
	*CcPHR3-1*	Tandem	0.044	0.107	0.415	7.69
	*CcPHR5*	Tandem	0.085	0.138	0.619	9.89
*CcRAD3*	*CcRAD6*	Segmental	0.144	1.636	0.088	117.50
	*CcRAD4*	Segmental	0.288	3.758	0.077	269.94
	*CcRAD5*	Segmental	0.213	2.091	0.102	150.25
*CcRAD4*	*CcRAD6*	Segmental	0.188	3.621	0.052	260.12
	*CcRAD3*	Segmental	0.288	3.758	0.077	269.94
*CcRAD5*	*CcRAD6*	Tandem	0.078	0.143	0.549	10.25
	*CcRAD3*	Segmental	0.213	2.091	0.102	150.25
*CcRL1*	*CcRAD2*	Tandem	0.418	2.159	0.194	155.10
*CcRL2*	*CcRL1*	Segmental	0.210	1.744	0.120	125.30
*CcRL3*	*CcRAD2*	Segmental	0.225	4.043	0.056	290.42
*CcSRM1*	*CcSRM2*	Segmental	0.464	1.053	0.440	75.63
*CcTRF3*	*CcTRF4*	Segmental	0.066	0.107	0.617	7.70
	*CcPHR3-1*	Tandem	0.044	0.107	0.415	7.69
	*CcPHR5*	Tandem	0.085	0.138	0.619	9.89
*CcRAD3*	*CcRAD6*	Segmental	0.144	1.636	0.088	117.50
	*CcRAD4*	Segmental	0.288	3.758	0.077	269.94
	*CcRAD5*	Segmental	0.213	2.091	0.102	150.25
*CcRAD4*	*CcRAD6*	Segmental	0.188	3.621	0.052	260.12
	*CcRAD3*	Segmental	0.288	3.758	0.077	269.94
*CcRAD5*	*CcRAD6*	Tandem	0.078	0.143	0.549	10.25
	*CcRAD3*	Segmental	0.213	2.091	0.102	150.25
*CcRL1*	*CcRAD2*	Tandem	0.418	2.159	0.194	155.10
*CcRL2*	*CcRL1*	Segmental	0.210	1.744	0.120	125.30
*CcRL3*	*CcRAD2*	Segmental	0.225	4.043	0.056	290.42
*CcSRM1*	*CcSRM2*	Segmental	0.464	1.053	0.440	75.63
*CcTRF3*	*CcTRF4*	Segmental	0.066	0.107	0.617	7.70

*The type of duplication, Ka (rate of non-synonymous substitution per non-synonymous site), Ks (rate of synonymous substitution per synonymous site), Ka/Ks ratio, and the duplication time in million years ago (MYA) are mentioned (T = Ks/2λ; where λ = 6.96 × 10^−9^ years)*.

Our analysis showed at least seven *MYB* genes (five *CcMYBs* and two *CaMYBs*) within the previously reported capsaicinoid QTLs (Han et al., 2018; Park et al., 2019) (Figure 1). At chr 6 of *C. chinense, CcMYB101* and *CcPHR8* are located within the dihydrocapsaicin QTL *SJ-dhc6.2*; *CcMYB46* and *CcMYB6* within the capsaicinoid QTL *HJ-tcp6.4;* and *CcRVE5* within capsaicinoid QTLs *HJ-tcp6.1* and *HJ-tcp6.2*. At chr 2 of *C. annuum, CaMYB3* and *CaHHO1* are located within the two QTLs, i.e., a dihydrocapsaicin QTL *PD-dicap2.2* and total capsaicinoid QTL *PD-total2* (Supplementary Figure 1A).

### Gene Structure, Motif, and Cis-Element Analysis

**Figure 2:**
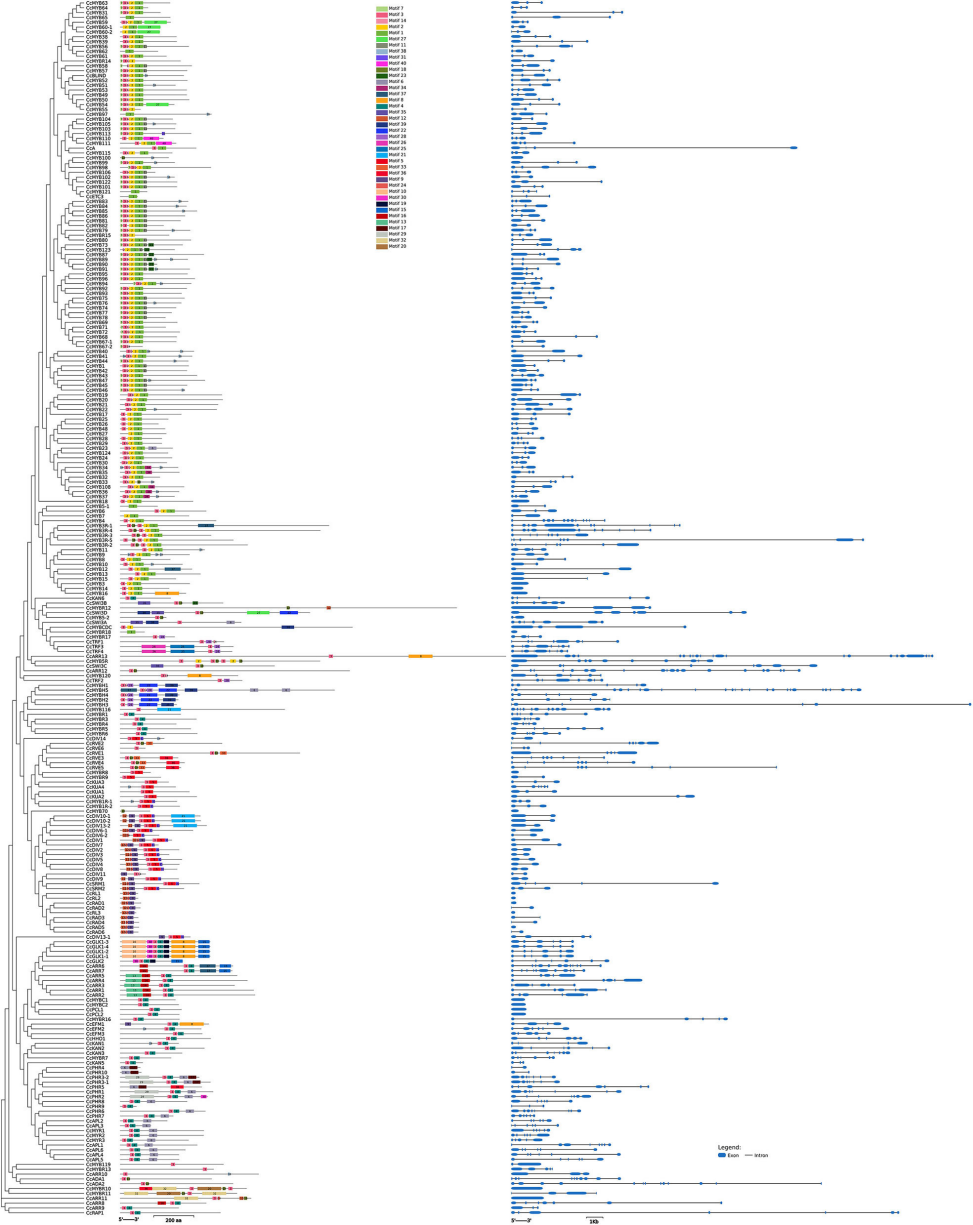
Conserved amino acid motifs and structure of *CcMYB* genes. The motifs are differently colored with a specific number. The exons are shown in blue blocks, while the connecting lines in between represent intronic regions in kb (kilobase).

**Figure 3:**
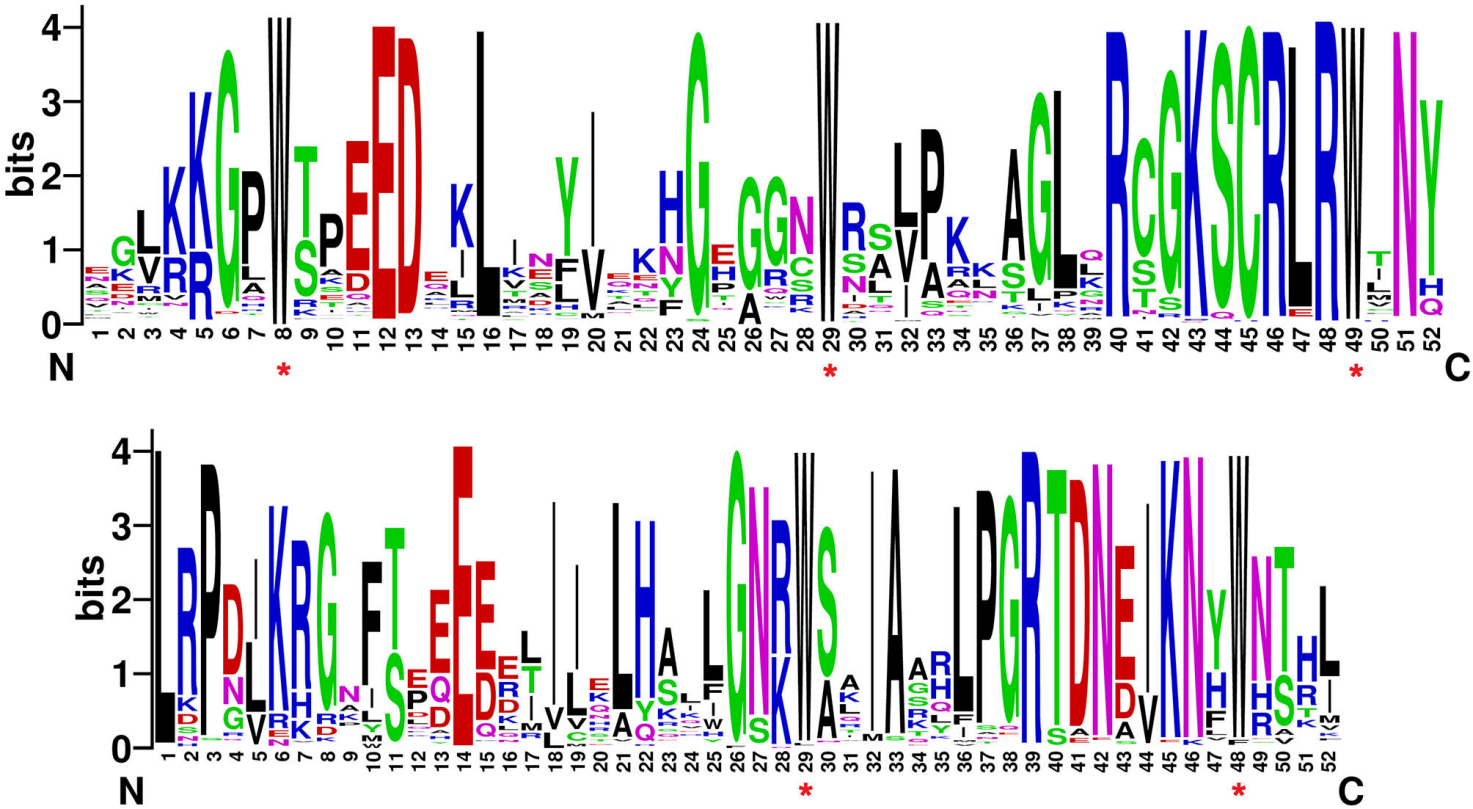
Sequence logo of **(A)** R2 and **(B)** R3 MYB repeats in *C. chinense*.

Most of the *MYB* genes, i.e., 60.95% in *C. chinense*, 61.22% in *C. annuum*, and 60.41% in *C. baccatum*, have two to three exons (Figure 2 and Supplementary Figure 2), and very few showed 11 or more exons. The motif analysis of CcMYB proteins revealed that in the R2 MYB domain, 18 (out of 52) amino acid (aa) positions were conserved in 80% of the R2R3-MYB protein sequences (Figure 3). However, 20 (out of 52) in the R3 domain were conserved, suggesting that R3 is relatively more conserved. The number and placement of tryptophan residues were found to be highly conserved; three tryptophan residues placed 20–21 residues apart in the R2 domain, and two tryptophan residues placed 19 residues apart in the R3 domain. The phenylalanine residue, which replaces the first tryptophan residue in the R3 repeat, is also found to be highly conserved. Apart from the MYB repeats, conserved motifs were observed on the C terminal of the MYB protein sequences (Supplementary Table 3). Previously identified motifs in *AtMYBs* were also identified in *CcMYBs*; for instance, motif-23 ([TY][SV]AN[LA][SR]HMA[QE]WESARLEAEARL[VS]R[EK]S[KQ]), which has previously been defined in *Antirrhinum majus* MIXTA MYB, was observed in CcMYB89, CcMYB90, CcMYB91, and CcMYB87 (Kranz et al., 1998) protein sequences. The putative *cis-*elements were also identified in *C. chinense, C. annuum*, and *C. baccatum MYBs* (Supplementary Table 4). Both elements binding to basic transcriptional machineries like the TATA and CAAT boxes (Forde et al., 1985) and cis-elements like hormone-responsive ABRE (ABA-Responsive Element; Yamaguchi-Shinozaki and Shinozaki, 1994); seed-specific like RY-element (Fujiwara and Beachy, 1994), AE-Box (Sevilla-Lecoq et al., 2003), AACA motif (Yoshihara et al., 1996), GCN4 motif (Müller and Knudsen, 1993) and Box II (Kim and Wu, 1990); light-responsive elements like MRE (Safrany et al., 2008), G-box (Schindler et al., 1992), GATA-motif (Reyes et al., 2004); low temperature-responsive LTRE (Dunn et al., 1994); and drought- and stress-responsive TC-rich repeats, were identified.

### Phylogenetic Analysis of *Capsicum MYBs*

**Figure 4:**
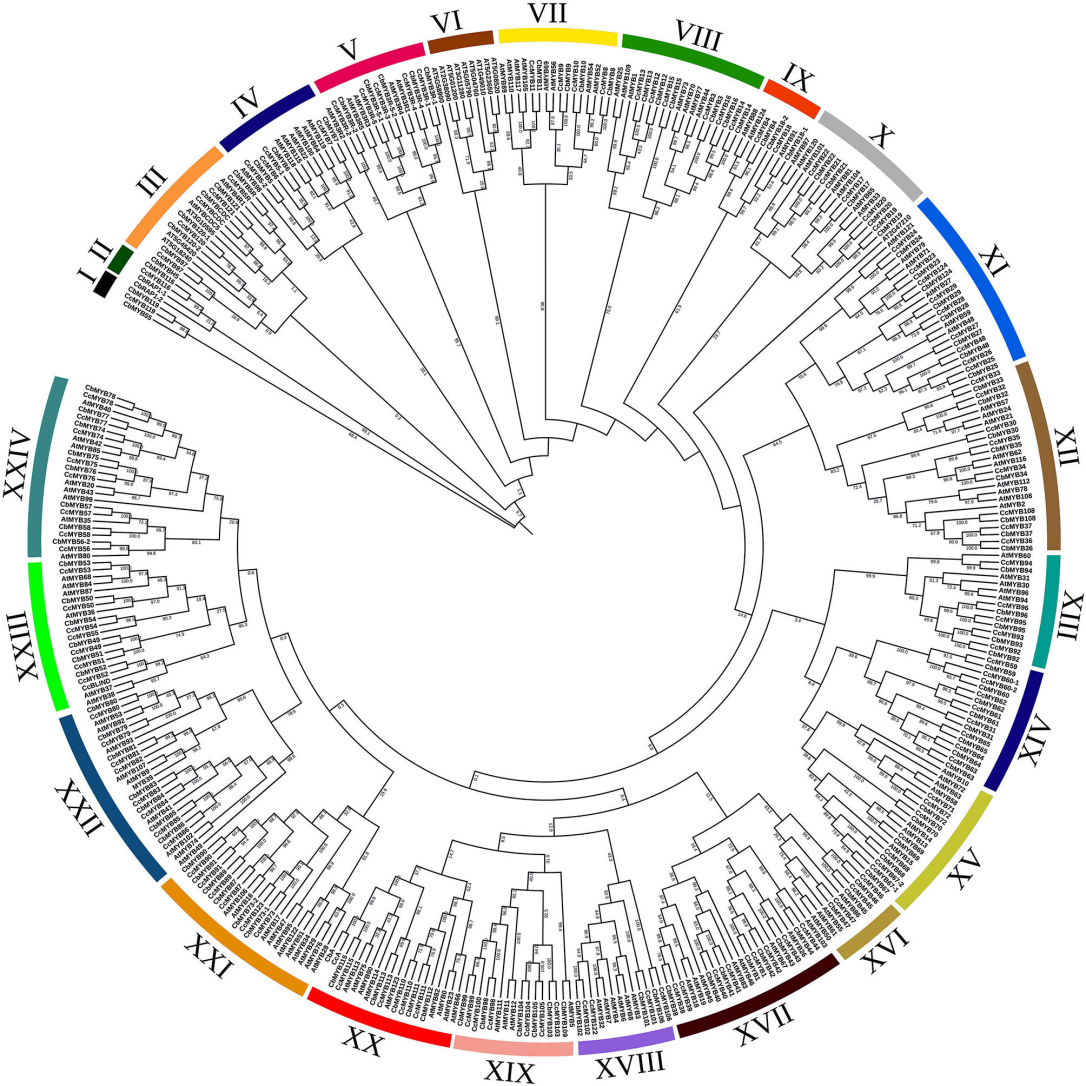
Phylogenetic tree constructed using R2R3 MYBs from *C. chinense, C. baccatum*, and Arabidopsis. The tree was divided into 24 subgroups, and bootstrap values are shown on the branches of the tree.

In the phylogenetic analysis, 126 *CcMYBs*, 123 *CbMYBs*, and 147 *AtMYBs* clustered into 24 subgroups (Figure 4) with >75% confidence in most of the branches, for instance, nearly 100% confidence between clades of *CcMYB97* and *CbMYB97* in subgroup III, *CcMYB7* and *CbMYB7* in subgroup IV, and *AtMYB121, CcMYB24*, and *CbMYB24* in subgroup XI were observed. Similarly, subgroup VIII MYBs like *CcMYB16, CcMYB13*, and *CcMYB14* share 99–100% confidence in their clades with *CbMYB16, CbMYB13*, and *CbMYB14*, respectively. However, *CbMYB18-1* in subgroup IX shares a sister clade with *AtMYB91* with only 51.6% confidence. MYB3Rs (subgroup V) formed a separate subgroup but share a distant common ancestor with *R2R3MYBs*, suggesting their common origin. Interestingly, *Capsicum MYBs*, such as *MYB31, MYB59, MYB60, MYB62, MYB63, MYB64*, and *MYB65*, were segregated separately in subgroup XIV. The phylogenetic tree constructed from a total of 734 MYB protein sequences from three *Capsicum* species resolved into 22 subgroups (Supplementary Figure 3). Several *MYB* genes, such as *CcMYBR14, CcMYBR17*, and *CbLHY*, clustered alone with no recent sister clade in the other two *Capsicum* species.

### Expression Analysis of *MYB* Genes

**Figure 5:**
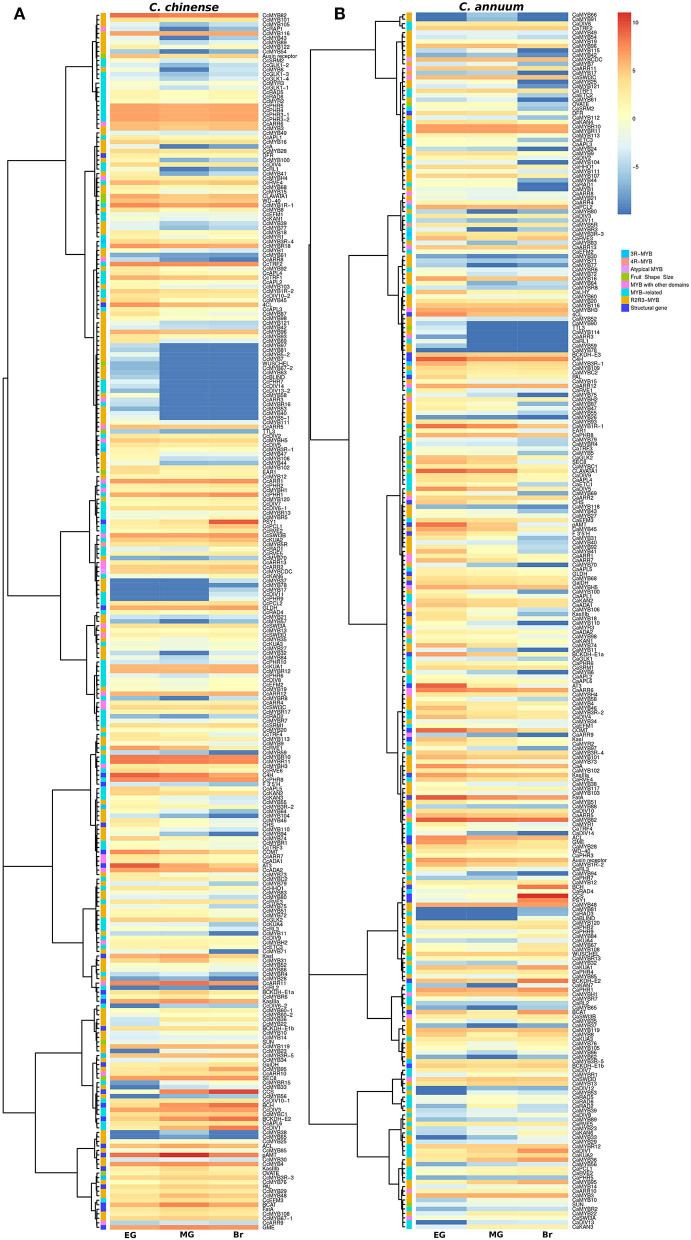
Expression profiles of *MYB* genes in **(A)** *C. chinense*, **(B)** *C. annuum* at the three different fruit developmental stages. The heatmap color scale represents the log2 fragments per kilobase of transcript per Million mapped reads (FPKM) expression of *MYB* genes.

**Figure 6:**
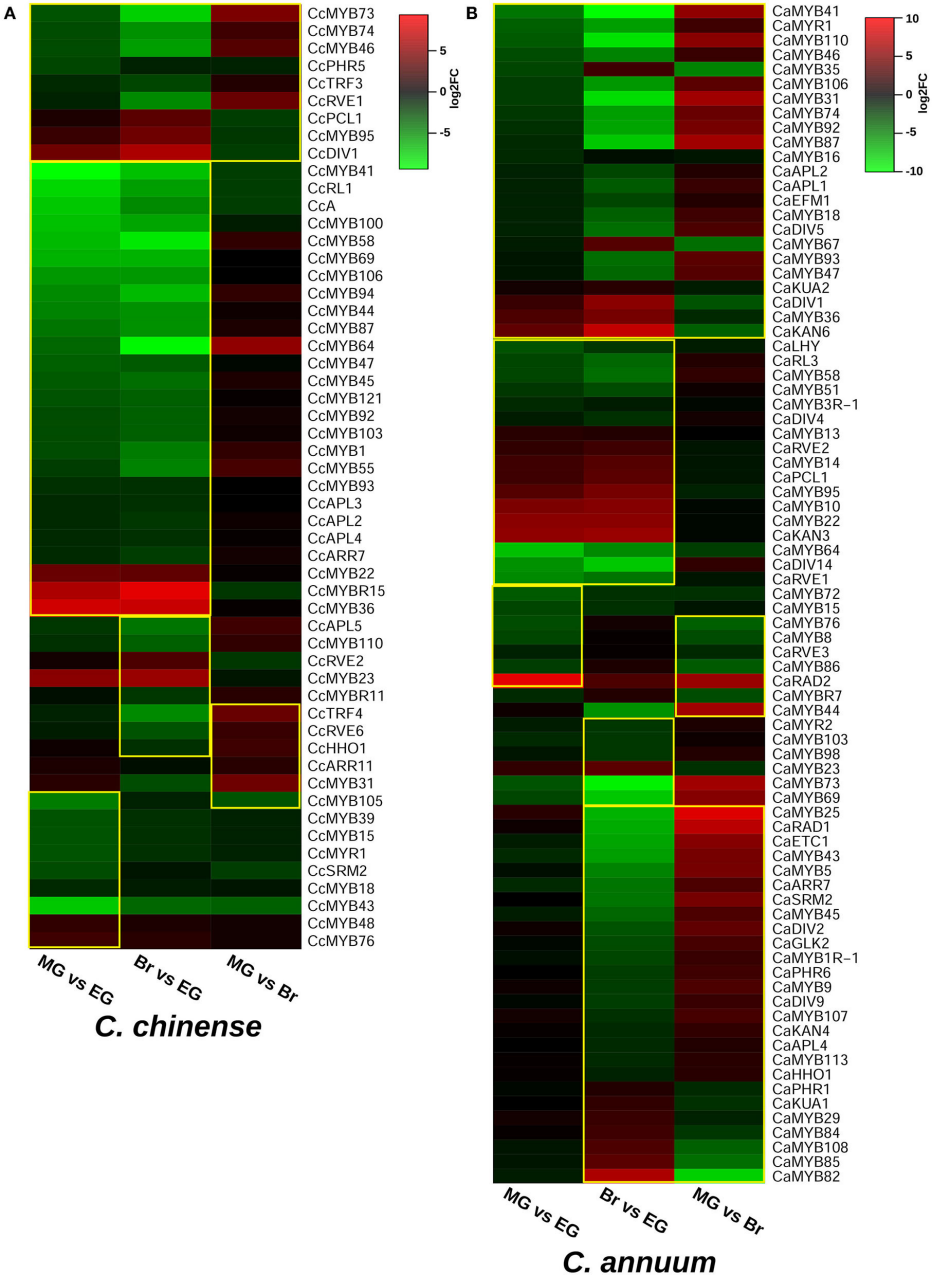
Differentially expressed *MYB* genes in **(A)** *C. chinense* and **(B)** *C. annuum*. Significant DEG *MYBs* are enclosed within the yellow box.

**Figure 7:**
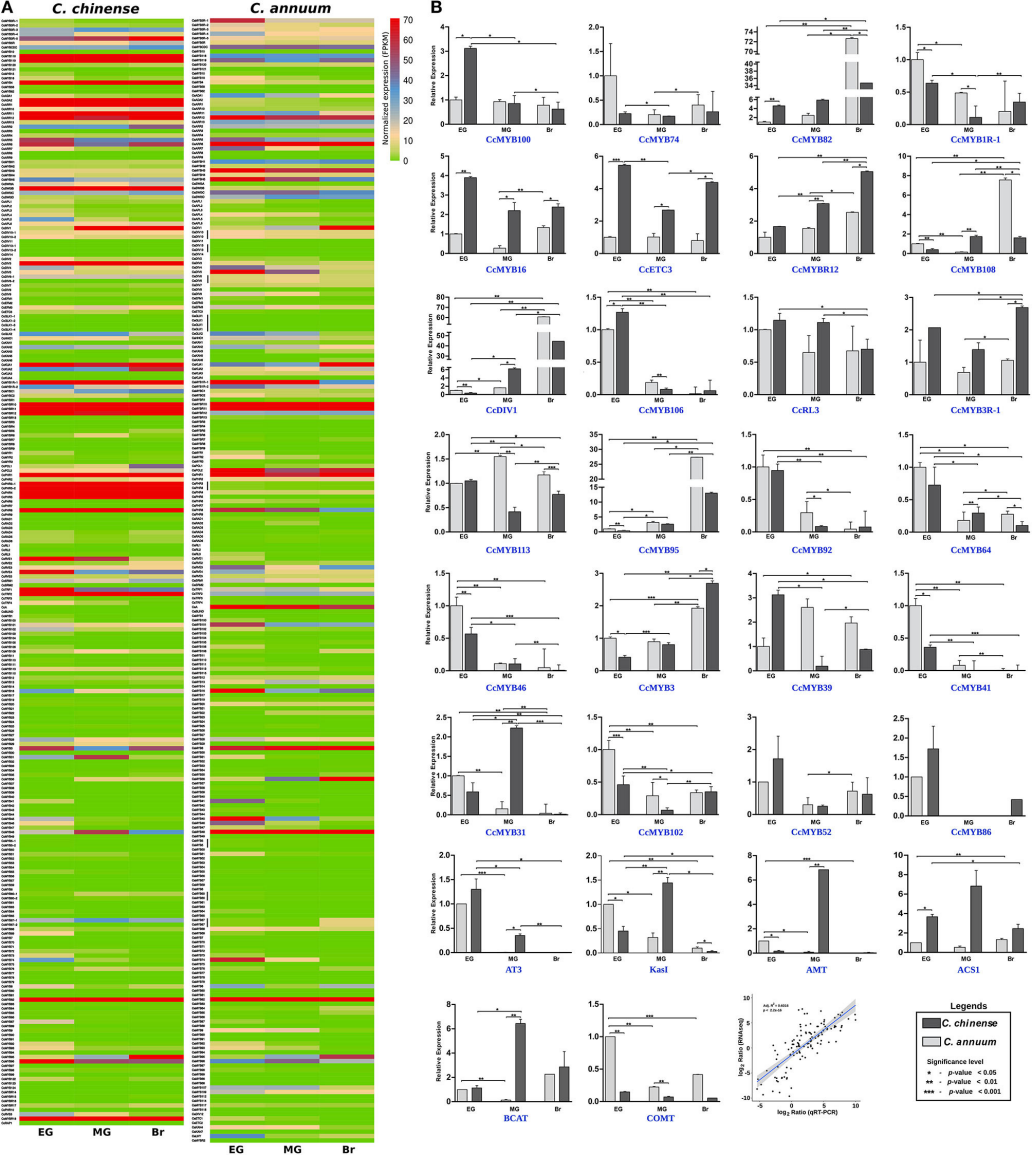
Ribonucleic acid sequencing (RNAseq) and quantitative real-time (qRT)-PCR expression data of *Capsicum MYBs* at the early green (EG), mature green (MG), and breaker (Br) fruit development stages. **(A)** Normalized FPKM expression of *C. chinense MYBs* and their *C. annuum* homologs by RNAseq, and **(B)** relative expression of 24 *CcMYB* genes and capsaicinoid biosynthesis pathway genes (*AT3, KasI, pAMT, ACS1, BCAT*, and *COMT*) between *C. chinense* (Bhut Jolokia; Acc-Cc74) and *C. annuum* (Acc-Ca18) by qRT-PCR in the three fruit developmental stages and correlation between RNAseq and qRT-PCR expression data. A Student *t*-test was performed to calculate the significant difference of expression. The significance level was represented as ***p < 0.001; **p < 0.01, and *p < 0.05).

The transcriptome data of the EG, MG, and Br fruit development stages of *C. chinense* and *C. annuum* were used to determine the expression values of all the *MYB* genes based on the normalized FPKM (fragments per kilobase of transcript per million mapped reads). A total of ~458 million raw paired-end reads were generated from the fruit tissues of *C. chinense* and *C. annuum*. Around 196.8 and 200.9 million reads out of ~237.4 and 220.8 million clean reads were aligned with an average alignment rate of 82.9 and 91.02% against the *C. chinense* and *C. annuum* genomes (Qin et al., 2014; Kim et al., 2017), respectively (Supplementary Table 5). We observed a variation in the expression of *MYB* genes among the different fruit developmental stages in *C. annuum* and *C. chinense*. A total of 236 and 238 *MYB* genes were expressed in at least one tissue in *C. chinense* and *C. annuum*, respectively. Based on the expression patterns of these genes in the fruit developmental stages, the co-expression analysis identified 15 and 20 different clusters of *MYB* genes in *C. chinense* and *C. annuum*, respectively (Figure 5). Also, several *MYB* genes, such as *CcMYB16, CcMYB28, CcMYB100, CcA, CcDIV4, CcMYB46*, and *CcMYB74*, were co-expressed with *DFR* and *CHS* from the anthocyanin/flavonoid pathway, while *CcDIV1, CcMYB4, CcMYB31, CcMYB52, CcMYB86, CcMYB108, CcMYBR6*, and *CcARR11* were co-expressed with *Kas, FatA*, and *BCKDH* from the capsaicinoid biosynthesis pathway. Moreover, the *CcMYB10, CcMYB82, CcMYB102, CcMYB1R1*, and *CcRVE4* genes showed similar expression patterns with genes related to fruit shape and size (Figure 5). Further analysis revealed a total of 54 DE *CcMYBs* (adjusted *p* < 0.01) in *C. chinense*, 36 in MG compared with EG (12 upregulated and 24 downregulated), 66 in Br compared with EG (20 upregulated and 46 downregulated.), and 50 in MG compared with Br (34 upregulated and 16 downregulated; Figure 6). While a total of 81 *CaMYBs* were DEGs (adjusted *p* < 0.01) in *C. annuum*, 39 and 42 were DEs in MG and Br compared with the EG fruit stage and 13 were DEGs in MG with respect to the Br fruit stage (Figure 6). Furthermore, we analyzed the expression patterns of *MYB* genes in *C. chinense* and *C. annuum* using the transcriptome data and compared them using representative heatmaps (Figure 7A). Most of the MYB genes showed similar expression patterns, but several MYB genes showed contrasting expression levels in the two *Capsicum* spp. For instance, *CcMYBR12* shows a higher expression in *C. chinense* and a moderate expression in *C. annuum* in all three fruit stages. *CcPHR8* is highly expressed throughout the fruit developmental stages in *C. chinense*, while its expression decreases from high to low during fruit development in *C. annuum*. *CcMYB31* is exclusively highly expressed in the MG stage in *C. chinense*, moderately expressed in EG, and lowly expressed in the Br fruit stage, while its homolog in *C. annuum* showed a low expression in the EG stage and a negligible expression in the rest of the two stages. *MYB48* showed a higher expression throughout the fruit developmental stages in *C. annuum* but only slightly higher in MG in *C. chinense* than the rest of the fruit stages.

The expression of 24 MYB genes showing DE in transcriptome data was validated by qRT-PCR analysis (Figure 7B). The *MYB* genes showed 74–83% similarity in their qRT-PCR expression profile with the RNA-seq data. Eight MYB genes- *CcMYB100* (BC332_00785), *CcMYB16 (*BC332_11900), *CcETC3* (BC332_24253), *CcMYBR12* (BC332_30379), *CcMYB106* (BC332_27082), *CcMYB3R-1* (BC332_21354), *CcMYB3* (BC332_04434), and *CcMYB31* (BC332_19185) showed a significantly higher expression in one or more of the fruit stages of highly pungent *C. chinense* compared with lowly pungent *C. annuum*. The expression of *CcMYBR12* increased from EG to the Br fruit stage in both the *Capsicum spp.*, but the level of expression remains higher in *C. chinense* throughout. *CcMYBR12* expression shows a high similarity between RNA seq and qRT-PCR data, except that it was highest in the Br stage in qRT-PCR and the MG stage in RNA-seq data. *CcMYB16* and *CcETC3* showed two to four times higher expression in *C. chinense*. The transcriptome data for *CcMYB16* suggest the same for the MG and Br stages; however, it was the opposite for the EG stage. *CcETC3* showed similar expression patterns in the transcriptome data. *CcMYB106* and *CcMYB100* showed expression only in the EG stage with a slightly higher level of expression in *C. chinense* as compared with that of *C. annuum*. *CcMYB100* showed three times higher expression in the EG fruit of *C. chinense* as compared with that of *C. annuum*, and *CcMYB31* showed approximately two times higher expression in the MG fruit stage in *C. chinense* compared with that of *C. annuum*. *CcMYB3R-1* was 2-3 times highly expressed in all the three fruit stages of *C. chinense*, while *CcMYB3* showed a lower expression in EG and MG, and a higher expression in the Br stage of *C. chinense* compared with those of *C. annuum*.

### Protein Structure Prediction of *C. chinense* R2R3 *MYB* Genes

The 126 CcMYBs having the R2R3 MYB domain were analyzed for their secondary and tertiary structures using the Phyre2 server (Supplementary Figure 4). The best models for CcMYB proteins showed 20–68% identity and 98.6–100% query coverage with their template sequences. The majority of CcMYB (116) genes were observed to have high coverage and similarity with the c6kksA protein model/template of Arabidopsis R2R3 type MYB2 TF (*WEREWOLF, WER*). For instance, both CcMYB106 and CcMYB113 showed 68% similarity, while CcMYB67-2 was just 21% similar to this template sequence. The remaining seven CcMYBs were modeled with different protein templates, out of which two, namely, CcMYB3R-4 and CcMYB5R, were modeled with a template c1h88C of ternary protein-DNA complex1 of MYB TF, while CcMYB70, CcMYB5-1, and CcMYB100 were modeled with a template named d1mbja of c-MYB DNA-binding domain repeat 3. Two MYBs, i.e., CcMYB121 and CcMYB5-2, were modeled with templates d1gv2a2 and d1h8ac1, respectively, and were related to the Myb/SANT DNA-binding domain family and three MYBs, CcMYB116, CcMYB119, and CcMYB120, with c2yqkA, d2crga1, and c5ylzJ, respectively (Supplementary Table 6). The CcMYBs contained 21–61% α-helix in their secondary structure, while 1–6% β-strands were predicted in only 20 CcMYBs (Supplementary Table 6). Overall, the modeled 3D structures suggested the helix-turn-helix structure similarity of CcMYB proteins with already known Arabidopsis models and were highly reliable.

### Synteny and Gene Duplication Analysis

**Figure 8:**
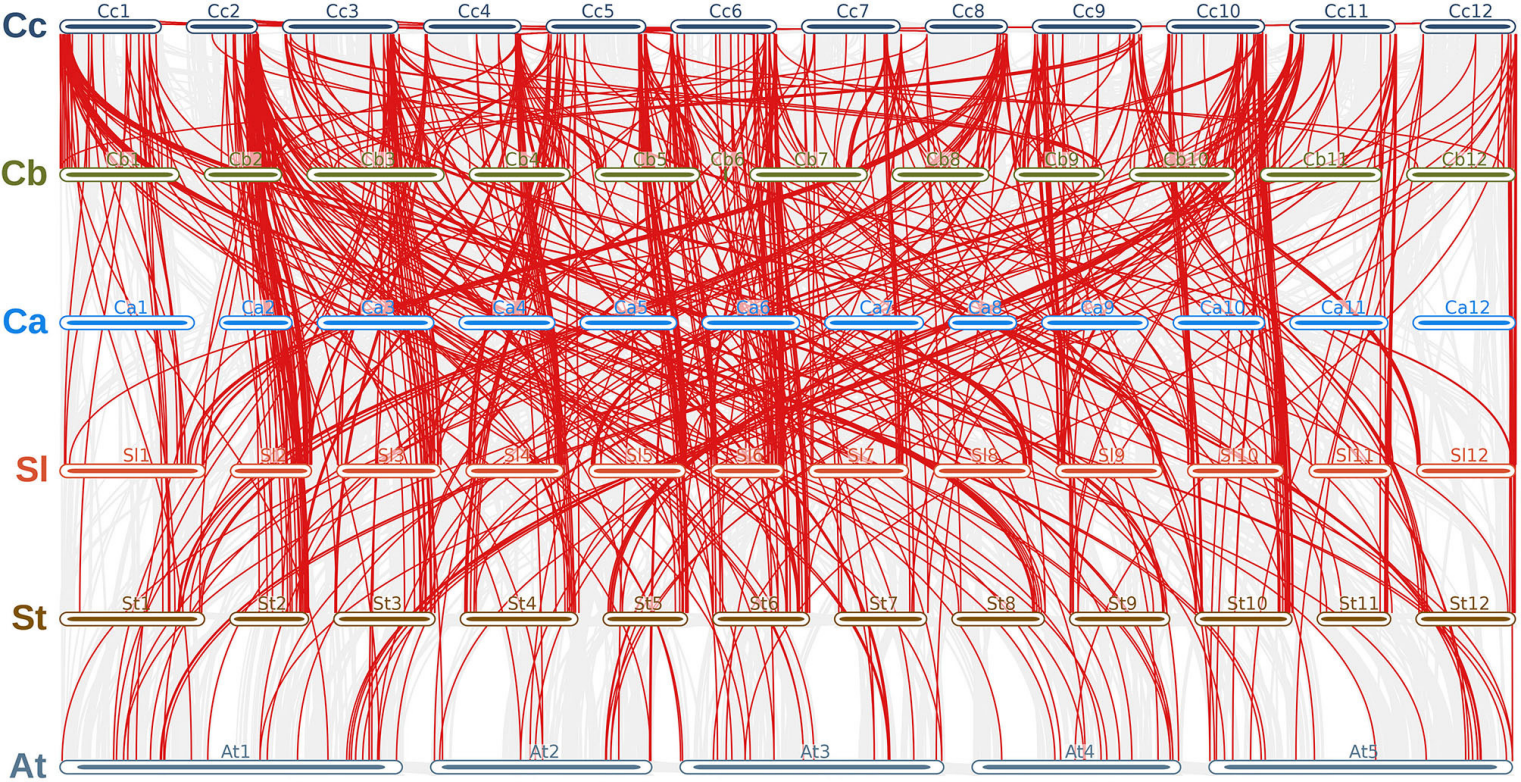
Synteny and collinearity of the *C. chinense* (*CcMYB*) genes with the *Capsicum annuum (Ca), C. baccatum (Cb), Solanum lycoperisucum (Sl), Solanum tuberosum (St), Solanum melongena (Sm)*, and *Arabidopsis thaliana (At)* genomes. Gray lines in the background show collinear segments, while red lines represent syntenic *MYB* gene pairs with respect to the *C. chinense* genome.

**Figure 9:**
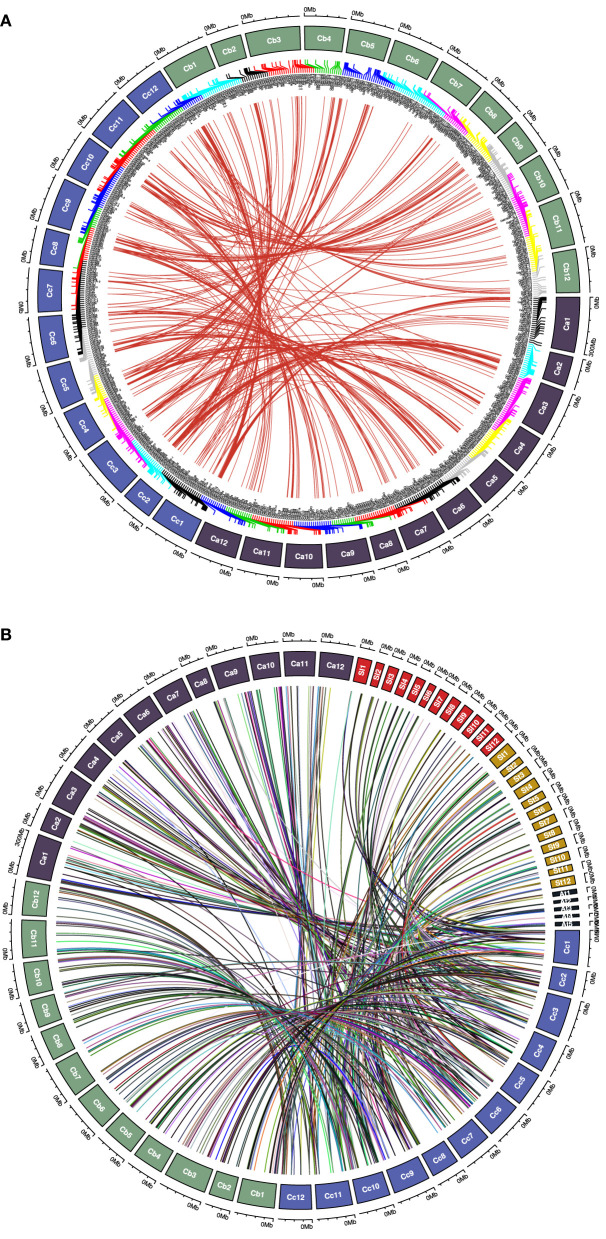
Chord diagram representing **(A)** syntenic *CcMYB* gene pairs with *C. annuum* and *C. baccatum MYBs* and **(B)** *CcMYB* homologs in *C. annuum*, tomato (Sl), potato (St), and Arabidopsis (At) across their respective chromosomes.

We analyzed the synteny and collinearity of five Solanaceae and Arabidopsis genomes with *C. chinense* (Figure 8) and identified a total of 717 conserved syntenic segments (CSSs) in all the species analyzed, ranging from 0.02 to 33.56 Mbp (million base pair) in size, which have at least one *CcMYB* gene in them along with other protein coding genes (Supplementary Table 7, Table 2). The highest number, i.e., 171 and 176 CSSs, was shared with *C. annuum* and *C. baccatum*, respectively. In these CSSs, 203 and 168 unique *CcMYBs* were homologous with *MYBs* of *C. annuum* and *C. baccatum*. Among the *Capsicum* species, most of the CSSs were present on the same chromosomes and had an order of genes similar to that of *C. chinense*, but few were found to be diverged (Table 2). For instance, 47 CSSs were present on different chromosomes, and 75 had a reversed gene order in *C. annuum* with respect to *C. chinense*. In particular, a CSS, harboring *MYB44* along with other genes on chr 1, showed a reverse order of genes in *C. annuum*. The size of this CSS is 1.607 Mbp in *C. chinense* and 1.662 Mbp in *C. annuum*. Another CSS of 1.9 Mbp in *C. chinense* and of 3.17 Mbp in *C. annuum* were present on different chromosomes, chr 7 in *C. chinense* and on chr 9 in *C. annuum* and had a reverse order of genes. In *C. baccatum*, a CSS with *CcMYB105* and *CbMYB104* and other genes was 2.29 Mbp in size on chr 1, while it was 2.47 Mbp in *C. chinense* on chr 8 (Figure 9A, Tables 2, 3).

**Table 2:** Number of conserved syntenic segments (CSSs), with at least one *MYB* gene in them, their size in million base pairs (Mbp), and their diversification in different Solanaceae genomes and *Arabidopsis thaliana*.

**Species**	***C. annuum***	***C. baccatum***	***S. lycopersicum***	***S. tuberosum***	***A. thaliana***	***C. chinense***
No. of CSSs	171	176	108	154	93	15
Size (Mbp)	0.06–28.79	0.07–33.56	0.05–2.75	0.06–3.07	0.02–0.89	0.29–10.95
CSSs on diff chr	47	71	55	74	86	12
CSSs with reverse gene order	75	76	42	65	37	5
*MYB* genes in CSSs	203	168	90	131	77	27

**Table 3:** Chromosome-wise distribution and the number of collinear *CcMYB* genes on the same chromosomes as *C. chinense* in Solanaceae members.

**chr No**.	***C. chinense***	***C. annuum***		***C. baccatum***		***S. lycopersicum***		***S. tuberosum***	
	***MYB* count**	***MYB* Count**	**Collinear**	***MYB* count**	***Collinear***	***MYB count***	**Collinear**	***MYB* count**	**Collinear**
chr01	16	16	16	18	18	8	4	8	2
chr02	18	15	15	12	12	10	10	14	14
chr03	18	15	15	15	6	8	6	14	9
chr04	10	12	9	7	6	9	5	13	7
chr05	20	17	17	17	10	11	5	16	7
chr06	28	20	19	2	2	11	10	17	16
chr07	12	12	8	8	7	5	3	7	6
chr08	9	8	6	8	7	5	2	7	2
chr09	18	11	11	14	12	6	4	7	4
chr10	21	14	14	12	12	8	7	14	14
chr11	15	6	6	11	11	5	3	6	2
chr12	10	9	6	9	8	3	1	8	3
Scaffolds	9	9	–	32	3	0	–	0	–
**Total**	**204**	**164**	**142**	**165**	**114**	**89**	**60**	**131**	**86**

As expected, the genomes of potato (154), tomato (108), and Arabidopsis (93) shared a lesser number of CSSs with the C. *chinense* genome. A number of CSSs were spread on different chromosomes compared with *C. chinense*, i.e., 55 in tomato, 74 in potato, and 86 in Arabidopsis, which was expected as they are more diverged compared with different species of *Capsicum*.

To study the effects of duplication events on the expansion of the *MYB* gene family, *Capsicum* MYB homolog proteins were identified in *C. annuum, C. baccatum*, tomato, potato, and Arabidopsis (Figure 9B). We found 758 pairs of MYB duplicates across the genomes of five species (Supplementary Table 8). Of these, our analysis showed 435 MYB duplicate pairs to be under purifying selection, 23 pairs under neutral selection, and 47 pairs under positive selection. Among these, 225, 213, 125, 79, and 23 CcMYB proteins were orthologous to *C. annuum, C. baccatum*, potato, tomato, and Arabidopsis MYBs, respectively. The value of Ka/Ks ranged from 0.001 to 50 in the homolog pairs. Homologous pairs CcMYB25 and CaMYB25, and CcMYB90 and CaMYB90 were under positive selection. Among others, CcMYB31 and CaMYB31, and CcMYB3 and SlMYB73 were under purifying selection. The average divergence time for CcMYB orthologs with tomato was 37.1 MYA, while that with potato was 33.56 MYA (Supplementary Table 8).

### Identification of Simple Sequence Repeat (SSR) Motifs

We identified SSRs in the *C. chinense MYB* genes and their 1.5-kb upstream promoter, which can be used as molecular markers in future *Capsicum* breeding programs. A total of 169 SSRs were identified in the *C. chinense MYBs*. Out of these, 114 were gene-based from 77 *C. chinense MYB* genes. The remaining 55 SSRs were in the 1.5-Kb upstream regions from the TSS of 49 *C. chinense MYB* genes. Among all the SSRs, the dinucleotide repeats were the most common, i.e., 70.4% in *C. chinense*, followed by tri-, tetra-, penta-, and hexanucleotide repeats. The most common dinucleotide repeat was “AT,” with a frequency of 42. The maximum size of a SSR motif in *C. chinense* was seven repeats of hexanucleotide [ATTTTA] in *CcMYBR11*. The primer sequences and the expected amplicon length of all the SSR repeat motifs in the *Capsicum* species are given in Supplementary Table 9.

## Discussion

*MYB* genes are important TFs involved in the regulation of several biological, developmental, and metabolic processes in plant species such as *Capsicum* (Liu et al., 2015; Xu et al., 2015b; Sun et al., 2019; Cao et al., 2020). Although previously reported in *C. annuum*; in *C. chinense*, however, being one of the most important species, including the naturally occurring highest pungency containing *Capsicum* genotype, Bhut jolokia/ghost chili, no report of *MYB* gene identification was reported. Additionally, in this study, we have identified *MYB* genes in *C. baccatum* (240). Previously, Arce-Rodríguez et al. (2021) reported 235 *MYBs* in *C. annuum*, but this study identified additional 10 new *MYB* genes (6 atypical R2R3 *MYBs* and 4 *MYBs* with other domains). The greater number of *MYB* genes in *Capsicum* might be due to genome expansion. Alternatively, it may also mean the deletion or loss of genes from other lineages. Despite a large number of *MYB* genes in *Capsicum*, there are few chances that their functions are redundant but more likely to overlap in their functionalities (Jin and Martin, 1999) or mask the functions of each other. The large size of the *MYB* gene family in plants can be attributed to the high rates of duplication and retention of duplicate copies compared with other TF families (Shiu et al., 2005). The retention of such large numbers of *MYB* genes, during evolution, in the members of Solanaceae indicates their positive selection and acquisition of new functions. R2R3-MYBs were the most common type of *MYBs* in other *Capsicum* like other plant species (Katiyar et al., 2012; Du et al., 2015; Li et al., 2019). The greater number of R2R3-MYBs in plants suggests their selective amplification and expansion after the loss of R1 repeat in ancestral three repeat MYBs (Lipsick, 1996) and can be involved in the plant speciation process. Whether an increase in genome size or the number of genes explains the huge number of *MYB* genes in phylogenetically related genomes or not remains to be explored further with broad sampling strategies.

### *CcMYBs* Within Capsaicinoid QTLs Were Differentially Expressed

The chromosomal distribution of *MYB* genes was found to be random in the three *Capsicum* species. Seven *Capsicum* chromosomes had around 60% of the *MYB* genes (Figure 1 and Supplementary Figure 1). Five *CcMYBs, CcMYB101, CcMYB46, CcMYB6, CcPHR8*, and *CcRVE5*, on chr 6 of *C. chinense* and two *CaMYBs*, namely, *CaMYB3* and *CaHHO1*, on chr 2 of *C. annuum* were found inside the previously reported capsaicinoid QTLs (Han et al., 2018; Park et al., 2019). Among these, two MYB genes, *MYB3* and *MYB46*, also showed a significant differential expression in the fruit tissues of lowly pungent *C. annuum* and highly pungent *C. chinense* in the qRT-PCR analysis (Figure 7B). *MYB46* was also found to be co-expressed with *CHS* in the co-expression analysis, suggesting its possible involvement in the transcriptional regulation of anthocyanin biosynthesis. Other two *MYB* genes in the QTLs, *CcPHR8* and *CaHHO1*, were co-expressed with phenylpropanoid gene *C4H* and anthocyanin biosynthesis gene *DFR* (Figure 5), suggesting their potential roles in the respective pathways. The motif identification analysis revealed motifs that are important to impart functional significance to *MYB* proteins. For example, recently, motif-23 was reported to mediate the interaction between *Cucumis sativus* MYB6 and a MYB-related protein CsTRY (Yang et al., 2018). Although functions for most of these motifs are still unknown, they may be involved in protein-protein interactions or other biological roles and are subject to further exploration (Millard et al., 2019). *MYB* genes also respond to environmental and hormonal changes to regulate gene expression during abiotic stress responses in plants (Urao et al., 1996; Abe et al., 1997; Li et al., 2015). Here, the *cis*-element analysis also suggests environmental and hormonal regulatory mechanisms for the *Capsicum MYB* genes. MYB binding motifs (MBSI) and MYB recognition elements (MREs) observed in the promoter regions of *Capsicum MYB* genes may indicate their regulatory roles in flavonol biosynthesis (Solano et al., 1997; Mehrtens et al., 2005). Previously, *MYB14* has been shown to contain MRE alongside AT-rich element, GATA-motif, ARE, Box 4, and circadian in its promoter region, and to be activated by UV-C light in order to activate stilbene synthesis in *Vitis labrusca* (Bai et al., 2019). Likewise, another *MYB15* that controls basal immunity in *V. quinquangularis* was found to harbor cis-elements like GCN4-motif, MBS, and TCA-element in its promoter region (Luo et al., 2019). The identification of cis-elements may aid in deciphering regulatory networks and may further lead to the isolation and characterization of corresponding TFs.

### Phylogenetic Analysis Showed Functional Importance of *Capsicum MYBs*

The phylogenetic relationships revealed in the MYB protein sequences can be utilized to relate their function. For instance, *CcMYB3, CcMYB12, CcMYB14, CcMYB16*, etc., along with their sister clades in *C. baccatum* and Arabidopsis, form subgroup VIII. *AtMYB1* in the same subgroup is involved in pollen development, and *AtMYB44, AtMYB73*, and *AtMYB77* are involved in lateral root growth and salinity response (Reňák, 2012; Kim et al., 2013; Zhao et al., 2014). Similar functions may be stipulated for *Capsicum MYBs* of the same subgroup. Subgroup XIII *MYBs* such as *AtMYB30* positively regulate fatty acid biosynthesis as well as hypersensitive cell death response, while subgroup XXIV *MYBs*, such as *AtMYB80*, is, again, important for pollen development and tapetal growth (Raffaele et al., 2008; Phan et al., 2011). Subgroup XXII includes *AtMYB49* and *AtMYB74* that provide tolerance to abiotic stresses like salinity, and *AtMYB102* provides resistance to biotic stresses like insect herbivores (De Vos et al., 2006; Xu et al., 2015a; Zhang et al., 2020). Subgroup X contains *AtMYB33* and *AtMYB101*, which are targets of the miR159 family upon ABA accumulation during seed germination, and are shown to be involved in drought stress (Reyes and Chua, 2007). *CcMYBs* such as *MYB31, MYB59, MYB60, MYB61, MYB63, MYB64*, and *MYB65* in subgroup XIV did not cluster with any *AtMYBs* (Figure 4). This was similar to the results of previous analysis on *C. annuum*, where clades 24 and 25 harbored *Capsicum-*specific *MYBs* (Arce-Rodríguez et al., 2021). Also, the same *Capsicum MYBs* clustered with the tomato and potato *MYB* homologs in a distinct subgroup, suggesting these *MYBs* to be Solanaceae-specific (Figure 4, Supplementary Figure 5). However, contrary to the findings of Arce-Rodríguez et al. (2021), other *MYBs*, such as *MYB116* and *MYB119*, which were not reported earlier, clustered separately from At*MYBs* and were present in subgroups I and II, respectively (Figure 4). *CcMYB98, CcMYB99*, and *CcMYB100* lie in subgroup XIX with *AtMYB11, AtMYB12*, and *AtMYB111* that control flavonol glycoside accumulation (Stracke et al., 2010). *CcMYB115* and *CcA* in subgroup XX share a sister clade with *AtMYB75* and *AtMYB90*. *AtMYB75* has been extensively studied for its role in anthocyanin biosynthesis and *AtMYB90* for phenylpropanoid biosynthesis (Borevitz et al., 2000; Teng et al., 2005). *CcMYB46* lies along with *AtMYB61* in subgroup XVI, which functions as a transcriptional control for the development of root, seed, and vascular tissues (Romano et al., 2012). *MYB3Rs* have been shown to regulate the cell cycle like c-MYB in animals by regulating cyclin genes *via* MYB recognition elements (MREs) in cyclin gene promoters *via* the R3 MYB repeat (Ito et al., 2001). One major challenge in the functional characterization of *MYB* genes in *Capsicum* could be the functional redundancy of duplicated *MYBs* (Dubos et al., 2010). The second phylogenetic tree among the *MYBs* in the three *Capsicum* species revealed *C. chinense-* and *C. baccatum-*specific lineages, leading to species-specific phenotypes. Similarly, when we analyzed the evolutionary relationship among *MYBs* between the *Capsicum* species and other Solanaceae family members along with Arabidopsis, *C. chinense-*specific clades having *CcMYBR14* were observed (Supplementary Figures 3, 5). However, the phylogenetic tree has a low coverage sampling and may have omitted the evolutionary processes leading to the emergence of these clades and is less representative of the process of speciation in *Capsicum* species.

### Expression Analysis Revealed Eight *MYB* Genes Were Highly Expressed in *C. chinense*

The expression analysis revealed the spatio-temporal expression pattern of *CcMYB* genes in the EG, MG, and Br fruit development stages of two *Capsicum* genotypes (Figures 6, 7). Of the 24 *MYB* genes showing DE in RNA-seq data and validated by qRT-PCR, eight showed a high expression in one or more of the fruit stages in *C. chinense* as compared with those of *C. annuum*. For instance, *CcMYB106* and *CcMYB100* showed a significantly higher expression in the EG fruit in *C. chinense* compared with that of *C. annuum* in both the transcriptome data and qRT analysis. *CcMYB100* is a homolog of *SlMYB12*, which has been reported to regulate the flavonol biosynthesis pathway in tomatoes (Ballester et al., 2010). Moreover, our co-expression analysis also showed that *CcMYB100* clustered with *DFR*, an anthocyanin pathway gene (Figure 5). *CcMYB16* and *CcETC3* show a maximum expression in the EG stage, which then decreases in the MG stage and then again increases in the Br stage in both the species. *CcMYB16* is also clustered with *DFR* in the co-expression analysis, suggesting its role in anthocyanin biosynthesis. In the phylogenetic analysis, *CcMYB16* had clustered with *AtMYB44, AtMYB70, AtMYB77*, etc., in subgroup VIII, which may suggest its role in plant growth and development, and abiotic and biotic stress responses (Figure 4; Jung et al., 2008; Shim et al., 2013; Zhao et al., 2014). *CcETC3*, on the other hand, was segregated with *AtETC2*, which plays a crucial role in trichome development and patterning (Supplementary Figure 3; Kirik et al., 2004; Hilscher et al., 2009). Here, the overall expression pattern of both the *MYB* genes indicates their possible functions in fruit ripening and development in *Capsicum*. *CcMYB3R-1* showed a similar pattern of expression in both *Capsicum* species; however, the level of expression was higher in *C. chinense*. *AtMYB3R-1*, which lies in the same subgroup V as *CcMYB3R-1*, is known to regulate cell cycle and abiotic stress-responsive genes, suggesting similar functions for *CcMYB3R-1* (Figure 4; Dai et al., 2007; Ma et al., 2009; Haga et al., 2011). *CcMYB3* showed a similar pattern of increased expression from EG to Br in both the *Capsicum* species. *CcMYB3* was found within the capsaicinoid QTL and clustered with *AtMYB73* in subgroup VIII, suggesting its role in capsaicinoid biosynthesis and abiotic stress response (Figure 4, Supplementary Figure 1A; Kim et al., 2013). Another *MYB* gene, *CcMYB46*, however, present in capsaicinoid QTL, did not show a higher expression in *C. chinense* as well as *C. annuum*. In the co-expression analysis, *CcMYB*46 was also clustered with the anthocyanin pathway gene *CHS* (Figure 5). *CcMYB31* showed a significantly higher expression in the MG fruit in *C. chinense* compared with that of *C. annuum* (Figure 7B). The qRT expression trends of *CcMYB31* and its homolog *CaMYB31* during fruit development were similar to those of their respective RNA-seq expression data (Figures 6, 7A). *CcMYB31* and *CcMYB48* were coexpressed with CBGs- *KasI* and *PAL*, respectively, in the co-expression analysis. Previously, the expression levels of their homologs, *CaMYB31* and *CaMYB48*, have been reported to correlate with capsaicinoid levels in *C. annuum* (Arce-Rodríguez and Ochoa-Alejo, 2017; Han et al., 2019; Sun et al., 2020). Furthermore, other *CcMYBs*, such as *CcDIV1, CcMYB4, CcMYB52, CcMYB86, CcMYB108, CcMYBR6*, and *CcARR11* also co-expressed with *Kas, FatA*, and *BCKDH* from the capsaicinoid biosynthesis pathway, suggesting their potential role in capsaicinoid biosynthesis regulation. However, the expression of *CcMYB52* and *CcMYB86* did not show a significant difference between *C. chinense* and *C. annuum*. Although *pAMT, KasIIIb, ACL, BCAT, PAL*, and *FatA* were grouped into the same major cluster in the co-expression analysis, other capsaicinoid pathway genes, such as *AT3*, clustered along with *COMT* and *BCKDH* with *KasIIIa* in their respective clusters (Figure 5). However, previously in *C. annuum, AT3, pAMT, Kas*, and *BCKDH* have been reported to be present in the same co-expression cluster (Arce-Rodríguez et al., 2021), which may be due to the selection of different species/genotypes and distinct fruit developmental stages. Sarpras et al. (2016) have previously reported that capsaicinoid pathway genes are highly expressed in *C. chinense* as compared with *C. annuum* and showed correlations with pungency levels of *C. chinense*, EG (315936 SHU), MG (763411.2 SHU), and Br (925084.8 SHU), and of *C. annuum*, EG (3478.4 SHU), MG (6656 SHU), and Br (7034.4 SHU) (Sarpras et al., 2016, 2019). In our study, several *CcMYB* genes also showed a significantly high expression in highly pungent *C. chinense* compared with lowly pungent *C. annuum*, while some of them co-expressed with capsaicinoid/phenylpropanoid biosynthesis pathway genes, which can be selected for further validation in correlation to pungency regulation (Figures 5–7). Additionally, we have also analyzed the expression of eight *MYB* genes in four other *Capsicum* accessions (two each from *C. chinense* and *C. annuum*) to further understand their expression pattern in high and low pungent *Capsicum* during fruit development (Supplementary Figure 6). Overall, the qRT-PCR expression and co-expression analysis suggested that *CcMYBs* potentially have a diverse role in the regulation of capsaicinoid, phenylpropanoid, and anthocyanin biosynthesis (Supplementary Figure 6). Furthermore, *CcMYB10, CcMYB82, CcMYB1R-1, CcRVE4*, and *CcMYB102* were co-expressed with fruit shape and size genes like *Auxin receptor, WD-40, SUN*, and *EAR1*, suggesting their potential roles in fruit development (Figure 5).

### Homology Modeling Suggested a High Structural Similarity of R2R3 CcMYBs With Arabidopsis R2R3 MYB Domain

With only sparse 3D structures characterized compared with increasingly known protein sequences, there is a massive need for the prediction of protein structures in order to bridge this ever-widening gap. In the absence of experimentally determined protein structures, computational tools for protein structure predictions provide a reliable prerequisite. For example, the 3D structure for MYB108-like involved in responses to drought and salt stresses in cotton was predicted using a Swiss model to better understand its mechanism of action (Ullah et al., 2020). We predicted the 3D models for the identified CcMYB protein sequences by sequence based homology modeling (Supplementary Figure 4). Most of the CcMYBs were modeled with a single chain model, c6kksA of MYB DNA-binding domain repeat with a unique helix-loop-helix structure in Arabidopsis R2R3 MYB2 TF WER (Supplementary Figure 4, Supplementary Table 6). The crystal structure of the WER complex with its target DNA was determined recently by X-Ray diffraction and showed that third recognition helices of both R2 and R3 MYB repeats bind to the major groove of DNA in a sequence-specific manner (Wang et al., 2020a). WER is a MYB-related protein that transcriptionally regulates the expression of *GLABRA2* to control epidermal cell patterning in a position-dependent manner in Arabidopsis roots (Lee and Schiefelbein, 1999). The CcMYB R2R3 domains shared a moderate degree of sequence similarity (<68%) to the identified template sequence of the MYB domain, which may indicate similarity in the mechanism of binding to its target DNA. Provided there is still no method to determine protein structures solely based on sequence information without known reference structures, the computational based analysis of CcMYB protein sequences can be a stepping stone toward structural determination.

### MYB Containing Conserved Syntenic Segments Showed Diversification Among Solanaceae Members

With an increasing number of sequenced plant genomes, little has been understood about the genomic divergence, chromosome evolution, and evolutionary relationships among them. Synteny and collinearity are one way to detect complex evolutionary relationships among plant genomes, especially in reference to multigene families. Synteny analysis among Solanaceae genomes displays the diversification and conservation of chromosomal segments containing *MYB* genes (Table 2). The *Capsicum* genomes shared more numbers of CSSs, most of them on similar chromosomes and with similar order of genes, as compared with Solanum genomes, which is expected. However, several CSSs were diversified in *Capsicum* spp. despite belonging to the same genus and being closely related. The least conservation in CSSs was observed with the Arabidopsis genome with only seven CSSs present on similar chromosomes as *C. chinense*. Only 142 (56.57%) and 114 (45.41%) *C. chinense MYB* genes were collinear with the *C. annuum* and *C. baccatum* genomes, respectively (Table 3). The lower level of collinearity suggests that *Capsicum* genomes have undergone large-scale chromosomal rearrangements during their evolution. Therefore, it may indicate that *Capsicum* genomes, and *MYB* genes, have diverged, and that there is a need to study specific genes and genomes. In the genomes of species belonging to the *Solanum* genus, even a lower level of conservation and collinearity of *MYB* genes was observed with respect to *C. chinense* as expected (Table 2).

Apart from the genome-wide duplications, tandem and segmental duplications within *C. chinense* highlight the duplication frequency of *MYB* genes within the *Capsicum* genome (Table 1). Genome-wide and tandem duplications have been implicated in the expansion of the *R2R3-MYB* gene family and are an important measure for the same (Du et al., 2015). In our study, we reported the average duplication time of *Capsicum MYB* homolog pairs as 28.56 MYA. The *Ka/Ks* ratios of 435 *MYB* duplicate pairs were <1, which indicates purifying selection. Forty-seven *MYB* pairs that had *Ka/Ks* >1 showed positive selection. We found 241 *Capsicum MYBs* with orthologs in tomato, potato, and Arabidopsis that were operating under purifying selection (Supplementary Table 8). The strong purifying selection of the *Capsicum MYB* gene family is similar to the tandem expansion and positive selection observed in the GRAS TF family and *R*-genes in Arabidopsis (Chen et al., 2010; Wu et al., 2014).

### *MYB*-Specific SSR Repeats Can Serve as Potential Molecular Markers

In an earlier study, gene-specific SSRs related to *Capsicum* fruit ripening showed high polymorphism among *Capsicum spp* (Dubey et al., 2019). In this study, the SSRs predicted in the genic and non-genic regions of *Capsicum MYBs* have rendered them useful in *Capsicum* breeding and improvement programs. Compared with the 1.5-kb upstream regions, more SSRs were present in the genic regions, 67.45% (Supplementary Table 9). However, previous studies have suggested higher conservation and less variation in the genic regions of different species (Kim et al., 2008; Zhang et al., 2014; Chhapekar et al., 2020). This may be due to the less coverage of the upstream region of *MYB* genes for SSR prediction, which is 1.5 Kb from the TSS in our study. The di- and tri-nucleotide repeats have been found to be varying in number from species to species but are the most common SSRs in plants (Saha et al., 2017). We also found di-nucleotide repeats to be abundant among all the SSRs in *Capsicum* species. Both gene-based and non-genic SSRs in *Capsicum MYBs* can be used as potential markers for the selection of associated genes in fruit breeding programs.

## Conclusion

A total of 251, 240, and 245 *MYB* genes were identified in the *C. chinense, C. baccatum*, and *C. annuum* genomes. Twenty tandem and 41 segmental duplication events may have led to the expansion of the *MYB* gene family in the *C. chinense* genome. Also, 225, 213, 125, 79, and 23 CcMYB proteins were orthologous to *C. annuum, C. baccatum*, potato, tomato, and Arabidopsis MYBs, respectively. The transcriptome analysis revealed that 54 *CcMYB* and 81 *CaMYB* genes were differentially expressed during fruit development in *C. chinense* and *C. annuum*, respectively. Eight *CcMYB* genes were highly expressed in highly pungent *C. chinense* compared with lowly pungent *C. annuum* in the qRT-PCR analysis. Additionally, our finding also suggests the *CcMYBs*, such as *CcMYB16, CcMYB28, CcMYB100, CcDIV4, CcMYB46*, and *CcMYB74*, as potential anthocyanin biosynthesis regulators in *Capsicum*. While along with already characterized *MYB31* and *MYB108* (Arce-Rodríguez and Ochoa-Alejo, 2017) other *MYBs* such as *CcMYB4, CcDIV1, CcMYBR6*, and *CcARR11* may be used as potential targets for the regulation of capsaicinoid biosynthesis. On the other hand *CcMYBs*, such as *CcMYB10, CcMYB82, CcMYB1R-1, CcDIV1, CcRVE4*, and *CcMYB102*, may be investigated for their role in fruit development/shape-size regulation in fruits of *Capsicum* species. The *MYB* genes identified could be studied for their functional roles, so that they can be manipulated for *Capsicum* trait improvement.

## Data Availability Statement

The original contributions presented in the study are publicly available. This data can be found here: The RNA sequencing data related to this study were submitted on NCBI under BioProject (PRJNA679780). Sequence Read Archive (SRA) accessions for *C. chinense* samples includes SRR12963502, SRR12963513, and SRR12963514 for early green (EG), SRR12963488, SRR12963489, and SRR12963490 for mature green (MG), and SRR12963491, SRR12963492, and SRR12963493 for breaker (Br) fruit samples. SRA accessions for *C. annuum* samples are SRR12963501, SRR12963503 and SRR12963504 for EG, SRR12963495, SRR12963496, and SRR12963497 for MG and SRR12963498, SRR12963499, and SRR12963500 for Breaker fruit samples.

## Author Contributions

NR conceived and designed the research. KI, AR, IA, MD, and JM conducted field and lab experiments. KI and AR performed *in silico* analysis and analyzed the data. KI and AR drafted and NR corrected and finalized the manuscript. All authors read and approved the final manuscript.

## Funding

This study was supported by the Department of Biotechnology (DBT), Ministry of Science and Technology, Government of India in the form of Ramalingaswami Re-entry Fellowship cum Research Grant (BT/RLF/Re-entry/46/2011), and Science and Engineering Research Board (SERB) (SB/EMEQ-086/2014), Department of Science and Technology (DST), Government of India.

## Conflict of Interest

The authors declare that the research was conducted in the absence of any commercial or financial relationships that could be construed as a potential conflict of interest.

## Publisher's Note

All claims expressed in this article are solely those of the authors and do not necessarily represent those of their affiliated organizations, or those of the publisher, the editors and the reviewers. Any product that may be evaluated in this article, or claim that may be made by its manufacturer, is not guaranteed or endorsed by the publisher.

## Supplementary Material

Supplementary Figure 1 Chromosomal distribution of *MYB* genes across 12 *Capsicum* chromosomes of **(A)** *C. annuum* and **(B)** *C. baccatum*. The capsaicinoid QTLs in *C. annuum* adapted from Han et al., 2018 are shown in shaded blocks.

Supplementary Figure 2 Gene structure and phylogenetic tree of *MYB* genes in **(A)** *C. annuum* and **(B)** *C. baccatum*. The length of *MYB* genes are in a kb (kilobase) scale, and the exons are represented using purple and brown blocks, while the connecting lines represent the intronic regions.

Supplementary Figure 3 Phylogenetic tree of MYB protein sequences from the three *Capsicum* species (*C. chinense, C. annuum*, and *C. baccatum*). The tree was constructed using the Maximum Likelihood method with 1,000 bootstrap replications.

Supplementary Figure 4 Predicted 3D structural models of 126 *C. chinense* R2R3 MYB proteins. Models were shown in rainbow color from N to the C terminus.

Supplementary Figure 5 The phylogenetic tree is drawn from MYB protein sequences of *C. chinense, C. annuum, C. baccatum*, tomato, potato, and Arabidopsis using the Maximum likelihood method with 1,000 bootstrap values.

Supplementary Figure 6 Relative expression of eight *CcMYB* genes in six genotypes, three *C. annuum* (Kosom Moso, JH15 and JH7) and three *C. chinense* (Lota, CCGHY6, and BJS) by qRT-PCR in the three fruit developmental stages. A Student *t*-test was performed to calculate the significant difference of expression. The significance level was represented as ^***^*p* < 0.001; ^**^*p* < 0.01, and ^*^*p* < 0.05.

Supplementary Table 1 Primer sequences used for the qRT-PCR expression analysis of *CcMYBs* and capsaicinoids biosynthesis pathway genes.

Supplementary Table 2 The characteristic features of *Capsicum MYBs*. **(A)** *C. chinense (CcMYBs)* and **(B)** *C. annuum* (*CaMYBs*) genes with RNAseq expression data in the three fruit developmental stages. **(C)** *C. baccatum* (*CbMYBs*) along with their genomic coordinates.

Supplementary Table 3 Conserved amino acid motifs identified in CcMYB protein sequences using the MEME suite.

Supplementary Table 4 Cis-regulatory elements identified in the 1.5-kb upstream promoter region of *CcMYBs* using the PLANTCare database. **(A)** Cis-acting elements along with their sequences, function, and presence in the *MYB* genes and **(B)** their frequency in the upstream region of each *MYB* gene.

Supplementary Table 5 RNA sequencing alignment summary. Raw reads and aligned read summary with Hisat2 in the **(A)** *C. chinense* and **(B)** *C. annuum* fruit samples.

Supplementary Table 6 The 126 R2R3 CcMYBs and the probability and identity of their corresponding homologous protein model/template.

Supplementary Table 7 *MYB* containing conserved syntenic segments (CSSs) from the *Capsicum chinense* genome compared with the *C. annuum, C. baccatum*, tomato, potato, and Arabidopsis genomes [Note: Cc, Ca, Cb, St, Sl, and At represent the chromosomes from the *C. chinense, C. annuum, C. baccatum*, potato, tomato, and Arabidopsis genomes].

Supplementary Table 8 The *C. chinense* (*CcMYBs*) and their homologous gene pairs from *C. annuum, C. baccatum*, tomato, potato, and Arabidopsis along with the *Ka/Ks* ratios and duplication time (in MYA) for each homologous pair.

Supplementary Table 9 SSRs identified in the *MYB* genes and 1.5-kb promoter regions in *C. chinense* with the primer sequences and the amplicon size of the SSR motifs.

Supplementary File 1 CDS and protein sequences of *C. chinense MYB* family members.

## Abbreviations

CcMYB: *Capsicum chinense* MYB
CbMYB: *Capsicum baccatum* MYB
TF: transcription factor
DBD: DNA-binding domain
CSSs: conserved syntenic segments
qRT-PCR: quantitative real-time PCR
SSRs: simple sequence repeats
HMM: Hidden Markov Model
BLASTs: basic local alignment search tool sequence
NCBI: National Centre for Biotechnology information
CDD: Conserved Domains Database
pI: isoelectric point
GRAVY: grand average of hydropathicity
SMART: simple modular architecture research tool
CDS: coding sequences
QTLs: quantitative trait loci
GSDS: gene structure display server
MEME: multiple em for motif elicitation
NJ: neighbor-joining
ML: maximum likelihood
DPA: day post anthesis
EG: early green
MG: mature green
Br: breaker
RIN: RNA integrity number
RNAseq: RNA sequencing
FC: fold change
DEG: differentially expressed gene
Kb: kilobase
TSS: transcription start site
Chr: chromosome
FPKM: fragments per kilobase of transcript per million mapped reads
DE: differentially expressed
Mbp: million base pair.
