# Supplementary material for: Capsicum chinense MYB Transcription Factor Genes: Identification, Expression Analysis, and Their Conservation and Diversification With Other Solanaceae Genomes
Source: Front Plant Sci. 2021 Oct 13;12:721265. doi: 10.3389/fpls.2021.721265 (PMC8548648; doi:10.3389/fpls.2021.721265)
Supplement: Supplementary Figure 1 — Chromosomal distribution of MYB genes across 12 Capsicum chromosomes of (A) C. annuum and (B) C. baccatum. The capsaicinoid QTLs in C. annuum adapted from Han et al., 2018 are shown in shaded blocks. [file Data_Sheet_1.zip › Supplementary Figure S1.PDF]

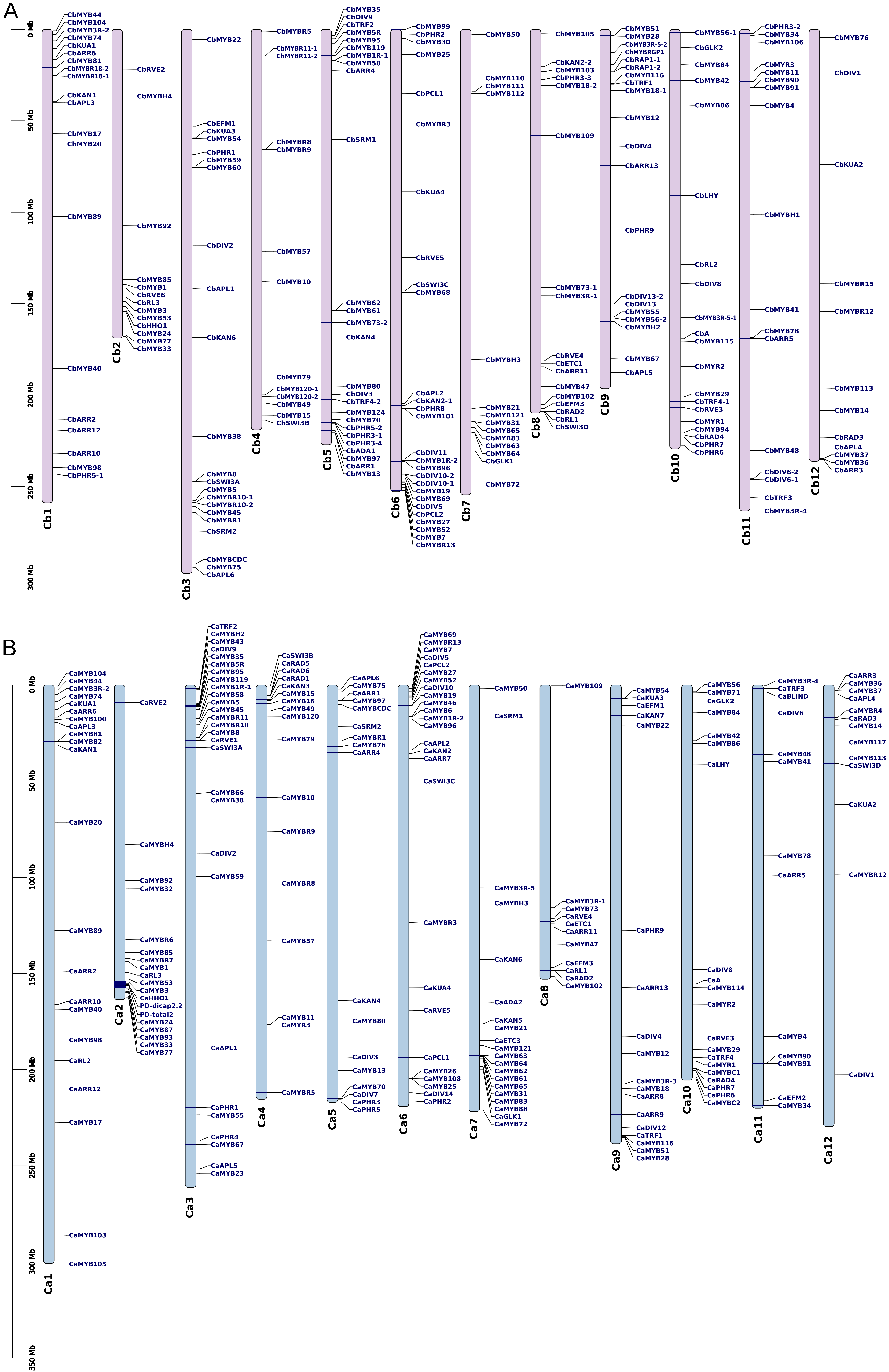
A
0 Mb
50 Mb
100 Mb
150 Mb
200 Mb
250 Mb
300 Mb
CbMYB44
CbMYB104
CbMYB3R-2
CbMYB74
CbKUA1
CbARR6
CbMYB81
CbMYBR18-2
CbMYBR18-1
CbKAN1
CbAPL3
CbMYB17
CbMYB20
CbMYB89
CbMYB40
CbARR2
CbARR12
CbARR10
CbMYB98
CbPHR5-1
Cb1
CbRVE2
CbMYBH4
CbMYB92
CbMYB85
CbMYB1
CbRVE6
CbRL3
CbMYB3
CbMYB53
CbHHO1
CbMYB24
CbMYB77
CbMYB33
Cb2
CbMYB22
CbEFM1
CbKUA3
CbMYB54
CbPHR1
CbMYB59
CbMYB60
CbDIV2
CbAPL1
CbKAN6
CbMYB38
CbMYB8
CbSWI3A
CbMYB5
CbMYBR10-1
CbMYBR10-2
CbMYB45
CbMYBR1
CbSRM2
CbMYBCDC
CbMYB75
CbAPL6
Cb3
CbMYBR5
CbMYBR11-1
CbMYBR11-2
CbMYBR8
CbMYBR9
CbMYB57
CbMYB10
CbMYB79
CbMYB120-1
CbMYB120-2
CbMYB49
CbMYB15
CbSWI3B
Cb4
CbMYB35
CbDIV9
CbTRF2
CbMYB5R
CbMYB95
CbMYB119
CbMYB1R-1
CbMYB58
CbARR4
CbSRM1
CbMYB62
CbMYB61
CbMYB73-2
CbKAN4
CbMYB80
CbDIV3
CbTRF4-2
CbMYB124
CbMYB70
CbPHR5-2
CbPHR3-1
CbPHR3-4
CbADA1
CbMYB97
CbARR1
CbMYB13
Cb5
CbMYB99
CbPHR2
CbMYB30
CbMYB25
CbPCL1
CbMYBR3
CbKUA4
CbRVE5
CbSWI3C
CbMYB68
CbAPL2
CbKAN2-1
CbPHR8
CbMYB101
CbDIV11
CbMYB1R-2
CbMYB96
CbDIV10-2
CbDIV10-1
CbMYB19
CbMYB69
CbDIV5
CbPCL2
CbMYB27
CbMYB52
CbMYB7
CbMYBR13
Cb6
CbMYB50
CbMYB110
CbMYB111
CbMYB112
CbMYBH3
CbMYB21
CbMYB121
CbMYB31
CbMYB65
CbMYB83
CbMYB63
CbMYB64
CbGLK1
CbMYB72
Cb7
CbMYB105
CbKAN2-2
CbMYB103
CbPHR3-3
CbMYB18-2
CbMYB109
CbMYB73-1
CbMYB3R-1
CbRVE4
CbETC1
CbARR11
CbMYB47
CbMYB102
CbEFM3
CbRAD2
CbRL1
CbSWI3D
Cb8
CbMYB51
CbMYB28
CbMYB3R-5-2
CbMYBRGP1
CbRAP1-1
CbRAP1-2
CbMYB116
CbTRF1
CbMYB18-1
CbMYB12
CbDIV4
CbARR13
CbPHR9
CbDIV13-2
CbDIV13
CbMYB55
CbMYB56-2
CbMYBH2
CbMYB67
CbAPL5
Cb9
CbMYB56-1
CbGLK2
CbMYB84
CbMYB42
CbMYB86
CbLHY
CbRL2
CbDIV8
CbMYB3R-5-1
CbA
CbMYB115
CbMYR2
CbMYB29
CbTRF4-1
CbRVE3
CbMYR1
CbMYB94
CbRAD4
CbPHR7
CbPHR6
Cb10
CbPHR3-2
CbMYB34
CbMYB106
CbMYR3
CbMYB11
CbMYB90
CbMYB91
CbMYB4
CbMYBH1
CbMYB41
CbMYB78
CbARR5
CbMYB48
CbDIV6-2
CbDIV6-1
CbTRF3
CbMYB3R-4
Cb11
CbMYB76
CbDIV1
CbKUA2
CbMYBR15
CbMYBR12
CbMYB113
CbMYB14
CbRAD3
CbAPL4
CbMYB37
CbMYB36
CbARR3
Cb12
B
0 Mb
50 Mb
100 Mb
150 Mb
200 Mb
250 Mb
300 Mb
350 Mb
CaMYB104
CaMYB44
CaMYB3R-2
CaMYB74
CaKUA1
CaARR6
CaMYB100
CaAPL3
CaMYB81
CaMYB82
CaKAN1
CaMYB20
CaMYB89
CaARR2
CaARR10
CaMYB40
CaMYB98
CaRL2
CaARR12
CaMYB17
CaMYB103
CaMYB105
Ca1
CaRVE2
CaMYBH4
CaMYB92
CaMYB32
CaMYBR6
CaMYB85
CaMYBR7
CaMYB1
CaRL3
CaMYB53
CaMYB3
CaHHO1
PD-dicap2.2
PD-total2
CaMYB24
CaMYB87
CaMYB93
CaMYB33
CaMYB77
Ca2
CaTRF2
CaMYBH2
CaMYB43
CaDIV9
CaMYB35
CaMYB5R
CaMYB95
CaMYB119
CaMYB1R-1
CaMYB58
CaMYB5
CaMYB45
CaMYBR11
CaMYBR10
CaMYB8
CaRVE1
CaSWI3A
CaMYB66
CaMYB38
CaDIV2
CaMYB59
CaAPL1
CaPHR1
CaMYB55
CaPHR4
CaMYB67
CaAPL5
CaMYB23
Ca3
CaSWI3B
CaRAD5
CaRAD6
CaRAD1
CaKAN3
CaMYB15
CaMYB16
CaMYB49
CaMYB120
CaMYB79
CaMYB10
CaMYBR9
CaMYBR8
CaMYB57
CaMYB11
CaMYR3
CaMYBR5
Ca4
CaAPL6
CaMYB75
CaARR1
CaMYB97
CaMYBCDC
CaSRM2
CaMYBR1
CaMYB76
CaARR4
CaKAN4
CaMYB80
CaDIV3
CaMYB13
CaMYB70
CaDIV7
CaPHR3
CaPHR5
Ca5
CaMYB69
CaMYBR13
CaMYB7
CaDIV5
CaPCL2
CaMYB27
CaMYB52
CaDIV10
CaMYB19
CaMYB46
CaMYB6
CaMYB1R-2
CaMYB96
CaAPL2
CaKAN2
CaARR7
CaSWI3C
CaMYBR3
CaKUA4
CaRVE5
CaPCL1
CaMYB26
CaMYB108
CaMYB25
CaDIV14
CaPHR2
Ca6
CaMYB50
CaSRM1
CaMYB3R-5
CaMYBH3
CaKAN6
CaADA2
CaKAN5
CaMYB21
CaETC3
CaMYB121
CaMYB63
CaMYB64
CaMYB62
CaMYB61
CaMYB65
CaMYB31
CaMYB83
CaMYB88
CaGLK1
CaMYB72
Ca7
CaMYB109
CaMYB3R-1
CaMYB73
CaRVE4
CaETC1
CaARR11
CaMYB47
CaEFM3
CaRL1
CaRAD2
CaMYB102
Ca8
CaMYB54
CaKUA3
CaEFM1
CaKAN7
CaMYB22
CaPHR9
CaARR13
CaDIV4
CaMYB12
CaMYB3R-3
CaMYB18
CaARR8
CaARR9
CaDIV12
CaTRF1
CaMYB116
CaMYB51
CaMYB28
Ca9
CaMYB56
CaMYB71
CaGLK2
CaMYB84
CaMYB42
CaMYB86
CaLHY
CaDIV8
CaA
CaMYB114
CaMYR2
CaRVE3
CaMYB29
CaTRF4
CaMYR1
CaMYBC1
CaRAD4
CaPHR7
CaPHR6
CaMYBC2
Ca10
CaMYB3R-4
CaTRF3
CaBLIND
CaDIV6
CaMYB48
CaMYB41
CaMYB78
CaARR5
CaMYB4
CaMYB90
CaMYB91
CaEFM2
CaMYB34
Ca11
CaARR3
CaMYB36
CaMYB37
CaAPL4
CaMYBR4
CaRAD3
CaMYB14
CaMYB117
CaMYB113
CaSWI3D
CaKUA2
CaMYBR12
CaDIV1
Ca12
