# Supplementary material for: Capsicum chinense MYB Transcription Factor Genes: Identification, Expression Analysis, and Their Conservation and Diversification With Other Solanaceae Genomes
Source: Front Plant Sci. 2021 Oct 13;12:721265. doi: 10.3389/fpls.2021.721265 (PMC8548648; doi:10.3389/fpls.2021.721265)
Supplement: Supplementary Figure 1 — Chromosomal distribution of MYB genes across 12 Capsicum chromosomes of (A) C. annuum and (B) C. baccatum. The capsaicinoid QTLs in C. annuum adapted from Han et al., 2018 are shown in shaded blocks. [file Data_Sheet_1.zip › Supplementary File S1.PDF]

Supplementary File 1: CDS and Protein sequences of *C. chinense* MYBs

>CcMYB3R-1 |Locus_ID=BC332_21354 |CDS_len=3117 |strand=-
ATGGAAAGTGATAGAATAAGCACTCCTTCAGATGGCACTAGCAGTAGTTTCCAAAGAGTTCGATCTTTGCATGGGAGAAC
TAGTGGTCCTACGAGACGTTCCACAAAGGGGCAGTGGACTCCAGAAGAAGATGAAATCCTATGCAAAGCTGTCCAACGTT
TTAAGGGGAAAAACTGGAAAAAGATAGCGGAATGTTTTAAAGACCGGACTGATGTACAATGTTTGCACAGGTGGCAGAAG
GTTCTTAATCCTGAACTTGTCAAAGGTCCATGGTCTAAAGAGGAGGATGAAGTAATAGCTGAATTAGTTAAGAAATATGG
CCCCAAAAAGTGGTCCACCATTGCTCAACATTTGCCAGGACGTATTGGAAAACAATGTCGGGAAAGGTGGCACAATCATC
TAAATCCTGGAATAAACAAGGAAGCTTGGACGCAAGAGGAGGAGGTGACTCTTATTCATGCCCATCAAATTTACGGGAAC
AAGTGGGCGGAGTTGACGAAATTTTTGCCTGGAAGGACAGATAATGCAATTAAAAATCACTGGAATAGTTCTGTCAAAAA
GAAATTGGACGTGTATTTGGCATCAGGTGCACTTGCAAAGTTCCCTACTCTGCCTAATGTCAACCATCAGAACCAATCAA
TCCCGTCTTCGATAAAGTTGCAACAGAGTAGTGAAGATGATAGTGTTCGTAAAGAAGGAACAGAAATGGAGGAAGTTTCA
GAATGCAGTCAAAGTTCAAATCTTGCTGGCTGTTCCCAATCTACAAGTGACATGGGCAACACATTTGTATATACAAGAGA
GGAGGGCAAATTGCTGGAGGATTCAAATTATAGGAAGGACCCAAGCGCCAGTTCAGCACCGTGTTCTGAATACTATACCC
CAGCTTTTGAAGATATTACTTTTTCAGTGGCAGAAGTGCCCAGTGAACTTGCTGAATCCAAGCTCCTGGAGCATAACTTC
TCACAGAACTGGGCAACATCCATGGGAAAAGATTGGCAGTTTAATCCAGATGACATACCTAATATTTCTCCTCTAGAGTT
GATGCAGGATTCTTCAGGTTTCTTCATGCAGTGTCTAACTGGTACTGGGAATCATGACATGGTAACCTTTCCACAGCAAA
ATGAAATGAAGTTTGAAACAACTAATGATGGGAGCATGGTTGTGGGGTTACATAAGCCGAATGAGATGTTTACTTCTGTG
GAGGGTTGCAGTATGGTATACCCTGAGGCAGGGATTCCTCAATGCTTTCCTTCTGAAATTGGTATGAATGGTGTAGATGA
AACTGCCGGTTCTATGATTTATCAGATCTCTGAAGGTGGAAATATGTCTATAGAAAAGTGCAATCCTCTCTTTTCAGATG
TTATGGGAACTGCATCCTGTCAACCCTGTTCTGTTCCTTCACAGTTTTCTTCAGAGCAAAGCTCACACATGTTTGGTATT
GCCTCAAATCAGTTTCATAACCCTTTGCACACAGCTCGTGAGTCCCAAACAAATAAATGTGATGGTTTTCTATATCCCAT
GGAATCTGATACTCCTTGTGACAACATAATGGATAATCCTCTCCTGAAAGACCAACTGGATCAAACGTCAGATTCTGTAC
AGCTAGTTTCTGTGAATGATTTTCGCTCAACTCCTTCAAATACTATTCAAACATGTCCATTGGTAAATGAAAATTCAACT
GTAAAGGAAGACCAGATGGATGGGGGGGCCCTATGCTATGAGCCTCCTCGCTTTCCGAGCTTGGATGTTCCATTTTTCAG
TTGTGATCTTATACAATCTGGTACAGATACACACCAAGAGTACAGCCCTCTTGGCATCCGCCAGTTGATGATGACTTCTG
TGAACTGTCTTACTCCATTTAAGTTGTGGGATTCACCAACAAGAGATGGTAGTCCAGATGCCGTCTTGAGAAGTGCTGCC
AAAACTTTCACAAGTACACCTTCTATATTAAAGAAACGGCACCGTGATTTGGTGTCACCTTTGTCAGAAAAGAGATGTGG
AAAGAAACTTGAAAGCGATCTCTGTCAAGAATCATTTTCTAATCTGACTAAGGATTTTTCTCGACTAGATGTTATGTTTG
ATGACTCTGCAAATGAAAAAGCAATGCCTTCCCTAACTAAGGATCAAACTCTGGAACTTCAAGCATCTGAAGATAAAGAA
AACATAAATCCAACTGAAGATGGACAAAAGGAGGAAGACAAGGGCCGAGATGGGCATTCAAGCTGTGGAACTTCTGGGAG
ACAGTTAGATGGAGGTGAAGTTCACTATAAGGATCAAGGAACAAGGGAGAGCAAAAGGCTTGGAGCCAAAGATGCAGTTG
GAAAGATAAAACAGCCTTCTGGAGTTCTAGTTGAACTGAACGCAAGTGGTCTGTTCTTTTCTCCTGATCGTTTTGGAGCC
AAGTCTGACAGAGGTTCAAATCTAAGCAATAAAGCTCTAGGAAATCAGTATTCTAGACGACTCGAAGCTGCATCAAATCA
AGGTTCTGTTTCATCTTCATTTGAGACTTCAAGTTTCTCTGTTATTTGCTCTCCTCGTATACATGGAAAGAAAGATGGAA
GTAGTTTTGTTATCACTACATCGATGCAATCTGCTCCAACATCAACTGCCTTGGAGAGCTCAGCTGAAACTTCAGGAAAT
GGAGCTGGAGCTGGGATTATTGCAAGCATATCTGGAGAAACACCTTATAAAAGGAGTATTGAATCTCCCTCGGCTTGGAA
ATCTCCATGGTTCCTCAACTCTTTCCTGTCAAGCCCGAGACTTGATAATGAACTTACTTTTGAGGATCTTGCACTGTTTA
TGAGCCCAGGGGACAGAAGCTATGATGCTATTGGATTAATGAAGCAATTGAGTGAGCAGACTGCAGGAGCATTTGCAGAT
GCCCAGGAGGTCTTGGGAGGTGATACTCCAGAGTCAATCCTACGGGGGAGGAACTCCAAAAACCAGAATGCAGATGAAAA
TCGTTCTCTTTTGTCAGCCAATGCTATGAGTGAGCGGCGTACACTCGACTTCAGTGAGTGTGGATCACCTGAAAAGGAAA
AGGAGACTGAAAAATGTAGTACAAACAACAACAGCTTTTCAAGTCCTTCTTCCTACCTCTTGAAAGGCTGCAGGTAG

>CcMYB3R-2 |Locus_ID=BC332_00348 |CDS_len=1902 |strand=+
ATGATACAAGTTAAAGAGGAAGCTTTGACTCTGGAATTTGGTGGATTCACTTCTTGCTCATCTTTTTCTGATAGCAGTTA
TGAGGCTAGCACTCCGAGATGCTCCTCTGAGCCTGGTTCCAGTTGTCAAAGGAGCTCAGGTCAAACTAAACGTTCTTCTC
AGGCAGGCTGGACAGCAGAAGAGGACAATCTGTTGACTGAAGTGGTGAAAAGGTTCAAAGGAAGAAACTGGAAAGGAATT
GCTGAGTGCTTGAATGGAAGGACTGATGTGCAGTGCTTGCATCGCTGGCAGAAGGTTCTGAATCCTGAGCTTGTAAAGGG
TCCTTGGACAAAGGAGGAGGACGACCATATTATTGAGTTAGTTGAGAAATTTGGCTGTAAGAAGTGGTCCATTATTGCCA
AGTCTTTGCCTGGTCGCATTGGCAAGCAATGCCGGGAAAGGTGGCACAACCATCTTGACCCAACAATAAAAAGAGATGCC
TGGACTGAACTGGAAGATTTGGTCCTATGCCGCTATCACCAAATATACGGGAACAAGTGGGCTGAAATTGCAAGGTTTCT
GCCTGGAAGGACTGATAATGCAGTTAAAAATCATTGGAATTCTACAGTGAAGAAAAGATTGGACTTGAATTTACTAAGTA
GGTTAGGGCCGGATATCCAAAGTGAGGGATCTCCAGATTTCTCAAGTCACAAGAAAAAACTAGAGATCAAGAAGCATTTA
GCACAGGCTCATAATGCAGAAACACTTTCTTTGAGCAAGCAGGCAGGAGTAGATAATGCTGCTGATGCTTGCTCAACTGA
TCTAAGAATTGGGTGTGCTTGTTCTCCTCGAAATGATTTGCACAAGGGTACTTCTTTATTTGGAGCCTGTAAATCATCAG
TAAAGAATGCAAGGGATCTGATAAAGCCACTTGGTGGAATACAGTTTGGCAAGGCAGAAATTCTTCCAAATGGTGAGACC
GACCAATCCAATTTAAATCTTACTAAAATATCAGATCCTCTCTTGACCTCTTTAGATTTTCCTTTGGATCCGTCACGCAA
GACCAGATGGAGTAGTTCTCAAGTTAAGGCTGTTCACCCTACTACTTTTGGGAGAATGTATGAATCTCCCAAAAGGTCTA
AGTACGGCACTGTTAGTGACACTGACCATGATTTTTTGACTTTGTCATTGGCTGGCTTCACCGAGGTTCATTCCCAAGGT
AACAAGAAGAATAAAGCATATGATATACAATCTTCTTTGGCTCTCAAGCAGCCCGGCTTCACCGAGGTTCATTCCAAAGG
TAACAAGAAGAATAAAGCATATGATACCCAATCTTCTTTGGTTCTCAAGCAGCCCGGCTTCACAAAGATTCATTCCCGGG
GTTACAAGAAGAATAAAGCATACGATACACAATCTTCTTTGGCTCACAAGCAGCCCGGCTTCTTGAATTATGAACCACCA

AAAGTAAAGGACTTGACGATTCCATTAACGGATGAAAACCTTAGTAGAGACAACATTATCGGGCGACAAAATAGCCATCT
ACTTTGCTCTACACCTCTTAGTTTAACAGTCTCTGCTAATAGTAGCAGTCCAGAATCTGTTTTAAGGAATTCTGCAATGA
GCTACACAAAAACTCCTTCAATCATAAGAAAGAAGACTTCGAGATTTTCAGAAGCTTCTGGGCATTATAGTTGCACAGGC
ACCACCACACCCATGCATTTTCTCCGGAGTGTTCCCGACAGGGAAGACACCTCAAACCTGAAGGACAGGATTTCTGGATG
TAAAAGATCTGTTTCAGGAAAATCTCTTGGAAGACGACTGGAATATGCCTTTGACATGGAATGGGATTCTTCTAGATGTT
GCACGCCAGTTTCTGCAGCTTCACCTTGTGCTATTGGAGCTAATACAATGTTGACACCATAA

>CcMYB3R-3 |Locus_ID=BC332_23333 |CDS_len=1356 |strand=+
ATGGATGAATTGCAAGTAGAGGAATGCTGTATTGAGAATAAGCAATCAGTTGCTGCTTCGAGCTCGTCGGTTTCTGAAAA
TAGTGGTAGTGTTAATTTAAGGTCACCAGCAGTATCTAGTCCAACCCCTGTATCACCAACTCACAGGAAAACCACTGGTC
CAATAAGGCGAGCAAGGGGTGGTTGGACACCTGAGGAGGATGATACTTTGAAAAAGGCTGTTTCAGTTTACAGAGGCAAA
TGTTGGAAGAAAATAGCTGAGTTGTTCCCGGATAGATCAGAAGTGCAATGTCTGCATCGCTGGCAGAAGGTTCTCAATCC
AGAACTTGTTAAAGGACCCTGGACTCGGGAGGAGGATGATAAGATTATTGAACTGGTCGCTAAGTATGGGCCGATAAAAT
GGTCTGTCATAGCTAAATCATTGCCTGGTCGAATAGGGAAGCAATGTCGTGAGAGATGGCACAATCATTTGAATCCCAAT
ATCAAAAAGGATGCTTGGACACTGGAAGAGGAACGGGCACTTATAGATGCTCATCGGATCCATGGTAACAAGTGGGCTGA
AATTGCTAAAGTTCTGCCAGGAAGGGCTGATAATGGAATCAAGAATCATTGGAACAGCTCTCTGAGGAAGAAGTTAGACT
TCTATTTAGCTACTGGAAATCTTCCATCTGTTAAATCTGAAGAGCTACTAGTTTGCTCAATTAAAACATCAGAAGCAGCT
GACGTAGTTTCATCAGGAGCTATAGATATATGTAAAATAGAAAACGGATGGCAGGTGACAGAAATCAATGGTGGTCCTCA
AGCTGAATCCACTGATTCTGAAATCGTCAGGTTGGAAGCTCAATCACCAGAAGAAGATGCTATTCAACACATCAAACAAA
GTACAGAATCAGAAAACAGGTGTAATGGCTGGAACATAGATAGTGCAGTAGGTCGATTACAAGTTTTTGAAGCACCATTT
CCTTGTGAAATCTCGACATGTGGTAGGTTATACTATGAACCACCAAGGTCAGAAAGTTGTATTCCACTGGATTCAGATCT
TCTAAATATCGGCTGGGCACAATGTGCATCTGATGCAAGTCCAAGCCCGTCACCAAATGGTTTCTTCACTCCACCTAGTA
CAAAGGACCATAGCCTATATGCACAAACTCCTGAATCTGTACTTAGAACTGCTGCTAGAAGCTTCAATACGCCGTCCATA
TTAAGGAAAAGAAAGTCTCAAGGTAAACTTTCGACGCCAACTAATAAAATGAGGAAGTCAGATGGAGATTTCTGTAAGGA
CAAACTTATTGATGCGTGTGACAAAGTCCAATTCGACAGCTCAGACAAATCTAGCATGGATAATGGAGTTTCTTAA

>CcMYB3R-4 |Locus_ID=BC332_29224 |CDS_len=2988 |strand=-
ATGTTGGATTATCAAGATTTGTCTTTGTCTCAAGCAACCATGGAAAGTGATGAAACAAGCAAGCCTCCTTCAGATGACAT
CAGTAGTCGGCCTTTGCATGGGAGGACGAGTGGTCCTAAGAGACGTTCCGTAAAGTGGACTCCCGAGGAGGATGAAATCT
TGCGCCAAGCTGTCCAACAGTTTAAGGGGAAAAGCTGGAAAAAGATTGCGGAATGTTTTAAAGACCGGACAGATGTTCAA
TGCTTGCACAGGTGGCAGAAAGTTCTTGATCCCGAACTTGTGAAAGGTTCATGGTCTAAGGAGGAAGATGATAAGCTAAT
CCAATTAGTGAACATACATGGCCCCCAAAAATGGTCCAATATTGCACAAGAGTTAGCAGGACGTATTGGAAAGCAATGTC
GGGAGAGATGGCACAATCATTTGAATCCTGAAATAAAGAAAGAAGCTTGGACAGAAGAGGAGGAGTTGACTCTAGTTCGT
GCCCACCAAGCTTATGGAAACAAGTGGGCAGAGATATCAAAATATTTGCCTGGAAGGTCAGACAATGCAATAAAAAATCA
CTGGCATAGTTCTGTCAAAAAGAAACTGGCGTCAGGTTTACTTGCACAGTCCCCTGCTCTGCCTAATGTCAATCATCAGA
ACCAATCAATCGCTTCTTCTTCTATGAAGTTGCAAGAAACTAGTGAAGATGAAAGTGTTCATAAAGAAGGAGCGGAAGTG
GAGGAAGTTACAGAATGCAGTCAAGGTTCGACTCTTGCTGGCTGTCCCCAGTCTACAAGTGACTTGGGCAACACATTTAT
GCATATAAGAGAGAATGATGCAATGTTGGAGGAATCAATCCATAAAAAGGACGCAAGCTCCGGTGCAGCACCATGTCCTA
GGAACAGTGCCTCAGCTTTTCAAGATGTCTCTTGTTCAATGCTAAATGTTCCCAGTGATCTTGTGGATTCCAAGTTCCTC
GAGTACATGTTCTCACATGACGGGGACAATTCCACAGAGAGAGATTGGCAGTTTAATATGGATGACTTTCCAGGGTTTAT
TGAGGAATTTTCAGGACATTACATGCACTGTTCGAATGACAATGAAAACCATGCTATGGTAGCCGATCCATTGCAAACTG
CAATGGGATCTGGAGCAACTTCTAATGCAGCGAACATGGTTGAGGGTCCATATAAGCCCAATGAAATGTTTGATGGTTGC
AGGATCGTGTATCCTGAGGCAGCAATTTCTCAATGCTCTCCGTCCGAAACTGGGGTTAATGGCACTGGTGAACCTGCAGA
TTGTTTAATTTACCAATCATCAAACTATCAGATCCCTGAAGAAGGAAATATGGCTCCAACAAACTGCAACGCTTTAAGTT
TTGATGACGTTGAAGCTTCAACACATCAGCAATTTTCTGTTCCTTCGCACTTTTCTTCTGAGGATAGATCACTTGTGTTT
GGTATTGCTTCAGATCAGTTCCATTATCCTCCGCTTGAGAACTCAGTTCTAGAGTCCTTCACCAGTAGATGTGATGATTT
TATATGTCCAAGTGAGTTTGGCAGTCCTTCCAATGACAATGGCATAGACAATGCCGTCCTGAAAGATCTACCTGATCATA
CAAAAGATTCCTCAAGGCTAGAAGAGCAGAAAGATGGAGGAGCTCTATGCTATGAGCCTCCTCGCTTTCCAAGCTTGGAT
GTACCCTTCTTGTATTGTGATCTTATACAATCTGGTTCAAATACACAGCAAGAGTACAGCCCTCTTGGCATCCGCCACTT
GATGATGAGTCCTGCGAACTGCCTTACTCCATTAAGGTTGTGGAATTCACCTTCAGGAGATGATAGTCCCAATGCTATCT
TGAAAAGTGCTGCCAAAACTTTCACTGGGACACCTTCTATAGTAAAGAAGCGACAGCGTCACTTGGTGTCTCCTCTATCA
GAAAAGAGATGTGAGAAAAAACTTGAAAGTTATCTAAATCAGGAATCATTCTCTAATATGGCTACAGAAATTTCCCGAGT
AGGTGATATGTTTAATGAGTCGGCTAATGAAAAAGCGTCTACTGAAGACAAAGAAAATTTACATCCATCCTCAGAAGATG
GAAGAAAGGAGCAGGGTGATGGAGTAACTGGACTTTCTTGCATTGAAAATTCAGAGAGGCAATTAGATGGTGGTGGTGCT
CACTACCATAAAGATCCCAATAGTGAAAGTGCTGGAGCCAATGATACACTTGGAAAGGTAAAACAGCCTCCTGGAGTTCT
GGTTGAGCGTAGCTCAAGTGATATGCTCGTCTCTCCTGATCGTTTTTTGATCAAGTGTGATAGAGCTACAAGTCTAAGTA
ATAAAGCTTTAGATAACCAGTACGCTGGACAGCTTGAAGCTGCATCAAATCAAGTTAATGTTTCATCCTCGTTTGAGACT
TCATTCTTGTCTGTTTTTTGCTCTCCTGACATACTTGGGAAGTGTCGAAGCAGTGTTGTCAAAGCTACATCAATGCAATC
TACTACATCAACTGCATTGGTGAATACAGCCGAAAATTCTGAAAAAGGATTTGGTGCTGAGACTTCAAACATATTTGGAG
AGGCGCCTCTTAAAAGGAGTACTGAATCTCCTTCAGCATGGAAATCGCCATGGTCCTTGAACTCTTTTCTGCCCAGCCCA
AGATATGACACAGAACTTACATTTGAGGATTTTGCGCCTTTTATGAGTCCAGGTGAAAGAAGCTATGATGCTATTGGGTT
AATGAGGCAATTAAGTGAGCAGACTGCACCTTCAATTGCGGATGCCCGTCAGATCTTGGGAAGTGAAACTCCAGAAACAA

TTCTGTCGAAGAGAACCAAGCAACAGAAAGCAGATGAAGATTGTGCCCTTCTGGCTTCAAATGCAATGAGTGAGAGACGC
ACGCTTGATTTCAGCGAATGTGTAACACCGCCGAGAAAGGGCAAAGAGACTACCAAATTTGGCAGCGTTTCAAGTCCTTC
CTCCTACCTGTTGAAATGTTGCAGATAG

>CcMYB3R-5 |Locus_ID=BC332_25921 |CDS_len=1689 |strand=+
ATGTTAAATGGCAAGATACTAATGAGAACCACTGGTCCAATTAGACGAGCAAAGGGTGGTTGGACTCCTGAGGAGGTAAT
CCGTTTTCCTGATAAGAATCATCTATTGATTATAAACTCCCCAATGCCACAGGACGATACTTTGAGAAGCGCTGTTGCAG
CTTACAGGGGGAAGTCCTGGAAGAAAATAGCTGAATTCTTTCCGGATAGATCAGAAGTGCAATGCCTGCATCGATGGCAA
AAAGTTCTGAATCCAGATCTTGTAAAAGGACCCTGGACTCAAGAGGAGGATGATAAGATCATTGAACTGGTGGCTAGGTA
TGGGCCTACAAAATGGTCTATCATAGCCAAATCATTGCATGGTCGGATTGGGAAGCAATGCCGTGAAAGGTGGCACAATC
ATTTGAATCCAAATATTAAGAAAGATGCCTGGACATTGGAGGAGGAACTAGCACTTGTAGATGCTCATCGGATCCATGGG
AACAAGTGGGCTGAAATAGCTAAGGTCCTACCTGGAAGGACTGATAATGCAATCAAGAACCATTGGAATAGCTCTTTGAA
GAAGAAGTTAGACTTTTATTTGGCTACTGGAAACCTTCCACCTGTTGGAAGAGATGGTCCTCAAAATGGTTGTGAGAACA
ACATCTTTACAGCTTCGGCTGAAAAGGCTCTTGTTTACTCACATAAAGGATCAGATTCAACTGAGCTTGCCTCGTCAGGA
ACTACTGACATATGTAAAATAGGAAATGGTGGTATGAATCATCAAGATGCCAAGCTGCTGGTTGCAAATATCGATGCTTC
CAATAGTGGTCCTCAAAATGAGTCCACTGATTCTGAAGTTGTTAGATGGGAGGCACAGTCACCAGCAATAGATGGTATCC
ACGTTAAACCAATTACAAAAATCAAGTTTGATGGATATGGAACAAATAGTGTCGTTGATCTTAAAGTCATTGAAACTCCA
TTTTCTTGTGAAATACCTACATGTGGTACACTGTATTATGAACCACCACAATTAGGAAGTTGTATTTCAATGGATTCAAA
TTATCTAAATATTCGATGGGCTCATTCTGGCCCAAGTCCAAGTTTATCACCTAGTTCTTTCTCGACTCCACCTAGTAGTC
TGAAGGGGAGTAGTTATGCACAAACCCCTGAATCCATATTGGAAACTGCTGCTAGAAGCTTCCCAAATACGCCTTCCATA
TTCAGGAAGAGGAGGTTGAGCTCCACCACGCCAGGTAATAAAATTGGTAATGCAGATGAAGATATGCGTAAGGAAGAACC
ATGTGACACTTGTGAGGAGAAACAAGTGGACAGTTCAGAAAAATATGGCATGAATAGGGGAAGTTCTTCGAGAAATCCTG
CTTACAATGGCATTGGACCATGCAATACCAGAGCTTTTAATGCCTCTCCTCCATATCGGTTAAGGTCTAAACGAAAATCC
ATCTTCAAGTCTGTGGAGAAAAAGCTTAAGTTTGCATTTGACAACGAGCAGCACAATACTAGTAATGATTCAACTATCAA
AGAGATATCTCATGTAGCAGATTGTTTACGTGCAACAAAAATGGGTTTTATGCTAGAATCAGTGTTTAACCCTGGTATTA
TCCGTGACACTGGCTGCCATGCTGTTGGATTTTTTGTAAAACCTCATCAACTTCCAGAAGAATTCACCTTGCATATTCAT
TTATGTTAG

>CcMYB5R |Locus_ID=BC332_09390 |CDS_len=2985 |strand=-
ATGGCCTTCGATTCCGACGACGATGATTTCTCCGGCACCGATTCCGATGAAGGCTTCCAAGCAGATATGGAAGCTCTCAA
AAAAGCTTGCCTTCTCTCCGGCAAAGACGCCGACGACCTACAACCTTCATCTTCCTCCGCCGATGGACACGTCGCCGGCG
CGAATGATGTAACGCTCTCTGATACTGACGCTGATGAGGATGAGGATGATGACTTTGAGTTGGTGCGTAGTATACAAGAG
CGATTTGCCCTATCAACGGAGGTTCACGACCCTATAAGTATGAAGCCGTTGTGCTCTATTCTTCCACCTGGTTCAGAGGG
TGATGAGGACGATGATTTTGAAACCTTACGTGTTATTCAGCGCCGATTTGCGGCTTACGGTGACGATAATGGGAATGGAA
GAGAGGAGAGTCTTTTTGATAAATTTGAGCAGATTTCCGAAGAATGCAGTAATGACGTTGCTAGGTCTAAGAATCTCACT
GATGGACATGATTCTGATGCTGAAACCACAGCTATATCAGTTAATTCTTCTCGCTTCCCAAAATCAGCACATGCATTTGT
TGATGCCATAAAGAAGAACAGGGCTTGTCAGAAACTTATTCGAGAGAAGATGATGCAAACTGAAGCAAGGTTGGAAGAGT
TGAAAAAAATTAAAGGAGCGGGTCAAGATCCTAAAAAGCTTTCAGCTCACTTGCAAAAAGAAAATGGGGCGAGCATTGTCA
CAGAAAAGAGATGCTCGTGTCCAGTTAATTTCTTTGCCAAAGCAAAGGTTCAGTGCAAAGCTCCCGGGAAAGAAGTTATC
TGCAATACATTCTGGACCTCCTGAGAATTCTCATGTTGCTAGCTTTAAGGAGGCATTGACACAATTTGCTGTCTCATTGA
GTCGCAAGGATTGGTCCAAGGAAGAGAGTGAAAATCTTGCGAAAGGAGTGAAACAACAATTTCAAGAGATGTTGCTTCAA
CGCTCGGTCAATTTACCTGTCAATCTACTAAGTGATGAGGATGGATGTTCTAGAGAATCTGGTGATCTTGATGACGTTAT
TGCTTCAATCAGAGACCACAAGATTACCCCCGAGACCATGAGGTCATTTATACCCGAGGTCAATTGGGATCAAGTAGCTT
CCATGTATCTTCCTGGTCGTTCTGGTGGTGAATGTCAGTCAAGGTGGTTGAACTGGGAAGACCCCTTGATTAAACATGAA
GGATGGGATTTATCAGAAGAAAAGAACCTATTGCAAGTTGTCCAGCAGAAGCGAATGAGCAACTGGGTTGATATTGCTGC
ATCATTAGGGGTGTCTAGAACTCCTTTTCAGTGCTTATCACATTATCAGAGGAGTTTGAATGCTTCAATCATTAGGGGGG
ACTGGACAGATGAGGAGGATAGTAGACTCTGTGCTGCTGTGGAGACTTTTGGTGAGAGTAACTGGCAGGTTGTTGCTTCT
GTCATTGAAGGCCGAACAGGCACTCAGTGCTCCAACAGGTGGATTAAATCTCTTCATCCAGCAAGGAGAAGGTGCGGGAA
GTGGAGTGCCGATGAAGATAAGCGTTTGAAAGTTGCTGTAATGCTTTTTTATCCTAAATGTTGGAGGAATATAGGTCAAT
CTGTGCCTTGGCGCACTCCAGTTTGGAAGAAGGTAGCTCAATATGTACCTGGGAGGACTCATGTACAATGTAGAGAAAGA
TGGGTCAATACCTTAGATCCTTCTTTGAAGCTGGATGAGTGGACAGAAGAGGAGGATTTGAAGTTAAAATCAGCCATTAA
TGAACATGGATATTCCTGGTCCAAGGTTGCTGCATGTGTTCCTTCACGTACTGATAATCAGTGTCGCAGGAGATGGATGG
TGCTATTTCCAGATGAAGTTCCTATGCTGAAAGAAGCTAAAAAGATACGAAGGGATGCTTTTATTTCCAACTTCGTTGAT
AGGGAGGAGGAAAGACCTGCCCTTAAACCCAATGACATAGTTCTGGCACATAAACTTAGTAAAGCTGGATGTGAAACTAC
TTCTGCTAATAAAAAGCGGAAACGACGGCCCAGAACAGCAAAGGATGATAAGACTCCCAAGTGTGATGCAGTACGTGAAA
TGGAGAAGCAACACTCGGAGGGGAGTGAAGGTCCAGAGTCTTCTGATCTTATAAACAACCAGGAGAGTGATAAGATTTTG
GATGGTGAAGAGGCGGTTGAACAGGGTTGTAATGATGCAAGAAAGAATAAGAGACGGTCTAGACGGCATCCGAGAAATAC
AAAGAAAGTTAAACCAAATGATAAAGTTCCAGAGGCTTCTGCTTCCAGTGCGGGTGAGTCAACTGTTGCAGATGACAATA
TCTGCAAGAGGAGGAGAACCAGTTCGCTAGTGAAGAAAAAGAGTAGAACTATCGCTTCTGCTTCCATTACAGTGGATTCT
ACTATTGCAGATGGCAATAGCTGTAAGAGGAGGCAGAGAACCAGTTCGCAAGTGAAGAAAAAGAGTAGTGATAAAAATGC
AAGACGGTTAGAAACTTCTGCAGTACCTGGCACTCTGGGAGGTGGCACTTCTGGAGCTTCCGTCCGTCACAAGCTTAATA
AATGCAATCAATTGAAGAATGATGATAGATCTTGCGTAGGGAATCTGCCGGAAACAGTAGGTGACTGCATGACCCTTGCT

TCCTTTGTCCGTAAATCACGAGCAAAAGGATGTTCATTGTCTTTTACAAAGGTTGCCAGGTTGCATCCAGACAAAGCTCA
AGGTAAGGCTATGGCTGGAGATCACTCTAGCAGATCTTGTATATCTGGTGGTCATGATGAGACGGAGAAGAGAACTAGCC
AGGAATGCACAAGTTCCAATCAGATATCAGGGACTGAAGTAGGAGATGACATGCCCCTCTCTCTTTTTATGGGCAAAGTG
AAAAGGGAGAAGATTGAAGTAGGGGATGACATGCCCCTTGCTTTTTTTATAGGCAAAGTGAAAAGGGGGCAACCATCTGG
AGCACAAGATAGGAAAAGGAAGTAG

>CcMYBCDC |Locus_ID=BC332_12725 |CDS_len=3468 |strand=-
ATGAGGATTATGATAAAAGGAGGAGTATGGAAGAACACGGAGGACGAGATTTTGAAAGCGGCGGTAATGAAGTATGGGAA
GAATCAATGGGCTCGTATTTCGTCTTTGCTTGTTCGTAAATCGGCGAAACAATGTAAAGCTCGCTGGTATGAATGGCTTG
ATCCTTCAATTAAGAAGACTGAATGGACTAGAGAGGAGGATGAGAAACTACTTCACCTTGCAAAGCTCCTGCCCACTCAA
TGGAGGACAATTGCTCCAATTGTTGGTCGTACACCATCCCAGTGCCTTGAACGTTATGAGAAGCTTCTCGATGCAGCATG
CACCAAGGATGAGAACTATGATCCTAATGATGATCCAAGAAAATTGAAGCCTGGAGAGATTGATCCTAACCCGGAATCCA
AGCCTGCTCGGCCTGACCCTGTTGATATGGATGAGGACGAGAAAGAAATGCTTTCTGAAGCACGAGCTCGGTTGGCCAAC
ACAAGAGGCAAAAAGGCTAAAAGGAAAGCCAGAGAAAAGCAGCTTGAAGAGGCGCGAAGGCTTGCTTCTTTACAGAAGAG
GAGAGAACTGAAGGCTGCTGGAATAGATGTCCGTCAAAGGAAGAGAAAAAGGCGAGGTATTGATTACAATGCTGAAATCC
CCTTTGAAAAGAAGCCTCCTCCAGGCTTCTATGATGTTACTGAGGAAGATCGTCCAGTTGATCAACCCAAGTTTCCAACT
ACCATTGAGGAACTAGAAGGTGAGAGGAGAGTTGATAAGGAAGCTCGCCTAAGAAAGCAGGATATTGCGAGGAACAAAAT
TGCTGAAAGGCAGGATGCCCCTACATCCATATTGCATGCGAATAAACTTAATGATCCAGAAGCAGTGAGGAGGAGGTCGA
AACTGAATCTTCCTGCACCACAGATTCCAGACCATGAATTAGAGGCCATAGCAAAGATAGGTATTGCCAGCGATTTAATA
GGGGGCGATGAACTGTCCGAAGGAAATTCCGCAACGCGTGCTCTTCTTGCAAACTATGCCCAGACACCACAACATGCAAT
GACTCCTATGAGAACACCTCAAAGAACCCCTTCAACTAAGCAAGATGCCATCATGATGGAGGCAGAAAATCAGCGAAGAT
TGACTCAATCTCAGACACCATTACTCGGAGGGGATAATCCTCTGTTGCACCCCTCAGATTTCTCGGGAGTCACTCCTAAG
AAAAGGGAAGTGCAAACACCAAATCCACTTTTAACTCCTTCAGCAACTCCTGGAGCCACGGGCCTTACTCCTAGAATTGG
CATGACGCCGTCCAGGGATTCTTATGGCATGACACCCAAAGGAACCCCTAGGGATGAGCTACGCATTAATGAAGAAATGG
ACAATAATGCTAAATTTGGGCAATTTAATTCAAAAAAAGAATTACTTTCTGGTTTGAGAGGCCTTCCTCAGCCCAAGAAC
GAGTACCAGATAGTCGTCCAACAACCTCCTGAAGAAAATGAAGAACCAGAAGAGAAGATTGAAGAAGACATGTCTGATAG
GATTGCTAGGGAGAAGGCTGAAGAAGAAGCAAGGCAACAAGCTTTACTCCGGAAAAGGTCAAAAGTATTGCAAAGGGAGC
TCCCTAGACCTCCTATTACTTCACTAGATCTAATTAGAAGATCCTTAATGAGGTCTGATGAAGACAAGAGCTCCTTTGTT
CCTTTTACACTAATTGAGCAGGCTGATGAAATGATTAGAAAAGAACTTGTGTCGTTGCTGGAACATGATAATACCAAGTA
TCCTCTAGATGAAAAAGCAGAGAAGGAGAAGAAAAAGGGGGTCAAGCGAAAAGCGGTTGCTGAACCTGCTATTGAGGATT
TTGAGGAAGATGAACTAAAAGAGGCTGATGAATTGATCAAAGATGAAGCTCAGTTTCTTCGTGTGGCAATGGGACATGAG
AATGAATCTCTTGATGACTTTGTCCAAGCACATGAAACAACTTTGAATGATATCATGTACTTTCCTACCCGGAATGCTTA
TGGTCTCTCAAGTGTTGCTGGAAACATGGAAAAGCTGGCTGCTTTGCAGAATGAGTTTGAGAATGTGAAGAAGAAAATGG
ATGATGATACTAAGAAAGCAACAAAACTTGAACAAAAGATCAAAGTTCTCACTAATGGATATCAGATGCGAGCTGGAAAA
CTTTGGTCAGAGATAGAGTCAACCTTCAAGAAAATGGACACAGCTGGGACAGAGCTTGAATGCTTCCGAGCATTACAAAA
ACAAGAGCAGCTAGCAGCAACCCACAGGATCAACAATATGTGGGAAGAAGTTCAGAAGCAAAAAGAGTTCGAGCGTACTT
TACAGAAAAGGTACGGTGACCTCGTAGCAGAAAAGCAAAAGATTCAGCAACTCATGGATGGGCATAGAATACAAGCTCAA
ATTCAGGAAGAGAATTGCAGCAAAAAATCGTGCTCTTGAGTTGGCCAAAGCAGAAATGGCAGAAAAGGATCGTGCCCTTGA
GTTGGCCAAAGCAGAAATGGCTGAAAAGGAGAGTGCTCTTGAGTTGGCCAAAGCAGAATTGGCAGAAAAGGAGAGTGTAC
CATCTGCTGGTGATGTGGAACCTTCAGGTACTGGCCAGTGCTCCAATACAGAGGAAATTTCTGCTTCTGCCTCTCATGTA
CCAATAGAAGCTGATGTGCATGTGGAGCCTTCAGGTACCGGTCAGTACTCTAATCCAGAGGAAAATCATGCTTCTGCCTC
TGATGTACAATCAGCTCCAATTGAAGCTGATTATGTGCCTGTGGAGCCTTCAGGCACCTCTAATGCAGAGGAAAATTCTG
CTTCTATTGAAGATGATTATGTGCTTGTGGAGCCTTCAGCTACATCTAATGCAGTGGAAAATTCTGCTTCTATTGAAGCT
GATGATGTGCATGTAGAGCCGTCAGGGTCCGGCCAGTGCCCCATTGCAGAAGAAACTTCTGCTTCTGTCTCCCATGATGT
AGCTCCTCTAGACGTTGAGGGACAGGTGAAAACGGGTCGTGTTTCCGCAATGGATGTTGAAGCCACAAGTGACCATGTAC
CTATGGAGGGTCAGCAAAACCTAGTAGAAGAGAGTAATATGGATGTTACTAAAACAGAAGACTCTGGAGCAGTAGCAGGT
GACGCGGATGTTCCTAAAACAGAAGACTCTGGACTAGTAGCAGGTGACACGGACGTTTTTAAAACAGAAGACTCTGGAGT
AGCAGCAGGTGACGCGGATGTTCCTAAAACAGAAAACTCTGGAGCAGTAGCAGGTGACGCGGATGTTCCTAAAACAGAAG
ACTCTGGACTAGTAGCAGGTGACATGGACGTTTTTAAAACAGAAGACTCTGGAGTAGCAGCAGGTGACGCGGATGTTCCT
AAAACAGAAGACTCGAGAGTAGTAGCAGGTGACGCGGATGTTCCTAAAACAGAAGACTCGGGAGTAGTAGTCACATGTGA
TGGGGAGGCTAATGCCGGAAAGTTGTAA

>CcMYB10 |Locus_ID=BC332_11130 |CDS_len=930 |strand=-
ATGGCAGAAGGTGGTGGTTATGGTGATGATACAAGAAGTAGTTGTTGTCCACGTGGCCACTGGCGGCCAGCCGAAGACGA
AAGGCTCAGGCTGCTTGTTGAACAATATGGTCCTCAAAATTGGAACTCTATTGCTGAAAAACTTCAAGGAAGATCAGGAA
AGAGTTGTAGATTGAGATGGTTTAACCAACTCGATCCAAGAATTAATAGAAGGCCATTTACAGAAGAAGAAGAAGAGAGA
CTTGTTGCTGCTCATAGAATACATGGCAATAAATGGGCATTAATTTCAAGATTATTTCCAGGTAGAACTGACAATGCTGT
GAAGAATCATTGGCATGTCCTAATGGCAAGAAAGCAAAGGGAACAATCCAAGATTTGTGGCAAAAGAAGCTTATACCAAC
AACAACATGATAATTTTCTAAGTGATTCTAAATCACCCTCCTATGGTTTTCGACGAAGGAACATCAACAACAATAATAAT
ATGACGAAAACACATGAAGGTAATTATGGCTCCAAAATCAACTTCTTTGAATTCCAAAACCCTAACAAAGATAGGGTTTT
CTCAATGTCTTCTACATATTCTTCTAATTCACATGACTTTTGTAGAAGAAATGGGAGCCATTTGGTTAGAGAAAGCTCAA
TAGACCAAAAATCTTTATGTCAAAATAATTTAAGCTTTTTAAGCCATGGAGGCGGAGCTAATTATAGTTACAAAAGGAGT

ACTATTATTCGAAACCCTTTCAGTTATGCTGATAGAAATGAATCTAACGGCATAACAGAAAGGGTTGTGAATATTACCGA
TGGTAAATTTTCCTTAGAGAAAATGCTTAGGGAAAGCATTGAACAACAACAACAACAGTATGGAGAAGAAGCAAGGGAAA
GGAAGGATATTCCCTTCATAGATTTTCTTGGTGTGGGGATTTCTTCTTGA

>CcMYB116 |Locus_ID=BC332_22985 |CDS_len=2457 |strand=+
ATGCAAATGGTGCAACAGGTCTCCTTAGACTGTGACACATGCCTCCATCCCGAGACTTTTATGTGCAAGGATGGGTTTTC
TGGGGTAACACAAAATCCTGCAGTGGCACTGCCAGTGATACCCTCAAAAGATCATCCTGTTTCACAGCAGTCTGTGCTGA
CCCATGATCATGCAGTTACACAGCTGGTATCATTCTCCCAAGATCAGGTTATACTACCGCAGCCAGCCAAAAGACAGACA
CGTCAATGGGCTGCATGGACACGTCAGGAGGAAGAAAGCTTTTTCTCTGCACTGCGACAAGTTGGGAAGAATTTTGAGAG
AATTACTAGTCGCGTGCAGAGTAAAAACAAGGATCAGGTCAGACACTATTATTATCGTCTTGTGAGGCGCATGAACAAGT
TGTTGGGTCCAGAACTTTGTCTGGATGCCAAAAATTCCAAGGAGACTAATGCTGCAATGCTGCGATGGTGGTCTTTACTG
GAAAAGTATAGCTGTAAAGCTTCAAAGCTTCATTTGAAACCACGGAGATTTAAAATATTTGTTGAAACTTTGGAGAGTCA
GCTGGTGAAAGACCGCAAAAAGAATGTAAAAAGGCGTCCTTCTCAAGGAGAAAGTAGCTCTACAGCTGCAGCATCTCCTT
CAAGTCATGGCAGAGTGTCAACTAATGATAGTCGGACAGTTAAAGTGGTTCTTCTTGACAACAAGGACGCACAAAAATTC
GGATCCGGTAAAGGTCCTTCTGTAAAACGCAATGTCACTATGGGTGTTAACAGGGGGAATGTAAAGGTAGACTCTTCTGT
GAAAAATGCTAGACATCGCCGAAGGACAGGTTCTTCATCAACAGCTGCATATAAAAGGTGGGAGAAGGCTGCAATTGCTG
GAGTTTCATTGGTAGCTGATGCTGCCGAGCATTTGGAGCGGACAACTATTGATAAAGATGTTGGACCGGTCCAGAATTCA
CAGGGTATAAATGGCTTTGAACATGTTGGCAAAGATGTGCATTCTTTGTCGACTTTATCTCAAATTTTTTTAAAGGAGAC
CAATTTACAGAGTTGTATGAAACTAAAGCTCCAACTGTTTCCTGTTGATGAAGGAACTAGAAGAGCCCTGGAAATGGATA
ATCATAACCCGTTCTTGGAACTCACTCTAAGTAATCGAAAAAAGATGTCATCCGTTATGGAACATCTCAATCGTAAATGG
GGAAGTTCAAGCATAGCAACTGGAGAACTGGTGCTTTTTCCTTTCCATGTACAGATGGAAAGCCTGGTGCGATCCCTGAG
ATGGACCAAAGATACCACACAAATTGCAGCAGATGTGCATAATCTGATTGGAAGACCTCCAGTTTTCCGTCTCAAATATG
GTTGGTTTCCAAATGCTGAGCTTGGAACGTCCCAAGAACCATTGTCATATACCGTTCCATTTGTGCAAAATACTAACACT
AACATAACAAACGAAAAGAATGCGGAAATACCAGCATCTTCCCATGGTTCACTTGTACGATTTTCTAAAGAACCACTACC
TTCTAACCCAAGAATGACTGTGACATCCTCCTCAAACAAGCTTGGTGAGTCAAACTTGCAAGCTAGCATGGGTCTGAATA
CCTACACTTCTGATCATGATGAAACTCTGCCTTCACATAGAAGGGAGAACTGGGATGCAACCACCGCGGAACAAGATGAC
ATGCATGATATGCAGGGAACCAAGGCCAGTGCTTTGTCAGCAGGGGATTGGGAAGATAGCTTTACCAACATAAGTGTTGG
AGATCTTCTCTTTGATGCACCTGATGATGAGCAAACAGACTGCGTTGACTCAGCTTTGCCCGGAAGCTCTCATTTTCTCC
ATCAGACGCCATTAAGCTGCGACTCGTTTGATGCAGCGATTGCTGCTCATATATATAAACATCAAAGCAAAGCTGATTCT
CAGATGGCTCTTCCACCTCAAGCATCTTCCATTTGGAATGCTGAAGATACATGTGATGCTTTTGCATTCAAGAAAAATGT
TGCTTTCCGTGACAAAGAGCAGCGGTCATCCAGCAATGTTGGTGCAGAGAATTCCCAGCGGATTGCTCAGTCATCATCCC
TGGTATTGGATGCAGGAATCAAGGATTTGCCTGGGAACATGGAATCGTTCACTGGCGAAACTGCTCATGAAGACCCAATG
GATGAGTGTCAATCCGATGCACAAGCTTTGGATGGTTCTGCAAAGGATCCTAATGGGCTCTCTGGCATATATTGGACAGA
TTCTTTAGGACCGCTAGAATTGGACGCACCTTCATGTAGATATCATAATGAAGACATAACTCTGAGTGATAGTTTTGGTG
GTTTGAACCGCCTCATAGTTGACAGCCTGGATGCATTTCAAAATTGCTCGTTTTTTGGGTTGGACAAGAAAGAGCCTGCA
TCATCTACTGCTAAAGCTGTAGAGACTTCAGATTTAAAAATCGGCGCTGAAGTCTAA

>CcMYB119 |Locus_ID=BC332_09126 |CDS_len=1542 |strand=+
ATGGTGCAAAAAAGGCCATTTGGTGAAGAGGACCTGTATGAGGTTTCATCTAAGCAACCAAGATGTGAACCCAGTTGTCA
GTTAGGTTCCGTTTTGGAACATTCTTCTCTGGAGGCTTATGCTTCAGGTGGTGATGAGGACAACTCTTCAAGAACCATCC
CTGATGCAAACAAGAAACCTGATAGTCACAATGTGGCTGAAGTTCTTTTTTCTTTTGATAAGGAAACTGAGATGAGTATT
CATGGATCTGCTTCAAACTCTTCCTGTCCCACTAGTAGCACCAGTGAGGAAGAGATCATGCCTGAGGCGCCTTTTCACAT
ATTAACATCTCCTGAATATTACTATTTTGATCATCCGTTCAGGGCTTCTGCTCATCACAGGGAGATACATTCTCCCCTTT
TAAGTAATCCTCAAAAGATGGTCCCTATTGGACCTGATTTTCAGGCAGAGCTACCAGAATGGGATGCATATAATAAGGAT
AACCCGTGCATAGATGGTATACATGAAACTCTGAGTCCTTCATCTCAAGCTGATGAAAATAAATTTGCTGGAAGGACTAT
CGTTCTGATGCCTAAAATGGAGCTACTTGCAGATAATGTTGAGAATATTGCAGAGAGAGGAGTTGAATGTTCCTGTGAAG
ATATAGGTTCCATAAGATGTGTTCGGCTACACAACATTGAAGCAAGGGAAAAACTAAAGCTAGCCCTTGGCGAGGAGACA
TTTGTTAGATTGGGTTTTTGTGACATGGGAGAGGTTGTGGCTGAAAAATGGAGTGAAGAGGAAGAGGAATTATTTCACGA
GGTTGTATTGTCCAATCCTGCATCGCTAGGCAAGAATTTCTGGAACCATCTTGCTATTGAGTTTCCTTCACGGAGTAGGG
AGGAGCTTGTCAGCTATTATTTTAATGTCTTTATTTTGCGAAAACGTGCTGAGCAGAACAGATTTGATCCACTGAATATA
GATAGTGATAATGATGAGTGGCATGAGACTGTTGATGATGCTGATGAGGGAGCCAAAATGACGGATGAAGATGAGGATTC
TGTGGTTGAATCCCCAGCATATTATAATTATCTCAGCTATAATATGGGCCATGAGGAGCACGATAGACCAGCATGGGAAT
ATTATAATCCTGTAAATTTTGGTAGCAGGAAGGTCTTTACTGATGTGCAGGAATCATGTCTGGGTAAGTTGTTTGACAGT
AACAGCAGTTGCAAACTTAGTATGCAGCCTCAAGACCAAGGACTACCAAATAAAGTGGTTGAGCAAGAAGTCCAAGGAGG
CTCCTGTATCACTGGTGCAGCTGGGGTTACTTCAGAATCGTCACTAGGGAAGACCAACAACAATAAACATTGGGCAAGTG
ATATTGCTGGTACGGGCATTGGCACCAACCATGACAGTCTGTTGGAGCCATCTAATCGCAAAGAATGGGATGTTGGATAC
TTGAGCTGTGCCAGAAACAAAGTTGATTTGTTACCAACACACACTATGATTGAAGAAGTCTTTGGTGATGGAGCTTGGAT
ACACAAGAGCCGAGATGTTTAG

>CcMYB120 |Locus_ID=BC332_11642 |CDS_len=1758 |strand=-
ATGATGGAGAAAAGGAAGTTCAAGAGAAGGGTCTTTGTTAGTGAACATGACATGTCCACTCTCTTACAAAGGTACACAGC
AATGACAATGTTGACACTGTTGCAAGAGGTGGCACAAGTTAAGGAATCGAAAATCGATTGGAATGAATTGGTGAAGAAGA

CTACTACTGGGATTACCAATGCTAGAGAGTACCAGATGGTGTGGCGCCATTTGGCTTATCGCCAAGTATTGCTTGATAAA
GTGGATGATGATGCTCAACCTCTGGATGATGATAGTGATCTAGAATCTGATTTGGAAGCTTTTCCTCCGGTAAGCAATGA
GACTTCATTAGAGGCTGCAGCCTGTGTGAAGGTATTAGTTGCTTCTCGGGCTCCATGTGATACAAATATGTCAGATGGAA
ATACAATTGAGGTTCCATTGACTATAAGCATGCCCAATAGGCAAACATCTGGAACTGCTGCAGAAAATTCACTCCATGGT
ATTTCAGCATATGGGACAAAACTTACAGTCCCGGTTACTGTGCAAATGCAGCCACTTCCTTCTGTCACAGCTGCTGAGGG
TTTGGACACCAGTGGACCAGGTACAGCCAGCTATAATTCTCGTCGAAGACGAAAACCTTGGTCTGCAGCTGAAGATAGGG
AGCTCATTGCTGCAGTACAAAAGTGTGGTGAAGGGAACTGGGCAAGTATATTGAAAGAGGACTTTAAGGGTGACAGAACA
GCTTCACAACTTTATCAGAGGTGGTCAATTATTAAAAAGCGGAACATGGCCGGAAATGCCTCACAACTGTCGGAGGCTCA
GCTTGCGGCTCGTCATGCAATGTCCGTGGCTTTTGGGGATAATTTAAGAGCAGCTTGTCCAACCAGCACTAATGTTGGGC
CAAATTCAAGTGGTGGACCTAGTAACTCTTCCCATTTTGCTGCTGCTGATATTGCATCAGGTGGTCCACAATCTAAGCAT
CGGCAAGATTTAGTACCTTCAAAACCTATAGTCCCAAAAAATCCATTAGCAGAGCCTGCTATTAACCCAGATCCAATGGC
CAAAGCTGCTGCTATGGCTGCAAGCTCCCCCATTGCTGTCCATTCAAGTGCTGCTGCCTCGCTGCAAAAGGCTGCACAAT
CTAAAAAAGGCATCCATATCATGCCTGGAGGAACTCCTGCAGTCAAATCATCAGTGCCAAGTAGCTTTAGTGGTTTGCCT
AGTAATGTACATTTCATTCGAACTGGCCTGGTGTCCCGTCCCGCAGGCCTCTCTAATGCCTCACAGTCTGGAACCCAGCA
ATTGCAGGCTCTAAGATCTGCATCACCTGCAGTTCAGCCTAGACCAGCGACTGTGCCATCCAGAACCAATACTGCATCTG
TGGTTCCCAGTGCCCCTACATCTTCTCCAGCAACCGCCGTGCTCGGAGTAAAATCTAAAGCCGCAGTTATGCAAGAAAAT
CAAACTGCTGTTCTGTCTAATGCAAGGAGTGAGAAAGCTGGAGTTATTCGAGCTGCTTCATTGGCGAATACCCCACAACA
GCAGGTCCAAAAAGGTCCAACTTTTGGTTCAGGCAACCGGTTGACAGTGAAGGTTGAAGGTCCAGAACTTGGAGGAGAGA
GTAAAGCTTCAAGAATTTGGATCCAAGAAAAACTAATCCCAAGTCAAGAGTCTCCCAGCAATAAGAATGAGAACACTAAA
ACAGAATTGGCAGCTGAAAACACTAGTGGTCAAGGTCCAGCACCTGTGACATGTAAGGTGGTGTTCAAAGATAGTTGA

>CcMYB121 |Locus_ID=BC332_19144 |CDS_len=402 |strand=-
ATGGCAGATTGTGATCGATCAAGCACATCAGATAATGCCTCAGTAGTATCAGCTGAATTTGAGCAAAAGTTATTGAGTTC
ACGTGAATCCACGATAGATCCACCCGCAGTGACGGCATTAGAGACCACCAACAAAGAAACCTCTGAACTTGAATTTTCAG
AAGATGAAGAAATTCTCATTGCTAAAATGTTTAATTTGGTCGGAGAGCGGTGGTCATTAATTGCTGGAAGAATCCCAGGA
AGAAGTGCTGATGAGATTGAAAAGTACTGGAAATTGAGAATATACTCCAAGAGCCAGGGCGAAAGTATGTCGGAGATACT
ACTCAAATCGAAAGATCAATCTCAAACCATTGCACTGAAAGAGGAATCTGGGCATTCAACGGAAAATGTTAAGGGGAAGT
AG

>CcMYB15 |Locus_ID=BC332_12046 |CDS_len=831 |strand=-
ATGCAGCTACCCCCTTCATTAGGATTTTCTTTTACTATTATACGGTGTTCTACATCCACTTCTTCATCTTCAGAATCATC
GTCATCTGAATCAACACCGCGTAATGATAACAAAGCAGAGAAGATAAAAGGACCATGGAGTGCTGAAGAAGATAAGATAT
TAACGAAACTTGTTGAACGTTACGGTGCACGTAACTGGTCTTTGATAAGTAAATATATTAAAGGTAGGTCTGGCAAATCT
TGCAGGCTTCGTTGGTGTAATCAGTTAAGTCCCAACGTTGAACATCGACCATTTTCTCCATCTGAGGATGAGACCATACT
AGCTGCTCATGCTAAATATGGCAATCGTTGGGCTACCATTGCACGTTTACTACCCGGTCGGACTGATAATGCAGTTAAAA
ATCACTGGAATTCAACGTTAAAAAGACGTTATCAACAATTGCTTCAGCAGCAAAATTCGGGTCAAATGGTTAACGGGTTG
GAATTCCGGTCCGGGTCAGGCTCCGGGTCGGGTTCTGGATCCGGGTCGGGTATGGAGTATATGAATGTTGATGAGAGTCC
AAAGGGGAATACTAACAATTATGCTACAACAATGACGACGATGGCTCAGTGTAATTATACAAGTGGGGTTTGGAGGAATA
ATAATAACTATGGTGGCGGTGAATGTGATGATCCAATGACGACTTTGTCCTTGGCACCACCGGGGATGGCCGGAGAAGAG
ATGCCGGAGAGGAAAACGGAGAGTTTTCCGGCGGGATTTTGGGATGTAATGAGAGATGTGATTGCGAAAGAAGTGAGGGA
ATATGTTGCTTCTACGGGGTTTCCTAATTAG

>CcMYB18 |Locus_ID=BC332_23339 |CDS_len=1086 |strand=-
ATGAGGGAGAGGCAACGGTGGCGATCTGAAGAGGATGCTTTATTGCGGGCATATGTTAGACAGTATGGACCAAAAGAATG
GCACCTTGTATCGCAGCGTATGAACACACCCCTCAACAGGGACGCTAAATCTTGTTTGGAAAGGTGGAAAAACTACCTGA
AACCAGGGATTAAAAAAGGATCACTCACTGAAGAGGAGCAGCATCTTGTTATCCAACTACAGGCCAAACACGGTAACAAA
TGGAAGAAAATAGCAGCTGAAGTACCAGGTCGAACTGCTAAAAGATTAGGAAAGTGGTGGGAAGTATTCAAAGAGAAGCA
ACAGAGAGAGCAAAAAGAAAACAATAAGGTTGTTGATCCAGTGGACGAGGGAAAATACGACCACATTCTTGAGACCTTTG
CAGAGAAGATTGTAAAAGAGCGAAGTGTTCCAGGTTTACTTATGGCTACTTCTAATGGAGGTTTCCTCCACACTGATGCA
CCAACTTCTACGCCACAGACTCTTCTTCCTCCGTGGCTTTCTAATTCCAGTGCTCCTTCAACTGTCCGATCGTCATCTCC
ATCTGTGACTTTAAGTCTCTCCCCATCAACAGTGCCACCTACTCCTACGCCCAGTATTCCGTGGCTACAGACAGATAGAG
GACCTGAAAATGCGCCCCTTATCTTGACCAGTTTTCCACATCATGGGGTTGCCCCTCCTTGTGGAGAAAATCCATTTATT
ACTGAACTTGCAGAATGCTGTAAGGAACTAGATGAAGGGCATCGTAGTTGGACTGCACATAAGAAGGAAGCAGCTTGGAG
GTTAAGGAGGGTGGAATTACAACTAGAATCAGAGAAAGCAAACAAAGTTAGGGAAAAGATGGAGGAAATCGAAGCAAAAA
TGAAAGCTTTGAGGGAAGAGCAGAAGGCAACTTTAGACAGGATTGACGCAGAATACAAGGACCAACTAGCAGGTTTGAGG
AGGGATGCAGAAGCAAAGGAGCAGAAATTGGCTGAGCAATGGGCCTCCAAACACCTACGCCTTGCTAAGTTTCTTGAGCA
GATGGGATGCCAATCAAGACTTGCAGAACCTAATGGTGGCCGCTGA

>CcMYB4 |Locus_ID=BC332_27825 |CDS_len=1431 |strand=-
ATGCAGAATGCTATGAAGAGAAGCGTTGAAGCTGCAAAGACTAAAGAAAGACATATTGTTTCTTGGTCACAAGAGGAGGA
TGATATACTAAGAGAGCAAATTCGAATCCATGGAACAGACAATTGGACGATCATCGCATCGAAGTTCAAGGATAAAACAA
CAAGGCAATGTCGAAGAAGATGGTTCACTTATTTGAACTCTGATTTCAAGAAAGGAGGGTGGTCACCTGAAGAAGATATA

CTTTTATGTGAGGCTCAAAAGATCTTTGGTAACAGATGGACTGAAATTGCAAAGGTGGTTTCAGGCAGGACCGATAACGC
TGTGAAGAATCGGTTCACTACACTGTGCAAAAAGAGAGCAAAGCATGAGGCTTTGGCTAAAGAAAACAGCAATTCATACA
TTAATCTGAATAACAAGAGGGTTATATTTCCAAGTGGCCTTAATATGGATAACATAACAGAAGCTACTGCTCCTATTAAA
AAGCTGAGGAGGAGTGACATTTCAGATGTCCTTCAAATTGGTAACGAGGGAGAAAAATCTATGGGTGATTGTGGAACTAC
AAATCCATTACTAAGACATCCATTTGCAGTACTAGCTCAAAACTTGCATAATGCTGGGGAAAACCTGGCATCTCATCAGC
GGACTACTGGAAGTGGTAGTGACAACAAAACTCAAGGAACATTCCTTAAAAAAGATGATCCAAAGGTACACGTTTTGATG
CAACAAGCAGAGCTGCTAAGCTCACTGGCTATGAAAGTGAACACAGAGAATACAGATCAAAGCCTTGAAAATGCGTGGAA
GATACTCCAAGATTTTCTTCAACAAACAAAGGATGGTGATATGCTCAAGTTCCAGCTCCCTGAGATGAATTTTGAACCTG
GTGATTATAAGAACCTGATAGCAGATTCAAGAAGTAGTAATGAAGGCACTCAACCATCTTGGAGGCAACCTGCTTTATCT
GAAGATTCTGCTGGCAGTTCTGAGTATAGTACGGGATCAACTCTGCTATCCCATGCACTGGCTGATAAAACAGAAGAAAG
TCAAGCTGAGGTGTGTGCACATCATCAGGATATTGAATCTGAATTACGAACGTCTCAGATGAGTGATCAAGCTGGGCTTC
ATGAATTAGAAAATGGAATCTCCTGCCGTGTATCCACCACTCGAGATACTTTGCCAGTTTGTGATGAAGAAAAAGTTAAT
AATGGACCTGCTACCGCTGAATGTGAATTTCTGAATACAGATTTCAGTTCTCCTCATCAAGTAACTCCACTGTTCAGATC
TTTAGCTGCAGAAATTCCCACTCCAAAGTTTTCAGAAAGTGAGAGACAGTTCCTATTGAAAACACTTGGAGTGGAGTCCA
CATCCCTGTATCCAGGCACTAATCTTTCACAACCTCCATCGTGCAAAAGAGCCCTCCTCCATAGTCTATAA

>CcMYB58 |Locus_ID=BC332_32563 |CDS_len=1068 |strand=+
ATGGGGAGGCCTCCATGTTGTAATGATAAGGTGCGTAATAGAAAATGTAATTGGATAGAAGATGATTCAAACACATCTAC
ATATGTCTCAAACAAGCATGGAATTGGTAATTGGGCAACCATGTCTAAGAAATCAGGATATGGGAGAAGTGGGAGGATTC
AGAAGCAGCGATGGAGTAATCGTCTGAAACCTGATCTCAAGCAGGACAACTTCACACCCCAAGAAGAGGAACTGATCATT
AAGCTTCATGCCACTATAGGTAGCAGGTGGTCTATAATAGCACAACAACTAGCTGGGAGAACAGACAATGATGTGAAAAA
CTTGTGGAACACTAAGCTGAAAAAGAAGCTATCTGCAATGGGGATTGATCCTGTTACTCACAAGCCTTTCTCCCAAATTC
TCACTGACTATGGAAACATAGGTGGCTTCCCGAAAGCCAGAACGCGTTTCGTGTCGCTTAACAGAGAGCTAAAGGGTGCA
TTTATGTCTAGACCAGAACAACTTCAACATTCTTTACAAAGTTTCCAAAACTTCCACAGCCTTTGTGTGAAACTGCCAAA
AACTGAAGCTTCAGAAGAATGTTTCTTCAGCAACAATCGAGATTCGAGCAATGTTAATCAGCCTCCGGTCGATCTGCTGT
CCGAGTTACAAGCCATAAAATATGTAACTGAGGCCTCAAATTACAACTCCCCAAAGGCAATATTCTCACATCATCCAAAT
CAGATGACTGATTGCTCTACTTCTTCACCTTTGTCCTCGTCCTCGTCATCATCAGCATCTCATTCATTAAACGAAAATCA
AGTCAACTGGTGTGATTATCTTCTTGACGATGCATTTATTCCATCGAATTTCAAAGATCAAGAAGACGAGTTAACCATTG
AAGAAAAGCTAGTGAGTTCAGGAGCTCAAGATGGGTCAAACAACGTGCCATCAACAAGGGATTTTCAAGCCTCGTTATCT
ACAAAGGGTACATCATCATCGTCATCATCATTTGTTGAAGCCATGCTAGAATGTGAAAACGAAATGTTCTTGAACTTCCC
TGGCCTCTCTGAGGATCCCTTTTACTAG

>CcMYB62 |Locus_ID=BC332_19189 |CDS_len=564 |strand=+
ATGAGATGTAGGAAGAGCTGCAGATTGAGATGGATGAATTACTTGAAGCCTGGCTTGAAGAAAGGGAATTACAGCCCTGA
AGAAGAACTCATTCTTAAATTACACAAGGAACTCGGAAACAAATGGTCAGCGATTGCTACAAGGTTACCAGGAAGATCGG
ACAACGATGTCAAAAACCACTGGGATGCTCATCATAAGAAACGGATAAGATTAACAAATGCCAATTCATCCACATCAACG
GAGAAATTTACTGAATCATCGTCGTCTGATTCTAAAAATGAACAATCTTGTAACAGTGACGAAGCTGACTATGATATGAA
AGAGGTGCCAATATCAAGTGATTTATCAAATAAGTTAAATGGAGTAACAGACTGGATTGAAGAAGAAGATAACCCCACCT
TATTAGGACAATGGAAAAACTTTCAAGTATCAATTCAGTGGAACAATCACTTCCAGATTCTCTTTGCTGGACAAGTCCTA
TTGCCAATTTTCAGACAAACTTTTGACACTAATTTTTGGTCCGAATCGCTACTCGACAATTTCTGGACACAACCTTTCTT
CTAA

>CcADA1 |Locus_ID=BC332_14478 |CDS_len=1365 |strand=+
CAGGACATCTTAGCTTTCCCACTTATCTGCCCGGACTGGAATGCGGATGAAGAGATGTTGCTTCTTGAGGGAATTGAAAT
GTATGGCATGGGTAATTGGGCAGAAGTAGGTGAACATGTCGGAACAAAGTCAAAAGAAGCGTGTGTCGACCATTTTAAGG
ACGCATACTTAGACTCACCTTACTTTCCTCTACCGGATATGACTCACGTCATGGGGAAAAATAGAAAGGAACTCCTTGCC
ATGGCCAAAGGGAATTTCACAGATAAGAAAGGAGTATCCTCACTTGGTGAAGTTGTTCCGAAAGATGAATCATTTTCTCC
GTCTCAAGTCAAAGTTGAAGACTCTCATAGAAAGGGTCCTTCAGGACGTTTATCTGCTGTATCCAATGCGGTCATTAACA
GCATGAATGCTTCAGGAATCACAGGCATTAAAAAACCATCCAGCAAGTCGCAAATCAAAAACCAAAATGAACCTGTAAAA
TTGGAAGGCAGAAATTTTGGAGGCAAGAAACCAAAATCGTTGAAAGAGGATGGATCCTCATTGATGAAACTAAGTGGATA
TATCCCCAAAAGGGAAGAATTTGATCCTGAATATGATAATGATGCGGAGCAACTATTGGCTGATATGGAATTCAAGGAAA
CTGAAACTGAACAGGAGCGCGAACTTAAGTTGCGTGTTCTGCGTATCTATTCCAAGAGGCTTGATGAAAGAAAGCGCCGC
AAGGATTTTATTCTAGAGAGGAATTTACTCCAGCCAAGTGAATTTGAGAAGAATTTGTCACCAGAAGAGAAAGATCTATG
CCGACGTTATGATGCCATTATGCGCTTCCTCTCGAAGGAGGAGCATGAAGAATTAATGAAGACTGTGGTCTCGGAACATA
GATATCTGAAAAGAATACAAGAACTCAAGGAATCGAGAGCTGCTGGTTGTCATTCATCTACTGAAGTTGATAGATACTTA
GAAAGGAAAAGGAAGAAAGAACTTGAAGATGGTGTTCCGAGAAAAGAAAGTTCTCAGATTGGCCCAATGAGCCAGGAGAG
CCTGAACATGCCTGCTTCTTCTGACTCACTTGGAACACATTCAAATAGAAAACCTTGTAGCCAGGCGAATTTGAGTTCCA
TCAACGATTCGGGGGTTGCAGCTCTTTCTGCAGGAGAACTGTTATCTGAACCTGAGAAACAACTATGCCGAGAAATCAGG
CTATCACCGCATCATTATCTTCGGATGCAAGAGGTTCTTACAATACAAATTTATAGTGGCAATATCACTAGAAAATCAGA
TGCTTATCCCTTGTTCCAAATAGAAGCAACTAAAGTAGATAGAGTTTATGATATGCTTTTGAAGAAAGGAGTTTCACCCT
TGTAA

```
>CcADA2 |Locus_ID=BC332_18760 |CDS_len=1686 |strand=-
ATGGGTCGTTCTCGGCCTGTTCGTCAATCCACCGATGATGATCCAACCCAGAGGTCTAAAAGAAAAAGGGCTGCACCAAA
TGTGGAGAATTTTGATACTGCAGCTGCTGGCCAAATGTTGACCGAAGGGAAAAAGGCTTTGTACCATTGCAATTATTGCA
ATAAAGACATATCTGGAAGGATTCGAATTAAATGTGCTGTGTGCTCTGACTTTGATCTTTGTGTGGAATGCTTTTCTGTC
GGAGCAGAAGTGCAGCCTCACAAAAGTAATCATCTATATAGGGTTATGGATAACCTGTCATTTCCTCTCATATGTGCTGA
CTGGAATGCAGATGAGGAAATGTTACTTCTAGAGGGGTTGGAAATGTACGGATTGGCAAACTGGGCTGAAGTTGCCGAAC
ATGTTGGAACAAAAAGTAAATTGCAGTGTATTGAACACTATAAGTCCATATATATTAGTTCTCCTTGTTTTCCACTTCCG
GACATGTCTCACGTTATGGGAAAGAGCAGAGAGGAACTTCTTGCCATGGCCAAGGAGCAAGGATATGCTGCTCCCGGGGA
AGTTAATGTTAAAGAAGAGTCTCCATTCTCTGCAAGAATCAAGATGGAAGATCAACGGGAAGAAAATTCAGCTGGCCTTG
CCTCAGTTGGAGGTTCTGCTTCTGGTAAATTAGCAGGCGCTGGAAAGAGGACATCTGCCGTGCTTAATAGTAAGGAGAAT
CATGATAGCATCAAATTGGAAGGTTGTCCTGCAGACAGGAGCGTTGGAGAGAAAAAGCCAAGGACATCGGTGGACGAGGG
GCCTTCTATGACAGAATTGAGTGGTTATAATTCTAAGAGAGAGGAGTTTGAAATTGAATACGATAATGATGCTGAGCAGA
TGCTGGCTGATATGGAATTTAAAGAGACAGATAGCAATGCTGAACGTGAACTGAAACTTCGTGTATTGCGTATATACTCT
AAAAGGCTTGATGAGAGGAAACGTAGGAAGGATTTTATTTTGGAAAGAAAACTACTTCATCCCGATCCTTTTGAGAAAGA
CCTCACTCCGGAGGAGAAGGACATATGCCGTTCCTACAGGGTATTCATGCGTTTTAGTACAAAAGGGGAGCATGAGGATT
TCCTTAGGAGCATAATCGAGGAGCACCGGATGGCAAAACGGATACAAAATCTTCAGGATGCCCGAGCTGCTGGTTGTCGA
ACTTCCGCTGAGGCAGAAAGATATATTGAACAAAAGAGAACACAGGAATCTGAAGAAAATATACGTAGACTAAAGGAGAA
CTCCCAGAATGGCCCAAGTGGCAAATATTTGCAAAGAGCAGGTCACTTTAAAGTGGAGCATGACAGCAGCCCCAGTGGAG
TTGTTAGAGGCCCTGAAATGCTGGATTCTTGTTGCAAGGACTTATCATCAACCACCGCACCACATGGTGTTGGAAGTGCT
TTAGACATTTGGGATGTCAGTGGCTTTTTAGGGGCTGAGTTACTCTCAGAAACTGAAAAAAAGCTTTGTGATGAGATCAG
AATCCTCCCGAGTCATTATCTGAACATGTTACAGACCATGTCTATGGGGATCTTAAATGGCAACATCACCAAGAGATCTG
ACGCGCATGGTCTATTCAATGTTGATCCGAATAAGGTGGACAAAGTGTATGAGATGCTTGTTAAAAAGGGCCTGGCTCAA
GCATAA

>CcARR1 |Locus_ID=BC332_14187 |CDS_len=1992 |strand=+
ATGAATCTTGTTGCTAGTTCTAGTGTTTCTTGGAAATCTAATAGTAATAATAGTGATAGAGTTGAAAAAAATGAGTTTCC
AGCAGGTTTAAGAGTACTTGTTGTTGATGATGATCCTACTTGTTTAAGGATCTTGGAGCAGATGCTTAGGAATTGTCATT
ATGAAGTCACCAAGTCCAATCGAGCGGAGATTGCCTTGTCAATGCTCCGCGAGAACAGAAATGGTTTTGACATTGTTATT
AGTGATGTCCACATGCCGGACATGGATGGTTTCAAACTTCTTGAGCATGTTGGTTTGGAAATGGACCTGCCTGTTATAAT
GATGTCTGCGGATGACAGTAAGGATGTCGTTATGAAAGGTGTTACTCATGGTGCATGTGATTATCTGATCAAACCGGTGC
GTATTGAGGCGTTGAAGAACATTTGGCAGCATGTGATTCGTAAAAAGAAGCACGAGTGGAAGGACAAAGATTTCGAACAA
TCGACAAACGTGGAAGATGGAGATCAACATCAAAAACCACCAGAAGACGTTGATTATTCATCTTCAGCTAATGAAGGCAA
CTGGAAAAGCTCAAAGAGAAGAAAGGAGGAGGAAGATGAAACTGAAGAAAGGGATGATACATCCACATTAAAGAAGCCAC
GTGTCGTTTGGTCGGTGGAGCTTCATCAACAGTTTGTACAAGCTGTCAACCAACTTGGAATCGACAAGGCTGTTCCCAAG
AAAATTCTTGAACTGATGAATGTTCCCGGACTAACAAGAGAAAACGTTGCCAGCCACCTTCAGAAATATCGGCTGTACCT
CAGGAGGTTGAGCGGTGTATCACAGCATCAGAATGGATTGAATGACTCGTTTATGGGACATCCGGAAGCAGCATACGGGA
CAATGACTTCTTTCAGTGGGCTTGAGCTTCAAGCTTTAGCTGCCACTGGTCAACTTCCCGCACAAAGTCTTGCTACCTTC
CAGGCGGCTGCATTAGGTAGGTCTGCGACAAAATCTGCCATATCTATGCCTCTCGTAGATCAAAGAAACCTTTTCAGCTT
TGAAAATCCCAAGTTGAGATATCCCGAGGGACAGCAACAGTTAAATAATAGCAATAAGCAAATTAACTTGCTTCATGGAA
TCCCAACAACCATGGAACCGAAGCAGCTAGCAGATCTGCACCAGTCGTCGCAGTCCTTTGTGGCTATGAATATGCAAGGG
AACGCCAGAATGCAACAAAATAATGCTCTACTAATGCATATGTCTCAACAACAACAAAAACAGCCGTCTAGGGGTCAAAT
GCTAAATGAATCCAGTAACGGTCAAGTTTCTCGGCCTCCGTTGTCCATGCCACAACCTTTGTCCTCAAACGGGATACCTG
CTGCAATCTTATCACGAAATGGCATCGTTGACAACGTACGAGGTCCGGTATACAATCCAGTCTCCCAAGCATCTTCAATG
GTAGATTTCTCGCTAAATCAAACTACGGAGTTGCAAAACAGTTTTCCTCTTGTGAGTAGCAATTCCGGGATGTCAACTCT
GACATCTAAAAGATTGCTTCCGGAAGAGGTTAACTCTGATATTAAAGGATCCAGAGGATTCCCTGTCGGTTACGATATTT
TTGAAGAACTGCATCAGCAAAAATCGCAGGATTGGGGTATACAAAATGTCGGATCAGCCTTCGGCGCCTCTGATCATTCA
GGTATACCAGGAACTCTAGATGTCTCACCGTCCATGTTAGTTCAACAAGGTATTTCGTCAATGAAGAAGAATGGACAAAA
TGGGAATTCGCCTATGGGCGGACCGCAACTTAACTTATTTTCCGGTGGAAATTTCCTTACTGTTAAAGCTGAACAACTTC
CTGATACGAGCTATCAAAGTAACTTTTCTCTGGAGCAATTTGGACAAGATGATCTCATGAGTGCCATTTTGAAGCAGCAA
GAAAGCGTCGGAGAAGTTGAAACTGAGTTTGGCTTTGATGGATATCCACCACTGGACAATCTTCCTGTGTAA

>CcARR10 |Locus_ID=BC332_02543 |CDS_len=2067 |strand=-
ATGGAAAACGACTCAGCAGCAGTTGCTACAACGACGACGAAGAAGACAACGACGACATGGAGTACATGGGAGGAACTTTT
GTTAGCCTTTGCTGTGAAAAGGCATGGTCTTAAGAACTGGGAATCTGTAGCTATGGAACTCCAATCCCGTATCTCTTTAC
CCGTTTTATTGACCGCTCAAATTTGTAAGGATAAATACCATGACCTCCGCCGCCGCTTCATGAATAGCTATAACAATAAC
AGTTATGATGAGAAGATTAACGACAGTTTTGATGAAGGAGTTGAGCTGGATGTTGATGGGAATGGAGATGTAACGATTCC
TTGGATTGAGGAGCTGAGACAACTCCGTGTTGCTGAGCTTAAAAAAGAGGTTCAACGCTACGATCTTTCGATCCAGTCGT
TGCAGTTGAAAGTGAAAACAATGGAGGAAGAGAGGGAACGGAGTTTGAAGGAGAAGAGTTTAGACGATGGAAAGAGACCA
GATCTGGAGGATGTAAAGGAAGAGAGATCGCAAAACGACAAAAACAGCGGTGCCGATGTTAAACCGGAGGATTCGACCGG
AAAAGCGGTGTCGAGCGAGGAATCAGACCGGGAAAACCGGTCCTTCAACGAGTCTAATTCAACGGAGAACAGAGAAACCG
GTGTTAAAAACGAACCGGAACCGGTCGAGATCGGTGAAGATAAACCGGTTCAGGAAGTTAAACCGGTTAGTGAAGCGGAT
TCATATAACGATAGCAGTTCAGATAGACAGGAGAAGAAAGTCCGTCGTGACTCAGGCGATTTACGCGATACAGTCAGCGA
```

GTCGAAGGAGGAGACAAAGGAAAACAGTGACGTGCAGAGCACGGCGACTTTAACGAAGAGAAAACGGCAGTGCAGCGGCG
GCGATGGTGGTAGAAACGGCGGAGATGCGGCGGAGATGGCTTCTCCGGCCGTTGGAATTAAAGGGGAGGTAACGGCGAAA
TCAGAGCCGTTAATTGAGTTTTTAGATATTATCAGGTCGCATAAGCGCGGCTCTATGTTCAAACGCCGGCTAGACAGCCA
GAAAGCGGACAAGTACAAGAGTATAATCCGACAACATATGGATCTTGAAACGGTTCAAGCTCGGATTGACGACGGATCCT
ATTGCTCTTGCCCCGCCAAATTCTACTTGGACCTCCTACTCGTCTTCAACAATGCTATCGTCTACTTCCCCAAGTCTTCC
TCAGAATCAACTGCAGCTAATGAACTCCGTAGCATTGTAATTGAAGAATTAAAAAAGACCAGAACCCAATCAAAGGATCA
ATCACCCGGACCTAGACTCGCACCATTAAAGATCCAACTCAAGCCCGAATTGGAGATATCCGATTCTTTGCTTGCAAAAC
ACAAATCTACCGTTCCTATTGTAGTTTGTCGCAAAAGGAGCTCCATTTCAGCTAAAGCAGCAGGTTCTGGTAATAACAAA
CCGGAAAAACATGGTGATAGTAAACCTCCTTTGAATCCAAAGCCACCGATAAAGTCTTCTTCGAATGAAGAAGAGAGTTC
AATCAAGCTTGGGATGAAAGAAAAGCCAGTGACTGGGGCAAGAAGTATGAGGAGAAGCAGCAAAGGTCGTTCAAATAACA
ATGCTTCACCATCCAACAACAACAACAACCAGAACACAAATCCCAAGCAAACCAATAGCGGTGGGGACAAAAAGGAAGAC
GCGAAACCAGAGAAGAAGAAAGAAGAATCTAAAAAACGAGGAGCTGCAGCGGACTTCTTGAAGAGAATAAAGAAGACTTC
TCCTACGAAAGGAACATTAATTGAGGCACTAAAAAATGATCCGACGGAGGATGTTGTTAGTGTTAAAGTTAGTAATAAAA
AAGAGCAGCAAAAGAAAAAAGTTGATGATCGGAGAGAAGTTCCAGTAAGGCGTAGTGGTGGAGGTGGTGGCAGCGCAATC
AAGGAAGAAGGTAGTCCGTCTAAAAGGAGCGTAGGTCGACCACCAAAGAGAGGTGGTAGAGAAGTAGTAGTGCAGGAAAA
GCGTGGAAGGGAAAATAGTGAAAAGGATGATTCCTCTAAACGGCCAAAGAAACGATCAAGGAGGTAA

>CcARR11 |Locus_ID=BC332_21691 |CDS_len=1953 |strand=-
ATGGCTTCCAAGAGCAGTGTCCGTCTTATTTCATATTCAGAAGAGATTATAAATGGTGAACCAATCTATATTTCCTCGAA
CTGCTGCCCTATAAAGCCAACAAATATGAGCCTGCTGGGCTTGCTTTTCACTCTGCTGCTCTTAGACTGCTTGGGCATG
TTGAGAAAGAAGACCCTGAGGATGGTAAAGAAGATGTTCCTAATGACAAGGAACAGCCATTTATGTATTCGTCAGAATCA
TACAGCTCTAAGGGTAAAAAGAAATCTTCTACTGGGGAAAAGATACAAGATCATTATGCTTTGTTAGGGTTGAGTCACCT
AAGGTATCTTGCTTCTGAGGATCAGATAAGGAAGAGCTATCGTGATGCTGCATTAAGGCATCATCCTGACAAGCTAGCTT
CTCTTCTTCTAGCCGAGGAAACTGAAGCTGCAAAACAAGCTAAGAAGGAGGAAATAGAAAACCACTTCAAAGCTATTCAG
GAAGCATATGAGGTCCTTATTGACCCTGTTAGAAGAAGGATTTATGATTCCACAGATGAATTTGATGATGAAATCCCGAC
TGAATGTGCTCCACAAGATTTTTTTAAGGTCTTTGGACCTGCATTTTTAAGGAATGGTCGTTGGTCTGTCACCCAACCTC
TTCCTTCATTAGGTGATGAGAATACTCCAATTAAAGAAGTGGATAGCTTTTATAATTTCTGGTACAGCTTCAAAAGCTGG
AGAGAGTTCCCACATGCTGATGAGTTTGACCTTGAACAAGCTGAGTCTCGTGATCACAAGAGGTGGATGGAAAGGCAGAA
TGCAAAACTGTCAGAGAAGGCCAGAAAGGAAGAATCTGCTAGGATTCGTACACTTGTTGACAATGCGTATAGAAGAGACC
CTAGAATCTTGGGAAGAAAAGAGGCGGAGAAAGCAGAAAAGCAGAGAAAGAAGGAAGCGAAAATACTGGCGAAGAAATTA
CAAGAGGAAGAAGCAGCTAGGATAGTGGAAGAGGAAAAGCGTAAGAAAGAGGAGGAAGAAAAAAAAGCTGCCGAGGTTGC
TTCACAGCAGAAGAAGTTGAAGGAGAAGGAAAAGAAATTATTGCGAAAAGAGCGGAGTCGTTTGAGAACCCTCGCAGCTC
CTGTTTTGTCCCAGCATTTGCTTGGTCTGACTGATGATGATGTAGAGGGTGTATGTATGTCACTTGACATTGAGCAGATG
AGGAACTTATGTGATAAACTGGATGGAAAGGGTGAGCTTGTGAAAGCTGAACTTCTCAGGGAGGCACTTGGACAGAAACA
CAACTCGAAATATGAGAATAAAGATGAAAATAGTAAGTCGCAGCAAAATGGTTCTCTGGAGGATAAAATACAAGTTTCTT
TGATGAGCAGTGAGAAAAGGGAGAAACCTTGGAGCAAAGAAGAAGTTGATCTTCTGAGAAAGGGGGTGCTGAAATATCCT
AAAGGAACCTCGCGAAGATGGGAAGTTATTTCCGAATATATTGGTACTGCAAGGACAGTTGAAGAGATCCTGAAGGCTAC
GAAAACGGTTCTGCTCCAGAAGCCTGACTCTGCCAAAGCCTTTGACTCCTTCCTCGAGAAAAGAAAGCCAGCACCGACTA
TTGCCTCTCCTCTTTCCACGAGGGCTGAAGTAGAGGGGGTAGATAACAGTAGCAAACCTGAAAGTGGAAGTGCTAAATTA
GCCAATTCTCAAGAGTCCCTTAGTCAAAACACAAACAGCCAAAAAACTGAGGATGCGCCTGCAGCAAACGGGGTCTCCTC
GAGTTCAGATTCAGACATATGGTCTGCTGTTCAAGAAAAAGCTTTAGTTCAAGCTCTGAAAACCTTCCCAAAGGAAACCA
GCCAGCGGTGGGAACGAGTTGCAACTGCAGTCCCCGGGAAAACAGTGAACCAGTGTAAAAAGAAGTTTGCATTACTCAAG
GAGAACTTCAGGAACAAGAAAAGTGCAGTGTGA

>CcARR12 |Locus_ID=BC332_02846 |CDS_len=3426 |strand=+
ATGGCTCCAGCAAAAAAGTCCAAAAGCATAAACAAGCGATTATCTCCTACAACTGAAATCTCTCCCAGTAAAGATGGCAA
TGGGGACAACGGAAAGAAAAGCTTGCAACGGAAGCGGAAATTGTCTGACATGCTTGGTCCTCAGTGGAGTGAGGAAGACC
TGACACGCTTCTATCAAGCATACCGCAAGTATGGTAAAGATTGGAAAAAGGTTGCATCTACAGTGAAATTCCGAACTGCA
GAAATGGTGGAGGCTCTTTACACAATGAATAGGGCTTATTTATCTCTTCCAGAGGGGACTGCGTCTGTGGCTGGGCTGAT
TGCCATGATGACTGATCACTACTGCAACTTGGCAGCAAGCGACAGTGAGCAAGAGAGTAATGAGGACGCTGGAACGTCTC
GAAAACCTCAGAAACGTGCATGGGGTAAAGTTCATCCTAATATATCTAAAACATCTGAGATGACATCTTCAGCATTAGCT
GCTAGCCATGGTTGTTTAACTTTGTTGAAGAAGAAGCGTTCAGGAGGAAGCCGTCCTCGAGCTGTTGGAAAACGAACCCC
ACGATTTCCTGTTTCTTTTTCTTATGAAAATCCCAAGGGTGAGAAGTATTTTTCTCCTAGTGGACAGAGTTTGAAAGTGC
AGCCACATGACACCGATGATGATGTGAAGATAGCGTTAGTTTTAACAGAAGCTTCACAAAGAGGTGGTTCTCCTCAGTTC
TCTAAGACACCAAGCCGTTGGACGGATGGTGCTATGTTCTCACCTATTGGGACTGCTGAAAGAAAGCGTGTTAAAGTGGA
TATGGGCAACGCCAAGCTTCTTAGTAATGAAGTGGATGAAGAAGAGGGAAGCATGGAAGCAGACACTGGTGAGCTGATGC
GGTATAAGAATGATTTTGAGGAAGCTGAAACCGTTGGTCGAACAACACAGAAGCAAAAAAGAGCATATGGTAAAAAGTTG
GAAGTTGATGATAGTGGTGAAAATCATTTTGATGATATCAAAGAGGCATGTAGTGGTACAGAAGAAGGTCAAAGATTGGG
TGCGGCGAGGGGTAAACTTGAAATGGAGGCCACAGATGAAAAGAATGCAAGGGCCTCGTTACAAGGCCCTAGAAAGAGGA
GCAAAAAGGTTCTTTTCGGGCGAGATGAAAGCTCTTCTTTTGATGCCTTACAAACTTTGGCTGATTTGTCTCTAATGATG
CCAGCAGCTGAAAATGAAGATGAGTCAATGATGCAGGTAAAGGATGAAATTGATGACCATTTCGATGAATCTGGCTCCTT
GGAAGCCGTTCCAGCACACAAACAAAGAGACAAACGTGGATCGGCGGGGGTTAAATCGAGATGGAGTCAATCTTTATCAA

AGTTTGAAGTTACTTCCAGTAGGACATCAAAGCATGGGAAAGTTACATCTTCTGATGTTAATGCTGTTCCTGAAACAAAG
CAGGCAAGGAGGGCACAGAAAGCAATGTCATCTAAGGCTCGCAAAGCTGAAGGTCATGTTGACTATGATGTTACTGAATC
TCAGGAAGCTGCGGTAAAAGACGCATCAAAAAAGTCCACCAGTAAGGGTAAAAGATCCTATCAGTGTGTGTCGCCTAAAT
TAATTAAAGATCAGGAGCATTCATCATGTGCAGATCCAAGAACAGAACGAAGTGATTCAGCTCAATCAACTGCACAAATC
CCTGTCGAAAACCAGGTGAACTTGCCTACTAAAGTTAGAAGCCGGCGTAAGATGGACCTGAAAAAACCTCAAAAACAAAA
AGATTTGAAGATTCCTGATAAAAGTTTGGATGATACTAGTGCATCCTTCACTGCCCTTCGTGACAAAGCATTCAGTCTTA
AGAAAAAGATTTCCAGTTGCCTTTCCAACCATCAGGTACGAAGATGGTGCATGTATGAGTGGTTCTACAGCGCAATTGAC
TACCCTTGGTTTGCTAAAAGGGAATTTGTGGAGTACCTGTATCATGTTGGATTAGGGCATGTTCCAAGGTTAACTCGTGT
TGAATGGGGCGTCATAAGAAGTTCTCTTGGAAAACCACGACGATTCTCTGAGCAATTCTTGAATGAAGAAAAGGAGAAGC
TTAATCAATACCGGGAATCTGTTAGATCACATTATACTGAACTTCGTGAAGGTACCAGGGAAGGGTTACCCACAGATCTT
GCAAGGCCTTTGTCGGTTGGGCAACTTGTGATTGCGATCCATCCAAAAACAAGAGAGATACATGATGGAAATGTGTTAAC
AGTTGATCACTCTAGATGCCGTGTTCAGTTTGACCGACCAGAGCTTGGGGTTGAATTTGTCATGGACATTGATTGCATGC
CTCTAAATCCATTTGAAAACATGCCCACGCTACTTACAAGGCATGCAGATGCTGTTGATAAGTTTTTCGAGAGCTTCAAT
GAGCTCAAGATGAATGCACGAGCAAATGAGTTTATGAAATTTCCGGCTGGTGACAGCACGGAGAATGGAGATGTCTCCTC
TCATTTCTCCCCACCAAGTCCTCCCGTCAGTAATCTCCTAAAGCAGACGAAGGTGGCTTCAGCAGAAGCTGATATGCAAT
CTAAATCTGGAGTCATGGAAACCACCGTATATCAACAGATAGCATATTCCAAGCCTTCTACTGTCGCTCAGATTCAAGCA
AAGGAAGCTGATGTACAAGCTCTTGCAGAGTTGACCCGTGCACTAGACAAAAAGGATGCAGTGGTTTTGGAGTTGAGTCT
CATGAATGATGATGTTTTGGAAAACCAAAAGAGTGATGACTGTTTTCTCAAGGACTCGGAGCCTTTCAAGAAGCAATATG
CTGCCGTGCTAATACAGTTAAATGAAGTCAATGAGCAGGTTTCTTCTGCTTTATATCGTTTGAGACAGCGGAATACCTAT
CATGGAAGCATTCCACTTGCATGGCAAAGGCCAGTTCCAAATTTTGCTGATCCCAGTATTTTGAGCACTTTTGATCGTTG
TATGAGTCAACCGCAGGAATCAGGGTTCCTTGTCAACGAGATAATAGAAAGCTCAAAAATCAAATCTTGGACTATGGTTG
ATGCAGCGGTGCAGGCGATGCTTTCATATACTGGTAGAGACAACACCACCGAGAAGATTGAGGAGGCTATTGATTATGTG
AATGATCGGATTCCACTAGATGATTCTTGCATGCCAATTCCACCTGATCTTAAGTCAAAGAATGTGTCAGATAGAAGAAA
TGAAGCAGAAATTCCTTCGGAACTTATCACCAAATGCGTTGCTACTTTGCTTATGATTCAGAAATGTACAGAACGACAGT
TTCCACCAGCTGATATGGCAAAAGTACTGGATTCTGCTGTAGCAAGTTTACAGCCCTGTTGCTCACAAAACTTTCCAGTC
TATGCTGAAATACAGAAGTGCATGGGAATCATCAGAAACCAAATTATGGCATTAATACCGACGTAG

>CcARR13 |Locus_ID=BC332_24051 |CDS_len=5760 |strand=-
ATGCATGGATGTGGTGCGGAATCTGATCCTATAGTCGATTCCGAGGCTGATTCCATGGGAGTTGGTATTGGTAACAAAAC
CTCTCCGCAGAGATCAACAATCGAGGAGGTTCAAAATGAGCTTAGGCAGGGTTATGGTTTCCTAGAGATAAAGAGAAGAG
AATTAGAATTTCTTGAGAAAGGTGGTGATCCACTGGATTTCATATTAGGGAATGCTACTTCAGTTAGTGTTCAATCCACT
TCTCTTACAGATCAACATCAAGATCAGCTTGTGACCAGTGAAGCAAAAGGTAGTTTTGCAATTACTGCTTCACCTCATGG
AGACTCAGTTGAAAGTAGTGGTCGACTTGGGGCTCCTCAACTTTGTGAACCCAACAGTGCTGACAATCTCATGCTATTTG
ATGGTGAAAATGAATTCATTGAAGCTGAAAGGTCCTGTAGATACCCCAGCAGGAGCAACATTACTCCATCAGAGCAGTCA
TCTAAGTTGGATAGAAGTCGAAATGCAAAAGAATTGGGCGATTCTGCTGCTTTTGGCGTCCCTAAAAAATCTTACAAGCG
AAGATACAGGCCCCGCCCTAATGGGGATGGCACTAGATCAAGTTCCACTGATATAATTCTAGGTCCTGGTGGTCATGGCA
CATCTTTGCCCTCACAACATTTTTCTAAAGATGTAAAAGCACAGGTTTCTGATGGAGAAAATCCAAAAGACCAGAGCTCT
TCTTTGAGCATTGCACGGCCTTCCATTCCAAATGGTTTTGTGCCTGTGAAAACTCCGCCATCTGATAATCTGCTAGATAC
TGAAATACATGGCTTTAAAGTTGCAGAGGCAACTACTTACTTGAAGAATGAGGATCTGGCATATAGCATCCCTGAAGCTA
GTGCTTCCAGATGTTTGCTGGATAACCAACATGATCAGAACTCACTCGCTGATATCGAGGATGTGTCTATTCAGGAAAGT
CCTGAAAGGCCTCAATCATCACTGGGGAAAGAAGGGGTAGGTTCTGCTGGACAGGAAGGTCCGTTGTGTACAACTGCAGT
AGGGCTTGAGAATCAAGCTAGTTCTAGCCATCATATAAATGGGAGTTGTGGGAAGAGTGACCAGAAAAGCATGCCAAATG
ATGCTCAAAGCAGTGGTGCAGCATTGGGCACAAAAGGTTTAGATTCAGAATCTTCTTGCAGTCGGGCCCGTAGTCTGAAT
CGAAATACCAATAATGCGATGTCTAACAATCCAACGAACTTAGACTCCAATGGTGACTTGAAGGAACAACTGTCGGTGCC
AGAGGCAACGCAAGTTATAGAAAGCAATCTTAAAGAACAGAAAGAGGTGAAAACTGATGACAGTTGTGGTTTCACTAATA
AGGGTGAGGTAAAAGATAAAATTACCACCCAAGTGGAAGCAGTTGAGCCATCTCCATCAGAAACTGAGAGGAAACCCACT
ACGAACATAAGTGATAGTTCCAACTCCCAAAAATTATATTCTTCTATTATTGGGCGTCAAGGTTCAATCGAGTGCAGAAT
ACCTGTGCCATCCCAGCATGTATCACCACATGGAGTCTTAACTCCTTTCCCTGAGGCACAAGCACCTGCGATCAATGTTA
AACTTGCCACCAGGGGTGATGAAGACTCAATCTTGAAAGAGGCACAGATTATAGAGGCGAAACGTAAACGAATTAACGAG
TTATCTGCTGTGGCCTTTCCACTGAAGAATCATCGGAAGTCTCACTGGGATTATGTACTTGAGGAAATGGTTTGGCTCGC
AAATGATTTTGCGCAGGAACGCCTTTGGAGAATGACTGCAGCGGCTCAATTGTGCCGTCAAGTTGCTTTTACCGCACGGT
TACGATTCCAAGAACAAAATAGTAGTTGCAAGCTAAAAAAGGTAGCTCATATTATGGCCAAAGCCGTTATGGGTTTCTGG
CAGTCGATCGAGGGGAAAAACGAACAACTGGAGCTACCAGTTACTAGAAAGGATCATGCTATTGCTATCAGGGAATATGC
CATGAGATTTCTGAAATACAATGACTCTGATGTTCGACAAAGCCTCGCTGAAGCTCCGGTGACTCCAGAGAGGGTGTCTG
GTGGTGGAATTGTGGATGCGCCTCGGGAAGATCATTTGGGAGAAGAGAACCTCTTCTATGCTGTCTCATTTGGAGCAATG
GATGCCTACCGGAAGTCTATAGAATCTCATGCGCTGCTCTGTGTGAAAATTGGCGGGAGCATGCATGAAGAGGTGGAGAC
ATCGGCATGCGATACAATTCCAGAGTTTGGAGCTCAAGACTATGCATTTGAAGAGGATGAAGGAGAGTCAAGTCCATATG
ATACGTCAGTTGCCATTGAAGGTAACAAATCATCAAAATTTTCCCAGAAGCAAAGGAAGATCCACATAAAGGCGTATAGT
GGAAGACCATATGATGTTCGCACTGATGTACCATTTACACGTGTGGAATACAAATTTGGTACTCAGCAATCTATGCAACT
GGGAAAAAGGCCATCTAGCAATCTTAATGCGTCTATTCCGACAAAGCGTATGCGTTCTGCTTCTAGGCAAAGAGTTGTCA
GTCCTTACAGTGCAACAACATCTGGATGTGCTCAGTTGCCAGTCAAAACTGATGCTTCAAGTGGTGATACCAGTTCATTT
CAGGATGACCAGAGTACGTTGCATGGGGGATCATTTATGCCAAATAGTTTGGAAGTTGAGTCTGTGGGAAATTTCAAGAA

GCATTTACCATTTGATTCCGCTGAAGTTTCAAAACCTAAAAAGAAGAAAAAAGTGAAGATTCTGGGTTCCGCATATGAAC
AACGATGGCAGGTTGATTCTAATTTTCAAAACGAGCAGAGGGATCCCTCCAGAAAGAGATTGGAGAGCCATCAACTTGAT
TCTAATGGTCATAATGGTTTATTTGGTCAACATGTTGCAAAGAAGCCAAAGATGATGAGACAATCAATTGAAAATTCTTT
TGAAAATGTTGGCTCTGTTGGTGGATTTGTCCCATCTCCAGCGGCTTCCCAGATGAGTAACATGTCCAACCCAAACCAAA
TTATGAAAATGCTTAGTGGTAGGGATCAGGGTAGGAGAGCCAAAACTTTTAAGATGTCTGCAGGACAAGCAAGTTCAGGA
AGTCCATGGTCACTATTCGAGGACCAGGCACTCGTTGTCCTGGTGCATGATATGGGTCCAAATTGGGAGCTTGTAAGTGA
TGCTTTCAACAGTACTTTACAATTCAAGTGTATCTACCGCAAGCCAAAGGACTGCAAAGAACGACATAAAATGCTTATGG
ACAGAAGTGGTGATGGTAATGATAGTGCTGATGATTCAGGATCATCTCAACCCTATCCTTCAACGTTACCTGGCATTCCC
AAGGGAAGTGCCAGGCAACTGTTTCAGCGCTTACAGGGGCCAATGGAAGAGGATACACTTAGATCCCATTTTGAGAAAAT
GATCTTGATCGGGCAGAAATATCTTTCACGAAAGAATCAGGGTTATAAATTTGATTCGAGACAAATCCAGCAACCACATG
ATTCTCACACACATGCTCTTTCCCAACTTTATCCAAATAATCTGAACGGAGGCCCTATTCTTACACCTTTGGACCTATGG
GATGCACCCTCGCCTAGTCCAGACTATCCTTCTGTTGGATGCCAAGGTCCACGCCCAAGCGGATTATCTGTTTCAAGTCA
TGCATTGAATTCAGTGCTCCCGGTATCTGGTGCAAACTCCACAGTTCAAGCTTCCTCCAGTATGATTGGTGAAAGCAACC
TTCCATCATCAAGTCCCCTCAATGCATCTGTTAGGGATGCTAGATATGTTGTTTCTAGACCTGCATCCCTTCCAGTTGAT
GATCAGCGATTGCAGCACTACAATCAAATGAGAAATATGCAATCCAATATGTCTGCTCCTGGAGTTCTTGCAGCCACTAA
TCGTGCTGGTGTTCGTACGCTTTCCAGTGGCAACAGCACATGCATAATGGGTGGGATTAATAGAGGTATTCCGATGGCAA
GGCCTGGTTTTCAGGGAGTTGCATCACCGTCTATGTTGAATTCTGGAAGCATGCTTTCCTCTGGGATGGTAGCATTGTCA
AACACTCTGAATATTCATTCTGGAGTGAGATCTAATCAGGTGAATTCAATGATGAGACCTCGTGATGGTTCGCTCATGAC
ACAGCCACCACAGAATCAGGAAGCTCAGAGGCAAATGATGGTTCCTGAGCATCAGCTGCAGGCTTCTCAGGGAAACAGCC
AAGTGGTTCCTCCATTTGGTGGGTTAAGTTCCTCTTTACCCAACCAGTCTGCCTCTCCCGTCACACCATATCCACTTCAT
CTTCAGCAATCACATCCTATGTCTTCACAGCAGCCACTTATGCTTAGCCCTCATCACCCTCATCTTCAAGGGGCTAATCA
TGCCACCAACTCACAGCAACAAGCTTATGCGTTTCGATTAGCTAAAGAGAGGCACCTGCAACACCGACTGTTACAACAGC
AACATTTTTCACATTCACAGCCCCAACATCCTATTTCATCTTCTTTGCAAAATAGTCCCAAGACCACATCACAATCTTCT
GCTCCACCAGTATCAGTGACATCCCCCACCTCAGTGACCCCAATATCTCGACCTCATGCATTGCCAGCTCATGGGCATGC
ATGGACTGCTCAAACTGCGGGCAGCAGTCTAACTACTCAGATGAGTAAGCAAAGACCACGTCAGACAGGGGGACAGCAGC
TTCAACCAGCAGGCAAGCACCAGCCTCCACAGCGGCAGCAGTCACAATCACAACAAGAAGCTAAATTATTGAATGGAGTC
GGCAGAGGGAACATGATGATGCACCAGAACTTGCAGATAGATCCCTCTCTAATGAATGGGTTTTCTAACAATAAGGCGAA
TCAATCTGCTTGGAAAGGTGTGCATGCTACTAGCTTGATGCAAGGCCATGGACTATATTCTGGATCAGCAAACAGCCCTG
CCCATCTTGTAAAACAAGCTATGGAACCTCATTCTTCAAGTCCACAGCAGCCCGAGTCTAAAAGTTACTCTGGCCGACTA
CCTCTATCTACTAAGCATCTTCAGCAGGAGATGCCTTGTAATCCGGATAATAGCAATCAAAGTCCTGCTTCTTTGGATGC
CTCTGATACTAATTCATCTCAGCTATCTGTTCCATCCTCAGTCGTGGGTTCTTCCAACCACCACGAATTAGTGCATCAAC
AGGCACAGTGGCAGCCACAGCCAAAATTGTTGAATCAAAAGGAAGCAACTGTACAGCGAGTTTTGCAGCAGAACCATGTA
GTAAATTCAAATCCATCAAAGAGGTTGCAAGCTGATGAATTTCAAGCTGAACAACGATCTACGTGCAAAACTTCTAAGAT
AGGTGCAATCACCTCAATGCCTCAGGATTGCAACAATCCAACTACTGTCTCAGGTGTTTCACCTTTGAGTGCTAATCAGT
GGAAGGGTGCAGAACCATTATTTGATTCAATTGGGGCTCCCCCAACAAGTTCTGCTGGCAGTAAGCCAGTTCCTCAAGTC
AGCCGAGGGGTAAGCCAAAGACAACCTTCAGGCAACTCATCTCCAACAGGGCCTGATTTTAGTGTCAACTGGCAGCAAAA
ATCCTCCCAGTTGCAGCCTCCTTCCTCAGTGACCCAGCCACAGTTGCAGCAGCAACAGCAGCAGCTGTCACCATCGCAAC
ATTCACAACAACAGGGTCAGGTTCTGCAAGCAGGGAATAGCAGTTTGTTTGCTAGGCCCAATGACTGTAGACTGGATTGA

>CcARR2 |Locus_ID=BC332_02094 |CDS_len=2013 |strand=+
ATGAATATTGGTAGTGGGTCATTGGTTACTGTTCCAAGTTCTACCGGTTCTTGGAAGTCCGGTGATGTAGTTTCCGATCA
GTTTCCGGTGGGGCTCCGGGTACTTGTAGTTGATGATGATCCGACCTGTTTAAGGATCTTGGAGAAGATGCTCAGGAAGT
GTCACTATGAAGTTACCAAGTGTAATAGGGCTGAGATTGCACTATCATTTCTCCGGGAAAACAAGAATGGGTTTGACATT
GTTATAAGTGATGTCCACATGCCAGACATGGATGGTTTCAAACTTCTTGAGCACATTGGTCTGGAGATGGACCTGCCTGT
TATAATGATGTCAGCGGATGATAGTAAGGATGTGGTTATGAAAGGGGTTACTCATGGTGCATATGATTATCTAATCAAAC
CGGTGCGGATTGAGGCACTAAAGAATATTTGGCAGCATGTTGTTCGTAAAAGGAAATATGAGTGGAAGGACAACAATTTT
GATCAATCGGGAAGTGTGGAAGAGGGAGATCGGCAGCAGAAACCATCAGAAGATGTTGATTACTCATCTTCAGCTAATGA
AGGAAACTGGAAAAACTCCAAGAAAAGAAAGGAAGAGGAAGAAGAAGTTGAAGATAGGGATGATACATCCAATCTAAAGA
AGCCACGTGTCGTTTGGTCTGTGGAGCTTCATCAACAATTTGTACAAGCTGTGCATCAACTTGGAATTGACAAGGCTGTT
CCCAAGAAAATCTTGGAACTGATGAACGTTCCTGGACTTACCAGAGAAAATGTTGCTAGCCATCTTCAGAAATATAGGTT
GTATCTCAGAAGGTTGAGTGGTGTATCGCAGCACCAGAATGGATTGAACAACTCTTTCATGGGACGCCCAGACGCAACCT
TTTGCACAATATCTTCCCTTAATGGACTTGATCTTCAAGCTCTTGCTGCTGCTGGTCAAATCCCTGCACAAAATCTTGCT
ACCCTCCAAGCAGCGGCCCTAGGTAAATCTGCTACAAAATCTGCCATTTCTATGCCCCTAGATCAAAGAAACTTTTTCAG
CTTCGAAAATTCCAAGTTGAGATTTCCAGAAGGGCAGCAACAACTGAATAATAGCAATAAGCAAATCGACTTGCTGCATG
GAATTCCAACCACCATGGAATCGAAGCAACTTGCAAATTTGCACCACCCTTCCCAGTCCTTCGTGGGTATGAATATGCAA
GTGAACTCCACATCACAACACAATAATTCTGTGCTTATGCGATTGTCCCAGTCACAACCTAGGACTCAACTGCTCAATGG
AGCGAATAATGGTAGTCAAGTTTCAAGGCTTCCGTTGTCCATGCAGCAATCTTTGTCCCCGGTCCTAACACAAAGTGGTA
TTGTCGACAATGCACGAGGGAGTGTGTACAATCCAGTCTCTCAAGCATCATCAATGGTAGATTTCTCGGTAAATCAAAGC
AAGGAGTTGCAAAGCTACAACTTTTCTCTTGGGAGTAATAGTTTGGGGATGTCTTCTTTGACTAATCGAGGGATGCTTCA
GGAAGAGGTGAATTCTGACATCAAAGGATCTAGAGGTTTCCCTTCTAATTATGATATTTTCAATGAACTCCAGCCAAAAT
CGCAGAACTGGGGTTTACAGAATGATGGTTCAACCTTTGACACGTCTCTTCATCCATGTATGCAAGGAACTCAGGATGTC
TCATCACAACTGTTACTTCAACAGGGAATTTCTTCAACTCATAACAGTGGACAAAACAGAAATGCTCCAATTGAGAAACC

AATGTATTCCAACGGGGATGAGATTGGACATTCTAATACTATGTGTGGCTCACAACTTAACTCTGGTGGTGGGAATATGC
TTGCTGTTAAAGCTGAAAGATTTCCTGACGCAGACTATCAGAATACCAATATCCGGGAGCAATTTGGACGTGATGACCTC
ATGAGTGCGTTCCTTAGACAGCAAGCAAGTGTTGGACCTGTTGAAACTGAATTTGGCTTCGATGGATACACATTGGACAA
TCTTCCTGTTTAA

>CcARR3 |Locus_ID=BC332_31526 |CDS_len=1707 |strand=-
ATGGCTACTTCCGGTGAATGTTTGAACTCCGGTGGATTTTCCGGTGAGTTTCCGACGTTCCGGTTGCTGGTGGTAGATGA
TGATCCTTCTTGTTTAAGAGTATTGGAATGCATGCTTAAGAAGTGTCACTATGAAGTCACCACGTGTGAGCACGCAGAGG
TTGCATTGTCATTGCTCCGCGAAAACAGAAACAACTTTCACTTGATTATAAGCGATGTGCACATGCCAGACATGGACGGA
TTTAAACTTGTCGAGCTCATTGTACTGGAGATGAACCTCCCTGTTATAATGATGTCCGTGGATGACAGTAGAAGTGTAAT
TATGAAAGCCGTGATTCATGGCGCACATGACTATCTGGTTAAACCGGTCAGCATTGAAGCGCTAAAGAACATTTGGCAGC
ATGTTGTTCGACAGAATAAGGACGAGTGGAAGGGGAAGATTTTGGATCGATTAGGAAATGTTGAAGGTGCTGAGTACTCG
GATGCAGCTAAAGAAGGGAGGCTGAAAAGCTCGAAGAAGAGAAACGACAAGGAGGAAAATGAAATCGGAGAAAGGAATGA
TACGAATACAGTGAAGAAGGCGCGTGTACTTTGGTCACCGGAACTTCATGAGAAGTTCATACAAGCTATCAATCAACTCG
GAGGGGACAGCAAGATTCCCCAGAAAATTCTGAAACTGATGAATGTTTCGGGGCTAGAAAGAGAACACGTTGCTAGCCAC
CTTCAGAAATACCGACTGCAACTACAGAAGAATCGGAGTGGACTGGAGAGTGGTTTCATGGGACATTCGGAAGTAACCAT
TGGGACACTGCTCAACAATCCTGATGTTCAAACTCTCGCACCTATGGATCAACATCCTGTTCAAAATCTTACTACCCACC
AGGGGCCGACGTGGGTTAGGCCGAAATCTCCCATATTTATCAAAGAGCCGACATGGGGTAGCCCTACAACAAAATCTCCC
ATATTTATGCAGAGAAACCATTTAAGCTTTCAAAATCCGGTGTTGAGATATCAAGGTCCGCAACAGATGAACAATAGCAA
TAAGCAAGTTAACTTGCCTTGTGGAATTCCAACAACTGAACAATTCTTGAGAATGAATATTCCAGTAGCACGGATTTTTC
AACCACAGTTACAAGTTCAGAATATGCTCAGTGAATCCAATAAGATCCTATCACAGAATGGTATTGTAGACGATACAAGA
GATAGTATGTACAATCAGGTTCCCCGAACAGCAGTAGATTTCTCATTGAATCAAAACATACCGCTGGCAGGAACCAACTT
TCTTTTGAGTGCCAATTCAACTGTATCAGCAACTACGGAACAGGTTAACATTGGTTATGACAATTTCAGCGAATTGTGTC
AACAAAAGGCACAGGACTGGGGCTTACAAAATGTTGTTGGATCAACCTTTGATGCCTTGATGGAGCAAAATGGACAGAAC
GTAAATGCTCCTATTGCGGAAGCAGTTTTCTCTAGTAGGCAAGAAATTGGACATGTTAATCCTATCGATGGTCCACAAGT
TAACTCAGTTAAAGCAGCTGAAAGATATACCGATTCAAGCTATCAGAGCACTATTTTTCTTGAGCCATTTGAACAAGATA
ACGACCTCATGAGTGCCCTTCTCGAGCAGCAGCACGAAAGTTCTGTTGGACAAGTTGACAGTGTGTTTGGTCACGATGAA
GATGCATTCGAAAATCTTCCAGTGTAA

>CcARR4 |Locus_ID=BC332_13151 |CDS_len=1899 |strand=+
ATGGCAAGTATGAGTGGCGTCGGTGCCACGTGTAAGTCGGAAGCTACAGTGGTTGCTGATCATTTTCCTGTAGGGTTAAG
AGTACTTGTGGTTGATGATGATGTTGTTTGTCTTCGAATTATTGAACAAATGCTTCGTAGATGCAAGTATTCAGTTACTA
CTTGTACCCGAGCTACTGTGGCATTAAACCTATTACGTGAAAGGAGGGAGACTTTTGATATTGTACTGAGTGATGTTCAT
ATGCCTGATATGGATGGTTTTAAACTTCTTGAGCTTGTTGGATTGGAAATGGACCTTCCAGTAATAATGATGTCAGGTGA
TGGGAGAACAAACCTCGTCATGAGGGGAGTTCAACATGGGGCTTGTGATTATTTGATTAAGCCTATACGGGACGAGGAGC
TAAAGAATATCTGGCAGCATGTTGTTAGGAAAAGATATAACTCCAATAAAGAGCATGAATGTTCTGGTAGCTTGGATGAT
AATGATCGGTATAAGCGAGGAAGTGATGATGTGGAATGTGCTTCTTCTGTAGTTGAAGGTGCAGATGGAGTGATAAAACC
TCAGAAAAAGAAAAGAGAAGCCAAAGAAGATGACACTGAAGTGGAAAATGATGACCCAGGTACCACAAAAAAGCCACGCG
TAGTCTGGTCAGTGGAGCTTCATCAGCAATTTGTTAGTGCAGTCAACCAACTTGGCATTGATAAGGCTGTACCTAAAAGA
ATCCTTGAACTGATGAATGTTCCTGGTTTAACGAGAGAAAATGTGGCAAGCCATCTGCAGAAATTTAGGCTATACTTGAA
GAGGCTAAGTGGAGTAGTTCAACAACAGGGTGGCATACCCAGTACCTTTTGTGGGCCAATGGAACAGAATTCAGAACTTG
TTTCACTAGGGAGATTTGACATTCAAGCACTGGCAGCCTCTGGACAAATTCCTCCTGAAACTTTGACAGCTCTTCATGCC
GAGCTTTTAGGTAGATCGACAAGTAACTTGGTTTTACCAGCAGTAGAGCAACAGAATCTCCTACAAGCTTCCTTGCAGCA
AGCAAAGTGCATGCCTGTTGACCAACTTGTGGCTTATGGTCAGCCTCTGTTGAAATGCCCTCCCAGCATTTCTAATTCTA
AACATCCTTCTCAATCTATCTTGTCAGCTGAGGATGTTCATTCAGGATTTGGATCCCAGCGAGCAAAAAAATATATGCATG
GTACCTAGCAGCAATCCCACTGGGATAGCTGCTCCGAACAGCAACATGTTTATGGCAATGATGCAACATCAGCAACAATG
GCAAAAGCAGCAGCAGCTGGAACTGCAACATAGGCAATCTGGACCTCTTGAATCCAACTATTCAATTAATGTTCAACCTT
CTTGCGTAGTATTGCCCTCTCAGTCGCCTGGTAATTTCCAAGTGGGAGATAGTCCTGCTTCCATCAGCCGTGCCAACAGT
CTAAGTAAATCTTCGGTGGTTGATTATGGAGTTCTCTCTCCACAATCAAATAATTCATCAGGTGTGATTCAAGTGTTAGA
CAGGGAACTCAGACCAGAATGTGGCTCAAACAGGCTCTCAAGTGGAGGTTCCCTCTCTTGTTCATGCTCGATAAATGCTG
ATAATAGTGTTGGTCTGCAATTTCATAACTCGAGCTCAGCGTTTGGTTCTTCTAAACAACTTCCAGGCCTTAACCCAAAT
CATTTGGGCTCGCCAGTTCCATATAATATTAACTCAAGCCAGGTTCTTGATCAGGGACATATAAGAAATCCTGTGGTTGG
TAAAGGTGCCTCCATTCCCAGTCGCTTTGCAGTAGATGAGTGTGAATCGGCAATGTGCAATTTGAATACTGCAAAAAGCT
ATCTCGAGGACGCTAAAGTTAAGCAGGAGCCTAATATGAATCTCATGGAAAATTGCTAA

>CcARR5 |Locus_ID=BC332_28216 |CDS_len=1746 |strand=+
ATGATGATGGAGAATACTAGTAAAAACAGTGGAGGGTTTTCTACTCCAAGAAGTGATAATAATACTTTCCCAGCTGGTCT
AAGAGTTCTTGTTGTTGATGATGATCCTACCTGGTTGAAGATTCTTGAAAAAATGCTTAAGAAGTGTTCTTATCAAGTGA
CAACATGTGGTCTAGCACGAGAGGCTTTGTATCTGCTCCGAGAGAGAAAGGATGGATTTGACATTGTGATCAGTGATGTT
AACATGCCTGATATGGATGGATTTAAGCTTCTGGAGCATGTTGGACTTGAGATGGATCTTCCCGTCATAATGATGTCTGT
TGATGGTGAAACAAGCAGGGTGATGAAGGGTGTTCAACATGGTGCATGTGATTATCTTTTAAAGCCTATACGAATGAAAG
AACTTAGAAACATATGGCAGCATGTACTCAGAAAAAAGATGCAGGAGGCAAGGGACGTTGGAAATCATGAACTTGACCAG

TATGATGAAGTGTGGATGCTTAATGGAGCTGAAATCCTTTCAGGCAAGAAGAGAAAAGATTTTGACAATAAGCATGATGA
AAAAGAAATGTCCGAACCAAGAGGTGTTGATTCTTCTTCCATGAAGAAAGCCAGAGTTGTTTGGACTGTAGATCTTCATC
AGAAATTTGTCAAAGCTGTTAACCAGATTGGATTTGACAAAGTTGGTCCCAAGAAGATCCTTGACTTGATGGGTATCCCA
TGGTTGACTAGAGAAAATGTTGCTAGCCATTTACAGAAGTATCGGCTTTACTTGACTAGGTTGCAGAAAGAAAATGAAGT
TAAGGCCTCATTTTGTGGGGTGAAGCATCCAGATGTTTCTTCTAAAGAAACTTCTTCTAGTCTCAGCCTTCAGAATCCAG
TGGATGTGTGCACTGATGTTACGAATGACAAGTATGGTGGTGTTTCTGGAGGCAAAGCTATTGTTCAAAATGGGAAATCC
AGCATATGTGAGAGCAAGGTAAAGGTTGTTGTTTCAGTGCCAGCAGCAGAGCCTAGTTCTGTGGTTGGAGATAACTTTGA
TTCTCCTGCTGCTGTTTCAAAGATAGGCCTCAACGATTCCTTTGGATTGGTGAATACTAATGTTAAGAATTCTAAGGTAC
CCACTACATATTGCTCTACTGGAGAAGCTCCACAACCTCAATACAAGCAAGACTTTAATCAGTCAGCAAATGGGTCAAGT
CACCAACCACTGCCGGTGGTTTCTCATCAAATCCCAGTGGATCACACACAAGAGACTTGTTTTCTCAGCCATGTACCTTC
TCGTGAAGAGAGGGACAGACATCCAGATACAAAAAATAAACCTTCTTTCTTCAAGAGCAAAAGTGAAGGAGTAGGAAAAT
ATTCTCCAATAGAAAGTACTATTAACTTGTTTCAACAAGTGAATCAACAAGCAAATTTTCATACTCTTCAGCAAATGGCA
AGTACTACATGGACTGGGACGAGTCATAATGTAGTCAATGGTCCACAATCAAGCGCAGGAAATCTAACTTCGAGAAGTGG
ATCAATTGTTGCATCTGCAGGTGAGGACATGTGTGGTGCTTCTATTCTAGGGGAATGTTTTCTAGCTAATAATGGCCTTC
CGAACACAGAACAATTTGGCTACAATGATCCACAACCCATTTCTGGAGTTCCAACATGCTTGTATGATACGTTGAGGTTT
GATTATGAGTATCCAGTTGATTCATTGGAAGGTATTGTGATGGACCAAGGTCTGTTCATAGTCTAA

>CcARR6 |Locus_ID=BC332_00798 |CDS_len=1683 |strand=-
ATGATTTGCATTGAGGATGAATTATTGGGTTGGAAAGATTTCCCAAAGGGGCTTAAAGTCCTGCTCCTTGATGAAGATAG
CAACTCTGCTGCTGAGATGAAATCAAGGCTTGAGAAAATGGACTATATAGTCTACACGTTCTGCAATGAGAGTGAAGCTT
TGTCTGCAATCTCTAGCAAATCCGAGGGCTTTCATGTTGCCATTGTGGAGGTAAGTGCAGGTGATAATGATGGTGTCCTC
CAATTTCTTGAAAGTGCCAAAAATCTACCAACTATAATGACATCAAATATTCATTCTCTCAGCACAATGATGAAGTGTAT
TGCGCTTGGTGCAGTTGAGTTCCTTCAGAAACCATTATCAGATGACAAACTCAAAAATATATGGCAGCATGTAGTTCACA
AGGCTTTCAATGCTAGAAAGGATGTGTCCGGACCACTTGAGCCGGTAAAAGAATCTCTTCTTTCGATGCTACAGCTACAA
CCAGAAAAGGGTGAACCAGATGACAAAAGTTCAAATGGAACAGAACCTCTCATTGCAGTTGCGGACAACAATACCGAACA
GTCATCGGGCTGTGATAAATACCCTGCTCCCTCAACCCCTCAATTGAAACAAGGAGTGCGGTCAGTGGATGATAGTGACT
GCCATGATCATACTATCTTCTCAGCTGACCAAGACAGTGGGGAGCATGATGAAGATATAATTTTGAAAGAGGAGAATGGT
TCATCTCCTCATCAAACTATGGAGGCTGATATTGCTACCTTTTCACAAATTAATGACTGCGCTGACAATAGTGATGGTTC
ATCTCCTCATCAAAAGACGGAGGCTGATATTGCTACTACCTCTTCACAAAGTAAAGACTGCCCTGACAATAGCATTAGTC
ATTCTGCTGAACCTAGTAAAGCTTCTGGTCCCCATAGCTCAAGTGGGACTAAATCCAATAAGAAAAAGGTGAAGGTAGAT
TGGACACCTGAACTACACAAAAAGTTTGTTCAAGCAGTAGAGCAACTTGGTATAGATCAAGCCATTCCTTCTAGAATACT
AGACGTGATGAAAGTGGAGGGCTTAACGAGACATAACATAGCTAGCCATCTCCAGAAATACAGAATGCATCGGAGGCAAA
TTTTGCCAAGGGAAGTGGAAAGGAGATGGCCCCATCCGCAACCTAGAGATTCAGTACAAAGGAATTACTATCCTCATAAA
CCTGTCATGACATTCCCACCATATCATTCTAATCATGTCGCCCCAGCTGGTCAATGTTATCCTGCTTGGGTACCACCGGC
TAGTTATCCGAATGGTTTACAAGTGTGGGGTTCACCTTACTATCCGGGATGGCAGCCTGCAGAGACTTGGCACTGGAAGC
CTCATCCAGGGCTGCTTGCTGATACATGGGGCTCCCCTGTCATGCCACCATCGTTTGGATCGTATCCACCATATCCCCAG
AATGCTGGAATGTACCAGTCTCACGGAATGCATAACAGATATAGCATGCTAGAGAAGTCATTCGATGTTCACCCGGCAGA
GGAGGTGATTGATAAAGTAGTAAAGGAGGCAATAACCAAACCATGGTTACCACTTCCTTTGGGCTTAAAACCTCCTTCCA
TGGAGGGTGTACTCGACGAGCTTTCTAGACAAGGAATCTCAACCGTCCCTCCACGAATCAATGGCTCTCGTTGTTGGAGA
TGA

>CcARR7 |Locus_ID=BC332_15933 |CDS_len=1674 |strand=+
ATGGTTTGCACTGAGAATGACTTACTGGGGTGGAAAGATTTTCCTAAAGGACTTAGAGTCCTCCTTCTTGATAAAGATTC
TAATTCTGCCTCTGACATGAGATCAAGGCTTGAGGAAATGGAATATATAGTTTACGCATTCTGCAATGAGACTGAAGCTT
TGTCTGCAATCTCAAGCAAGTCTGAAGTATTCCATGTTGCCATTGTAGAGGTAAGTGCTGGCAACAGTGATGGAGGACTC
AAATTTCTCGAAGGTGCTAAAGATCTGCCAACTATAATGGTGTCAAATATTCACTCTATTAGCACCATGATGAAGTGTAT
AGCGCTTGGTGCAGTTGAGTTCCTTCAGAAACCATTATCAGATGATAAACTGAGAAATATATGGCAGCATGTAGTTCACA
AGGCATTTCATTCTGGAGGAAAGAATGTCGCTGAGTCGCTTAAGCCGGTTAAAGAATCTCTTTTGTCCATGCTAGAGCTC
CAACCAGTAAAGCGCGAAGCAGATAGTGAAAATTCTAATGAAGCTGAACCCTTGACCTCAGTATTGGAAAACCAAAAAGA
ATCACCACCTTGCTGCGATAAGTATCCGGCTCCTTCAACCCCACAACATAAACAAGGAGTGAGGTCAGTGGATGATGGTG
ATTTCCAAGATCATACTATCTTGTCAAATGAACAAGATAGTGAGGTACATGAAGGGGACACAAAATCTGTCGAAACTACT
TGTTGTGATTCTGTTGCTGAGACTAGTATCCTGGCAGATTCTGCCGGGCGACTAGAAGTGGCTATCACAAAGGATGAGCG
TGATTCTGCTGCTGATCAAAATATGGAGGATCCCATTGCTACTTGTTCACGAAGTAATGACTACCCAGCCGATGGCAGTA
CTCGTTCTGCTGAATCTAATAAAGCTTCTGGTCTCCATAGTTCAAGTGGGACAAAAGCTAATAAGAAGAAAATGAAGGTA
GACTGGACACCTGAACTACACAAAAAATTTGTTAAGGCAGTCGAGAAACTTGGTATAGATCAAGCCATTCCTTCTCGAAT
ACTAGAGCTGATGAAAGTAGAAGGCCTGACAAGACATAATATAGCTAGCCATCTCCAGAAATTCAGGATGCAGCGGAGGC
AAATTTTGCCAAAGGAAGACGAGAAAAGATGGCCTCGTCCTCAACTTAGAGATTCAGTACAAAGAACCTATTATTATCCA
CATAAACCTGTCATGGCCTTCCCGACCTATCATCCGAATAATGCACCCACAGCTGGTCAATTCTATCCTCCTTGGATACC
ACCAGGAGGTTACCCGAATGGTGCGCATATGTGGGGTTCGCCTTATTATCCTGGATGGCAACCACCCGAGAATTGGCACT
GGAATCCTCACTCAGGACTGTATGCTGATGTATGGGGTTGCCCCGTTACACCTCCATCTTTAGGATCATGTACACCGTAC
CTTCAGAATGCATCTGGGATACACAACAGATATAGCATAATACAAAAATCAGTTGATCTTCACCCGGCAGAGGAGGTCAT
AGATAAGGTGGTAAAGGAGGCAATACACAAGCCATGGTTGCCTCTACCATTGGGCCTAAAATCTCCTTCGACGGAGAGTG

TTCTCGACGCGCTTTCCGAACAAGGCATCTCCGCCGTTCCTTCACGCATCAACGGTTCTCGTCGCCCGCATTGA

>CcARR8 |Locus_ID=BC332_23287 |CDS_len=1284 |strand=-
ATGGCAGCTCCCTATTACGGTATGATTGATGAAGTTCATGTGGTGCTTGTTGATGACGACAAAGAGTTTGTTAATAACAT
GGTTGATTTACTAAAGTCTTATGACTATAAAGAAGATACTCCTTATTGGACTAAACTTTTAGCACTATTATGTACTTACT
GTGATAGCCAAGATGTGATAGATAGGGCAGGGAGCATGATCTATAATGGTAAATCTCGTCATATAAAATGGAGGCATAAT
ACCATTAGAGAACTACTCTCAAGTGAAATTATCACGGTTGACTATGTGAGGTCAAAGGATAATGTGTTGATTCCACTAAC
AAAAGATCCATCTAGAAAAGGAGTTAAGAGTTCATTGAAGGGAATGGATTTACGGCCTAGGACAAGTCATCACTGTGTTA
TGGTGGTTGGTACGGCTTTAGATGCAATGGCAATGCTCTCCAAAGGAAAGCAAAAAATTGATGTAATGATACTCAATATC
CATTCATCGAACACACTTTCTTTTCACCTCTTAGCTCGAGTTGTGGCCTTAGATATAATTTCACTCGTTGTATGGGATGA
ACAAAACGAACTCGTAGTAAAGAAGGCTTTTGACGAAGGAGCTTATCTTTGCCTTAAAAAGCCATTTGATAAGGACATTA
CCAAATACATCTGGCAATTTGTTGTAGGAGAAAAACTAAAAAGAGAGAAAGCAAGAAAAGGATTCGAAAATAATAAAAAT
CAGATGAATAAGATCGTTAAGCGAAAGAATTGCACAGAATGGACTGAGGCTCTTCATGCCAAATTCATGGAAGCTGTACA
ATTACTTGGTGAAGGAAGATGCTATCCCAAGGAGATTCTTGAGGTTATGAATGTGCCTGGTCTTACTAGGATGCAAGTTG
CAAGCCATCTCCAGAAATGTCGTCGCAATGATTGGAGATCTCCGAAAGAACGAAAATCTCTTCATCGCCCATTAGGTCAG
GGATCCTCAAGTGATTTTCATGAATCAAGAAGCAATGGGATAGTATCTAGTAGAAATATTAGAAATGAGATAAGTAATGG
CTACAACTTGAATGTCAGTATGGATAATGTGACATCATATTCAAGTAACGCAACGATGTATGATACTTATGTTGGAAATG
TGGCTATTAATGGATTGGAAGAAGAAAATCCAAATGTTCAACAATATATTGGTGAACCAAATATATCTAATATGAATAAT
CCCTTCCAGAAGCTTGAACCTCCAAGTGCAAGCCTACATAATGAACAAGACAATGACTTTGATCAGGTTTACTATGATGA
TTAG

>CcARR9 |Locus_ID=BC332_23270 |CDS_len=873 |strand=-
ATGAATAAGATCGTTAAGCGAAAGAATTGCACGGAATGGACTGAGACTCTTCATGCCAAATTCATGAAAGCTGTACAATT
ACTTGGTGAAGGAAGATGCTATCCCAAGGAGATTCTTGAGGTTATGAATGTGCCTGGTCTTACTAGGATGCAAGTTGCAA
GCCATCTCCAGAAATGTCGTCCCAATGATTGGAGATCTCCGGAAGAACGAAAATATCTTCATGGCCCATTAGGTCAGGGA
TCCTCAAGTGATTTTCATGAATCAAGAAGTAGCTTTAAAAAATTTGGGACAATGCCTCATCTTCAAATAAAGGAACCAAA
TCTGAATTGTAACCCAGATAATACCCAAAGAAATTCAGATCTTTCATTTCCAAATCTTAGTACCAATAATACTTTTGCTC
GAGAAGAGAGTCCATTTCAACAACATTTTTATCGCCCGCAACTTCATGTTCAATCTCCCTACCTCAGCAACTTCAACCCA
TTCAGTAACCCATTTTTATCAATTCAAAATACTATTAGTGGTGGATTACAACAACAACAACAACAGAGATCATTATTCGG
AATATTGTATTCACAAGGACTACAAGGCCCAATTATTAAAAATAATAATTATAGGTCTGATGTTGTGATAAACAGTGGGC
ATCATCATACTCAAAGTGATTATACCTTGAATTCTATTGTAGCCCAAGGAGCAACATATTCAGGTAGTGGGATAGTATCT
AGTAGAAATATTAGAAATGAGATAAGTAATGGTTACAACTTGAATGTCAGTATGGATAATGTCACATCATATTCAAGTAA
CGCAACGATGTATGATACTCATGTTGGAAATGTGGCTATTAATGAATTGAAAGAAGCAAATCCAAATTTTTAA

>CcMYBH1 |Locus_ID=BC332_28265 |CDS_len=894 |strand=+
ATGGGTGCACCTAAGCAGAAGTGGACTTCAGAAGAAGAAGCTGCTCTTAAAGCTGGGATACTTAAGCACGGGCCAGGTAA
ATGGCGCACGATTCTCAAGGATCCAGAATTTAGTGGAGTATTGTTTTTGCGTTCAAACGTAGATCTCAAGGACAAGTGGA
GGAACATGACTGTAATGGCAAATGGTTGGAGTTCTCGAGAGCAAACAAAGTTGGCTGTCAAAAAGATGAAACAGGCCCCT
AAACAGGATGGGAGTCCTATGACTGATACGACTGCTGCTGAAAGTGATGAAGAAGCTGCTGAAGCAAGGCCAGCCACAAC
TTCTAGTGGTTCTCCACAGATACATGGTTCAAAAAAATCTATGATAAGGTTGGATAATCTTATAATGGAGGCTATAAGCA
GCTTGAAGGAGCCAGGTGGTTCTAACAAGACTACAATAGCTGAATACATAGAGGCTCAATACTGGGCACCTCCAAACTTT
AAAAGGCTATTGTCAGGGAAACTGAAGTATTTAACTGCAACAGGAAAACTTATCAAGATGAAGCGTAGGTATAGAATAGT
ACCTACTTCAACACCATCTGACAGTAGAAGAAACCTGTCCATTACACTTTTGGACAACAAACAGAGGATCTTCTCCAAGA
TTGATCGTGATGATTTGAACATGCTTACATCTCAGATTGATTTAGAGTTGGCTAAAATGAAGCACATGAAACCTCAAGAG
GCTGCAGCTGCTGCTGCACAAGCAGTTGCTGAAGCAGAAGCAGCTATAGTTGAGGCTGAGGAGGCGGATAGGGAGGCTGA
TGCTGCTGAAGCTGATGCTGAAGCTGCTCAAGCCTTTTCAGTAGCTGCAAACAAGACACTACATGGGAGAAGTGCCCCAA
GGATGGTAAGACAT

>CcMYBH2 |Locus_ID=BC332_09595 |CDS_len=867 |strand=+
ATGGGGAATCCGAAAGTGAAGTGGACGTCGGATGAGGAAGATGCCCTAAAAGCCGGCGTAGCAAAGCACGGTACCGGAAG
GTGGAAGAATATCCTTAGGGATCCTGATTTCGCCTCTCTTTTGTCCAACCGCTCTAACATCGACCTCAAGGACAAATGGC
GAAATATGTCTATTTACCCTGCTGGGCAAGGCTCTAAAGATAAATCTAGAGTTGCTCGTGCAAGACCAATGGCAATAGAT
GCACTTGTGCCTTCCACTGCTCAAAATGTTACGCCTATTGCTTCAGCATTCGAGCCTTCCCTTGCTCAAAGTGTTCCGGC
TATCACTTCAGCATTCGAGAATGAAGCCATTGATGATCCTCCCAAAAACTCCCAAGAAGGCCTTCACCTGTCTAATGATA
TGATCTTTGAAGCTCTGTCATCCATGAAGGGTTCAAATGGATCTGATCTTGGTGCCATTGTTGGATTTATTGAGCAACGA
CATGAAGTACCACAACATGCTAGAAGGTTATTGACAACAAAGTTGAGAAGACTCGTATTGCAAGGAAAACTTGAAAAGGT
CGAGAACTGCTATAAGATGAAAGATGCAACATCAGAAACCAAAATACCTACTCCAAAACAAAAACATGTCCGCTCATGGC
CAGTTCCAAATTCGGCTGTAAAAGTCTCCTGTGGAACAACAGAAGAAGCTGCAAGGATTGCTGCTGAAAAGATTGTTGAT
GCAGAACATAAGTGTTTCTTGGAAGTTGAAGCGATTAAAGATGCAGAAGTGACGTCAAAAATGGCTGAGGAAGCAGAGAC
AATGTTACTACTTGTCAAAGAGATCTATGAACAGTGTTCGCGAGGTGGAGTTGTTCTCTTGGCTTGA

>CcMYBH3 |Locus_ID=BC332_18613 |CDS_len=843 |strand=-

ATGGGAAATCCAAAGCAGAAATGGACATCGGAGGAAGAAGAAGCACTTCGTGCTGGCGTCGCAAAGCACGGAGCTGGCAA
ATGGAAGAATATTCAAAGAGATCCCGAATTCAATCACCTCCTTTACTCTCGCTCCAATATCGATCTTAAGGATAAATGGA
GAAATTTAAATGTTAGTGCCAATGGACAAGGGCCAAGAGATAAATCAAGGACACCGAAAGTGAAGACTAATCTTGATGCT
GCAGCAGCTCCATTGCCCATCATGCAGGCCCCTGTTTCTTCCACTCTGGTTCCACAAGATGCATCAGCCGACACTGTTAT
GGAAGATTCTTCAAAATGCGCATTAGATGGAAAAAATGCTTCAAAGTATAATCAAATGATATATGATGCACTTTCAAGTT
TGAAAGAGCCTAATGGATCTGACACAAGCACACTTGTTAACTTCATTGAGCAAAGGCAGGAGGTGCCCCAAAACTTCAGA
AGGCTGCTGAGTTCTAGGCTGAGGAGGCTTGTTCAACAAGACAAACTGGAAAAGATTGAAAATTGCTACAAGATTAAGAA
AGAGGTTTTGGAAAAAACAAAGATAGCTACCCCAAAACAAAAACATGTTGGGCCAAGGCAGTTTCCAAATAGTGTCTATC
TTGGTGACACTGCCGAAGAAGCAGCAATCACTGCTGCATATAAGATTGCAGAAGCTGAAAATAAATCATTTGTAGCAGCT
GAAGCGGCCAAGGAAGCTGAGAGAGTCTCAAAGATGGAGGAAGAAAGTGATAATTCACTACAGGTGTACAAGGATATCTT
TGACAAATGTTCACATGGAGACATTGTGCTTCTGATGGCATGA

>CcMYBH4 |Locus_ID=BC332_03653 |CDS_len=879 |strand=+
ATGGGTGCACCAAAGCAGAAGTGGACAGCAGAAGAAGAAGCTGCCCTTAAAGCTGGTGTAGCGAAGTATGGAGTTGGCAA
ATGGAGCACTATTCTCAAAGATCCAGAGTTTGCTGTTGTGTTGCGCTCTCGTTCAAATGTGGACTTGAAGGATAAATGGA
GGAATCTACATGTCATGGCTAGTGGTTGGGGATCTCGGCATCGAGGAAGGGCTGTTTCTAAAAGTGTTCAGCCTGCACCG
AAGCACGATGACAACAACTTGGTTAGCTCTGTGGGTGAGAATGATATAGAAGTTCTTGATGCTAAACCTCTTGCATCGTC
TGGTGAAGTGCTGAAGGATGTTGGCTCTAAAAAACCTCTTACAAGATTGGATGATCTTATACTGGAGGCAATAGTCAAAT
TGAAGGAACCACGTGGATCTAGTCGGAATGCAATTTCTTCTTTCATAGAGGAGCGCTTTGCGGCACCTCCACACTTTGAA
AAATTGTTGGCAACAAATTTGAAGGCATTGACTGAAAAAGGAAGACTGGTAAAGGTAAAGCATTACTACAGAATTGCACC
GAGTAGAGTATCATCTGATGCCAAGGGTGGCTCTTTCTCTTCACTTATGGGTGGGAAGCTTGATTCCACTGTGGTAGAAA
AGAATGAAACTAGAATACTGACTAAAGCTCAGATTGATGCGGACTTGGAGCAAATGAAGAGCATGAGTGCGGAGGAAGCT
GCTGCAGCCGCTGCACAAGCAGTTGCAGAGGCTGAAGCTGCAATTGCTGAAGCAGAACGGGCAGCAAGGATTGCAGAGGA
GTGTGAAGCAGAGGCAGAGGCAGCACAGTGCTTTGCTGAAGTAGCATCAAAGGCATTGCAGCATCAAACTATCCATGTC

>CcMYBH5 |Locus_ID=BC332_32845 |CDS_len=3201 |strand=+
ATGAGTTATTATGATCAACCTCCTCCCGTTGGTGTACCTCCCCCTCAAGGATATCCACCAAAAGATGCATCATATGGGTA
CCCTCCTCAAGGGTACCCTCCGCAAGGTTATCCTCCTCAAGGTTATCCTCAACAGGGTTATCCTCAGCAAGCCCAACAAG
GTTATCCTCAGCAAGGTTATCCTCCTCCGCCATATGGTGCCCCTCAATACGCCGCTCCACCTCCTCAGAAGAACTCATCT
GGATCGACCGACATCCTGACTGGCTGGATTTTTTTTTTGAATTCTTGGATGGGTGCACCTAAACAGAAGTGGACTCCAGA
CGAAGAAGCTGCTCTTAAAGCTGGGGTCCATAAGTATGGACCAGGCAAATGGCGGACAATCCTCAAGGACCCAGAATTTA
GCAAAGTGTTGTCCTCACGTTCAAATGTAGATCTAAAGGACAAGTGGAGGAACATGAGTGTGATTGCAAACGGGTGGGGT
TCTAGAGAGAAAGCAAGGTTGGCTCTCAAAAGAATGAATCAAGCCCGTAAACAGGATGAGAGTTCTTTAGCTGTCATTAC
TGAGGCTAAAAGTGATGAGAAAATGGCTGAAGCAAGGCTAGCTACTACTTCTATTGGTTCTCCACAGATTGGTGGGTCAA
AAAGATCTATCATAAGATTGGATAATCTTATAATGGAGGCTATAAGCAACCTGAAGGAGTCAGGTGGTTCCAACAAGACT
AGTATAGCAACATACATAGAGGATCAATATTGGGCACCTCCAAACTTTAAAAGGCTGCTTTCAGCGAAACTGAAGTACTT
AACTGCAACTGGGAGACTTACCAAGGTGAAGCGGAAGTATAGAATAGCAACAATACCTGTATTATCGGACAAAAGAAGAA
TCCCTTCCATTCCGCTGTTGGAAAGCAGGCAGAGGATCTCCGCTACAGTTGATCAGGATGATTTCAAGATGCTCATTAAG
GCTCAAGTAGATTTAGAGTTAACTAAGATGAGGAGTATGACACCTCAAGAGGCAGCAGCAGCTGCTGCACAAGCTGTTGC
AGAAGCAGAAGTATCCATGGCAGAAGCTGAGGACGCTGCTAGAAAGGCTGATGCTGCTGAGGCTTATGCCGAAGCTGCAC
AAGCCTTAGCAGAAGCTGCAATGAAGGCTCTAAAAGAGAGAAGCAGCCCAAGGATTCCAACTCTCTTGTTGGCCTGTAAA
TATGGTAAAAGTGGTGTTTGTAATAGCAAAGGCCTTCCAGCCATGGATGGAAATTTGAATGATAATGCATTTTTGCGGGC
TCGGATCAAAGAATTGGAGCGCGAGCGTGATGAATTGCATAAAGACATTGAGCAGCTATGCATGCAACAAGCTGGACCTA
GCTATATTGGGATGCCTACTCGCATGCCTCTTCATAGGATAGCTGGGTTGGAGCAGGAGGCTGAAAGCTTGAAAAAGAAA
CTATCTGCTTGCACACGAGAAAATCAGAATCTTCAAGAAGAGCTATCAGAAGCTTATAACATCAAAGGTCAACTGGCTGA
TCTACACAGCGCAGAAGTTTCCAAGAATATAGAAGCAGAAAAGCAGCTTAAATTTTTTCAAGGTTGTGTTGCTGCTGCAT
TTTCTGAGCGAGACCATGCTGTAATGGAGGCTGAAAAGGCCAAGGAACAGGAGGAGCTTATGTCAAGAGGACTTCATGAA
CTTCAGCAGAGGATTGAAGAGCTAAGCTGTGAATTACTTGAAGAGAAAATATTGACGGCTACTTTGCAGGGTGATATGGA
CAGGGAAGAGAGGTTGGATGAGATTTTCAAGGAGGTTGTCCATAAGTTCTATGAGATTAGACGGCAGACATTGGAGGATA
TTGAAGATGTGTCCTGGGAAGATAAATGCGCGTGCCTCCTGCATGATTCGTCTGAAATGTGGACTTTCAGCAATCCTGGG
GAAACATCCACCTCTAACTACATTAGTTCTCTGGAAGAACAAGTGGAGACACTGCGAAAATCTTTGGATAATCTTCAGTA
CAAGCTACTAGTAGGTCTTGAAATAGAAAATCACTTGAAGAAAAAAGTGCGCGCCTTGGAAAAACAGAAAATTCTTTCAG
AAGAGAAGCTGAGGGCTCAAATTTCGGCATTTCGCCACTATCATTCTCAGCATAGAGTTAATATTACAAGCTTTCTTGAT
GAGGGATTTTCTCATATTAAATCAGCTATGAACATGGTGGAAGAAAAGCTCAAGGACTGCAGCATAAGTGAGAGAGACTT
AAATTCTTCTCAAGTGGATGATTTACAGTTCAGTGAACTTGAATGTCAAGATGTTCAGATAAAAAATGTTGATGGTTCAG
ATTTGATCTTTAAGAGGAATCAGCCTAGCTTGACAACTACCATTACTGTTGGAAACAGTGATGCTTCTAAAGCCCTTGCT
GTGGCACTAAACGAGAAGGTGGAAACGTTATTACTTCTCTCACAACAAGAAGAAAGACACTTATTGGAGAGGAATGTCAA
TGCGGCACTGCAGAAAAAAGTTGAGGAGCTCCAGAGGAACTTACTACAAGTTACAAATGAGAAGGTCAAAGCTCTGATGG
AGTTGGCACAGTTGAATCGGGACTATCAAGTATTGCATGAGAAAGTTAATGAGGCCTCTAGACAAGGAAAATCTCTTGGT
GAGATTGCAGTGAAAAGGACTCTCCAGGAAAAAGATGGAAGACTGAAAAATCTGCTGAAAACTTCATATTTGAGACGCTG
GACTGGTATACAAGATTCAGATGTAAATGATGCTGACACTCATCGAGATTCTGAAGCAAATTTTGCTGATAGGAGGCCTA
CCAATAACGTAGATTTTGCTAGGATGAAGATAGAGAATGCAACTTTAAAAGAGAGCTTAGAAAGTATGGAGCATCTAATT

CGTGCAGTTAGGAGGCTTCGCCTTTCACTTTTAAAGTTTAAAGAGTCTGCTGCATCCGAAGGTATGGAGAACTGCTACTC
GGTGTCATTGGACAATATTATTAATGAGGCAAGTCAGTTAAAAACTGCACTTGGAAGCTCCCTCCCTCTTAGTTGGTCAG
CGGAGGCAGATTCAGGATCAGTTAGCAAACATGTTGAAGAGGAGACAGATAATGGACACTCTACACGTGAAAATATGGAC
TTTGTTTCTGCTGCTGGATTTGAGATGGTTGAGCTTCTGGTGTTTGTCGCGCAGCTATTGAAAGATTACAAATGCAGTTG
A

>CcSWI3A |Locus_ID=BC332_08722 |CDS_len=1806 |strand=-
ATGCATAACTATTCAAGCAAACCAATTAGTATTCATTTCACAAAGGGCTATTTGTCCACTTCCCCTCATTACATAGTAAA
TCAATTCCTTGTTTGTGCAGGTGAATTTGTAATGGACACATCTCGCTCGCAAGACGAACCGGACCACGACCTTTACACAA
TCCCTAGTTACACCAGTTGGTTTTCTTGGCAAAGCATACACGAAGTGGAAAGGCTATCATTGCGAGAATTTTTCGATGGA
AGTTCGATTACAAGGACACCCAGAATATACAAAGAGTATAGGGATTTTATTATTACATCCTACCGTGAAGATCCGACCCG
AATGCTATCGTTTACGGAGATCCGAAAGGCGTTAGTGGGTGATGTGACTGTGTTACATAAGGTGTTTACGTTTTTGGAGA
AGTGGGGTTTGATTAATTTTGACCCGAGTAACGCGGCTGCGGCTGCGGCGGCGGTTGGTGGTGTTGCGGAGGAAGAGGAT
AAGGAGGATGAGAAGTGGAGGATTAGGGTTGAAGAAGGGGCCCCACATGGGGTTAGGGTTGTGGCAGCTCCGCATTCTTT
GAAACCACTGATACCTGTGAAGGAGGTGGTTATTGGGGATGGAGGTGGAGGTGGTAGGGTGGGTGGTGTAGTAAAGGTGT
CGCCTTTGGCATCGTATTCTGATGTTTATGGGGAGTTGGTTGGGGTGGAGAAGAAGGAAAGTGTGGTGTGTGAGAGCTGT
AAAGAGCAGTGTGCTTCGGGTTATTATGAGTACAACAAGGATGCGAGCATTAATTTATGCGAGAAATGTTTTAAAAGTGG
TAATTATGACAAGAACAAATCGACAGATGAATTCAAGTTAATGGATGGGGCGAACCCTAACGCCAACTGGACTGAAGCAG
AAACTTTACTCCTATTAGAATCTGTACTGAAGCATGGTGATGACTGGGATCTTGTTACTCAAAATGTCAAAACAAAGAGT
AAACTGGATTGTATCTCAAAGCTTATACAGTTGCCATTTGGGGATTTTATGCTTGGTTCTATTCATAGAAAGCTTAATTT
TTTGGACAAGAATGGTGAGGTAAGTGGCGTGGATCAAGCTCAACCTGCAATAAGTGAGTCTGGAGAAACTCCTGGAAATC
AATCTCATGAGCAGAACCAGGAGCATCAACAGAATGGAGATGCTGAAGACGAAACCCCTCCTCTCAAGAAAATACGCAGG
GCTCCAATTTCGGAAGCCAGTAGTTTCCTGATGAAACAGGTAGCTCGCATCTCTGGTGCTGTTGGTTCACATATCACAGC
ATCTGCAGCTGAGGCTGCTGTTACAGCCCTTTGCTATGAGAACCAGTGTTCCACTGTGATTTTTGATGAAGACGACGATG
GATTGGGATCGATTGCTGATATCAGTGAGACAGAGAGGGCGAGTCAAGTCGAAAGTGCTGAAGGGGTGGAAAAGCCTGGC
AGATCAGAGACAGAGGTGGATGTCTCTCAGAGGAATACTATACCACTAACTTTACGTATGAGGGCTGCAACTGCAACTGC
TGTAGGTGCTGCTGCTGCTCATGCCCAATTGTTGGCGGATCAGGAAGAGAGAGAAGTAGAATATTTAGTTTCTACATTGG
TTGAAGCTCAGATGAAGAAGTTGCAACGCAAAATGAAACATGTCGAAGCTCTGAATCTGATGATGGAGAAGCAGCATGGC
CAAATGAAGGATCTAGAAGAGTCTTTAGTTGCAGAGAGAATGGACGTCTTGCAGAAGATGTTTAGTGCCGGGGTATCTAG
ATGGAAGGATCATGCTCCGGTCAAATCTCAAAGCAGCAGCGTATAG

>CcSWI3B |Locus_ID=BC332_13234 |CDS_len=1536 |strand=-
ATGGCCGAATCCGGCGAAATCAACCCACCGGCACCTAAACCCAACCCTGAAACTACCTCTCCGCCACCGCCGCAGACAGA
GCCACAGACACCATTACTGCCGCATCCGCCGCCGCCACCACCGCAACCGGGATCCTCCCAAGCAAAGCGTCCCCAGCAAC
CGGAATCCTCCCCAAACCCGACGACCCCTTCCGCCACCGAATCCAAAGACAAAGACGTCACTCCCACCACAGCCATATCC
TCCCGCCTTGCTGAACCTGAATTCGTCTATATTCCCAGCTATTCCCGTTGGTTTTCATGGAATAGCATTCATGAATGTGA
GGTTCGCTTTCTTCCTGAATTCTTCGACGGAAGGTCACCTTCTAAGAACCCTAAGACATACAAATACTGTAGGAATACAA
TTATTCGTAGATTCAGAGATAACCCCACTAAAAAGATCACCTTTACTGAGGTTCGCAAGACCATTGTTGGAGATGTGGGT
TCAATTCGTAAAGTTTTCGATTTCTTGGAGACATGGGGTTTGATTAATTACACTGCTAACACTTCCAAGTCTCTCATCAA
ATGGGAAGAAAAAGAGTCAAAATCAGCATCTGCATCAGCTTCGCATAATGCTGACGCTAATGGAACTTCCACTGATTGTA
CTGTCCCCAAGAAAAGGGTGTGCAGTTCCTGCAAAACTATTTGCAGGATTGCTTGTTTCGCTTCTGATAAGTATGATTTG
ACTCTTTGTGCAAGATGCTATGTTAGTGGTGATAATAGAGTCGGTGTGAATAATTCAGACTTTCGGCGAGTTGAGATTAG
TGAAGAAGCTAAGACAGATTGGACGGACAAAGAGACTTTGCTCCTTTTAGAAGCTCTTCTGCATTATGGTGATGATTGGA
AGAAGGTTGCTGCGCATGTTGGTGGCAGAAGCACGAAGGAATGTGTGGCTAGATTTGTGAAGCTTCCTTTTGGTGAGCAG
TTTATGGGCCCTCTTGAATCTACAGAAGTGGATAGTGAACCCAAGTCTGAAACTATGCATCCTCAGTCCAAGAGAATGCG
CCTCACTCCCCTTGCCGATGCAAGCAACCCAATAATGGCTCAGGCTGCTTTCCTCTCTGCTCTTGCAGGAAAAGAGGTTG
CAGAATTAGCAGCACATGCTGCTGTTACAGCTTTGTCTGAGATTGGAGCGGGAATAACCAAAGGGAATCTAGGATCTCTA
CTCGGTGGTGCAGAGGGGCAAGAATCGGATGGCACTTCCAATGGGTATGGCAAGGATATCGAAAGAGCTATTGTTGAAGC
AAGGCCTCAGCTTGAGAAGGAGGCACTGGACCTGGAGAGAGCCGTCTCCGATGTGGCAATTGAGACCAAGGAGATTGAGG
AAAGAATTGCTCATTTTGAGGAACGTGATTTACAGATGGAAAAGGAGTGGCAACAGCTGATGCAGCTTAAGAACCTGATC
TTCATTGATCAACTTACTTTGCTGCTGAATAAAGCTAGTGCTCCAAAAACTGGAGAAACCATTGGGGAGGAGGTGATAGA
TGTTAAGGCAGAATGA

>CcSWI3C |Locus_ID=BC332_15732 |CDS_len=2304 |strand=-
ATGAAGAATCAGAACGATGAGGACGGGTTCGAAGACGAGGAAGATGAGGAAGAAATTGAGCAACAAGAGATGGTTGATGA
CCTCCAAAACCCTAATAATTCAGTGGATCGAAGTTATACAAACTCCGTTGTTGAGTTGGTGTCGGAAAGTAAGGAGAGGA
TTAGTGAGTTTCCGATGGTAGTGAGACGTGCTGTAACGCGGCCGCATTCATCCGTGTTGAATATTGTGGCGATAGAGAAG
GCTGGGCAGAGTGGGGAGAGTAAGCAGAATGGTGGTGTGGTATTGGAGAATTTGTCGTATGGGCAGCTTCAGGCACTTTC
GGCTGTGCCTGTAGATCATCACGCGTTGTTGAGTGATGAAAGAGGAGAAGGGAGTGGGAGTGGTTCGTATGTGATTACTC
CGCCGCAGATATTGCCTGGTCGCGGTGTTGTTAAGCATTATGGTAGTGCAGGTCGAATTCATGTTGTGCCTATGCACGCA
GATTGGTTTTCACCTAATACTGTGCACAGATTAGAAAGACAAGTGGTACCACATTTTTTCTCTGGAAAGTCAGCTGAACA
TACACCCGAAAAATACATGGAATGTAGAAATTGTATTGTTGCAAAGTATATGGAGTCTCCTGTAAAGCACTTGTCAGTTG

ATGATTGCCATGGGATAGTAGCTGGCATTAGTGCTGATGATGTAACTCGAATTGCAAGATTTCTTGATCATTGGGGGATC
ATCAACTACTGTGCTGTTCCTCCTAAAGATGAGGCACCAAAAGATGGCACATATTTGTATGAGGACTCAAACAATGATAT
CTGTGTTCCGGTAGCTGGTTTGAAGTCTATTGACAGCTTAGTCCAGTTTGACAAGCCCAAATGTCGGCTGAAGGCAAAAG
ATATTTATCCTGAACTTGTGCGTGACTGCGATGACGACTCCGATTTTGACAACTCAATCCGAGAAGTCTTGTCCGAAATT
AAATGCAATTGTTGTTCTCGGCCTGTTTCACTGGCGTACTATCAGTCACAGAAGGAGATTGATATTCTATTGTGTTTGGA
TTGCTTCCACGAGGGTAGGTTTGTTGCTGGTCACTCTAGCTTGGATTTTGTCAAAGTGAGCTCTATGAAAGATTATGGAG
ATCTAGACGGAGATTCTTGGACTGACCAGGAAACCCTATTGCTTCTCGAGGGAATGCAACGCTACAAGGAAAACTGGAAT
CAAATTGCTGAACATGTTGGCACCAAGTCAAAAGCACAGTGCATCCTTCATTTTGTTCGTCTGCCTTTGGATGGTGCCTC
GTTAGACAATATTGAACTTCCAGGTGCATCTGGTCCCTCGAGTTCTAGCACAGGTGAAGATCCTAACAAATCTCATTCAA
TTTTGAATGGGAATCTTGCAGGACCAAGCACTGAAAACTTTCATCCTGATAGCAAATTTCCATTTGAGAACTGTGGAAAT
CCAGTAATGTCCCTGGTTGCTTTTCTGGCATCTTCTGTTGGACCGAGAGTTGCTGCAGCATGTGCCCATGCATCCTTGGC
AGCATTATCTAAGGATGATACTTTAGGTGCAAGACGAAACATGACTCAGATGGATGGATCCACAGCAAATAATGGGACCA
GTGTAGGAAGAATGCCTGGAAAGAATGGAAGTCCTCATGGAGATGTTGGAAATTCGCATCAGCTAAAAGATGAAAAGTCA
GGAGGACAAGGTCCACATGATACTGGAGGTGCTCCATTATCCACTGTAAGTGTAAGAGCTGCTGCTAAAGCTGGTCTTGC
AGCTGCAGCTATAAAATCAAAGCTTTTTGCTGACCATGAAGAACGTGAGATTCAACGGTTATCTGCAAACATAGTCAACC
ATCAGTTAAAGAGGTTAGAGCTGAAGCTGAAGCAGTTTGCAGAGGTGGAGACCTTGTTAATGAAAGAATGTGAACAATTG
GAGAGGACAAGGCAACGATTTACTGCAGAACGTTCTCGGATGATATCTCAACCTGGTTCCATTCGAGTATCCCGACCAAT
GGGGGTGTCTGGTGCTGGTGCAGCAGTGGTCAGTAACGCAGGAAATAGTAGGCAGCAACAAGTTGTTTCAGCTCCTCAAC
AACAAGGTTTTGTTTCTGGATATGGAAACCAACAACAGATGCATCCCCAAATGTCATTTCTGCAGCAACAAGGAATGTAT
GGATTTGGGCCTCGGCTGCCACTATCAGCGATACACCCTTCCTCTTCAACACCCGGTATGTTTAATGCATCAGCAAGTTC
CCAGCCTGCTCTCAGTCATTCAATGCTTAGACCTGTATCTGGGACAAAATCTGGTTTAGGTTGA

>CcSWI3D |Locus_ID=BC332_22454 |CDS_len=2832 |strand=+
ATGGAGGAGAAACGGAAGGACACCGGAACTCCACCACCGCCGTCCGCCGACACGCCGCCGATATCGGCTGACGCTCCGTC
AGTTGAACCACCTACGTCGCGTCGGAGAGGTGGCGGCCACAAACGAAAAGCAAGCGCCATTGGGAGTGGCGCTAGTTCTA
CTCCTCCCTCAACTTTATCGAAGCGTCAGAAACAATCAGCAGTACCTTTCCCACCGATCCACAATGGTCCGCTCACAAGA
GCTCGGCAGCAGCCAAACCACGCCGCAGCTGCCGCCGCCGCCGCAGCTGGTTCTCCGTCAGGTTTTGGTTTTACGATCGA
ATCGGAGGTGTTTCCGAAGGCCGAGGCAGGTGTAGAAGAGGCAGTGAGAGTTGAGAAAGAGTCGAATCAAGTGAAGGAGG
ATTTGGAGGCATTAGAGGCTGAAATTGAAGCTGAAATTGAGTCAATTGGATCCCGAGATCGTAATGTTCATATTGTGCCT
ACTCATGCAGGTTGGTTCTCTTGGACGAAGGTTCATCCTTTGGAGAAGCGAACAATGCCGTCATTCTTCAATGAGAAGTT
GCAAAGTAGGACTCCAGAAATATACATGGAGATACGGAATTGGATCATGAAAAAGTATCACACTAACCCCAATATTCAAA
TTGAGTTAAATGATTTGTCCGAGCTCTCAACAGGAGATTTGGACGTTAAACAGGAAGTGATGGAGTTTTTGGACTACTGG
GGTTTAATTAATTATCACCCTTTCCCACAAACCAATTCAGTTATGAACGTTGATATTGATGGGGACAAGGCAGCAAGGAC
AGATTCTTTGATTGATAAACTGTTTCGATTCGAATCAGATGAAACGTGGACCCCAGTTCTTCCAAGGTCTAGTGTAGCTA
CTCCTTCTGTGTCTTCGGGGTTCTTTCCAGAGTCAGCAATTGCTGAAGAACTAAAGTCCGAAGGGCCAGCTGTTGAGTAC
CATTGCAACTCTTGCTCAGCTGACTGCTCGAGAAAGCGGTATCATTGCCAAAAGGAGGCGGATTTTGACTTGTGTAGTGA
ATGCTTCAATAATGGGAAGTTTGGATCTGGGATGTCCCCTTCAGATTTCATTGTTATGGAGCCTGGTGAGGTTGGCAGTG
CAAGTGGTGGAAAGTGGACAGATCAGGAGACTCTTCTTCTCCTTGAGGCATTAGAGCTGTATAAGGAAAATTGGAATGAG
ATTGCGGAGCATGTGGCTACAAAGACTAAAGCTCAGTGTATTCTGCATTTCATTGAAATGCCAATAGAGGATAGGTTCCT
GGATACTGATGCTGAAAGTAACAAGCTTGTTAAAGAAAAAGAAGATGCAGTTTTGTCTAAAGATGATACATCAGCCAGTA
AAGATGCCCCAGAAACAACAGAAACCAAGGATGATGGAAATGACGATCAGCTTTCATCCACAGTGGAAACATCGAAACCA
GAGAATGTTAATGGGCCAATTCCTCAGGAGGAAGTTGGTGAGAACTGTGCACTCAATGCACTGAGGGACGCATTTACAGC
TGTTGATTTTTATCCTCCACCTGGCGAACGTGCTTCATTTGCTGAAGCTGGCAATCCTGTAATGGCATTGGCAGCATTCC
TGGTGAAACTGGTAGAAGCTAAAAAGGTTTCTGCCTCAGTGCGCAGCTCTTTGAAATCCATTTCTGGTAATCCTTCTGGT
GAGCAGCTAGCCTTAAGGCACTGTTTTATTTTGGAAGATCCACCAGATGATGGGAAGACATCATCTGATTCAGATAGACC
TGCTAATGGATCAGTTGATCCAGAACATAAGAAAGATGAGGATGACAATGTTGAAATGCAGGAGGAAAAGTTGACATCAG
TTATTGATGAGAAGAGTTTGTCAGTTGGACAAGATAAAGAGACCAAAGGTGAAACAAACATTGACAAAAAGTGCGAAGAG
CGGGATGGTGAAAACCATGAAGAGAAAAATGAGAAGGAACTTGAAGTAGCGACACATTTGGTTTCTACGAGTGACAAAAG
TCAGGAGAAATCTGATACTTCAAAACAATCTGGTGCAATTTCTACTGACAAAGAAGGGGAATCTGCATCACTTAAAGAGC
CAGATGATGCAGGCTTGGCAGTGGGACAGACACCAAGTACCCACAGCAGAATCAAATGTTTTAACGTCTAAACTAGAGGTC
CCACCTGGGTTCGAAAAGGAGTCAGTTGATGAAGCTTCAATGGCCATTCCTTCTAACTCTCCAGATACTCCGAAAGATGA
GGACATGATGCCCACTGTGCAAACAAAGGAGCCTGAGCAATCCATGAAATCGAACATTGTACTTGAAAATGGCAAAAATA
CAGGGACCGTAGAAGTCAAAGACTCTGTAGATGGGAGAAAAGATCCATTGAAGACCAAAAATGATCTGGATATTGATAAG
ATCAAACGTGCAGCAGTCACTGCTTTGTCAGCTGCTGCAGTCAAGGCGAAGTATCTTGCAGATCAAGAAGAGGACCAAAT
TCGGCTGCTTACTACGGCATTAATAGAGAAGCAGTTACACAAGCTGGAGAGCAAGATAACCTTCTTTCATGACATGGATA
ATGTGGTTATGAGGGTCAGAGAACTGTTGGAGAGGTCAAAACAGAGGCTTGTCAGCGAGCGTCAACAAATAATTAATGCA
AGACGTGCTGGGCCACAACCTGTACTTGCTAACCGGCCTGGAATGACTCTTGCTAATTCAGCACCTAGGCTTCCAACTGC
AATGAGCTCCCCGAGGATACCGAATTCAAGACCCATAATGGCAGGAGCTCCTACACCAAGCTCTTTTATGCCTACAACAG
TTTCAGGAAATTCAATGCAGCCTTCAAAGTAG

>CcRAP1 |Locus_ID=BC332_23251 |CDS_len=1497 |strand=+
ATGAATTTTGATTATATTTTCAACAATAATATTGCTGGAGATAATGAAGAGCCAAACAGAGAGAAGAAGAGAGGTAGAAA

AAGCTCGAAAGAGATTCAAGAAGAAATACACCAAACAAGTGTTATTAATCAGGCTGTAAGGCGACAAACCTGCACAATAT
GGACTGACGATCTTCATGCCAAATTCATGGAAGCTATACATCAACTTGGTGAAGGGAGATGTTACCCTAAGGAGATTCTT
GAGGTGATGAACGTTCCTGGTCTTACCCGTACCCAAATCTCTAGCCACCTTCAGAAATGTCGTAGCAACAGTTGGAGAGC
TCCACGAAAGCAAAAAGCTATTTGTGACTCATTAAGTCAGGCATCCTCAAGTGGTACTCAACAAAAGAATAGCTTTAAAA
ATTATGGAGCAATGCCTCGTCTACCAAATCTACAATGTAACCTAGATCAAATGGAAAGAGGCTCAGAGTTTTCATTTTCA
GTACCCAATATCAACAATATTTTTGCTAGAGGAAAGAGTTTGACTCAACAACAACTTTATCATCCACAGCCTCAGGTTCA
ACCTCAAGCTCAAAACAATGTTGGTGGTGGTCTGCAGCAACATGGACCATCATTTGGACTGCTGGGTTCACAAGGATTAC
AAGGCCCAACTATTGGAAACATTATTTATAAGCCTGGTCTAACATTTAACAATAGCGATCATTATACTCAAAGTGATTAC
AACTTGAATCTCAATGCTGCCCATGACACAACATATTCAAGTAGCGGAATAATGTCTGACACAAACATTGATGCGGCAAT
TAATTACTACAACTTGAAGGTCAATTTGGATAACGTGACAACATATTCAAGAAGTTCAATGATTTCTAATACTGGTGTTG
CAAATTCGACTATTAATGAACTGGGAGCAGCAAATGCAAATTTCCAGAAGAATATTGGTGAATCAAATGAACATGACGAC
GAGCTTGTTCAAGTTCATTTTGATGATCAGGTTCGGACGCTCAATTTTCTGACCGAGCTTAGACAGGATCAGTTAGCCAG
CATCAGCAGTAGTGGTAAATCCTCATACTTCGAGGACAAGTTTATCTTATATTCAGTTCGGATGCTCAGTTTCCCAATCG
AGCTTAGAAAGGATCAGTTAGCCAGCATCAGCAGAAGTGGTGAGTCAGTTGTTGCTAACCAAATCTATAAAAAGTGTCTG
ATCACTGTCCTTCATAAGGTCATGTTAGCAGATTTGATAGAGTTAGATATGGTGGATTTTGATCTTATTATTGGTATGGA
TTGTCTTCAATTCTATTATGCATCTATAGATTGTTGCACCTATGTGGTGAAGTTTCAGTTCCTAGATGAGCCAGTCTTTA
AGTGGTCAGAAGATCTCCTAGAGGTACCTCCCGATAGAGAGATAGAATTTGGTATTGACCTTCTTCCAGATACTCAACCC
ACATCCATTCCTCCTTATTATATGGCTCCGACCGAACTTAAGGATTTAAAGGAGTAG

>CcAPL1 |Locus_ID=BC332_07646 |CDS_len=1149 |strand=-
ATGTTCCATGCTAAGAAACCTTCGACTATGAATTCCCATGACAGACCCATGTGTGTTCAAGGAGACTCAGGTCTTGTCCT
CACCACTGATCCTAAACCTCGTCTTCGTTGGACCGTTGAGCTTCACGAGCGTTTTGTTGATGCTGTTACTCAGTTAGGTG
GACCTGATAAGGCAACACCTAAAACTATTATGAGAGTTATGGGTGTTAAGGGTTTAACCCTTTACCATCTCAAGAGCCAC
CTTCAGAAATTTAGACTGGGAAAGCAGCCTCACAAAGAATTCAATGATCACTCGGTGAAGGATGGTGATAGAGCAACCAG
TTTAGAACTCCAAAGAAATTCGGCATCCTCATCTGGAATGATCGGGCGTAATATGAATGAGATGCAAATGGAGGTACAAA
GAAGGCTGCATGAACAACTGGAGGTACAAAGACACCTTCAGTTGAGGATTGAGGCACAGGGAAAGTATATGCAGACTATA
TTGGAGAAAGCTTGTCAAACATTGGGGGGCGAGGAAAATATGGCGTTACCAGCAAGAACATTTAAAGGCATTGGAAATCA
GGGAGGACTAATACCGGACATTTCAGCTGCCTTTAAAGAGTTTGGAACCCCTCCTCTAAGTTTCTCCTCATTTCAGGACC
TGAATATTTGTGGAGGACACGACCAACAGCTCGAACTTCATGCTCAGAGTAGCATGGGTGAAAGGTCATCATCCTTCGAT
GGATTCATGAACCTAGGCAATACTACTGATCATAACTTGACTCTGGGAAAGAAGAGGCCTAGCCCTTATAGCAGCAATGG
GGGTAATAGCAATGGAAAGAGCCCCTTTATGTGGTCTGATGATTTCCGTCTCCAAGAATTAGGCGTAGCAACAGCAGCAG
CTTTAGGTCCTAATAATGAAGACGACCACCAGATTATTCAGATGGAAAGGAGTTGCAATCCCGAGCTTGATTCCGTGTCT
GACCTTTACGAGTCAAAGCCAATCCTGCAGGAGGAAAAGAAATGCGACGCCAAGCTGGAGAGGCCCTCCCCGAGAAGAGC
CGCCCAGATTTCGAGTTTGAGTGCCCAAGGTGGACGAAGCTCTGTTTTCGGTGAATTACTAAGTTGGCAAGATGGAAAGG
TCACCGACGGTGGAGAGAGGCGGGAGTAA

>CcAPL2 |Locus_ID=BC332_15958 |CDS_len=702 |strand=+
ATGTATGAGAATGATGGGGTGGTGATGACAAGAGATCCAAAGCCAAGATTGAGGTGGACTGCTGAATTACATGACCAATT
TGTGGATGCTGTAACTAAGCTTGGTGGTCCTGATAAAGCAACTCCCAAATCAGTACTGAGGTTAATGGGCTTGAAGGGTT
TGACACTATACCATTTGAAGAGTCATTTGCAGAAGTATAGACTTGGACAGCTGGCCAAGAAACAAAATGCAGCGGAGGCA
AACAAAGAGAACTGTGGGGACTCCGGACAATTTAGTTTGCATTCCTCAGGCCCCAGTACCTCTTCATTAAGTATGAACTT
TATGCAAGGAGAAGTCGTCCCAGTCGCAGATGCAGTAATGTGTCAGATTGAAGTTCAGAAAAGATTACAGGAGCAGCTTC
AGGTGCAACAGAAATTGCAAATGAGAATAGAGGCACAGGGCAAGTATTTGCAAGCAATATTAGACAAAGCTCAGAAGAAG
CTCCCCAACATGAACTCTCCTAGTGCACCAGAAGCAACAACAGCTCAACTCACTGCTTTCAGTTCACCTCTGTCAAATTT
ACTGGATTACACAGTACATGGAGAGAACCGGGATAACAACATAGAGCTGTTACCAACACCTAATCACATCGTTGAGAAAG
AACAAAAAAATGATATGAATTCCATTAATTTCGATTTGAACTCAAGAAGTAGCTTTGTATGA

>CcAPL3 |Locus_ID=BC332_00901 |CDS_len=456 |strand=-
ATGGATAGGATGTATAATGGTGGTGGAGATGGTTATGAAAATGGAGTGGTGATGACAAGGGACCCAAAGCCAAGATTGAG
GTGGACTGCTGATTTACATGATCGTTTTGTTGATGCTGTCACTAAGCTTGGTGGACCTGATAAAGCAACTCCCAAGTCCG
TACTAAGGTTAATGGGATTGAAGGGCTTGACACTCTATCATCTCAAGAGTCATTTGCAGAAGTATAGACTTGGACAGCAG
GCAAAGAAACAAAATGCAGCAGAACAAAACAGAGAGAATATTGCAGCGGAGTCCTTCGGACAATTCAGTTTGCATTCCTC
AGGACCGAGTATCACTTCATCAAGCATGAATGGTATGCAAGGAGAAGCTCCAATCAGTGAGCAACTGTGGTGTCAGTTTG
AAGTCCAGAAAAGATTACAGGAGCAGCTTGAGGTTCAACAGAAACTACAAATGAGA

>CcAPL4 |Locus_ID=BC332_31366 |CDS_len=876 |strand=-
ATGTATTCTGGTTTGGGTATGGAAAGAAATGGAGGAATGGGTGAATATCATAATCATCATTTTCATCATAATTTACAAAG
TTCAATTACTGGTGAAATGAACAATTTGGTAGGGGATGCTTGTCTGGTTTTAACTGCAGATCATAGGCCACGTCTCCGGT
GGACGGCTGAGCTACATGAAAGATTTGTTGATGCTGTTACTCAACTTGGTGGTCCTGATAAGGCGACACCGAAAACAATT
ATGAAGGCAATGGGGGTGAAAGGACTCACCTTATATCATTTGAAGTCACACCTGCAGAAATATCGCATAGGGAAGCAATC
TAAGGAGGCAGCGGAGAACTATAAAGACGAATCATGTATGGCAGAGAGTCAAGATACAAGTTCCACAGCGTCGGGTTCAT
CAAAAGTAGTTGCCCAGGATATAAATGACTGTGGATACCAAGTTACCGAGGCTTGCCGAGTACAGATGGAAGTCCAGCGA

AGACTACAAGAGCAGCTAGAGGTACAACACCATGTCCAGCTTCGTATCGAAGCACAGGGGAAGTACTTGCAAACGATACT
CGAGAAAGCATGTAAAGCTCTTAACTACACTGCCTTGGAATCTCCTGAACTAGACACTGCCAGGGAACAACTTTCTGAAT
TAGCAATCAAAGGCGCCAACGCCAACAACTGTGATGGGATTGTCGCGGTTTCTTCATTACCTGAAGTTGTTACAAGTTTC
GAGAACCAAAATGCTTCCGATATGCCTGCTACAAGCTTGGTTTCTCCCACGAGCATCGGTGTTCTAAAGAAGAGACCACG
ACCTCTAGCTAACGGGGATGCCTTGCCCGTGGAAGACAATATGACACCAGTGCAATGGATGATGACTAATTCCTGA

>CcAPL5 |Locus_ID=BC332_06463 |CDS_len=879 |strand=+
ATGTACTCAACATTGCCTATAAGGAGTTTAGGTATAGAGGAAAGTGGTGATTTTTTCAATCTACAAGGTTGTCTTTCAGT
TGATGGTACAAATTTGCCAGGTGATTCTTGTGTAGTTTTATCAACTGATCCTAAGCCACGTCTTAGATGGACTACTGAAC
TTCATGAACGTTTTGTTGATGCTGTTACTCAACTTGGTGGTCCTGATAAAGCAACGCCAAAAACAATTATGAGAACAATG
GGGGTGAAAGGTCTGACCCTATACCATTTGAAGTCACATCTCCAGAAATACCGCATGGGGAAGCAATCTGCTAAAGAGGG
AACTGAGAATTCTAAAGACGTATCATGTCCGGCAGAGAGTCAAGATACAGGTTCCTCTACATCAGGTTCATCAAGAGTTA
TTGCGCAAGATATAAATGAAGGCTTACAAGTAACTGAGGCTTTGCGAGTACAGATGGAAGTCCAGCGAAGACTACATGAG
CAGCTAGAGGTACAACGCCGTCTACAGTTTCGTATAGAGGCACAAGGAAAATATCTGCAATCAATTCTTGAAAAAGCTTG
TAAAGCTTTTAATAGCCAGTCCCTTGAATCGAATGGCCTAGAAATGAACGGAGAAGACCTCCCTGCATTAGCAACGAAGG
TTGCAAACGACTGTGAAGGGATCATCACTGTCCCTTCATTACCTGAGATTGGCTCGAGTATTGAGAACAGGAATGCTTGT
CACTTGCCTGCCAGACTTGGCGATTGCATGCTTGATGACTGCTTGCCATCAAATGGAATCGGTGCTCTAAAGAAGAGACC
GCGAGCTTTTACTAACGCCAATGGATTGCCTATGGAAAGCAATACAAGGGAGGTGGAATGGATGATGAGTAATATATGA

>CcAPL6 |Locus_ID=BC332_12550 |CDS_len=972 |strand=-
ATGTATCCAAGATTGATTCAGTCTGGACAAGAAGATGAGTATATACAACATCATGGGTGTGATGTTAATGTTGTTGGGAT
TCATCATCAAAGGGTAAATGGAGGAGACCCTTGTCTTGTTCTAACTTCAGATCCTAAACCTCGTCTCAGATGGACTGCTG
ATCTTCATGAACGATTTGTTGATGCTGTTACTCAACTTGGGGGTCCAAGTAAAGCTACCCCAAAGGCGATAATGCGGACA
ATGGGTGTCAAGGGACTAACTCTTTTCCACCTAAAGAGTCACCTTCAGAAATATCGACTAGGTAAGCAATCTCAGAAAGA
TCTTGATGAGGCTTCGAAAGATGGACTTACAGCTACGTATTCGTTAGAAAGCCCTTGTTCTGGTGGTACTCCTCAGCAAT
TGCCTGCATCGGACTTGAATGAAGGTTTTGAAGTCAAGGAGGCATTGAGAGCTCAAATGGAAGTGCAAAGTAAATTGCAC
CTGCAAGTTGAAGCCGAGAAACACTTGCAAATTCGTCAGGATGCTGAACAAAGATATATTACCATGCTGGAGAAGGCCTG
TAAAATGCTTGCTGATCAATTCATCGGTGATGTAGTTACTGAAAATCATCAAGAGACTTACCAAGGATTAGGAACAAAGA
CACAACTTAGTCCTTTATGTAATCCAAACGGATTGTGTCCCTCGGATTCTGCTGACCTTATTGGAGTCCATGGACCGGAA
GAAGTTTCCCCCAGAATCCATCCACAGCGCACGGATTGCTCCACTGAAAGCTGCTTAACTTCACATGAGAGCCCAGCTGG
ACTTCCTCTAGAAGGATCTTCACCTGGAGGGAAAAAACGAGGGCTAAGCGGAGATTCAACACATGCATCATATGTTTGGG
GTGAAGCTGATATGAGATCATCAGGTGTTCACGCGATACAGGTTAATTGCTTCGGGATTACTGGCTCTAACGTTCAAAAT
GTCTCGAATTAA

>CcDIV1 |Locus_ID=BC332_32995 |CDS_len=771 |strand=-
ATGTGGATAGAGTCATATTACCAGAACACCTCCTTAAATTCCTCAGCAAAAACCCTCTCTTTATTTTACATTCAAACCAA
AAAAAAAATAACCAAATCAACGATGATGTACACAACGAACAATCGGTGGATGATCGGCGACGCGGCCAACAATTCAACGG
CTATGATTCAATCAAATTCAACATGGACACGATTCGAAGACAAGCAATTCGAGCAAGCCCTAGTACTGTATTCTGAGAAC
GACGTTGAACGTTGGCAGAAAATAGCAAATCATGTACCTGGAAGAACAGTAGAAGATGTGATGATGCACTATGATACACT
TGTTCATGATGTATTTGAGATTGATTCTGGAAGAGTTGAACCACCGAGTTATCCTGATGATGATGGAGGATTTGGTGGTG
ATTGGGATGAATTGGATGGAGGTAAATTGAGTCAGATCTCCTTTGGTGGTGGAGCCAGTAAGAAGCAAGAAGTTGAGAGG
AAGAAAGGGACTCCATGGACTGAAGAAGAACATAGGTTATTCCTTATTGGGCTGGACAAATACGGGAAGGGTGACTGGAG
AAGCATTTCAAGAAATGTAGTGGTGACGAGGACACCGACACAGGTGGCCAGTCATGCTCAAAAATACTACCTTAGGCAGC
AGTCAATGAAGAAAGAACGGAAAAGATCGAGCATACATGACATTACCACCGCAGTGGATACGAAGATGGTGCCTCCTCAA
AACAGTCTTCAGAATCAAGGAGCGTATCAGAATTTCAACTTCCCCATGTAG

>CcDIV10-1 |Locus_ID=BC332_15633 |CDS_len=1197 |strand=-
ATGAGCACCGATCAGATATGCAATAGCACCTTCTGGACCAAGGAGGAGGAAAAAGTCTTTGAGACTACCCTAGCGATCTA
CTTTAAGGGTGGTGATCTATTGACGAAGATGGAAGAAGCGCTTCCTTGTAAATCGCGTGATGATATCATAAATCACTATA
AAATATTAATCGAGGATGTTGATGCCATCGAGTCTGGACGTGTTCCATTACCTAATTATCCAGAATTGCCAAGCCATTCC
AACCAGAAAAGCCGATTTCCAAAAACAAATCTAGCACGGCGAAAAGGGGTTGCTTGGACAGTAGAGGAACACAGGTCGTT
TCTCCGGGGGTTAGCTATATATGGGCGAGGTGACTGGAGAAGTATAGCCAGGAACTGTGTGATAACAAGAACATCAATGC
AAGTGGCTAGCCATGCCCAAAAATATTTCAATCGCCTCAAAGCCGTCAACAAAGAGAATAGAAGAATGAGCATTCACGAT
ATAACTATTGTGGATGCTGAAGTCGCTGGAACTTCCCAAGTACCATATACTGAGGGCATGATTTGGCCTGCTTGTGGAGG
TTCGCAAGCGGTGGCATGCACTAGCAATCAGAACATGTTACCTCCAGAAAGCACCAATGCTGAGCAGATGATAGCAGTTG
CTGGAGGAGAGTCCTCAGGTCATACTGCTGCATTTGGCAGTGGAATGAATAGTATGTCTGATGAGGATGATTTCATATTG
CATATTGATGACCTAATCGTGGATCCCGAGGACGCTAATGAATCTGAGATTCTTGTTGATGGTGGAAAGTCGCTATTACC
CAGCAAACAACCATGTACTGCTGGTAGTAGTGGAAGTAACAATCATCCTATCTTCAGAGTTGGAAGTGAACTGGGAGCAT
TAATCACTAAGCAAATGGACGAAGACTATGATTTCTTTTCCATCTTTGATGATGGGGATGCACCAATTTCTCATGCAATG
GTGTCCAATGCTGCTCATAGTGGGATGACAAGTTTTGCAGTGGCTAGCAACAGTGCTAACAATCCACCTCAGAACACGGT
GTCTGCTCATAGCGGAAGGACCAGCTTTGCAGCTGCTAGCACCGGTCCTAACAAGGCACCTCTGAACATGGTGTCTGATC
ATCCTCAATTACCTCACGTTGCCCCTTCATCTAACTGTGTTGTCGGAGAATGGCTCCAATTTAAATACTACCATTGA

>CcDIV10-2 |Locus_ID=BC332_15631 |CDS_len=1203 |strand=-
ATGAGCACCGATCGGACATGCAATAGCTCCTTCTGGACCAAGGAGGAGGACAAAATCTTTGAGACTACCCTAGCAATCTA
CTTTAAGGGTGGTGATCTATTGACGAAGATGGAAGAAGCACTTCCTTGGAAATCGAGTGATGATATCATAAATCACTATA
AAATATTAATCGAGGATGTTGAAGCCATCGAGTTTGGACGAGTTCCATTACCTAATTATCCAGAATTGCCAAGCCATTCA
AACCAGAAAAACAGATCTTCAAAAACAGATCTAGCATGGCGAAAAGGGGTTGCTTGGACAGTAGAGGAACACAGGTCATT
TCTCCGGGGGTTAGCTATATACGGACGAGGTGACTGGAGAAGTATATCCAGGCACTGTGTGATAACAAGAACATCAACGC
AAGTGGCTAGCCATGCCCAAAAGTACTTCAATCATCTCAAAGCCGTCAACAAAGAGAATAGAAGAATGAGCATACACGAT
GTAACTATTGTGGGTGCTGATATCGCTGGAACTTCCCAAGTACCATATACTGAGGGCATGATTTTGCCTGCTTGTGGAGG
TTCGCAAGTGGTGGCAAGCACTAGCAATCAGAGCTTGTTACCTCAAGAAAGCACCAATGCTGAGCAGATGTTAGCAGTTG
TTGGGGGAGAGTCCTCAGGTCATAGTGCTGCATTTGTCAGTGGAATGAATAGTATGGATCCTGATGAGGATGATGAGTTC
ATAAAACATATTGATGACCTAATCGTGGATCCAGAGGACGCTAATGAATCTGAGTTCCTTGTTGATGGTGGAAGGTCGCT
ATTAGCCAGCAAACAACCATGTACTGCTGGTAGTAGTGGAACTAACAATCATCCGATCTTTATAGTTGGGAGTGACCTGG
AAGCATTAATCACTAAGCAAATGAACGAAGACTATGATTTCACTTTCATTCTTGATGATGGGGAGGCACCAATCTCTCAT
GCAATGCTGTCCAATGCTTCTCATAGTGGAATGGCTAGTTTTGCAGTGGCTAGCAACAATGCTTACAATCCACCTCAGAA
CACGGTGTCTGCTCATAGCAGAATGACCAGCTTTGCAGCTGCTAGCATCGGTCCTAATAATGCACCTCAGAACATGGTTG
CTGCTCATCCTCAATCGCCTCATGTCGCCCCCTCATCTAACTGTGTTGTCGGAGAATGGCCCCAATTTAAATACTACAAT
TGA

>CcDIV11 |Locus_ID=BC332_16553 |CDS_len=381 |strand=-
ATGGTTCCAAATAAAACCATTGACGAGCTGAAACAACATTACCAATTGTTAGTGGATGATGTTACAGCCATTGAATATGG
CTATGTTCCAATCCCAAATTATACTGTAGAGGAAAATAGCTATGGCTATTCCAGAGTTTCGAGGGATGATTGGCAGTCCA
ATGGGAATGAGTTTGCAGAGACAGCCCAATGCCCAGAGAGAAGAAAAGGGACACCATGGACTAAAGAAGAGCACAGGTTA
TTTTTGCTTGGCTTAGAGAGGTATGGGAAAGGCGATTGGAGAAGCATTTCAAGGAACATTGTGATATCAAGGACGCCATC
TCAAGTGGCCAGTCATGCTCAAAAGTACTTTAACCGTTTGAAGAAAAACCAGCATCCATGA

>CcDIV13-1 |Locus_ID=BC332_23075 |CDS_len=1047 |strand=-
ATGGTGGCACATCGCGGTGAGCCACTAGAAATTGATAATTGGGAACTGGGGCTTCAACGCGATGCGGGCTTATCACGAAG
GTCCACTAGAATTGGACAATTGTCATTTTGGCATCCACCGCGACACGGAAATAGAAGAGATAGCCTAGAAATCACTGAAG
GAAGAGAAGAGGAAGAGCAAATAGATACAGTCTATGTTGATTTGGCCGCTGAAAGGAAAGTAGACATTAAAGGACCAAAA
GATGACACTAAGAGCACTTTCCATAGTAACACGGGTAGTGAGATTGAGCACTTGTCAGTAGGCTTGAGTGACTATGAAGG
TGAGGGTTTTGACTTCGATTATGAGAAGTACATTACTGATAATACATGTCTTGCTGCGGTTGATTCTGTTTACCAACCAG
ATATTTATGGTTGTTTTTGTGATATAATGAATCTCATCCGTTTTCTGAATCCTCTGCAAGAACTTACCCTTTTCATGCTT
GAGCCTTTCCTTGTATGTCAAAAAGAAATGAGAACCGACCGGACATGCAATAGCTCCTTCTGGACATTGAGGAGGATGGA
AGAAGAACTTCCAGGGAAATCGGGTGATGATATAATAAACTACTATAATATATTACTTGAAGATGTGGAAGCCATAGACT
CTGGACGTGTTCCAATACCTAATTATCCAGAACTGCAAAGCATTTCCAACCAGAATTCAGAAGCAGATGTAGAAAGGCAA
AGAGGGGCTTCTTGGACAGAATTGGAACACAGGTCATTTCTCAGGGGCTTAGATAAATATGGAAGAGGTGACTGGAAAAG
TATATCTAGGTACTGTGTGATAACAAGAACACCAACGCAGGTGGCTAGCCATTCCCAGAAGTATTTCAAGCACCTCAAAG
CCGTCCCCAAAAAGAATAGAAGAGCGAGCATTCTCGATATAACTAGTGCAGATGCTGAAGCCGCAGGAACATCACAAGCA
ATGCCAAGTACTAACAATGAGAGCATATTTCTTCGACAAAGCACCAATGCTGAGCAGATCATAGCAGTTGCTGGAGGAGT
GGCTTGA

>CcDIV13-2 |Locus_ID=BC332_23074 |CDS_len=1290 |strand=-
ATGTTGAAACTCATCCGTTTTCTAAATCCTCTGCAAGAACTTTCACTTTTCATGTTTGAGTCTTTCCTTGTAGATCCAAA
GAAAATGAGCACCGATCGGACATGCAATAGCTCCTTCTGGACTAAAGACGAGGATAAAGTCCTTGAGAATACCTTAGCAA
TCTACTCTGACGATAAAGATCTTTTTACAAAGATGGAAGAAGCACTTCCTGGGAAATCGCGTGATGATATCATAAATCAC
TATAATATATTAATTGAAGATGTGGAAGCCATCGATTCTGGACGTGTTCCATTACCTAATTATCTGGAATTGCAAAGCGA
TTCCAACCAGAATTCAAGAGCAGATGTAGAATGGCGAAGAGGGGCTTCTTGGACAGAATTGGAGCACAGGTCGTTTCTCC
GGGGCTTAGATAAATATGGAAGAGGTGACTGGAAAAGTATATCTAGGAACTGTGTGATAACAAGAACACCAACGCAGGTG
GCTAGCCATGCCCAAAAGTACTTCAAGCGCCTTCAAGCCGTTCCCAAGGAGAAGAGAAGAGCAAGCATTCTCAACATAAC
TAGTGCGGATGCTGAAGCCGCAGGAACTTCACAAGCAGTGCCAAGCACTAAGAATGAGATCATGCTGCCTCGAGAAAGCA
TCAATGCTGAGCAGACCATAGCAGTTGCTGAAGGAGAGTCCTCAGGCCATGATGCTGTTTTTGTCAATGGAATGGATAGT
CTGAATCCTGATAGGAATGGTGAGTTCATATTCAGCATTGATGACATAATCATGGAGGAGGAGGACGTAAATGAAACTGG
ATTTCTTGTTGATGCTGGAAGATCCCTATTACCTAGAGAACAGCCATGTGCAGCTGCTAGCACTGGAGCGTACGGTCATC
CTATCACTAGAATTGGGAGCGACCTTGAAGCGTTACTCACTGAACCCAGGGAAGAAGACAATGATATCAATTCCATATTT
GTTGTTAGGAAAGCGCCAACTTCTCATGAAGTGTTGCTCAATGCTGCTCATAGCGGTATATCCAGTTACGCAGCTGCTAG
CTTCGGTGCTGAAAATGCACCTGAGAGCAATGTGGCTGCTTATAGCGGAATGTCCAGTTATGCAGATGCAAGCTCCGATG
CTGTCAATGCACCTGATAACATGGTGGCTGCTCATAGCGGAATGACCAATTTTGCAGTCACTAGCAGATTTGCTTCTAAT
GCACCTCAGGACACGGTGGCTGCACATGCTTCTCAGTTGCCTCCCTTCTCCCTCTCATCTAACTTTGATGTTGGAGATGC
CCCAATGTAA

>CcDIV14 |Locus_ID=BC332_14807 |CDS_len=657 |strand=+

ATGGAGAATTCTCCAGAGAACAATGATGTTTTCATTGTTTCTGATGATGATGGTCATGATCAACAACATCACCTAGTGCA
ACAGGAAGATAATCATCGTCCATGTCGTCGTCGGGGAACTCCATGGACTAAAAACGAACACCAGTTGTTTCTTATGGGAC
TTAACAGATTTAAAAGAGGGGATTGGAAGAGCATTTCAAGATACTATGTTGTTTCAAAGACACCGACACAAGTGGCCAGT
CATGCTCAAAAGTACTTCTCTCGTCAGGATAGTAAGACTCCGGTAGAGCGTCGTCGCCCCAGCATCAATGACATTCAAAC
TGTCAGTCTTAATCTCAAAACAACGTCAATAACAACTATGGGAGCTACTTATCTAATCAATAATAACAGATTTGCTTACA
ACAATAATCATCAAGCCGCTTCTTCATCAATACATTTTCACTATCCTAGTTACACCTTTAGTGGTCCAACATTTAGCAAT
AACTTCAATGAAAATTTTGAAATATCTCATTTGGGTGAAACATCTGGCAGCAACTCGTCTGTTATGGGGAAAAACAACAA
CAACAACAAATTCAACAATCTAAATAGGCCAGTGTCTCCTCGCCCATTTCTTTCGACATACTTGTCTTCAATTAGGAGGA
ATGACCCCCAAATCTAA

>CcDIV2 |Locus_ID=BC332_07426 |CDS_len=879 |strand=+
ATGGAAACTTTATTTTCTAGTAATTCGTTTTTACCAAATTCAAGTTGGATGAGCAAGAGCATAGAATGGACTAAAGAAGA
GAACAAGCAGTTTGAAAGTGCTATTGCGATATATGACGAGAATACTCCTAATAGATGGTTCAAGGTGGCAGCTCACATCC
CTGGAAAGACAGTTATTGATGTAATGAATCAATACAAGGAACTTGTTGCAGATGTTTGTGACATTGAAGCCGGATTGGTC
CCAAATCCTGGTTATTTTGGCTCTTCGATTACTCTAGAATTTGTCGATCATTGTGGATTACAAACATTTAGAAAAAGAGG
CAGCAGATCTTCTGATCAAGAAAGAAAGAAAGGTGTACCATGGACAGAAGAAGAGCACAGGAGATTCCTGATGGGACTTG
AGAAGTATGGAAAAGGGGATTGGAGGAACATATCCAAGAAAATGGTAATTTCAAGAACACCAACTCAAGTGGCTAGTCAT
GCTCAGAAATACTATCAGAGACAGATTTCAGGAGGCAAAGATAAGAGGAGACCTAGTATTCATGACATTACCACTGTCCA
TTTCACCACAGACGCCACGTCCCAAAACAACCTAGCGTACAACATTAATTCTTTGTCTAAAGAGAAGTTCTATGCAACCA
GCTCCTCTTTCCAGAAATTCACAACCACAACAGATATAGTGAACTGCTGGAATGCCAGCTTATTAAGTGAACACGTAGAT
CTGATGGATTTTGGATCATTGTATGGCAGTTCTCCGTTTGTCGCATACCCATACAAGATTGCTTCACAGTATTGGGATGA
ACATGGAGCAAACAGTATTACTTTAAGTGGTAATCCACAGCTCCAGCTCCAATCCACAAGATATCAAATATGGGATTGA

>CcDIV3 |Locus_ID=BC332_13887 |CDS_len=729 |strand=-
ATGGAAACTTTATGTCCTACTTCTTTTGTACCAAATTCAAATTGGACTATGCAACAGAGCAAGAGTACTACAGAATGGAC
TAAAGAAGAGAACAAGAAATTTGAAAGTGCTCTTGCAATATACGACGAGACGACTCCAAATCGATGGTCTAAAGTAGCAG
AAATTATCCCTGGGAAATCAGTTCTTGATGTGATGATGCAGTACAAAGAATTAGTTGCAGATGTTAGTGACATTGAGGCT
GGTTTGGTCCCAAATCCGAGGCGTTTCGTGTCTTCTTTCGCGTTGGAATTTGCAGATCATCGCAAGATTCATACGTTTAG
TAAGAGAGGCAAGTCTTATGATCAAGAAAGAAAGAAAGGTGTACCATGGACAGAGGAGGAACACAGGCGATTCCTAATGG
GGCTAGAGAAGTATGGCAAAGGAGATTGGAGGAACATATCGAGGAATTTCGTGATCTCTAAAACTCCAACACAAGTGGCT
AGTCATGCTCAGAAGTACTACTTAAGACAACTTTCAGGAGAGAAAGAGAGAAAGAGACCTAGCATCCACGACATCACCAC
CGTCAATCTCACAAACAACGAGTTGCTCAACAACAACAACAAATTGATGTGTGAAGTGAAGTCTTATATGTCACCATTGC
AGAGATCAACAAGCCCAGCAGACGTACTAATCGGCTGCTGGAACAACTCATTTGATGGAGACCTTATGGCATTTGGTCAA
TTTGTATGA

>CcDIV4 |Locus_ID=BC332_23536 |CDS_len=882 |strand=-
ATGAATAGTGAAATGGGATTTCTATCCCCTGCAACATACATCAAGAACTCAAGTTGGTTATTTGAGGAAAACAAAGGAAC
AAGATGGACACCTGAAGAGAACAAAGTGTTTGAAGAAGCGCTTGCGTTGTTCGATAAGGATACACCGGATAAATGGTATA
ATGTAGCTGCCATGATTCCAGGGAAGACAGTGAATGATGTGATCAAACAGTATAGAGAATTGGTAGATGATATTAGCGAT
ATTGAGGCAGGACTAATACCTGTACCTGGCTATACAACTAGTAATTCTTTTACATTAGAGTGGGTTAATAATCAAGATGG
TTTTCATGGATTCAAGCAATTTTATGGTCCTTCTGGGAAGCGTGGCTCGTCAAATCGATCTTCTGAACATGAGAGGAAGA
AAGGTGTGCCATGGACTGAGGAAGAGCACAGGCAATTTCTGTTGGGGCTGAAAAAGTATGGAAAAGGAGACTGGAGAAAT
ATTTCGCGGAATTTTGTGACCACAAGAACTCCAACTCAGGTAGCTAGCCATGCTCAGAAATACTTTATTAGGCAGCTTTC
CGGTGGAAAAGATAAAAGGAGATCAAGCATACATGATATCACAGTTGTCAATCTTACGGAATCTAAGTCGACTTCCCCTG
AGGATCATAAATCTGCCCCCACTCCACAAGCGCAACAGCATTCACAAATGAATGGTATGGTAAAAACACTATGTGAGTAT
AATTCACCAAATCAAGAATCAGACATAACATTCAACTCATCAAGTGGTGGTGGTCTGATTACCATGCCTTTTCGAGGGGC
TCCATCTTATGAACTCGAGCATGGAATGCATAACATTGCTCTACAAGGTCTTCAGTTTGGTAACTACAATACTATGTGTT
GA

>CcDIV5 |Locus_ID=BC332_17375 |CDS_len=921 |strand=-
ATGGAGATTCTATCACCAGCTCCTTATCTTGCAAATCCAAGCTGGTTTCTTGATGAGGGAAAGAGTACTAAATGGACTCC
AGCTGAGAACAAGGCTTTTGAAAATGCTCTTGCTTTGTTTGATAAAGATACACCAGATAGATGGCAAAGAGTTGCAGAAA
TGGTACCAGGGAAAAGTGTTGGTGATGTGATAAGGCAATATAAGGAATTAGAAGATGATGTGAGTAGAATTGAAGCTGGA
TTAATTACTATTCCTGGTTATAGTACTTCACCTTTTACATTAGAATGGGGGAATAGTCATAGTTTTGATGGGTATAAGCA
AACTTATGTTGTTGGTGGCAAAAGGCCTTTGTCTAGTAGGCCTCCAGAACAAGAGAGGAAGAAGGGTATACCATGGACAG
AAGAAGAACATAAGTTGTTTCTAATGGGGCTTAAAAAGTATGGAAAGGGGGATTGGAGGAACATCTCGCGCAACTTTGTT
GTTACTAGAACTCCAACTCAAGTGGCTAGCCATGCTCAGAAGTACTTTATTAGACAACTTTCTGGTGGCAAAGATAAAAG
ACGTGCGAGTATTCATGACATTACAACAATAAATCTCCACGATAATCAAACGTCTTCACCAGATTACCAGAAACCAACTT
CACCTGATCAACCAGCTGTGATTTCGCAGCAGCCAAAATCGGCTGCCAATGCCGTGCACAAAATGCCATTTCAGTGGAAT
CAGGCCAATAATGGGACAGCCATGGGCTTCAATTCTGCACAAGGAAGTTTGTTTATGTCCCCTCTTTATGGAGTAAACTC
CTATGGGAACAATAAAATGCAAAGCCAAAGCCTACAAAGAGGTGCTTTGCATGAACCTTACTTTGGATCTCAAAGTATGA
ATTTTCAGCTGCAATCAGCACATCAGTACCATCAAGCGTAA

>CcDIV6-1 |Locus_ID=BC332_28756 |CDS_len=1086 |strand=+
ATGAGCCTCGACCGAACATGCAAGAGGTCCTCCTGGACTAAGGAGGAAAACAAAGCATTTGAAAATGCCTTGGCTGATTA
TTCTGGAGATAGAGATCTAATTCAGCAGATTGCTGCTGCAGTTCCTGGGAAATCTCTTGAAGAAGTTATCGAACATTATC
TTGTATTAGTTGAAGATATTAAAGATATTGAGTCTGGTATAGTTCCGCTACCTAAATATCGGAGAATGCAAAGTTCTACC
GAACGGCGAAAAGGGATTCCTTGGACAGCAGAGGAACACAGGTTGTTTCTCCAGGGGTTGGCGAAATATGGAAAGGGTGA
TTGGCGGGCTATATCGAGGAACTTTGTGTTATCTAGAACACCAACACAAGTGGCTAGCCATGCCCAGAAGTTCTACACTC
GATTAAATGACAAAAACAACCCGAAGAAGAGAAGAAGTATTCATGATGTCACTAGTGCTGCTAACATTACTGAACCTTCA
CAAGGACCTTGTGGAGGACGATTACAGTGGCCAATCACCAACTATGTCACTGATGCTTTCAACACAGGGATGCTATCTAT
ACCAGAGCCAGTCACAACCCGCATAAGTACTGATGCTATTGAAGGAGCATCAGCTGCTGACCTGGAGAAAATCCCTCTTG
GTGCTGCTGTTGGTAATGAGTTGAATAGTTCATTTCCAGGCATGGTTGAGTTCGTCCAAAGTATAGAAGGCCATATCATT
GTACCGGCAGAGGTGCCCTCTGGAGTGTGTCATGGGGTTGACACTAAGACATCTCCATCGCATAGCTTGCAACCGTCAAT
TGCTGTTGGCTCTGGAATGTACATTCCTTCATTCAATAATGTTAGGTATGATTTGACAGCGCTAATGAGCAAGCAGTTGT
TTGGATCCAGTCAAGTAGGTCCTACAGTTAACTCTGCAAGTTTGCCATTACCAACTATTGATCGTATTGGAGTCCAGGAT
TGTATAGCTTCACCATCCATTCCTGGAAGTAGTAGCGGTGGAACTTATCCCTGTTGGAATCCTAGCAACATTGATGACAA
TACCTTTGATTTGGTAAACCTATTCCCAGATGGTCTAGGCAAATAA

>CcDIV6-2 |Locus_ID=BC332_28750 |CDS_len=579 |strand=+
ATGAGCCTTGACCGAACATACAAGAGCTCCACCTTGACTAAGGAGGAAGACAAAGCATTTGAAAATGCCTTGGCTGATTA
TTCTGGAGATGGAGATCTAATTCAGCAGATTGCTGCTGCAGTTCCTGGGAATCTCTTGCAGAAGTTTTCTAACATTATCT
TGTTGTTTCTCCAGGGGTTGGAAAAATATGGAAAGGGTGATTGGCGGGATATATCGAGGAGCTTTGGGTTATCTAGAACA
CCAACACAGGTGGCAAGCCATGCCCAGAAGTTCTACACTAGATTAAATGACAATAACAACCCGAAGAAGAGAAGAAGCAT
TCATGATGTCACTAGTGCTGCAGACATTATTGAACCTTCACAAGGACCTTGTGGAGGACAATTGCAGCGGCCAATCACCA
ACTATGTCACTGATGCTTTCGACACAGGGATGCTATCTATACCAGAGCCAGTCACAACCCGCATAAGTACTGATGCTATT
GAAGGAGCATCAGCTGATGACCTGGAGAAAATCCCTCTTGGTGCTGCTGTTGGTAATGAGTTGAATAGTTCATTTCCAGG
CATGGTTGAGTATGTTTAA

>CcDIV7 |Locus_ID=BC332_14309 |CDS_len=570 |strand=+
ATGTCGATATGGAGTCGAAACGACGATAAATTATTCGAAAACGCGTTAGTTTTATACCCCGAAAATATCGCCGATCGGTT
TCAATTAATCGCCGATTATGTACCTGGAAAATCAATCGATGATATTATGGCTCATTATGATGACCTTGTTCATGATGTAT
TTGAAATTGATTCGGGTCGGATCGAATTTCCGGGTTATACCGATGATGATTCGGGTTTGGAAGGTCATTCGGGTCGGGTT
GAATTAGAAGGTGATTCGGGTCGGGTTTCGGCTGAAAGGAAAAAGGGGACTCCATGGACTGAAGAAGAACACCGGTTATT
CCTTATCGGGCTGGAAAAATACGGGAAGGGCGACTGGAGAAGCATTTCAAGAAACGTGGTTGTATCAAGGACGCCAACAC
AAGTAGCTAGTCATGCGCAAAAATACTTCATTAGACAACAAGCAATGAAGAAAGAACGAAAAAGGTCCAGCATCCACGAT
ATTACAACTGCAGTGGATACCAACACAGTGCCTCCTCAAAGTAGCTTACAAAACCAAAGAGGCACTCAAAACTTCAACTT
CGCACAGTAG

>CcDIV8 |Locus_ID=BC332_25861 |CDS_len=849 |strand=-
ATGAATAGTGAAATGGAACTTTTTTCTCCTCCTCAAGCAACATATCTTAGAAACTCAAATTGGTTATTTGAGGAAAACAA
GGGAACAAAATGGACTCCTGAAGAAAACAAGATGTTTGAAGATGCGCTTGCGTTATTTGACAAGGAGACTCCGGATAGAT
GGTACTATGTAGCATCCATGATCCCTGGAAAGACGGTGGACGATGTGATGAAACAGTATAGAGAATTGTTAGAAGATATT
ACTGATATAGAGGCAGGATTAATACCGATACCTGGCTACACAACTAGTAGTAATTCATTCACATTGGAGTGGATCAATCA
AGATCACGAAGAAGGCATCTTCCATGACTTTAACAAGCAATTCTATGGTCCATCTGGCAAGCGAAGCACATCAAATCGAT
ATTCTGAACACGAAAGGAAAAAGGGAGTCCCATGGACTGAGGAAGAACACAGGCAATTTCTGTTGGGGCTGAAAAAGTAC
GGAAAAGGTGATTGGAGAAATATCTCTCGCAATTTCGTGACCACAAGAACTCCAACTCAAGTAGCTAGTCATGCTCAGAA
GTACTTCATCAGGCAGCTTTCAGGGGGTAAGGATAAGAGAAGATCAAGCATACATGACATTACCACTGTCAATCTTACAG
AAATGAAATCTCCTTCCCCCGAGAATCATATATCTTCGAGTTCTCCGGACAAATCTGTTCCTTCTCCACAAAAAGCCTTA
TGTGAGTACAATTTTCCAAATCAAGAAGCAGTTATAGGATTCAAGACATATGGACTAAAGTTGCATGAACATGGCATGCA
TAGTGTTATTTTGCAGGGACTTCAGTTTGGTCAGTACAATACTATGTGA

>CcDIV9 |Locus_ID=BC332_09843 |CDS_len=873 |strand=-
ATGTCACCTTCAGATTGGAGCAGAGAAGAAGAAAAGGCTTTTGAAAATGCCATTGCTATGCACTGGGTTGAAAATTGTGA
ACAACAATGGGAGAAATTTGGTTTAATGGTCCCTACAAAAACCATTGATGAGCTGAAGCAACATTACCAGTTACTTGTTG
ATGATGTTACAGCAATTGAAGCAGGGCAAGTTCCAATACCGAATTACAAAGGAGAAGAAGCTTCTTCTTCTTCGACCAAA
GAGGGAAATCTAGGGTATTCTGCAGGTTCAGTAGACCGCCGGTCTAATTGTGGTTATTCAAATGGATTTTCCGGGACGAA
TCATGACCCGATTGGCCATGGCGGAAAAGGGAATTCTAGGTCTGAACAAGAGAGACGAAAGGGGATTCCATGGACTGAAG
AAGAACATAGGTTATTCTTGCTAGGTTTAGAGAAATTTGGGAAAGGAGATTGGAGAAGTATATCGAGGAATTTTGTGATA
TCGAGAACCCCAACACAAGTAGCTAGTCATGCTCAGAAGTATTTCATACGTTTGAATTCCATGAATAGAGATAGACGAAG
GTCGAGTATACATGACATTACAAGTATTAACAATGGAGACGTATCTACTCACCAAGCGCCAATTACAGGCCAACAAGTGA
ATACAAATCCATCAAATGCAGCAGCACTTGGACCATCTGTTAAGCACAGAACTCAGCCAAATATGCATGGTATGGGTATG
TACGGTGCTCCCATGGGTCATCCCGTCGCAGCACCGTCGAGTCACATGGCGTCAGCTGTTGGAACTCCGGTTATGATGCC
TCATGGACATCCTCCTTATGTACTTCCAGTTGCATATCCCATACCACCGCCACCACCACCCATGCACCAGTGA

```
>CcEFM1 |Locus_ID=BC332_24443 |CDS_len=1323 |strand=+
ATGAATATGACATCATCAAATTCAGAACTAAGCCTTGAATGCAAACCACATCAAAGCTACTCTATGCTTTTGAAATCATT
TGGGGAGAAAATTGATCAAACACAAAATCTTGAAGAATTCTTAGCTAGACTTGAAGAAGAAAGGGTTAAGATTGATGCTT
TCAAACGTGAACTTCCCCTTTGCATGCAACTTTTAACCAATGCTATGGAGGCTTCAAGGCAACAACTTCAATCCCATAGG
GAAAATAATCATATTGGACACAGACCAATTCTTGAGGAATTCATTCCACTTAAGAATAACAATAATGCAAGTGTTGAATT
AGGAGCAGAAAAAAATAGTTGCAAATAACACATCTACTATTGTTGATAACAAGGCAAATTGGATGACTTCTGCTCAATTAT
GGAGTCCACCAAGTGATCATCAAGAAACAAAGCAAATACAAACATCCAACAATACTTCTAACAAAGAAAATGATCATCAT
ATAGGCTTTTCTATTAGCCCAAAATTAGCCTTGGACAACAATTCTTCATGTCCAAGTCCAACAACACTAGCACTTGCTTC
ATCTAACAAACAAAATGAGTCAACAATAGTGGAAGATCATAACAAGAATTTGGAAGGAGTGAATATTCAACAAGATTCTC
AAAGCATTGGAGGTGGTACCAACAGCACAAGTACAAGTACTCAACAACAACAGCCACATAGAAAGGCAAGAAGATGTTGG
TCACCTGATTTGCATAGGCGTTTTGTTAATGCTCTTCAAATGCTAGGTGGTTCTCAAGTGGCCACACCAAAGCAAATTAG
AGAATTAATGAAGGTTGATGGATTGACCAATGATGAAGTTAAAAGCCATTTGCAGAAGTACAGACTTCACACACGAAGAC
CAAGTCCAAGTCCACAAGCAACAACAGCACCACCACACCTAGTGGTTTTAGGTGGCATATGGGTCCCACCAGAGTACGCG
GCTGCCGCAGCCACACATGGTGGTGCACCACCTGCAGCGACATTTTATGGACCACATTCAACTAGCCATGCACCATCACC
ACATTACATTGCGGCCCCACAAGCGGTCACACAAGAGTTCTACAGTACGCCACAACCGTTACAATCGCTCCACCACCTCC
AACACCACCAACAAATGTACCATCATGGTAGTCACATGTACAAGCCACCTTCAAAATCACATTCTCATAGCTCACCTGAA
TCCGATGCTCGAGGGACCGGTCAACACGGTGATCGATCATCGGAGAGTATCGAAGATGGAAAATCGGAGAGTTGTAGTAG
TGAAAATGGTGGAGAAAGGAAGGTTGTGGTACACAAATTCTAG

>CcEFM2 |Locus_ID=BC332_27315 |CDS_len=1209 |strand=+
ATGATGATCAACAACAATAATTTCAATGAGAAAATGCAAAGATGTCAACAATATATTGATGCTCTTGAAGAAGAACGTAG
AAAAATTCAAGTTTTTTCTAGAGAACTTCCTCTTTGTCTTGAACTTGTCACTCAAGCTATTGAAACATACAAGCAACAAT
TATCTGGGACAACAACAGAGTACAACCTTAATGCACAATCTGATGAATGTACTGATGATGATGAACATACATCTAGTGAT
GTTCCTATTTTGGAAGAATTTATTCCATTAAAAAGGACATTTTCTCATGAATATGAAGATCAAGAAGATGCTCATGATGA
GGAAAATGAATCTCATAAATCAAAAAATAATAATAGTAATAATTCTACTATTTCTAAGGACAACAAGAGCAACACAAAGT
CTGATTGGCTTAGATCTGTTCAGCTTTGGAATCAAACTTCAGATCCCACTCTAAAAGAGGTAATTAATTTTTACTATTTT
CTTACTTTCAATTTGTTTCTCTTTTTTCTTTTCTTTTTTATTTTTAAAATCTCTCGTAATATTTTTTTTTGTGATTATCC
AATTTTAGGTATATATTTGTCCATGTATGTTATCATTCAATTAAGTCTAGCTTTGGTTCGTTTAGAAGAAAATAAAGAAG
AGAAAGATGGACAAAGAAAACAAAGAAGGTGTTGGTCCCCAGAGTTGCATAGAAGATTTTTACAAGCCTTACAACAACTT
GGTGGCTCTCATGTTGCTACACCAAAACAAATTAGGGAGTTAATGAAGGTGGATGGACTTACTAATGATGAAGTTAAAAG
TCATTTACAGAAATATCGGTTACACACGAGAAGACCTAGTCCTTCATCAATTCACAACAACAACAACCAACAACCACCAC
AATTCGTGGTAGTCGGAGGAATATGGGTCCCACCACCAGACTACGCCTCTATGGCCGCTGTAGCACCAGCGGCCTCAGGC
GAAGCATCAGGAGTCGCAAACTCCAATGGAATCTACGCACCCATTGCTACCCATGCAAAGGGTCCCCTACATGATCATGC
ATCATTAGGGGGAACCCTAAAACAACTAAGGCAACAACACAACGAGAAGCCATCGCGCTCCTCTGAGCGCGATGGTAGCC
ACAGTCACAGTGAAGGTGGTGGCGTACATTCTAGCTCTCCGGCTACGTCGTCTTCTACACATACTACTACTGCTTCACCA
GCTTACTAA

>CcEFM3 |Locus_ID=BC332_22367 |CDS_len=1224 |strand=-
ATGGGTTCGATCCCACCTGAATTGAGCTTAGATTGTAGACCAAGTTTTGTTCCTGAATCTGTTACTGATTTTCTTAGAAA
AGTTTCTTTGATCCGTCATATACCTGATAAACTAGCACAGATTGATGATTATATATCCAGACTTGATGATGAAATGAGGA
AAATTGAAGCCTTTAAACGCGAGCTTCCTCTTTCTGTTCTTCTTGTTAAAGACGCTATTGGAACTTTGAGAGAAGAATCA
ATGCAATATAGAAAATCAAGAAAAGAACCAGTATTGGAAGAGTTTATTCCACTGAAGAAGAGTTTGGATGAAGATACTAA
AGCTGAAATCACGGCTAAAGATAAGGACAGTAGAGAAAAGATGAGTTGGATGAGCTCTGTGCAGCTCTGGAACAGTGAAT
CTCACTGTGAAAAGACCGATGAAGTGAATAATAAGCAGCCATCTAAATCAGAATTGAAAGTGGATACTCGGAAATCCCTT
GAAGAAGAAGGGCATAGTTCAATAACTGAAGATCCATTCCGGTCGTATAAAACCATGAATGTGGGAAAGGCATTTTCACC
TTTCAAGGGTTTTTCAGTGGCAGCAGTTAGGAAGGACAATAAAGATGAGCTACCAGGAGTAACAGGATTATCGCTTCATA
CTCCTGGAATTGCGAAGCTGAGGGAGGATACTGTGACTAGTGGTTTGAATTCAAAACACAATGGTAGCAGAGGTGGATTA
TCTAGTGCAGCAGGTTGCCAATCAAATATAAGGACTGGATCACTGGCTCAGCAGCAGTCTGCTAGAAAGCAAAGAAGATG
CTGGTCACCAGAACTGCATAGGCGATTTGTCGATGCACTCCAACAACTTGGGGGTTCACAAGTTGCAACTCCTAAGCAGA
TTAGAGAGCTCATGCAAGTGGATGGCCTAACCAATGATGAAGTAAAGAGTCATTTGCAGAAATATCGGCTTCATACACGA
AGGGTTCCAAATTCACAGACCCAACCAGCAAATAAGTCTGGCGTGGCTCTGGGAAATTTATGGATGTCCCAAGGCCAATA
CGTGGAATCCTCAAAGCGCAGTAGTTCTCAATCTGGTTCCCCTCCCGGTCCTCTACACTTGGCTGTGAGTTGTGGCGGTA
CTTCCACGACTGTGGGTGATAGCATGGAAGAAGAAGATGATTTAAAATCTGAGAATCACAGCTGGAAAAACCATGTTCAC
ACCTCTGGAAAGATTGATGTATAG

>CcETC3 |Locus_ID=BC332_24253 |CDS_len=261 |strand=+
ATGCAAGGTAAGGCTGCATACGATACACATCTTTGTGGTGTGCCCTTTCCCGTACAGAAGGAGAGCCTTGGAGTAACCGC
ATTTGAGGCCAATAATGAAGAAACTTCGAAGCTTGAATTTTCAGAAGACGAGGAATTCCTCGTTACTAAAATGTTCAACT
TAGTTGGTGAGAGGTGGTCATTAATTGCTGGAAGAATTCCAGGAAGAACTGCAGAGGAAATTGAGAAGTACTGGAACTCA
AGAAATTCCACCAGCCAGTAA
```

>CcGLK1-1 |Locus_ID=BC332_34438 |CDS_len=1344 |strand=-
ATGCTAGCTGTGTCACCTTTGAGTAACACAACGGCAAGAGATGATAACACGATGGAGAGTTTTGCGATTGGAGGAGGAGT
TGATTTCCCTGATTTCGTTGGGGAGAATTTACTTGACAGCATTGATTTTGATGACCTATTTGTTGGAATCAACGATGGAG
AGGTACTGCCAGATTTGGAAATGGACACTGAAATACTTGCAGAATTTTCAGTTAGTAGTGGCGATGAATCCGATGTGAAT
AACTATAGCAGTAGTAATAAAACTACGTTTATTTCTACTGCTACAAAAAATGTAGAAAGAAAAGATGAAACGGAAAGAGC
AGCCTCGGATGTAGGTTCGGGTTTAACGAGCTTAAATCAAGGAGAAGAAATTGTTAGTACTCAAAAAAGTGAAGAATCTA
CACTACAAGTGAAACAGAATATCACTCCAAAAGAAAGTGATAAAGGCAAAAAGTCATCCAAGAATAATCTTCCAGGCAAG
AGGAAAGTAAAGGTGGACTGGACGCCAGAATTGCACAGAAGATTTGTACAAGCAGTAGAGCAGCTAGGTGTAGATAAGGC
AGTCCCATCAAGAATTTTGGAGATCATGGGAATCGACTGTCTTACTCGTCATAATATTGCCAGTCATCTTCAAAAATATC
GATCCCATAGGAAACATTTGCTTGCAAGAGAAGCTGAGGCCGCGAGTTGGAGCCAGAGAAGGCAACTTTACGGTGGTGCA
GCCGTGGTAGGAGGGGGAGGAGGAAAGAGAGATATGACCCCGTGGCCTGCACCAACTATTGGCTTTCCTCCTCCTCCTAC
GATGGCGGCTCCAATGCCTCATCATTTTAGACCCCTACATGTATGGGGTCATCCATCTGTCGACCAATCATATATGCACA
TGTGGCCTAAACATCTAGCGCCTTCGCCTTCTCCACAACATCCATCACCAGCATGGGCTCCTCCTCCTCATCTTCATCCT
CCGCCACCTTCAGATCCTTCTTTTTGGCATCCACACCATCAACGAGTACCAAATCCTCTTACACCAGGAACTCCTTACTT
TCCAGCACCTATAGCACCAACGAGGTATCCGGGTGGTCATCATCCAGTACCTGGCATACCACCCGCAGCGCATGCGATGT
ACAAAGTAGACCACGTTCGAAGTACTGCTCCTCCAACACAACCTCTACCTAAACCTCCTTGTGATTTTCATCCTTCAAAG
GAGAGCATAGATGCGGCTATTGGAGATGTTTTATCTAAACCATGGCTACCACTTCCCCTCGGACTCAAACCTCCGGCTGT
CGACAGCGTGCTAGGTGAATTACAACGTCAAGGGGTGCCTAAAATACCACCAACTTGTGCATAG

>CcGLK1-2 |Locus_ID=BC332_34411 |CDS_len=1344 |strand=-
ATGCTAGCTGTGTCACCTTTGAGTAACACAACGGCAAGAGATGATAACACGATGGAGAGTTTTGCGATTGGAGGTGGAGT
TGATTTCCCTGATTTCGTTGGGGAGAATTTACTTGACAGCATTGATTTTGATGACCTATTTGTTGGAATCAACGATGGAG
AGGTACTGCCAGATTTGGAAATGGACACTGAAATACTTGCAGAATTTTCAGTTAGTAGTGGCGATGAATCCGATGTGAAT
AACTATAGCAGTAGTAATAAAACTACGTTTATTTCTACTGCTACAAAAAATGTAGAAAGAAAAGATGAAACGGAAAGAGC
AGCCTCGGATGTAGGTTCGGGTTTAACGAGCTTAAATCAAGGAGAAGAAATTGTTAGTACTCAAAAAAGTGAAGAATCTA
CACTACAAGTGAAACAGAATATCACTCCAAAAGAAAGTGATAAAGGCAAAAAGTCATCCAAGAATAATCTTCCAGGCAAG
AGGAAAGTAAAGGTGGACTGGACGCCAGAATTGCACAGAAGATTTGTACAAGCAGTAGAGCAGCTAGGTGTAGATAAGGC
AGTCCCATCAAGAATTTTGGAGATCATGGGAATCGACTGTCTTACTCGTCATAATATTGCCAGTCATCTTCAAAAATATC
GATCCCATAGGAAACATTTGCTTGCAAGAGAAGCTGAGGCCGCGAGTTGGAGCCAGAGAAGGCAACTTTACGGTGGTGCA
GCCGTGGTAGGAGGGGGAGGAGGAAAGAGAGATATGACCCCGTGGCCTGCACCAACTATTGGCTTTCCTCCTCCTCCTAC
GATGGCGGCTCCAATGCCTCATCATTTTAGACCCCTACATGTATGGGGTCATCCATCTGTCGACCAATCATATATGCACA
TGTGGCCTAAACATCTAGCGCCTTCGCCTTCTCCACAACATCCATCACCAGCATGGGCTCCTCCTCCTCATCTTCATCCT
CCGCCACCTTCAGATCCTTCTTTTTGGCATCCACACCATCAACGAGTACCAAATCCTCTTACACCAGGAACTCCTTACTT
TCCAGCACCTATAGCACCAACGAGGTATCCGGGTGGTCATCATCCAGTACCTGGCATACCACCCGCAGCGCATGCGATGT
ACAAAGTAGACCACGTTCGAAGTACTGCTCCTCCAACACAACCTCTACCTAAACCTCCTTGTGATTTTCATCCTTCAAAG
GAGAGCATAGATGCGGCTATTGGAGATGTTTTATCTAAACCATGGCTACCACTTCCCCTCGGACTCAAACCTCCGGCTGT
CGACAGCGTGCTAGGTGAATTACAACGTCAAGGGGTGCCTAAAATACCACCAACTTGTGCATAG

>CcGLK1-3 |Locus_ID=BC332_34315 |CDS_len=1344 |strand=+
ATGCTAGCTGTGTCACCTTTGAGTAACACAACGGCAAGAGATGATAACACGATGGAGAGTTTTGCGATTGGAGGTGGAGT
TGATTTCCCTGATTTCGTTGGGGAGAATTTACTTGACAGCATTGATTTTGATGACCTATTTGTTGGAATCAACGATGGAG
AGGTACTGCCAGATTTGGAAATGGACACTGAAATACTTGCAGAATTTTCAGTTAGTAGTGGCGATGAATCCGATGTGAAT
AACTATAGCAGTAGTAATAAAACTACGTTTATTTCTACTGCTACAAAAAATGTAGAAAGAAAAGATGAAACGGAAAGAGC
AGCCTCGGATGTAGGTTCGGGTTTAACGAGCTTAAATCAAGGAGAAGAAATTGTTAGTACTCAAAAAAGTGAAGAATCTA
CACTACAAGTGAAACAGAATATCACTCCAAAAGAAAGTGATAAAGGCAAAAAGTCATCCAAGAATAATCTTCCAGGCAAG
AGGAAAGTAAAGGTGGACTGGACGCCAGAATTGCACAGAAGATTTGTACAAGCAGTAGAGCAGCTAGGTGTAGATAAGGC
AGTCCCATCAAGAATTTTGGAGATCATGGGAATCGACTGTCTTACTCGTCATAATATTGCCAGTCATCTTCAAAAATATC
GATCCCATAGGAAACATTTGCTTGCAAGAGAAGCTGAGGCCGCGAGTTGGAGCCAGAGAAGGCAACTTTACGGTGGTGCA
GCCGTGGTAGGAGGGGGAGGAGGAAAGAGAGATATGACCCCGTGGCCTGCACCAACTATTGGCTTTCCTCCTCCTCCTAC
GATGGCGGCTCCAATGCCTCATCATTTTAGACCCCTACATGTATGGGGTCATCCATCTGTCGACCAATCATATATGCACA
TGTGGCCTAAACATCTAGCGCCTTCGCCTTCTCCACAACATCCATCACCAGCATGGGCTCCTCCTCCTCATCTTCATCCT
CCGCCACCTTCAGATCCTTCTTTTTGGCATCCACACCATCAACGAGTACCAAATCCTCTTACACCAGGAACTCCTTACTT
TCCAGCACCTATAGCACCAACGAGGTATCCGGGTGGTCATCATCCAGTACCTGGCATACCACCCGCAGCGCATGCGATGT
ACAAAGTAGACCACGTTCGAAGTACTGCTCCTCCAACACAACCTCTACCTAAACCTCCTTGTGATTTTCATCCTTCAAAG
GAGAGCATAGATGCGGCTATTGGAGATGTTTTATCTAAACCATGGCTACCACTTCCCCTCGGACTCAAACCTCCGGCTGT
CGACAGCGTGCTAGGTGAATTACAACGTCAAGGGGTGCCTAAAATACCACCAACTTGTGCATAG

>CcGLK1-4 |Locus_ID=BC332_34226 |CDS_len=1344 |strand=+
ATGCTAGCTGTGTCACCTTTGAGTAACACAACGGCAAGAGATGATAACACGATGGAGAGTTTTGCGATTGGAGGTGGAGT
TGATTTCCCTGATTTCGTTGGGGAGAATTTACTTGACAGCATTGATTTTGATGACCTATTTGTTGGAATCAACGATGGAG
AGGTACTGCCAGATTTGGAAATGGACACTGAAATACTTGCAGAATTTTCAGTTAGTAGTGGCGATGAATCCGATGTGAAT
AACTATAGCAGTAGTAATAAAACTACGTTTATTTCTACTGCTACAAAAAATGTAGAAAGAAAAGATGAAACGGAAAGAGC

AGCCTCGGATGTAGGTTCGGGTTTAACGAGCTTAAATCAAGGAGAAGAAATTGTTAGTACTCAAAAAAGTGAAGAATCTA
CACTACAAGTGAAACAGAATATCACTCCAAAAGAAAGTGATAAAGGCAAAAAGTCATCCAAGAATAATCTTCCAGGCAAG
AGGAAAGTAAAGGTGGACTGGACGCCAGAATTGCACAGAAGATTTGTACAAGCAGTAGAGCAGCTAGGTGTAGATAAGGC
AGTCCCATCAAGAATTTTGGAGATCATGGGAATCGACTGTCTTACTCGTCATAATATTGCCAGTCATCTTCAAAAATATC
GATCCCATAGGAAACATTTGCTTGCAAGAGAAGCTGAGGCCGCGAGTTGGAGCCAGAGAAGGCAACTTTACGGTGGTGCA
GCCGTGGTAGGAGGGGGAGGAGGAAAGAGAGATATGACCCCGTGGCCTGCACCAACTATTGGCTTTCCTCCTCCTCCTAC
GATGGCGGCTCCAATGCCTCATCATTTTAGACCCCTACATGTATGGGGTCATCCATCTGTCGACCAATCATATATGCACA
TGTGGCCTAAACATCTAGCGCCTTCGCCTTCTCCACAACATCCATCACCAGCATGGGCTCCTCCTCCTCATCTTCATCCT
CCGCCACCTTCAGATCCTTCTTTTTGGCATCCACACCATCAACGAGTACCAAATCCTCTTACACCAGGAACTCCTTACTT
TCCAGCACCTATAGCACCAACGAGGTATCCGGGTGGTCATCATCCAGTACCTGGCATACCACCCGCAGCGCATGCGATGT
ACAAAGTAGACCACGTTCGAAGTACTGCTCCTCCAACACAACCTCTACCTAAACCTCCTTGTGATTTTCATCCTTCAAAG
GAGAGCATAGATGCGGCTATTGGAGATGTTTTATCTAAACCATGGCTACCACTTCCCCTCGGACTCAAACCTCCGGCTGT
CGACAGCGTGCTAGGTGAATTACAACGTCAAGGGGTGCCTAAAATACCACCAACTTGTGCATAG

>CcGLK2 |Locus_ID=BC332_25081 |CDS_len=936 |strand=-
ATGCTTGTTGTATCTACACCATTGAGCTACAAAAATGAAAGGGGAAATTATGATTTATTTCAAGATTTTCCTGATGGGAA
TTTAATCGACACCATTGATTTTGATGACTTTTTTGAGGGAATCAACGATGGAGATTTGCTGCAAAATTTGAAAATCCTTG
ATGAATTTGATATTAGCAAGAATAATACAACTACTAATCTAAATGTGAAAACAAAGTCAAAGGAAAATGATAAATCCAAG
AAATCGTCAAGCCAAATCAAGAATCCTGAAGGGAAGAAGAAGGTGAAGGTTGATTGGACTCCAGAGCTGCACAGGAGATT
TGTAAAAGCAGTAGAGAAATTAGGTGTTGATAAAGCAGTTCCATCAAGAATTTTAGAGCTTATGGCTACTGATGGTCTCA
CTAGACATCACATTGCTAGCCATCTTCAAAAATATCGAGCTCATCGAAAACATTTATTAGCAAGGGAAGCTGAGGCAGCA
AGCTGGACCCAAAGGAAGCAAATGTATGGTGGAGCCACCGCGATCGGAGGCGGAGGGAAGAGAGTTATAATGAACCCATG
GTCTGCACCACCAACCATGGGTTTTCCACCCATGGCTCATCATATTAGACCCTTACATGTTTGGGGGCATCCATATGTAA
ATAATTCTTTTTGGCATCCACATTATCGAGTACCAAATTCTCTTGCACCAGGCACTCCTTGCTTTCCTTCACCAACGAGA
TTTGCAGCACCTCTTATGGTCCCTGGCGTTCCACCACCCATTGCATCAGGACAAACTCCACATTTGCATCTTACAAAAGA
GAGCATAGATGCAGCAATTGAAGATGTTTTATCAAAGCCACAAACGCCACTTCCTATAGGATTGAAACCTCCATCAATTG
ATAGTGTGTTGAATGAATTACAATGTCAAGGGATTACAAAAATACCTCCAACTTGA

>CcHHO1 |Locus_ID=BC332_05708 |CDS_len=1350 |strand=-
ATGGGTTCAAATTCTAGAGAAATGGACATAGATTTGAACTTTATTTATGTTCCAAAAATAATTTCAGATGTTTTAACTGA
AATTTCTGTCATGGATGACATCTCCAAGAAATTAATAAAGCTCAATCAATTTATTATTGCTTTAGAAGAAGAGCTCAGAA
AAATCGAAGCTTTCAAACCTATTGAAAGATTAAAGGAGGAAGCTTTACAGTATAAAGGGAAAGATAAAAAGCCTGTGATG
GAGAAATTCATCTCACTGAAGAAAGGTAATTCTGATGAAAGTGGAAGGGTAAAAAAGTCAAATGATCTTAATGATAAGAA
AAACTGGATGAGTTCGGCTCAGCTGTGGAGCACTCCAGTTCAGTATGAACAAAGCTATGATCTTGAAAAGCAAGGACCTT
TTTTACACCTAAATTCTGGTGGGGTAGAAGAGAAAAACAAAGAGAAGGAATACCAGTTAGGGGTACATAAATTGAATAGT
GATAGAGGGGCATTTTTGCCATTTCAAGGACAAACTTTGAAGGATGGGAAGAATGGTTTGCCAGTTAAAGATTTATCTCT
GTGTATGGCAGTAACAGTGGCTGAAGGGCAAAAAGGTCAAAATCCAATTGATTTGAGTGTAAAAAATGATAATGGCACAT
CAAATGGGTGTAGTTTTTTACAGGATAAATCACAACAGAGGAAACAGAGGCGTTGTTGGTCACCAGAATTGCATCGTAGG
TTTGTTGATGCTCTTCATCAGCTTGGAGGTGCCCAAGTGGCTACACCAAAACAGATCAGAGATATCATGCAAGTTGATGG
CCTGACCAATGATGAAGTGAAAAGCCATTTACAGAAGTATAGGCTTCATGTGCGACGAGTCCCAGTATCGGGTTGTTCGT
GGTCTAACACGGATGAAATAGGAGAATCATCGAAGACAAATGGCACACAATCTGGTTCCCCTGAAGGTCCTCTTCATTTT
ACTGGTTCAGGAAAAATGACAAGCGATAGTCAAATGCAAGATGCAGCAACGTCTATGGGGGCAACTAATATTGCCACATC
AAGTCGCACAAATGCTCCGCCTACAATGGCACTGACGGAGAAGCCCGAAAAATTTACGGGCATTGACTTTAAACGATGGC
AACAAAAGATGTTCTTTTACCTCACCACTTTGTGTCTGCAACGGTTCACTAGCGAAGACGCTCCTGAGGTGCCCGAGGGA
ACCTCGGACAAAGAGCGTTTCGTAATTGTAGAAGCTTGGAAACATTCAAACTTTCTTTGCAGGAATTATATTCTCAGTGG
TCTCCAAGACGACCTCTACAATTTTTACAGTGGAACTAAGACATCGAAGGAATTGTGGGGGGGCACTTGA

>CcKAN1 |Locus_ID=BC332_00869 |CDS_len=873 |strand=-
ATGGAACTATTCCCAGCACAACCAGACTTATCCCTAAAAATTAGCCCACCAAACATCAAGCCCTCCTCATCTTCAACTAA
TTGGAAGAGGAAAACTACAAGTAGTACTGAAGACGAGAATTTGGATTTAGGGTTTTGGAAAAGAGCTATGGTGGAATCAA
CAAATTCTATGTTGAATAATAATAGTAACACTTCTTCTTGTTTTGAGCTTTCTTTATCCAATAATCCAACTAAACCTAAT
AATTATTCTTGTGACCCTAATTCTTCACATCATCTCCAAAATTACATTTTGCAACAAAATCAAAGGATAATAACCACCCG
AGAGCTTGGTTTCTTGATGCAACCTATTAGAGGAATTCCAGTGTACCATCAAAAGCCCTTTGATAATACCATTACTACAT
TTCCTATACCGTCTTCTTCTAATTGTTCTATTCTTAACAATATTAATAACAACAACACAATTACTTCTTGTGCCTCTACT
AGTCCTAATTTTCAACAAGGTGGGTTTCTGAGATCAAGATTTTATTCAAGATTTCCAGGCAAACGGAGCACGAGGGCTCC
CAGAATGCGATGGACAAGTACTCTCCATGCTCGTTTTGTTCACGCTGTTGAGCTCTTAGGTGGACATGAAAGAGCAACAC
CAAAGTCAGTTCTTGAGCTTATGGATGTCAAAGATCTCACTTTAGCACATGTCAAATCCCATTTACAGATGTACCGGACA
GTGAAGACAACTGATAGAGCAGCTTCTTCAGGCCAATCAGATACATCAGAGGAATTGATTGTTGAAACTGAAGAATATTT
AAGAAAGTCTGAAATAACAATTCAACAAGGTAGACGGAATATTCATCAAAATAATGATTTTCAGGGCCTTTAA

>CcKAN2 |Locus_ID=BC332_15978 |CDS_len=1257 |strand=+
ATGGAGCTATTCCCAGCACAACCAGACTTATCTTTACAAATCAGTCCACCAAATAGCAAAGCATCATCAACAGCTTGGAA

GTCTAGTATGGTCACTAATAATACTAATAGTACTACTAATCAAGATGAGATGGATTTACTCTTCTGGAAAAGAGCTTTGG
AATCAAGAAACTCAAAATCTGATAATAACAGCAATAATTCTTCTTCTCTTTTTGAACTTTCCTTATCCAATATTCCAAAT
AAACCTTCTCCTCCTCATCAACTTATTAACACTACTTCCCATTTCCATCCCACCATCCAAAACACTCACACCAATTTCAT
CCACCAATCTTTACAACAAAATCAAGAACTTAGTTTCTTAAGACCCATAAGAGGAATTCCAGTGTACCATCAAAACCCTC
CTCCTCTTCCTTCTCCTTCTATTCCCGTTTCTCATCATAACTACCCAATATTTGCTCACCAAACTTTTGACAATACTAGT
ACTACTGCAGCTACATTACCTATACATTCTTCTGGTTGTTGTATTAGTAGTTTTAATAACAATATTAGTAACAAAACTAT
CTCTAATTCCTCACCAATTATTCCTTTTCATCAATCCCATCATCAAGCTGGCCTAATGCGATCGAGATTTTTGTCAAGGT
TTCCAGCCAAACGTACCATGAGAGCTCCACGGATGCGCTGGACTAGTAGTCTTCATGCCAGATTTGTTCATGCTGTCGAG
CTCTTGGGTGGCCATGAAAGAGCAACTCCCAAGTCGGTTCTTGAGCTAATGGATGTGAAAGATCTCACTTTGGCACATGT
TAAATCCCATTTACAGATGTATCGCACGGTCAAGACTACCGACAGAGCAGCAGTTCCCGCTTCTTCAGGCCAATCGGAGG
TATTCGATAATGGGTCTTCTGGGGATAATTCAGAGGATTTAATGCCTGACATGGAAAATTCAAGAAAGTCTGCTGATTTA
TTATCTGACCAACATGCTGGCAGAAATAATATGCATCTTCAAGAGAAAGATTATCATGGTCTTTGGAGCAACTCTTCAAG
CAGGGAAAGTTGGCAGCTGCATGGCAAACATGGTGACTACCCTGGGAACATGGCACCTCTTGAGAGAACAGAGAAAAATC
AGCATAAAGACATGGAAGCAAATGCAAAATGCTTAAGCTATGACAGAATATCAGGAGAAGTGAGCTCATCAAGCATAACA
GAGACAAGTCCAAAGAAGCCTAATCTTGAATTTACTTTGGGAAGGCCTTCAGAGTAA

>CcKAN3 |Locus_ID=BC332_11999 |CDS_len=924 |strand=-
ATGAGGGACACAGTACTTTCAATGGTACCTGATCTTTCTTTACAGATCAGCTTACCACCATGTATTTCAAAAAGTGATCA
GTTCAAAGAAGCTATACGCTTCGACTCCACAACAGATTCAGGTAGCAGTGCTAGTGTGGGGAGCGATTTGAGCCACGAAA
ATAATGGATATTTTCACCTAGACAAAATTAAACCGTTTAATCTTCCCTTAGTTCCTTCTTATGAGCCAACCCTGAGTCTA
GGGTTCGGACAGGTACCAACAGGTACAAACCATCAGTATCAGCATTATCAGCCACAAATATACGGTCGAGATTTCAAGAG
AAGTTCAAGAATGAATATTAGTGGGGTGAAAAGAACCGCTAGAGCTCCTAGAATGAGATGGACTTCAACTCTTCATGCCC
ATTTTGTTCATGCCGTTCAGCTCCTTGGTGGACATGAACGAGCAACACCAAAATCCGTGCTAGAGTTGATGAATGTGAAG
GATCTAACTTTAGCTCATGTGAAGAGTCACTTACAGATGTATAGAACAGTGAAGAGCACTGACAAAGCCACAGCTAAAGG
GCAGGGAGAAAATGTATTTGTGAACCAACACAAGGGGATTAATAATATTGGTGAATATGAAAAATCTGGTGAAGAGATTG
ATGCAGTATCACAATCAAGCACAATGCCACAAAATATGGCTCATAATATGAGGGATCCATCGTCATTCATGCCTGACCAG
ACAAATGCATGGAGCCACTCAAATAGAGAAAGACCATTTGCTAATTATCCTCACATCTACTCAAATATGGGCGGAATTGA
GACAGCCAATAACTCATCTTCTAATGATACATTGGTTAATCTTGAATTTACACTAGGAAGACCAAGCTGGAAAATGGAAC
AAGCTGGATCATCATCAAATGATTTGACTCTTCTCAAGTGCTAA

>CcKAN5 |Locus_ID=BC332_19106 |CDS_len=336 |strand=+
ATGCCAGAAAGTAGCAAGAAGCACAAGGGAGAAACATCTGAAAAAGTTGGGCCAAGTCATGACTTGAACAGAAAACCAAG
ATTTAAGTGGACAGTGGAACTCCATGAACACTTTGTTAAAGCTGTCAATGAATTAGGAGGTCCTTATGAAGCAACACCGA
AGAATATTGTGAAACTAATGGATGATGAAGATATCACACCAGACCATATCAAAAGCCATCTCCAGATATATCAAGGTAGA
AGCAGATCTATTAAGGTCCAGGGAGTGTCATGTCATCCGCAAGTTTTGATTAAGGCCCGAGGAGCGTTATGCCACCCGCA
AGTTTCGATCGATTGA

>CcKAN6 |Locus_ID=BC332_18917 |CDS_len=753 |strand=+
ATGATGGAATATTTCTCAATGATGAAAAAGAGGAGTTCTAATAAAATAGGGGTTAGACAATACAAGAAATCATCAGTTCC
TCGTCTTAGGTGGACACCTGAACTTCATGAACTCTTCATTGAAGCTGTTCAACTTCTTGGAGGAGGACATAAAGCAACTC
CAAAGAGAATTCAACAGATGATGGAAGTAAAGGGATTGACGATTTCTCATGTTAAAAGCCATCTCCAGATGTACAGAAAC
TTGAAGGAACGAGCCAGCCCAAATATTGTAAGCTCTGTCAACAACTTTCTTGAAGAGAGGCCACAGTTCTCTTGGTCTTC
CCCACGGCATGTGTGTGAAACATTAGAACATCGTCCTTCAAAGAGCAGCAATCAGATAAACCCTGGAAGTGATCAATCCC
CTTACAAAGCAAGAGTCGATGCACACAGCGATCAAATTGCGGAAAATAATATATATTGCTGTGACTACCAGGAAGCACAA
GGAAGTTTAAGTAGTGGAATGACAAAGGAAGAAGAAGAAAGTGGCGGTCCAACAAGTGAAATTTGTGAGTTTCCAGAGGC
AGATGGCGATTATAAAAGGGAGAACAATATTTGGCCTTTGGATGATTGGGTAGCTCGGTGTACTAAAGGTTCAGCTGAAT
ATTCCCAATCTGATTCCTCCACAAAGTTTCTAGACCAGCAGGTTTTGCAATCTCCAAAGGACAATTCTCACATTAACCTA
GATTTATCTATCTCTTCATATTTCTATTGTTAA

>CcKUA1 |Locus_ID=BC332_00616 |CDS_len=1032 |strand=-
ATGACCCGAAGATGTTCACATTGCAGTACTAATGGGCATAACTCAAGAACTTGTCCTAATAGAGGTGTTAAGTTGTTTGG
AGTTAGATTGACTGATGGGTTGATTAGAAAAAGTGCTAGTATGGGTAATTTGACTCATTTTGGTGGTGGTGGTGGAGGGG
GAGGTGGTAGTAGTACTGGTCTAAATGGTGTTGTTCATGATTCACCTGGTGATACACCTGATCATCCTGCTGTTGGTGGT
GCTGCTGCTGATGGTTATGGTTCGGAAGATTTTGTTGCTGGTTCTTCTTCTAGCCGTGAAAGGAAGAAAGGGGTTCCCTG
GACTGAGGAGGAGCATAGGATGTTTCTACTTGGTTTGCAAAAGCTTGGTAAAGGTGATTGGCGTGGAATTGCTCGTAACT
ATGTAATTACTAGAACACCCACCCAGGTTGCAAGCCACGCCCAGAAATATTTCATTAGGCAAAGCAATATGTCAAGGAGA
AAAAGACGCTCCAGTCTGTTTGATATTGTTGCTGATGAATCAGGGGACACTCCAATGGTATCAAGGGATTTCCTCGCTGA
AGATCCGCAAGCCGAGATGCAAAGCAACAATCCGCTGCCTCCTACTCCTGTTGTGGATGAAGAATGTGAATCAATGGGTT
CAGCAGCTTCTGCCAACTCCATCGATGGGGAACATTCTCTTCCTATACCTGAGAGTTCACAGTACCCTCATCCACTCGTA
TATCCTGCTTATGTTGCTCCATTTTTTCCAATGCCTTATCCATGTTGGCCAGGTTACACTGCAGAGCCAGCAAAGGCAGA
GACCCATGAAGTTCTGAAGCCAACAGCGGTTCATTCAAAGAGTCCAATTAATGTTGATGAGCTGGTTGGTATGTCAAAGC
TAAGTTTAGGTGAATCCATTGGTAATGCCAAGCCACCTTCTCTTTCACTAAAGCTGGTAGAAGGCTCCTCCAGGCAGTCA

GCTTTCCATGCTAATCCGTCATCTGGTAGCTCAGGCATGAACTCTAGCCACAGTCCAATCCATGCAGTTTAG

>CcKUA2 |Locus_ID=BC332_30669 |CDS_len=1143 |strand=+
ATGACTCGGCGGTGTTCCCATTGCAACAACAATGGACACAATTCCCGAACTTGCCCCACCAGAGGCGGCGGCGGCGGTGC
CACCAGCAGTATCGGTGGTGGCTCTGCCATCGGTGGTGGAGGTGTGCGGCTGTTTGGTGTGAGGCTTACAGATGGATCGA
TAATGAAGAAAAGTGCTAGCATGGGGAATCTATCTTCCCTTCACTATCATTCTTCTTCTTCCGCAGCTGCTTCGCCTAAT
CATCCTGGTTCTCCTTCTTCAGATGTTCTTCGTGAAGCTGTTCATTTGACTGATGGTTATCTCTCTGATGATCCTGTTCA
TGCTTCTTGTTCTGCTAATCGCAGAATTGAAAGGAAAAAAGGTGTTCCTTGGACAGAGGAAGAGCACCGACTTTTCTTGA
TCGGTCTACAAAAATTGGGCAAAGGAGATTGGCGGGGTATATCACGAAACTTTGTGACATCAAGGACTCCTACCCAAGTA
GCTAGCCATGCCCAGAAGTATTTTATCCGGCAGAGTAGTGCTACTCGGAGAAAGAGAAGATCCAGTCTTTTTGACATTGT
TGCTGATACGGCCGCTGATGCTTCCCATCCACTTCCTGATGAACAATTTATGCTCCCACCTAGAGCAATGGAAAGTGATA
AGGAACAGTTAGCACCTTCTGCAACAAAAGCAATAGAAACTGATTTTGCAGATTCACTCCCTTCCTTGGATCTTTCTCTC
AAGTCAGATTTTGAATCCATGGAAACCGTTCCAAGTGAACCTGTTCAAGAAACGAAACCAAATACCGCAACCAGCGAGAT
TCCTCCAGCATTTCCAGCATTCTTCCCAGCTTACATTCCAGTTCCTTATCCCTTCTGGCCATCAAATGCAATTCCTGTGC
CTGAAGATAGGGGACCAGAACCATCCCATCATCAGATCCTCAAGCCAATTCCAACCGTCCCCAAAGAACCTGTGAATGTA
GATGAACTAGTGGGAATGTCCCAGCTCACTTTAGCAGAAGCTGGTTCTGGCCATTTTGAGCCTTCACCACTCTCCCTTAA
GTTAACAGCAGAACCGTCAAGACAATCAGCTTTTCATGCAAGCACACCAGGCAAAAGCTCAGAGATTACTAAGGGTGAAA
CTGCTCCTATTCAAGCGCTATAA

>CcKUA3 |Locus_ID=BC332_24632 |CDS_len=720 |strand=-
ATGGGGAGAAAGTGTTCACATTGTGGCTATATTGGTCATAATTCAAGAACCTGTAGCACATTAAAATGTGCTATTAGTGG
TAGTAATTTTATTGGTGGATTAAAACTTTTTGGAGTACAACTTGATACTGCTAATTCTTCTTCTTCTTCTTCATCATCTT
CTTCATTTTCTAGCCATAATTTGACGAAAAGTTTTAGTTTGGATTGTTTGTCTTTAACAAATAGCCATATTGGCCATAGC
CACTTATCATCATCATTATCTCCTTCTCCATCTATTAATGAAAGTTGTGAAAAGAGTACTACTTCTATTAATAATAATGG
TTATCTTTCTGATGGTATTGGTGTAGGGTGTGTTGGTGAAAGGAAGAAAGGAGCACCATGGACAGAAGATGAACATAGAA
AATTCTTAATTGGACTTGAAAAGTTAGGTAAAGGTGATTGGAGAGGAATATCAAGAAATTTTGTGACAACAAGGACACCA
ACACAAGTTGCAAGTCATGCACAAAAATATTTTCTAAGACAATCAAGTATCAACAAAAAGAAAAGACGTTCAAGCCTTTT
TGATATGGCAAGGATCAACAACAAATTTGAAGATTGTCAAGAAAATTCACATTGGATTTATGGATCACAAAATTCTCATC
ATAATGAAGTGCCAAATAATAAGTCCATTTCTTCAAATGGAAATATTGATTTAGAGCTAAGTCTTTCAAATAATATTGTG

>CcKUA4 |Locus_ID=BC332_15336 |CDS_len=828 |strand=+
ATGGGGAGCATGACGAAAGATGCCAGGAAGTGCTCACATTGTGGGCACAATTCAAGAACATGTAATTCGAAAGGAGTTGG
CGGAATCAAATTATTTGGAGTTCGGATCGACGATAATTATAATAGTAACAGTAACAATAATAATCATGGTCATGGTAACA
TAAAAATTAAGATGCGGAAGATAGGTGATTATGAATCCATAAGAAGGAGCAAAAGTTTAGGCAATCTAGAACATGCCGCG
AATTATGACAACATCGGTGGCGTTGAGGCGGGATATCTTTCTGATGGGCCCATTCATTCTCTCAGACATAGAAAAAAGGG
GACATCATGGACTGAGGAAGAGCACCGGTGTTTTTTAATTGGCTTAGAAAAGCTTAGAAAAGGTGATTGGAGAGGAATAT
CAAAACATTATGTGCCTTCCAGGACGCCAACACAAGTAGCTAGTCATGCTCAAAAATATTTCATTAGAATGTCCTCAATT
GATAAGAAGAAACGTCGGCCTAGTGTTTTTGATGTGTATCTAAAAGATCTGAATCCACAGATTCCCTCCAAGTCCTCACC
TTCTGTCTCAAACATGGTTTCTGAAGTCTCTAAGGATGCAAGTTCCCGGGTGGTGCAACTGTTTGACACTTGCCAAATTA
AAGGACAGGCTAGCAAAGGTGTTCCTTGTGCAACTCGTGAAAGGCCTCCACTTTCACCATCACCAATTAGTAGACCTTTT
GGAGTCCGTAACTTACGCCACATGCCTTACATGATGGGAGCTCCTCCGAATATCCATAGCGTTACACCAGCCAAAGCAGG
ACCAACAATCTCATGGATTCTCCCGTAG

>CcMYB1R-1 |Locus_ID=BC332_32578 |CDS_len=846 |strand=-
ATGACGATAACCTACGGCCATAGCTCTTTCATCTCCGCCGGAGAGTTGCCGCCGGCCGACGTGTCCGGAGGAGAGATTAT
GCTGTTTGGTGTGAGGGTGAAGCTGGACCCCATGAGGAAGAGTGTGAGTTTGAACAATCTGTCTCAGTATGAACAGCCTA
ATTCTAACGACAACAATAATAGCAACGGCGGAAATACCAATGACTCTTCTTCTAAAGTCGCCGATGAGGGCTATGCTTCT
GCCGATGACGCTGTTCCTCATCACTCCGGTAGTGGCCGTGAGCGCAAGCGAGGAGTTCCATGGACGGAGGGGGAGCACAA
GCTATTCCTTTTGGGATTGCAGAAAGTAGGTAAAGGAGACTGGAGAGGAATCTCTAGAAACTTCGTCAAGACTCGCACAC
CGACACAGGTTGCAAGTCATGCTCAGAAATACTTTCTCCGGCGAACTAACCTCAATCGTCGCCGCCGCCGTTCTAGCCTC
TTCGATATCACCACTGACTCGGTATCTGTATTGCCAACTGAAGAAGCAAAGAATCTGCAAGAAGCTCATGTAGAAACTTC
CAAAATCAATGCATTTCAGGTGACATCTGTGCCAATAAAATTTGCGCCTCTTGTCTTACCAGCACAAATGGACAAGCCAA
TAGAAAGTCCAATCCAAAGTTATAACGCTTCATCTATATACCTCCGGCCTGTTCCTGTTGTTGTTCCAGTGTCTAATCAG
AGCTCTGCAATAGAGTCATCTTCGTTATCGTTGACATTGTCCTTGTCATCACCATCATCATCATCTACTAGATATCAAGT
GATGTCAAATTTTAGTAATGGAGAGAGCATCATCAGTGTGGCATGA

>CcMYB1R-2 |Locus_ID=BC332_16646 |CDS_len=888 |strand=+
ATGTCCAGCGCGTGCGGTGATGAATCATCGGCGACGGAAGTTACCGGCGCCGGTGAAATCATGTTGTTTGGCGTGAGAGT
GAAAGTCGATCCTATGAGGAAGAGCGTCAGTCTCAACAATCTCTCACAGTACGAACAGCCGAATATTAACAACTCCGGCG
GTGATAGCAAAAATGAATCGTTGAAAGTGGCGGCTGATGAAGGTTATGCCTCTGCAGATGATGCTGTTCAACATCAGTCT
AACTGTGGACGTGAGCGTAAGCGAGGAGTTCCTTGGACGGAGGAAGAGCACAAGTTATTCCTTCTAGGATTGCAGAAAGT
GGGTAAAGGAGACTGGAGAGGAATCTCTAGAAACTTCGTAAAGACTCGTACGCCGACGCAGGTTGCAAGTCATGCTCAGA

AGTATTTTCTCCGTCGAAGCAACCTAAACCGCCGTCGTCGCCGATCTAGCCTCTTTGATATCACCACTGACTCGGTATCA
GCAATGCCAAAAGTAGAAGGGGAAAATAAGCAAGAAATCCCAGTTCTAGCACCAGCAACATTGCCTACTGTAGAAACTAC
CAAAACAAATGCATTTCCGGTGGCACCGACTGTTGGTCCTATCATGATACCAGCGCAGATTGATATGTCAAGAGATAGTC
CAACGCTGTTGCGACGCAATCATGGGAATTCATCAATGCTGGTTGGTGCTGTTCCTATGTTTTCACTGCCCAATCCATCA
CCAGTGATTGACCTTAATGCGAACCAGAACACAACAATTGAGCCCTCGCCTTTGTCACTGAGATTATCATTGTCACTTGA
TCAGGGACAAGCATCATCTACTAGACACCTGGGATATCAAGTCATGTCAAGCTTCAGTAATGGAGAAAGCATCATCAGTG
TGGCATGA

>CcMYBC1 |Locus_ID=BC332_26707 |CDS_len=825 |strand=-
ATGAGGGAAGAAGATTCCAATTGGTTTGCTAAATGGGAAGAAGAATTACCATCTCCAAAGGAACTGATGCCTTTATCCCA
AACCTTAATTACTCCTAGTTTAGCCATAGCTTTTCATATTCAAAACCCCAATACCCCAAATCCCAGAATGTCACCACTAG
TTCATACTCCTCCATCAGCCGAGTTTGAGTCGAACTCGGCTGAGTTGGGTGGCAGGGCAGGTTCATCAGGTGTTGGTCCT
GATGAACCTGCCCGAACCCCGAAAAGGGCTAGACTTGTGTGGACCCCACAGCTACACAAGAGGTTTGTGGATGCAGTTGC
ACAATTGGGGATCAAGAATGCTGTCCCAAAGACTATAATGCAGTTGATGAGTGTAGATGGCTTAACTCGCGAAAATGTTG
CTAGTCATTTGCAAAAATATAGGCTTTATTTGAAGCGTATGCAGGGTTTTTCTAGTAGTAGTATGTCCGGTGCAGGGGTG
GATCCCGCAACAGATCATCTGTTTGCGAGTTCACCCGTGCCTGCACATTTTTTGCATCCAGGGAGGGGAAATTCTGAACA
TTATATGCCATTTGTGCCAGTGGCTGCAATTGTTGGACATGCTCCACAACTTCAGCAGCAGCATAGGCATTTGGGGCCAT
CGCCAAATGGGCAATTTGAGGTTCCATTTTTGTCTCGCCAGTCGCAGCAGCAGCCGGTTCAGAGAATGGGGACATCAGTT
CATAGTAGTAGTCCAGTGTTGCCTTCTTATGTAGAGGGGTTGGAATCAGCTACAACTGCTAATGGGAGAAAGGTTCTTAC
TTTGTTTCCAACTGGGGATGATTGA

>CcMYBC2 |Locus_ID=BC332_27055 |CDS_len=873 |strand=-
ATGAGGGAAGAAGATTCAAATTGGTTTGCTAAATGGGAAGAAGAATTGCCTAAACCAGAAGAGTTAATGCCTTTATCACA
AACCCTTATAACTCCGGATCTTGCCATAGCTTTTGATATTCCAAACCCCACTAGTCCAAATCCTCAAAGTAAACAGCAGC
AGACTCCTCTTGTTCCATCTTCTCAGCCAAATTCATCAGCTGAATTTGAGTCTGCTGAACTGAATGGGACAGGAGGTGGA
GATGAACCAGCAAGGACGTTAAAAAGGCCGCGTCTTGTGTGGACACCACAGCTACATAAGAGGTTTGTGGATGCAGTTGC
TCATTTGGGGATCAAGAATGCTGTCCCAAAGACTATAATGCAACTGATGAGTGTAGATGGATTAACGCGCGAAAATGTTG
CTAGTCATTTACAGAAGTATAGGCTTTATTTGAAACGTATGCAAGGTCTTTCGAACAGTGGAAGTGGTGGGAGTAATGCT
CAGTCTTTATCAGGAGCAGGTGTTGATCCAGCAATGGACAATTTGTTTGCAAGTTCACCTGTTCCGGCACATTTTCTGCA
CCCGGGGAGAGGTAATTCTGACCATTTTATGCCGTTTGTGCCAGTGGCACCTATGCAGCATCATCATCATATGGGAGTGG
TTGTTGGACACCATCCACAAGTTCAACAGCAGTATAGGCAATTTGGGTCACCGGCGAATGGACATTTTGAGCACCCTTTT
TTGTCTAGGCAATCACAGCAGCAGGTTCAGAGAATGGGAACATCAGTGCATAATGGTAGTCCTGTGATGTCATCTTATGT
GGAGGATGTGGATTCAGCCACCGCCGCGAATGGGAGGAAGGTACTTACACTGTTTCCAACAGGGGAAGATTGA

>CcMYBR1 |Locus_ID=BC332_13048 |CDS_len=903 |strand=+
ATGGGTAATTGTGGAAGAAATGGAGGAGTAAGGCAATACATAAGATCAAAAGTTCCACGTTTGAGATGGACACCTGATCT
TCATCATTGTTTTGTTCATGCTATTGAAAAACTTGGTGGCCAAGACAAGGCTACTCCTAAGCTTGTTCTACAGATGATGG
ATGTGAGAGGACTTACTATTTCACATGTGAAAAGCCACCTTCAGATGTACAGAAGCATGAAGAATGATGTCAACAGGCAA
GCAGAGAGAATGAGCACTCAACAAAGAAAACATCAGTCCTTAGAAGATCATGATTGTCATTATCAAAAACAAGAAGGGGT
GTGTGTTGAGCAACAAAAGCTATTGGTTTACCAACATTATCCATCTTTTAGCAGCAGCCCCATGGAGAGATCAGATTCTA
CACAATACTTTAACAGTATTCTACCCACAAAAAGAGCTAGAATAGAGGTGTGCAGCAACCAGAGAATACGTGAAGCAGTG
TCCAATCAGTACAACAGTAGAAATGATTATATAGAGAAAAATGGTGTAAAAGGAGAGAGGGCTGGTTTCATATGGCAACA
GAATAGTGAAACAAACAGACAAACAGCACATCCATTGTGCAATAGTAGTAGTACTACTACTCTTTTCTTTAACCCTCTTA
AGAGAAGCAATGAAATTCAAGAATCTGAATTTTTCAAGGTGACGAAGACACAAGATTCAAATGCAGGAGGCGGAGGAGGA
GGAGAAGACAATGGCGAATGTGGATTATCGTTATCTCTCTCATTGCATCGTCCTTCTACTCAGAGAAGCAATGCATCGTC
CATAATAAGTGAAATTGGTGAAACAATTTCTTCATATTCAGCAGGCTCCAATGTGAATCAACAGAGTGTTAGTGTTGATC
TTAATCTATCTATTGCCTTATGA

>CcMYBR10 |Locus_ID=BC332_32618 |CDS_len=1887 |strand=-
ATGGACCAAGGTGATGAGAAGGAGGATATGCTCCGGAAGCTGGAGAAGAAGTCCAAGAAAAGTAATAAGAATGCTGCCAC
CAGCGGTCTTGGAGGAGAGAGTGATGGGTGTGAAGTTAAGAAAAAGAAAAAAAGAAAAGGTGGAGAACATCCTGGAAAGT
GCAACAAAGACAATGATGAAGATGTTGTGAAGTATATTGAACGAAAGAAAAATAATAAGCTAAAGAAAAGCTGCTTGAAT
GAACCACCTTTAGACTTCAAAATTAGTGCTCCTGCAAGTGCAAGTGAAATGCGGGACAGTGAAAAAGTGGTTGAGACCCT
TGGTGAAATCTCTAGTGGAGACGTTGTGGACGAGATTAGGAGGAAGAAGAAATCCAAGAAAAACATCAAGACTACCAATG
TTGTAGGAGTGGCAAACACTGATATTGAAGCTAAGACTGATGATTGTGACCTCAAGAGAAAGAAAGAAAAGAAACATGGA
AAACATTCTGAGAAGTTTAATGAAGACAGCGATGAGGTTGCTTTGAGAATTGTTGAAGGAAAGAAGAGTAGGAAGTTGAA
GAAAAGCCGCGTGGCTGAGCAGGAGAAAATGCATGATACTGAGGATGTACAGGAAGATATTGTTGCTGAAGTCGACGAAG
GTGATATTTCTTCGCCTATCAAGATTGAAGAAAAAAACAGAACAGACAATGGGAATATTAAAAAGAGGAAAAAGGTAAAA
TCAGGACATAGCTCTGAAGATCCTACGCATGAGATTGAAGAAAAAAGCAGAACAGACAATGGGAATATTAAAAAGAGGAA
AAAGGTAAAATCAGGACACAGCTCTGAAGATCCTACGCATGAGATTGAAGAAGAAAACAGAACAGACAATGGCAATATCA
AAAAGAAGAAAAAGGTAAAATCAGGACACAGCTCTGAAGATCCTACGCATGAGAAGAGTGCAAAGAAAGTGAGATTTTCT
GGTCATGTTCAGATTTTCCCTTCTTCTGATGATCCAAGTGATGAGAAGCACGAGATAGAGGATGAAAATTTACTGCGTGG

CAAGCGATTCTCAAAGCTAGAAGATGAAATTGTGAAAGAGGCTGTTCATAAATACGTAGATTTTCATAACTTGGGCGAAG
AAGGGCTGAAAAAGATTTTAAATTCTAGATCTTATCCTGAAATAAGGGGTTGCTGGAAAGAAATAGGGAGTGCTATACCA
TACAGGCCTTATACTGCAGTTTATTATCGTGCACAGATCTTGTTTCGAAGGAGTGAATCACGTAAATGGACTGAAGAAGA
GTATGAAATTGTACGAAAATTCCAGAAAGAGCATGGGAACAATTGGAAGGTCTTAGCTGATGAACTTGGAAAACATCGGT
TTCATGTGAAGGATACATGGCGAAGGATAAAACGGCCAAATCAGAATAAAGGACAATGGACTCAGGAGGAGTACCAGTCT
TTGTTTGATTTAGTAAACACCGATCTGAGACTGAGGCTTTCTGAAGAGAAGAGATCTAAGTATGGGATGTTACGGGATAA
TATTGCATGGACTGCCATAAGTGACAAATTGTCCACGAGAACTGAGGCAAATTGCTGCTTGAAATGGTATGATCAATTAA
CATCACCCATGGTGGCCAAAGGTGAATGGGCGGATGTTGATGACTATCGCCTAGTTGATGCACTTTTTGAGCTGGACGCA
AGCTGCATAGAAGATGTGGACTGGGATAATCTTCTTGACCACAGGCCTGGAGAGATATGTCGAAAGAGATGGAACCAAAT
GGTTCTTCACATAGGTAAACTTAAAAACAGCTCATTTGCTGAACAAATAGAAGTTCTAGCTAAGAGATACCGCCCGGATT
TCGCCGAAGTAAGAGAGGCTTGGGACAATAAACCAGTCGTTCCATGA

>CcMYBR11 |Locus_ID=BC332_32616 |CDS_len=1743 |strand=-
ATGGAGCGTTACCTCTTCAAACGCCGCGCCGACGAGAAAGAAAGAAAAAAACATGGAAAACATTCTGAGAAGTTTAGTGA
AGACTGCGATGAGGTTGCTTTGAGAATTGTTGAAGGAAAGAAGAGTAGGAAGTTGAAGAAAAGCCGCGTGGCTGAGCAGG
AGAAAATGCATGATACTGAGGATGTACAGGAAGATATTGTTGCTGAAGTCGACGAAGGTGATATTTCTTCGCCTATCAAG
ATTGAAGAGAAAAACAGAACAGACAATGGGAATATTAAAAAGAGGAAAAAGGTAAAATCAGGACATAGCTCTGAAGATCC
TACGCATGAGATTGAAGAAAAAAACAGAACAGACAATGGGAATATTAAAAAGAGGAAAAAGGTAAAATCAGGACACAGCT
CTGAAGATCCTACGCATGAGATTGAAGAANNNNNNNNNNNNNNNNNNNNNNNNNNNNNNNNNNNNNNNNNNNNNNNNNNNN
NNNNNNNNNNNNNNNNNNNNNNNNNNNGAAGAAGGGCTGAAAAAGATTTTAAATTCTAGATCTTATCCTGAAATAAGGGG
TTGCTGGAAAGAAATAGGGAGTGCTATACCATACAGGCCTTATACTGCAGTTTATTATCGTGCACAGATCTTGTTTCGAA
GGAGTGAATCACGTAAATGGACTGAAGAAGAGTATGAAATTGTACGAAAATTCCAGAAAGAGCATGGGAACAATTGGAAG
GTCTTAGCTGATGAACTTGGAAAACATCGGTTTCATGTGAAGGATACATGGCGAAGGATAAAACGGCCAAATCAGAATAA
AGGACAATGGACTCAGGAGGAGTACCAGTCTTTGTTTGATTTAGTAAACACCGATCTGAGACTGAGGCTTTCTGAAGAGA
AGAGATCTAAGTATGGGATGTTACGGGATAATATTGCATGGACTGCCATAAGTGACAAATTGTCCACGAGAACTGAGGCA
AATTGCTGCTTGAAATGGTATGATCAATTAACATCACCCATGGTGGCCAAAGGTGAATGGGCGGATGTTGATGACTATCG
CCTAGTTGATGCACTTTTTGAGCTGGACGCAAGCTGCATAGAAGATGTGGACTGGGATAATCTTCTTGACCACAGGCCTG
GAGAGATATGTCGAAAGAGATGGAACCAAATGGTTCTTCACATAGGTAAACTTAAAAACAGCTCATTTGCTGAACAAATA
GAAGTTCTAGCTAAGAGATACCGCCCGGATTTCGCCGAATTTAGTGAAGACTGCGATGAGGTTGCTTTGAGAATTGTTGA
AGGAAAGAAGAGTAGGAAGTTGAAGAAAAGCCGCGTGGCTGAGCAGGAGAAAATGCATGATACTGAGGATGTACAGGAAG
ATATTGTTGCTGAAGTCGACGAAGGTGATATTTCTTCGCCTATCAAGATTGAAGAGAAAAACAGAACAGACAATGGGAAT
ATTAAAAAGAGGAAAAAGGTAAAATCAGGACATAGCTCTGAAGATCCTACGCATGAGATTGAAGAAAAAAACAGAACAGA
CAATGGGAATATTAAAAAGAGGAAAAAGGTAAAATCAGGACACAGCTCTGAAGATCCTACGCATGAGATTGAAGAAGAAA
ACAGAANTCAGGAACAGAAGCATACCATACACTGGAAAAACAGTAGAAACATGAGGAACCGAAGAGGAATACTAGCGGAT
TTGGACCAAGGAAAATTATCTTTAAAAAGGAACATCAGGCGGGCTCCTGACTTGGTATTTTAG

>CcMYBR12 |Locus_ID=BC332_30379 |CDS_len=5025 |strand=-
ATGCCGCCAGAGCCATTGCCTTGGGATCGGAAGGACTTCTTCAAGGAGAGGAGGCACGATCGGTCAGAAGTGTTGCCACG
GTGGAGGGAGCCTGCATCGCACCACCACTATACTTCTTCTCGTTGGCTTCCTGATTTTCGCTCTAGAGCCCCTCCTCCTG
GTCATGGTAAGCAGGGCAGTTGGCAAATGTATCCTGAAGAACTCGGACATGGTTTCATGACTTCTCGTTCGAACGAAAAG
ATTGTTGAAGATGAGAGCTGTCGGCAGTCTCGTGGTGATGCTGGCAAGTATGGAAGGAATAGCAGCAGCAGCAGGGAAAA
TAGGTCCTTTGGTCAGAGGGATTGGAGAGGTGGTGGTGGTGCTCACTCTTGGGAAGCTGCATCCCCCAGTGGTTCTGGTT
CTGTAAGACAAAATGATGCTACAAATAATCAGAGGTCATTGGATGTTATGGAGCCTCACTCTGAACATGTTAATACATGT
GATCAGTCTCACTCTAGAGACCAGCACAATAAGAGTGCTACTGCTAATGCAACAGCAAGCACGGGGCAAAGATTTGAGAG
AGAGACCTCGCTGGGGTCAATTGAATGGAGACCACTCAAATGGACTCGGTCTGGAAGTTTATCATCAAGGGGATCATTAA
GTCATTCTGGTAGCATGGGAGTGGATTCTAATGAGACAAAGCCTGAGTTGCGGCTTGGAAACTCAAAAGCCGTTCAATCT
CTTACAGGGGATGCAACTGCCTGTGTAACTTCTGCTGCCCCTTCTGAAGAGACAACTTCCAGGAAGAAGCCGCGCCTGGG
TTGGGGAGAAGGACTGGCCAAGTACGAGAAGAAGAAAGTTGAAGGTCCTGAGGATAATGCTGTTAAGGTGGGAACTTCTA
TCTCTGGTGATAGTGTAGAACCTAGTCATTCTCAGCTCTTGAACCAGGCAGATAGAAGCCCAAGAGTTGCTGTTTTCCCA
GATTGTCAATCTCCTGCAACTCCTTCGTCGGTTGCTTGTAGTTCTTCACCAGGTCTTGAGGATAAACAACTAGTCAAGGC
AACAAACATTGATCAGGATGTTGGTAATTTATGTGGCTCACCCAACGTTGTCTCCCAATATTACGCTGAGGGATCAGGTT
TTAACTTAGAGAATTGGGATCTTGCTCAGATTTCCAATTTGAACTCTTCAATCAATAAACTGCTACAATCAGAGGACCCA
AGTTCTGTGGACTCTGGTTTTATGAGATCAACTGCAGTGAACAAGTTGCTAGTGTGGAAGAGTGATATTACTAAGGCCCT
AGAAAAAACTGAAGCGGAGATTGATTCACTTGAGAATGAACTGAAGACATTGATCTCTGGACCTAAAAATACCCAGCTTG
TTCCATCTGCTTCCTGTTCTCCACCAAAAGATTGCAATGCAAATTCTCATGAAGATCAAGGCGCTACTTCTAATATAGCT
TCTGGACCTGCTCCTTTGGTAGTTGATATACCTGACGATTTGGTTGGAGAGGAAGGGGCTAATATACATGAAAATGAGCA
GACCGAGGTAAAAGTTGAGGACATTGATAGCCCTGGTAGTGCAACTTCAAAGTTTGTTGAATTGCCCTCAGAGAAAGATG
TGGCACCTGTTGATGTCTTGAAACATGGTGGAATATTTATTTCAGATGACAGCAAGTCTAGAAGATTGAATGTCAATATG
TGCAGTTTCACCGAGGAGAGCGCTAAATCTAGAAGTTCAGATGTAAAACTGTGCAGCTTTAACGAGGAAAAAGCAAGGGA
CACACTTGTTTGCTGGGAGCGCTCCCAGCCAACTGCCGGTTATTCTTACTCTGCTTCCAATGGCATTTTGAATGGTGGAG
AAGATGCTTTATATAATTTGATATTAGCTGCCAACAAAGATTCTGCACGGAGAGCATTTGAAGTATTTAAGAACCTACTG
CCTGCCAGCAAATGCAGTTTTGATTTTTCAAGAACTGTCAGAGTCTCTTTGTTGCAAATTGATCATGCGGTTGAAGAAAG

ATTTGCTAAGAGAAAGCAGTTTCAACAGTTCAAAGAAAAGATAATTGCCTTAAAGTTTAGGGTACATCAGCACCTTTGGA
AGGAAGATATTCGCATGCTTTCTGTAAGGAGGTTTCGTGCAAAGTCTCAGAAGAAGTTTGACTTGAGCCTGCGCCCTGTG
CAGATAGGACATCAAAAACACCGTGCTACAGTTCGTTCACGATTTTCTACCACTGTTGGAAACTTAAGTCTGGTGCCCTC
ATCAGAAATATTGAACTTCGCAAGCAGGCTACTCTCAGAACTTGGGGCAAAGGTCTACAGGAACACATTAAGGATGCCTG
CTTTAATTTTAGATCAGAAGGAGAGGATGATGACAAGGTTCATTTCAAAGAATAGTCTGGTGGAAGATCCCTGCGCTGTT
GAGGAAGAAAGGGGTCTGATCAATCCATGGACATCTAAGGAGAGAGAAATTTTTATTGAAAAGCTTGCTACCTTTGGGAA
AGACTTCAGGAAGATAGCTTCTTTTCTTGATCACAAAACAACTGCTGATTGCATAGAGTTCTATTACAAGAATCACAAAT
CAGATTGTTTTGAGAGAACAAGAAGGAAACCAGATTATTCTAAACAAGCGAAAGTTTGTTCCGCAAATAGGTACCTTGTT
GCTTCTTCTGGGAAAAGATGGAACCGTGAGGCTAATTCTGCTTCTCTCGATATCTTAGGTGCTGCATCAGCAATTGCAGC
TAATGTGGAGGACAGTATTGAAATTCGGCCTAAGGGTACGTCAAAGTACACCGTTCGAATGATCACTGAATACAAGGCGT
CCAGACTTAATGAGTTAGAGAGGTCAAACAGTCTTGATGTATGCCATAGTGAAAGAGAAACAGTGGCTGTTGATGTTTTG
GCTGGTATCTGTGGTTCCTTATCATCAGAAGCTATGAGTTCTTGCATTACAAGTTCAGTTGATCCTGGAGAAGGAAACCA
GGAATGGAAGCACCAGAAAGTGGGTTCGTCGACCAGATTGCCCCGGACTCCAGAGGTTACCCAGAGTATGGATGATGAAA
CCTGTTCAGATGAGAGCTGTGGGGAGATGGATCCTACTGATTGGACAGATGAAGAGAAATCAGCCTTCATCCAGGCTGTG
TCAGCTTATGGAAAAGATTTTATTACGGTCTCAAGATGTGTCGGAACGAGATCCCGTGATCAGTGCAAGATCTTCTTTAG
CAAGGCTAGGAAGTGTCTTGGACTAGATAAGATTCTTCCTGTACCTGGCAATTTGGAGAGGCAGGATGTTAACGGTGGAA
GTGATCCTGATGCTTGTTCTATGGAGACTAAGCTGTTGTGTAATGGAAAATCAAGTTTGATGTTGGAAGATGTATCTGAT
TTATGTATGAAGGTGGGGATTTCAAATCACAATTTGACCAGTTCTGATGAAAAAGATGGGGCTGGAGAACTGGATTCTGT
AGATATTCTTCAGGTAAACTGTGATGTAGATAAGCAAGAGGTGGAATTTAACAGGGATTGTGATCAGATTGGTGCTTGTA
TTGGAAATGGTAGAGGGGATGAGAATATGGTTATAGTTTCTCAGGAAGGTGTTGAAATTGGTGGAGATGCCTCGGAGAAT
GGCCTGCTTGACATACCGTGTCCGAATGAAGTTTCTGCTAAGCATTTAGGTGAAGAAATTTCTTCGCCTGAGCATGATTT
GAAAGATAGAAAGGCAGAAATAGCGGAAGTAGGTAGGTCAAACTGCTCTTTGGAAGATAGAAAGCCCAATATGGTGTTGT
CGGGAAATAATTCTGGTCTTGCTGCTCCTAGGGGAGGGGGATTGTGCCCTCTTAATGGATTCCAGAATATGACTCATTTG
GAATCTGATTCTGAGTGTAAGCTTTACTTGGAAAGCAATATTTCAGTTCAGAGTAAACAGATGCCTGAAGGTGCTAATGC
TGATAAATTATCAGAAGAACTTGAGAATGTTTGTGATGAGCAGTGTGAGAATGCTACTCAGTCGGCTGAGCTGCCTTTGC
CGAGCCATTCCCGGTTGGCCCAGGTTGAATCCTGCCAGATTCTTGGCAGCTATTCAGTGGGGGAGTCTGCTCTCACACAG
TTTGGAGATCCTGGCTGCAGAACATCAGCTGCATTGCAAGAAATCCAGAAGGTAGGTAGGAATCTACAGTTAGATACATT
TTCTACAACTGGTTGCTTTCTCCAGAAGTGCAATGGTACAAATCGTGGTGGTGGTTCTGTTTCTGATGTTCTGATTCCTA
ACCGAGAACAGACAGCAGGTAGTTCATCAAACTTGGAGAAACATTGTAGGAATGGTGATGTAAAATTGTTCGGACAGATT
CTCAGTAAACCATGCCCTCAGGCAAATCCCAGTTCCAATGCACATCGCAGTGATGGTAGCAATCAGCAGTTGAAAATTGG
TAGCGATTCCTTTAGTGCAAGTCATATATTAGATGGCAATTCAGCCACTGCCAAATTTGAGCGTAATAACTTCCCGTGTT
CTGAGACTCATCAATTGAGAAGTTTTGGTTTTTGGGATGGGAATAGAATACAGACAGGGTTTTCTTCGTTGCCCGACTCT
GCTATCCTCCTGGCAAAATATCCTGCAGCATTTGGTAATTATGGAATATCTTCCACCAAAATGGAACAGCCTACTTTGCA
TGGGGCTGTGAAGATGGCTGAGCGGAATTTGAATGGTGTCCCTGGGTTTCCAACAAATGCAAGTGGCAACAATGGAGTAG
CAGCAGCAGATTATCAAGTGTACAGAAATCGAGATGTGCAGCCTTTTACTATAGAAATGAAACAGAGGCAAGAGGCGTTC
TCTGAGATGCAAAGAAAAAAATGGGTTTGATGTTGTGTCTGGTGTGCAGCAACAAGCGAGGGGTGTGCTTGTTGGAAGAGG
AGGGATTCTGCAGTGCACTGGTGTCTCTGATCCGGTGGCTGCTATCAAAATGCATTATGCCAAGGCTGAACAATTTAGTG
GGCAAACGGCAAGCATCATCAGAGAGGATGATTCTTGGAGAAGTAAAGGGGACGTCAGCAGGTAG

>CcMYBR13 |Locus_ID=BC332_17457 |CDS_len=1395 |strand=+
ATGGGTATCGACACCCATATTTCCCAATGGATTCTCGAATTCATTCTCCGACAACCACTCGACGACAACATTCTCAACGA
TCTCATTAACGTTCTTCCACTCCCGAACGACAATTTGAATCTCAAAAAGGCGCTGCTTGTCAGAAAAATCGAATCCGAGA
TTGCTAACGGTTCGGTTACTGAGAAAATCCTCGAGTTAATGGAATTAATCGAGGAATTGGATCATCAAGAAGGAATAATT
AGGGATTCAGTGGCTACGAAAGCCGCTTATTGTGCGGTGGCAGTGGAGTGTACGGTTAAGTTTTTGAAAAGTGAAAGGGC
GGGAAGTGATAAAGGGAAGTATTTTGAAGCAGTAAGGAGGATATGGAAACGGAGGATTAATTTAATAGAGAAAGTGGAGA
ATGTAGGCGTTTTATCGGAGGAGCTGTGGAGTTGGAGGGATGAAATAGAGGCGGCGTTATGGGAAGATAGGTGTGCTGAG
AATGTGATAAGGAGAAGTAAAGGCGTTTTTGCTGTTGACGTGGTTAATGCTTTTGTTAGAGAAGCTAAAGAGAGAATTGG
GGCTCCTTTTCTTGAAGTTGTCGCGGAAACTTACCATACTGATGAAGCAGTAAAGTCAGTTTTCGGAGAGGTGAACAAAG
GAGGGGCTAGTAAGAAAAATGGAAAAGAGGTATGCAAAGGAAATGCATTGCCCAGGAAGAAACATGTTGCTTTCAAACGC
ACCAGAGGAGCATCAGGTATCAGGATCAGTGATTCTATTGAGTCGGAAGTTGAGGCATCTGGTGGACAGGATGATTCTTC
AGCTGAAGTGAAGATAGCTAAGAAAGCACTTAAATTGAGTTCATTGGAGCTGCGTGCTATGGTGAAGGATCCTCTACCTG
ATGCACTACGTTTTGCTGAGACTTTGTGTTCTGTGGTAAGGGATAATATGGGTCATCAGCCTGCTGAAAATAACAGTGAC
ATGGCCCCTCAACCAATGGTTTCCAGTAGCAGAATGGCTCAAGCCGGTGGGGAGAACCGTGAGGCACAGCAAAATTGTCA
TCATGCTGCTGCATCCAAGCCAGATCTAGCGAACCGAAGCAGCGCTGCCCATACATTTGAGTGGGACTCTTTCTTGGATG
ATATAAATGAAGGATCACCAAGTGGTGTGAATAGGGTTACTTTACCTAGCCCAAAGAGGACAAAAGTTTCTCCCCTAAAG
AAGTATGAATTTAAAAAAATCACCACGAGGAGAAAGCCCGTGAAATGGAGTGCGCTGGAAGAAGACACTTTGAGAACTGG
TGTACAGAAGTATGGTATTGGAAACTGGAAGGTCATCTTAGATGCTTATCGTGACATTTTTACCACGAGACAATCGGGTG
ACTTGAAGGACAAGTGGAGAAATATGATATCGTAG

>CcMYBR3 |Locus_ID=BC332_15220 |CDS_len=1137 |strand=-
ATGGAAGGAGGAAGCAGTAGTACAGAAGAATCAAAGTTGAGCCCCAAAAACCAAATTGAATTCGGAGAAGAGGAAGAGGA
GGAGGACGATCTCGAGACCGAAGAAAAAAGCAAAGCTCAAGGTACAACAAGAACCTGCTCTAGCAACAGCACTGTGGAAG

AGAACGGAAAAAAGACCAATTCAGGAAGTGTACGGCAGTATGTACGTTCCAAAACTCCGAGGCTACGGTGGACGCCTGAG
CTCCATCTTCGCTTTGCTCATGCTGTTGAAAGATTAGGAGGCCAAGATAGAGCCACACCAAAATTGGTTCTTCAATTAAT
GAACATTAAAGGCCTCAGCATAGCGCATGTCAAGAGCCATCTTCAGATGTACAGAAGCAAGAAAACTGATGACCCCAGTC
AAACAGTTTCTACGGACGGAAGAACCTTACTTGAGAACGGAGATCATCATATTTTCAATCTCACCCAACTTCCTAGGCTA
CAAGGGTATAACCAAACTTCTAGTTCAAGCTTAAGGTATGAGAATGCTTTATGGAGTCGTCAACCTAATTCTATGTACAA
TTCTTATAACGTGAATATTGGAGGTGGCTCCCGTACAAGTACAAGACATGGGTTTGCAAGCCACATCAATGGATACTCAT
CAGATGGACAATTATTTCCATGGAATAAACCCATTGATACTGAGAAAAAACAAAACCGGCCTCAATTCCTTCAAAATCAA
AGATTTTGGCCGACTCAAATTGGAATAAGCTCCGCAAAACAGAATCTCCTAATGACATTACAATTCAATAGAAATACAGT
TGAGACAGCCACACAATTCAACAGGAGAGAGCAAGAAATGATGACACAAGTGCGGGCTAATATATCTCTTTCTTCTTCAA
GGGGAAAAGGATGGACGAGTGAGGAGGTTGCAGGAACAATAACAAGCACATCCAATAAAAGAAAGATTCAAGATTCAGAA
ATCAATCTTGATTTGAACCTTTCACTGAAAGCAACAAGAGAGGATCATGATGATCATTTGAAAAGATTAAAAGCTGAACA
AGTTGGGGATCTTCTTTCTCTTTCTTTGTACTCATCAAGTACCAATGATCAGGAAAATAATGGAAAGAAGGCAAATACCC
TAGATCTGACTCTTTGA

>CcMYBR4 |Locus_ID=BC332_24070 |CDS_len=837 |strand=-
ATGTTGGAGGATAGTGATGAGACTGGATGCAATTCCAAGACAAGTTCTAATTTTAAGAAAAATGAGGATGAAACGGAGGA
GAACGGTTCGAGCAAGTTTAAGGATGGAGGAAGCTCAAGCGACAGTACACTTGAAGAGAGCGAAAAGAAGCCCTATGTGA
GACCTTACGTTCGATCCAAGATGGCTAGACTCCGCTGGACACCTGATCTCCATCTCCGTTTTGTTCATGCTATGGAAAGA
CTTGGTGGACAAGATCGAGCAACACCAAAGCTAATTCTTCAAATGATGAACACCAATGGACTTAGCATTGCTCATGTCAA
GAGCCATTTGCAGATGTATAGAAGCAAGAAGATGGATGGCCAAAGTCAAGGGATTGGACACCATCGCAAGCTTTTTATGG
AAGGTGGAGATCCAAATATTTTCAACTTGATCCGATTCCCAATGTTCCCTGCTTATTATCAGAGGCTCAATTCAACTTTT
AGGTATGGAGATGCTTCTTGGAATTGTCATGGTAATTGGATGCCTACAATGGGGCAAACTACACTAACCGGGACAGGAAG
AGGATTGTACACTACACTGAATCATTTATGTACAAGGATTCCCTCTTTTCCTGAAATTAAAGATGTACTGGGATCATCCA
GAGGAAACCCTAGTGATGACCTCACTAGGTTGTTTAACATAGCTGCAAAATCTCAAGCAAGGGCTTTTGATGGAAATGGA
ATTGCTTCTTCTCCAGATTTGGAAAGAGCCATAACTCCTTTGAAAAGGAAAGTTTCAGAGTGTGACCTTGATTTGAATTT
ACACCTAGGGGGCTACGAGGTTTATAGAAGATGCTAA

>CcMYBR5 |Locus_ID=BC332_10237 |CDS_len=1053 |strand=+
ATGGAACATGGCAATGGAGAAACAAGTGAGGAATATTACATGGAAAAGGTCATGTCATCCATTGATTTGAATGAAGAAGT
TGATCATGATCATAGCAAAATTCAACAAATTGATGATGAAGCTATTGAAGTTATTAGTGTGGAAAGTGATAAAATTACAA
ATGAAGAAAATAATAGTAGTTCATGTGGAAATGAAATCAATATTAAGAACAAGATGAGCAAAGTTAGACAATATGTAAGA
TCAAAATTGCCAAGGCTAAGATGGACTCCACAACTTCATCTCTCTTTTGTTAATGCCATTCATAGACTTGGTGGTCAAGA
AAGAGCAACACCAAAATTGGTTCTTCAGATGATGAATGTGAGAGGACTCAGCATTGCCCATGTTAAAAGTCATCTCCAGA
TGTATAGAAGCAAGAAACTTGGAGAGTTTGGACAAGTGTTAGGCCATGGTCATAGAGCAAATCAAAGAAGAAATTATCTC
TATGGAGATTTAAGCCAAAGATCTTTTAATCCTCTTCAAGATTTCAAAATGAAGAATGGTGCAATTGTATTAGCAAGAAA
TTTTAATGATGATGACAACATTAATCACCATTTTCAAAATTCATTTGCTAGAACTCAATCCAGATACATGGAATGGTCTT
CCCATCAAGGAACCAACAATTTATTGAAAATGAAAATCTTGGAAAATCATCAAATTTGTCCTATTAGGCAAAGACATTAT
TTTCTTGAAGAAAAAAAGTGGCCTCCATTTGAATTCAATAAAAATAAATTGAATTCTCAATCTTTGTTTCATCATACTGG
ACCAAAATGGAGTTGCAGATATAACAACCTTGATCCAAAATATAAGACCCCTCTTAGATTTGAGATGAAACAAGACAAGA
AGTTCATTTTGGAAAAAGATTATTGGCTGCCTGATTTGCAATTGGGATTGAGCAGAAGTGAAGAAAATAATATGAAGGAG
AAAATTATTCACCATAGTAATAAAGTGGAATTATCAGATATAAACACTATGCTTTCTCTTTCTTTGCCTTCTTATTCATC
AAGTGCAACCTAA

>CcMYBR6 |Locus_ID=BC332_04479 |CDS_len=1149 |strand=+
ATGGCTGAAAATTTAGTGAAACAAGACCATGAAGAGATGATGATGATTATGGAAAACTCATCCTCCTCGTCCATTGATTT
GAACGAAGACAACAAAGGGGAAAGCGAAGAAGAAGAAGAAGAAGAAGTCATTGAGGTGGAAGATAGTAGTGAGAAAGAAA
CAGAAAGAATAAGTAATTATAATAGTTGTGATGTTGAAAAGAAAAACGTAAGGCAATACGTTCGATCGAAATTGCCGAGG
CTCCGTTGGACTCCAGAGCTCCATCGCTCCTTTGTTCAAGCCATTCAAAGACTAGGTGGTCAAGAAAGAGCCACACCTAA
GTTGGTTCTCCAAATGATGAATGTGAGAGGTCTAAGTATCGCCCATGTAAAAAGTCATCTACAGATGTATCGAAGCAAGA
AACTAGATGAATCTGGACAAGTGTTAGGTCGAGGTAATAATAGGGGAATGCAAGGAAGAGGCTATTTCTTTAGAAACCAC
CTAGGTGGTCAAAGATATAACCCTATACAAGATTTCAAGATGAAGAATGGTGCCATTGTATTAGCAAGGAATTTCAACTA
TGATGACCATGCACTTAAAGGCCGTTTTCGAAATTCCTTTTCGAGACCACCATATCAGTCCAAAGGCATCATTTCCAGGT
ACATGCAGTGGTCTTCCGATAATCAAGGAGGCCTATGTAATACTACCAAGTCTCTGCAAGGTGAAGATTTGTTGAGAATG
AAGAGCTGGCAGACACCAAGACAAGCAGTAATTGAAGAAAATGGAATACATGAAATTGTCCCAATTAGGTCAACCCAATT
TCTTGAACAGAAAAAGTGGCCTCCATTCATCCCCAATCAGTGGGAGGAGAAAAGGGCTGATTTTTGTTCAGCTAAAAACT
CCCATTTTTTGCTTCAACAAAATTTGGGACAACCATATTCCAAGTGGAAGTACTGCAGAAACAATAATGCCCTCGAGCAA
AATCTTGTGACACCTTTCACGTTTGAGATGAAACAAGACAAGAGTCCCTTGGAGGAAAAAGACTGGTTACCTGATTTGCA
ACTAAGATTAAGCAGAAGCACAGAGGACAAGAATGAGAGCAATAGGAGGGATCAATCAGATATAAACACAATGCTTTCTC
TTTCACTGCCCACTTACTCAGCAACCTAG

>CcMYBR7 |Locus_ID=BC332_05115 |CDS_len=759 |strand=+
ATGATGAAGGAAAATTCTTCCAAAAATTCTCCTTGTAATTTATCCATGAGACTTGGTCACGTGGAGCAGCAAATATTAGA

AAATAGCAATAAATCATCATCAGGGGTAAGACCATATGTTAGATCGAAAATGCCGAGACTCCGATGGACGCAAGATCTTC
ATCGCAACTTTGTGCATGCTGTTGAGCGACTTGGTGGAGAAGATCGAGCAACTCCAAAGATGGTGCTACAGCTGATGGAT
GTGAAGGGACTCACCATATCTCACGTTAAGAGTCACCTTCAGATGTACAGAAGCATGAAACATGAACAGATGATGCAAGC
TGAAGCAGAAGCTGCAAATGGGATTAAGAGGAATAGGATGGATGGTCCAGATCAACTGAATTATCTTAATTACTATGATG
GCAAAGCGCTGTTAGATGGGCATCCTAATTCGAAAGTAACGATTAACTACTTCAACTTAATTGCCTCTACTCCTAACTTT
TCTCCTGCTCAATGGAAAGATATGCAAGAGAAGATGATGGGATTGGAAGGACAATCTAAACCAGCTTGCAACATGTTCAG
GGATTTCTTCAATGGCTGTACTACTGTTCAAGATGGGAATTATAATATTGGAGAATGTGGTAGCAGTTTGTCAAATAACC
GTTGTTCAATAATTGACACTGAAGAAGAGGCCCAAGACTCCAGCAGCACTATGTCTTTAGAACCATCTGCCTGTTTAGAT
CATGGGACTAATATCTCTCTTGACCTTACCCTTGGTTAA

>CcMYBR8 |Locus_ID=BC332_11027 |CDS_len=453 |strand=-
ATGGCTATGGGGAGGAGGAACTGCACCTTGTGTGGTGGGAATGGGCATAATCAAAGAACGTGTATTGAGAAAGGCAAAAG
CATCAAATTATTTGGTGTGGAGATCACTACTTCTACTGTTGCTGCAATGTCTAAGATAGGAAGAAAAATCAAGAAAGGAA
ATCCATGGACAGAGGATGAACAAATAGCATTCTTGAAAGGATTGAAGTTTCATGGGAAAGGCAATTGGGCTAAAATTGCG
AAGGATTTCTTGCCTAGTAGAACATCAACACAAATTGCTAGTCATGCTCAGAAGCACTTCATGAGATTGGATGCTAATTC
CAGTGAGAGAAAATATCTCAAGAAGTCAAGTGTTTTTGACCTTCATCTTGAAAAAACAGAGGACAGGGAACATGCTATTG
TTCCAGTGGAAACTCATCATGTTCCAAGTTTTCTTTCCAATTACAATAGATGA

>CcMYBR9 |Locus_ID=BC332_11026 |CDS_len=606 |strand=+
ATGTTTAGAAAAGATTACAGAAGAAAAATCAAGAAAGCAAATCCATGGACAGAGGATGAACAAATAGCATTTTTGAAGGG
ATTGAAGCTTCATGGGAAAGGTAATTGGGCTAATATTGCTAAAGATTTCTTGCCCAATAGAACATCAACACAAATTGCTA
GTCATTCTCAGAAGCACTTCATGAGATTGGATGTTAATTCAAATGAGAGAAAACATCACAAGAAGTCTAGTGTTTTCGAC
ATTCATCTTAAGAAAATAGAGGACATTGAACATGCTATTGTTCCACTAGAAAATAATCAAGAATCTCTTCATGTTCCAAT
TTTTCTTCCCAATTTCAATTTGACCAAAAGAATCCAAGTACCCAAAGTAACGCCTGTTACTTGGGTTTACATGTATCCAT
ATCATCAGTACACCTCTACATCAGCCAAACTTCTTGAGACAATATTTTGCTTCTTAGATTTTCAAGATATTAAGGAATCC
CCAATTTTGATAAGAGACCCTGTGATTGATTTTTTTGTTAAGGGACAAGCTGTCCAATCTGCACCACTAAGTTCAGTGAT
AAAGGTTATAGGTTCACTTGACAAAAGTATTCCTTGTCCTGGTTAA

>CcMYR1 |Locus_ID=BC332_26464 |CDS_len=1248 |strand=-
ATGTACCATCATCACCACCAAGCTACCAACATGCACTCTTCGACAAGAATGTCTTTCCCAGAAAGGCATCTGTTCCTGCA
AGGCGGGAATGCCAACGGAGACTCAGGACTTGTTCTTTCAACTGATGCTAAGCCAAGATTAAAATGGACGCCAGACCTTC
ATGAGAGGTTTATAGAAGCAGTTAACCAACTTGGTGGGGCAGACAAAGCCACGCCAAAATCAGTTCTCAAACTTATGGGG
ATCCAGGGATTAACCTTGTACCACTTAAAGAGCCATCTTCAGAAATACAGACTAAGTAAGAACCACCATGGACAAGCAAA
TTCTAGCAGGGACAATAAAGCTGCAGCAAGTATGGAGAAAATATGTGAGAGCACTGGAAGTCCTACGAGCAATCCAAGCA
TTGCACCCCAACCAAACAACAATATACCAATAAGCGAAGCAATACAAATGCAAATTGAGGTCCAAAGAAGGCTTCATGAG
CAGCTTGAGGTGCAACGACATTTACAGCTAAGGATAGAGGCTCAAGGGAAATATTTGCAGGCTGTGCTCGAGAAAGCACA
AGAAACTCTTGGAACACAAAACTTGGGAACAATTGGATTAGAGGCAGCCAAGGTTCAACTATCGGATTTGGTATCCAAAG
TATCCAACCAATGTCTAAATTCTGCATTTTCGGAAATACAAGAACTTTCAGGATTTCACACCCCACAAACACAAGCAACC
CAACGGTTAGCAGATTGTTCACTGGATAGCTGCTTAACCTCCTCTGAAGGCCCCTTGAGAGACCTGCAGGAAATGCATAA
TAACCAAGTTGGGCTGAGGACTTTAAACTTTGGACCATGTACAGAAGATATTGAAAATCAGGCGAGGTTACATCGGACTG
ATCTAAGATGGCGTGACGACCTCAAGGAGAACAGATTGTTTCCCAAAATGGATGAGGATACAGAAAAAGAATTTGCCAAA
GAGACAAACTGGAGCAACTTATCAATGAACGTTGGAAGCCAAGGAGAAAATCGGAATGTTAACAGTAGCTACGTTGATGG
ACGACTTAATGGAATAGATGCAGACATCAAACTTTTTCACCAAGCTCCAATAGACAGAAGTGATTCAACGAAACCAGAGA
AGCAAGTGTCACCACAAGAGTATAAGCTGCCTTACTTTGCTCCGAAGCTGGACCTAAACACGGATGACCAAACTGATGCT
GCTTCTAGTTGCAAGCAACTTGACTTAAACGGTTTCAGTTGGAACTAA

>CcMYR2 |Locus_ID=BC332_26060 |CDS_len=1245 |strand=+
ATGTACCATCATCACCACCAGGATAATAGCATGCACCCTTCAACACGAATGTCTATCCCTGAAAGGCATCTGTTCCTGCA
AGGTGGAAATGGCAACGGAGATTCAGGGCTTGTCCTATCAACTGATGCTAAGCCAAGATTGAAATGGACGCCGGACCTCC
ATGAGAGGTTTATAGAAGCAGTTAACCAACTTGGTGGAGCAGACAAAGCCACTCCAAAATCAGTTCTAAAGCTCATGGGG
ATCCAAGGATTAACCTTGTACCACTTAAAGAGCCATCTTCAGAAATACAGACTAAGTAAGAATCTCCATGGACAAGCAAA
TGCTAGTGGAGCAAATAAAGCTGCAGCAGGAGTAGAAAGGATATCTGAGAATAGTGCAGCATGTATAAGTAATCCAAGCA
TGGGACCCCAACCAAACAAAAACATTCAAATAAGTGAAGCAATACAAATGCAAATAGAAGTGCAAAGAAGGCTTCATGAG
CAGCTTGAGGTACAACGACATTTACAGTTACGGATAGAAGCTCAAGGGAAATATTTGCAGTCCGTGCTGGAGAAGGCACA
AGAAACCCTTGGAAGACAAAACATGGAAACAGTCGGATTAGAGGCAGTGAAGGTTCAACTGTCAGAATTTGTATCCAAAG
CATCCAATCAATGCCTGAACTCTGCATTTCCAGATATAAAAGAACTATCAGGATTTCACAGCCAACGAACACAAGCTACA
CAGCCAACAGATCGTTCAATCGACAGCTGCTTGACCTCTCGTGATGGCTCTTTGAGGGACAATGCAATGCATGATAACCC
AATTGGATTGAGGCCTTTCGACTTTACACCATCTATGGAGTGTAAGGATATTGAAAATGACACCAGGATACAGCAGACAG
AACTACGATGGTGTGACAACCTCAAGGAGAACAGACGATTATTCTCCCCAGTGAACGAGGGTAGAGAAAAAGCATTCACC
AGAGAGACAAACTGCAATAACCTATCAATAAGTATAGGACTTCAAGACGAAAAACTGAATGGAAGCATGAACTACTCGGA
TGGAAACTTCAGCGGAACAGAGAGAGATGTTAAACTTTTTCACCAGGTAGCCAAAAGAAGTGAATCAGTGCCAGAGAGGC
AGAAATCATCGCAAGAGTATAAACTGTCATACTTTGAGCCTAAACTGGATCTAAACATGCATGATGAAACTGATGCTGCT

TCAAGCTGTAAGCAATTTGACTTAAATGGTTTCAGTTGGGGTTGA

>CcMYR3 |Locus_ID=BC332_27762 |CDS_len=1020 |strand=+
ATGAACTTAGTTTTGTCAAGTGATGCCAAGCCTAGACTAAAATGGACTCCTGATCTTCATCAACGATTCGTCGATGCAGT
CTCTCAACTAGGAGGTCCAGACAAGGCTACACCAAAATCATTGATGAGAGTGATGAACATTCATGGGCTAACTCTGTATC
ACCTCAAGAGCCATTTACAGAAATATCGTCTAGGAAAAAGTCAGGTGACTGATCAATCTTTTGATGAAAAGAAACAAGAA
GGTAAGCTGGAATTGTGTACAATAGCACATGATGATGATACAAAGGAAAATGAAATATCCAAAAGCTTAAATCAGAGGAC
TTATTTTGATGCAGAGAGTGATCAGTTGTCACTTGGAGTTTCATGTGATGGATCTCAGAATCAAATGAATGAGAGTTTGC
AAATAGCAAGAGCTTTACAAATGCAAATGGATGTACAGAGGAAACTTCATCAGCAGATTGAGGTACAAAGACATTTACAA
CTGAGGATTGAAGCACAAGGCAAGTATTTACAATCAGTTTTAAGGAAAGCACAGGAGACACTAGCAGGATATGGTACATC
TTCAGTTGGAGTTGAACTTGCTAAAGCAGAACTGTCTCAATTAGTTTCAATAGTTAACATGAGCTGTTGTCCAAGTACTT
CATTGTCAGCCATAACAGAAATTGATGGTTCAATCTCGAAAGACACACAGACGAAAAATTTGAAAGGCGGAATCTTGTCT
TCACTTGAAAGTTCTTTAACATCATATGAGAGCTCGGTAAGGAAGGAAGATCAAAATGATACGCGGAACAACAAGAATAC
TAATTGTATCGGTCTTTCATTGAATCAAGAAACTAAAGAAAGAAAAAGAAGTAGAAATAACATTTGTGATCATGATATGT
GTGTTGAACAACCATCTGGTAAAAGATTTTTGGAGACAATTGATTTGAACAGCAAGTGCCCAAATGAGTATGATGAAAAT
GGTCCAAAAGTTATAGACTTGAACTACAAGGAAGTAGAGCAATTCAATGAGTATAGTTAG

>CcPCL1 |Locus_ID=BC332_15022 |CDS_len=915 |strand=-
ATGGGTGAAGAAGTGAAAATCACCGACGGTGATGCTGCTTTCGCCGGAGATGATAACCGTGTTCTCGAATGGGAAGACGG
ACTTCCTAGTTTTGACGATCTGACGCCGTTATCTCAGCCGTTAATTCCGCCGGAATTAGCATCGGCGTTTAGAATCACGC
CGGAGCCGGCGAAGACGATGACCGATGTTCATCGTGCTTCGGAGAGTACGTTTTCTTCTCTCCGTGCTGGAGGAGGATTA
CATATGTTGTCTGATAAGTTTAATTTCAACGAAGGAGGAAGGAATGAGAATGATGAGGTGGATCTGACGCGGGATGGATC
GGATTCGAGGAAGACGAGGAGGATTGACCCGGAGATGGTTCCTGAAGAAGCTGATTCGGCATTGAGGAATGAAATGAATT
GTGGAGATGATAATTCAGCTAAGACATTGAAAAGGCCGCGGTTAGTATGGACACCGCAGCTGCATAAGAGATTTGTGGAT
GTGGTTGCGCATTTAGGGATTAAAAATGCTGTGCCGAAGACAATTATGCAGCTTATGAATGTGGAGGGATTGACGAGGGA
AAATGTTGCTAGTCATTTACAGAAGTATAGGTTGTATTTAAAGAGAATGCAAGGATTGTCCAATGAAGGACCGTCTTCGT
CTGACCATTTGTTTGCGTCCACTCCTGTGCCACAGAGTTTGCAGCAGTCTGGTGTTGGTAATGGTAATGGGAATAGTGGT
AATGGACATAGTAGTAATGGTCATATGCCTATGCCTATGCCAATGCCAATGATGTATCCACCACAAATGGTGCCAATGCC
TATGATGGGAATGTCAGGGCATGGGTATCATCATCAGTATAATATGGGGCAACCGCGAGATTGGTCGGGAAATAAATTTG
GTTCTTATCATCATGTTGCTCCTGGTGAAAAATAG

>CcPCL2 |Locus_ID=BC332_17321 |CDS_len=882 |strand=+
ATGGGTGAAGAAGTAGTTACAAAGCTTACCGACTATGATTCTAACGGCGTTAACGATGACAGACTTCTCTGGGAAGCTGG
ACTTCCCAGCGCCGATGATCTAACGCCGTTGACTTTGCAGTTAATTCCGTCGGAACTTGCTCATGCGTTCAGAATCTCGC
CGGAATCATCAAAGACTATGAACGATGTGAATCGCGCTTCGCAGAATACGTTTACTTCTCTACAGAGATGGCACTCACAG
GATATGGCTTCAATGAATAGAGAAGAAACTGTAACGGAAAGAGATGAAACGGATCTGGTAAGACAAGGATCCGACCCGAA
GAAGCTTCGGAGAGTTGAATCTGGAGGAACTGATGAGGCGGCTGATGCTGATTCTGCCTTGTGTAGCGAGAAATTTGAGA
CTGTAAAGCGGCCTAGGCTTGTGTGGACGCCGCAGTTACATAAGCGTTTTATTGAAGTGGTAGCGCATTTAGGTATTAAA
AATGCGGTGCCAAAGACGATTATGCAGCTTATGAATGTGGGAGGATTGACACGAGAAAATGTTGCAAGTCATTTACAGAA
GTACAGATTGTTTACGAAGAGAATGCAGTCGAACGAGGAGCCTTCTTCGTCGCCTGATCATTTGTGTACTTCCAGGGGGG
CGACGCCTCAGAGTTTGCGTGAGTCTAGTGAGAGTTGTCACCCGCACAATACTAATGGTCATATGGGAATGCCAACTATG
ATGCCGTACCAACCGCAAATGATGCCGATGCCAATGATGATGGGAATGACAAATGGAGGACATGTAGGCATGCCTGTTGG
TTACGCAGGTGGACCGCCAGCTGGATTTCATCATCCGTATAATTTTGGTTACTATCATCCTGTTGCTTCCAATGATAATT
AG

>CcPHR1 |Locus_ID=BC332_07350 |CDS_len=1386 |strand=+
ATGGAAGCACGTCCTGCTGTGTCTGTTCAAAATGTGGTTGCAAGTCAACTTAGTAATTGTGGTGCATCAGGGACACTTTC
TTCTTCCCTGTGTATGTTTCCAACAGCCCTAGGAGAGAAGTACCCTAAGTTTCCAGACTTGCAGCAGGCTTCAATGGAGA
AGGAACTCAAGCAACATCCTGGAACTGTTGCCTCTTCGTTGCCTTCCAACAGTGGGGCTGTTGGTCATATGTTCTCATCA
TCATCAGGATTTTCTGCAGATCTCCATTTCTCATCTGTTTCAACCCAGGAAAAACATTCAGGACCCACATCTTTCATTTC
TCAGTCAACATATAGTGAGACATCGATCACATTTCCTCATTCGGGAGTTCTTCAATCCACAGCATCAAGCCAATATTTGA
ATGAAAACCATGAACCCTGGTGTATAGATCCATTACCTAATTTCCTTGATTATTCAGTGGATACCCCTGTCCAGAATAGT
CAGGTAGCTTGTAGCGATAAACAAAGTGATTGGCAGGAATGGGCAGACCTTGTAATTAATGATGAAGATGCTCTAACTTC
TAATTGGAACGACATCATGGCTGATACAAGCATTGCCGACTTGGAGCTGAAGTATCAGGAGGGAAAACAACCTTTAAATT
TCCAAATGCAGCAAGTCCAAGAATCAGAGCAAATCCCGGCTGTGCCTGTAGAAAATTTAGTTATTGCTCCTGCGTCCTCC
CCTGCAAGTAGTGCTGCAACAAAGCAACGCATGCGTTGGACACCAGAACTTCATGAAGCCTTTGTCGAAGCAGTCAACAA
GCTTGGTGGAAGTGAAAGAGCTACTCCCAAAGGAGTGCTAAAATTGATGAAAGTTGAAGGTTTGACCATCTATCATGTAA
AAAGCCATTTGCAGAAATATCGAACAGCTAGATATAAACCAGAAGCATCAGAGGCAGGGTCTTCAGAGAAGAAACAATCT
TCACTAGATGATTTGTCATCTCTGGACTTAAAAACGGGAATTGAGATCACTGAGGCATTGCGGTTGCAGATGGAAGTTCA
GAAGCGTCTCCATGAACAGCTCGAGATTCAAAGAAATTTGCAGCTGCGTATAGAAGAACAGGGGCGATACCTCCAGATGA
TGTTTGAGAAGCAGTGCAAGTCAATGCCTGGTGTGGACTTGGCTAAAGGCTCATCATCCACGGCAGATGATGCCTTTGCA
CAGTTGTCAGATGCAGTTCAAAATTCCCCTGGCACAAACGAACCTGGAGTGTCAAAATTTGTTACTGCTAAAGAGGTTGA

TGAGAAACAGAAACTGCGGGAAAGGGAAGTTCTTGAAGATCCTGAGACGAATATAACCAGCCCATCTGATTCACCACCTT
TAAAGCGCTCTAAATTGGATGAATAA

>CcPHR2 |Locus_ID=BC332_14656 |CDS_len=1305 |strand=-
ATGGAGGCGCGTCCTGCGTTATCTGTTCAGAGGGAAGGTGCACATCCACTCAGTAATTGTGGAGCTCATTCTTCATTACC
CGTGCTTCGAGCAAGCCTGGAAGAGAAATATCCTAAGTTTCCAGGGCCTGTCCTTTCTTCGTTGCCTTCAAATAGTGGAG
CTGTTGGTCATTTGTTTTCCTCATCATCAGAATTCTCCACCAATCTTCACTTTTCATCTGTTCTGCCGCATGAAGAGCAT
TCCGCATCTGCACCTTTCATTCCTCAGGCAACAAATACTGAGGCATCAGTCCGATTAGCTGCATCAAGCCAGTATGTTAA
TGAAAATAGCGAACCCTGGTGTATAGATCCATTGTCTGAGTACCTTGATTACTCTTTAAATACCCCCGTCAAGAACACTC
AGCTAGATTGTAGCAATACAGGTGATTGTCACATCCCACCTGAGGATCTAAGTAAACAAAGTGATTGGCCTGAATGGGCT
AACCTTATAATGAATGATGATGATGCTCTTTCTAATTGGAACGAGATTATGGTTGACGCTGGCATTGTTGATGCAGCAGA
GCCGAAGATGCAATATCAGGAACAAAATCAACCTTCAAATTTCCCAGCACACCAAGGTCAACAATTGCAGCAAATTCCTA
GTGCATCTCTAGAAACATCTGGCATTGTTCCTGCATCATCGACTGCAAGTGGTGCTTCATCGAAGCAGCGGATGCGTTGG
ACACCAGAACTTCATGAAGCCTTTGTGGAAGCGGTTAACAAACTTGGTGGAAGTGAAAGAGCTACTCCCAAGGGTGTGTT
AAAGTTAATGAAAGTTGAAGGTTTGACCATCTATCATGTAAAAAGTCACTTGCAGAAATATCGAACAGCTAGATATAAAC
CAGAAGCATCAGAGGGATCTGAGAAGAAAGAGTCATCCATTGGCGATTTGTCATCTCTGGACTTAAAAACGGGTATTGAG
ATCACTGAAGCATTGCGGTTACAGATGGAAGTGCAGAAACAGCTGCATGAACAGCTTGAGATCCAAAGAAATCTGCAGCT
GCGAATAGAAGAACAAGGACGCTGCCTCCAAATGATGTTCGAGAAGCAATGCAAGTCAATGCCCGGTATTGATGCCTCCG
CTCAGCCGACAGATGCAGTTCAAAGTTCCTCAAACAAAAATGATCCAGGAGTATCACTGGTCGATCATCACGAAGCAGGA
GATAGAACTCAAAGGACGGTGGGGGAGAAGCAGAAAGAGAACGAAAAAGAAGATACTAGCTCATCAAATTCACCACCATC
GAAGCGTGCTAAAGTCGATGAATAA

>CcPHR3-1 |Locus_ID=BC332_14381 |CDS_len=1347 |strand=+
ATGAACATGCGCCGTGCATTATGTATTCAACGATCTAGTGAAGCTCAAAACGGTAACATGGGTGTTTCTGGAGCTATGTC
GACGTCTTCAAACCTCATTCCGTCACAGGCAATCCCGTCAGCATCTAAGAGTCGAACAGTAGGGCATCTATTATCTTCGA
CTTCTGAACCCCATAAAGATTTTAACTTTTCTTCTACCTCCTCTCAAGAAAGTAGGCAACGTAAATATCCATTTATTTCA
AGTGAAGCTTCTGCGGCAACCTGCCGATCTTCACTCTCAAGTATACATTCGACATCTTTGGATAATTACCCCATAGGGAA
CGACAGTAATTCCTCGGGCAAAGATGCATGTCATGACTTTATCAACTTTCCTGCGAATGATCTCGTTCAGAATGGCCAAG
TCGAAAGCCTTGCAGTTGTCATGCCATCTGACGACCAGGCTAAGAGATTTGATTGGCAAGAGTGGGCTGATCAGCTAATT
AATGACGATGATGATCGTTTGGGTTCGAGTTGGAGCGATATTCTTGTTGATATCAATCCTCCAGAAATTAAGTTTCTGGA
AACGTCTGAAGTCCTGGCTTGCGAGTCCCATATTCTTCCACCTCCTCCTTCTCCTGCTGCTGCCGCTGCTGCATCGTCTG
GGCAAATTTGCCCAGTTGGTAGCCCATCGTCTACCACAGCGCTGGCAAAGCCTCGCATGCGTTGGACACCTGAACTTCAC
GAAGTCTTTGTAGAAGCCATCAGTAAGCTTGGCGGTAGCGAAAAAGCTACACCAAAGGGAGTCTTGAAGATCATGAATGT
TGAAGGCCTAACCATCTATCATGTCAAAAGCCACTTGCAGAAATATAGAACTGCCAGATATAAACCAGAACCCTCGGAAG
GAACACCGGAAAAGAAGCCCACCAGTGTCATAGAGATGCCATCGTTGGACCTGAAAACGACTATGGGAATAACAGAAGCG
CTGAGACTGCAGATGGAAGTACAAAAGCAGCTTCATGAACAACTTGAGATCCAAAGAAAGTTGCAGTTGCGGATTGAAGA
ACAAGGTAAATACCTCGAAATGATGTTTGAGAGGACGAAGGACATTGGGAAAGATTTCAAGGTATCATCATCAATAACAG
ATGAACATCCTTCACCATCAACCGAGCCAAAGCATTCGCCTCCCAACGACAAATCAGAAGCACTCGAGAAAGATCCTGTT
AGTTTGAATGATCGTTCAAGTTCTGGAGGGAAATCACCGGACAAGAAAGCTCGTGAAGATCACGACACAGATGGTACAGA
CTCGTGTTGTTCACCTCCGTCAAAACGAGCAAGAACTGAGGAAAGGTCAGCTGTTCATGAATCTTGA

>CcPHR3-2 |Locus_ID=BC332_14380 |CDS_len=1179 |strand=+
ATGGGTGTTTCTGGAGCTACGTCGACATCTTCAAACCTCATTCCCTCACAGGCAATCCCGTCAGCATCTAAGAGTCGAAC
AGTAGGGCATCTATTATCTTTGACTTCTGGACCCCATAAAGATTTTAACTTTTGTTCTACCTCCTCTCAAGAAAGTAGGC
AACATAACTATCCATTTATTTCAAGTGAAGCTTCTGCGGCAACCTGCCGATCTTCACTCTCAAGTATACATTCGACATCA
TTTGATAATTGCCCCATAGGGAACGACAGTAATTCCTCGGGCAAAGATGCATATCATGACTTCATCAATTTTCCTACGAA
CGATCCTGTTCAGAATGGCCAAGTCGAAAGCCTTGCAGTTGTCATGCCATCTGACAACCAGGCTAAGAGATCTGATTGGC
AAGAATGGGCTGATCAGCTAATTAATGACGATGATGATGGTGGTTTGGGTTCGAGTTGGAACGATATTCTTGTTGATATC
AGTCCTCCAGAAATTAAGTTTCTGGAAACGTCTGAAGTCCTCGCTTGCGAGTCTCATATTCTTCCTTCTCCTGCTGCGGT
TGTTGCTGCTGCATCGTCTGGGCAAAGTTACCCAGTTGGTAGCCCATCGTCTACCACAGCGCTGACAAAGCCTCGCAAGC
GTTGGACACCTGAGCTACACGAAGTCTTTGTAGAAGCCGTCAGTAAGCTTGGCGGTAGCGAAAAAGCTACGCCAAAGGGA
GTCTTGAAGATCATGAACGTCGAAGGCCTAACCATCTATCACGTCAAAAGCCACTTGCAGAACTGCCAGATAAGATATAA
ACCAGAACCCTCGGAAGGAACATCGGAAAAGAAGTCCACCAGTGTGATAGAGATGCCATCGTTGGACCTGAAAACGACTA
TGGGAATAACAGAAGCGCTAAGACTGCAGATGGAAGCACAAAAGCAGCTTCATGAACAACTTGAGATCCAAAGAAAGTTG
CAGTTGCGGATTGAAGAACAAGGTAAATACCTCGAAATGATGTTTGAGAGGACGAAGGACATTGGGAAAGATTTCAAGGT
ATCATCATCAATAACAGATGAACATCCTTCACCATCAACCGAGCCAAAGCATTCGCCTCCCAACGACAAATCAGAAGCAC
TCGAGAAAGATCCTGTCAGTTTGAACGATCGTTCAATCACCGGACAAGAAAGCTCGTGA

>CcPHR4 |Locus_ID=BC332_14379 |CDS_len=300 |strand=+
ATGGGAATAACAGAAGCGCTAAGACTGCAGATGGAAGCACAAAAGCAGCTTCATGAACAACTTGAGATCCAAAGAAAGTT
GCAGTTGCGGATTGAAGAACAAGGTAAATACCTCGAAATGATGTTTGAGAGGACGAAGGACATTGGGAAAGATTTCAAGG
TATCATCATCAATAACAGATGAACATCCTTCACCATCAACCGAGCCAAAGCATTCGCCTCCCAACGACAAATCAGAAGCA

CTCGAGAAAGATCCTGTCAGTTTGAACGATCGTTCAATCACCGGACAAGAAAGCTCGTGA

>CcPHR5 |Locus_ID=BC332_14389 |CDS_len=1218 |strand=+
ATGGTTGCGTTTCCTGCCTCTCTAGTTTCTTCTGCATTTGAAATTCATGGAGATTGTAATCTGCGCATTGCAGGAACATC
GGAAAAGAAGTCTACCAGTGTGATAGAGATGCCATCGATGGACCTGAAAACGACTATGGGAATAACAGAAGCGCTAAGAT
TGCAGATGGAAGTCCAAAAGCAGCTTCATGAACAACTTGAGATCCAAAGAAAGTTGCAGTTGCCGATTGAAGAACAAGGT
AAATACCTCAAAATGATGTTTGAGAGGACGAAGGACATTGGGAAAGATTTAAAGGTATCATCATCAATAACAGATGAACA
TCCTTCACCATCAACCGAGACAAAGCATTCGCCTCCAAACGACAAATCAGAAGCACTCGAGAAAGATCCTGTTAGTTCGA
ACAATTGTTCAAGTTCAAGTTCAGGAGGAAAATCACCGGACAAGAAAGTTCGTGAAGATCACGACTCAGATGGTGGAGAC
TCGTGTTGTTCACCTCCGTCAAAACGAGCAAGAACTGAAGAAAGGAGGATGACTACTCCTGATGATGCTGAAACAAAAGT
CAAAGTGAAAGATATTATGAATGCAGCTCAAATACATCTAGTTGTGGCCACACTACTAGTGACGGTCACATTCGCGGCCG
GTTTCACATTACCGGGAGGTTTTGAGAGCGATCACGATAGCCCTAACAAAGGAATGGCGATTCTGACGAAAAAATCAGCA
TTCTGTGCATTCGTTGTTACGGATGCCATTGCCTTTGTATGCTCCGCAGGTGCTGTGTTTAGCTACTTCGTCATGGCTGC
GAATCACCGGCCAAAGACCGCAAAAGAGCTGAGAGTTCTGATGGACATTTATAGAGTTGCAACAATTTTGCAGTTCCTTT
CAATGTTTGCAGTTGTAATTGCATTTATAACTGGGGAATGGGTGAAGAGACATGAAGCTTTTAGGGATCGTTTGGTACGA
GGCATAAGGAATAATAATCTCGGAATGAAATATGAAGATCAGTTTATCCCGCGTATGCTGCTGAAATGGACAGAAGCTGC
AAAATACTTGCGATCTCGTAAAACCTCTGCATATATAACTGTCAACTATTATAGTTACAGTCATGCCGAAGTAGGTTCTT
TAACTACGCCTGCTTCAAGGAAGAACAATCATAACCGAGCAGAATATAACGAAAGAGATGCAGCCAATGATACAGACAGC
AATGGCTGGACCAACTGA

>CcPHR6 |Locus_ID=BC332_26869 |CDS_len=1272 |strand=+
ATGAGTATTCAGAACTATGAGTTACCAAGTGATTACAACTTGGAATTCCCACAGATGGGATTTTGTTTTCAGCCGGAAAA
TGATCACCAACAGCTACAGCAGCAGCAAAATTTGTGGCCTAGTAATGATTCATCATCATCATCAAGAAGTATGATAAGTC
GAATCGGATCGTCACCTTCAGGTTTTTTTGCTACTGAGAGGTACCTTGGATTAACACAATATGAATACCAAGACAACAAT
AACAACAATAGTTGTTCTCAACTGTCAAAGAATCATGATCCTCAAGTAGCGGCGTTTACTCAGCAATGTGGAAATGGATT
CTTGGTCACTTCATCATCAGCACGAGTTGACACCGATATTCCAAAGATTTCAATGCCATCATTCATCAGACCACTTGATT
CTCATGGACTCTATGGAAATCCTTTTAGTAATCTATCAGAAAAAGAGAGGGTTTTGCTTCTTAAGAGAGGTCACGGGTTC
AAGCCTTGGAAACAGCCTCTAGCAGAAATGCAAGGTAAGGCTGCGTACGATACACCCTTGTGGTGGGGCCCTTCCCCGGA
CACCGCGCATAGCGGTAGCGTTAGTGCACCGGGTTTCCCTTTTATGAAAGTCTCAAATAATACATGTGGTTTTAACTTGG
TACATATAAGGCAACAATCTGGAAGTCAATCAGCAAATAGTTATAACAACTCTGGATGTTCAGGAGGATCTTTATCGAGT
AAGACACGAATCAGGTGGACTCAGGATCTCCACGATCGATTTGTTGAGTGTGTAAACCGTCTTGGAGGAGCTGACAAGGC
GACGCCAAAGGCAATACTAAAGCTGATGGATTCAGAAGGCTTAACCATTTTTCATGTAAAAAGTCATCTTCAGAAATATC
GAAATGCAAAGTTCATCCCTGAATCGACAGAAGGAAAAACTGAAAAAATGGACAAACAGAATAATGTGACACAGATCGAC
GGCAAAACTGGAATGCAAATCAAAGAAGCACTGCATATGCAGCTAGAAGTCCAGAGGCGTCTTCACGACCAACTAGAGAT
TCAGCGAAAGTTACAGTTGAGGATCGAAGAACAAGGGGAGCAACTGAAGAAGATATTTGAACAACAACAACAAACAACCA
GGAGCCTCTTGGAGACGCGGAATTCAAGCATTTCATCTCATGCTGATCCGTTCACCCCACACGAAGATGAAGTTTTCGTT
GAAGAAAGCTTCGATAATTCCCATTTCCAATCTAAAATAAGCTACAATGACATGGAAACAACATTTGTTTGA

>CcPHR7 |Locus_ID=BC332_26620 |CDS_len=792 |strand=+
ATGTCATCATATGATCCTCAACAATGTCGAAATGGATTCTTCGAAGACTCAATGGTACAATCTGAGCCTATTTCACTGCC
ATCATTCATAAGTCCTGAGGGACCTTGCAGACAGTACACTTTTTCCGATGCATCGGAAGAAGAGAGGATGTTGCATCTGA
AAAATAAGTTGTTGGGAGAATTCGATGCTTCATATCGGAGGCACCCTGCGTTGCCTTTTGATGGAAATCAAGATTACTGT
CTCTGTCATGATTTGTGTGGCTCTCAATTGACATGTATGCAGCAACAATCCTCGAGTCCTTCACTTACTTGTCATAACTC
TGCATCTTCTGGTGCAACACAGAAGCCTAGTAAGACAAGAATCAGATGGACTGAAGATCTACACGATCGATTTCTTGAGT
GTGCAAATCGCCTTGGAGGCGCTGACAAGGCTACGCCGAAGCAAATACTAAATCTGATGGACTCAGAAAGCTTAACTCTT
GATCAAGTTAAAAGCCATTTGCAGAAATATCGTAATGCAAAACATCCAGAATCTGCAGGGAAATCTGAAAAGAGAAACAG
CCTGGATACTGTGACAGATATCGAGAGCAAAACCGCAACAGAAATCACCGAAGCACTGAAAATGCAACTAGAAGTACAAA
GGTGTCTTCACGAACAACTAGAGAATCAACGAACATTACAAACGAGGATCGAAGAACAAGCAAAAAAGTTGAGGATGATA
CTTGATCAACAGCAAAACACAAACAGGACTGTTTTGGAGACGAGAAATTCAAACGTTTCATCTGATAATTAG

>CcPHR8 |Locus_ID=BC332_16139 |CDS_len=999 |strand=-
ATGTATCAGCCCAAAGGCGTTCCTACTTCAAGCTTAGCCCACAACAATGTTGCTGTCAATAATCAGTCATTAGACTGTGC
CGGCGGCTCAATGGACCCGATCAGTGGAGGCACCAACAATCCTAGTCTTGCCTCAAAGCAACGTCTGCGTTGGACCAATG
AGCTTCATGAACGATTTGTTGATGCTGTAGCACAACTTGGCGGCCCGGATAGAGCTACACCGAAAGGTGTTCTTAGAGTC
ATGGGTGTACAAGGGCTAACCATTTACCATGTAAAAAGCCATTTACAGAAATATCGACTTGCTAAATATCTTCCAGATTC
CTCATCTGATGGGAAAAACTCTGATAAGAAAGAACCAAGAGATATGCTTTCAAGTTTGGATGGTTCATCAGGAGTGGAAA
TCACTACGGCTCTAAAGCTGCAGATGGAGGTGCAAAAGAGACTGCATGAGCAACTGGAGGTCCAAAAACAGCTACAACTT
AGAATAGAAGCCCAAGGTAAGTATTTGAAGAAGATAATCGAAGAACAACAGCGGCTCAGTGGAGTTCTTTCAGAAGTCCC
TGGTTCTGGGGTCACTGCTCCCCCAACTGGTGACAATGGCCCAGAATCTGATAACAGGACTGATCCAGGGACTCCTGCAC
CGTCATCTGAGTCTCCTCATGTTGATAAGCCTGTAAAAGCACATGCCCTAACAAAGAGCCTTTCTATGGATGAATCGTTC
TCCTCGCATCATGAGCCTCTCAGTCCAGATTCTGATTTCCATGAAACTTCGCCTATAGAGAGCCCTAACGGTGAGAGGTC
ATCAAAGAAACAAAGAGTGGGCACAAGTGCAGCATTCACAAAAGACAACATGGCACTTCCACATCAGATATTGGAATCAA

GCTTGAGCTCACCTTACCAGCAACCACATTCAGCTTTTATGACGAGGGACCAATTTAATTTTACATCAGGATTATCTCTT
GGCATCGATGATCAGAAAGTTTCTGGTAGCAACATCTAG

>CcPHR9 |Locus_ID=BC332_23400 |CDS_len=240 |strand=-
ATGGCTTCAAATCGTTCCGATTGTTCATCGAAAGAGAGACTGAAATGGACACAAGAGCTTCATGATCTATTTGAGAAGGC
AGTTAGTCAGCTTGGAGGTCCAGAAAGAGCAACACCAAAAGGAATTTTGAAAGTTATGGGGATTCCTGGTCTGACAATTT
TCCATGTGAAAAGCCACCTACAGTTGGCAAGTCCGAAAGGAAAAGCATATCTGAAATTTTGCCAAACTTCAGCGCTACAG

>CcRAD1 |Locus_ID=BC332_32239 |CDS_len=309 |strand=+
ATGGCCACAAATTCGACGTGGAGTAACCAGCAAAACAAGTTGTTTGAGAATGCATTGGCCATTTACGACACCGACACGCC
GGACCGGTGGCGAAAATTGGCCAAAGCAGTTGGTGGAAAAACAGAAGAAGAGGTGAAAAGTCACTATGAAAAACTTGTTG
AAGATATTAAACATATTGAATCTGGAAATGTTCCTTTGCCTAATTATAATAATGGTGGTGGTGGTGGTGGTGGTGGTCGT
AGCAATAATGGCAACAATATTATTATGGATCATGAACAACAAAGGCTGAAGTATTTGAAGCTCCAATAA

>CcRAD2 |Locus_ID=BC332_22475 |CDS_len=300 |strand=-
ATGGCATCGAGTTCACTTCGATCATCATCATCATCATGGACTCCCCAACAAAACAAGTTGTTTGAAAGGGCTTTAGCTCA
ATTCGATAAGGACACACCTGATCGTTGGCAAAATGTGGCAAGGGCTGTTGGAGGTGGAAAATCAGCTGAAGAAGTTAAGA
GGCACTATGAAATACTTCTTGAGGATCTTAGGCGTATTGAATCTGGCCATGTCCCTTTTCCAACTTATACGAATAATGCC
AACAATGGAGTTGGAGCTAATGAAGAGCATAGGCTTCTGAGGTATATGAACCTGCACTAG

>CcRAD3 |Locus_ID=BC332_31082 |CDS_len=270 |strand=+
ATGGCCACATGGACAACAAAACAAAACAAGAAATTTGAGGATGCATTGGCGTTATATGACAAGGATACTCCTGATAGGTG
GCATAATATTGCTAGGTATGTTGGTGGAAAATCAGCTGAGGAAGTGAAGAGACACTATGAATTGCTTGAAAAAGATGTCA
TGAAGATTGAAAATGATCAAGTTCCTTTGCCCAATTATAGGGCTAATGCTAGCAATGGAAGAAGTAATTATGCTAATGAA
CAAAGGCTTCTGCAGAATCTGAGGTTATAG

>CcRAD4 |Locus_ID=BC332_33762 |CDS_len=279 |strand=-
ATGGCAACAAATTGGACAACAAAACAAAACAAAAAGTTTGAGGAGGCATTGGTTATGTATGACAAGGACAATAATGGAGA
GAAATGGCATAATATAGCTAGACATGTTGGTGGCAAATCAGTTGAGGAAGTTAGAAGGCATTATGATCTTTTGCTAAGGG
ATATTACACAAATTGAAAATGGTCAAGTCCCTTTGCCAAACTATAGGAATGCTTCTGAAAGCAATGCTAGAGGATATGCT
AATGAACAAAGGCTTTTGAAGAATCTAAAGCTACAGTGA

>CcRAD5 |Locus_ID=BC332_32248 |CDS_len=279 |strand=-
ATGGCCTCCTGGACTGCAAGGCAAAACAAGAAGTTCAAAGAGGCTTTGGCTTTATACGACAGGGACACTCCTGATCGCTG
GCATAACATTGCCAGGTGCGTAGGTGGAAAATCAGCAGCGGAAGTGAGGAGGCATTATGAGCTGCTTGTGACGGATATCA
TGCAAATAGAAAATGGCCAAGTACCTTTACCCAATTACAAAGCCGCTGAAAACAACAACAGAGGTTACGCCAATGAACTA
AGGTATCCCAAATCTCTACTATCCATTCAGCCAAACTAA

>CcRAD6 |Locus_ID=BC332_32247 |CDS_len=270 |strand=-
ATGGCCTCCTGGACTGCAAGGCAAAACAAGAAGTTTGAAGAGGCTTTGGCTTTATACGACAGGGACACTCCTGATCGCTG
GCATAACATTGCCAGGTGCGTAGGTGGAAAATCAGCAGCGGAAGTGAGGAGGCATTATGAGCTGCTTGTGACTGATATCA
TGCAAATAGAAAATGGCCAAGTACCTTTACCCAATTACAAAGCCACTGAAAACAACAACAGAGGTTACGCCAATGAACTA
AGGCTTCTGAAGAACATGAAGCTACAGTGA

>CcRL1 |Locus_ID=BC332_22472 |CDS_len=264 |strand=+
ATGTCTTCTTCTCAAGCTTCCCAAGGATCAGCAAGTTCATGGACTGCTAAGCAAAACAAAGCATTTGAGAGGGCATTGGC
AGTGTACGACAAGGACACCCCAGACCGTTGGTCCAACGTTGCTAAGGCTGTTGGAGGGAATAAAACTGCTGAAGATGTCA
AAAGACATTATGAAGTACTTATCCATGATATCATGTTCATTGAGAGTGGTGGTGTGCCCTTCCCAAACTATAAGACCACT
CGTGGACGTACTAATACCAATTAA

>CcRL2 |Locus_ID=BC332_25561 |CDS_len=267 |strand=-
ATGAGCTCAATGTCCTCTCAACATGGGTCTCCAGGATCATGGACAGCAAAACAGAACAAGGCGTTTGAGAAGGCACTGGC
AGTGTACGACAAAGAGACTCGTGATCGTTGGTCCAATGTTTCCAAAGCAGTTGGTGGCAAGACTGCCGAAGAGGTGAAGC
GACACTATGAAATCCTTTTGCGTGATGTTTTCTATGTTGAGACTGGTAGGGTGCCCTTCCCCAACTACAAGACTACTGGA
GGAAGCCATAATTCCACCTCTGACTAA

>CcRL3 |Locus_ID=BC332_05377 |CDS_len=237 |strand=-
ATGGCATCAAATTCACTCAGTGCTTGGACCCCAAGAGAAAACAAGAAATTCGAGCAGGCACTTGCTGTGTATGATAAGGA
TACACCTGATCGCTGGCAAAACATTGCTAGATACGTTGGTGGAAAATCAGTTGAAGAAGTTAAACATCACTATGCTATCC
TTGTGGAGGATCTCAAACACATTGAGTCTGGTGACGTCCCATTCCCAAAATACAAGTCAGATGGAAAGTCTCGTTAA

>CcRVE1 |Locus_ID=BC332_07817 |CDS_len=2682 |strand=+

ATGACAAACGGCAAGAGAGCAGAAAAAAACAAGGATATAATGGAATTGAAGGATGATGTTTTAGTTTGGGAGATCATCTC
TCGCTTGCCCTTGAAGTTGGCAGTTCAATGTAAAGTTCTAAACAAGGATATTAAGGGTCGAATTTCTGATCCTGAATTTT
TACGAACTTGGTTTCGACGCCAGGAAGATCCTTCTACAGAACTCATCTATACATTAAATGGGAGGCACAGATCGCTCCAC
AAAATCACCTTATACCCTATCCCAAACACTCAAATGGAGACGCCTCTCTCCTGTGATATTGAGATTTTGGCCTCATGCAA
TGGTCTCATTCTCATTGACTTTGATCGTGTTACGAAATACTGTGTTTTCAATCCCCTAACTGGAGAGCACCAATTGATAC
CATACCCGCTGATGCCTTCAAATAGGCTTCATAGTATGGGCTTCGCTGTTGATTACCCGGATTCAGATCAATATAAATTG
GTAACCATCAGTGAACGCGTTAAATATTCCCACAGGTTCTACAAATTTAGCCTACTGTCATCGCAGCAACCTGGCTTCTG
GCATGAAATCCAACAGGAAACCAGTTCTTTCAGGCCTTTTCCTGAGGGTAATCCACCTGTTTATTGGTGTGGTTCTCTGT
ATTGGCTCAGATGTGAGGGTAGTGTTGTAGCATTTGATACGAGGAGACAAGCGTCAAGAGTAATTGAAGCTCGGTTTATT
CCTCAATATGATTTCAAGTATGGTAATATTAGCACTGGTAACAATATGAGGCTAGGGACGGCACAAGGTGTACTTACCCT
TGCATGCATTTTCAGAAGGCTCATAGTCCTCGCGACTTATGATAATGCACGTAGTAGTTGGACGGTTACTCACACTGTGG
ACAACTTTATTTCTCGTATGAGCGGCTTTGTTGCTGGCTTTCCAGTCTGGATCGACAGCAAGCAAGTGTCTTTTCTTATG
GAGCGTCCCTGGGTAGTGACACATTATTGGGATCTATATGAGTATGATACTGAGATCAATGGGTACCGAAAAGGTGCAGA
GTGGCACTCAGTTGGTTATCCCATGTATTACTTCCATCCAACGTTAGCTTCTGTCCATCAAGCGCCCTTCAAAAATGTAG
AGGCTGATGATCTGTTGTATATTGCTGCAAAGTTGAATTACATCAGAGGATTTATTATTGAAGGTACCAGTCAGTTTCAA
AATCAAGCTGAGGGGGCTTGGCAAGGTGCCTCATCCGTGAATAGCTCAAAGTTGGAAATTGTGGCTACTCAGGAGAAGGC
ACCGACTTGTCTTGCCAATAAAAATGCTTTAAAGGTTAGGAAGCCTTATACCATTACTAAACAGAGAGAGAAATGGACAG
AAGAAGAACACCAGAGATTTCTTGAAGCCTTGAAGCTGTATGGCCGTGCTTGGCGCCAAATAGAAGAATATGTTGGAAGC
AAAACTGCAATTCAAATTCGTAGTCATGCACAGAAATTTTTTGCAAAGGTTGCTCGAGATTCAGTTAATGATGGTGATGA
ATCCCTAAACGCGATTGACATACCTCCTCCTCGGCCAAAGAAGAAACCTCTGCATCCATACCCTCGCAAGATGGTTGATT
CCCCTGTAGCTAACAAGGCAGTTTCAGGTCAGCCTGAAAGCTCTCCTTCCCCAAATGTATCAGGAAGGGTGAGTCGTTCT
CCAGATTCAGTTTTGTCAGCAATCGGTTCAGGTGTTTCTGAATATCCAGTTGCTGAGCAGCAGAACTCTCGCTTTTCACC
AGCCTCTTGTACTACAGATGTACACACTGCAAATATAATTTCTGCAGAGAATGATGATGAAAGTATGACCTCAAACTCCA
GCACCGTGGAGGAAGTCCATGTTGAATTAAAACCAATATCTGCTAGTACCAGCCCGATTGCCAATTCTGACATGGTAATT
CCTCTTGAAGCCTGTAATCTATCTGCTTTACCATGCTATTTAGGCTGTACACTTACCTCAGGTTTGTATGTTCCTTTGCA
GGAATGTGATATAGTTCATAGGGAGAATTCATGTAATGGTGAAAAGCTAGCTGTTGAAGCACCTTCAGCAAGTATCAAGC
TGTTTGGACAGACGGTTTTCATACCAGATGCCAATAACCTTGCTCTGCGAGCTCCATACAACTGCAACTCATTGCCATCT
AAATCCACAGAAAAGGAAATCAAGATCAGCAGTGAAGATGTACTTCACAGCTTCCAAGCTAACCAAGCAAATTCACAGTT
TATGCTCGCCATGGTTCCAGGCAATATGATTCCACCAGCTTCTTGGTTGTCTCACAACATGCTTGAAAACAATCCTGAGA
TTACTACTGTTTTCCCTACTACAGTTTCTTGGTGGTCCTGGTACCAAGATCTAGTAAATCGATCTATCTTATCGTGTGAC
CAAAAAGCCATGGAGACTGCTGTTCATTGTCAGGGACCGAAAGATGAAGAACCCCAAAGAGAGGGATCATCGACTGGTTC
AAGCATCGGCTCAGCTAGTGAAGTTGACGATGGAAACAGGAGTTCTGAAACAGCTGAGTCCAAATGTACAGCCAAGAGCT
ACAAGTGGAACAACAGTAAGGGATTTGTACCATACAAAAGATGTTTAGCAGAGAGGGACGACAAGTCATCAGGAGCTGTT
TTAGAAGAGAGAGAATCACAACGGGTTCGTGTGTGTTCATAA

>CcRVE2 |Locus_ID=BC332_03406 |CDS_len=1521 |strand=+
ATGCAGCTCATGGAGTGTAGAGTAGGTTTGGATCATGGCTTGACTGAGATTATCGTGAAAACTGACACTGGCTCTAGACA
ACAGCTAATTTATCGGGATCAAAGTGAGTCGGACACTACACCTCCCAGCAGAAGTAGGATCTCACTTGCTGTGGGAGCTC
GCATTCAGCTTGAAGAGCAAGTTACTCCAGCAGAAGAATATGCTCCCAAGATAAGAAAGCCATACACCATTTCAAAGCAA
AGAGAGAGGTGGTCAGAGGAAGAACATAAGAAGTTCCTTGAAGCTTTAAGGCTGCATGGTAGGGCATGGCGACGTATAGA
AGAGCACGTGGGCACCAAAACTGCAGTTCAGATCAGAAGCCATGCTCAGAAGTTTTTCTCAAAGGTTGTTCGTGAATCGA
ATAATGGTGATGCAAGCTCTGTGAAATCCATTGAGATCCCTCCTCCTCGGCCTAAAAGAAAACCAATGCACCCTTATCCA
CGTAAAATGGCAACTCCAGTTAAAAGTGGAACCCTAGCTCCAGGGAAATTGAAGAGCTCTACTTCACCTGATCTCTGTCT
CTCTGAACCAGAGGATCAGTCTCCTACCACTGTGTTGTCTGCCCTAGGTTCTTTTGCATTTGGCACTGTACATTCCAGTA
AGCCAAGTGAGCACTCATCACCTGTTTCATCAGATGTTGCTGAAAATTCTGGTGATGTTGTACTTTCTGAACCATCCGAT
ATCATTCTTGAGGAGAGAAGATCTTCACCCGCCCGAGGTTATGCTAGTTCAAATCAAGACAACCAGTCTTGCATGAAACT
AGATTTGTTTCCGGAAGACAATGATTTTGTGAAAGAAGGCTTAGATGAGGCATCATCCACGCAGTGTTTGAAGTTGTTTG
GTAAAACTGTATTAGTCACTGATTCTTATAAGCCATCTTCAACTTGTGGCGAAATATTGCTGACAGATGAGAATGATGAA
CCAGCGTCACCGACATTGCCTCAAAGCTCCGTCCCTACGAAATATTTGCGCTGGGGTTCAGAATGTGCCCGAAGTACCTC
AACTATTGGACTCCCTGCACAATCATATTACATCCCCGCACCAAATGGAAGTTCAGGACCTAATCAAAGTACCACTTCCC
CTCTTTTGCCGTGGGGCTCATCATATGTATCTGATAAATTTCCTTGTGCTAAGGTGCATAGCCCAATTCCAATCAAGGGA
TGTCCACTCTTTAATGAAACAGGTTTGGATGATAAGGAAACTCAAAAGGAAGGGTCTTCCACCGCCTCCAACGCAGAATC
AGCAGATGTGGAGTCGAGTGGAGACACGAATTTGGAAATTGAAGCTCAAAGTGGTAACGGCAATCTGGTTGTAGTGTCCG
TCAGAGTCAGTGTACCCCCTCTGTTTGAGCGAAGAGCAAATTTCACAAAGCGTGTGGAGGGTTTTGTTCCATATAAGAGA
TGTTTGGCTGAAGGAGGCATCAGTTCCTCAACAATAACCGGAGAAGAAAGAGAGGAGCAACGAACCCGATTATGTTTGTA
G

>CcRVE3 |Locus_ID=BC332_26256 |CDS_len=864 |strand=+
ATGGCCAACAACAGTTCAACTCCCACCGACGCATCCGGTAAGAAGGTTAGGAAACCTTATACAATAACAAAATCAAGAGA
AAGCTGGACTGAAGAAGAACATGACAAGTTCCTTGAAGCTCTTCAACTGTTCGACCGTGATTGGAAGAAAATTGAGGATT
TCGTAGGCTCAAAGACAGTAATTCAGATTCGCAGTCATGCCCAAAAATACTTCTTGAAGGTCCAGAAGAATGGTTCAATA
GCACACGTGCCACCACCTCGGCCCAAGCGTAAAGCAGCTCATCCATATCCACAGAAGGCACCAAAAAATGTATTGGTACC

GTTGCAAGCATCTATGGGTTACCCTTCTTCAATGAATTCCCTTCCACCTGGATATCCATCATGGGATGACACTTCTGTAC
TTATAAACTCTCCAACAGGCGGAGTAATGTCATCACAGGACGAATACCATCTTCAAAGAATTCAGGCTGATATTGGATCA
AAGGGAGCTACATTGATTGGTAACAGCAGCATCAGTGGCATTAGAAGTTCCAACAGAGCGGCACCGAATTCTGAACTACC
AGAGCAGAGCAAACTAGGTTCTGTTCCTCACGGCATACCCGACTTTGCAGAAGTTTATAACTTTATTGGGAGTGTCTTTG
ACCCGGACACTAGAGGTCATGTACAGAAACTGAAGGAGATGGACCCTATTAATTTTGAAACTGTTTTGTTGTTGATGCGA
AACCTTACAATGAACTTGGCTAGTCCTGACTTTGAGCCCATTAAAAATGTATTGTCAACGTATGATCTCAATACAAAAGT
TGTGGGTCTTCCTACAGGTGGTGCTGTCAGTAACCACAATGATCTATCATGTCAGACAATATGA

>CcRVE4 |Locus_ID=BC332_21524 |CDS_len=960 |strand=-
ATGGTATCTGTATATCCAACACTACCGGGTCAAGATTTCCAGTACATGGGTGATCCGATGAAAACGGGTCTAAGGGGTTT
GCATCCGGTACAATCGGGTACGGGTTTGGTGATGCCAGATGATCCGAATAAGAAGACACGGAAGCCATATACGATTACTA
AGTCTAGGGAGAGCTGGACTGAGCAGGAGCATGACAAGTTTCTCGAAGCTCTGCAATTGTTCGACCGTGATTGGAAGAAG
ATCGAAGCATTTGTCGGGTCAAAGACTGTTATCCAGATACGTAGCCATGCACAGAAATACTTTCTGAAGGTTCAGAAGAG
TGGAACAAGTGAACATTTACCTCCCCCACGCCCAAAGAGAAAAGCAGCTCATCCTTATCCACAAAAAGCTCCTAAAAAGG
TTATGTCCCAAGTTGGCAGCATCCAATTTCAACCTCCCGGAGCTTTGCCTGAACCAGGATTTGGTATCGGGCCTGATCCT
TTATCAGTTCCTGGGAATGCAATTAATTTTTCTCCGTGGACTTACGACAATGTGCCTGCTATCAGCACGACACAGATGAG
AAAAGATGATGCACAATTAACCAGTGGAGGAGTCATGCAAAATTGCAGCAGCAGTAGTAATGAAAGCATGCCTAGGAATC
GTAAGACTCTAGAGGCCAATGATCAAAAGGAGAGCCAGAAGCAAATGAAAGTTATGCCAGATTTTGCTGAAGTTTACAGC
TTCATTGGTAGCGTCTTTGATCCAAGTACGAGGGACCACTTAGAGAGACTAAAAAAGATGGACCCCATAGATATTCAGAC
GGCACTGATGTTGATGAAGAATCTGTCCTTCAATTTATCGAGCCCTGAATGTGAGGATCATAGGAGGTTGCTTTCATCAT
ATGGCTTGGGCATTGAAAAAGATAAAATGGACCTTCCTATAAAAACTGATTTTAGAGGTAATGCAATCCAAGCTGTCTAG

>CcRVE5 |Locus_ID=BC332_15390 |CDS_len=906 |strand=+
ATGGTCATGGCCCTTCCTAATCTCGGCTCTTTCTCCAATCCAACTGCCACCGCTTCTCCCGCCGGTGCAACGGCGTCGAC
CTCCAACCCATTGTCTCCTTCCGATGACCCGTCGAAGAAAATTCGAAAACCCTACACCATTACCAAGTCACGAGAAAGCT
GGACTGAACCCGAACACGACAAGTTCCTTGAAGCTCTTCAGCTATTTGACCGTGACTGGAAAAAGATCGAAGCATTTGTT
GGATCAAAAACTGTTATTCAGATACGTAGCCATGCTCAAAAATATTTTCTGAAGGTCCAGAAGAGTGGAACCAATGAACA
TCTTCCTCCTCCTCGGCCAAAAAGAAAAGCTGCTCATCCCTACCCACAGAAAGCCTCAAAAAGTGCTCCAGCTGTCTCAC
CGGTGCCTACATCTTTTCAAGCTTCCCTTCCTTTACCAGAACCTGGATTTGTAAAAAGGCCCGATTCTTCTTTTTTACCT
AGCAATCAAGTTACTGTTGTGCCTGTGCCGTCATGGACTGACAACTCCGTGCCACCAGTTAGTTTGTCGCAAATAAAGAA
AGATAACGTGAGAGAAACAGGTCAGCTGGTCGCTAATAACCGTTGCTGCAGTAGTAATGAAAGCACTCCAAGATCAAAAT
CAACCGTTAAGGTGATCGAGCGGACTCAAGCACCCTCACTGAGAGTTCTGCCTGACTTTGCTCAAGTATACAATTTCATC
GGTAGTGTATTCGACCCTGCGGTTACTGGACACTTGCAGATATTGAAAAAAATGGACCGCATTGATGTCGAGACGGTGTT
ATTGCTGATGAGAAACCTCTCCATCAACCTTACAAGCCCTGATTTCGAGCATGATAGACAGCTGCTTTCATCTTATGATA
TAGACATGGAGAAGAAGTGCAAATAA

>CcSRM1 |Locus_ID=BC332_03442 |CDS_len=1179 |strand=-
ATGACAATAGATGAGTCAAGTAGCTCCATATGGAGTAGAGAGCAAGATAAGGCATTTGAGAATGCCCTGGCAACTTACCC
TGAGGATTCTGCAGATAGGTGGGAGAAAATTGCGACTGATGTTCCAGAAAAAACATTAGAAGAGGTTAAGCATCACTACG
ACCTCTTACTAGAAGATGTTAGCCGGATCGAGTCCGGCAGTGTTCCTTTACCTTGTTATAACTCTTCTTCGGACAGTTCT
CCAAGTCATGGTGGTGATGATGGAACTGGAAAGAAGACTGGGAATTCAGGGAACTTAAATGGTGAATCAAATCATGGAGG
TAAGTCTATGAGGGCAGATCAGGAGCGCCGCAAAGGCATTGCTTGGACAGAGGATGAGCATAGGTCGGGTTCAAGGTTGA
GTTATCTTTGTGAAGCATCTGCTGGCTTATCCATTCTTGCTCCTCCTGATGAACTTGATGACAGCTTTAAGTTGCATTTT
CCAGATATGCTGGAAAGTGGCAAATGTAGTGTACTAGAATGGTACGATGAATTACTAGAATGCCTGGTGGACAGTTACCT
GGTACCGGTACCTATTGCCGGTGGGAGGTATCCCGTGGAGTTAGTCGAGGTGCACGCAAGTCTTTATAGGGAAAGTGACA
ATCTCATAATGCTTTATCTTCGTGGGTTTGAAAACAGATTGTTCCTTCTTGGTTTGGACAAATACGGGAAAGGTGATTGG
CGAAGTATCTCCCGAAATTTTGTTGTGACACGTACTCCGACACAAGTGGCCAGTCATGCTCAAAAATATTTTATACGTTT
AAACTCTATGAATAGGGACAGGAGACGGTCAAGCATACATGACATCACCAGTGTCAACACCGGAGAAGTTTCAGTGCCTC
CAGGTCCAATTACTGGCCAAGCAAATGGTTCTTCTGCAGGAAGTTCTAGCAAACCAAACAAACAACAAACGCCGGTGCGA
GCTACACCCCTTGGTGTTAGCATATATGGAGAAACTAGCATTGGACAACCAATAGGAGGGCCATTTGTTTCCGCAGTTGG
CACACCAGTGAATCTTCCACCACCAACACATATGGAATATGGTGTCCTGCCTCCAATTCCTGGAGCAGTTGTTCCTGGCG
CTCCAAAGAATGTGGGTCCATTGACGTATCCAATGCCACATACGTCAATGCATAGGTAA

>CcSRM2 |Locus_ID=BC332_12967 |CDS_len=951 |strand=-
ATGACACTAGCTAAATCAAGAAGCTCAATATGGAGCAGAGAGCAAGATATAGCATTTGAGAATGCATTGGCAACTTATCC
TGAGGATTCTGCAGATAGGTGGGAGAAAATTGCAGGTGATGTATCAGGGAAAACCTTAGAGGAAGTTAAGAATCACTATG
AAATCTTAGTCGATGATGTGAGCCGAATCGAGTCAGGTTGTGTTCCTTTGCCTTGCTACAACTCTTCTGTTGACGGGTCA
TCGAGCCATGGTGGTGGTGAAAGAACAGGAAAGAAGAGTGGGATTTCAGGGAGGTTAAATGGTGAGACAAATGATGGAGG
TAAGGCTTTAAGGTTGGAGCAGGACCGGCGGAAAGGGGTTGCTTGGACCGAGGATGAACATAGATTGTTCCTTCTTGGTC
TGGAAAAGTATGGAAAAGGTGATTGGCGAAGCATTTCTAGAAACTTTGTTGTGACAAGAACTCCTACGCAAGTAGCCAGT
CATGCCCAAAAGTATTTCATACGTCTGAACTCTATGAACAAAGATAGAAGACGAACAAGTATACATGACATTACTAGTGT
TAATAATGGAGATGTCCCAGTACCTCAAGTTCCAATCACTGGCCAAACAATTGGTGCTGGAGTAGCAGGTTCTTCTGGCA

AATCCAACAAACATTCTCAAACAGCACCAATCGCTCCCGTTGGAGTTGGCATATATGGAACAACCACCATCGGACAACCA
TTCAACACTACATCCGCCTCTGTTGTTGGTACACCGGTGAATCTTGTTCCACCGACAAACAAGTCATGCGGTGTCCAACC
TCCAATACCAGAAGCAGTGGTTTCTGGTGCTTACAATGAAGCACAAATCTACACATTAGTGACATTTTTGAAACATAAGC
GTACAAAGTCAAGTGAAGAGTCTTATGCTTATTCATGTTTAGTGCTATCTTCTTCAGTGGTGCATGAATAA

>CcTRF1 |Locus_ID=BC332_22999 |CDS_len=1548 |strand=+
CAGAATTCAGCATTTCGAAGTGGTCTTGCTTCTCTAGGGGTCTGGTTTGATGAACCAGAAACCCGTGACGATCATGCTTC
TATCTCTGGTGCCTTGTGTTTTGATGAGAAGAGGGTATTAAGGGGCATGGAAAGGAAAGGTGCTTTGTACAATATAGGTT
CCAGTATTGCACCTAAAAGGGAAAATGATATTAACCACGAAGTGCTTGATCTTAAGCAAAGTCAATTTGAAGCTGGAAAA
GTTCAAGCACAAAATGATCTTTCCACTCCATGTGAAGATTATCTTTTAGATATTGAATTTGCTGAAAGCATCACAAATCT
GGATTATGGTTCAAGTAGAGGATTGCTGGACCCTATTTTAGAAAGTCAGATTACATTACCTAGTTGTGATGGAACTGACT
CATGTGGAATGCCATACTCTTCAGGATTAGTTGCGCAAGAAGCTCAAAGTCAGAATGCTATGTTTGGCCTTACCAATTCA
GAGTTGCATGACCTTTCTCACCACAAGTGTGAAAGTCGGATACTAGTTGAGTACAATGCTGTGAAATTCCCAACTGCGTT
CAATCCAGGAGATATCAGCAATGTGGATGATGTGGCAGACTTCTTTGCTGAAGCTGTGTCTTCTGAGAAGATGAATGACA
GCATGATTGACCATTCTGCACTAACATCTAACGGAATTTCTTTGATGTCTAATCCTACGCAAAATGAGGGGCAAGATGTA
AACTGTGAAAGCTTTGATGTGCATGAATCCGCAGTTATAGGCCCTCCAGTCACTTCTCAAGTGATAAACGTTGACACTCC
TGTCACTCAAAAGCGTCTACGCAAGCCTACACGAAGGTATATTGATGAATCCTCAGATCTGAATGCTAGACGTTCTAGGA
AAAAGACTGAGGTTTCTACTAGTGCACCAGAATCAAAGAATAAGTTTCTCAAAGTCAGATGTCAAAAGAAATCTCGTGCA
GAGTCTATGGAAATGGAATTGTTTTCCGAGGAGTCTTCTTATAAAGCTATTCAAGTGCCTTTTGCTTCTCTTGTGAACGA
AGAATGTGATGAGAGGCCTGCATCCAATGCAATACAGGTTTTTAAACCTCACAAGGAGGATCTTCATAAGGAGCCTATGG
TTGTGAATCCTAAAGATGATGCCGTTGCGCCAAAGAAGTTGGATCAAGATGGTGCCCGGAGGAAACATCACCGGCTCTGG
ACTGTTTCAGAAGTCAGAAAGTTGATTGATGGAGTTGCCCAATATGGAGTTGGAAGATGGAGTCATATAAAGAAGCTTTA
TTTCTCATCATCTGGTCATAGAACACCTGTGGATCTCAAGGATAAATGGAGAAATCTTCTTAAAGCATGCTATCTCCAGA
AACAAAGCAAAATAACGGAAAAGGGTAAACATAATCTGTCTTGGCGACCTTTGCCCAAGTCTGTCCTTCATCGAGTCGGT
GAACTGGCCAACATGCATCCGTATCCAAGAAACCGGAGATGCGAGAGTTCCCGTTCTCCATGTGCCTCACCTTCTCCCGA
GCATTTTAACAATACTAACATCCAGTGA

>CcTRF2 |Locus_ID=BC332_09582 |CDS_len=1821 |strand=+
ATGGATGCAATTGAAGCCAAAACTGAGGTTAGTGTTGTTCCGGTAACGAACACCTTTGGGGCTTTGGAGAAGGAGATTAC
TGATCCTATTGTTTACCAGCTTGTCCGGGTTGATGGCGATGGGAGATTAGTCCCGGCAACTCAGGATGAAATTATGGTGG
TTGAGGACTTGCTTGAAGATGATAAGTGTGAACCCAAGCTTGTTCCAGATACCAGACAGATTTCGGAATCCTGCATAACT
GAGGGATACCTTATGGCGAGAAATTTATTTCAAGTTCCAGAAGAAAAGTCAAATGTGTTAGTTGATGCAGTGCCTGACTT
GGGAAACATAGACGCTCCTGAAAAGGTGGCTGTTCATGGATTGAATCCTTTCTCAGAACTCAGCGATTTTGACCAATCTG
TGAGGACTGAAACATGCTTCCTTTCCCAGGATGCATCAAATGAGGATATGCCATCAACCTCTGCTGCTGGTTCTAGTTGG
AAGCCTGACTTTTCAAAGTTGGAGGGAAAGATATGTTTAGACAGCCTAACAGTTAAAGAACTTCAGGAAACTTTTAAAGC
AACTTTTGGAAGGGAAACTTCTGTCAAGGACAAACAGTGGCTTAAGAGGAGGATTACCATGGGGCTGACTAATTCGTGTG
ATTTTTCATGCACTACTTTCATAATAAGAGATAATGTAGTGGTGAAGAACGGTGAAGAACAAATATATCATCGTGTAAAG
AGTAGAATTTCTGCAGATTCTGAAGATGGAGTGGCAAATTCAGACTCTATAGGGTCATTAGGTGATCGTGATAACAGAAT
AAATGATGATGCTGATCTTTGTGGCGAAGATGTTTCTACTTCAGCCTTTGAAAGTTGTAATGTTACTAAAGATCTGGACA
CAGAGCAGAGAACAGCCAAACGAGCTCGCAAGCCAACAAAAAGATATATTGAAGAACTTTCTGAATTAGAATCTAGGGAA
TCCAGTGAAAAGTTAGCGTCACCAGATAAAATCTCCAGATACCGATTTGCATGTCCACAAACTCATATGAGGCCCACCAA
AAATGTGAGGTCAAATGCGAGACCTCTGGTCACCAGGGAAGATTCTTTAGGAGGTTCTGGGATTCAAATTCCGTTTGTTT
CTCGGATTCGAAGGAGCCGTCCGCGTGAGAACTTCATGCCTCTTTTGAAACTTCAGCCCTGTGGTATGGACATGGTGACC
AGAAAAGTAACGAGTGCTTTTGATGTATCTGGGCCACAAGAAGATGATAAGAGAAACAATGACCTGATCAAGTCCAGCTC
ATCTCCTGGGTGGACTCAACAGCCTCTTATTGCTGCTTGTGAAAAAGACGATCACTACAGTGGGATGAAAATTGTTGAGC
TGGAGAATGACGTAGAGCCGAATGACTGCTCTGAGTACAATTCGGATGATAATGTGGTGACTGTACCCACCCAAAAAGGA
GGAATGAGGAGAAAACATCATCGACCTTGGACTATCGATGAGGTTGTTAAGCTTGTGGAAGGTGTAGCCAGGTATGGTGC
AGGTAGATGGTCTGAGATAAAACGGCTTGCTTTTGCGTCTTGCTCTTATCGAACTTCGGTGGACCTGAAGGACAAGTGGA
GAAATCTGCTGAAAGCTAGTTTTGTGCAGTTGCCTGCTGAAAAAGGGATTCTGAATTCCCGGAAACAGGCTTCAGTTCCG
ATCCCTGCTGCTATTCTCCGGCGTGTGAGAGAGCTTGCTGATATGCAAGGTCAGTTCCCACCGGGTCTCAGTACAGGGAA
GTCAAGTGGACATAGTAGTAGCGATAGAAGTGTACATGATGCTAGGTCTGGATTCCTGTAA

>CcTRF3 |Locus_ID=BC332_29142 |CDS_len=1689 |strand=-
ATGCAAAATGCTATTGATCCGTTCGAGTACAACTTGAACACATCGAATGAGAGTGCAATGAGTTTTCAACAGGAGCCTGA
ACAGGTAGTAGGCGCTGCATCCGTTTATTTTGCATTGGAGGAGGAAGCAATAGGAGTGGAACAACTATTAGCAGAACCTG
AGTATGATGATATACTTAATACGCTTCTAGATTTTAACACATGTGCATTCAGTGTTCCCGAGAGCTACTTTAAGGAGTTT
TCAGCTTCGGATACTGTCTTAACAAAAAGTGACTGTGGTGCACTTCAAAGAAGTCATAATGAAGGGGATAAAACAAAGGA
GAAGTTTCTTGATGGATTGTCAAACGGAGATGATGAAAATCATTGCTTCATTTCATGTGAAGATTATCTTTTGGATATTG
AACTTGAGGAAGACACTCCCACCCTCCATGATGTCACAAGAGATATATCCTGTATAGAGAGTGTGAATTTGGAGAACCAG
CTAGCTGATTCAGATGGAAGAGACTGTGGCGTTCATGTGTTAACGTTATCTGGTGCAAGTACCTCATCTGATCACGATGC
TACGATATTAGACAATTTTGATTGCATGTCAACTGATAAACTACTCAAGGTGTTCAGGGAGATGTTGGGACATCAAACTC
CAGTTTCGGATAACCAATGGCTGAAACCCCACATGATAGCTGGCTTGGAAAACCAGGAGATATCAGACAAGAATTTCAGT

TTTCCAAAACGCTCTCTTGATTCGAGTGAAAACCAAGGGGTAAAAGTCCCGCCAGCAAGTTGCCAAAATCTCCTTACAAT
ATCTACTGCCTTTGCTAGTATATTTACTTTCAGAAGAAAGCCAAGAGTTCAACAAGTGAAAAGGAGAGGGCGTATCCAAT
GGAATTCCTTCAAATCCTTATCTTCTGTAGCAGGTGAAATACATCTCGATTGCCCGGATAAGTGGGCTAGCAAGGAGTCG
GCTGAAGAAAATGTAAAATGTGATGGAATGAAATCAGGAATATCTGAACAGTATTTGAAATGTAAACCTTCGCGAAGAGG
ATTCGGTCGGCGATATTACCGCAGAGGAGCCACAGTTACATCACAAGGTCTTGGTAAAAGAAATATTCTGCCCACTGAGG
ATCAGCTTATCAGGAGGGAGGGACATTTATCCAAAGATTGCAAAGCGAACAGAGCCCATGACGACAACATCTATTCAGCT
GAGGCACAAGATGATCCTTCTGGCACTCTTTCAGACCAGTCTAGTGATGATACATCTGAAGATGATTGGACTATAGGTTG
TGAGACCCGAGGCACTAAACAGGACCGAAAACGCAACAATTATTGGTCAACACGTGAGGTTCTAAAGTTGGTGGAGGGTG
TTTCCGAGTATGGCGTTGGCAGATGGAGTGACATAAAGAAAATGTTTTTCCCATCCTCAATGCGTCGTAGTCCAGCTGAT
CTGAAGGACAAATGGCGGAATCTTTTGAGGGCTAGCTGTCGAAGATTGCAGAGCCGGAGAGGGGTTGATGCAAAGAAGAA
GCACCGCATGCGCTCGATTCCTCAGAGTGTGTTGAACCGCGTTCGAGAGCTTGCTGTCATCTATCCACATTCGAAGCAAC
GTAGATCAAGAATCTCATCAACTGCATCCCGCTCCTTTTCTTCGAACGTGAAGTTCGATAACCATTTACCAGTGCTTGCC
TATGAATGA

>CcTRF4 |Locus_ID=BC332_26430 |CDS_len=1671 |strand=-
ATGCAAACTGCTATGGATCCGTTCGAGTACAACTGGAGCACATGGAATGAGAGTGCAACGAGTTTTCAACAGGAGCCTGA
ACAGGTTGTAGCTGCTGCATCCGTTTATTTTGCATCAGAGCAGGAAGCAATAGGAGTGGAACAATTATTAGCAGAACCTG
AGTATGATGATATAGTTAATACTCTTCTAGATTTTAACACATGTGCATTCAGTGGTGTTCCCGAGAGCTACTTTAAGGAG
TTTTCAGCTTCGGATACCATCTTAACAAAAACTGATTGTGGTGCACTTCAAAGAAGTCATAATGAAGGGGATAAAACAAA
GGAGAAGTTTCTTGATGGATTGTCAAACGGAGATGATGAAAATCATTGCTTCATTTCATGTGAAGATTATCTTTTGGATA
TTGAACTTGAGGAAGACACTCCCACCCTCCATGATGTCACGAGAGATATATCCTGTGTAGAGAGTGTGATTTTGGAGAAC
CAGCTTGTTGATTCAGACGGAAGAAACTGTGGCATTCATGTGTTAACGTTATCTGGTGCAAGTTCCTCATCTGATCACGA
TGCGACGATAATAGACAATTTTGATTGCATGTCAACTGATAAACTACTCATGGTGTCCAGGGAGATGTTGGGGCATGAAA
CTCCAGTTGCGGATAACCAATGGCTGAAACCCCACATGATAGTTGGCTTGGAAAACCAGGAGATATCAGAAAAGAATTTC
AGTTTTCCAAAATGCTCTCTTGATTCGAGTGAAAACCAAGGGGCAAATGTCCCGCCAGCAAGTTGCCGAAATCTCCTTAC
AGTATCTACTGCCTTTGCTAGTATATTTACTTTCAGAAGAAAGCCAAGAGTTCAACACGTGAAAAGGAGAGAACATATCC
AATGGAATTCCTTCAAATGCTTATCTTCTGTAGCAGGTGAAATACAGCTCGATTGCCCGGATAAGTGGGATAGCAAGGAG
TCGGCTGAAGAAAATGTAAAATGTTGTGGAATGAAACCTGGAATATCTGAACAGTGTCTAAAATGTAAACCTTCGCGAAG
AGGATTTGGTCGGCGATATTACCGCAGAGGAGCCACAGTAACATCACAAGGTCTTGGTAAAAGAAATTTTCAAGTTGGAT
TTGATCAGCTTATCAGGAGGGAGAGACAATTATCCAAAGATTGCAAGGCGAACCGAGCCCATGACGACAACATCCATTCT
GCTGAGGCAAAAAATGATCCTTCTGGCACTCTTTCTGACCAGTCTAGTGATGATACATCTGAAGATGATTGGACTATAGG
TTGTGAGCCCCGAGGCACTAACAAGGACAGAAAACACAGGAAGTATTGGTCAACACGTGAGGTCCTAAAGTTGGTTGAGG
GTGTTTCCGAGTATGGTGTTGGCAGATGGAATGACATAAAGAAAATGTTTTTTCAATCATCAATGCGTCGTAGTCCAGTT
GATCTGAAGGACGAATGGCAGAATCTTCTGAGGGCTAGCTGTCGAAGATTGCAGAGCCGGAGAGGGGTTGATGCAAAGAA
GAAGCGCCGCATGCGCTCGATTCCTCGGACAGTGTTAAACCATGTTCGAAATCTTGCTGTCATCTATCCATATTCGATGC
AACGTAGATCAAGAATCTCATCAGCTGAATCCCTCGCTTCTTCTTCGAACGTGAAGTCTGATAACCATTAA

>CcMYBR14 |Locus_ID=BC332_32768 |CDS_len=900 |strand=-
ATGGGGAGGTATCCATGTTGCAATTATGATTATAGTGATTTAAAGAAAGGGCCATGGACAAAAGATGAAGATGAGAAATT
AATTGAATATACATACAAAAATGGCCATAATAATTGGAGATTAGTTACAAAAAATGCAGGATTAAAAAGATGTGGAAAGA
GTTGTAGATTAAGGTGTAATAATTATCTTAAGCCTGGTATTAAGAGAGGTGGATTTACAAAAGAAGAAGAACAAATCATC
ATTAATCTCCATTCTTCTTTTGGAAATAATGCAAATCAAGATGAAGGACTTATTGCATGGTCTTCTAGAATTGATGGTAT
TGAATGCTCACTTCCCAATGGTTATCCTTCTATTTCAAGTTCCTTGAAAGTCCATTCAGAGACCTTCAATAAAATTGAAA
CGATACAGAGTAGATCGGCATGTCTCATGTGCAACGATGAGAAGCACAAATCCAAAAATGGTCCAAATTCTTTAGAATTT
TGTCAAGCTAATGAATCGAATTCAAGTGCAAATCAAGTTGAAGGACTTAATATTGCATGGTCTTCTAGAATTGACAGTAT
TGAATGCTCGCTTCCCAATGATTATCCTTCTATTTCAAATTCCTCGAAAGTCCATCCAGAGACATTTGATAAAATTGAAA
CGATACAGAGTAGATCAGTATGGCTCCTATGCAATGCTGACAAGCACAAATCGAGAAATGATCCAAATTCGTCAAAAGGA
ATTCAAGCTAATGAGTTGAATTCAAATCATGTTGAAGGACTTGTTCCCTCTACTTCAAAAAGTTCCAATTTAAACAAAAA
TTGCATTTCCAAATTCTCAGAATTAGATGATATTGATGCATGGGAAATACCTCAAGATGATGAAGCCATAAGTTTCTTTT
GGAATACTATTTTTCAATGA

>CcMYBR15 |Locus_ID=BC332_30589 |CDS_len=729 |strand=+
ATGGGAAGGTCTCCTTCTTGCGATGAAACAGGCCTTAAGAAAGGTCCCTGGACTCCTGAAGAAGACCAAAAACTCATCAA
CCATATCAAGAAACATGGCCATGGGAGCTGGAGAGCTCTCCCTAATAAACTTGCAGGTCTTAATAGATGTGGAAAGAGCT
GCAGATTGAGGTGGACTAATTATCTTAGGCCAGATATCAAGAGGGGAAAGTTCACTCAAGATGAACAGAAAACAATTCTC
AGTCTCCATGCTATTCTTGGCAACAACTTGCCTCATCTTATAGCTCTTGCAAATTTGAAGGAATTAGTCGAACATCACCA
CTCTTCGTGGGAAGGACAACAAGCTGCTATTGCTATAAGATCACTACAAACTGAAGTTGCTACACTTCAATATTTGCATT
TACTTCTTCAAACTCCAAATATGCCTGCAAACAACACAACTATTAGTGCCAATATCCCAGAAATTGAAGCTTATAATAAT
CTTTTAAACTCAATGAAAGCTGACCAAATTATTATTGGCAATAATTCCTTGAACAATATTATTCCACCACCTAATTCAAC
TAGTCTTCAAGATTCAATCCCTTTCTCCCATTTGCCTGAATTACAAGCTAGTAGTAGCTTCCACTTATCAACTTCTCTTC
ATAAAAAAGAAATCATAAAAACACCCACTACTATATACTACTTCACCATGGCAGCTTCCTTCTTCTCTGGGAGTTGTTGC
AACAAATGA

>CcMYBR16 |Locus_ID=BC332_24180 |CDS_len=885 |strand=-
ATGAATGTTGATGATATTGATAATAATAATATTGTTGGAGATGAAGAACAAGATGGAGAGAAGAATAATGACAAAAATGA
TGTTGTATCAAAAGGAAAATACAATTTATGGAAGAAGAGAGGCAGAAAAAGCATGAAAGATATTAATGAAGGAAGAAGTC
AAAGCAGCGCTATTAATAACACCGTCAAGAGAAAGGACTGCATAGAATGGACTACAGATCTCCATTTCATATTCACAAAA
GCTGTGAAACAACTTGGTGAAGGAAGATGTTATCCAGCAGAAATTCTTGAAGTGATGAATGTACCTAGTCTTACAAAGAA
ACAAGTTGTCAGTCATCTTCAGGTTCAGCCTGGCTTTTACTATGGGGATCATCAGGATTATGGCTTGAATCGACATACTC
AAAATGGTTACAACTTAGATATCAATGCGGCCCATGTACCAACATATTCAGTGGTTGTTATCAGTGTGAACCCCGCATGT
CGAACTAGAGGCATTTGTAAAACAATTATACCATTATTCGGGGACTTGCCTTCAGATGGTTTTGTATCTTTGTATCTTGG
CCACATCCATATGGTGGAATATACATTTTCTGTAACTTTCAATCTTGCAAACAGGACATTCTTGCCTTTGGTTTTTGGTA
CTTTCGGAAGAAATTCTCTGAAATTATATCCATCCGTAGATGCAAGGCCAATAGAGTGGCCTTCCGCGATATTGGCGCAT
CGCGGAAAGGGCCAAATCCCTAATTGTCTAATTCCAGTGAGGGTCCGTGATATTGGTGCATCGCGCCAAACTTCCAGGTC
CCATCCAAGGTGGAAACGTGATACGACCACATCGCACCATCGTGTTTATTCACAGTTGGCAATTTCCAGTGGCTTGGCGC
GATAG

>CcMYBR17 |Locus_ID=BC332_15032 |CDS_len=813 |strand=+
ATGGAAGCAGTGACGGCAATACTTGGTGGTGGGCTGCGGATTGGTGTTGTTTTTCAGGGTAAGAAGGTTAGAGATGACAA
CAAGACCTTATTTCAGGCTGGAATTTCACATGAGGACAAACTTGATGCTCTTGGTTTTACCCTGGAACCTAGCCCCTCTC
GAGCTTCCCCACCTCTAGCTCCAGAAGGTCGTTCTCGTGTACTTCCTTGTGAGACAGCTCAACCACTAACAAGGTGCTCA
CCTGCTCCTACTGTTATTCGTAATGGCATACAACAGGGGACATATAATGGCTTCACGGATCATCCAAGAACAAATTTGAG
TAACTTCATTGATAGCGATCACGATTCAGCTCCGTCCCCACCTGATGCCTTTCTGGAGAAAAATGCTGCAGATTCAAGAG
CATTGGTTCCAGTTGCTGCAGTGGATGCAGAAGCCTTGGCTATGGTTCCAATGCGAAAGCCCAAGCGTTCTGAAACCGCG
CAGCGTCGAATCCGCAGACCTTTTTCTGTTTCTGAAGTGGAAGCACTTGTTCAAGCTGTTGAGAAGCTTGGAACTGGAAG
GTGGCGTGATGTCAAAATGCGAGCTTTTGATAACGCCAAACACAGAACTTATGTCGATTTAAAGGACAAATGGAAAACAC
TGGTGCACACAGCGCGAATATCCCCTCAGCAAAGGAGAGGTGAGCCTGTGCCTCAGGAACTCCTTGACAGGGTCCTCATT
GTGCATGCTTACTGGTCCCAACAACAAGCTAGGCAACAGATGAAACATCATTCCGAGACGCGTCTTTTGCTTTCAGATGG
TTGTAAAGGATAA

>CcPHR10 |Locus_ID=BC332_14338 |CDS_len=315 |strand=-
ATGGGTGTTTCTGGAGCTACGTCGACATCTTCAAACCTCATTCCCTCACAGGCAATCCCGTCAGCATCTAAGAGTCGAAC
AATCCAAAGAAAGTTGCAGTTGCGGATTGAAGAACAAGGTAAATACCTCGAAATGATGTTTGAGAGGACGAAGGACATTG
GGAAAGATTTCAAGGTATCATCATCAATAACAGATGAACATCCTTCACCATCAACCGAGCCAAAGCATTCGCCTCCCAAC
GACAAATCAGAAGCACTCGAGAAAGATCCTGTCAGTTTGAACGATCGTTCAATCACCGGACAAGAAAGCTCGTGA

>CcRVE6 |Locus_ID=BC332_05079 |CDS_len=375 |strand=+
ATGTTTAATTCAGAGATGTCTCTGATGGTTAATAACGCTATTGCTCCCCGCGATCAAAGTGGAGAAAGTGGGGTGGACTC
GATCCATTCCAGCAGGAATCAAATTTCGTCAGATGTGGGAGCATCATCAACAAGGAGCATCAAGCTGAAAGAGCAAGTTG
CTTCTGCAGATGAATATGCTCCCAAGGTCAGGAAGCCATACATCATTACAAAGCAAAGGGAGCGCTGGACAGAAGAAGAG
CATAAAAAGTTCCTTGAGGCTTTGAAACTGTACGGTAGGGCATGGAGGCGTATAGAAGGTAGAAATGAAAAACGATTAAT
GTTTATTAGAGACATCTCTTGGTTATCATTGCATATTAACGGAGATCTTGATTAA

>CcMYBR18 |Locus_ID=BC332_00996 |CDS_len=363 |strand=-
ATGCCCCAACAATGGACAATAATCGCTCCTATTCTTGGTCGTACCTCATCTCAGTGCATTGAACGTTATGATAAGATTCT
CGATGGTGAGGGTTGGGAACGAGAAGATGATGAGAAACTTCTTCACCTTGCAAAAATCATGCCCCAACAATGGACAATAA
TTGCTTCTATTCTTGGTCGTACATCATCTCAGTGCATTGAATGTTATGATAAGCTTCTCGATGGTGAGAATTATGACAAA
TTGCTTCCATGGGAGATTGAGTCACATCCAGATCAATCAAAGCCTGCACCTCCTGATCCTATCAATATGGACGATGATGA
AAAAAAAAATGCTTTCTCATGCACCGCAGATTCCCGACTATGA

>CcA |Locus_ID=BC332_25948 |CDS_len=1134 |strand=-
ATGGTTAAAAGTGTAATGGAGAGCGGTGAATTAAGTTGTGGCCAAAGCGATGTGGTAATTAGTCACTTGATGAACGGGAG
AGGAAAAATCTATCCAACGTTCATAAAGAATGATAGACACGTGGAATTACATATGCTTTGCGTTGATTCTAATAACTCCA
GACCTATATTGCGGGTTAAAGTTGTTAGAAGGTCCCTGAAAAAAGCTTCCACATCAACCCTACCCCCACCCCCACCCCCG
GTAGTGGATGATACGTCAATGGAATACGATTGCATGAGTGGTGATGAATGGAATAATAGTGAAGATTATGCAGAAAAGGA
GTGTGGAGGAGCTGAGGACAGGCAGTTCGAACCACAAGGAAGCCACTCTTTCTCGGATGGGACCAATCTTTTTGTAGGAC
AAATATTCAAGGATAAGAATGTTTATGTAATTTTAAATATCATGAATACTGCTATTATTGCCAAGTCCTCTGGAGTCAGG
AAAGGTGCATGGACTGAGGAAGAAGATTTTCTATTGAGAAAATGTATTCAGAATTATGGAGAAGGAAAGTGGCATCTTGT
TCCCATTAGAGCTGGTCTGAATAGATGCAGAAAGAGCTGTAGATTGAGATGGTCACTTATTGCTGGGAGATTGCCGGGAA
GAACAGCAAACGATGTGAAAAACTACTGGAACTCACACCTACAAAAGAAGCTAATAACTGCTCCTCATCGACAAGAGAGA
AAGTACAGTACTGCCCTCAAGATCACCAAAAAAAACGTACTAAGACCTCGCCCTCGGACCTTCTCATCAAGTGCAAAGAA
TAATATTTCTTGGTGCACCAACAAAAGTACTGTTATCACAAACACACTAGACAAAGACGAACGCGACAAAGAAATAGGAC
TAAATATTTGTCAGAAGCTCACAAGTGAAACGTCGTCGACTATAGATGATGGAGTTCATTGGTGGACAAGTTTACTGGAA
AATTGCAAGGAAATTGAGGAAGATGTGGCTGCAGTTGGGATCTTTGAGGAAAAAAATAAGTTGGTACCAAGTTTGTTGCA

TGATGAGATTAATTCACTAACCATGCAACAAGGACAAAGTGATGGTTGGGATGACTTTTCTGCTGATATTGACCTATGGA
ATCTACTTAATTAG

>CcBLIND |Locus_ID=BC332_29076 |CDS_len=948 |strand=+
ATGGGAAGAGCACCATGTTGTGATAAAGCAAATGTAAAGAGAGGGCCATGGTCACCTGATGAAGATGCTAAACTCAAAGA
TTTTATTCACAAATTTGGCACTGCTGGAAATTGGATTGCTCTTCCACAAAAAGCTGGACTAAGGAGATGTGGGAAGAGTT
GTAGATTAAGATGGCTAAATTATTTAAGACCAAATATAAAGCATGGTGATTTTTCCGATGAAGAAGATAGAATTATTTGC
ACCTTGTATGCCAACATTGGAAGCAGGTGGTCGATTATAGCAGCTCAGTTACCGGGAAGAACCGACAATGATATCAAGAA
TTATTGGAACACGAAGCTCAAGAAAAAGCTCATGGGATTAATAATACCTAACAATAATAATAATAATAATTTTATTAATA
TCCAAAAAAAATCACCCCATTTTCTATCTACTACTACTACTACTTCTTTTCATCAAGCCCAACAAAATCTTGGGCCTTTA
ATTACAGGCCTAGAACAGCCCATTATTTCTTCAGCCCAACAAAATATTTTGCCTAATATGATGATTATGACAAATAATTA
TAACTTTCCTAATTTTGGTGCAACAAATTATAATTTGCAAAATTACCCAAAATTTGAAGCAAATTGTTTGCAAAATTATT
CACAATTTGGAGAAGTTGCAAGTTGTTCTTCATCAGATGGAAGTCATTGTAGCCAAATGAGTTTTGGTAATAAAGAAATT
AATAATAATATTAAAAGAGAAGAAATTATGAGATTTGGTCAAACTATTTTTGAAGGAATTAATAATCAACAATTTAATCT
TGATTATTATGGTACTATGGAGCAACTTGCTACAAATTGTTGTACTAATGGCAATAATGGTAGTACTAGTATTAGTAGCA
ACAATTTGTTGTTGTACAATGATGAGAATTGTAACAAGTCAAATGAAATAGGGATGTTTTATTATTGA

>CcMYB1 |Locus_ID=BC332_04984 |CDS_len=1023 |strand=-
ATGGGCCATCACTGCTGCAGCAAACAGAAAGTTAAGAGAGGCCTTTGGTCTCCAGAAGAAGATGAAAAACTCATTAAGCA
CATCACCACCCATGGCCATTCCTGCTGGAGTTCTGTCCCTAAACTAGCTGGATTACAGAGGTGTGGAAAAAGTTGCAGGC
TGAGGTGGATAAATTACTTAAGGCCAGATCTGAAAAGGGGTTGTTTTAGTGAACAGGAAGAAAGGATCATAATTGATGTA
CACAGAATCTTGGGAAACAAATGGGCACAAATAGCCAAACATTTACCTGGCAGGACTGATAATGAAGTGAAGAATTTCTG
GAATTCTTGCATAAAAAAGAAGCTTATTTCTCAAGGTTTTGATCCAAACACCCACAATCTCCTCTCTCGCGCTGCTGCTG
CTGCTACTCCTCCTCATCGAAACAACAAGAAAAAAACCAACAATAATCATACCACATCATCAGTTTTCACTATTGAAACG
AGTTTATCTCCATCATCAACCAAAGAATTAGTTTCAATGGACATGATCAAAGCAAGTCTTGCAGCATTGTCACCTTTTCC
TCACACTAATAGTAGTACTACAGTATTAACTATGCCTCAGTTCCAAACAAGTTGGAGTCACTTATATGAATCTGCTTGCA
ATTATTATAATCATGTTCCCATCAAGACGAATATTGTTTACCAAAACCACAGTACGTCAACAGGGACGTTTGGAATTATT
AATGAGAATTGTATGTGGGGTGTTACTGGATTCGAACCACATCATGAATTTGGTCCCTCCAACAATATTGGACATGAAGA
AATGCAACAAGGAGCACAAGTTCAGCTTCAGGAAGAACATTGTCTGGAGGAAGTGTACAAGGTTAATGATCTTCATGAGT
TCAACAAGCATGGACGAATCGCGGAGAATGTAACATTCGATGACAGCTCTGACTTTGATTTTGAGTTTGTGGATTCTGAA
TTGATACCTTGTGGAATTTACACTAACGTAAATTCCATAGATCAACTTGCATGGGATTGTTAA

>CcMYB100 |Locus_ID=BC332_00785 |CDS_len=723 |strand=-
TTAAAATGCTTATTTTGCAGGTGGTCTCTAATATCAGAACATTTACCAGGTAGAACAGACAATGAGATAAAAAATTATTG
GAATTCTCATTTGAGCAGGAAAGTAGAAAGCTTAAGGATTCCAAGTGATGAAAAGCTGCCACAAGCTGTAGTAGAACTAG
CCAAAAAAGGAAAACAAAAACAATTTATCAAACAAAGATGCAAAAGAAAAACCAATGCTAGCGTTTTGAATTCCACAAGT
GAAACTGCAGTTCCTATTACGCCAAGGCTAAATATTGAGAAAGAAACCCCAAACACTATAGGAAACGGCGATAATAATGC
CATGGAATTAGATCGTAACGATCATCAGATGTTGTGGCATGACGATATTGTCCTAATGGACGATAAGGATGCAGAGATAG
ACGAAGATTTCATTTTCAATTGTTTATGGAACGACGAAGGAGAAAATCTTGAATTAGTCGAGAAAAACAACAACAATAAT
AATGAAATATTTGGTGAGGACAGCTATGGTACTCTTAATTGTTGGGATTGGGAATATTTAGGTGAAATATTGAATAATGA
AACATTGGCAAGTACACAAGAAGCAGGGCAAGAGAATAACGGTATGTCAAGTGTTTCAATATTTGACATGAATGACAATA
TGCCAAACTGCATGAATGAAGAAGCAACGGTTGAAAATATTGATCTCATGCAAAGTGATTTGGTAAATTGGCTTTTGTCA
TAA

>CcMYB101 |Locus_ID=BC332_16135 |CDS_len=846 |strand=-
ATGGGACGTTCACCTTGTTGTGAAAAAGCACATACAAATAAAGGAGCATGGACTAAAGAAGAAGACCAACGCCTCATCAG
TTATATTCGTGCTCATGGTGAAGGTTGCTGGCGTTCTCTCCCTAAAGCTGCAGGATTGTCAAGATGTGGAAAGAGCTGCA
GATTGAGATGGATAAATTATCTAAGGCCTGATCTCAAAAGAGGGAATTTTACTGAAGAAGAAGATGAATTGATCATCAAA
CTCCATAGTTTGCTTGGAAACAAATGGTCAGTTATAGCCGGAAGACTGCCGGGAAGAACTGATAATGAAATCAAGAACTA
CTGGAATACACACATTAAGCGAAAACTCATCAGCCGCGGCATTGATCCTCAAACTCACCGGTCACTCAACACTACCGCGA
CTATCTCCACCACCACTGCCATTCCCACCACCACAACAACCACCAAAAACATCAACATGGATTTCGAAAACGTTGATCAA
AAACCTAATATTATTATTGCCACGTCATCATCATACGATGAAACAAAATGCAGTAGTGGTACTACTGAGGAAACAAAGCC
ACTAGACATTATACCAAAAATACCCTCATCACAAGTTATGATAAATCTTGAACTATCAATAGGGTTACCATTGCATACTG
ATCATATTTCCTCAGCTGAATCAACTGCCTCATACAACTTCTTAGCCACCGTAGCACCGCCACCAACAGTGGCAGTGCCG
GTAGTAGCCACCGCGTCGGAGACGGTGGCAAAAACAGTTTGTTTATGTTGGCAAATTGGATATCAAGGGGGTACTCAGTA
TTGTGGTAAATGCAAAAACACCACTGGATTTTACAGATATTACTGA

>CcMYB102 |Locus_ID=BC332_33436 |CDS_len=810 |strand=-
ATGGGAAGATCACCATGTTGTGAGAAAGCACACACAAATAAAGGAGCATGGACTAAAGAAGAAGATGAAAGGCTTATTGC
TTATATTCGAACTCACGGTGAAGGTTGCTGGAGATCACTTCCTAAAGCTGCTGGACTTCTTCGTTGCGGTAAAAGTTGTC
GTCTCAGGTGGATTAATTACCTCAGACCTGACCTTAAACGTGGTAACTTTACTGAAGAAGAAGATGAACTCATTATCAAA
CTCCATAGCCTTCTTGGTAACAAGTGGTCACTTATAGCCGGAAGGTTACCGGGAAGAACAGATAACGAGATAAAAAACTA

CTGGAATACGCATATAAGAAGGAAGCTTTTGAGTCGGGGCATTGATCCAACAACACACAGGCCCATTAACGAGCCCAGCA
CGACACAAAAGGTGACAACAATTTCATTTGCAGGTGGAGATCATAAAACAAAAGATATTGAAGATCAAGATTATAAGATG
ATTAATGTCAAAGCTGAATCTGGCTTAAGTCAAGTAGAAGATGAAATTAATAGCAGCCCATTTCGAGAACAATTTCCAGA
TTTAAATCTTGAGCTCAGAATTAGCCCTCCTTCTCGACAAAATTACCAACAGAGCCATCATGTTCAAGATTTGAAACAAA
GTCCCACAAGGTGTTTTGCATGCAGTTTGGGTATACAAAATAGTAAAGATTGCAATTGTAGTAAAAATAATAATAATAAT
AATAATAATATGGGTAATATTGCAAGTTATAATTTTTTAGGATTAAGGGCTAATGGCGTTTTGGACTACAGAACTTTGGA
GACTAAGTAA

>CcMYB103 |Locus_ID=BC332_02487 |CDS_len=819 |strand=+
ATGGGAAGAAAACCTTGTTGTAGTAAAGAAGGTTTGAAGAAAGGTGCATGGACTGCAAAAGAAGATATGCTTCTTACTGA
ATACATTAAGGAACATGGTGAAGGTACAGGATGGAGAGCTCTTCCTAAGAAAGCTGGACTACTTAGATGTGGCAAGAGTT
GTAGATTAAGATGGGTGAATTATCTTAAGCCGGGGCTCAAGAGAGGGACCTTTACACCTGAAGAAGACGATCTTATCGTC
CGACTTTATACTCTTTTGGGTAGTCGATGGTCCCTCATTGCCGGAAGAATACCCGGTCGAACAGACAATGACATCAAAAA
TTATTGGAACACACATCTTTTGAAGAAACTCAAAGCTCAAGGAAATGAGCCAAGAAGACACAAATCTATTAGAAAATCAA
AGGAGAAAGAAAAAGCTTCTTCCCAAAAAAAACAAACAAGGAATCAGAAAACAAAGGAGAGGAAAAAGAAGAGAAAGATA
GCTGAAAATGATAGCAATATTGAAGAGAGAGAACAGGTAGCTAAGAAGACAGAAGAGCAGCTACAGATGCAGGATTTTTC
GCAAGCGGTGTCAGAAAATAATTACAATTTTACTGGTTCCTCCTGCAGTCAAGATGGCACAAGTAGTGGTGCCTCTACTA
GTGAAATAAATCTTGAAGAAAATAATTACAACAGTGGAATTTATGAGAAATTTCAAGTGATAGATGAATTACTCCTCAAT
GATCAATGGACTGAGTACAATAATGAAAGCCACATGATGTTGAAAGAGATTTATGAAGAGTATTTTCAACTTGTTTCAAA
AAATTCATTGTCGTTTTAA

>CcMYB104 |Locus_ID=BC332_00057 |CDS_len=783 |strand=-
ATGGGAAGAGCCCCATGTTGTGCAAAAGAAGGATTACGTAAAGGGCCATGGTCTTCTCAAGAAGATTCATTACTCACTAA
TTATATTAATCAACATGGTGAAGGCCATTGGAGATCTTTGCCTTTAAATGCTGGGTTGCTTAGGTGTGGGAAAAGTTGCA
GGCTAAGATGGGTGAATTATTTAAGACCGGGGATTAAAAGAGGAAATTTTTGTGAAGACGAAGATGATCTTATTGTGAGA
TTACATTCACTTTTGGGTAATCGATGGTCACTAATAGCGGGGCGATTACCTGGTCGAACAGATAATGAAATCAAGAATTA
TTGGAACACGCATTTAGTCAAGAAGCTTAAAAATACCGGAATTGACCCAAAACCCCTCAAGAATTTCCCTAGAAGGAAAA
AAACTCAAAAGGAGGGGAAGAAATGCAAGAGTCAGAATTCTAAGGGTCAAAGTGTACAAGTCGAGAAGATTAAAGTATTT
GCACCAAAGCCCATAAGGATTTCTTGTGTGAATTCAAGGGACAATAGTTTTGAAAATGATACGTTGAGTACAAGTACTTG
TTCCTCAAATAGTAATTTTGAAGAAGTTGATGTTAGAAAAGAAAAGGAAAATGAGGTGAATTTTCTTGCAAGGGACTTAG
ATTTTGGTGAATTACTTAAAGGGGATGAATTTTGTGATGAATTTCTAATGGGAGAAAGTTGCAATTTTTCAAAATGCCCC
TTTTCATTGGATGATAATATGGTGGAGAAAGTGTATCAGGAATATCTTCTACTTCTTAGTTAG

>CcMYB105 |Locus_ID=BC332_20335 |CDS_len=834 |strand=+
ATGGGGAGAGCACCATGTTGTGCTAAAGAGGGACTTCGTAAAGGTCCATGGTCTACTAAAGAAGACTTATTGCTTACTAA
TTATATTAAGGAAAATGGTGAAGGCCAATGGAGAAACTTGCCTAACAAAGCTGGGTTACTTAGATGTGGAAAGAGTTGTA
GACTAAGATGGATGAATTATTTAAGGCCAGGAATCAAGAGAGGAAATTTTAGCCAAGACGAAGATGATCTTATAATAAGG
CTCCATTCGCTTTTGGGCAATCGTTGGTCACTCATATCTGGTCGATTACCAGGTCGAACTGACAATGAAATCAAGAATTA
CTGGAACACACATCTCATCAAAAAGCTCAAAATTGCTGGAATTCACCCCAAAGTCCACAAGATTTTCAACAAAAAAGAGC
CCAAGAAAAAGCCCAAAAGTGATAAACCAAGAAAGAAACAAGACAAAAAAAAGAAAAATAACAAGAAAAAAGACCAATCT
TTAGTAAATAATAATAATAATAATGACACACCTCAAGTCGTATTTGTCCCAAAACCAATTAGACTTATGAGGAATTACAG
TGTTGAAATGTTGTCGCGCCCTTGTCAGATCTTCAAATTCAACATGCATGCTTCGGCATTATTGAAGAGTCAGAATAACG
CAGCTTCATCATCAGATAATTCTGAAGAATTAGCTGAAAATAACAAGAAGAATATTAATATTATTACTAAAGATGATGAG
AATTTTTTATTTGATGAATTTTGTGATTTTTCAAATGAAAGCATGGTGGACAAGGTATATGAAGAGTATCTTCAACTTAT
TTCTGAGAAATGTTATGATCCAATGTTAATGTAA

>CcMYB106 |Locus_ID=BC332_27082 |CDS_len=522 |strand=+
ATGAGAAAACCTGGTTGTGATTACAAAGAGGAAATGCATAAAGGATCTTGGTCTAAACAAGAAGATCAAAAACTCATTGA
TTATATCACTAAACATGGTGAAGGTTGTTGGAGAGATTTACCTAAAGCTGCTGGTCTGCTTCGTTGCGGAAAAAGTTGTA
GGCTAAGATGGATGAATTATCTTAACCCAAATTTAAAAAGAGGAAATTTTTCTGAGGATGAAGATGATCTCATCATCAAG
CTTCATGCTCTTCTTGGCAATAGGTGGTCCTTAATAGCTGGAAGATTGCCAGGAAGAACTGATAATGAAGTGAAGAATTA
TTGGAATTCCCATTTGAGAAGAAAACTTATAAAAATGGGAATCGATCCAAAAAATCATAATATTTCTCATTATCTTCGTA
TAAAAAGACTTGAATATTTCCAAGAAAATAGCATAAAATCAGAAAATAATGCAGTAATATCTGATGCTACAAGTTCTAAT
TGTGTTACAAGTTCATTGCCTGATCTTAATTCACTTCCATAG

>CcMYB108 |Locus_ID=BC332_14002 |CDS_len=954 |strand=+
ATGGATCATCATCAGAAAATTGAAGTTGATTCAGGACGAGGAGGAGGACGAGGAGGAGATTATACGAAAAATAATCAAGA
AATTAGGGAAGAAGATATTGTTATGGACCTTAGAAGGGGTCCATGGACTGTTGAAGAAGACTTTACACTTATCAATTTTA
TTGCTCATCATGGTGAAGGTCGTTGGAATTCCCTTGCACGTTGTGCTGGTTTGAAGAGAACAGGAAAAAGCTGCAGATTA
AGATGGCTTAATTATCTTCGACCAGATGTTCGACGTGGGAATATTACTCTTGAAGAACAACTCTTGATTCTTGAATTGCA
TTCACGTTGGGGCAATCGTTGGTCTAAGATTGCTCAACATCTTCCTGGAAGAACAGACAACGAGATAAAAAATTACTGGC
GAACTCGAGTGCAAAAGCATGCAAAACAGCTCAAATGTGATGTGAACAGCAAGCAATTCAAAGATACCATGAAGTATCTT

TGGATGCCAAGGTTAGCCGAAAGAATTCAAGCCGCCGCCGCTTCTAACCCGGCGACAGCTTCTTCCTCTGGCCCCCCCAC
CACCACTTACATCCAAAACCAAGAAATTCAACATTCACTACCAAACATGAATCATCATATGTCAGACTATACACCTATCC
CATTTATCGAGAAAACAAACAGTCTGAATTATTCAGCTACTTCAGCTTCGTCAGACAATTACTCGTCCGACCTCACCGAT
GGTTGCTACAATTTCCCAATTAACCAAAGCAATAATCAGGATTATTCTCAAGTTAATCAAAGTACTAATCAAATGTGCTA
TGGAGAATCCACAATTAACCCAACAAGTTACGATTTTCACCATGGATTTCAAGGATTCCAAGAAGTTGATCAACAAAACA
ATACTCAATGGATGGACAATCCATGGAATATTGAAGATGTGTGTTTCTTACAACAATTGAACAATGACATGTAA

>CcMYB11 |Locus_ID=BC332_27759 |CDS_len=1260 |strand=+
ATGGGTGGTGCTTCTACTTCAATGGGGATGTTTTTTGCTGATATGAATTCACTTTCCATAAATACTCCCAAAAGTGTTGA
CGAGGGTAAAGGGAGGAAGGGTTCTTGGGATTTTCAATTCTTGAATAAAGGTGGGGCAGTTTTTTCTTGTTGTCAGAATC
TTGATGAAGTCCAAAATAGTGAAGAAAATAATGAGAGTATTGATCTTAATGCTTCTTGCTACAATGAAGAGAAACAAATT
ACACTGCATAGTACTTTTAATAGTAATAATAGCAGTAGTGGTGGTGGTAATAATGGTAAGGAGACAGAGAGTGGTCAATC
AAAGCTTTGTGCTAGAGGCCATTGGAGACCAGCTGAAGATGCACAACTTAAAGAACTTGTTGCTATTTATGGCCCTCAGA
ATTGGAATCTTATAGCAGAAAAGTTAGAAGGAAGATCAGGAAAAAGTTGTAGATTGAGGTGGTTTAACCAGTTGGATCCA
AGAATTAACAGGAGGGCATTTAGTGAAGAGGAAGAGGAAAGATTAATGGCAGCTCATAGATTGTATGGTAACAAATGGGC
AATGATAGCAAGGCTATTTCCAGGGAGAACAGATAATGCAGTGAAGAATCATTGGCATGTAATAATGGCTAGAAAATATA
GAGAACAATCAAGTGCTTATAGGAGGAGGAAAATGGGACAATTTGTTTATAGAAGATCAACACTAGTAGTTGAAGAAGCA
GATAGTGGCAAAGCACAAGCTCCAATTATCAGTTGCCAAAATTCTGTTAACCAATTTGGTGCCACTGCTAATACTAGTGT
GAAAATAAATAATGATGGTCCAAATGAAGCAGCAGCAGCAACTCCTAGCAGCACCAATAATGTCACTCTCCTTTCTCCTT
ATTCACCATTTTGTGCTCCCCCTTTTGACTCATTACCTGCAGGTCATTGTGGGAATGAGATGATGAATATTCTAAACCAG
CAAGGGAACAAATCTTGGGATAGGAAAATGGAAATTACTCATCACCAACACCACCATCCTACTCCACCATCTAATTCATT
AATGATGATGAACAGTATGCAACAACAGTCACAATACTTACAGTTTCCCTATGTCACAACGTCAAGAACACAAATCTCAT
CATCACCCTCAGCAGCACAAGCAGAAGTACTAGTAAAAGAGGAGGACAGAAGAAGCAGCAGCAGCAACAACAACAACAAC
AGTGATAATAGCACAATTGCACCACCATTTATAGACTTTCTTGGAGTAGGTGCCACTTGA

>CcMYB110 |Locus_ID=BC332_18300 |CDS_len=645 |strand=+
ATGAAGGATAAAGAAGTTAAAACAAGAATGAAGAGAGGATTTTGGAAACCTGAGGAGGACTTGATATTGAAGAATTGTGT
GGAGACTCATGGAGAGGGAAACTGGGCTACCATTTCTGAGAAGTCGGGATTATTGAGGAGTGGTAAGAGCTGCAGACTAA
GGTGGAAAAATTACCTGAGGCCAAACATCAAGCGAGGAATGATGTCAGAAGATGAAAAAGACCTCATCATAAGACTCCAT
AAGCTTCTTGGCAACCGATGGTCGCTAATTGCTGGTAGGCTACCTGGAAGAACAGACAATGAAGTTAAGAACTTCTGGAA
CACACATTTGAACAACAAGAGATCCTGTAAAGGCAAAAAGAAGCATGTGAAGTCCAAGGAGGCAAATACTCACAGCCCAC
AAGGCAAAACGCAACAGTGCCCTGCTGAGACAGTGAGAAACCAAGAAGTGGCCACCAAAACAGTTTTAGATTCATGGATA
GAAGAAATGCAGGACTTTAACTGCAGCTTACTATCACCTGTGATGAATAACATGCCATTTCTCGAAGACGAACCTTTTAT
TCCCATATTGGACGACATAGTCTTGCTTGAAGCATTCACAAGCACTGGTAAAGAAGCATGGAGTGAGATTCAGACATTCC
TTTAG

>CcMYB111 |Locus_ID=BC332_18314 |CDS_len=834 |strand=-
ATGAAGGTGACAGAGATGAGAATGTTGGGATGGATATGTGGACTTACTAAGAAAGATAGCGTTATAAATGAGACTATTCG
AGACAAGGTGGGAGTAACCTCGGTGAAAGAAAAGATGCAGGAATTGAGATTAAGACGATTTGAACATGTGATGAAAAGGT
TAACAGCAGAGAGGAAGAGTAAAAGTGCAATGAAGGATAAAGAAGTTAAAACAAGAATGAAGAGAGGATTTTGGAAACCT
GAGGAGGACTTGATATTGAAGAATTGTGTGGAGACTCATGGAGAGGGAAACTGGGCTACCATTTCTGAGAAGTCAGGATT
ATTGAGGAGTGGTAAGAGCTGCAGACTAAGGTGGAAAAATTACCTGAGGCCAAACATCAAGCGAGGAATGATGTCAGAAG
ATGAAAAAGACCTCATCATCAGACTTCATAAGCTTCTTGGCAACCGATGGTCGCTAATTGCTGGTAGGCTACCTGGAAGA
ACAGACAATGAAGTTAAGAACTTCTGGAACACACATTTGTACAACAAGAGATCCTGTAAAGGCAAAAAGAAGCATGTGAA
GTCCAAGGAGGCAAATACTCACAGCCCACAAGGCAAAACACAAGAGTGCCCTGCTGATACAGTAAGCAACCAAGAAGTGG
CCACCAAAACAGTTTTAGATTCATGGATAGAAGAAATGCAGGACTTTAACTGCAGCTTACTATCACCTGTGATGAATAGC
ATGCCATTTCTCGAAGACGAGCCGTTTATTCCCATATTGGACGACATAGTCTTGCTTGAAGCATTCACAAGCTCTGGCAA
AGAAGCATGGAGTGAGATTCAACCATTTCTTTAG

>CcMYB113 |Locus_ID=BC332_30875 |CDS_len=1059 |strand=+
ATGGGTAGAAAGCCTTGTTGTTCTAAAGAGGGATTAAACAAAGGTGCGTGGACTCCGATCGAGGATAAAATTCTAATAGA
TTACATCAAAGTTCATGGTGAAGGGAAATGGAGAAATCTTCCCAATAGAGCTGGTCTTAAAAGATGTGGAAAGAGTTGTA
GACTAAGGTGGTTGAATTATCTAAGGCCAGATATCAAGAGGGGAAATATAAGTCCAGATGAAGAAGATCTCATAATCAGA
CTTCACAAGCTTCTTGGAAACAGATGGTCTCTTATTGCCGGAAGGCTACCAGGACGAACAGACAATGAAATCAAGAACTA
CTGGAACACCAACATTGGAAGAAAACTTCAAGAAGCAAAAGGTTCTGGTCAGCCAAATCGCGTAACGTCTTCATATCGTC
AGCGCAATCGCTCTAGTCATGCCAAATCAGCCAACCCAAGCCCAATTACCCAACTACAGCAAAATAATCAAGAACAAACA
GTTCAGCAAGATTCAAATTGTCTACTACAAACAGACGTTGGACGTGAAGGATCATCGTCCTCCTCCTCCACCTGTTTGGT
CGTCTGCAATAAGGCAATTTGGTGCACTAAGATTTTTATCAGTCCACAAAATACTGATCAGTCAGTCAATGAGATTACTC
AAATTGATAACAATGACAAGGCAATGACAGAGGAAAACGCGACAACTAATATTGTAACATCCTCATCATCGTCATTGTCC
GAGCAACAACAACAACCGGTATCAGAATCATATTCGCCAACAAATTTGTCCGTAGAAATGGAGAATTATAACTTCAATTT
CATGTTTGGTCTCGATGTTGATGATCCTTTTCTTTCTGAACTTCTTAGTGTACCTGATTTACGTGACCAAATTTTGGAAA
ATAGTACTATAAGTAGTACTGTTGATGCTGGTGATGAAGTTGGAGATTGTAATATCTCCGTCAAAAAGGAAAGGCAAAGG

AGCTATTTTCCTCCAAGTTCTAGTCAAATTTCAATTTTCTCAGAAGGGACGCAACACAACGATTTGGAACTTTGGATCGA
TGGGTTCTCTTCTTGTTAA

>CcMYB115 |Locus_ID=BC332_34193 |CDS_len=777 |strand=+
ATGAATACTCCAATAATCTGTACAACATCGTTGCAAGTAAGGAAAGGTGCATGGAATGAAGAAGAAGATTTTCTTTTGAG
AAAATGCATTGAAAAATATGGTGAAGGAAAGTGGCACCTTGTTCCTGCTAGAGCTGGTCTAAATAGATGTCGGAAGAGCT
GCAGACTTCGGTGGTTGAATTATCTGAGGCCACATATCAAGAGAGGTGACTTCGATCCAGATGAAGTGGATCTCATTTTG
AGGCTTCATAAGCTCTTAGGAAACAGGCAATTTTATGTTTTAGATTCACCTAAATTTGCGGGATCATCTCATTTGAAAGT
TAATGATATTAGAGATCTAGAATGGTCACTTATTGCTGGTAGACTTCCGGGAAGGACAGCGAACGTTGTGAAGAATTTCT
GGAATACTCGCCTTCTGAGGAAGGTAAATATTGCTCCGATTAACAATAAGATCGGAGACAATATTAATACTAAGAATGAG
ATAATAAGACCTCAACCTCGGAACTTCTCAAGTACCATGAAGAATGTTTCTTGGTGCAACAACAAAAGTATCATAAATGA
AGCAAATATACTGGAAAATTGCTATGAAATTGAAGAAGCAATAGCAACTGGAACTAGAACACCTTTATGCAAGAATATCA
GCTCTGAGAAAAATTGCAATGAAATTGATAAAACACCATGTTTTTAAATGGTGGAGGCAACGCCATGCAACAAGGACAA
AGTGATGGTGGTTGGGATGAATTTTCTATGGATGACATATGGAATCTACTTAATTAG

>CcMYB12 |Locus_ID=BC332_23833 |CDS_len=1071 |strand=+
ATGGAAAACGATACAGCGGAGGTTCAAGAAGTCACCGTCGCCGTTAACGGTTTTTACGGCGGCGGCGATGATGAGGAAGT
GTTGATTACAGAAGACGGAAGTGGAAAGAAAAACGCGGACCGCGTAAAAGGGCCATGGTCACCTGAGGAAGATGCGATAC
TTAGTCGTTTGGTGAGTAATTTTGGGGCGAGGAATTGGAGTTTGATCGCACGAGGAATACCAGGTAGATCGGGGAAATCG
TGTCGGTTAAGGTGGTGTAATCAGCTTGATCCTGCTGTTAAACGCAAACCCTTTACCGATGATGAAGATCGGATTATCCT
TCAGGCCCATGCTATTCATGGGAACAAATGGGCATCAATTGCAAGGCTTTTGCCAGGTAGAACAGATAATGCCATTAAAA
ATCATTGGAATTCAACCTTGAGGCGCCGTTGTGCCGAACTTGGTAGGCTAAGAAGTGACTCCAGCAACGTCATGGAGGAT
GTTAGTGTTGAAAAATCAAAAGCATCATCCGAGGAAACCCAATCATGTGGAGATGTGAATTCTTTGAAAGCTATTGAGGG
AAAAGATGTTAGCTCTGAAGAAAACCAAGAAGATAATCTCTATGAGGATAAGAGTCAAGGAGATGTTCAACAGAGTGATG
CAGCAAATGATCCTCATACGCTCTTCCGTCCTGTGGCTCGTGTAAGTGCTTTTAGCGCATATGGTTCTTTGAATGTCCCA
GAATCCCAACTGCAAACTCCAAGGCTAATTCCTGTCCAAGCGCCTGATCTTGGGATAAGCAAATTGCTTGAAGGAGCTTT
CACTGACAGATTAGTGCCTCACCAATGTGGACATGGCTGTTGTGGCAATGGCAATCAAGCAATTAACGGAAGTTCACTGT
TGGGGCCTGAGTTTGTTGACTATGATGAGCCTCCTTCCTTCTCAAGCCATGAGTTGGCCACATTAGCCACAGAGATAAGT
AATGTTGCTTGGTGTAAAAGTGGGTTGAAAAACAGCAGCATTGAGGTTATCTTCAATCCAACAACCAGGGCAGTGTGTGG
TGCCTCTCACTTGCCAAGCAGACCGTTCTAA

>CcMYB13 |Locus_ID=BC332_13912 |CDS_len=1197 |strand=-
ATGAACGAAAACGCTGAAAAGAGCAATTTCTCCGCTGTCGCTGTCACCGCCGCCGTCGAAAATGTCTCATTCGAAGGCGG
CGATGCGGTGTTACACGGCGGCGGAAGCGAAGGCAACGGTGGAAATGCCTCAGTCGACGGCAACGGAGGAGATGATTTCG
TTGACGACGGCGGTGATGTTGAGATGTCGGAGAGCGGAAGAGGTAACAGAGTGAGTAGTGGAAGAGTGAAGGAGCCGTGG
TCAAGGGAAGAAGACTTAGTATTGATGGAACTCATATATAAGTTTGGTGCAAGGAATTGGAGTATGATAGCACAAGGAAT
TCCAGGTCGTTCTGGGAAATCGTGCCGACTTCGTTGGTGTAATCAGCTTGATCCTCTGCTTAAACGCAAGCCTTTTACTG
ATGAAGAAGACCGCATTATAATTAATGCGCATGCAGTCCATGGAAATAAATGGGCATCAATTGCAAAATTGCTCCCTGGT
AGAACAGACAATGCAATTAAGAACCACTGGAACTCTACACTAAGGCGCCGGTTTGCAGAGGTATGGAGGGTTAAACCATT
GTGCAGCGTGATGTTAGATGATAGCATTGATAACGCCAAGGTATTATTTGAAGAAATTAAGTCACATGGAGATTTTAACC
AAATTAAGCCCCTGGAAATGCTGGAAGGTAGTCGCGCAGAATGCATGTCCAGCCAGTCTGAAGACAAAACTCAAATATAT
GAAATATATGATATTCCTGAGAAAAGTCCAAACTTCGTCTCTGAAAGAAGTTGTTCTACGTCTGTGGAAAACAGTGATCC
ATCTGCTTCTCGTCCAACTGAGAAAATTGGCGGATTTTATGTTTGTAACCCTTCTGCAACTTCAAGAATAGTGCCTATGC
AGGGACCCCTGATCCACACATTCTCACCAGACTTTGATATATGCAAACTTCTTGAAGGTTGTGTATCTGCTGGTGAAACC
ACAGTTCCTCAGGGATGCGGTCATGGTTGCTGTGCAGCTTCCACCAATAGCTCCTTCAAAAGTTCATTGTTGGGTCCCGA
GTTTGTTGAATACGAGGAACATCCTCCCCACCTTGGCCATGAGTTAGCTTCTATAGCCACAGATTTGAACAACATTGCTT
GGATCAGAAGTGGACTAGAAAAAGCTGGTGAATTAAGAAATTCTCGACATAGTTCTACTCAAGTGGGTTTTGGATAA

>CcMYB14 |Locus_ID=BC332_31012 |CDS_len=729 |strand=+
ATGAAAACCGTGAAGGAATTGATTCGGGTCAAGGGTCCATGGAAAAAAGAAGAGGACGAGCTATTGCAGAAGCTAGTTGA
AGAACACGGTGCAAAGAACTGGTCGATTATTGGTAAATCGATTCCAGGACGATCAGGAAAATCGTGTAGGCTCCGTTGGT
GTAATCAACTTTCTCCTCAGGTGGAACATCGACCTTTTACACGTGATGAAGACGACAAAATTATCAAAGCCCGTGCAGAA
TTCGGTAACAAATGGGCTACTATTGCCCGTTTGTTAACAGGTCGAACTGATAACTCCATAAAAAATCACTGGAATTCTAC
CCTTAAAAGAAAAGGTCTTGAACTTTCGAAAGATTTGGCGTTTGAAAATCCTCAACCGCCATTGAAAAGATCGTTGAGTA
CCGGGTCTGGTACAAATTCGGGTACTCCATCTGGATCTGATTTGAGTTCGGGTTTTGCCCGACCCGATATTTCAACGTCT
CTGAGTCTCTCTTTACCTGGGTTCGGGTCCAGCGAGGATTCAAATACTCCAATAATTCAGAATTGCAACTTTGGTCCTTC
TAGTATTGAGAAACAGTTATTTAGTCCTGAATTTTTGAGAGTTGTGCAAGATATGATACGAAAAGAAGTTGAAAGTTATA
TGTCACAGTATGAGGAGGAAAGGGTTGCAATGTACACTGAAGCAGTAAAAAATAATGCTATTAACAACATTGAGATTTGC
AAAGATTGA

>CcMYB16 |Locus_ID=BC332_11900 |CDS_len=981 |strand=-
ATGGCGGCGAGGAAAGATTCGGATCGGATCAAAGGTCCATGGAGCCCCGAAGAAGATGAGCTGTTACAGTCGTTAGTGGA

AAAACACGGGCCGAGGAATTGGACTTTGATAAGCAAATCGGTTCCGGGTCGATCCGGAAAATCGTGCAGGCTCCGATGGT
GTAACCAGCTTTCACCACAAGTGGAGCACCGTGCGTTCACACCTGAAGAAGATGAAACGATTATTCGGGCTCATGCGAAG
TTTGGTAACAAATGGGCGACGATAGCCCGTTTGTTATCGGGTCGGACTGATAACGCGATTAAGAACCATTGGAATTCTAC
TCTTAAACGTAAGTGTCCTTCTATGTCTGAAGATTTAAGCTTTGATAATCCTCAACCACCTTTGAAAAGATCGGCTAGTG
TTGGCCCGAGTATGATGAATCCGGGTAGTCCTTCTGGATCCGACTTGAGTGATTCGGGTCTTTCCGGTTCGGGTTCGGGT
CTGGATCTCCCTCTACCTCTTGTTTATCGTCCTGTTCCCCGTACCGGCTGTATTTTCCCTCTCCCTCCTCCTCCTGCTCC
ATCTGTACCCGACCCGCCCACTTCTCTATCCCTGTCTCTACCCGGCTCAGTATCCGTACCCGGATCCGGATCCTTTGAAA
AGCCAAGACAGTCCCCTCAAACACCACCACTACCACCACCGCAGCCTCCTCCTTTTGCGGCGGTTGAAAAGCCAATTCCA
CCACCTGCACCGGTACTTTCTTCTACCCCGCCTTTCATGGCCCAGGTCCCTCAAACCCAGCAACAAAGTTACGAATTTTG
TGCACCAAAATCAGGAGAAAAGCAGTTTTTTAGCCCAGAGTTTTTGTCAGTTCTGCAAGCTATGATAAGAAAGGAAGTGA
AGAGTTACATGTCTGGGTTTGAAAAAAATGGTTTCTGCCTGCAAACTGATGCAATAAGGAATGCAGTAATTAAGCGTATT
GGAATCAGCAAAATTGAGTGA

>CcMYB17 |Locus_ID=BC332_01622 |CDS_len=912 |strand=-
ATGAGTGAGATAAAGAAAGGGCCATGGAAAGCAGAGGAAGATGAAGTATTGATTAAGCATGTGAAGAAGTATGGGCCAAG
AGACTGGAGCTCCATTCGATCCAAAGGCCTTTTACAACGTACTGGCAAGTCTTGTCGTCTTCGTTGGGTCAATAAACTCC
GTCCCAACTTGAAAAATGGAGTGAAGTTTTCAGCAGAGGAGGAAAGGACTGTGATTGAACTTCAGGCACAATTTGGGAAC
AAATGGGCTAGAATTGCTACATATATGCCTGGAAGAACTGACAATGATGTCAAGAATTTTTGGAGTAGCCGGCAGAAGAG
ATTGGCTAAGATTCTGCGGAACTCACCGCCACAGCCTAATAAACCACAAAAGTGTATCCACAGGGAAGCCCCTCCTGCAA
CTCAAAAAGTTCCCTCAGTAGAGGCACCAAAATTGAACTCATCAGCAGAGGAAAGGCCTTTGTCCATGTCCCAGGATTGC
TCATCATCCTATATGAATAACTTCGAGACAACCAGCATGGTCCCATTGCGTGAGTTAGTGAATTCAACTTCACTTCCTTT
CGAGCAAGAACTGCTTCCACTTGAGTTTACTCCCAATGAGAAGAGAACCAGCATCTGGCCACAGTTTCCGCTGTCTTTCC
CTCAGATTCCGCTCCAAACTACCTTTGATCATCCACTAGGACATCAAGAATTACCCATGAAGCTCGAGGATACCAACTTC
CTAGATTATTTTGACATGGGAAATGTGCAAGTCCCTCTCGCACCATCGTGTTCAGGACAAGAGAGAACCTCTGAGATTGC
TATGAAGAGAGAAATAATGGACTACCCCCTTACCCCAGATAGCTTCATCCATGACTTCCCCATGGACATGTTCGATTATA
TTGATCCACTGCCAAGCCCATCCGAATGGTGA

>CcMYB19 |Locus_ID=BC332_15639 |CDS_len=1518 |strand=+
ATGAGTATGACAAGTGAAAGCGATGACAGGATGGCATCACAAGATGGGGTGGATTCGCCATCTGTCGAAGAAGCTTGTGG
TGGAGGAATTACCGGAGGAGGTCTACCACTTAAAAAGGGCCCATGGACTTCTGCAGAAGATGCAATTTTAATGGATTACG
TCACGAAACACGGTGAGGGGAACTGGAATGCTGTCCAAAAGCACTCAGGACTTGCTCGGTGTGGTAAAAGTTGCCGTTTG
CGGTGGGCCAATCACCTGAGACCAGATCTAAAGAAAGGTGCATTCACTCCCGAGGAAGAGCGGCGCATAATTGAACTGCA
TGCTAAGATGGGAAACAAATGGGCACGAATGGCTGCTGAGTTGCCTGGGCGCACAGATAATGAGATAAAGAACTACTGGA
ATACCAGGATAAAGAGACGTCAGCGTGCAGGCTTGCCAATTTACCCTCCAGATATTTCTTTTCAGGCGATCAGCGAAAAC
AAACAAAATGAGGAGTTGGGTGCGTTCTCCTCTACTGATTCACAGTATCCTGATTTCTTGCCAATGAACAATTTTGAGAT
TCCAGCTGTGGAATTCAAAAGGTTGGAATTCAATAAGCAGTTGTGTCCACCAGCACTTCTTGATATTCCCAATGGTGGCA
TACTTGATATTCCTGGAAGAAGCCTGTTGGCACAGGGTCTTAATTCTGCCTATTACAGTCGGTCATTCCTCTCAACAATG
CCTCCAGCCAAGCGTATACGAGGTGCAGAATCTCTGTTCTCTGGTTTAAATGGTGATTGCTCTCCATCAAAAAATGAAGG
TTCTTTTCCAACCTGCCATCAATATCAGGAAGATGGTTCCTTGCTTGCTCAGTCGATGGGATTTTCATCTTCATTTAATC
AAAATTTAACATCTGTTCATCACCCCTCATCCTCGGGTGTAATTCCTGGCAGCCATGCCCCTCTAAATGGCAAGACCTCT
TCTTCAGAGCCCTTGTGGGCAGAGAAGCTCGAGCTCCCTTCATTCCAAAGTCAGATGGCAAGTTGGGGCTTATCTTCTTC
CCCCCTTCCTTCACTTGAGTCTGTCGACACTATGATTCAGTCCCCTCCAACTGAGCATACTGAATCATGCAATTTGTCAC
CTAGGAACAGCGGTTTGTTGGACGCTGTACTTTATGAATCTCAAACTATGAGAGCTTCAAAGAGTATCTTGCACCAGGAG
AATTCTGGTGATGTGGTTGATAATTCATGTCCTGATCTTCACGAGACTGGATGGGAAACATACGGTGATCCAATCTCTCC
ATTAGGTCATTCTGCTGCATCAGTGTTTAGCGAATACACTCCTACCAGTGGAAGCTCCCCAGAGGAGCCCCAATTGGTGA
CAATGCCAGGTTGCAAGGTTAAGCAGGAGAAGTTTGATTTCGGACCCTATGATGGGAAGGAAGATGCATCAGACCTGATC
TTTTCCCGGCCAGACTACTTGCTCGAATCCAATTGTTTTGGTCATATGCAAAAAACCGCAAGATCGATCTGGCACTGA

>CcMYB20 |Locus_ID=BC332_01521 |CDS_len=1530 |strand=-
ATGAGCATCACAAGTGAAACCGATGATATGATGACATCCAAAGTTGACATGGATTCGCCAGATGAAGCTAGTGGTGGAGA
AAGTGTACCACTTAAAAAAGGTCCCTGGACTACTGTGGAAGATGCGATTTTAGTGGATTATGTCATGACAAATGGTGAAG
GGAACTGGAATGCTGTCCAGAGGCATTCAGGACTTGCTCGTTGTGGTAAAAGTTGCCGTTTGCGGTGGGCAAATCACCTG
AGACCAGATTTAAAGAAAGGTGCATTCACTCCAGAGGAAGAGCGGCGGATACTTGAACTACATGCTAAGATGGGAAACAA
ATGGGCACGAATGGCTGCTGAGTTGCCTGGCCGCACAGATAATGAGATAAAGAATTACTGGAACACTAGAATAAAGAGAC
GACAGCGTGCAGGATTGCCAGTTTACCCTCCAGATATTTCTTTCCTAGCAAATCAGAACAAACAAAATGAGGAGTTGGGT
GCATTCTCCTCTGTAGATGCCCAAAATTCTAATGTCTTGGGAATTAACAATTTTGAAATTCCTGCTGTGGAGTTCAAAAA
TTTGCAGCTCAATCATCTGTTGTATCCGCCACCACTTGGTGAAATTCCTGCTGTAAGTAGCTTTCTGGCCCAGGGTCATA
GAGCTCCCTATGGTAGTACGTATTTGCTTTCAACAATGCATCCCTCAAAGCGTATACGAGGATCAGAATCGATGTTCTCC
GGTTCAAATGGTGATCTTCTCCTCAGCTCCTCACAATACCATAATGGCGGTTCTTTGCTTGCTCAACCCTTGGGGTTTTC
TTCATATAATCATCATTTAACATATGATGATGACCGATCATTCTCAAGTGTAGTTCATGGCGGCCATGCCTGTTTAAATG
GCAACTCCTCTTCTTCAGAGCCCACATGGGCAATGAAGCTGGAGCTCCCTTCACTCCAAAATCAGACAGCAAACTGGGGC
TCACCTCCTTCCCCTCTTCCTTCATTAGAGTCGGATGATATTCTGATTCAATCCCCTCCAGCTGGAAACAGTGAATCAGG

TAGTCTGTCACCTAGCAACAGCGGCCTACTGGATGCTGTGCTTTATGAATCCCAAACTATGAAAGCTTCAAACGATAACT
CACACCAAGGGAAGGAGACATCTGGTGATGCAGTCAATAAAGGATGGGAAAGTTATGGGGATCCAGTCTCTCCTTTACAT
CATTTCTCTGCATCAGTTTTTGGTGAATACACCCCTGTCAATGGAAGTTCATTACATGAGTTCCCATCAGTGGCCACAAT
GCCAGGATGCAAGATTAAGAAAGAGATTGGCGATCTAGCCCCCCTGGATGAGAATGATGACTCTTTGAACCAGACAATCT
TTTCCAGCCCCAAGACACAGCATGCTAAGAACAGTCTGGCATTGAAAGAAGTAATTAGTTCTGGCTTTTTCGATGATTGT
GGCTGGGATTGCAAGCAAATCCGTGCAGTAGCAACATCATCAGGTCAAGCTTGTGGATGCAGTTCTTGGGATGCCATGTC
AGCTGTGTAG

>CcMYB21 |Locus_ID=BC332_31637 |CDS_len=1461 |strand=+
ATGGCTACAAATGATAGAGGAATGAAGAACAGCGGAGGAAAAAACGGAGGAGGAAATAGACAGGTGGTAAAGAAAGGTCC
ATGGACAACAACAGAGGACACAATTTTAATGGAGTATGTGAGGAAGCATGGAGAAGGGAATTGGAATGCAGTTCAGAGGA
ATTCTGGATTGATGAGATGTGGAAAAAGTTGTAGGTTAAGATGGGCTAATCATCTGAGACCTAATCTTAAGAAAGGTGCA
TTTTCTCCTGAAGAAGAAAGAATAATTATTGAGCTTCATGCTAAAATTGGCAATAAATGGGCTCGCATGGCTGCTCAGTT
ACCTGGACGAACAGATAATGAGATCAAGAATTACTGGAATACGAGGCTAAAGAGAAGACAAAGAGCTAATTTACCTATAT
ACCCTCAAGAATTACAATCACAAAACCAACCAGAGCATAACCAAGCCCAACAAAGCCTTATTTCTTCAGCATTTGATCCC
CAAAGGACAACTTATAACAATCCTCCCTCTCTTTCACTTTTGGGTATCTTAAATCCTTCAAGCATGAGACCTCCTATTAC
TCATCAATTTCCCCTAAATGGTAACAAAAATTTTCGCAATCCTCCTAAAGGCCTTTCCATGACACCTCCCTCATCTATGC
AAAATTCACAATTTTCATCATTACCAGTATCTGTGTCTAACAACAATTTTGGTCACGACTTGTTCAATTCAACGCCAATG
CCATCATTTCAACAGACCTATCCAGATTTTAGCTCAACAATGAGGCCATTTTTGGGGAATATTCCTTCAAACCAAAATGG
ATTAATGTTTGATATGGGTATTAATTCTATTAATTACCCCTCAGGGCAATTATCTATGCCTGTAACAACTTCATCATCTT
CTGAGAATACAAGCAGTGGTTTTGGTTCAAGCGATGCTAATAATTATACAAATGTTGGTGGATTATCACGAGGAAATAGT
GGATTATTGGAAGATTTGTTGGAGGAGTCTCAGACTTTGACTCGAGCGATGAAAATTGAGGAAAGAGGAAAGTCATTATG
GGAAAATTATGGATTAGTGGAAAAAGAGTTAGGAGATCATCAAGATGGAGCCATTTTTACTGAAGAATCAGTTTATAGCT
TTGCTCATGGAGATGATGTCACCCTGTATAACAACTCTGAAAGCTCCAGCTCAAATCCCAATGCATCGCCAGGGACAATT
TTTAAAAAGGAAGGTTCACTAAAAGGAACTAATCAAGTGGATGAAGATCTTATGTGTCTTCTTGATAGTTTTCCTCTAGG
TGTATCAGTTCCAGATTGGAATGACCATAATTCAAAAATTGCTTCTGATGATTACAAAGCAGAAAATCTGACTGAAGATT
CTATCAATTCGCAAGTAATAATAACAACAAATTCAGGGTCAAAGAAACATGAATTGGGACTTGAAGGGTGTTGTTGGTAC
AACATGCCTTCCTTTTGTTAG

>CcMYB22 |Locus_ID=BC332_24264 |CDS_len=1440 |strand=-
ATGGCCCCAAACGGAGGAGGAGTGAACAAGAACAATAATGGGACATCAATAGGAAGAAATCACAATACAAGGCAAGTACT
AAAAAAAGGTCCATGGACAACAACAGAAGATGCAATATTAATGGAGTATGTGAAGAAGCATGGTGAAGGGAATTGGAATG
CAGTACAAAGGAATTCAGGTTTAATGAGATGTGGTAAAAGTTGTAGGTTAAGATGGGCTAATCATTTGAGACCACATCTC
AAAAAAGGTGCTTTTTCTATAGATGAAGAAAGACTTATTGTGGAACTTCATGCTAAACTTGGAAACAAATGGGCTCGTAT
GGCTGCTCAGTTGCCTGGTAGAACGGATAATGAAATCAAGAATTATTGGAACACGAGACTAAAGAGAAGACAAAGAGCTG
GATTACCAATATACCCTCAAGACATACAACCACAAAATTACCAACAAGATCAAAACCAACAACATAGTACTAATATCCCT
TCACCATTTGATAATAATCACCAAAATTCCAATTACAACAATAATAATTCCCCTCTTTCCCTTTTAGATATCTTCAATCC
ATCAACTATGAAACCTAGTAGAAACATTATTCCACATCAATATCAATTCAACAATAATAACCCTACTTCTTCATTTCTCA
CAAATACAAATAATATCAACAATCAAGTCAAGTTTTTCCGAGACCCTCGCGTTAGCCTTTCTCTAACATTAGCATCATCG
ATGAAGAATTCACAACTTCCTTCAATGATAGCGCCCGTGCCTAATAATTTTAGTCAGGGCTATTCTAGCTCAATCCCGGC
TCCACCACTTCAACATAACTATCCAAATTTCACCACAACTACAAGACCTTTTCCCGGAATTTCTTCAAACCCTAATGGTT
TAATCTTAGGTATGGGCACAGAGCAACGTTCAATCCAATTGTCCATGCCTGAGACTACAACAACAACAACATCAAGTGAT
CATGCTGATAATAATTACGTTGTTGAACGTGGATTATCGCGCGGTAATAGTGGATTATTGGAAGACTTATTAGAGGAATC
TCAGACATTAACAAGAGGTGAAAAAATAGAGGAAAATTGTAATGTTGATAATGAGGATAATAAAGGGAAATTAGTGTGGG
AAGAATATGGATTAACAGAAGAAGCAGCAGATGCAATTTTAACTGAAGAATCAACATATAATTTTGCTCATACTGATGAT
AATAATGCTACACAAAGTAAACATTCTGAAGATTCAAGTTCTCTCAATTCATCCTCAGGGATAACAACAAAGGAAGCTTC
ATTAGAGTTGGGTAATCAAGTGGATGATGATATTATGAGATTTCTTGATAATTTTCCACTAGGTGTACCTGTTCCTGATT
GGTGTGATGATCAACAAAATACATCAAATGCCCAATCATTTGAATGTGACCAAATTCAATTGTCAAAATCAAGTTCATGA

>CcMYB23 |Locus_ID=BC332_06584 |CDS_len=783 |strand=+
ATGGTTTGTGAAATAATTAGTGAGCAAATGGGGAGCAACTGGGGATTTATTGAAGAAGTATGGAGAAAAGGGCCATGGAC
TGCTGATGAAGACAGATTGTTAATTGAATATGTCAAGTTGCATGGTGAAGGCAGGTGGAATTCTGTTGCTAGGCTTACAG
GATTGAGAAGAAATGGAAAAAGTTGTAGACTAAGATGGGTAAATTATTTGAGGCCAGACTTGAAGAGGGGGCAAATAACC
CCAAATGAAGAGAAGATCATACTTGAGCTTCATGCTAGATGGGGCAATAGATGGTCAACTATTGCTCGAAGCTTGCCAGG
GAGAACGGATAACGAGATCAAAAACTACTGGAGGACGCATTTCAAGAAGAAGACCAAGAACTCATGTGACAACTCTGAGA
AGTCACGTGCAAGTCTTTGGAAAAGACAACAATTCCAACTGCAGCAGCAACAACAACAACAAATCAAAAATCAAATCGAC
ATCAAAAAGGTCATGTCATTACCAACTATGGCTCAAGCAAAACAAGAAATGGCCATCTTGTACCCAAACATCTACACAAT
TGATCAACAAGATCAAGTTGGGTTGTTCTACTCGATGCTTAATAACTCGACCTCTATATCACAGTCTGAACCCTCTTCGT
CGAATGAAGATATCATGTGGGATGACTTATGGAACTTGGATGATTTTTATGGCCATTTTATCAACTCTACAACTTATAAC
AAAACCAACACTAGTCCTTGCCTTCAACCTATGGCTAGTACTACTACTCCATCTTTCTATTAA

>CcMYB24 |Locus_ID=BC332_05804 |CDS_len=774 |strand=-

ATGGTAGCTATGATGGGGTGGGGAGCTAATTCTGATCAACAAGGATGGAGGAAAGGGCCTTGGACTCCTGAAGAGGATAA
GATGCTTTCTGAGTACGTTAAGTTGCATGGTGAGGGAAGATGGAGTTCTGTAGCTCGATGTGCAGGTTTGAACAGGACGG
GGAAGAGTTGCAGGCTAAGGTGGGTGAACTACTTGAGGCCTGGACTTAAAAGAGGCCACATAACACCTCAAGAGGAAGGC
ATTATTATTGAATTGCATGCCTTATGGGGCAACAAATGGTCAACTATAGCCCGTTACTTGCCTGGAAGGACAGACAACGA
GATAAAAAATTACTGGAGGACACATTTCAAGAAGAAGGAAAAAGCTTCAGGAAAGCAAGACAAGAGGAAAATTATACGAC
AGAAAAACCAACCACAACCAAACTGCACCATAGCCACGATTGATAAAAAGTTGTCACCTCAAGCAGAAGAAGTAGTGATC
ATGAAGAGTGATGACAGTAACGCGTATACTAATTATTACCATAAGAATATAATGGATCAGGATCCAGTTGTGGAGTTGCC
ACCAGTGACAACTTGTTCAGACACATCGTATGCATGGACGGATACTTTTCTGATGGATGGTTTATGGGGAGGATTATGGA
ACTTAGATGATCATTCACTGATGCAGGCAACTAATGACAAGTGCAAGGTGGCAAATATCCAAAATCAACCAGCTAATTGC
ACTTATGCAACTGATTATGCAGTTAACTTACATAATGGAGGTTTTATTTTCTAA

>CcMYB25 |Locus_ID=BC332_14907 |CDS_len=717 |strand=-
ATGGCACAAGAAGAAATGAGAAAAGGACCATGGACAGAACAAGAAGATCTTCAGCTAGCATTTTATGTGAACTTATTTGG
CGATCGACGATGGGATTTCTTGGCCAAAGTTTCAGGTTTGAGAAGAACAGGAAAGAGTTGTAGATTACGTTGGGTGAATT
ACTTGCATCCTGGTCTTAAACGAGGAAAGATGACTCCTCAAGAAGAACGCCTTATTCTTGAACTCCACTCCAAATGGGGA
AATAGATGGTCAAAAATTGCCATGAAATTACCGGGCAGAACTGATAACGAAATCAAGAATTACTGGAGAACTCACATGAG
GAAAAAGGCTCAAGATGAAAGGAAAAACAAGGCTTCTACTATTTCTCCATCTTCATCCTTCTCCAACTCTTCATCTTTCT
ATTCCAACAGTCCTGATGTCGACTTCACGCCCATTATGGAAAACAATGAAAGGAACTTCTATGATACAGGCGGAGTTGAG
AAAAAGATTAATGATGGTGGCAAAATGAAGGTGTACTCCATGGATGAAATATGGAAAGATATTGAATTATCAGAAGAAAG
TGAGACAATGAATAATATTAAACCAATCATGTCATCACCTTTATGGAATTATTGCCCCAATAACACACTATGGATGAGTG
ATGAAGAAGAAACTAAGATGTTTCCTCAGTTTTATAATTGCTTGGACAACCAGGATAACACATTTTTTGCTGGCTAA

>CcMYB26 |Locus_ID=BC332_14908 |CDS_len=567 |strand=+
ATGGCACAAGAAGAAATGAGAAAAGGGCCATGGGCGGAACAAGAAGATCTTCAGCTAGCATTTTATGTGAACTTATTTGG
CGATCGACGATGGGATTTCTTGGCCAAAGTTTCAGGTTTGAGAAGAACAGGAAAGAGTTGTAGATTACGTTGGGTGAATT
ACTTGCATCCTGGTCTTAAACGAGGAAAGATGACTCCTCAAGAAGAACGCCTTATTCTTGAACTCCACTCCAAATGGGGA
AATAGATGGTCAAAAATTGCCATGAAATTACCGGGCAGAACTGATAACGAAATCAAGAATTACTGGAGAACTCACATGAG
GAAAAAGGCTCAAGATGAAAGGAAAAACAAGGCTTCTACTATTTCTCCATCTTCATCCTTCTCCAACTCTTCATCTTTCT
ATTCCAACAGTCCCGATGTCGACTTCACGCCCAGTACAGAAGATAATGAAAGGAACATATACGATACAGGTGGATTTCAA
AAGAATGTTGCAGAAAACATGAAGGTGTACTCCATGGATGAAATATGGAAAGACATTGAATTATCAGAGGAAAATGAAAC
AATGTGT

>CcMYB27 |Locus_ID=BC332_17123 |CDS_len=687 |strand=-
ATGGACAGAACAAGAAGATCTTCAGCTAGTATTTTATGTGAAGATGTTTGGAGATCGTCGCTGGGATTTTCTGGCGAAAG
TTTCAGGTTTGAGGTTGGTTGGAGGGAGAGAAAAAATAAACTAGGTTTGAAAAGAAGTGGAAAGAGTTGCAGATTACGAT
GGGTTAATTACTTGAATCCTGGTCTCAAACGAGGCAAAATGACTCCTCATGAAGAACAACTTGTTCTTCAACTCCACTCC
AAATATGGAAATAGATGGTCAAAAATTGCGGGGAAATTACCGGGAAGAACTGATAATGAAATCAAGAATTACTGGAGAAC
CCACATGAGGAAGCTAGCACAAGATGAAAAGAAGAACAATAAGGCTTCAATTTCTCCATCTTCATCCAACTTCTCCAATT
GTTCATCTTCCTCTGCCAACAGTCCTGCTGTGGACTCCAGTACAGAACAAAACGAGTTGTTGCAAGTTGTTGAAGCAAAT
AAAACATATGAAGAGGATCAGAACATGATGGTATACTCCATGGATGAAATTTGGAAAGAAGTTGAATCATCACAAGAAAT
TGAAACAAGGAATGATTTACCAGTTATGGCATCTAACACCTGGGATTATTGTCCAGATTCGCTATGGATGACTCATGATC
ATTATTACTATGACAATACTCAAGATTTCTCATTTTTTACTGGCTAA

>CcMYB28 |Locus_ID=BC332_22951 |CDS_len=621 |strand=+
ATGCAAGAAGAGAAATTACGAAAAGGCCCTTGGCTCGACGAGGAAGACGAGCGACTAGCATTTGTTGTCTCCATTTTAGG
TGAACGTCATTGGGATGCGTTAGCAAAAGCTTCAGGACTCAGGAGAAGTGGAAAAAGTTGCAGATTGAGATGGATGAACT
ACCTTAGACCTAATTTAAAACATGGCCAAATTACTGAAGATGAAGAAAATTTAATTATTAAACTTCAAAAACAATTGGGA
AACAAGTGGTCAAAGATTGCTAAACAATTGCCAGGAAGAACTGATAATGAAATAAAGAATTACTGGAGAAGTCATTTGAG
AAAGAAAGCACTTATTTATGAACAAGAATGCTTTGGAAGCAATAATACAAGTGGTGATTCACAACCAAAATTATCATTTG
CAAAAGATGATAATTGTCAGAGCTTTGATGATGGTGAAGAAGAATCCTCAAGATTATTGGATTGGATGATACCAAGTTGG
ACATATGAACAAAGTCAAATGGAACATCACATGTACCTTTGTAAAATACTAAACCTATGTTCTTGTAATTATTCTTCTCA
AGATGAAAATATTAGCAGTACATGGGATGATGCTTCATCATCTTCTATTTGGGAATACTAG

>CcMYB29 |Locus_ID=BC332_26333 |CDS_len=621 |strand=-
CAGTTAACCATGCAAGAAGAGGAACTTCGAAGAGGGCAGTGGCTTGAAGAGGAAGATGAGAGACTGGCTATGATTGTAGC
CATTTCAGGTGAACGTCGTTGGGATGCATTAGCAAAAGATTCAGGGTTGAAGAGGAGCGGGAAAAGCTGCAGATTGCGAT
GGCTGAACTACCTCCACCCAAATCTAAAGCACGGTCATATTACTGCAGATGAAGAACGTTTGATAATCCGACTTCAGAAA
CAGTTTGGAAACAAGTGGTCAAAGATTGCAAAACAATTGCCTGGAAGAACTGATAATGAAATCAAGAACTACTGGAGAAG
TCATTTGAGAAAGAAAGCACCTGTTTATGAACAAGAATGCTTTGAAGATAATACAAGTAATTCAGAACAAAAGTCATCAA
TTTCGAACTGCGCTACTGTCCTTAATTCATCCAACAGTGATGACTCTTTTTCTGAAAAAGGTGATTGTTACTCTGCTGAT
TCTTTGGCAAATGAAGTAACATTATTGGATGGGATTCCAAGTTGGTCATATGAACAAAGCAGAATGGAGCATCATATGTA
CTTTTGCAGCTCAAACCTATGTTCTTGTTATGCGCCATGGACCTCTGAAGACAATAATTAA

>CcMYB3 |Locus_ID=BC332_04434 |CDS_len=1038 |strand=+
ATGGATCGGGTTAAAGGTCCATGGAGCCCCGAAGAAGATGAGCTATTACAACAGCTTGTTCAGAAACACGGGCCGAGAAA
TTGGTCTTTGATTAGCAAATCGATTCCGGGTCGATCCGGTAAATCTTGCCGGTTACGATGGTGTAATCAGTTATCTCCCC
AAGTTGAACATCGGGCTTTTACACCGGAAGAAGATGAGACTATTATTCGGGCTCATGCCCGATTTGGTAACAAATGGGCT
ACCATAGCCCGATTACTTAACGGGCGAACCGATAACGCAATAAAGAATCACTGGAATTCTACGTTGAAGAGGAAGTGTTG
TTCGTTGAGTGCTGATGAAGGTAAAGAACTTGCTGATCAGATTTTTGAAAATCATCAACCGCCGTTGAAGAGATCCGTTA
GTGCTGGATCCGCTATGCCGGTTTCGGGTTTTCATTTCAGTCCGGGTAGTCCGTCGGGTTCGGATAGTGATTCGAGTCTT
CATGTTACTTCGTCGTCTCAATCTCATGTGTTTAAGCCTGTTGCTAAAACTGGTGGAATTTTTCCGCCGCCGTCGATTGA
TATCTCATCTCCATCAATTGATCCTCCAACTTCTCTTAGCCTTTCGCTTCCCGGTGCTGACTTGGCTGAGTTATCATCTC
GTTCGGCCGAGTCGACTCAGTCGAAAAATCCGTTTCAACTGTCGCTGCCTCCACCGGTGCAGGTTCCACCACCACCGCCG
CCTCCACCTCCACCACCGCAGCCTCAGCCAGTGACGGCGGTGCCGTTCGAACAGCAAGACAAGGTATTTGTGCCTTTTAG
CCAGGAGTTGTTGAGGGTGATGCAGGAGATGATAAAGACTGAAGTGAGGAACTACATGATGGGAATTGAACAGAAGCAGC
AGCATCCGCAGCAGCAACATTATCAACAACAGCAGCAATTTCAGCAGCAAACTCATCAATTGCCAAGTGGTTTAGGACTT
GGCTTATGTATGCAACAAGCTTCTGATGGATACAGGGATAGGGCTGCTAATCGCATGGGAATAAGCAAATTTGATTAG

>CcMYB30 |Locus_ID=BC332_14493 |CDS_len=696 |strand=+
ATGGAAGCATCAATGGAGATGAAGAAAGGGCCATGGAGTTCAGATGAAGACTCTAAACTCATCCATTCCATCTCCTTACT
TGGCCAAGGACGTTGGGATTCTCTCGCTCGCTTTGCTGGGTTGAAGCGTAGCGGTAAGAGTTGTCGATTGAGATGGTTGA
ATTATTTGAGACCAAATTTACGACGGGGAAAAATAACTCCTCAAGAACAACTCCTTATTATACTCCTTCACTTTCGCTTT
GGAAATCGATGGTCAAAGATAGCGGAGAACTTGCCAGGAAGAAGTGATAACGATGTAAAGAATTACTGGAGAACCAAAGT
GCAAAAACATGCGAAAGAGCTTCATTGCAACGTAAATAATAGCCACCAATTTCGACACTTTCTTCTCTATCAATGGCTTC
CATCTCTGCTGGCTCAACAAATATTGCCTACTACGCCACCTTCTTCTCCTAACAACACGATGACATGTCCAAATGCTGAT
TTCATCTCATCACCAGAAGATGATAATACTAGATATAATATTTGGAACAACTTGTCTAGTTTTGAAGATGAAATTAATAT
TTCTCATGATTATTCAATCCCTACTGATGATGAGTACTACTGTCACAATTATTGGCCAACTACACCATCACAAGTACTAT
TACCAGATGAAATGCTATGTTTGGATGATGATATGGACCTCTGGCTCTTTCCTTAG

>CcMYB31 |Locus_ID=BC332_19185 |CDS_len=597 |strand=-
ATGGTGAGAACACCTTGCTACGACGAAAATGGAATGAAGAAGGGGACATGGACTCCTGAAGAAGATAGGAAGTTAACAGC
ATATATTGCAAAATATGGCTCATGGAACTGGCGCCAACTTCCCAAGTATGCTGGACTAGCAAGGTGTGGAAAGAGCTGCA
GACTTCGATGGATGAATCACTTACGACCAAATGTTAAAAGAGGGAATTATACCAAAGAAGAAGATGAAATCATCTTGAAC
CTCCATGCTCAACTTGGAAATAGGTGGTCGGCGATTGCTGCTCACTTACCAGGAAGATCAGACAATGAGATAAAGAATCA
TTGGCACACAAAACTTAAGAAGCGCGGTACTAATTATGCGACAAACTCAAGTGATGAATCAAGCAAGAAATGTAAGAATA
ATACTAAGAAGAGGTATACTGAAAGTAATACCAATAAAAATACAAGTCATAATAATATGCAGGAAAATATAGTACTGGAA
AGTTCAGAATGGTCACCAAAGGAATCATCAAGTGAAGAACTCTCCTCTTACAGTACCACTAATTATCAACAGCAACATAA
AGTGTTTCAAGAGGAAATAACTAGTGGAAGCTTTTGG

>CcMYB32 |Locus_ID=BC332_03892 |CDS_len=591 |strand=-
ATGGATAAAAGACCATGCAACTCTCAAGATGCTGAAGTGAGGAAAGGACCTTGGACTATGGAAGAAGATTTGATTCTCAT
TAACTACATTGCTAATCATGGTGAAGGTGTTTGGAACTCCCTAGCTAAATCTGCTGGTCTCAAACGTACCGGAAAAAGTT
GTCGGCTCCGGTGGCTTAATTATCTCCGGCCTGATGTCCGGAGGGGCAATATTACACCTGAAGAGCAACTTTTGATTATG
GAGCTGCATGCTAAGTGGGGAAACAAGTGGTCGAAAATTGCGAAGCATTTACCTGGAAGAACAGATAACGAGATAAAGAA
CTATTGGAGGACTAGGATTCAGAAGCATATTAAGCAACCAGAGAACGTGAATGGACAAGGAACTTCAGAGCAAAATATTG
ACCATCAAGAAGGAAGCAGTAGCCAAATGTCCTCTGCTGGTCAAGTAGATACCATAGAGACATACTCTCCAACTTCATAC
AATGGAAATATGGACGCTAATTTTCAAGCCCCTAATTTTCTCACTGAAACAAATGACAATATGTGGAGCATGGAGGATAT
CTGGTCTATGCAGCTGCTCCATGGAGATTAA

>CcMYB33 |Locus_ID=BC332_05936 |CDS_len=510 |strand=-
ATGGATAAAAGAACATGCAATTCTCAAGATGTTGAAGTTAGGAAAGGTCCTTGGACTATGGAAGAAGATTTAATTCTCAT
CAATTACATTGCTAATCATGGTGAAGGTGTTTGGAATTCCCTTGCTCGATCTGCTGGTCTGAAGCGTACCGGAAAAAGTT
GTAGACTCCGGTGGCTTAATTATCTCCGGCCAGATGTCCGGAGGGGAAATATTACACCTGAAGAACAACTCTTGATCATG
GAACTACATGCCAAGTGGTCAAAAATTGCAAAGCATTTGCCGGGAAGAACAGATAACGAGATAAAGAATTATTGGAGGAC
GAGGATTCAGAAGCACATTAAGCAAGCAGAAAACATGAACAGATTATCATCAGATATTTCTGATCAGCACAATAATGACC
AACAAGCAAGCACAAGCCAAGCATCTATTGGTCCTCCAGATACCATTGAAACCTACTCTGGAAATAATAATAACAACAAT
AATATTATGGGCAGTAATTTCCATCATCAG

>CcMYB34 |Locus_ID=BC332_27190 |CDS_len=864 |strand=+
ATGAGTAGTAATAATAATAATAATAATTTATCATCAAATGAAGATGAATTATTTGAATTAAGAAGAGGTCCATGGACACT
TGAAGAAGATAATCTTCTTATTCATTATATTTCTACTCATGGTGAAGGTCGTTGGAATGCTTTAGCAAAATGTGCTGGAT
TGAAGAGAACAGGAAAAAGTTGTAGACTAAGGTGGCTGAATTATTTAAAACCAGATATTAAACGTGGAAATCTCACCCCA
CAAGAACAACTCTTGATTCTTGAACTTCACTCCAAATGGGGTAATAGGTGGTCGAAGATTGCTCAGCATTTGCCAGGAAG
AACGGACAACGAAATAAAAAACTATTGGAGAACAAGGGTGCAAAAACAAGCAAGACAACTCAAAGTTGATTCAAATAGCA

AGAAGTTTGTTGAAGCAATAAAGAATCTATGGGTACCAAGATTACTTGAAAAAATGGAACAATGTTCTTCATCATCCACC
ATTGACAAAAAACAAAATATTAATAATCTTTCATTTCCAAATTTACCCTTAATCAACAATCAAGAATCATGTACAAAGGA
CAACAAGTCATCACATGATGATGATAATACTAATACTAATAGTAATAACAACACAACATGGGGTTCACTAGGAAGTTCAA
TGAATGAAAATTTCAATATTTGCAACAATTCAATGCATCAAGATGATGAGTGTTACTATGTGGAGAATAATTTCAACCAG
CAGCCACAACATTTTTCATATCAAGAAATGTCAATATCAGAATGTGATGAGGTGGCAGAAGCTGATTGGTTTACTAATGA
AGGATCATTGTGGAACATGGATGAGCTTTGGCAATTCAAAAAGCTAGGAGATGTAGATATTTGA

>CcMYB35 |Locus_ID=BC332_09701 |CDS_len=882 |strand=-
ATGGAAAGAGACATGTCTTTCTTGTCTAAAACTTCAAGTAGCAGCTCTAGTGATGATGATATTGAATTGAGGAGAGGTCC
ATGGACTATTGAAGAAGACAATCTCCTTGTCCATTACATTACCAATCATGGGGAAGGCCGATGGAATATGCTAGCTAAAC
ATGCTGGTCTGAAGAGAACAGGCAAGAGTTGCAGATTGAGATGGCTGAATTACCTTAAACCGGACGTTAAACGAGGAAAT
CTCACTCCACAAGAACAGCTCATGATTCTTGAACTTCATTCCAAGTTGGGCAACAGATGGTCAAAAATTGCGCATTATCT
ACCGGGGAGAACGGATAATGAGATCAAGAATTACTGGAGAACGAGAGTGCAGAAACAAGCGAGGAATCTGAAGATTGACG
CGAATAGTGCAGCATTTCAAGAAATGATTCGTTATTTGTGGATACCGCGGCTTCTTCAAAAGATACAGAGCTCCTCTTCT
GTTATGCCATCAATTCAAAGTCCACAATCAACTCCACAAGATTTCAACATAGAGAAAAACAGCACCAACACCAGCAGCAG
CAGCAGCTGTTCAAGTTCACATTCGTCGAATATCTATAATTCTACCAACATTATGTCAGAATGTCCTAAAATTCCACCAC
GCGACATTGGCGATTCTGACTTAAATTCATTTGTCAAGGCTCATTTTCCAGTTGATGACAACAGTTATGATGTGGATAAT
TATGGACAATTAGCAACTGGAAATTACTATAACGATGTGATGGATTATGACCAAATGAGTGGAGATCAAAACAAGCTGAC
TGGTGATGTTTTATCTGACAGCTTTTGGAGCAATATGGATGAATTTTTCTACCAGAAACAGGATATGAGCCATTGTAGAT
AG

>CcMYB36 |Locus_ID=BC332_31414 |CDS_len=879 |strand=+
ATGAATCATTACAGTCAATACGTCGACAACAATCAAAAAAGTGATGAAGAAACTAATAATTTGGACCTTAAAAGAGGTTC
ATGGACTATTGAAGAAGATTTCACTCTTATGAATCATATTGCTCTTCATGGTGAAGGTCGTTGGAATTCGCTCGCTCGTT
CTGCAGGTTTAAAGAGGACTGGAAAAAGTTGTAGATTAAGATGGCTTAATTATCTTAGACCAGATGTTCGACGTGGAAAT
ATTACACTTGAAGAACAATTATTAATTCTTCAACTTCATTCACGTTGGGGCAATAGGTGGTCGAAAATTGCGCAGCATTT
GCCGGGAAGAACGGATAACGAGATCAAGAATTACTGGAGAACGAGAGTGCAAAAACATGCAAAGCAATTAAAATGTGATG
TGAATAGCAAACAATTCAAAGATACATTGCATTATCTATGGATACCAAGATTAGTTGAGAGAATTAAAGCTTCAACAAAT
TCAAATAATAATCAAGTGAATAATATTCAACAAACAAATTATGACAATATTATTAGTAACAATAACAATAATAATAATAA
TAATAATAATATTGTCCAAAACATTATGAATAATTTCAAACAAGAAAATTATTCTAGTACAACAACAACTTCATCAGAAA
ATTCATTTGGGAGTACACAAATTTCAGACATGTCTGATAATTGTTACACTAATTACCCTCTTAATCAAGGTGATTATCAT
GGGGAAATATTAATTAATCCCACAAGTGATTATTTTCAACATGGTATTATTGATGATAATAATCATCAACAAAACATGAG
CCAGTTATTTTTTGATGATGTTTCAGACAAGTTTTGGAACCAAGAAGATATGTTGTTCTTACAACAACAACTCAATTGA

>CcMYB37 |Locus_ID=BC332_31413 |CDS_len=810 |strand=+
ATGGATAAAATTCGCGAAAATAATATTGTTGAGCTTAAAAGAGGTCCATGGACAGTTGAAGAAGATTTTATTCTTGTGAA
TTATATTGCTCATCATGGTGAAGGTCGTTGGAATACGCTCGCTCATTGCGCTGGTTTAAAGAGGACGGGGAAAAGTTGTC
GATTAAGATGGCTGAATTATCTTAGACCTGATGTTAGACATGGAAATATTACACTTGAAGAACAATTATTGATTCTTCAA
CTTCATTCACGTTGGGGTAATAGGTGGTCGAAAATTGCTCAGCATCTGCCAGGAAGAACGGATAACGAGATCAAGAATTA
CTGGAGAACTCGAGTGCAGAAACATGCAAAAACCCTAAAATGTGATGTGAATAGCAAACAATTTCAAGATACATTGCATT
ATATTTGGATACCAAGACTAGTTGAGAGAATTCAAGCATCCAAAGGTCAAAATAATAATTCGATTAGAGTAGCTCAGCAA
TCCGTTCTAAGAACAGGTATAACTGAGCTCGTGCCAATTCAACTGGCGAACAACAGCAACAACAACAACGACAATAATAA
TAGTAATTATTTAAGCGCGAGCGTTACACCGGAGAATTCCAGCGTGGCAACTTCATCAGAAAACTCAATTGGAACAACAC
AAGTTTCACATAATCAAGATTATTATCAAGCTAATCAAAGTGATTATCATCATCAATTATGGTTTGAAGATAATTATCAA
GCAATGGATCAACAAAATAGTATACAATTATGGATGGATAATGAAGATGTTTTTGACAATTTGTGCAACATTGAAGACAT
TTGGTCCTAA

>CcMYB38 |Locus_ID=BC332_08081 |CDS_len=840 |strand=-
ATGGGGTGCAAGGCAGCAGAGAAGCCAAAGCAAAAACACAAGAAGGGATTATGGTCTCCTGATGAAGATGATAAGCTCAA
AAATTACATCATTAAACATGGTCATGGCTGCTGGAGCTCTGTTCCCATTAATGCAGGCTTGCAAAGGAATGGAAAGAGTT
GCAGATTAAGGTGGATTAATTACTTAAGACCTGGCTTAAAGAGAGGGGCATTTAGTTTAGAAGAGGAAGACACAATCTTG
ACCCTTCATGCCATGTTTGGCAACAAGTGGTCTCAGATTGCACAACACTTGCCAGGAAGGACGGATAACGAGATAAAGAA
TCACTGGCACTCATATTTAAAGAAGAGAGTGACCAAAATGGCAGAAAATGAAGGCGACACTAAATCTGATAACACAGAAT
CCTCACCTTCTTTAAAGAAATTGACTCCCCAGAATTCTAGTTTGGATTCATTTGAACACAGAGAAGGATCATTAGCAGAT
TCAGATCAATCTATTTATCCAAGAGAGTCTCAGAAAAGTAATTTACCCAAAGTATTATTCGCGGAATGGCTTTCGTTGGA
TCAATTTAATGGGCAAGAGTTCCACAACTCAGGGAATTTTGAACCTTCCACGAATAATTTTGGGTATAATAATTCAGAGT
TACATGACATATTCATGCATAGTTTACCCATGAACAATGGTAGCTATGGGAATGGTGTAAATCAAGAAGTTAACAATGGG
ACAGTAGTAGATGATATTTTTCAAGCCCAACTCAAGTTTGAGGATCCCATGTCTGCTAACGGATTTGAGGAATTCATGTC
AGGGGAATTCAACATTAACGACGATGTCATGTACATATGA

>CcMYB39 |Locus_ID=BC332_02119 |CDS_len=843 |strand=-
ATGGGATGCAAGCAAATGGACAAGCCAAAGCAAAAGCATAAGAGAGGATTATGGTCTCCATATGAAGACCATAAGCTTAA

AGACTACATCCTCAAGCATGGCCATGGCTGCTGGGCATCCGTGCCTATTAATTCTGGATTGCAAAGGACTGGAAAAAGTT
GCAGATTGAGGTGGATTAATTATTTAAGGCCAGGTCTAAAGAGAGGAACATTTAGCACTGAAGAAGAGGAGACAATTCTG
ACCCTTCATGGCATGTTAGGAAACAAGTGGTCTCAAATAGCACAACATTTACCTGGAAGAACTGATAATGAGATAAAGAA
CCATTGGCACTCTTATCTAAAGAAGAGTGTGTCCAAAATGGCTAAAACATATAGTGAAAATGCAGAAGCTTCATCTTCTT
CCATGAAATTGGTTTCCAAAAAATCAAGTAATCTGGGCTCCTTTGAACTCGTAGAAGGTGCACTATTAGCCGATATAGAT
CAATCCACTTTACATGTTCAACTTCCACAAGAAAATCGCGAATGTAATTTACCCAAAGTGTTATTTTCGGAGTGGTTCTC
ATTAGATCAGTTTAATGTTCAGGATTTTAAGAACACTATCAAGCAACATCATTCTAAAAACAACTATGGATGCAACGATT
CAGTGTTCCAATTAGATGCTTTTGCTCAATATGGTCCACTACTAGCGATGAATGAAGCTATTACCTATGGGAATGACATA
AATGGCGGTTTTAACAATATGTTTCAACCACCTCTCAAGTTTGACGATCAAATCTTTGTCAATGGTTTTGAGGAATTTAT
ATCCGGGGAATTTAACGTGAACGGCGGTGTGATGTACATATGA

>CcMYB40 |Locus_ID=BC332_02384 |CDS_len=1101 |strand=+
ATGAGGAAGCCGGAACATAATAGTAGTAATCCATCCCCATCGACAATGATGAAGGAGAAGGAGAAGGAGAAAGGGAATAA
GGCAAAGTTAAGAAAAGGGTTATGGTCACCAGAGGAAGATGAGAAGTTGATGAATTACATGTTAACAAATGGGCAAGGAT
GTTGGAGTGACATTGCTAGAAATGCTGGCTTGCAAAGGTGTGGCAAGAGTTGTCGTCTTCGATGGATTAATTACTTGAGG
CCAGACCTTAAACGAGGTGCTTTTTCTCCACAAGAGGAAGAACTCATTGTTCATTTGCATTCTATTCTTGGAAATAGGTG
GTCACAAATTGCGGCTCGTCTCCCGGGAAGAACAGATAACGAAATCAAGAATTTTTGGAATTCCACAATAAAAAAGAGGC
TGAAAAACAACAACAACAACAACAATCACAGTAACAACAGCACGCCATCACCTAACGCCAGTGATTCTTCCTCAGATCCT
AGGGCTCTCGTGGGAGGAATATTCCCTATGCAAGGGCACGATGTAAATGTCATGGCAGCCTTCTGCATGGATAATTCATC
GTCTACTACCTCAGGGTCATCCATGCAAGCCATGGTTCCTAGCAACCATTTTAACCCTTTCCCTCAGCTTGACAATACAA
GCTACGATATCTCAGGATTAGTCGGGCCAGTAAATTTAGGTCAATTTGGTTGTAACGGTTGTGATGGTGGATTTTTGGAT
TATGGAGTTGAGGAGACTTATAGTATGATGGGGTTAGAAAGTGATGAATTTTCAGTACCCCCACTAGGGGGTGTTCATAA
GAGTACTATGGGGGAGAATAATAACAATAATAGTCATGTCAATTTTAGTAGTAACATTGTGATTGGAGCCAATGACTATA
TGGTTGAGAAGAAAATTACAAATGGTAACAACAACAACAACCAGCACTTGATGAATATGAGTGGGAGTAGTGATCAAAGC
CTAAAGGTTGAGGACTATATGGTTGGTTTTGGGAATCATAATCATTGGCATGGGGAAAGCTTAAGAATTGGAGAATTTGA
TTGGGAAGGTTTGTTGGCAAATGTTTCCTCTTTACCTTACCTTGATTTTCAAGTTGAATAA

>CcMYB41 |Locus_ID=BC332_28534 |CDS_len=1074 |strand=-
ATGAGGAAGCCTGAGTTCTCCTCCTCATCCTCCTCCGGCAAGAACAATAACAACAACACGAATAATAATACAAACGTGAA
ACTAAGAAAAGGGTTGTGGTCGCCAGAGGAAGATGAGAAGCTCATGCATTATATGCTGACAAATGGACAAGGGTGTTGGA
GTGATGTAGCACGGAATGCTGGATTACAAAGATGTGGGAAGAGTTGCAGGCTCAGATGGATTAATTATTTGAGGCCAGAT
CTTAAGAGAGGTGCATTTTTACCTCAAGAAGAAGAGCATATAATCCATTTACATTCCCTTCTTGGTAACAGGTGGTCTCA
AATAGCTGCACGTTTGCCTGGACGTACTGATAATGAAATCAAGAATTTCTGGAATTCAACCTTGAAAAAGAGGCTAAAGA
ATTCATCGTCATCCTCTACACCATCACCAAATGCAAGTGATTCATCCTCAGATCATCTCTCCAAAGACCTCAACATAGGA
GTTACTCAACAAAGATTCATGACAATGCAAAAACATAACCTAATGTCCATCAACATGGACTCAACTTCATCTTCTTCCTC
GTCTATGGCCCTAAACACGATTAATATTGATCCTTTACCCACATTAGACCATACCTTAATTAACATGTCTAATGGATTTA
ACATCTCGCCCTCATACTTGAGTACTACGCAACCATGCATGGTACAAGGAGGGAATATTGTGGGTACTAATAATGGTGGA
AGTCTATTTTATGGGAATAATCATGGGATATTTGGAGGAAATCTTAGTATGGAAGGCCATGAGCTCTATGTTCCACCATT
GGAGAATGTAAGTATTGACTATAATCAAAATGTTGAAAATGGGAATTATGTTGAAAGGAGCAATCATCATCAAAACAACA
ATTACGTTAATAATATGAGCAGCTTGAATAGTACTAGTCATAATTTCAATAGTAGTAGTAATATCAAAGTAGAAAATTTT
GGAGGGATTGGGAATTATTGGGAAGGAGATGAACTAAAGGTGGGAGAGTGGGACTTGGAGGAATTGATGAAGGATGTTTC
ACCTTTCCCTTTTCTTGATTTTCAAGTTGAATAA

>CcMYB42 |Locus_ID=BC332_25321 |CDS_len=996 |strand=-
ATGGGCCATCACTGCTGCAGCAAACAAAAAGTTAAAAGAGGTCTTTGGTCTCCAGAAGAAGATGAAAAACTCATTAGACA
TATCACCACCCATGGTCATGGCTGCTGGAGTTCTGTTCCAAAACTAGCAGGACTGCAAAGATGTGGAAAGAGTTGTAGAT
TGAGATGGATAAATTACTTGAGGCCAGACTTAAGAAGGGGTTCATTTACTGAACAAGAAGAAAGAACAATTATTGATGTT
CATAGAATCTTGGGAAATAGATGGGCACAAATAGCGAAACATTTACCAGGTAGGACAGACAATGAAGTGAAGAATTTTTG
GAATTCTTGCATCAAGAAGAAACTTATTGCTCAAGGGTTAGATCCAAATACCCACAACCTCCTCAAGAACAAATCTAACA
ATCCAACCAAAAAATCCGACTACACTTATCATCATCAAGATTCCACCTCAGTTTTCACCATTGATACTTCAACAAATAAA
GAAGTAATTTCAATGGATTTAAAATCAACTCTTGCAACCTTATCTCCTAATTTCCCTCCTAGTGATAATATTATTACTTC
TAGTACATACAATTACACTCCTATTGTTCCTAACATTGAATACCAAAACCCTACTTTCGCATGGAATTCCATTTTGGATT
TCGCAAGTAATTGTTCTTTAATGGAAAGCACGACGAATATTAATCTGTCATCTTATATAAATGAGAATAAAATGTGGAAT
GGAACTGGACTTGAACCAACAACATTAAATCCTGTCAATGGACAAGAAGAAGAAATGCAAGTACAATTACAAGGGGATCA
ACAATTTCCAATTATTCAGACCAAGTTTTGTCATGATCTAGAAGATGTGTACAAGGTTAATGATCATGATCAAACAGTGG
AAAATACATTTGACGACAACTCTAACTTTGATTTTGAGTTTATGAATTCTGCATTTGTGCCTTGTGGATTGTACACTAAT
GATAATTCTATGGATCAACTTTCATGGGATTGTTAG

>CcMYB43 |Locus_ID=BC332_09604 |CDS_len=1146 |strand=+
ATGGGGCATCATACATGTTGTAGCAAACAAAAAGTAAAGAGAGGGTTATGGTCTCCTGAAGAAGATGAAAAACTCATCAA
GTACATTACTACTTATGGTCATGGTTGTTGGAGCTCTGTCCCTAAGCTTGCAGGACTTCAGAGATGTGGAAAAAGCTGCA
GATTGAGGTGGATAAATTATCTGAGACCAGATTTGAAACGTGGGAGTTTCTCACCTCAAGAAGCAGCACTTATCATTGAG

CTCCATAGCATTCTTGGCAACAGGTGGGCACAAATAGCAAAGCATTTACCTGGAAGAACAGATAACGAAGTGAAGAATTT
TTGGAATTCGAGCATCAAGAAGAAGCTCCAGCTTTCACATGGAACATTCTCTGATCTCTCCATCATCTTTCCTAATAATA
ATAATCTCATTGCTAATCCAAACCCTAATCCCTCTTTTGATAATTCCAACTCATTCAATAACCCCAATGTTCCTATTACT
AATAGTTTCATGTCAAGTACAAATAACTCATCTCCCCTAATTCAACAACTTGACCAAATGATTAATTTTCCTAATGGGGA
TTTAAATTTAATCAATCAAGTTGTGCCCTATTCCCTTCCTATGTCATTTGACTCAATGTCAAATGATCCAACATGGTTTA
ATTTCAATTATCCTCATCATCTTCAACATGATCTTGAATATAACCCTAGTGTTATTAAACAAGACAATTCCAATTCCATG
TTCACCTCCAACATTTCTTTGAATTGTACTAATGGGGAGAACTTCATGGATTCAACAAACGCGGATATCAAAATTCATCA
AGATTTAATGATGGCGCCTTTGCTGTTGCCCAAGCTTTCTGAAACGATAAAGGGGAATGAAGGTGGCATTCTAGAATCAA
CTCCTGTTGCGTCACAATACATTCACTCAATGGCGATCAGCAGTCTCCCATGTACCATCCCGACTGGTTATACTATGAAT
CCACATAACAATAATAATGTGCACCGAGTCGTCCATGAAATGAAGCAGATAGAATCCGTACACATGTCATCATCACCATC
TTCATCTAAATCTTCAGCATCAGGTTCATCATCATTGTCAGCACTTTCATGCAGTCAATTTATGATGAACCCTAGTGGCA
ACCTCCCTACAACTTGGGATTCATAG

>CcMYB44 |Locus_ID=BC332_00146 |CDS_len=1017 |strand=-
ATGGGGCACCATTCATGCTGTAATCAACAAAAGGTAAAGAGAGGACTTTGGTCCCCAGAAGAAGATGAAAAGCTTATCAG
ATATATTTCTTCTCATGGTTATGGCTGCTGGAGTGAAGTCCCTGAGAAAGCTGGGCTACAAAGATGTGGAAAAAGTTGTC
GTTTGAGATGGATCAATTATTTGAGGCCTGATATTAGAAGAGGAAGATTTACCCCTGAAGAAGAAAAATTGATTATCAGT
CTCCATGGTGCTGTTGGCAACAGATGGGCACATATCGCAAGTCATTTACCCGGAAGAACAGATAACGAGATAAAAAATTA
CTGGAACTCTTGGATTAAAAAGAAGCTGAAAAAGCCTTCAAAGCCATCAGCAAACACAACTTCTATTACTGATCATCATC
ACCAGCAGTCACATCAAAGATCTCAATTGGTTAATTACAACACAATTACAAGCCAACAAGACATATTTTTCACTCAAGAT
CTTGGAACAAAATCTCAAGTACTCCTCCAAGATTCTACCCTATTCAATTCTCCAAGCCAATTGTTCTTTTTCGACAGTGG
TTCACTCGACTCTATGACAAATGCTATTATTAATGATGCCACAAATCGAAGTACTACTACTAATAATGCCTCATTGTTTC
AAGAAACATCTACATTGAACTCAGAGTTCTGCTGGCAAGTCGATCAACAACAACAACAACAACTTCAAGTACAGACATCT
TCGTACGCGATAGGGATGGATTCGAATTATTTGCCACCATTGATAGAGAGTATGGTACCACCTATGGAAATACCTCGTAA
TAATAACAACAACAACAATATTATGGTGGAAGGGCAAGAAAATAATAATCAATTGGATGAATGGAGTAGTCAAGTGGACA
CACAACAATGTTGTCCAAGTTATCTATTTTGGGATCAAGAAAATGGATCAATTGGTGGTGATCATGAGATTAATATAGAT
CCAACTACATCAAACAATATGGGACAAATATTATCTTCTTTTCCTTCTTCTTTATGA

>CcMYB45 |Locus_ID=BC332_08924 |CDS_len=999 |strand=-
ATGGGGCGCCATTCTTGTGGTGTGAAGCAGAAGCTAAGAAAAGGATTGTGGTCACCTGAAGAAGATGAAAAGTTAAGCAA
TTATATCACCAATTTTGGTATTGGCTCCTGGAGTTCAGTCCCTAAGCAAGCTGGCCTGCAAAGATGTGGAAAGAGTTGCA
GATTGAGATGGATAAATTACTTGAGGCCTGATCTTAAAAGAGGAATGTTCTCACAAGATGAAGAGGATAGGATACTTGGT
CTCCATCAAGTTCTTGGAAACAGGTGGGCACAAATTGCTGCACAACTACCAGGGAGAACGGATAATGAGATAAAGAATTA
CTGGAATTCGAGTTTGAAGAAGAAGCTAATGAAGCAGGGGATTGATCCAAACACCCACAAGCCATTAAGGGAGAATCAAG
TAATCAAAGATGAAGAGAATTGTACTAATAAGACCTCAATGTTTGAGGTACCATTTCTTAATGAAATGGACCAACCATTT
ACACAATTAGCTTCTTCAAAAGACCAATATGTGAGTAAACAAGTCTTTGATCTTCTATTTGCTCATGAATTCCAATCCAA
TGAGAATAATTCACAGACGTTAGCTCAGTACCATGATCATCATCATCAGAGCGAATACGAGGAAAATCCAAATTATGTGT
TTTGTTCAGCATCAAGTGTATCAAAATTAGAACATGGACAGATGACAGATACTGATTTTGCTAGCAGCTCAACTTCAAGA
ATGAGTTCAAGCAACAGTTCAAATATGTGTAGCAATCAAAACACTGCTAGAATTCGGACGGAGAACAGTGAAGCCTTGTC
ATGGGATGTTGAGAACAAGATGGAATCTCTGTTCCAGTATCCCTACATAGGAATCAAGAATGAAGAATTAAGGTCAAGTC
CAAGCCAAGAAAGAGAGCTATATGGTCACAGTTCTGGAGATTTCATGAGTAATTATCCATTAAGTTCCTTAACAGAAGAA
TTAAGTGGGGCTAATCTAGATGTCTTCAAGCAAATTTAA

>CcMYB46 |Locus_ID=BC332_16679 |CDS_len=963 |strand=-
ATGGGGCGCCACTCTGTTTATGTTAAAGAGAAGCTAAGGAGAGGATTGTGGTCACCAGATGAAGATGAAAAATTGTATAA
CTATATCACTAGATTTGGTGTTGGCTGCTGGAGTTCAGTCCCTAAGCTATCTGGTTTACAAAGATGTGGAAAGAGCTGCA
GATTGAGATGGATAAATTACTTGAGGCCTGATCTTAAAAGAGGAACATTTTCACTAGAAGAAGAGGATATGATTATTACT
CTCCATGAAGTTCTTGGAAACAGATGGGCACAAATTGCAGCAAAATTACCGGGGAGAACAGATAACGAGATAAAGAATTA
TTGGAATTCATATTTGAAGAAGAAGCTAATAAAGCAAGGGATTGATCCAAATACACACAAGCCACTAAGTGAAAATCATC
AAGTAAGAAATGACACAAATTGTACAGACAAGGCCTCAATGTTGCTGATGTCCACATTCCCAAATATGTCAAATTCAGTT
GAAATAGAGCAACCATTTCACTTTAACAAAAGAATTTTCAACAGTGAGGCAATTACTAGACAGTTAACAGAAGTTTCAAG
AAACCAACTTGTGAGTAAACAAGTCTTTGATCCTCTATTTCTGTATGATTTCCAAGCAAATATGAATCCAATTGGACCTT
ATGATGGGAATCAAGATTTTGGGATTTGTTCAATGCCAAATTTAACAAATTTTGAACATGGACACATGACAGAAAGTGAT
TTTTCTGACAGCTCAACTTCAAGAATGAGTACAAGCAACAGGACAAATACTATGATTAGACATCATAGCTCTGCTGGAAT
TCATCAGATGAATGAAATGTTGGAAAATTCTCAAGAATTGACATGGGATGCCGACAACAAAATTGATTCTCTGTTTGAGT
ATCCTTACGATGGGATCAAGAATGAAGAAGATTTCAGTAATAATAATAATAATAATAATAATCCATTGAGCTCTTTACTC
TGA

>CcMYB47 |Locus_ID=BC332_22066 |CDS_len=1263 |strand=-
ATGGGGAGGCATTCTTGTTGCTACAAACAGAAGTTAAGGAAAGGCCTTTGGTCACCTGAAGAAGATGAGAAACTTATAAA
ACATATTACTAAATTTGGTCACGGCTGTTGGAGCTCTGTCCCTAAACTAGCAGGTCTTCAGAGGTGTGGAAAAAGTTGTC
GGCTGAGGTGGATTAACTACTTGCGACCTGATTTGAAAAGAGGAACATTCTCACAGGATGAAGAGAATTTGATCATTGAA

CTTCATGCAGTTCTTGGGAACAAGTGGTCGCAAATTGCAGCTAGATTGCCTGGAAGAACTGATAACGAGATTAAGAATCT
GTGGAACTCGTCAATTAAGAAAAAGTTAAGGCAAAGAGGGATTGATCCAAATACTCACAAGCCACTTTCTGAGGTTGAGA
GCGAAGAGAAAGCGTCGGCAAACAGTAACAATAAGAACAATGACAAAGTTTCTGAAAGTTCAAATAATGAGTTCAATTTT
GTTGAGGCTCATGAAAATGGATTCTCAACAGAAAAGGGTATAAATAATATGGAACGTTATCCACTTATCGATCATCAGGC
CAATAATATTGCCCCACCAACTCATGAATTCTTCACAGCCAATTGCAAGTCACCTGATTTGTCTAGTTATCTCTCCTTTC
ACAATTATAGTCCCAACACCAATATTCTCTTCAATACCAAAAACTCTTCTGCTGACAATATGGTATCTGATCATCATCAG
TTCAATTGTAGCACCTTGCCAAATGCTACCTTTTCCACTATGTCATCGAACTCGATTCTCAGTACATCCCCGTTGGCACG
TACTTTTAATATCAACAAGTTTCAGAATTGGGAAGCATGTACTATCAGCAGCAACGGCAGCAACAACAGCAATGGCAGCA
GTAACAGTATCGAATTACAAAGCAACTGTTCATTCTTCGACAACAGTAATGCTGCTGCTGCTGCTTTCACTTGGGGATCA
ACGAGCGCAGCAGCAGCAGCAGACGGTTCAGGGAAACAGGAGAGGGAAGAAATTAAATGGTCCGAATATATGCAAACCCC
GTTTTCACTAGGTGTTAATAATAACACAATCCAGAATCATCATCATGAAATTTCTCCTCATCATCAAGATTTGTACGACG
GCGAAACAAAGTCTGAGACACAATTCATGACACATGGCACATGGCTTCAGAATCAAACGCCGCAGACTTCTCTGCAAACT
GCTGATTTATACAGTAATAATAATTTCCAAAGGCTCCCTGCCGTCTTCGGTCAATTCTCTTAG

>CcMYB48 |Locus_ID=BC332_25434 |CDS_len=669 |strand=+
ATGGCGCAAGAGGAAATAATGAGAAGAGGACCGTGGACAGAAGAAGAAGATCTGCAGCTTGTATTTTATGTTAAATTATT
TGGAGATCGACGATGGGATTTTCTGGCCAAAGTTTCAGGTTTGAAAAGAACAGGAAAGAGTTGCAGGTTACGTTGGGTTA
ATTACTTGAATCCTGATCTCAAACGTGGCAAGATGACTCCTCAAGAAGAACGTGTAGTTCTTGAACTTCACTCCAAATGG
GGAAACAGATGGTCAAGAATTGCCCGAAAAATACCAGGGCGAACTGATAACGAAATCAAAAATTACTGGAGAACTCATAT
GAGGAAGAAAGCTCAAGAACAAAGGAAAAAGTCTTCTATTTCTCCATCTTCATCATTTTCCAACTCGTCATATTCTTCTT
CAATCTCTCACGAAGAAAATGAGAGAAATTTTTATGATACCGGTGGACTCGAACTCTTGAAAGCTGAAGGAGAAATGAAA
TCTAGTGATCAAGAACAAGGTGGAGAAAGTATGAAAATTTACTCCATGGATGAAATTTGGAAAGATATTGAATTATTAGA
AGAAAATGAAGAAACAACGAATAAACCAATTATGGGGTCTATTTCACCCCTATGGGATTATTCTCTAGACTCACTTTGGA
AAGATTTTGATTGGAGTAGCTTTCGATAA

>CcMYB49 |Locus_ID=BC332_11766 |CDS_len=993 |strand=+
ATGGGGAGAGCTCCTTGTTGTGACAAAAACAATGTCAAGAGAGGACCATGGTCACCTGAAGAAGATGCTAAGTTGAAGTC
CTATATTGAGCAAAATGGGACTGGTGGAAACTGGATTGCTTTGCCTCAAAAAATTGGCCTTAAGAGATGTGGAAAGAGTT
GTCGGCTTAGGTGGTTAAATTATTTGCGGCCAAACATCAAGCATGGAGGATTTTCAGATGAAGAAGATAGAATTATATGC
AGCCTCTACATAAGTATTGGAAGCAGGTGGTCAATAATTGCAGCACAACTTCCTGGAAGAACTGATAACGATATAAAGAA
CTATTGGAACACTAAGCTAAAGAAGAAGCTATTTGGAAAACAACGTCAAAAGCAAGGTTCAAGAAAAGGAAAAGAAATCA
AATCCATGTCATCAATTTCCACTTCCAATAACCTGAACCAAAACCCTTGTTGGCCTGAGCTTCAACCATTACCATACTCA
AACAATGAACCAAGATTCATTAACAATGACCATACATCCATCAGAAAGCTATTAATCAAGCTTGGAGGTAAATTTTCAGA
AAATGACCAGTTGACAAACGTTGTGCCAAATTCTCATGAATATCCTATCGATAATTCATCATCGATGCAACTGATGTATC
AGGATCATATCAATTTGATCTCTTCAGCTCCAATAGATCACAATGTCTTCAACATCACCGCACCAAATATTGATCTATAC
AACATGGATGGGGCAGCTAGCAATTTTACGGCTGAATTTGAGCATATGATAAACACTAGTCAACAAAAAATAGATGGTCT
TGAATTTTTATATGAGAATCTGGTTATCGATAAATTTACTTCTACTTCTGGAGGAAATTTAGACTGGGAATCGATGAATC
CTTACGTGCTTCCTCTCCCTCCTATTGTTGCTTCTAGTGATGGAGGTTTTCAACAAGGTGTTATATTTCAACAAGGTGCA
CTTGATGAGCTAAGATACCCCATGGTACAATAA

>CcMYB5-1 |Locus_ID=BC332_08856 |CDS_len=558 |strand=-
ATGGGTGAAGCACCTAGTTATTGCTCATTAAAAAACTATGACTCGAAAAATGAGGACCACTACAAACAAATCGTTGGTTG
CTTTGACGAAGACTTTCATGACATGAAAAAGGATTTGTGGACGGAGGAGGAAGACAGGATATTAATCGAAGCACATGCAG
AAATAGGAAACAAATGGGCAGAAATAGCAAAAAGGCTTACTGGTAGAACTGAGAATTCCATAAAGAACCATTGGAATGCT
ACTAAAAGAAGGCAATTCACAAGACGAAAATGTCGTACCAAATGGTGTAGCCCAAGTTCAATCCTCCAGAATTACATCAA
AAGTTTGAACTTCGAAAAAGGTAATAATAATTCTGCACCTAAAATAGAGCCAATTGATCAGCAAGTTCCTGCAGATAATT
ATGATTTTGGTGAATTCGATCTGGAAGACAAGCTGTTTGATGATTTGGAGAATGTTCCTAATTATTGTGAATTGGCTCAA
TTGATCATGCAAGATGCTGAAGTAAATAAGGAGGTTGACTTGATGGATTTCATTAGTCCTAATCCAGAGAATGTATGA

>CcMYB5-2 |Locus_ID=BC332_08857 |CDS_len=687 |strand=-
ATGGATTTTGATCCTAGCAGTAAACAAGATGTTTTTTGCACAATCCCTACAGATTATCTTCATGATTTTCATCACCTCGA
TCTTACTTTTTCGCTCGCTGGAGGTCCATTAACCTTCAACACGAACCCTGATCACATGATCGAAATGGATAGCGGTTATG
AGCACCCGTTATTCGATCCACTCTCAAATCATGGTGATAACTTCTCATCAATGGGTCAAGATTTCAACTTTGGCTATGAA
TTCAAGCCATTTGAGCAAAATGGCAATGCTAGTGGTAGCACACTATTCATGAAGAATTTTGAGAGCTCTACGGATATGAA
GTCCATCAACTTCAATGTTGTGCGTGATGAAAGTTCATGTGTTACTAGTACTATTGAAAACAATGGTACTAATAATGTGT
ACAATGATAAAGAAATTGGTGGAAAAAAGAATAAGAAGCTTATAAAAAGCAATGATATAAAATCATCTTCTTCTTCATCT
ATGAGAAAATCAGGAAGGGGTACTCGAAAAAAATCAAAATCAGCCAAAGATCAATGGACTATTGAAGAAGACAGGATTTT
GATCAATTTGGTAGAGAAATTTGGAGATAGAAAATGGTCAGAAATAGCAAAAATGTTGAAAGGGAGAATAGGGAAACAAT
GTAGAGAGAGGTGGAACAATCATCTTAGACCTGATATCAAAGTATGA

>CcMYB50 |Locus_ID=BC332_17980 |CDS_len=1029 |strand=-
ATGGGTAGAGCTCCATGTTGTGACAAAGCAAATGTGAAAAAAGGACCATGGTCACCAGAAGAAGATGCAAAACTCAAGGC

TTATATTGAAGAAAATGGCACTGGTAACAATTGGATTGCTCTTCCCCAAAAAATAGGGCTTAAGAGATGTGGAAAGAGTT
GCAGGTTAAGATGGTTGAATTACTTAAGACCTAATATTAAACATGGTGGATTTACTGAAGAAGAAGACAATATCATTTGC
AGTCTCTATATTAGTATTGGAAGCAGGTGGTCGATAATAGCTGCACAACTTCCTGGAAGAACAGATAACGATATCAAGAA
TTACTGGAACACCAGACTGAAGAAGAAGCTTCTTGGAAAAAGAAAACAATCTCAAATGAATAGATTATTGCTTGCTAATG
GTGGTCAAGATCTTAAAGAACTAAATGGATTAGAAGAAAATCCATTATTACAAAACCTAAGTAACTCAGCTCTTGAAAGA
CTTCAACTTCATATGCAACTTCAAACCCTAACAAACCCTCTTTCTTTTTATAATAATCCATCACTTTGGCCTAAGTTAAC
CCCTCTTCAACAAAAAATGATCCAAACCCTACAAGTTAATGGCTTAAATGAGGATCAACCACCTCTTTACGCGCCGATAA
ACCCTAGTGAAAACCATGCTCAAGAATTAGGGCAAAAAGTTGGGATGAATGAATTTGCTAGTACTAACTTCAAGGTGAAT
GATCAAGTGGAAAAAAGTAGTCCAATTGATGTTCTTGACACGAACGTAGGTCAAGAAAACACGAGAGATCAGATCCAGAT
TCAGGGCGTTCAGGGTTTTACTCAGCTGGAGATCGATGACCTAATTCTCAGCAACAAGGGATCGATAGGCATGAATATGA
ACTCCGAATTCGATTTGGCATGGTGGTCTAATGAATTTGATACAAATAGAGCTTCAGCATCAAATTCTTGGGGTTCATCT
TCAAATATTCTTCAAAATACTCATGATGAAATGTATCAAGATTATGCACTAGGGTATAATTTGCAATAA

>CcMYB51 |Locus_ID=BC332_22966 |CDS_len=822 |strand=+
ATGGGAAGAGCTCCATGTTGTGATAAAGCAAATGTGAAGAAAGGGCCATGGTCACCTGAAGAAGATGCTAAATTAAAAGA
ATATATTGACAAATTTGGCACTGGTGGAAATTGGATTGCACTTCCACAAAAAGCTGGGCTAAAAAGATGTGGGAAAAGCT
GCAGATTAAGATGGCTTAATTATCTTAGGCCTAATATTAAACATGGAGAATTTTCAGATGAAGAAGATAGAGTCATTTGC
AGCCTTTATGCTAACATTGGAAGCAGGTGGTCGATAATAGCAGCCCAGTTACCGGGGAGAACAGATAACGATATCAAGAA
TTATTGGAACACAAAGCTTAAGAAGAAATTAATGGGATTTGTCTCTTCATCTCACAAGATTAAGCCTCTTAATCATCATC
ATTATCAACACCAAATTCCCACTAATTATAATTACAACAATTATTCCCCACAAATTTCACTTTTTCAAACTTCATCTTTA
TTAATTTCATCAAATTATAACACCAACACCAACAACACAACTTTCCCATGCTATGACATAAATGCCCCTATAAGTAATTT
CTTGAGCAATGGTGCATCAACTAGCTGTACCTCAGGCATTACTACTAGTACTTTCCAAGAAAATAATTTCGTGGGGCCCA
CTGCTACGTCTTCCGACGAGAGTTATGGCGTATCGAATTTAGATTTTCGTACCTATATGTATAATAACGGTGTTATTGGC
GAAGAAGGAGAGGATCAGGGTAACTCGAAATTCTTGATCAGTGGAAATAATGATGGTGGTGGTTGTTATGTTGATGAGCA
ACAAAATCCATTAGATTATAGT

>CcMYB52 |Locus_ID=BC332_17052 |CDS_len=1002 |strand=-
ATGGGAAGAGCTCCTTGTTGTGATAAGAATAATGTGAAAAGAGGGCCATGGTCACCTGAAGAAGATGCTAAGCTTAAAGA
ATTCATTGACAAATATGGAACTGGTGGAAATTGGATTGCTCTTCCTCAGAAAGCTGGATTAAAGAGATGTGGAAAGAGCT
GCAGATTAAGATGGCTAAATTATCTAAGGCCAAATATCAAACATGGTGATTTTTCTGATGAGGAAGATAGAATTATATGC
AGCTTGTATGCCAACATTGGAAGCAGGTGGTCAATTATAGCAGCTCAATTACCTGGAAGGACTGACAACGATATCAAAAA
CTATTGGAACACAAAGCTTAAGAAGAAGCTCATGGGTTTTATTCATACATCATCTAACCAGAGAATTAAATCACCAAATT
TATTCTTGCCTCCTACAACTTTTCCACCCCAAATCCAATCCCAAGCAATTTCAAATATTTTTAAAGATGAATATTTAGAG
CCCATTTCATCACTCCAACCAAATTTCATGTACCACAACAATAACATGAACTTTCAGCTAGGTACAAATAATCAATATTC
TTATTTTCTTGAAAGCTTAATGAATCCCATGCAAGCAAGTTGTTCTTCATCTGATGGAAGTTGCAGTCAAATTCCAAGAA
GTAATTTTGAAGAAACTCAAAAGTTTACACTGGATAATTATGGGAATAATGGAGGCACTTGGGCTGATCATGATCAAAAG
CCAGATGGATATTTTGGGAACAACCATCAAAATCATAGTAGTCAATTCCAGTATGATACTAGTAATATTGAAGAAGTTAA
GGAGCTGATTAGTAGTAGCTCTAGCAATGGGAATGGATGTAATAATAACAGCAATGTGTTGTTCTTCAGTGATGAAAACA
AGGCAAATATAGGGGCTGTTCCAGCCTTAACCAGAGGTCGAGGGTTCGACCCTGAGTATGGAGAAAACTCTGTTGGGAGC
GCTGCCACCTTAATGGGCCCTGTAAAGCGCAATCCGGATTAG

>CcMYB53 |Locus_ID=BC332_05620 |CDS_len=993 |strand=+
ATGGGTAGAGCACCTTGCTGTGACAAAGCCAATGTTAAGAGAGGACCATGGTCACCTGAAGAAGATTCCAAACTCAAGGC
CTATATTGAGCAACATGGCACGGGTGGTAACTGGATTACCCTTCCACAGAAAGTCGGTCTCAAGAGATGTGGCAAGAGTT
GTCGGCTTAGATGGCTGAATTATCTCCGGCCAAACATCAAGCACGGAGAGTTCACCGATGAGGAAGACACCATAATCTGC
ACTCTCTACATGAGTATTGGCAGTAGGTGGTCAATTATTGCTGCACAATTACCTGGGCGAACGGATAATGATATCAAGAA
CTACTGGAATACAAGGCTGAAGAAAAAGCTACTATTATGCAATAAGCAGCGAAAGGATCATCGGCCGCGGACCGGATACA
ATCACAATAAGGTAGATATGATGAAGGAACAGGAGGATTTCTTTGCTACTCATCAAACCACCAACAATGCATATTCTTGG
TCTCCGCAACAAATACTCTTCTCTACACTAATTGCACCACAAAATCATGACGTGGCAGAATGCAGTGCAAATAGCCACAA
CTTTCAATACTCCAGCACTGATCAAGCTTATTTCTCCCAAGATCAATTGTGTCAAATTAGCTCCACCAATCATCTTGCAT
CGATGAATCCGGTAAATGGCGCTACCTGTAATTCCTCAATGATGGTAAGCAGTGGCGCCTATTACCCGAGCAACGGGTTT
TGCAACAACGGTCTGCAAGAGTATAACAATTACATTTCCGTTGGGCTGGATCATGACGTGCTCAACAGTACTAGTACTGC
TCATTCTCAGCAAGTAGATAAAAGTGTATTGGAAATGGTCAATAGTAGCACTATTAGTACCACATCACAAGATCAAAGTA
CAAGCTGGGAAGAATTGAGCCCTCTTGTTAATTATCCTCCATCAGTATTCGAAACCGTATCACCTTATTATGTATTTGAA
GAGCCAAGGTACCTGGGGTTGTTGAAACAGTAA

>CcMYB54 |Locus_ID=BC332_24646 |CDS_len=807 |strand=-
ATGGGAAGAGCTCCTTGTTGTGACAAAACCAAAGTTAAAAGAGGACCATGGTCACCTGAAGAAGACAACATTCTCAAGAA
TTTTCTTGAGAAAAATGGCACTGGTGGCAATTGGATTGCTTTGCCTCAAAAAGCAGGTTTAAGAAGATGTGGAAAGAGTT
GCAGATTAAGATGGCTAAATTACCTAAGGCCAGATATTAAACATGGAGGATTTACTGAGGAAGAAGATAATATTATTTTA
AATCTCTATAATCAAATTGGAAGCAGGTGGTCAGTAATAGCTGCAAATCTACAAGGAAGGACAGATAATGATGTGAAGAA
TTATTGGAATACCAAATTGAAGAAAAAGCTTTTGGCTTCTCAAAATACTAATATTATAAGTACCAACAATAATATTAATT

CTAGTGATTTTAATCACATTAATTATTCAAGAAATTACTATGGGACATTGGATTATTCTAACACATTCACAAGTCATATG
GACCCAAATTTAACCAATTATTCAAATAATTGTGATCAATGCCCTCTTCTTCCTTCCTTGGTGGAAATTCAAGAAAATGA
TGCAACAATTCAATCGTCGGAATCATGTCAGATCCTCCAGAAGTGTGCAACTTTGAAAGAATCCGACACGCATCCTTCGA
CGTTTTTGCAGAATCCGAACAACATAGGCTATAATTATTACGATGATATTCTTGAAAATGGATTTGATTTTCAAGAAAAT
GCTATTGGAGGAGCTAATCCAAATTCTTCATATTACAATAATATTCTTGAAATTGATGATCAACTTTTCAAAGGATTTGA
AAATTAG

>CcMYB55 |Locus_ID=BC332_07302 |CDS_len=303 |strand=+
ATGGGGAGGACACCATGCTGCGATAAGACCAAAGTGAAAGGGGACAGTGGTCTCCAGAGGAAGATGAAATTCTCAAGAA
TTATATGCTTAAACATGGCACTGCTGGCAGTTGGATTACTCTTCCACATAAAGCTGGGCTAAATCGGTGCAGCAAGAGTT
GCAGATTAAGATGGCTGAATTATCTGAGACCAGACATCAAACTTGGAAAATTTACACAAGAGGAAGACAACATTATCCAT
TCTCTCTACTCTCAACTTGGAAGCAGGCTAAATCTTGCTCTTCAACCTTTATTTATTTCCTAA

>CcMYB56 |Locus_ID=BC332_24914 |CDS_len=1023 |strand=+
ATGGGAAGAATTCCATGTTGTGAAAAGGACAATGTCAAGAGAGGGCAATGGACTCCTGAGGAAGATCACAAGCTTTCATC
TTACATTGCTCAACATGGAACTCGTAATTGGCGTCTCATTCCTAAGAATGCTGGTTTACAGAGATGTGGAAAAAGTTGTA
GATTGAGATGGACAAATTACCTTAGGCCTGATCTCAAACATGGCCAATTTTCAGAAGCAGAAGAACAAACCATTGTCACA
CTTCATTCTGTTCTTGGCAATAGATGGTCTGTGATTGCTGCTCAACTGCCAGGTAGAACAGACAATGATGTCAAGAACCA
TTGGAACACAAAGCTCAAAAAGAGACTATCTGGTATGGGCATTGATCCAGTAACACACAAGCCATTTTCTCACTTAATAA
GTGAAATTGCAACAACATTATCTCCACCACAAGTCCCTCATTTAGCAGAAGCAGCACTTGGTTGTTTCAAAGATGAAATG
CTTCATCTTTTAACCAAGAAAAGAATTGGTTTTCAGTTTCATCAACAGTTTCCAAATAATGCACCAAGTACTAGTCATGT
CAAGAATGAAGAGAATGACACAGTTGAGAAGATCAAGTATGGATTGTCAAGAGCTATTAAGGGAAGTGACAAGAATTGGG
AGACAAATGGAGGAGCAACATCTAGTAGTTTTCATGATAATGGTGGATATAGTAACTATAACTTTGCTAATTTGCTTAAC
GACGAAGACGGTTCACCATGGAACCAAAGTTTGTGTAGTGGAAGTACATGCACAGCAGGAGGAGAATTACAACAACAACA
ACAACAACAACGCAACAAGAAAACTAGCAATGGTAATTGTGAGGATAGTGGTAAGGAAGTGGTAACAACAAGAAATGGAT
CAACGACAACTATGTTCAATTCCGACTGTGTTCTGTGGGATATTTCATCTGATGATCTGATGAATCCAATGGTGCTCCGT
GGAAGTAGTCAAGGTGAGCGCAAGCTGACCGAGACACCAGAGTCATTTAAGAAATCATTGTAA

>CcMYB57 |Locus_ID=BC332_10902 |CDS_len=999 |strand=-
ATGGGAAGGCCTCCTTGTTGTGACAAAGCAAATGTGAAAAGAGGCCCTTGGACTGCTGAGGAAGATGCAAAAATTCTTGC
TTATGTAGCCAGTCATGGAATTGGCAACTGGACTCTGGTCCCTCAAAAAGCAGGTCTGAATAGGTGTGGAAAAAGCTGTA
GGTTGAGATGGACAAATTATCTGCGTCCTGATCTTAAGCATGACAACTTCACATCTCTAGAAGAAGAATGCATCCTTGAG
CTTCACAAAACCATAGGTAGCAGGTGGTCTTTAATAGCAAAGCGATTACCTGGGAGAACAGATAATGACGTCAAAAATTA
CTGGAACACAAAGCTGAAGAAAAAGCTAGTGAACATGGGAATTGATCCTGTAACACATAAACCATTTGCCCAGGTCTTTG
CTGAGTATGGGAAAATTAGTGGTCTCCCAATTCAAAATGCAAAAAATCACATCCATTTGCCCAACAATACCACTGAAAAA
AACCATGTTTGTATGGAATCAAAGCAATTTCCATTCTCAAATGGGACTCCCCATGATAAGTTTGTTCATAAGATTATGAA
TGACCAGTTGCAAGAGAACTACACTAAAAATTACACATGGGATTCTAAGGAGACTCTTCAAACACATAGTTCCCTTTTGA
TTTCAACATCAACTTCTTGTAATCCCACAGTACACAGCTACTCCCCTTCTTTTTCTTCTGTGCAATCTCAGGTTCATACT
ATGCCAGCTTCTACGTCTACGTGTAATTGGAACGAGTTTATATTTGGAGACCCTTTTACATCATCAGGTGCAGAGCAAAA
ACAGGAGACATTTTTGTCAAACTTACTTCAGAAGGAGAATCCTACTTGTGGGGAAGTTACTGAAGTTGAGGAAAAGCAGC
TTGTTGAAGAAGCCACTTGTTCCTCTGCCATGGATTCATTCGTGGACACTATCTTGGCTAACGAAAAGCAAATGCTAATG
GATTTTCCACCACTTCTAGATGTCTACCTCGATTATTGA

>CcMYB59 |Locus_ID=BC332_07716 |CDS_len=753 |strand=-
ATGGAGGTGATTATAATTAAGAAGGGGGCATGGTCTCCAGACGAAGACCAGAAATTGAAATCTTATATCATGAGATATGG
CATCTGGAATTGGAACCTCATGCCCAAATTTGCAGGGCTTTCAAGGACGGGCAAAAGTTGTAGACTACGATGGATGAATT
ATCTTCGCCCTGAGGTAAAGAGAGGACCCTTTACCATAGAAGAAACGGAAACAGTAATCAAAATGTATCAAGAATTGGGA
AACAGATGGGCAGCTATTGCAAGGAGATTGCCAGGAAGAACAGACAATGAAGTTAAAAACTTTTTCCACACACACTTAAA
GAAGCATCTTGGACTGAAAATTGATCAGAATGCCTCAGTGAAGCTGTGTAAGGCCGTAAGACGCAAAAGAGTACTGGAAA
AGACCAAGAAAAGTGACAAGAAAAACTTAGCTCAAGAAAAACCTGTATTGTATCTGGATATTTCTGGAACATCCAGCAAC
AAGAGTAGTTTCACGATATCACCTAGTGGTTCTTCATCCAGTAGCAGCATTATTACAATCGACCAAAATCAAATGACCGA
TATTCATGTGGATCAACCAAATTCAATCATTGACGACATGGAAACTACCGTTCTGATCTTGGAAAGCAATCCTGCAATTA
GTACTCACCATGATCAGTCCTTAGACAGTTTGAGCATTGATTCATTGTATCAGTTTGACACGAGTTCATTCTGGCTTCAT
CTACTTAATGATGCCCATCGTCTTATTTTCTGA

>CcMYB6 |Locus_ID=BC332_16696 |CDS_len=1284 |strand=-
ATGGAACTTGATCAAAGTAACAACCAAAACCTTTCAATTCATCAGCCATTTTTTATGCCCAATAACAACATTTTGAAACC
AGAAATTGATGATGATTTTTGCATAGACTCTAAAGATTATTATCTTAAAGATTTTGAACACCTTGATCTTAATTTTTCTA
ATACCACCTTCAACAATTTTTCTCAAAATTTTATGATTGATGATGATACCAATGGTACTACTTATCATGACCCCTTTGAT
CCATTTTCAAATATTGAGGATAATTTTTCTTCGTTAGGGGATTTTAACTTTAGTAGTCATGAGCTTACTCCATTTGATCA
AGAAAATGGAGAGAGTGGTGGTAGCAAAGTAATGAAGAATAATTTTCATGATGATGAATTTATGAAGCCTAATTTGAACT
TTGTTAATGTGCCTGATGATCAAAGTTCATGTGTCACTGGTGATAACAATTTATCTTCATCTATTAAGAAAAAGCTAGGT

GGTAGAGGGAGGTCGAAGAAGTCGTCCAAATCGTCCAAAGGACAATGGACGATTGAAGAAGACAGGCTTTTGATCCATTT
GGTAGAGAAATTTGGAATTAGAAAATGGTCTCAAATTGCTCAAATGCTCAAAGGGAGGATAGGCAAGCAATGTAGGGAAA
GGTGGCACAACCATCTTAGACCTGATATAAAAAAAGATTTTTGGACGGAGGAGGAAGACAGGATCTTAATCGAAGCTCAT
GGTGAAGTGGGAAATAAATGGGCAGAAATTGCAAAGAAACTTCCTGGAAGAACTGAGAATTCAATTAAGAACCATTGGAA
TGCAACAAAAAGAAGACAATTTTCAAGAAGAAAATGTCGTACCAAATGGCCGAGACCTAGTTCTCTTCTCCAAAATTACA
TCAAAAGTTTGAATTTAGAAAAAGGAAGCACCAAAAAAGACACAAATCCCAATGGTCGCATGGAGTATACAGGTTCAGTC
GCTGAAAACAGAAATGAAAATTTGGCTAACAGGCCTAAGCGCCATGCTAATATAGCCAAAGTGAAGGTGTCGCCTAAGCT
AGAAGCGATTGAATTTTGCCAAGACAATAATGCTGGTGATTTTGATTTGAGCGAAGTGCCAGAATTTGCATTGGGTGATA
ATTTTTTTGATGAGAATAAATCAATGGATGCTTTTATGGAAGAGATTAATCCTAATGTTGGTACTTCCATTATGCCTTGT
AATTATGAAATGCCAACATTGATGATGCAAGGTGAAGTCAACAAGGAGATTGATTTGATAGAGATGATTTCTAGAGTTAA
TTAA

>CcMYB60-1 |Locus_ID=BC332_03169 |CDS_len=603 |strand=+
ATGTTTTCTAAATCTCCTGAACTTTGGTCGCACGTGGTTGCAGGGCTTTCACGTACAGGGAAAAGTTGCAGACTTAGATG
GATGAACTATCTCCACCCTGATGTTAAGAGAGGACCTTTTACCATGGAAGAAGTCGAAACAGTGATCAAAACATATCAGG
AACTTGGAAGCAGATGGTCAGCCATAGCAACAAAATTGCCAGGAAGAACAGACACCGAAGTTAAAAACTTCTTCCACACA
CACTTAAAGAAGCATCTTGGACTGAAAAATTATGATGCCCAACTGAAGACTAGGAAAGGCCACAAACAAACCCAAGAAAA
TGAGAAGGAAAATAGTACTCAAGGAAGACTTGTGCTTGAAACGATCAACAGCAGCAGTTCATTATCACCAGATGTTTGTT
CACCCTGCAGCAGCAGTATTACATGTAAAGAAAACCAAATGATGGATCAGTTCACGAATTTCTCACTTGTTGTCGATCAA
CAAGTTTCCCACGTGGAAAACACAAATATTCAGTCATGTTTCATTCCCGGTGGTTCTACCTTTAGCACCTTCGATCAATT
CGATATGAACTCATTCTGGTTTGATGTACTTGGAAATTTCTGA

>CcMYB60-2 |Locus_ID=BC332_07717 |CDS_len=594 |strand=+
ATGCTAAGAGTACCACAACAACAACAGCAGAAAGGGCTTTCACGTACGGGGAAAAGTTGCAGACTTAGATGGATGAACTA
TCTCCACCCTGATGTTAAGAGAGGACCTTTTACCATGGAAGAAGTCGAAACAGTGATCAAAACATATCAGGAACTTGGAA
GCAGATGGTCAGCCATAGCAACAAAATTGCCAGGAAGAACAGACACCGAAGTTAAAAACTTCTTCCACACACACTTAAAG
AAGCATCTTGGACTGAAAAATTATGATGCCCAACTGAAGACTAGGAAAGGCCACAAACAAACCCAAGAAAATGAGAAGGA
AAATAGTACTCAAGGAAGACTTGTGCTTGAAACGATCAACAGCAGCAGTTCATTATCACCAGATGTTTGTTCACCCTGCA
GCAGCACTATTACATGTAAAGAAAACCAAATGACGGATCAGTTCACGAATTTCTCACTTCTTGTCGATCAACAAGTTTCC
GAAGGGGAAAACACAAATATTCAGCCATGTTTCATTCCCAGTGGTTCTACCTTTAGCACCTTCGATCAATTCGATATGAA
CTCATTCTGGTTTGATGTACTTGGAAATTTCTGA

>CcMYB61 |Locus_ID=BC332_19187 |CDS_len=690 |strand=+
ATGGTGAGAACACCAATTATTGACAAGAATGGAATAAAAAGAGGTGCATGGAGTGAAGACGAAGACAACAAATTAAGAGC
TTATGTTGAAAGATTTGGTCATCCCAATTGGCGGCAATTGCCTAAATATGCTGGTCTAATGAGATGTGGGAAGAGCTGCA
GACTGAGATGGATGAATCACTTGAGGCCTGGTTTGAAGAAAGGCAATTATAGACTTGAAGAGGAACAACTCATAATTAAA
TTACACAAGGAACTGGGTAACAGATGGTCAGTGATTGCTGCAAACTTACCAGGAAGATCGGACAATGATGTGAAAAACCA
TTGGCATGCTCATCTCAAGAAACGTGTGAGACTAACAAATTGTATCAATAATTCATCAATGTCTATGGAGCAATTTACTG
AGTCTACGCAGTCTGGATCGTCTCAATTAGATGAACGGTCTGCTAGTATGAATGTTGTTGTTGACACTTCACCAACCTTA
CTGCCAATCTCTTTCCCAGAGGTTTCATCAAGTGATTTATATTCCATAACGGACTGGATCGAAGAAGAGTCAATGGAACA
ACTTCCTATTTTGAGTTCAATGGAACTACTTGCAGCAGACACTTTTTCCTGGACAAATTATCCCATTGACAATTTTCAGA
CAGAATCCTTTGACCATTTTTGGACCCAACCCTTCGATACTTTCTTTTAA

>CcMYB63 |Locus_ID=BC332_19183 |CDS_len=744 |strand=-
ATGGTGAGAACACCTTGCTGTGATGAAAATGGAAGGAAGAAGGGGACATGGACTCCTGAAGAAGATAGGAAATTAGCAGC
ATATATCACTGAATATGGCTCATGGAATTGGCGCCAACTTCCCAAATATGCTGGACTAGAGAGGTGTGGAAAGAGCTGCA
GACTTCGATGGCTGAATTACTTAAGGCCAAATGTTAAAAGAGGGAACTACACCGAAGAAGAAGATGAAATCATCTTAAAC
CTCCATGCTCAACTTGGAAATAAGTGGTCGGCGATTGCAGCTCACTTGCCAGGAAGATCAGACAATGAGATAAAGAATCA
TTGGCACACTGCACTCAAGAAGCGTGCAAATAACTCAGGTGAATCAAGCAAGAAATGCAACAGTAAGAACAACATTAGAA
GGAGTACTAGTGTGGCAAATAAAAATGCAAGTCCTAGCACTTCACATGAAAATATAGTACTGGAAAGTTCACAATGGTCC
CCAAAGGAGTCATCAAGTGAAGACTTTTTTTCTTACAGTAATACTGATTATCAAGAGCAACATAAAGTTTTTCAACAAGA
AGCTTTGGATGAAATAACTAGTGGAAGCTTTTGGACAGAACCATTTCTAGTGGAAAGTTTCAATACAACTGGAACTGATT
TTCTAGCTCCTCCAATTGATTACTGTGGACTTGTGTGTCCACCTTCTCCTTTTATAGGTCATGAATTTCTTTCATCCTTT
GATTTTGATCATTTTAATTATTAA

>CcMYB64 |Locus_ID=BC332_19182 |CDS_len=411 |strand=-
ATGGTGAGAACACCTTGTCATGACGAAAAAGGAAGGAAGAAGGGAACGTGGACACCTGAAGAAGATAGGAAATTAGCAGC
ATATATTACTAAATATGGCTCCTGGAATTGGCGCCAACTTCCTAAGTATGCTGGACTAGCAAGGTGTGGAAAAAGCTGCA
GACTTCGATGGATGAATTACTTAAGGCCAAATGTTAAAAGAGGGAACTACACCAAAGAAGAAGATGAAATTATCTTGAAC
CTTCATGCTCAACTTGGAAATAAGTGGTCGGCGATTGCTGTTCACTTGCCAGGAAGATCAGACAATGAGATAAAAAATCA
TTGGCACACAGCCCTTAAGAAGCGCGCTAATTATGTGTCAACCTCAAGTGATGTGTCAAGCAAGAAATCTAATAAGAAGA
AGAATAGTGGA

>CcMYB65 |Locus_ID=BC332_19186 |CDS_len=747 |strand=-
TTAGCAAGGTGTGGAAAGAGCTGCAGACTTCGATGGATGAATCACTTAAGTCCCAATGTTAAAAGGGGGAACTACACCAA
AGAAGAAGACGAAATCATCTTGAACCTCCATGCTCAACTTGGAAATAAGTGGTCGGCAATTGCGGCTCACTTGCCAGGAA
GATCAGACAATGAGATAAAGAATCATTGGCACACAACACTCAAGAAGCATGCTACCAATAATGCCACAAACTCATCAAGT
GATAAATCAAGCAAGAAATGTAGTAATAATAAGAATAAGAATACCAAAAGTAATGCCAGTAAAGATGCAAGTCCTAATAA
TAATATGCATGAAAATGTTATACTAGAAAGTTCAGAATGGTCGCCAAAAGAGTCATTAAGCGATCAAGAACTAATCTCCT
CTGATTCCACTAGTGTTGATTATCAAAACGACGTTTTTCAAGAAGAAAATTTGGTGGAAATAACTAGCGGAAGCTTTTGG
ACAGAACCATTTGTTGTAGAAAGTTTCAATAGTCCTAGAATTGATTTTCTAGGTCCACCTTCAATAGATTATGGACTCGT
GTGTCCACCTTCTCCTTTTATAGGTGAAGGATTTGTTTCCTCCTTTGATCTAGATGATTATAATTGTGGCAGAATTGTTG
AAGAAGCTGAAGATGGAAAGAAGCAACATACAAAGAAGAAGGAGAAGACAAAAAAGATGAACAGAAAGAAGAAAAAGAAG
AAGCAGACAAAAAAGATGTGGTCTTAG

>CcMYB67-1 |Locus_ID=BC332_06786 |CDS_len=837 |strand=+
ATGGTTAGAGCTCCTTGTTGTGAAAAGATGGGGTTGAAGAAAGGACCATGGACACCTGAAGAAGATCAAATTCTTGTTTC
TTATATTCAAGCAAATGGTCATGGCAATTGGAGAGCCCTTCCTAAACTAGCTGGATTATTGAGATGTGGAAAGAGTTGCA
GACTTCGTTGGACAAATTATTTGCGTCCAGATATAAAGAGAGGAAACTTCACTAGAGAAGAAGAAGATAGCATTATCCAA
TTACATGAAATGCTTGGCAATAGATGGTCTGCAATAGCAGCTAGATTACCGGGTCGAACAGACAACGAGATTAAAAATGT
ATGGCACACTCACTTGAAAAAAAGGCTTAAAAATTATCAGGCTCCTGAAAATTCCAAAAGACACTGCAAAAAGAACATTG
ATTCCAAAGCTCCTAGTACTTCTCAAATTACCTTGAAAAGTTCACACAATTTTAGCAACATCAAAGAAGGCATTAATGGG
CCCGACCCGAACTCGCCACAATTGTCGTCTAGCGAGGCATCGACCGTCACGGCCGATTCTGAGTCGGTCATGACGATGGG
TGTCACAAACGGTGGTGATCAGATGGACTCGTATGAAAATTTTATTCCAGAGATCGATGAGAGTTTCTGGACAGACGATT
TATTATCTACGGCCGATAACTCGAGTTTCGATATGGCAATTAGTGGTGGTGAAGAGTTACAAGGTCAATTTCCTTTTTAT
GACATGAAGCAAGAAAGTGTGGAGTTAGATGTTGGAGCAAAATTAGAGGATGATATGGACTTTTGGTACAATGTTTTCAT
AAAGTCTGGGGACCTATTAGAATTACCTGAATTTTGA

>CcMYB67-2 |Locus_ID=BC332_10106 |CDS_len=333 |strand=+
ATGGTTAGAGCGCCTTGTTGTGATAAGACAGGTTTAAGGAAAGGGCCATGGGCTTTTGAAGAAGACCAAATTCTTATATC
CTACATTCAAAAATATGGTCATGGCAATTGGCGCGCTCTTCCTAAACAAGCCGTCGTGACATCTGATTCATCATCGTCGA
TCATCGCCTTTGGAGTGACGATCGACAATACGATAGCGGTTAAACTGGAAGAATCGGACTCGTCAGAGTATCTTACCGAG
ATCGATGAGAGTTTCCGGACAGACGATGATATGGACTTTTTGTTCAACTTTTTCATAAAGGCTGAGGACTTTTCAGAATT
ACCAGAACTTTGA

>CcMYB68 |Locus_ID=BC332_15553 |CDS_len=843 |strand=-
ATGGGAAGAGCTCCTTGTTGTGAGAAGATGGGGTTGAAGAAAGGGCCATGGAGTCCTCAAGAAGACAACATTCTCATCTC
TTACATTCAAAATAATGGTCATTCTAATTGGCGTGCCCTTCCTAAACTTGCTGGATTATTGAGATGCGGGAAGAGTTGCA
GACTGCGGTGGACTAATTATTTGCGTCCAGATATAAAAAGGGGAAATTTCAGCAAGGAAGAAGAAGACACCATTATCCAG
CTTCATGAAAACCTTGGCAATCGATGGTCTGCAATAGCAGCAAGATTATCGGGACGAACGGATAATGAAATAAAAAATGT
TTGGCACACTCACTTGAAGAAGAAGCTCAAGAATTATCAACCTCCTCAAAATTCCAAAAGACACTTCAAAACTAATCATG
ATTCCAAAAAGGGTCCTACTACTGAATCATCCAATAATTCTGATTTTAGTACTAGTGCTAATAACACCAACACACATAAA
AAACACATGAAAATTGCCCCCAACTCACCAGTACTTTCATCTAGTGAAATGTCAACTGTGACATTAGTAGTCGATGATCG
TCAAAGGGTGATTATTAAGGAAGAAAAAATAGAGTCGCCATTGGAGTATTTTCCTAAGATCGATGAGAGTTTCTGGACAG
AAGAATTGTCAGCGGAGAAAAATAATGATAACGACTACGTTATGGCTGCTGGTCTTGATCAAGATATACAAGCTCAAATT
CAATTGTCCAGTGTGAAGGAAGAAAATTTGGACAATACTAGTACAAAAATGGAGGAGGATATGGACTTTTGGTACAATGT
TTTCATAAAGACTGGGGACTTTCTAGAATTACCGGAATTTTGA

>CcMYB69 |Locus_ID=BC332_17589 |CDS_len=852 |strand=-
ATGGGGAGAACTCCATGCTGTGAGAAGTTAGGATTGAAAAGAGGTCCATGGAGTAAAGAGGAAGATGATTTACTCATAAA
TTACATTAATAAAAATGGTCATCCTAATTGGCGTGCACTTCCCAAACTTGCTGGTCTTTTGAGATGTGGAAAGAGTTGTA
GGCTTCGATGGACTAATTACTTGAAACCTGATATTAAGAGAGGGAATTTTACTAATGAAGAAGAAGACACCATTATCAAG
TTGCACCAAGTTCTTGGAAACAGGTGGTCGACGATTGCAGCAAGATTACCAGGAAGAACAGACAATGAAATAAAAAATAT
TTGGCATACTCGTCTGAAAAAGAAAATAAGTAAATCTCATGCTCAAAAAATTCCGGAGTTACAAGAAGAAACATCCATGG
AAACATCAAAATCTGATGAACAAAATTTGGACACACATTTAGAGCCTGAAAATTCTAATTTGGATATACATTTGGAGTCT
GATAATTCTAAGGATAATTGTGAGATATCGAGTCCTAAAACAAATAACGAGATTCAACAACAATCAAGTTCTTCGGTACA
TTCATCATCATCGATATCATCATCGAGTGAAGATGCATGTTCAAACACAACTGCAACGAGTCCGGAGCCAAGAGATCAAT
TCATGTTGGATAATTTGGTTGAAATTGATGATGATTTTTGGTCCGACGTACTATGGACAACGGTCGATGACTCTAATTAT
AATGACAATAATTTTGATTTGCCATTAATGGAGGATAATTATCAGATTAATCCTAGCTTGAATGATAACTGGTTTTGGGA
TGATCTTTTTACAAGATCCAATGAGTTAATGTTAGAATTGTCAGAATTTTGA

>CcMYB7 |Locus_ID=BC332_17369 |CDS_len=1026 |strand=+
AAACTGCTGAGGTTGGTAAAACAATTTGGAGTAAGAAAATGGGCACAAATTGCTGAGAAAATGGGAGCAAGAGCAGGAAA
ACAGTGTAGAGAAAGGTGGCATAATCATCTACGTCCAGATATTAAGAAAGATACATGGAGTGAAGAAGAAGAGTTGCTGC

TAGTGGAATTACACAAGCTACTTGGAAACAAATGGGCTGAAATTGCTAAAAGAATTCCTGGAAGGACTGAAAATGCAATC
AAGAATCACTGGAATGCCACTAAAAGAAGGCAAAATTCAAGGAGAAATAAGAACAAAAAAACTCAACAAGCTGATGGCAC
TCAATGTGAAAACAAATCGCATCGTTCCACCGTTCTCCAAGATTATATTAGAAGCAAATATTTCATGAATGATGATAATT
CTCCCCTCACCGCTACCCTATATTATGGTAGCGGTGGTACAGCTAGTGGTGGTGGGTACAGTCCCTCCACCACTACTTTC
ATAGGAAGTACTAGTAACGCGACCAACGCTACACCAACATACTCTGACGATGATTCTCCATATTTGATCACTCATCAAAC
ATATGATGAAGAAATGAACTTCATGCAAAGTTTATTTGGAAATAGCAATAATATCAATGGTACTAAGGTCATGGGCACTA
TGGAGACAAATGTAGTGACTCAAGATCTCCTCGACAAGAGCTCGTCGAGCTCTTCTTTTGGTTTGAACTCCTCTGGTGAA
ATCCCTCTAGCTGAGTGTAACGTTGAGAGTTACACTACAACAAAAAGGGTGATCGCGGACAATAATGCTAGGAATTACAT
CCCTGATTATTCTCAATATTATCCTCAAGACCTCTATTTATCGCGCCTTCTAGATGGTTCATTGGTGCCTAATTATTCAG
ACTGTTTTGAATGTGGAAGCATGAATAATCAAGCTGGTTGTTCTTCATCAGGAGGAAACATTAAGGAAGTTGATTTGATG
GAGATGATGAATTCAACTAATTCTCAATTCTCTCAACCTGGAACCTTCAACAAAACTTTCTTTTGA

>CcMYB70 |Locus_ID=BC332_14283 |CDS_len=444 |strand=-
ATGTTCTTTTTCTTCAGATGGTCTGCAATTGCATCGAGGTTGCCAGGAAGAACTGATAATGAAATCAAAAATTTCTGGCA
CACTCACTTGAAGAAAAGATTAGAACAACACAATATTGATCTAGCATCTACTACAACTACCAAACAAAACCATGAAATGA
CAACAAATGTTACACAAAATATTGACCACCATATAATATCTCCAAATTATTATCAAGATTCTCAAAATATGGTTACTTAT
CATCCAACTTATTGTCATGATCATCAAGAAGATTTTCAAGAAAATAATTCAAGTTCTCATGATCATACTCAATCAAACAA
GGAGGAGATCATGCAATATTCAACAAATTATGGACATGAAGACATGACTAGTTTCAATAATGATATGGTTTTTTGGTATA
ATGTCTTCATGAGTTCAGGAAATAACCCGTCCGAGATCAGTTAG

>CcMYB71 |Locus_ID=BC332_24933 |CDS_len=678 |strand=+
ATGGGTAAAGGAAGAAGAAGTTGTTGTGATAAAAGTGAAGTAAAGAAAGGGCCATGGACTCCTGAAGAAGATTTGAAGCT
TATTTCTTTCATTCAGAAACACGGTCATGGCAATTGGCGCGCCCTCCCTAAACAAGCCGGGCTAGTGAGGTGTGGGAAGA
GTTGTCGATTGAGGTGGATTAACTACCTGAGACCTGATGTAAAGCGAGGGAATTTCACCTCACAAGAAGAGGATACTATT
ATAAACCTACACCGCTCTTTTGGGAATAGGTGGTCAAAGATAGCATCTCATTTACCAGGAAGAACGGATAATGAGATTAA
AAATGTATGGAATACGCATTTGAAGAAGAGATTATTACTCATGAAGAAAGAGGAACACAATTCTCCTTCATCAACACCAA
CATGTAGTAGCACTACTTTAGGGTTTTGCTCTCCAACTTCTTCGAAGGCCAATGATCAAGTGATCGACTTTTGGGAATAT
ATGTTGGATACTAGTAATCCTACTAGTATTAATAATTTGGATCATTTCGACAGCTATTCCAAACAGGACACTACTAGTAC
TAATACCGAACATCATCAACCTCAGATGGCCGACGAATATGAATGCCAAAAGTGGTTGACATACTTGGAGATTGAACTTG
GACTAACAACAAATGAAGCACAACACCAAATAAAATAA

>CcMYB72 |Locus_ID=BC332_20160 |CDS_len=888 |strand=-
ATGATGGGAAAAGGGAGAGCACCATGCTGTGATAAGAGTAAGGTGAAGAAGGGTCCATGGAGTAGTGAAGAGGATATCAG
ACTCATTGCTTTTATTCAGAAACATGGTCATCAAAACTGGCGATCCCTCCCTAAACAAGCTGGGCTGCTGAGGTGTGGGA
AAAGTTGTCGTTTGAGGTGGATTAATTACCTAAGACCTGATGTAAAACGAGGAAATTTCACCCCTCAAGAAGAGGAAACT
ATCATCAAATTGCACCAATCTTTTGGAAATAAGTGGTCAAGAATTGCGTCTCATTTGCCTGGAAGAACGGATAACGAGAT
TAAAAATGTTTGGAACACACACTTGAAGAAACGACTCATGAAAAGGTTGCATGGTGATAAAGCAAAATTAGACGGTACAT
GTGCTAATTCACAATCTTCCTCTTCATCAACTTCAGTAGTATCACATACTGATGATACTTCCAATGAGGGAAAATTCAAG
AACGTAGACGCCAATATGATAATTGAGAATGCAACCAAAGAAGAAGAAGCAACAAAATCCTGCTCTCCTACTTCTTCCTA
CGCCTCAAATTTGAGCCAAGTTCATGAGGCTTCAAGTCCAAATAATGTAGACATGGATTGGATATTCCAATTCAGTGAGG
AGCCTTTTCCAAGTACTACAGCGAGAAAGGTGCATGATGACGACGACGAAGGGATAATTGAAATCCCATTGGAATGTGAT
GTTGATTTTTGGGACATGTTAGATATTATGGGCCCTTCTCCTCCTCCTCCTCCTCCTCCTAATTCAGAATCCAAATTGGA
CCATCACTCTGAAGCGTCTTGTCAAGGCTTGTCAGAATTCGAACGTCAAAAGTGGTTCACATACTTTGAACGTGAATTGG
GTGTTTGA

>CcMYB73 |Locus_ID=BC332_21478 |CDS_len=933 |strand=+
ATGGGGAGAACACCATGTTGTGATAAGAATGGTCTGACTAGAGGACCTTGGACACCTGAAGAAGATGAGAAGCTTGTTGA
GTTTATCAACAAGAATGGTCACGGTAGCTGGCGTTCACTCCCTAAACTTGCAGGTCTTCTTCGTTGTGGGAAAAGTTGTC
GTCTTAGATGGACGAATTATCTAAGACCAGACATCAAACGAGGTCCTTTTAGCCCCGAAGAACAGAAGCTTGTCATACAG
TTACATGGCATTCTTGGAAACAGATGGGCTGCAATTGCTTCACAATTACCTGGGAGGACAGACAATGAGATAAAGAATCT
ATGGAATACTCACCTGAGAAAGCGTCTGCTTTCCATGGGCATAGATCCTCAGACTCATGAACACCACTCTGACCCCAATG
GACTGGTGAGAAGACCGGCTACATCATCTTTAGCTCGTCACTTGGCACAATGGGAAAGTGCTAGGCTCGAAGCTGAGGCT
CGTCTGTCAAGAGAGTCTCAATTTTTTGGTTCCGTCATCAGTTGGGAGGTCTGAGACCGACTATTTTTTGCGCATATGGAA
CTCAGAGATAGGAGAGTCATTTCGTAAATTCAAGAAAGGAGAAAAAAGCGCTTGTCACAGTCCAGCCTCTCAAGCGTCTT
CGTGTACAAAGTATGGATCAGCTTCAGGCATTACGACTGAGGTTGAGATCGGTGTTGCTGGTTCACCAGTGACAGGGAGC
AACCAAAATGAATGTATGGAGTGGAAGATTGGCCAACCATATACTGAAGATTTCTTGCAAGGAGCAGATACCTGTAGCTC
CAATGCCATGGATGAGGATTCATCCGAATCAGCATTGCAACTTCTTTTGGATTTCCCTAGTAACAATGACATGAGCTTTC
TAGGACATAGTGATTCCTATAGCTTGTACCCCTTTTTGAGTGAAAGCTATTGA

>CcMYB74 |Locus_ID=BC332_00519 |CDS_len=834 |strand=-
ATGGGAAGGCAACCTTGTTGTGACAAACTAGGTGTGAAGAAAGGTCCATGGACAACAGAGGAAGACAAGAAACTCATCAA
TTTTATTCTTACAAATGGCCAATGTTGTTGGCGTGCTGTCCCTAAACTTGCCGGTCTTCGGCGTTGTGGTAAGAGTTGTC

GTCTTCGATGGACTAATTACCTTCGACCCGACTTGAAACGCGGACTTCTTAGTGATGCTGAGGAGAAAATGGTCATTGAT
CTTCATGCTCGTCTTGGAAACAGGTGGTCCAAGATTGCTGCAAGATTACCGGGAAGGACCGATAATGAGATAAAAAATCA
TTGGAACACACATATTAAGAAAAAGCTTCTCAAAATGGGGATTGATCCTGTTACACACGAACCACTAATCAAAGAGGAAA
ATCCAACTGATCAACCTATCCAAGAATCTGATCAAAATAAAAATAATGATCATCAGCAGGTAGTGGTTGTAACAGCAGGA
GCCACTTCTTCAGAATTAGATAACTCTTCTACCTCATCTTCTTCTGCTTCTTCGTCTGAAAACTCTTGTAACGAATCCAA
ACTTGTTCTTGACACGTTTAAAGAAAATGATCCGCTGCTAAGCTGTCTACTTGAGGCCGATGATGCTCCTCCTCTCATCG
ATTCGTCCTGGGAACTTCCAGTAAAAAGTTTCGATAACAACAACAACATGACATCGTCGTGGACGACGAAAGTTTCAAT
TGGCTATTTGATTGTCAAGATTTCGGGATCCATGACTTTGGTTTTGATAATTGCTTCAATGATGTCGAATTGGAGGTCTT
TAACACCATATATGACATGGAAAACAAGAAGTGA

>CcMYB75 |Locus_ID=BC332_12501 |CDS_len=960 |strand=-
ATGGGAAGACAACCTTGTTGTGACAAAAATGGTGTGAAAAAAGGTCCATGGTCAAGTGAAGAAGACAAGAAACTCATTAA
TTTCATCCTTAACAATGGCCAATGTTGTTGGAGATCTCTCCCAAAACTTGCAGGTTTATTGAGATGTGGAAAGAGTTGTA
GATTGAGATGGACAAATTATCTAAGACCAGATGTAAAAAGGGGACTTTTATCAGAATATGAAGAAAAAATGGTTATCGAT
CTTCATGCTCAACTTGGCAATAGATGGTCTAAAATCGCGTCTCATCTGCCGGGAAGAACTGATAATGAAATCAAGAATCA
CTGGAATACACATATTAAGAAGAAGCTCAAGAAAATGGGGATTGATCCAATCACTCACAAGCCAATTACTAGTGACCAAC
CAACATATGATCAACCAATCATTCAACAAGAAAAACAAACATTAATTGCGCCTCCGTCTCAAACAGATTTGAATCGTCAA
GGGAATGAGAACACTGGGACATGTCCAAAAAATATCAAGACGACAACAACAGCAACAACAACAACAGTAGCTATGCCTTC
GTCTGAGACAAATTTGGATCGTCAAGAAAACAAGAACGTTGGAACATGTCCAAGACATATCAAGACGACGACAACAACAA
CAACAGTAGTTACGCCTCAGTCTGAGACAAATTTGGATCGTCAAAAAGTCCCTTTAATTCCACCAACTGAAATTTTAATC
CCTTATGAATCAACAACTTCATCAACATCTTCATTTTCCTCACAATCATCCAATATGATCCTTGAAGACTTGAAATTTTT
GCCAAATTTTGATAATTGGCAAAGTGATTTCAACATGGAAATTAATTGGACAAATGATGATTTTAGTAGCACTTTGGATT
ATTTACTCAATGATGATGATATTAGTGACATGAACAACACCACCATTTCCCAAGATTGGTCACAAGTGTTGGAAGTTTGA

>CcMYB76 |Locus_ID=BC332_29469 |CDS_len=912 |strand=+
ATGGGGAGACAACCTTGTTGTGACAAAGTTGGATTGAAGAAAGGTCCATGGACAACTGAGGAAGACAAGAAACTCATTCA
CTTCATTCTTAACAATGGCCAATGTTGTTGGAGAGCTGTCCCTAAACTTGCAGGGCTATTAAGGTGTGGAAAGAGTTGTA
GATTAAGATGGACAAATTATTTAAGACCAGATTTAAAGAGAGGACTTTTATCAGAATATGAAGAAAAAATGGTTATTGAT
CTTCATGCTCAACTTGGAAATAGGTGGTCAAAGATTGCTTCTCATTTGCCTGGAAGAACCGATAATGAAATCAAGAATCA
CTGGAACACGCATATCAAGAAGAAGTTGAAGAAAATGGGGATTGATCCAGTCACTCACAAGTCACTTTCCACTATTACAA
ATGATCAAACAAACATATTAGAACAAGAAAATCAACCAATTCAACAAGAAAAAGTTGAAGATCAATCAACTATGATAGAG
ATCAAAGCAGAAGACGACAACAACAACAGCAACAACAACAACAGTAATAATGATAATAACACAATGGAGACGAGTTGCGC
GAATAATTTTAACTCAACTATAGTGGAAGTCAACAATAATGGCTTTGGTATAGATGAAGTTCCATTGATTGAACCTCATG
AGATGTTAGTTCAAGAATTATCATCAACGCCATCAACATCATCATCATCATCATCATTTTCATCATCATCGTCATCCAAC
ATTCTTGAAGATTTGAAGTTTTTGCCAAGCTTTGATGAATGGCCATTAATGGAAAATAACAACATGGGATTTGGATGGGA
AATTAACAATGATTTCACTAGCACATTGGATTTCTTGCTTGAGGATGATCATAGTGACATGATGAATAATGTCACATTTG
ATGAATCTTGGAAGTTTGAGCAACTCTTGTGA

>CcMYB77 |Locus_ID=BC332_06135 |CDS_len=768 |strand=-
ATGGGAAGGCAGCCTTGTTGTGATAGAGTTGGATTGAAGAGAGGTCCATGGACTATTGAAGAAGACCACAAGCTAGTTCA
TTTTATTCTCAGTAATGGCATTCAGTGTTGGCGAACCGTTCCCAAGCTTGCAGGGTTGCAAAGATGCGGCAAAAGTTGTA
GATTGAGATGGATTAATTATCTGAGACCTGACCTCAAAAGAGGGGCGCTATCTGAAGCTGAAGAGGATCAGATTATACAA
CTCCATGCTCGTCTTGGTAACAGGTGGTCTAAGATAGCATCATACTTTCCGGGGAGGACAGATAACGAGATCAAGAACCA
TTGGAACACTCGGATTAAGAAGAGATTACAGCTTATGGGAATAGACCCTTTAACTCACCAGCCAATTGACATGGAAAAAT
ATCAACATGAACCTGATCAAACAGAGAAATGTGATAAAAGAAACGAACCAGAGAACTTCATTGAAAATACTAATGATGTT
TCACTACCAAACAATCCACACCACAATATGTTGAGTGACAGTTTTAATGTGGACTTATGGAACAAAAGTGGTAACACTAT
GTCCACTTGTTATAGCCCTTCAATTTCTTTGGAGGCTGAAGAATCTATCAACTTGTCAACAACAACATCAGCTGCTACTG
AATCATCAAGTAATGTAGCTGCACTCACAGAAGAACAAGATTCTCTACAGCAATGGATGGATTCAATTTTCTCATGTGCG
GTCAATCAACTAGAAGAAGACCTCATCTTTTTGAGGAAATACAATTAA

>CcMYB78 |Locus_ID=BC332_28218 |CDS_len=675 |strand=+
ATGGGAAGACAACCTTGTTGTGACAAGATAGGATTGAAAAGAGGTCCTTGGAAAATTGAAGAAGATCATAAGCTTATGAA
CTTCATTCTCAACAATGGTATACAATGTTGGCGTCTTGTACCAAAATTAGCAGGGCTAATGAGATGTGGGAAGAGCTGTA
GATTAAGATGGATAAATTATCTGAGGCCAGACCTCAAAAGAGGAGCACTCACTGAAGCAGAGGAAGATATAATCATTAAG
CTTCATTCTCATCTTGGCAATAGGTGGTCGAAAATAGCAGCGCATTTTCCAGGACGTACAGACAATGAGATCAAGAATCA
TTGGAATACAAGGATTAAAAAGAAGCTAAAATTTCTTGGCATAGACCCTCTAACTCACAAGCCCATTGAACAGAATGATG
ATATTAAGCAACAAGTTTCTGTAGAGATTGATGAGAATGTGAAGGAACTAGCAGAAAAAACCTTAGATTATCCACGAGAG
CAGCAAAATATTCAAAATTCAACATCTAGTTCCAGCTTAAATGAGAGTTTGGATATGGGGTCATCAAGTGAAATATTCCA
AAATTCACACACATTGGACATGTATAATCCAGGAGTTGAAGATCCTTTCCAGAATTGGATAGGTAGTCCAATACATTGGG
ATCTTTTCGACAACTTAGAAGACAATTTTCTATGA

>CcMYB79 |Locus_ID=BC332_10962 |CDS_len=1044 |strand=-

ATGGGGAGGTCACCTTGTTGTGATGAAAATGGGCTGAAAAAAGGACCATGGACTCCTGAAGAAGATCAAAAACTTGTTGA
TTATATTGACAAACATGGTCATGGCAGTTGGAGAGCCCTTCCTAAGCTTTCAGGACTTAATAGGTGTGGCAAGAGCTGCA
GGTTAAGATGGACTAATTATTTGAGGCCTGATATCAAGAGAGGCAAATTCTCTGAAGAAGAAGAACAAACAATTCTTCAT
CTCCATTCCATCCTAGGAAACAAATGGTCCGCAATCGCGACACACTTACCGGGAAGAACAGACAACGAGATAAAGAATTT
TTGGAACACACATCTAAAGAAGAAGCTAATTCAAATGGGATATGATCCAATGACTCACAGGCCAAGAACTGATTTATTTG
CAAGCTTGCCTAATATAATAGCTTTGGCAAATTTACTTCAGCATCATCCACTTGAAGATCAAGCTATAAGATTACAAGCA
GAAGCTGCCCAAATAGCTAAGATTCAGTACTTACAATTTCTATTACAATCTTCAAATAATAATTATAATCCAATACCACC
TACTACCTCATCAAACACTCAATATTACAATAATCTTGGAGATATTGGGGCATTTAATTTGACAAATTTTGTTAAAGAAA
ATAGTACTACCCCTTCTTTAAATTTATCCAATCTTGAAAATCAAACACTTTTCTCCAATGAAACTGGCTCTCAACTACTC
CACAATCCAGATAACACACAACAAGTCCCTTTCAATTTCCAAACACATTTGAATAATAACAATAATAATGATATGAGTGA
TATTGATGGTAACAATTTTAATTTTGATCTAGAAGATAATTCACCAAGTTCCCCATTGAATAATACTATTATTCCCTCTC
CACCTTCTTCAAGCCATTTGCCACATTTGACTGAAATTTCCATCAGCAATAATAATCAAGGAGATTCCAGTTGCAATTCC
TCTAACAATGTTGCTGATCAAGGGACTTCTTCTTCATATTGGCCAGAGCTGTTTTTTGAAGAACACTTCATGCATGACAT
TTGA

>CcMYB8 |Locus_ID=BC332_08524 |CDS_len=747 |strand=-
ATGTGTAGCAGAGGGCATTGGAGGCCTCATGAAGATGAGAAACTGAAAGAGTTAGTTGCTAAATATGGACCTCATAACTG
GAACGCTATTGCAGAAAACTTGCAAGGCAGATCAGGGAAGAGTTGTAGGTTGAGATGGTACAATCAATTGGATCCAAGAA
TCAACAGAAGCCCATTCACAGAAGAGGAAGAAGAAAGACTTCTTTCCTCACATAGAATTCATGGAAATCGTTGGGCTATG
ATAGCTAGACTATTTCCAGGTCGTACTGATAATGCTGTGAAAAATCATTGGCATGTTATTATGTCCAGAAGATGCAGAGA
AAGATCTAAGATTTATTCCAATAGAGCTTCTGCTGCTGCTGCTGCTCAAAAATCAACAGACCAACAAGACAATATTAATA
ATATCCAAGATATGATGATCAGAAAAAGAAGTTCCAACAATTTTATTGACCATCAACAGTTATTAGAAAGATATAGTAAT
TGTTACCAATTCAGTAGCTATGATCACAATTCCTTTTATCCAACAGAGTTTCATTTCAACCACCTACCTCGGCATTTCAA
GATGGATAATCAAGAGAAGAATGAAGAAGTGGAATGCTATGATTTTTTACAAGTGAATAATACTGCAGGCTCAAACAAAA
GTGAAGTGATAGATCAAGAAGTTGAACAGAAACGTGATATTTCTCCTCAAAGTAAAAAAGCTCCAGTGAGATTTATAGAT
TTCCTATCAGTTGGAGACTCGTCCTAA

>CcMYB80 |Locus_ID=BC332_13875 |CDS_len=1056 |strand=+
ATGGGAAGGTCTCCTTGTTGTGATGAGAGTGGTCTAAAGAAAGGTCCATGGACTCCTGAAGAAGATCAAAAACTCACCAA
TTATATCAATAAAAATGGACATGGCAGCTGGAGGGCCCTCCCTAAACATGCAGGTCTTAATAGATGTGGAAAGAGTTGCA
GATTGAGATGGAGTAATTATCTTAGGCCAGATATTAAAAGAGGCAAATTCTCTCAAGAAGAGGAACAAACAATTCTCAAT
CTCCATGCTGTCCTTGGTAACAAGTGGTCAGCAATTGCAACTCATCTACCAGGGAGAACTGACAATGAAATCAAGAATTT
TTGGAACACTCATTTGAAGAAGAAGTTGATCCAAATGGGTTATGATCCAATGACTCATAGGCCTAGAACTGATATCTTTA
ATAGCTTGCAACATCTCATAGCTTTAGCAAATTTGAAGGAATTAATTGAACATAATCACTCATGGGAAGAACAAGTTATG
AGACTTCATTACTTGCAAAATCTTCTACAACAACCTCACAACAATAATATTAGTACCTTAAGTGCTATCCAAAATGTGGA
AGCTAATTACAATCTTTTAAATTCTTTAAAAGATAGCCAATTCTTAGACTCAAATAGTCATTTGGGAAATCACATAGTTC
ATCAAATTCCTTCAAGTCTTGATCAACCTATTCAAAATTCAATCCCTTTCTCCCATTTGACTGAATTACACACCCCTTGT
AGTTTCCAAACTTCTTTGAACAAAGATAAGGTTCCACCTCAAGACATTGAGTTTACGGTTATGAGCCACGAAGAAACTTC
GCCTGCTTCTTCGTGGCTCCCTCCTTTATCTCCTTCTCTTCCTCCTCCTCCTCCACCGGTATCAAACAATCAAAGAAGAA
AAGAAAACTCTTCAGAAGTGGTGATCTCATCTTGCTTGCCAGAAGATATGAAGGTTTTGGATAACTTTTTTCAACAAGAT
AGCGAACAATCACTCAGCAATGAAGGAGCTCCACCTTCTATTTGGTCTGACCTTCCACTTGAAGACTCTTTTTTCCAAGG
TCTTGATAAATTCTAA

>CcMYB81 |Locus_ID=BC332_00728 |CDS_len=900 |strand=-
ATGGGGAGATATCCATGTTGCAAATTGGAGAATGATTTGAAGAAGGGGCCATGGACAAGTGAAGAAGATGAAAAATTGGT
GGAATATATTCAAGAAAATGGACATGGAAATTGGCAATTAGTCCCTAAAAGAGCAGGATTAAATAGGTGTGGGAAAAGTT
GTAGATTAAGGTGGACTAATTATTTAAGGCCAGATATTAAAAGAGGAGCATTCTCTAATGAAGAAGAAGAAACAATTATC
AACCTTCATTCTCTTCTTGGAAACAAGTGGTCAAGAATTGCAGCTCGTCTTCCGGGAAGAACTGATAACGAAATCAAGAA
TTTGTGGAATACTCATCTCAAGAAAAAGCTTCTTAAATCTGGAATTGATCCAGTAACACATCAACCAATAACTGATCCAA
ATTTACTTCTTAGCCTTTCTAATTTGATGAACCCTTTTGAATCTGCTCTTAGGTTACAAGCAGAATTTACTGAAATAGCC
AAAATTCATCTTATTCAAAACATAATTCAAGTCCTAAATACTCCCCTAGCTCCATTAATTCCATCTTTACAAGAAAATTA
TCCCATGCAACAATTTTACAATAATATTCTTGCACCATTTGACCAAATTCTTGCTAATGAACCCTCTCCAATTTCGAAGA
ATTTATCGGACAATATGGTCGTAAATTCGAGTATTTTGGAGAATAATATGATTAATAATGGATATAGTAAAGTGTGTGAG
GTTGATAATTCAGAATATTCATCACTTCCATCACTAGTTACAATTACACCTGAAAATTCAGTACCCTTTGATCAGAAGAT
TGATATAATTAATCCAAGTTATGATGATTTTGAAGCATTGGAAAAAAGTCTGGATGATGACTTCTGGAAAGATATTAGTA
GTACAATGCAATTTGTGTAG

>CcMYB82 |Locus_ID=BC332_00729 |CDS_len=645 |strand=-
ATGGGGAGGTATCCATGTTTTAAAGTTGATGAAGATTTAAAGAAAGGGCCATGGACACCAGATGAAGATAAGAAATTGAT
GGATTATATTACAAACAATGGACATACAAATTGGCAATTAATTCCAAAAAAAGCAGGTTTAAACAGATGTGGAAAGAGTT
GTAGATTAAGGTGGACTAATTATCTTAGGCCTGATATTAAAAGAGGAGCATTCTCCTTTGAAGAAGAAGAAATCATTATC
AGCCTACATTCTATTCTTGGAAACAAGTGGTCAAGAATTGCATCTCATCTTCCGGGAAGAACTGATAATGAGATCAAAAA

CTTCTATAACACTCATTTAAGGAAAAAATTACTCAGATTGGGAATTGACCCCAGGACGCATAAGCCAATTTCTGACCTCA
ATTTCCTCATAAATCTTTCTCATCAATTTCCTTCTAAACATAATCTTTTTATAAATAATCCCTTAGCATATGCTCTTAAA
TTACAAGCACAAGTCACAGAAATAGCCAAACTTCAACAACTCTTACAAGACCTTTATGCACCCATAAATACAAATATTCC
ATTATTGCCATCTAATTTTCAAGAAAATAATTTTGCTACAAGTTCAAGCCATGTTATAACAACAAATGTTAGTACCAACA
CCCAC

>CcMYB83 |Locus_ID=BC332_19190 |CDS_len=1014 |strand=+
ATGGGAAGATCACCATGTTGTGATAAAAATGGACTCAAGAAAGGTCCATGGACACCAGAAGAAGATAATAAACTCATCCA
GTATATCCAGCTTCATGGTCCTGGAAATTGGAGAACCCTCCCTAAGAATGCTGGACTTCAACGGTGTGGAAAGAGTTGTC
GTCTTCGTTGGACGAATTACTTGAGACCGGATATTAAGAGAGGAAGATTCTCATTTGATGAAGAAGAAACTATTATCCAA
CTTCATAGTGTTCTTGGAAACAAGTGGTCGGCTATAGCGGCTCGTCTTCCGGGAAGAACAGACAACGAAATCAAGAATTA
TTGGAACACTCACATTAGAAAAAGGCTTCTAAGAAATGGTATTGATCCAGTGACCCATAGTCCTCGTCTTGATTTATTAG
ACTTATCATCCCTCCTAAATTCGACTCAATTCAACCTTTCGAGCTTACTTGGACTACAAGCACTTATAAACCCTGAAGTC
TTTAGGCTTGCAACTACTCTGTTGGCATCACACAATGAAAGTTCCCCAGAATTGTTACTACAAAAACTTCAAGAAAATAA
CCAAATGTTTAGCAACTCTCAGCTACAAAACCAAGAAGGGTCTGAATTATTGTTACAGAAACTTCAACAAAATCAATTAT
TGAATGCCCAAGTGCAAAATCAACCTCAATTTTTCCAATACCAAAATCAGATTCAAGAAATACCAAATTTCACCACCACA
CAAAATGTTGCTTGTTCATCTTCATTATCACAGCCCATGCAATTATTAGGACACGTGGACGAATCCTATTTGATGAATGG
CCAAATGCTCCCTCCACAAAATTATGGTTATTGTAATTCTGATGCTTCTGATAATTCAACTTTCCAATCACTCAACAACA
ACAGCAGCAATCAGAACTTCAGCCTAGATAATTCAGTGTTGTCGACGCCCACTGAAGATGAAAAGAGAGCTATTGCAGT
AATTTGATGAAATTCGAAATTCCAGAGAGTTTAAATTTCGATGATTTTATGTAA

>CcMYB84 |Locus_ID=BC332_25152 |CDS_len=990 |strand=+
ATGGGAAGAACACCATGTTGTGATAAAAATGGACTAAAGAAAGGTCCATGGACTCCAGAGGAAGATCAAAAACTCATTCA
ATACATTCAAGTTCATGGCCCTGGAAATTGGAGAAATCTTCCCAAGAATGCTGGACTTCAAAGGTGTGGCAAGAGTTGTC
GTCTTAGGTGGACAAATTATTTGAGGCCTGATATTAGAAGAGGAAGATTCTCTTTTGAAGAAGAAGAAACTATTATCCAA
CTTCATAGTGTTCTTGGCAACAAGTGGTCAGCTATAGCAGAACGTTTACCAGGAAGAACGGACAACGAAATCAAGAATTA
TTGGAACACACATATAAGAAAAAGATTGTTGAGAATGGGCATTGATCCGGTGACTCATAGCCCTCGTCTTGATTTCTTAG
ACTTTTCATTACTTTTCAACTCGACTCAGCTTAATCTTTCAAGCTTGTTGGGACTACAAGCACTCGTAAACCCTGAAATC
TTCAAACTTGCAAATTCCATATTACTTTCCCAAACTCATGAAAATCAAGAACTATCATTACAAAATCAAAACTCTCAAGG
GCCTGAACTCTTGCTACAAAAACTTCAACAAAATCAATTATTGCATAATCAGTCCTCTATTTTCCAACATAATGATCAAT
TGCACAACCAAATTTCAAAAATACCAAATTACACCCCACAAAATGTTTCATCTTCATCTTCCCAGTCCATACAAGGTAAC
ATGGGGTCTTATTTGATGAATGGCCAAGCATTCCATGAAAATGTGATATCTCTTCAAAACTATAGTTATTCTGCGTCTGA
AGTATCTGAGAACTCAACGATTCAATCCTTAAACAACAATAACAACAACAACAATCAGAATAGTCAGAATTTCAATTTTG
ATACACCTTTGTCAGGCACGGAAGAAGAGAAAGAAAGTTATTGCAGTAACTTCAATAGTTTCATGAAGTTTGAAATTCCA
GAGAGTTTAGATTTTGATGATTTGTTGTAA

>CcMYB85 |Locus_ID=BC332_04760 |CDS_len=1146 |strand=+
ATGGGCAGAGCTCCTTGTTGTGACAAAAATGGACTTAAGAAAGGTCCATGGACACCAGAAGAAGATCAAAAGCTCATTGA
TTACATTCAAAAACATGGTTATGGTAATTGGAGGACTCTTCCTAAGAATGCTGGGCTTCAAAGGTGTGGAAAGAGTTGCA
GGCTTCGTTGGACCAACTATCTAAGGCCAGATATTAAAAGGGGGAGGTTCTCTTTTGAAGAAGAAGAGACTATCATTCAA
CTCCATAGTATTTTAGGAAACAAGTGGTCTGCTATTGCTGCACGGTTGCCCGGTAGAACTGATAATGAAATTAAGAACTA
CTGGAATACGCATATAAGAAAAAGGCTTTTGAGAATGGGAATTGATCCAGTGACTCACAGTCCACGCCTTGATCTTCTTG
ATCTTTCTTCCATTTTAAACCCTTCATTTTACAATAACTCATCATCTCTTCATCAAATGAATCTTTCAAGATTGTTAGGC
CATGTGCAACCTTTGGTAAATCCTGAAGTCTTGAGATTAGCTACTTCTCTTTTATCATCCCAACGCCAAAACACAAATAA
CAGCTTCATGATCCCAAATAATCATCAAGAAAATCAAATTTGCCAAAACCAATTGCCACAAATGGTCCAAAATAGCCAAA
TTCAAGCCCCTATTCAAGATTATCCAACTTGCACAACTTTAAGTACAACCCCATGTGTTCCATTTTCTAGTCATGAAGCT
CAACTCATGCAGCCACCAACAACAAATATGGAGGGCTTCTCATCAAAACTTGAAAATTTCAGTAACTCAACAAGTTGCCA
AGTTAATGATGAGTGGCAATTAAGCAATGGGATGCCTACTTCAAATTTAACGGATGATTATTTTCCTTTACAAAATTATG
GTTACTATGATCCTCTACCGTCCGATGGCTCAACATTAATTCAATCCAACGACAGCAACAACAACAACAACTTCAGCTTC
CAATCAACTGTTTTGTCGAATTTATCGACACCTTCATCGAGTCCTACACCATTGGATTCAAACTCTACTTACTTCAACAA
TAGCAGCAGCACAACTACTGAAGATGAAAGAGATAGCTACTGCAGCAACATGTTGAACTTTGATAATATTCCAAATATTT
GGGATACTACTAATGAATTTATGTAA

>CcMYB86 |Locus_ID=BC332_25248 |CDS_len=966 |strand=+
ATGGGAAGAACACCTTGTTGTGACAAAAATGGACTTAAGAAAGGGCCATGGACCACAGAAGAAGATCAAAGGCTCATTGA
TTACATACAAAAACATGGCTCTGGAAATTGGAGGACTCTTCCAAAGAATGCTGGTCTTCAAAGGTGTGGAAAAAGTTGCA
GGCTACGTTGGACTAACTATCTAAGGCCAGATATTAAAAGAGGAAAATTCTCTTTTGAGGAAGAGGAAACAATCATCCAC
TTGCATAGTATTCTTGGAAACAAGTGGTCTGCCATTGCTGGTCGTTTGCCTGGAAGGACTGATAATGAAATCAAGAACTA
TTGGAATACTCGCATTCGGAAAAAACTTATGAGGATGGGAATTGATCCAGTGACTCATAGTCCTCGTCTTGATCTTCTTG
ATTTAAACTCAATTTTCAACCCTTCAATCTACAATTCATGTCAAGTGATCAATAATAATCTTTCAAGATTATTAGGTGTA
CAATCCCTAGTAAATCCTGAGCTTTTGAAATTAGCCAATTCGCTCTTATCTTCCAACCACCAAAACCAAAATTTCTTGTT
GGGAAGTAATTTTCAAGAAAATCAACCATTCAATTCCCATGTCCAAAACCAATTGACCTCATTATGCCAAGCTAACCAAA

TTCAAAACCCTATTCAAAATACTTCTACCTGCTCCGATTTAAATATTCAATCTGTTCCATTTTATAGTGACACTCAGGTC
ATGCAACAACCAAATGTGGAGCAATTATCCTCAAATGTTTCAACCTTTAGCTCGCAAAATAGCCAACAAAATGAGTGGAA
AAATTTCACCTTCGATTCTGCTTTGCCAAATTTATCAACACCCTCATCAAGTCCTACATCATTGAATTCGAATTCCAGCA
CAACTTCTGAAGAAGAGAGAGAAAGTTATTGTAGTATGTTGAACTTTGATATTCCAAATATTTTGGATGTTAATGAACTT
TTGTAA

>CcMYB87 |Locus_ID=BC332_06008 |CDS_len=1248 |strand=-
ATGGGTCGATCTCCGTGCTGTGATAAAGTTGGGCTGAAAAAAGGACCTTGGACACCTGAAGAAGATCAAAAACTCTTAGC
TTATATTGAAGAACATGGTCATGGTAGCTGGCGTGCATTACCTGCCAAAGCTGGACTTCAAAGATGTGGAAAGAGTTGTA
GGCTCAGGTGGACTAATTACCTAAGGCCTGATATCAAGAGAGGAAAATTTACTTTACAAGAAGAACAAACCATCATTCAA
CTCCATGCTCTCTTAGGGAATAGGTGGTCGGCCATAGCCACTCATTTACCCAAACGAACAGATAACGAGATCAAGAATTA
TTGGAATACGCATCTTAAGAAGCGGCTAGTGAAAATGGGCATTGACCCGGTGACCCACAAGCCCAAAAACGATGCACTGT
TGTCCCATGACGGTCAGTCCAAGAACGCGGCTAACCTTAGCCACATGGCTCAGTGGGAAAGTGCTCGGCTCGAAGCTGAA
GCTAGACTCGCTAGACAATCGAAGCTCAGGTCCAGTAGTTTCCATAGTTCAATCACTTCTCAAGAATTTACTGCTCCTTC
ACCTTCTAGTCCTCTTAGTAAACCGGTCGTGGGCCCAACACGTTGTCTAGACGTGCTGAAAGCCTGGAACGGTGTTTGGA
CCAAACCAATGAATGATGTTTCTGTCGCGAATGCTAGTGCTGGTATTTCAGTCACTGGCCTCGCAAGGGACTTGGAATCC
CCTACTTCTACACTAGGCTATTTCGAAAATGCTCAACATATTTCCACATCAGGAATTGGAGGAAATTCAACTATTCTGTA
TGAATTTGTTGGCAATTCATCAGGGTCTAGTGAAGGTGGAATTATGAACAATGAAGAAAGTGAAGAAGATTGGAAGGGAT
TTGGAAACTCATCAACTGGACATTTGCCTGAATACAACAAAGATGGGATTAATGAAAATTCACTTTCATTCACATCAGGA
CTACAAGATTTGACCCTACCAATGGACACAACATGGACAGCAGAGTCTCTAAGGTCAAATACAGATCAAATTTCCCCTGC
CAATTTTGTTGAGACATTTACTGATCTCTTGCTTAGCAATTCAGGCGATGGTGATTTGTCCGAAGGCGGTGGCACGGAGT
CCGATAACGGAGGGGAAGGTAGTGGCAGTGGAAATGCTAGTGAGAACTGTGAAGATAACAAGAATTACTGGAATAGTATT
TTTAACTTAGTCAATAATCCTTCCCCATCTGATTCAGCTATGTTCTGA

>CcMYB89 |Locus_ID=BC332_01852 |CDS_len=1008 |strand=+
ATGGGAAGGTCACCTTGTTGTGAGAAAGTGGGATTAAAGAAAGGGCCATGGACTCCTCAAGAAGACCAAAAACTGACGTA
TTACATTGAAAAACATGGCTGTGGTAGCTGGCGTGCTTTACCAACAAAAGCTGGACTTAAGAGGTGTGGAAAGAGCTGCA
GACTAAGATGGATAAATTATCTAAGACCTGATATTAAGAGAGGAAAATTCAGTTTGCAAGAAGAACAAACGATCATTCAA
CTCCATGCTCTTCTGGGTAACAGATGGTCAGCTATAGCAACTCATTTGGCTAACAGAACAGACAATGAAATCAAGAATTA
TTGGAACACTCATTTAAAGAAGAGATTAACCAAGATGGGAATTGATCCAAATACTCATAAACCAAGATCCAATTTTGGTT
CTGCAAACCTAAGTCACATGGCTCAGTGGGAAAATGCTCGGCTTGAAGCTGAAGCTCGACTTGTTCGCGAGTCCAAGAAA
CAACAAGAAGAAATTATTTTGATTAACAACAACATTAATAATTATAATAATATCCACTTCGGTACTAATAATTTGGGTAC
TAAAAATGTTTTACTACCCCTTCAAACAAAATTACCATCACTACCCTGTCTTGACGTGCTAAAAGCATGGGAAGGAGGGG
CAAATTGGTCAAATAGGCCAAAAATCAAAAAAGAAAAATTTTTCGATAATTTTCCAACATCCTCCACAAGCTTATCAAAC
AACAACTTGTTACTTATGGTACCAAGTAACAACAACAATAATAATAATATTTTGGGAGCTGGATTAGTCAACACGTGTTT
GGTTGGTGCAGGAAGTTTCTTGGGGAATAATATTAATGAAATTTCATCCTATTCTAGTTATCCATGTTTGAATGATATTC
AAGGGTTCACACAATTGGAAAGCGTTGTTGGAAGCTGCCAAGATGACTACGACAACAATGACAACAATTATTGGAATACT
ATATTAAATTCTTGTACTTCACTTGCTGATGGTTCACCAGTGTTTTAA

>CcMYB9 |Locus_ID=BC332_26517 |CDS_len=1035 |strand=+
ATGAACTTTTTAGTCCCTTGTGTTGAACAAAGTCCTTGGAGTTTTAATCATGAAGAAAATTTACGGAAGTTGAAAGATGA
AATAGAGGAAGAAAATTTAGTGGATATGAAGAAAAATAATAATAATAATAATGGAGGAACAAAGATATGTGTTAGAGGAC
ATTGGAGACCACATGAAGATTCAAAACTTAGAGAACTTGTTGCTCAATATGGTCCACAAAATTGGAATCTCATTGCTGAA
AAACTTGATGGAAGATCAGGAAAAAGTTGTAGATTGAGATGGTTTAATCAGCTAGATCCAAGGATCAACAGAATGGCATT
TTCAGAGGAAGAAGAAGAAAGACTATTAACAGCACACAAAATGTATGGCAACAAATGGTCAATGATAGCTAGGCTATTCC
CAGGGAGGACAGACAATGCAGTCAAGAATCATTGGCATGTCATAATGGCAAGAAAACATAGAGAACAAAACAGTGTGTAT
AGAAGAAGAAAGAGTACTTCATCAAATTTGCAACAAATTATTGCTCCTAATAATAATAATAATGATAGTATCATCCAAAT
TAAGAGCAATAATATGCAAAGTGATTTAACAATTTCTAGCAACAATTGCAACAACAACAACATTATTGTTGATGATCATC
AGTCAGCTTCAACTTGCACTGATCTTTCACTTACTCCCTCTTCTTCTAAACCCATGTTTCCTCCTTTTCTCATGACAAGA
TTTACACATCATCATCATGTGGAGCATCAAGGATTTCTTGATTCTAACAAAGGCATGTTGTCAAGAGATGAACAAGAGGT
GAAAATGGACCAAAAATTCTTGTACGGAAAAGAGCCTATTGAGAGTTACCATCATCATCATAATCATCAACATTATGGAT
GTGGTAATGTGTCAGATACAAATTCAGAAATTTCTGCATCAGAATCAGTAGCCAACAACATGACCAATGTCAAGATTTAT
GGTCATCAAAATGAAGAAGAACAAGATATAAAAACTATTCCCTTCATTGATTTTCTTGGAGTTGGAGCCACTTGA

>CcMYB90 |Locus_ID=BC332_27735 |CDS_len=549 |strand=+
ATGGGAAGGTCCAAATGTTGTGATAAAGAGGGATTGAAGAAAGGGCCATGGACACATGATGAGGACCAAAAACTATTGTC
TTTCATTGAGAAACATGGTTGTGGTAGCTGGCGTTCTTTGCCTGCTAAAGCTGGTCTGCAGAGATGTGGGAAGAGTTGTA
GACTAAGATGGACAAACTATCTAAGGCCTGATATTAAGAGAGGAAAATTCAGTTTACAAGAAGAAAGAAGCATCATTCAC
CTTCATGCTCTTCTTGGAAACAGATGGTCAGCAATAGCAACTTACTTGCCAAGAAGGACAGACAACGAGATAAAGAACTA
TTGGAACTCACGTTTGAAGAAGAGATTAACCAAAATGGGAATTGATCCAATGACTCACAAACCAAAAATCACAAACAACC
TTAATGGTGTAGCCTCATCAAAATATGTTGCAAATCTTAGTCACATGGCTGAATGGGAAAGTGCAAGACTAGAAGCAGAA
GCTAGACTTGTTCAAAAATCAAAAGACTCAAAAATCCTCTTCAATAACAACAAGTGTCAACAACATTAA

>CcMYB91 |Locus_ID=BC332_27736 |CDS_len=1038 |strand=-
ATGGGAAAGTCCAAATGTAGTGATAAAGAGGGATTAAAAAAAGGTCCATGGACTGCTGAAGAAGATCAAAAGCTTTTGTC
TTTCATTGACAAACATGGCTGTGGTAGCTGGCGTGCTTTGCCTGCTAAAGCTGGTCTGCAAAGATGTGGAAAGAGTTGTA
GACTAAGATGGATAAATTATCTAAGGCCTGATATTAAGAGAGGAAAATTCAGTTTACAAGAAGAAAGAACCATCATTCAC
CTTCATGCTCTTCTTGGAAATAGATGGTCAGCAATAGCAACTTACTTACCAAGCAGAACAGACAACGAGATAAAGAACTA
TTGGAACTCACGTTTGAAGAAGAGATTAACTAAAATGGGAATTGATCCAATGACTCACAAACCAAAAGTCACAAACAACC
TTAATGGTGTAGCCTCATCAAAATATGTTGCAACTCTTAGTCACATGGCTGAATGGGAAAGTGCAAGACTGGAAGCAGAA
GCTAGACTTGTTCGTAAATCAAAAGACTATGAAATCCTCTTCAATAACAACAATTACAACATTAACCGTAGTACTATTTC
CCAACAATTACCTTATTATCAACTCCCTTGTCTAGACATATTAAAAGCATGGCAAATGTCTAGCACAAAATTACCAACAA
TAAATGACATTAGTGCCATTCTTCTTAATAATTCGAGGAACAACAACCTCGAATCATCGATACCCTCAACGTTACATTCC
TCTGAGACTTTATTCGTGAACAATAATGTACCTACTACTACTACTACTACAAATGTTCGTGATGATCATCAAATTCCTCC
TAATTTATCTACAATCGAGTCGTGTTTTGAAGATGATCAACTCCAAACAGATCTTCCAAGTTTCATGCAGGAGTTTTCGG
GGCTATTTCCAGAATATTATACACAAAATTCAACAAATATACAAGTGGATAATTTGATGGGAAGCTGTTCTGGAGATTTT
GAAGATAACAAGTTAATTAATTGGAACAATTTCCCTAATTATTTGGTTAATTCACCTATTGGTTCTCCAGTATTCTGA

>CcMYB92 |Locus_ID=BC332_03833 |CDS_len=993 |strand=+
ATGGGTAGACTCCCTTGCTGTGATAAAGTAGGGGTGAAGAAAGGACCATGGACTCCTGAAGAAGATATAATGTTGGTCTC
TTATGTGCAAGAACATGGTCCAGGGAATTGGAGGGCAGTTCCCACTAAAACAGGATTGCGCAGGTGTAGCAAGAGCTGCA
GATTAAGATGGACAAACTACCTGAGGCCAGGGATAAAAGGGGTAACTTCACAGATCAAGAAGAGAAAACGATTATCCAG
CTCCAAGCTCTTTTTGGAAACAAATGGGCTGCAATAGCTTCATATCTGCCGGAGAGAACAGACAACGACATAAAAAACTA
CTGGAATACTCATCTAAAGAAAAAGCTGAAAAAGTTTCAATTAGATCAATGCTCTGGATCAGATGATCATCTATTCTATG
TAGAAAATGGTCTTACTCATTTATCATCATCAAATTCAAGGGGGCAATGGGAAAGGACACTTCAAACTGATATAAACATG
GCCAAAAAGGCTTTACATAATGCCTTGTCACTTGAAAACTCAACAAGCCCACTCATTAATATTAAACAAGAAAATCCACA
AGTTTCTTCTTCTACCTATGCATCAAGTACTGAAAATATATCTAGATTGCTACAAGGATGGATGAGATCATCAACTTCTG
AGAATTCAAAAACTTCATCTAACAACATTGCAACAAATGATTCATCTTCATGTGATGGGACACCAAATGCAGAAAGCAAA
GGTGGAATGGGGTTAATGGAAGCCTTCGAATCACTTTTTGGTTTAGAGTCATTTGAATCATCAAGTTCAGATGAGTTCTC
TCAAACAACATCCCCCCAGGCTGAAGCTACTAAAGTTGAAATCAAGAAGGAAGAAAACGAAAATGTGGAAGCACCAGCTG
TGCCACTGTCCATCATATTAGAGAATTGGCTACTTGATGAAAATACCCTCAATATTCAAGCCAAAGATGACTTGACTACC
TTCTCCTTTGATGAAACTGCTCCCCTTTTCTAG

>CcMYB93 |Locus_ID=BC332_06027 |CDS_len=915 |strand=-
ATGGGTAGACCACCTTGTTGTGATAAACTAGGGGTGAAGAAAGGACCCTGGACTCCTGAAGAAGATATTATGTTGGTCTC
TTTTGTTCAAGAACATGGTCCAGGGAATTGGAGGACTGTTCCCACTCATACAGGGTTGCGCAGATGTAGCAAGAGCTGCA
GGCTAAGATGGACTAACTATCTCAGACCAGGAATCAAGAGGGGTAGCTTCACTGATCAAGAGGAGAAAATGATTATCCAG
CTTCAGGCACTTTTAGGCAACAAATGGGCTGCCATAGCTTCATATCTCCCGGAGAGAACAGATAACGACATAAAAAACTA
TTGGAATACTCATTTAAAGAAGAAGCTGAAGAAGCTCGAAGCAAACGATTTATATTCCAGCGATGGACACTGTTTATCAT
CTATAAAGTCAGCCTCCAGAGGCCAATGGGAAAGGACACTTCAAGCTGATATAAACACAGCCAAACAAGCTTTACAAAAT
GCCTTGTCACTCGATAAATCAAGTCCAATTTCCGAATATACTATCAAGCCCATAAATATTGACTGTTATCCCTCATACAT
AAAACAAGAAGCGAAAATGTCTACTTCTACATACGCATCAAGTGCTGAAAACATCGCGAAATTACTCAAACAATGGACTA
GAAGTGATTCCACAAATAATTCTGAGCAATCAAAGGCATCGTCGAGTACTCAATTCTCTTGTAATACTACTAACAATACT
AATGCCATGACGGAATATTCATCATCATTTGAGTCATTTGAACCATCAAATTCAGATCAATTTTCTCAGGCTACGACACC
TGAGGCTGCTGGTAAATTTTATGGTGAAAGCAAGAGAGAATTGGATGATGAAGTACCATTGTCAGTAATGCTGGAGAGTT
GGCTGTTTGATGAAAATGACAATTTATTAATTTAG

>CcMYB94 |Locus_ID=BC332_26534 |CDS_len=1059 |strand=+
ATGAATGAATTAATTAAAAACCATCTTCTTCTTCTTTTTCTTTCTTCCAATCACCTCCCTTTATTTAAACACCTATCTAT
ATGTGCCTCCTCATTTTCATTTCAACTTTCTATACATACATCAAAAACACTTCACACAAAAACAACAACAACAACAACAA
CACCCCCCCCCCCCCNNNNNNNNNNCAAACAAACTATGGGGAGGCCTCCTTGTTGTGATAAAATAGGGATCAAGAAAGGT
CCTTGGACTCCTGAAGAAGATATCATTTTAGTCTCTTATATACAAGAACATGGTCCTGGAAATTGGAGATCAGTCCCTAC
TAATACTGGGTTGATGAGATGCAGCAAAAGTTGTAGGCTAAGGTGGACAAATTACTTGAGGCCAGGAATCAAAAGGGGGA
ATTTCACTCCACATGAAGAAGGAATGATTATCCATCTTCAAGCTTTATTGGGTAACAAATGGGCAGCCATAGCTTCATAT
CTACCACAAAGGACAGACAATGACATCAAGAATTATTGGAATACTCATCTAAAGAAGAAACTCAAGAAACTCCAAACAGA
ATTGGATTCACATAATTTGGCTCCTCTTCTTCCTAATAATAATAATAATAATATTAATTATGATAATCATCATCAATTCT
CATGGCAATTAAGTGTCAATAATAATGATAAGCAAGGGATCTCATCAAATTCTTCATCATCATCATTGTATGCTTCAAGT
ACAGAAAATATTTCAAGACTTTTAGAAGGTTGGATGAGATCTTCCCCTAATAATCCTTCTAGAAGAAACAATATTGATCA
TGACCATGATGATGAGATGTTGCATGTGGCACAAAAAAATGATCAAGATCAAGAAATTGTTAAGAGTACTTGTGAGGAAA
ATGAAAATTCTTGGATTATTCCAAAAGAGAAATCAAGTAATTGTGAGAGCAATGGAGTTGTTGTTGTTGATCATGGAAAG
ACAAGTCCACCTCCATTCACATATCTTGAGAAGTGGCTACTAGAAGAAAATGGTGGTCAAGTTGAAGAACTCATGGAACT
TCCTATGATATTCACATGA

>CcMYB95 |Locus_ID=BC332_09091 |CDS_len=1005 |strand=-

ATGGGAAGGCCACCTTGCTGTGATAAAATTGGTGTTAAAAAAGGACCATGGACACCAGAAGAAGATATCATCTTGGTTTC
ATACATTCAACAACATGGTCCTGGTAACTGGAGAGCTGTCCCTGGTAATACTGGTTTGCTTAGATGCAGCAAAAGCTGTA
GGCTCAGATGGACTAATTATCTCCGGCCTGGAATTAAACGTGGTAACTTCACTGAACATGAAGAGAAAATGATTATCCAC
CTCCAAGCTCTTCTTGGCAACAGATGGGCAGCGATAGCTTCGTATCTTCCCCAAAGGACGGACAACGATATAAAGAACTA
CTGGAATACTCATCTGAGGAAAAAGCTGAAGAAGATTCAAGGAAACGATGAAAATAGTACTGGTCAAGAAGGAGCAACCT
CATCATCATCTCAATCAAATTTCATAAAGGGACAGTGGGAAAGGAGGCTTCAAACAGACATCCACATGGCTAAAAAAGCC
CTTGTTGAAGCTTTGTCACTTGACAAATCTGATTCCCTGGCTAATCCAATTAGCCCAGTTCTAGCTCAACAAACCGTTCC
AGGGTCCAGTTCTTATGCATCCAGTGCTGAAAACATTTCTAGGTTGCTTCAAAATTGGATGAAAAATTCCCCCAAATCAT
CTCAATTAAGTCGATCGAATTCGGAGACTACGCAAAGCTCGTTGAATAACCCGTCAATCGGGTCAGGTTCGGGGTCAAGT
TCGAGTCCTAGCGAAGGTACCATAAGTGGTGCAACGCCAGAGGGCTTCGACACCCTCTTCAGCGAAGGCAATGCTTTCAC
GCCTGAAAATTCCGGGATTTTTCAGGTAGAAAGCAAGCTGAATTTTCCGAATAGTAATTCGGAAAATGGAATTTTGTTTC
AAGAGGAGTCACAAGCCCCGTTAACTTTGCTGGAGAAGTGGCTATTTGATGATGCTATTAATGCACCAACACAAGAAGAT
GAGTTTATTGGGATGGACTTGGGTGAATCTGCTGGCTTTTTTTGA

>CcMYB96 |Locus_ID=BC332_33109 |CDS_len=1113 |strand=-
ATGGGAAGGCCACCTTGTTGTGAAAAAACTGGGGTGAAAAAAGGACCATGGACACCAGAAGAAGATATCATTTTGGTTTC
ATATATTCAAGAACATGGTCCTGGTAACTGGAGAAATATTCCAACTAATACTGGTTTGCTAAGATGCAGCAAAAGTTGTA
GACTCCGATGGACTAATTATCTGAGGCCAGGGATCAAACGTGGAAACTTTACTGAACATGAAGAGAAAATGATTATCCAC
CTTCAAGCTCTTCTTGGCAATAGATGGGCAGCCATAGCTTCATACCTTCCACAAAGAACCGACAATGATATCAAGAATTA
CTGGAACACTCATTTGAAAAAGAAGCTGAATAAGAAGGTTGAAAGCCATGGTAATGATGCTCATGATCATGAAAACAATC
ATCAAGAGGGAGCATCAACATCATCATCATCATCTCAATCAAGAATCTCAAAAGGTCAATGGGAAAGGAGGCTTCAAACA
GACATTCACACTGCTAAACAAGCTCTTTGTGAGGCTTTATCACTTGACGCCTCTCCAAATAAAACTATTCCCATTAATCC
TGAACAACAACAACAACCAGCAGTTCAAGCATCTATGACCTATGCATCAAGTGCTGAAAACATTGCTAAGCTGCTTCAAA
ATTGGATGAAAAATTCACCCAATTCCACCTCTTCAAGTAGCTCGAAAATCACTCAACAAATGTCCTTGAACAACAATTTA
TCAGTCGGTGCGCTTTCGAGTTCTAGCCCTAGTGAAGGGACCGTAAACGTTGCACCCCCACAAGGTTTGGACTCGCTCTT
TAGCTTCAATTCATCTAATAATTCGGATGTATCGCAGTCTGTGTCGATCGATGAGGGTGGTAATTTGATCAGTACACCTG
AGAATAACAATGCTGGGATTTTCCAAGTTGAAAGCAAGCCAATTTTGCCTGATTTCAAGCCAGAAAGTGGGATTCATCAA
GAGGAGAGCAAGCAAAATTTGGAGACACAAGTGCCTTTGACTTTGTTGGAGAAGTGGTTGCTTGATGATGCTAATGCACA
AGCTGAACAAGAAGATCAGTTAATGGGAATTGGGATGGGAATGGCCATGAGTGAAACTGCTGATTTGTTTTGA

>CcMYB97 |Locus_ID=BC332_14191 |CDS_len=1362 |strand=-
TTATTAAGATGTGGAAAGAGTTGCAGATTAAGATGGACAAATTATTTGAGACCAAATTTGAAGAGAGGCAAATTTACTGC
AGAAGAAGATGAAACTATTGTCAAATTGCATAGCACCTTGGGAAATAGGTGGTCTTTGATAGCCCAGGATTTACCCGGCC
GAACAGATAATGAAATAAAGAACTATTGGAACTCCAATTTACGTCGGAGACTTTACTCCTTCAGGAACTTCAAAGAACTT
ATTAAGACCACTACAAATGTACCAAAAATCGCGGTTGATGGCGATTCTGATGAATCCTTGAGAAAACAAGATACAAGTAC
AATGTCGAAAGAGGGTTCAATTGAATCTCTTTTGCCAGAAGATAATATTGTTGTTGATAGAGGCTCCACTCGAAAAAACA
GGTCATTATGTGTTGGAAGCAATGAAGGGGCTACGTGGTCTGAAGGGGATTCAACTGAATCTCTTTTACCGAAAAATTGT
ACAGTTGATATAGGTTCCATGCGGAAACCCTACTCCTCGTGTCCTAGAGGCAAGGACGATGTTTCGTGGTATAGAGGGGG
TTCAACTGAATCTCTTTTGCTAGAAAATATAGAAATAAACTTCGCGTGTGAAGAGAATTCGATTGGGTCCCTTTTGATGG
AAAATTGTTCTTGTCCTGGAGGCAGGGATGATACATCGTGGTCTGAAGAAGGGGGTTCATTTAAATCTCTTTTGTTGGAA
AATATAGAAACCAACTTTTCATGCCCCAAAGGCACATGCAATGCTACGTGGTCTGAAGGGGGTTCAGTTGAATTTTTTTC
GTTGGAAAATTGTGCTGTTGATCTAGGTTCTAATCGAGAAAACAAATCTGACAAAGGGGGTTCAATTGAATACCTGTTGA
CGGGAAATTATACTGTTGATATAGGTTTTAATCGAGAAACCAATTCCTCATCATGTCCCAGAGGCAACGCCGAAGCTATG
TGGTATGAAGAAGGGAGTTCAATTGAATCCTCATCGACAGAAAGTTATATCGTTGATCTAGACTTTCTGACTCAGTCACC
GACTCGTAAAACGGAAGAACTAGCCATGCAACAACATGAACATCATGCAAAATCAAAGATACATGAGCCAAATGAAGAAG
GAGAAGCCAAGAACATCAAAGACATTATTCATGATTCACCTAAACAGCAATTGTGTCAAGGGATATGTTCCATTGGGGAA
CAAGAACAACAAATACAAGGACCACATGAGCAAAATGAAGAAAATGAAATTGTAATTCAACAAAAACATAATGATTGTTT
GCATAATTTTGATAATAATAATTGGTCTAATATAAATTTTGATTATTTCTTAGAAGATGAATATGGAATTTGGAACCATT
GA

>CcMYB98 |Locus_ID=BC332_02702 |CDS_len=1353 |strand=+
ATGGTCTCTCTATTTATTGGCACTGGGCTAATTACGCTGCCTCCACATTGCCACTTCAATAGTAATATAATAATAAGAAG
AGAGAGAGAGAGCTACTATTGGTGTTGGTACGCAAATATGGGAAGAGCGCCTTGTTGTGAGAAAGTGGGTCTAAAGAGAG
GCAGATGGACTGCCGAAGAAGATCAAATTCTCACTAATTACATTCTTTCTAACGGTGAAGGCTCTTGGAGGTCATTGCCC
AAAAATGCTGGATTGCTGAGATGTGGAAAGAGTTGCAGACTTAGATGGATTAATTACTTGAGGTCTGATTTGAAGAGAGG
TAACATAACCTCTCAAGAGGAAAACATAATCATTAAGTTGCATGCTACTTTGGGCAACAGATGGTCTTTAATAGCCGGAC
ATATGCCAGGTAGAACAGACAATGAGATAAAAAATTATTGGAACTCTCATCTAAGTAGAAAAGTTGAAAGCTTAAGAATA
CCAAGCGATGAAAAACTGCCTCAAGCTGTAGTTGATTTGGCTAAAAAGGGTGCATTGATGCCAATTAAACAAAGCAGAAA
AACAAGTCGAATATCCAATATGAAGAAAAATAAAACGTCAAAGAATTCAAACTTGGCAGAGCCTAAGGAAGCTAAAGAAA
ATAGTACTAGTGGACCTTTGAGTGCATCAGCAGTTTCTATTCCCTCAACACTAAACATGGAAAAAGAGGCATTGTCTAGT
ACTATTAGCTCATGGCTAGATGGTAAGAACATGCCAGCTAGTGATAGTGCCATGGATTCAATGCAAGAAGATGTAGCAGA
CGTAGCGGCGCCAAATCCTAGGGTGGGGTCTAGAGAGTCCCACAGCAGCCTTAGTTCAGAAGATTGTGATATGGAATGGC

TTGAGGAGATTATGCCAGACGTGGTCATTAATGATCAAGATATGGACCCAAATTTCATTTTGAGTAGTTTAGAGAACGGG
CAAGGAGAAACCCCTTTGAACGAAACAGGAAATAATTACTGCAGGACCACGCACAGAATTGATGAACGAAATAAAACTGT
ATCAAGTCAGGAGGCAGTGTCATCAACAAATGAGAAAAGCAGATTATTAATGGAGGCTGCAAATGATGGTAGACTTGAAT
GGGATTGGCAAGATGATCATGAATTAGTATGGCCAGCCATGTCATGGGAATTAGAAACAAAGCAAGAGAACATAGGGTCA
TCATCAATTCCTAGTTGGCCTCTATGGGATACTGGCACAGATTTACTTCAAAATTTTATGAATCATGAAGATCCTGTGGA
AATTGATTCAATGCACATTATGAGTGAAAATGACCAAAACCCTAGTGCCCTTGCTGCTTGGCTTTTGTCTTGA

>CcMYB99 |Locus_ID=BC332_14511 |CDS_len=813 |strand=-
ATGGGAAGAACACCTTGTTGTGAGAAAGTAGGTCTTAGGAAAGGGAGATGGACTAAAGAAGAAGATGAAATCCTCACTAA
CTACATTCTTGCTAATGGTGAAGGCTCTTGGAGGTCATTACCCAAAAATGCAGGGTTACTAAGATGCGGAAAGAGTTGTA
GATTGAGATGGATGAATTATTTGAAAACTGATTTGAAGAGAGGTAATATAACCTCTGATGAAGAAGCTATTATCATCAAA
TTGCGTGCAACTTTGGGTAACAGGTGGTCTGTAATAGCAGAATATTTACCAGGTAGAACGGATAACGAAATAAAAAACTA
TTGGAATTCTCGTTTGAGTCGAAAAGTAGATAGTTTAAGAATTCCAAGCGATGAAAAATTGCCACTAGCTGTTGTCGAAA
TGGCGAAAAATAAAGGAATACAAGAACAAATAGTCAAACGACAAAGACGAAAAAGAAAAGCCGATTCAAATTTCATGACT
GAAACTTCAGTTTCTACATCAAGTTGCTATAATAATAATAACAACAACGACGATGTTGTTTTGATGGACGATAAGGATGC
AGAACTGGATCAAGATTTTATTTTTAATTGTTTATGGGATTGCGAAGGAGAAGATCTTGAAATAGTCGAAAACAATAATA
ATGAAGTATTGAGTTATGATAATTATGATCAACTTGATTGTTGGAATTGGGAACAATTAGATGAAATATGGGCAAATGAA
GAAGCAACAAGACAAGAGATTGATAATAATGACAATGTGCAAAATTGCATGAACCAACAACAAGTACCAATAGAAAATGT
TGTGATTTGGTAG

>CcMYB122 |Locus_ID=BC332_25527 |CDS_len=849 |strand=+
ATGGGAAGGTCACCTTGTTGTGAGAAGGCACATACAAACAAAGGAGCATGGACTAAAGAAGAAGATGAAAGACTTATTGA
TTACATTAGAGCTCATGGTGAAGGTTGTTGGAGGTCTCTTTCTAAACCTGCCGGACTTCTTCGATGCGGCAAAAGTTGCC
GTCTCCGGTGGATTAATTACTTGAGACCTGACCTTAAACGTGGTAACTTCACTGAAGAAGAAGATGAACTTATTATCAAA
CTCCATAGCCTCCTTGGGAACAAGTGGTCGCTTATAGCAGGAAGATTACCAGGAAGAACAAATAACGAGATAAAAAATTA
TTGGAACACACATATAAGACAGAAGCTTTTGAGTCGGGGTATTGATCCAACGATACATCGGTCTATCAACGAGCCTACAC
CGAAAGTGACAACCATTACTTTTGCTGCTGCTGCTGACAGTACAGATGATCATCATCGAGATCAAAAGATAAATATCAAA
GCCGAATTTGATGAAACAAGCAACGAAGATAATAATGAAGTTCCAAAAATGTCATGTCTTCCTGACTTGAATCTTGAGCT
CAGAATTAGTCCTCCACATCATCAACAACTGGATCATCATCATCAAAGATCAAATTCTTTATGTTTTACATGTAGTTTGG
GAATACAAAACAGTAAAGATTGCAGTTGTAATAATACTAATGGAATTGGATGCAGCAATAATATTGTAAGTATGAACATC
GTAACAGGTATTAGCCTATGTTGCTTGAATGCGGCTGTTTTGCCGCCGAACCCATCTCGACATGAGACTGAGGTGTGGAT
GGGTGCTTTTGCCGTCTCTGATTCGATCAATCGATTTCAGAGATGTTGA

>CcMYB123 |Locus_ID=BC332_13322 |CDS_len=813 |strand=+
ATGAAAAAGCTTGTTGAGTATATCAAGAAGCATGGTCATGGTAGTTGGCATTCTCTTCCGCATCTTGCAGGTCTTGCTCG
TTGCGGGAAGAGCTGTCGTCTTAGATGGACGAATTATCTAAGGCCAGACATCAAAAGAGGACCCTTTAGCCATGAAGTAG
AAAAGTTTGTCATACAGTTACATGGAATGCTTGGCAATAGATGGGCTGCAATCGCATCACAACTGCCTGGAAGAACGGAT
AATGAGATCAAGAACTTGTGGAATACTCACCTGAAGAAACGCCTACTTTCAATGGGCATTAATCCTCAAACTCATGAACC
TTCTTCAGCACCTAATGGGTTGCTGGTAACTCCACTTACATCGCTTGCAGCCCGCCATATGGCACAATGGGAAAGTGCAA
GGCTTGAAGCTGAGGCTCATCTCTCCAGGGAATCCCAACTCTTGGTTCCATCATCAGTAGAAAGTTCTGGAACCGACTAC
TTCTTGCGCATATGGAATTCAGAAGTAGGAGAAGCATTTAGGAAATTCAACAAGAAAAAGGCAGGACTAGTGAAAGTCCA
GCCTCTCAAGCATCTTCATCAACAAAATACAAAGTGGAAGAATGGTCAACCATATAAGGGAGATGTCTTCCAAGGATACG
ATGACACCTCTAGCTCCAGTGGACTGGAGGATTCATCTGAATCAGCACTGCAACTTCCATTTGATTTTTTGAGTAATAAT
GACATGAGCTTTCTTAGACACAGTGATACCTACAGCTTGTATCCTGGATTCTTGAGTGAAAGCTCCTTGAAGTGCTCTTC
AACAGAACAATAG

>CcMYB124 |Locus_ID=BC332_14051 |CDS_len=714 |strand=+
ATGGGAAGATGGGGAATTATAGAAGAAGAATGGAGAAAAGGTCCTTGGACAGTTGAAGAAGATAAATTGCTCATTGAATA
TGTTAATTTGCATGGTGAAGGTAGATGGAATTCTGTTGCCAGGCTTGCAGGATTGAAAAGAAATGGAAAAAGTTGTAGGT
TGAGATGGGTGAATTACTTAAGGCCAGACCTTAAGAGGGGGCAAATTACTCCATATGAAGAGAGGATAATTCTTGAACTT
CATGCTATATGGGGAAATAGATGGTCAACAATTGCTAGAAGCTTGCCAGGGAGAACTGACAACGAAATCAAGAATTATTG
GAGGACTCATTTCAAGAAAAAGGTGAAGAAATCAAGTACTGACAACTTCGAAAAAACAAAAGCCCGTCTTCTGAAAAGAC
AACAGTCTCAACAGAAGCAATTATTGAACAGTCAAATCGACATGAAAAGGTTCATGTCACAAAATGAGAATAAAGTTCCA
AGTCATGTACCAAAACAAGACATGGCCATTTTGTACCAAAATAACTCATTTCATGAGCAAGAGCAAAGTGGTTTCTTCTA
TTCTATGAACAACGGATATGCTATTGTGCCTGAGGCCTCGTCTAATGAAGACATCATGTGGGATATTGGCTTGTGGAACT
TGGATGATTTTAATGTTAATTTGCCGGTGTCAGCGGTAGAATCAGGATTTGCAATAAAAGAGTTCAAAATATGA

Protein Sequences

>CcMYB3R-1 |Locus_ID=BC332_21354 |Protein_ID=PHU09494.1 |Protein_len=1038 |
chr08:163059837-163069990 |strand= -
MESDRISTPSDGTSSSFQRVRSLHGRTSGPTRRSTKGQWTPEEDEILCKAVQRFKGKNWKKIAECFKDRTDVQCLHRWQK

VLNPELVKGPWSKEEDEVIAELVKKYGPKKWSTIAQHLPGRIGKQCRERWHNHLNPGINKEAWTQEEEVTLIHAHQIYGN
KWAELTKFLPGRTDNAIKNHWNSSVKKKLDVYLASGALAKFPTLPNVNHQNQSIPSSIKLQQSSEDDSVRKEGTEMEEVS
ECSQSSNLAGCSQSTSDMGNTFVYTREEGKLLEDSNYRKDPSASSAPCSEYYTPAFEDITFSVAEVPSELAESKLLEHNF
SQNWATSMGKDWQFNPDDIPNISPLELMQDSSGFFMQCLTGTGNHDMVTFPQQNEMKFETTNDGSMVVGLHKPNEMFTSV
EGCSMVYPEAGIPQCFPSEIGMNGVDETAGSMIYQISEGGNMSIEKCNPLFSDVMGTASCQPCSVPSQFSSEQSSHMFGI
ASNQFHNPLHTARESQTNKCDGFLYPMESDTPCDNIMDNPLLKDQLDQTSDSVQLVSVNDFRSTPSNTIQTCPLVNENST
VKEDQMDGGALCYEPPRFPSLDVPFFSCDLIQSGTDTHQEYSPLGIRQLMMTSVNCLTPFKLWDSPTRDGSPDAVLRSAA
KTFTSTPSILKKRHRDLVSPLSEKRCGKKLESDLCQESFSNLTKDFSRLDVMFDDSANEKAMPSLTKDQTLELQASEDKE
NINPTEDGQKEEDKGRDGHSSCGTSGRQLDGGEVHYKDQGTRESKRLGAKDAVGKIKQPSGVLVELNASGLFFSPDRFGA
KSDRGSNLSNKALGNQYSRRLEAASNQGSVSSSFETSSFSVICSPRIHGKKDGSSFVITTSMQSAPTSTALESSAETSGN
GAGAGIIASISGETPYKRSIESPSAWKSPWFLNSFLSSPRLDNELTFEDLALFMSPGDRSYDAIGLMKQLSEQTAGAFAD
AQEVLGGDTPESILRGRNSKNQNADENRSLLSANAMSERRTLDFSECGSPEKEKETEKCSTNNNSFSSPSSYLLKGCR

>CcMYB3R-2 |Locus_ID=BC332_00348 |Protein_ID=PHU28255.1 |Protein_len=633 |
chr01:6524478-6532140 |strand= +
MIQVKEEALTLEFGGFTSCSSFSDSSYEASTPRCSSEPGSSCQRSSGQTKRSSQAGWTAEEDNLLTEVVKRFKGRNWKGI
AECLNGRTDVQCLHRWQKVLNPELVKGPWTKEEDDHIIELVEKFGCKKWSIIAKSLPGRIGKQCRERWHNHLDPTIKRDA
WTELEDLVLCRYHQIYGNKWAEIARFLPGRTDNAVKNHWNSTVKKRLDLNLLSRLGPDIQSEGSPDFSSHKKKLEIKKHL
AQAHNAETLSLSKQAGVDNAADACSTDLRIGCACSPRNDLHKGTSLFGACKSSVKNARDLIKPLGGIQFGKAEILPNGET
DQSNLNLTKISDPLLTSLDFPLDPSRKTRWSSSQVKAVHPTTFGRMYESPKRSKYGTVSDTDHDFLTLSLAGFTEVHSQG
NKKNKAYDIQSSLALKQPGFTEVHSKGNKKNKAYDTQSSLVLKQPGFTKIHSRGYKKNKAYDTQSSLAHKQPGFLNYEPP
KVKDLTIPLTDENLSRDNIIGRQNSHLLCSTPLSLTVSANSSSPESVLRNSAMSYTKTPSIIRKKTSRFSEASGHYSCTG
TTTPMHFLRSVPDREDTSNLKDRISGCKRSVSGKSLGRRLEYAFDMEWDSSRCCTPVSAASPCAIGANTMLTP

>CcMYB3R-3 |Locus_ID=BC332_23333 |Protein_ID=PHU06844.1 |Protein_len=451 |
chr09:34354987-34359612 |strand= +
MDELQVEECCIENKQSVAASSSSVSENSGSVNLRSPAVSSPTPVSPTHRKTTGPIRRARGGWTPEEDDTLKKAVSVYRGK
CWKKIAELFPDRSEVQCLHRWQKVLNPELVKGPWTREEDDKIIELVAKYGPIKWSVIAKSLPGRIGKQCRERWHNHLNPN
IKKDAWTLEEERALIDAHRIHGNKWAEIAKVLPGRADNGIKNHWNSSLRKKLDFYLATGNLPSVKSEELLVCSIKTSEAA
DVVSSGAIDICKIENGWQVTEINGGPQAESTDSEIVRLEAQSPEEDAIQHIKQSTESENRCNGWNIDSAVGRLQVFEAPF
PCEISTCGRLYYEPPRSESCIPLDSDLLNIGWAQCASDASPSPSPNGFFTPPSTKDHSLYAQTPESVLRTAARSFNTPSI
LRKRKSQGKLSTPTNKMRKSDGDFCKDKLIDACDKVQFDSSDKSSMDNGVS

>CcMYB3R-4 |Locus_ID=BC332_29224 |Protein_ID=PHU03973.1 |Protein_len=995 |
chr11:250147876-250156283 |strand= -
MLDYQDLSLSQATMESDETSKPPSDDISSRPLHGRTSGPKRRSVKWTPEEDEILRQAVQQFKGKSWKKIAECFKDRTDVQ
CLHRWQKVLDPELVKGSWSKEEDDKLIQLVNIHGPQKWSNIAQELAGRIGKQCRERWHNHLNPEIKKEAWTEEEELTLVR
AHQAYGNKWAEISKYLPGRSDNAIKNHWHSSVKKKLASGLLAQSPALPNVNHQNQSIASSSMKLQETSEDESVHKEGAEV
EEVTECSQGSTLAGCPQSTSDLGNTFMHIRENDAMLEESIHKKDASSGAAPCPRNSASAFQDVSCSMLNVPSDLVDSKFL
EYMFSHDGDNSTERDWQFNMDDFPGFIEEFSGHYMHCSNDNENHAMVADPLQTAMGSGATSNAANMVEGPYKPNEMFDGC
RIVYPEAAISQCSPSETGVNGTGEPADCLIYQSSNYQIPEEGNMAPTNCNALSFDDVEASTHQQFSVPSHFSSEDRSLVF
GIASDQFHYPPLENSVLESFTSRCDDFICPSEFGSPSNDNGIDNAVLKDLPDHTKDSSRLEEQKDGGALCYEPPRFPSLD
VPFLYCDLIQSGSNTQQEYSPLGIRHLMMSPANCLTPLRLWNSPSGDDSPNAILKSAAKTFTGTPSIVKKRQRHLVSPLS
EKRCEKKLESYLNQESFSNMATEISRVGDMFNESANEKASTEDKENLHPSSEDGRKEQGDGVTGLSCIENSERQLDGGGA
HYHKDPNSESAGANDTLGKVKQPPGVLVERSSSDMLVSPDRFLIKCDRATSLSNKALDNQYAGQLEAASNQVNVSSSFET
SFLSVFCSPDILGKCRSSVVKATSMQSTTSTALVNTAENSEKGFGAETSNIFGEAPLKRSTESPSAWKSPWSLNSFLPSP
RYDTELTFEDFAPFMSPGERSYDAIGLMRQLSEQTAPSIADARQILGSETPETILSKRTKQQKADEDCALLASNAMSERR
TLDFSECVTPPRKGKETTKFGSVSSPSSYLLKCCR

>CcMYB3R-5 |Locus_ID=BC332_25921 |Protein_ID=PHU05099.1 |Protein_len=562 |
chr10:174296581-174317749 |strand= +
MLNGKILMRTTGPIRRAKGGWTPEEVIRFPDKNHLLIINSPMPQDDTLRSAVAAYRGKSWKKIAEFFPDRSEVQCLHRWQ
KVLNPDLVKGPWTQEEDDKIIELVARYGPTKWSIIAKSLHGRIGKQCRERWHNHLNPNIKKDAWTLEEELALVDAHRIHG
NKWAEIAKVLPGRTDNAIKNHWNSSLKKKLDFYLATGNLPPVGRDGPQNGCENNIFTASAEKALVYSHKGSDSTELASSG
TTDICKIGNGGMNHQDAKLLVANIDASNSGPQNESTDSEVVRWEAQSPAIDGIHVKPITKIKFDGYGTNSVVDLKVIETP
FSCEIPTCGTLYYEPPQLGSCISMDSNYLNIRWAHSGPSPSLSPSSFSTPPSSLKGSSYAQTPESILETAARSFPNTPSI
FRKRRLSSTTPGNKIGNADEDMRKEEPCDTCEEKQVDSSEKYGMNRGSSSRNPAYNGIGPCNTRAFNASPPYRLRSKRKS
IFKSVEKKLKFAFDNEQHNTSNDSTIKEISHVADCLRATKMGFMLESVFNPGIIRDTGCHAVGFFVKPHQLPEEFTLHIH
LC

>CcMYB5R |Locus_ID=BC332_09390 |Protein_ID=PHU24283.1 |Protein_len=994 |
chr03:257728677-257740791 |strand= -

MAFDSDDDDFSGTDSDEGFQADMEALKKACLLSGKDADDLQPSSSSADGHVAGANDVTLSDTDADEDEDDDFELVRSIQE
RFALSTEVHDPISMKPLCSILPPGSEGDEDDDFETLRVIQRRFAAYGDDNGNGREESLFDKFEQISEECSNDVARSKNLT
DGHDSDAETTAISVNSSRFPKSAHAFVDAIKKNRACQKLIREKMMQTEARLEELKKLKERVKILKSFQLTCKKKMGRALS
QKRDARVQLISLPKQRFSAKLPGKKLSAIHSGPPENSHVASFKEALTQFAVSLSRKDWSKEESENLAKGVKQQFQEMLLQ
RSVNLPVNLLSDEDGCSRESGDLDDVIASIRDHKITPETMRSFIPEVNWDQVASMYLPGRSGGECQSRWLNWEDPLIKHE
GWDLSEEKNLLQVVQQKRMSNWVDIAASLGVSRTPFQCLSHYQRSLNASIIRGDWTDEEDSRLCAAVETFGESNWQVVAS
VIEGRTGTQCSNRWIKSLHPARRRCGKWSADEDKRLKVAVMLFYPKCWRNIGQSVPWRTPVWKKVAQYVPGRTHVQCRER
WVNTLDPSLKLDEWTEEEDLKLKSAINEHGYSWSKVAACVPSRTDNQCRRRWMVLFPDEVPMLKEAKKIRRDAFISNFVD
REEERPALKPNDIVLAHKLSKAGCETTSANKKRKRRPRTAKDDKTPKCDAVREMEKQHSEGSEGPESSDLINNQESDKIL
DGEEAVEQGCNDARKNKRRSRRHPRNTKKVKPNDKVPEASASSAGESTVADDNICKRRRTSSLVKKKSRTIASASITVDS
TIADGNSCKRRQRTSSQVKKKSSDKNARRLETSAVPGTLGGGTSGASVRHKLNKCNQLKNDDRSCVGNLPETVGDCMTLA
SFVRKSRAKGCSLSFTKVARLHPDKAQGKAMAGDHSSRSCISGGHDETEKRTSQECTSSNQISGTEVGDDMPLSLFMGKV
KREKIEVGDDMPLAFFIGKVKRGQPSGAQDRKRK

>CcMYBCDC |Locus_ID=BC332_12725 |Protein_ID=PHU17030.1 |Protein_len=1155 |
chr05:11257233-11267742 |strand= -
MRIMIKGGVWKNTEDEILKAAVMKYGKNQWARISSLLVRKSAKQCKARWYEWLDPSIKKTEWTREEDEKLLHLAKLLPTQ
WRTIAPIVGRTPSQCLERYEKLLDAACTKDENYDPNDDPRKLKPGEIDPNPESKPARPDPVDMDEDEKEMLSEARARLAN
TRGKKAKRKAREKQLEEARRLASLQKRRELKAAGIDVRQRKRKRRGIDYNAEIPFEKKPPPGFYDVTEEDRPVDQPKFPT
TIEELEGERRVDKEARLRKQDIARNKIAERQDAPTSILHANKLNDPEAVRRRSKLNLPAPQIPDHELEAIAKIGIASDLI
GGDELSEGNSATRALLANYAQTPQHAMTPMRTPQRTPSTKQDAIMMEAENQRRLTQSQTPLLGGDNPLLHPSDFSGVTPK
KREVQTPNPLLTPSATPGATGLTPRIGMTPSRDSYGMTPKGTPRDELRINEEMDNNAKFGQFNSKKELLSGLRGLPQPKN
EYQIVVQQPPEENEEPEEKIEEDMSDRIAREKAEEEARQQALLRKRSKVLQRELPRPPITSLDLIRRSLMRSDEDKSSFV
PFTLIEQADEMIRKELVSLLEHDNTKYPLDEKAEKEKKKGVKRKAVAEPAIEDFEEDELKEADELIKDEAQFLRVAMGHE
NESLDDFVQAHETTLNDIMYFPTRNAYGLSSVAGNMEKLAALQNEFENVKKKMDDDTKKATKLEQKIKVLTNGYQMRAGK
LWSEIESTFKKMDTAGTELECFRALQKQEQLAATHRINNMWEEVQKQKEFERTLQKRYGDLVAEKQKIQQLMDGHRIQAQ
IQEEIAAKNRALELAKAEMAEKDRALELAKAEMAEKESALELAKAELAEKESVPSAGDVEPSGTGQCSNTEEISASASHV
PIEADVHVEPSGTGQYSNPEENHASASDVQSAPIEADYVPVEPSGTSNAEENSASIEDDYVLVEPSATSNAVENSASIEA
DDVHVEPSGSGQCPIAEETSASVSHDVAPLDVEGQVKTGRVSAMDVEATSDHVPMEGQQNLVEESNMDVTKTEDSGAVAG
DADVPKTEDSGLVAGDTDVFKTEDSGVAAGDADVPKTENSGAVAGDADVPKTEDSGLVAGDMDVFKTEDSGVAAGDADVP
KTEDSRVVAGDADVPKTEDSGVVVTCDGEANAGKL

>CcMYB10 |Locus_ID=BC332_11130 |Protein_ID=PHU19979.1 |Protein_len=309 |
chr04:176182994-176184609 |strand= -
MAEGGGYGDDTRSSCCPRGHWRPAEDERLRLLVEQYGPQNWNSIAEKLQGRSGKSCRLRWFNQLDPRINRRPFTEEEEER
LVAAHRIHGNKWALISRLFPGRTDNAVKNHWHVLMARKQREQSKICGKRSLYQQQHDNFLSDSKSPSYGFRRRNINNNNN
MTKTHEGNYGSKINFFEFQNPNKDRVFSMSSTYSSNSHDFCRRNGSHLVRESSIDQKSLCQNNLSFLSHGGGANYSYKRS
TIIRNPFSYADRNESNGITERVVNITDGKFSLEKMLRESIEQQQQQYGEEARERKDIPFIDFLGVGISS

>CcMYB116 |Locus_ID=BC332_22985 |Protein_ID=PHU06496.1 |Protein_len=818 |
chr09:5931700-5937660 |strand= +
MQMVQQVSLDCDTCLHPETFMCKDGFSGVTQNPAVALPVIPSKDHPVSQQSVLTHDHAVTQLVSFSQDQVILPQPAKRQT
RQWAAWTRQEEESFFSALRQVGKNFERITSRVQSKNKDQVRHYYYRLVRRMNKLLGPELCLDAKNSKETNAAMLRWWSLL
EKYSCKASKLHLKPRRFKIFVETLESQLVKDRKKNVKRRPSQGESSSTAAASPSSHGRVSTNDSRTVKVVLLDNKDAQKF
GSGKGPSVKRNVTMGVNRGNVKVDSSVKNARHRRRTGSSSTAAYKRWEKAAIAGVSLVADAAEHLERTTIDKDVGPVQNS
QGINGFEHVGKDVHSLSTLSQIFLKETNLQSCMKLKLQLFPVDEGTRRALEMDNHNPFLELTLSNRKKMSSVMEHLNRKW
GSSSIATGELVLFPFHVQMESLVRSLRWTKDTTQIAADVHNLIGRPPVFRLKYGWFPNAELGTSQEPLSYTVPFVQNTNT
NITNEKNAEIPASSHGSLVRFSKEPLPSNPRMTVTSSSNKLGESNLQASMGLNTYTSDHDETLPSHRRENWDATTAEQDD
MHDMQGTKASALSAGDWEDSFTNISVGDLLFDAPDDEQTDCVDSALPGSSHFLHQTPLSCDSFDAAIAAHIYKHQSKADS
QMALPPQASSIWNAEDTCDAFAFKKNVAFRDKEQRSSSNVGAENSQRIAQSSSLVLDAGIKDLPGNMESFTGETAHEDPM
DECQSDAQALDGSAKDPNGLSGIYWTDSLGPLELDAPSCRYHNEDITLSDSFGGLNRLIVDSLDAFQNCSFFGLDKKEPA
SSTAKAVETSDLKIGAEV

>CcMYB119 |Locus_ID=BC332_09126 |Protein_ID=PHU24019.1 |Protein_len=513 |
chr03:253724494-253726291 |strand= +
MVQKRPFGEEDLYEVSSKQPRCEPSCQLGSVLEHSSLEAYASGGDEDNSSRTIPDANKKPDSHNVAEVLFSFDKETEMSI
HGSASNSSCPTSSTSEEEIMPEAPFHILTSPEYYYFDHPFRASAHHREIHSPLLSNPQKMVPIGPDFQAELPEWDAYNKD
NPCIDGIHETLSPSSQADENKFAGRTIVLMPKMELLADNVENIAERGVECSCEDIGSIRCVRLHNIEAREKLKLALGEET
FVRLGFCDMGEVVAEKWSEEEEELFHEVVLSNPASLGKNFWNHLAIEFPSRSREELVSYYFNVFILRKRAEQNRFDPLNI
DSDNDEWHETVDDADEGAKMTDEDEDSVVESPAYYNYLSYNMGHEEHDRPAWEYYNPVNFGSRKVFTDVQESCLGKLFDS
NSSCKLSMQPQDQGLPNKVVEQEVQGGSCITGAAGVTSESSLGKTNNNKHWASDIAGTGIGTNHDSLLEPSNRKEWDVGY
LSCARNKVDLLPTHTMIEEVFGDGAWIHKSRDV

>CcMYB120 |Locus_ID=BC332_11642 |Protein_ID=PHU20491.1 |Protein_len=585 |
chr04:216159659-216165069 |strand= -
MMEKRKFKRRVFVSEHDMSTLLQRYTAMTMLTLLQEVAQVKESKIDWNELVKKTTTGITNAREYQMVWRHLAYRQVLLDK
VDDDAQPLDDDSDLESDLEAFPPVSNETSLEAAACVKVLVASRAPCDTNMSDGNTIEVPLTISMPNRQTSGTAAENSLHG
ISAYGTKLTVPVTVQMQPLPSVTAAEGLDTSGPGTASYNSRRRRKPWSAAEDRELIAAVQKCGEGNWASILKEDFKGDRT
ASQLYQRWSIIKKRNMAGNASQLSEAQLAARHAMSVAFGDNLRAACPTSTNVGPNSSGGPSNSSHFAAADIASGGPQSKH
RQDLVPSKPIVPKNPLAEPAINPDPMAKAAAMAASSPIAVHSSAAASLQKAAQSKKGIHIMPGGTPAVKSSVPSSFSGLP
SNVHFIRTGLVSRPAGLSNASQSGTQQLQALRSASPAVQPRPATVPSRTNTASVVPSAPTSSPATAVLGVKSKAAVMQEN
QTAVLSNARSEKAGVIRAASLANTPQQQVQKGPTFGSGNRLTVKVEGPELGGESKASRIWIQEKLIPSQESPSNKNENTK
TELAAENTSGQGPAPVTCKVVFKDS

>CcMYB121 |Locus_ID=BC332_19144 |Protein_ID=PHU12214.1 |Protein_len=133 |
chr07:197563984-197565536 |strand= -
MADCDRSSTSDNASVVSAEFEQKLLSSRESTIDPPAVTALETTNKETSELEFSEDEEILIAKMFNLVGERWSLIAGRIPG
RSADEIEKYWKLRIYSKSQGESMSEILLKSKDQSQTIALKEESGHSTENVKGK

>CcMYB15 |Locus_ID=BC332_12046 |Protein_ID=PHU20895.1 |Protein_len=276 |
chr04:227488618-227493207 |strand= -
MQLPPSLGFSFTIIRCSTSTSSSSESSSSESTPRNDNKAEKIKGPWSAEEDKILTKLVERYGARNWSLISKYIKGRSGKS
CRLRWCNQLSPNVEHRPFSPSEDETILAAHAKYGNRWATIARLLPGRTDNAVKNHWNSTLKRRYQQLLQQQNSGQMVNGL
EFRSGSGSGSGSGSGSGMEYMNVDESPKGNTNNYATTMTTMAQCNYTSGVWRNNNNYGGGECDDPMTTLSLAPPGMAGEE
MPERKTESFPAGFWDVMRDVIAKEVREYVASTGFPN

>CcMYB18 |Locus_ID=BC332_23339 |Protein_ID=PHU06850.1 |Protein_len=361 |
chr09:35908165-35909250 |strand= -
MRERQRWRSEEDALLRAYVRQYGPKEWHLVSQRMNTPLNRDAKSCLERWKNYLKPGIKKGSLTEEEQHLVIQLQAKHGNK
WKKIAAEVPGRTAKRLGKWWEVFKEKQQREQKENNKVVDPVDEGKYDHILETFAEKIVKERSVPGLLMATSNGGFLHTDA
PTSTPQTLLPPWLSNSSAPSTVRSSSPSVTLSLSPSTVPPTPTPSIPWLQTDRGPENAPLILTSFPHHGVAPPCGENPFI
TELAECCKELDEGHRSWTAHKKEAAWRLRRVELQLESEKANKVREKMEEIEAKMKALREEQKATLDRIDAEYKDQLAGLR
RDAEAKEQKLAEQWASKHLRLAKFLEQMGCQSRLAEPNGGR

>CcMYB4 |Locus_ID=BC332_27825 |Protein_ID=PHU02574.1 |Protein_len=476 |
chr11:32488763-32494411 |strand= -
MQNAMKRSVEAAKTKERHIVSWSQEEDDILREQIRIHGTDNWTIIASKFKDKTTRQCRRRWFTYLNSDFKKGGWSPEEDI
LLCEAQKIFGNRWTEIAKVVSGRTDNAVKNRFTTLCKKRAKHEALAKENSNSYINLNNKRVIFPSGLNMDNITEATAPIK
KLRRSDISDVLQIGNEGEKSMGDCGTTNPLLRHPFAVLAQNLHNAGENLASHQRTTGSGSDNKTQGTFLKKDDPKVHVLM
QQAELLSSLAMKVNTENTDQSLENAWKILQDFLQQTKDGDMLKFQLPEMNFEPGDYKNLIADSRSSNEGTQPSWRQPALS
EDSAGSSEYSTGSTLLSHALADKTEESQAEVCAHHQDIESELRTSQMSDQAGLHELENGISCRVSTTRDTLPVCDEEKVN
NGPATAECEFLNTDFSSPHQVTPLFRSLAAEIPTPKFSESERQFLLKTLGVESTSLYPGTNLSQPPSCKRALLHSL

>CcMYB58 |Locus_ID=BC332_32563 |Protein_ID=PHT98484.1 |Protein_len=355 |
scaffolds:284407-286110 |strand= +
MGRPPCCNDKVRNRKCNWIEDDSNTSTYVSNKHGIGNWATMSKKSGYGRSGRIQKQRWSNRLKPDLKQDNFTPQEEELII
KLHATIGSRWSIIAQQLAGRTDNDVKNLWNTKLKKKLSAMGIDPVTHKPFSQILTDYGNIGGFPKARTRFVSLNRELKGA
FMSRPEQLQHSLQSFQNFHSLCVKLPKTEASEECFFSNNRDSSNVNQPPVDLLSELQAIKYVTEASNYNSPKAIFSHHPN
QMTDCSTSSPLSSSSSSSASHSLNENQVNWCDYLLDDAFIPSNFKDQEDELTIEEKLVSSGAQDGSNNVPSTRDFQASLS
TKGTSSSSSSFVEAMLECENEMFLNFPGLSEDPFY

>CcMYB62 |Locus_ID=BC332_19189 |Protein_ID=PHU12259.1 |Protein_len=187 |
chr07:200245005-200245699 |strand= +
MRCRKSCRLRWMNYLKPGLKKGNYSPEEELILKLHKELGNKWSAIATRLPGRSDNDVKNHWDAHHKKRIRLTNANSSTST
EKFTESSSSDSKNEQSCNSDEADYDMKEVPISSDLSNKLNGVTDWIEEEDNPTLLGQWKNFQVSIQWNNHFQILFAGQVL
LPIFRQTFDTNFWSESLLDNFWTQPFF

>CcADA1 |Locus_ID=BC332_14478 |Protein_ID=PHU18783.1 |Protein_len=454 |
chr05:236841288-236847872 |strand= +
QDILAFPLICPDWNADEEMLLLEGIEMYGMGNWAEVGEHVGTKSKEACVDHFKDAYLDSPYFPLPDMTHVMGKNRKELLA
MAKGNFTDKKGVSSLGEVVPKDESFSPSQVKVEDSHRKGPSGRLSAVSNAVINSMNASGITGIKKPSSKSQIKNQNEPVK
LEGRNFGGKKPKSLKEDGSSLMKLSGYIPKREEFDPEYDNDAEQLLADMEFKETETEQERELKLRVLRIYSKRLDERKRR
KDFILERNLLQPSEFEKNLSPEEKDLCRRYDAIMRFLSKEEHEELMKTVVSEHRYLKRIQELKESRAAGCHSSTEVDRYL
ERKRKKELEDGVPRKESSQIGPMSQESLNMPASSDSLGTHSNRKPCSQANLSSINDSGVAALSAGELLSEPEKQLCREIR

LSPHHYLRMQEVLTIQIYSGNITRKSDAYPLFQIEATKVDRVYDMLLKKGVSPL

>CcADA2 |Locus_ID=BC332_18760 |Protein_ID=PHU11830.1 |Protein_len=561 |
chr07:148423949-148442581 |strand= -
MGRSRPVRQSTDDDPTQRSKRKRAAPNVENFDTAAAGQMLTEGKKALYHCNYCNKDISGRIRIKCAVCSDFDLCVECFSV
GAEVQPHKSNHLYRVMDNLSFPLICADWNADEEMLLLEGLEMYGLANWAEVAEHVGTKSKLQCIEHYKSIYISSPCFPLP
DMSHVMGKSREELLAMAKEQGYAAPGEVNVKEESPFSARIKMEDQREENSAGLASVGGSASGKLAGAGKRTSAVLNSKEN
HDSIKLEGCPADRSVGEKKPRTSVDEGPSMTELSGYNSKREEFEIEYDNDAEQMLADMEFKETDSNAERELKLRVLRIYS
KRLDERKRRKDFILERKLLHPDPFEKDLTPEEKDICRSYRVFMRFSTKGEHEDFLRSIIEEHRMAKRIQNLQDARAAGCR
TSAEAERYIEQKRTQESEENIRRLKENSQNGPSGKYLQRAGHFKVEHDSSPSGVVRGPEMLDSCCKDLSSTTAPHGVGSA
LDIWDVSGFLGAELLSETEKKLCDEIRILPSHYLNMLQTMSMGILNGNITKRSDAHGLFNVDPNKVDKVYEMLVKKGLAQ
A

>CcARR1 |Locus_ID=BC332_14187 |Protein_ID=PHU18492.1 |Protein_len=663 |
chr05:230788833-230794556 |strand= +
MNLVASSSVSWKSNSNNSDRVEKNEFPAGLRVLVVDDDPTCLRILEQMLRNCHYEVTKSNRAEIALSMLRENRNGFDIVI
SDVHMPDMDGFKLLEHVGLEMDLPVIMMSADDSKDVVMKGVTHGACDYLIKPVRIEALKNIWQHVIRKKKHEWKDKDFEQ
STNVEDGDQHQKPPEDVDYSSSANEGNWKSSKRRKEEEDETEERDDTSTLKKPRVVWSVELHQQFVQAVNQLGIDKAVPK
KILELMNVPGLTRENVASHLQKYRLYLRRLSGVSQHQNGLNDSFMGHPEAAYGTMTSFSGLELQALAATGQLPAQSLATF
QAAALGRSATKSAISMPLVDQRNLFSFENPKLRYPEGQQQLNNSNKQINLLHGIPTTMEPKQLADLHQSSQSFVAMNMQG
NARMQQNNALLMHMSQQQQKQPSRGQMLNESSNGQVSRPPLSMPQPLSSNGIPAAILSRNGIVDNVRGPVYNPVSQASSM
VDFSLNQTTELQNSFPLVSSNSGMSTLTSKRLLPEEVNSDIKGSRGFPVGYDIFEELHQQKSQDWGIQNVGSAFGASDHS
GIPGTLDVSPSMLVQQGISSMKKNGQNGNSPMGGPQLNLFSGGNFLTVKAEQLPDTSYQSNFSLEQFGQDDLMSAILKQQ
ESVGEVETEFGFDGYPPLDNLPV

>CcARR10 |Locus_ID=BC332_02543 |Protein_ID=PHU30450.1 |Protein_len=688 |
chr01:196871152-196875839 |strand= -
MENDSAAVATTTTKKTTTTWSTWEELLLAFAVKRHGLKNWESVAMELQSRISLPVLLTAQICKDKYHDLRRRFMNSYNNN
SYDEKINDSFDEGVELDVDGNGDVTIPWIEELRQLRVAELKKEVQRYDLSIQSLQLKVKTMEEERERSLKEKSLDDGKRP
DLEDVKEERSQNDKNSGADVKPEDSTGKAVSSEESDRENRSFNESNSTENRETGVKNEPEPVEIGEDKPVQEVKPVSEAD
SYNDSSSDRQEKKVRRDSGDLRDTVSESKEETKENSDVQSTATLTKRKRQCSGGDGGRNGGDAAEMASPAVGIKGEVTAK
SEPLIEFLDIIRSHKRGSMFKRRLDSQKADKYKSIIRQHMDLETVQARIDDGSYCSCPAKFYLDLLLVFNNAIVYFPKSS
SESTAANELRSIVIEELKKTRTQSKDQSPGPRLAPLKIQLKPELEISDSLLAKHKSTVPIVVCRKRSSISAKAAGSGNNK
PEKHGDSKPPLNPKPPIKSSSNEEESSIKLGMKEKPVTGARSMRRSSKGRSNNNASPSNNNNNQNTNPKQTNSGGDKKED
AKPEKKKEESKKRGAAADFLKRIKKTSPTKGTLIEALKNDPTEDVVSVKVSNKKEQQKKKVDDRREVPVRRSGGGGGSAI
KEEGSPSKRSVGRPPKRGGREVVVQEKRGRENSEKDDSSKRPKKRSRR

>CcARR11 |Locus_ID=BC332_21691 |Protein_ID=PHU09831.1 |Protein_len=650 |
chr08:176152943-176154895 |strand= -
MASKSSVRLISYSEEIINGEPIYISSNCCPIKANKYEPAGLAFHSAALRLLGHVEKEDPEDGKEDVPNDKEQPFMYSSES
YSSKGKKKSSTGEKIQDHYALLGLSHLRYLASEDQIRKSYRDAALRHHPDKLASLLLAEETEAAKQAKKEEIENHFKAIQ
EAYEVLIDPVRRRIYDSTDEFDDEIPTECAPQDFFKVFGPAFLRNGRWSVTQPLPSLGDENTPIKEVDSFYNFWYSFKSW
REFPHADEFDLEQAESRDHKRWMERQNAKLSEKARKEESARIRTLVDNAYRRDPRILGRKEAEKAEKQRKKEAKILAKKL
QEEEAARIVEEEKRKKEEEEKKAAEVASQQKKLKEKEKKLLRKERSRLRTLAAPVLSQHLLGLTDDDVEGVCMSLDIEQM
RNLCDKLDGKGELVKAELLREALGQKHNSKYENKDENSKSQQNGSLEDKIQVSLMSSEKREKPWSKEEVDLLRKGVLKYP
KGTSRRWEVISEYIGTARTVEEILKATKTVLLQKPDSAKAFDSFLEKRKPAPTIASPLSTRAEVEGVDNSSKPESGSAKL
ANSQESLSQNTNSQKTEDAPAANGVSSSSDSDIWSAVQEKALVQALKTFPKETSQRWERVATAVPGKTVNQCKKKFALLK
ENFRNKKSAV

>CcARR12 |Locus_ID=BC332_02846 |Protein_ID=PHU30753.1 |Protein_len=1141 |
chr01:225825416-225842774 |strand= +
MAPAKKSKSINKRLSPTTEISPSKDGNGDNGKKSLQRKRKLSDMLGPQWSEEDLTRFYQAYRKYGKDWKKVASTVKFRTA
EMVEALYTMNRAYLSLPEGTASVAGLIAMMTDHYCNLAASDSEQESNEDAGTSRKPQKRAWGKVHPNISKTSEMTSSALA
ASHGCLTLLKKKRSGGSRPRAVGKRTPRFPVSFSYENPKGEKYFSPSGQSLKVQPHDTDDDVKIALVLTEASQRGGSPQF
SKTPSRWTDGAMFSPIGTAERKRVKVDMGNAKLLSNEVDEEEGSMEADTGELMRYKNDFEEAETVGRTTQKQKRAYGKKL
EVDDSGENHFDDIKEACSGTEEGQRLGAARGKLEMEATDEKNARASLQGPRKRSKKVLFGRDESSSFDALQTLADLSLMM
PAAENEDESMMQVKDEIDDHFDESGSLEAVPAHKQRDKRGSAGVKSRWSQSLSKFEVTSSRTSKHGKVTSSDVNAVPETK
QARRAQKAMSSKARKAEGHVDYDVTESQEAAVKDASKKSTSKGKRSYQCVSPKLIKDQEHSSCADPRTERSDSAQSTAQI
PVENQVNLPTKVRSRRKMDLKKPQKQKDLKIPDKSLDDTSASFTALRDKAFSLKKKISSCLSNHQVRRWCMYEWFYSAID
YPWFAKREFVEYLYHVGLGHVPRLTRVEWGVIRSSLGKPRRFSEQFLNEEKEKLNQYRESVRSHYTELREGTREGLPTDL
ARPLSVGQLVIAIHPKTREIHDGNVLTVDHSRCRVQFDRPELGVEFVMDIDCMPLNPFENMPTLLTRHADAVDKFFESFN
ELKMNARANEFMKFPAGDSTENGDVSSHFSPPSPPVSNLLKQTKVASAEADMQSKSGVMETTVYQQIAYSKPSTVAQIQA

KEADVQALAELTRALDKKDAVVLELSLMNDDVLENQKSDDCFLKDSEPFKKQYAAVLIQLNEVNEQVSSALYRLRQRNTY
HGSIPLAWQRPVPNFADPSILSTFDRCMSQPQESGFLVNEIIESSKIKSWTMVDAAVQAMLSYTGRDNTTEKIEEAIDYV
NDRIPLDDSCMPIPPDLKSKNVSDRRNEAEIPSELITKCVATLLMIQKCTERQFPPADMAKVLDSAVASLQPCCSQNFPV
YAEIQKCMGIIRNQIMALIPT

>CcARR13 |Locus_ID=BC332_24051 |Protein_ID=PHU07562.1 |Protein_len=1919 |
chr09:205001111-205026436 |strand= -
MHGCGAESDPIVDSEADSMGVGIGNKTSPQRSTIEEVQNELRQGYGFLEIKRRELEFLEKGGDPLDFILGNATSVSVQST
SLTDQHQDQLVTSEAKGSFAITASPHGDSVESSGRLGAPQLCEPNSADNLMLFDGENEFIEAERSCRYPSRSNITPSEQS
SKLDRSRNAKELGDSAAFGVPKKSYKRRYRPRPNGDGTRSSSTDIILGPGGHGTSLPSQHFSKDVKAQVSDGENPKDQSS
SLSIARPSIPNGFVPVKTPPSDNLLDTEIHGFKVAEATTYLKNEDLAYSIPEASASRCLLDNQHDQNSLADIEDVSIQES
PERPQSSLGKEGVGSAGQEGPLCTTAVGLENQASSSHHINGSCGKSDQKSMPNDAQSSGAALGTKGLDSESSCSRARSLN
RNTNNAMSNNPTNLDSNGDLKEQLSVPEATQVIESNLKEQKEVKTDDSCGFTNKGEVKDKITTQVEAVEPSPSETERKPT
TNISDSSNSQKLYSSIIGRQGSIECRIPVPSQHVSPHGVLTPFPEAQAPAINVKLATRGDEDSILKEAQIIEAKRKRINE
LSAVAFPLKNHRKSHWDYVLEEMVWLANDFAQERLWRMTAAAQLCRQVAFTARLRFQEQNSSCKLKKVAHIMAKAVMGFW
QSIEGKNEQLELPVTRKDHAIAIREYAMRFLKYNDSDVRQSLAEAPVTPERVSGGGIVDAPREDHLGEENLFYAVSFGAM
DAYRKSIESHALLCVKIGGSMHEEVETSACDTIPEFGAQDYAFEEDEGESSPYDTSVAIEGNKSSKFSQKQRKIHIKAYS
GRPYDVRTDVPFTRVEYKFGTQQSMQLGKRPSSNLNASIPTKRMRSASRQRVVSPYSATTSGCAQLPVKTDASSGDTSSF
QDDQSTLHGGSFMPNSLEVESVGNFKKHLPFDSAEVSKPKKKKKVKILGSAYEQRWQVDSNFQNEQRDPSRKRLESHQLD
SNGHNGLFGQHVAKKPKMMRQSIENSFENVGSVGGFVPSPAASQMSNMSNPNQIMKMLSGRDQGRRAKTFKMSAGQASSG
SPWSLFEDQALVVLVHDMGPNWELVSDAFNSTLQFKCIYRKPKDCKERHKMLMDRSGDGNDSADDSGSSQPYPSTLPGIP
KGSARQLFQRLQGPMEEDTLRSHFEKMILIGQKYLSRKNQGYKFDSRQIQQPHDSHTHALSQLYPNNLNGGPILTPLDLW
DAPSPSPDYPSVGCQGPRPSGLSVSSHALNSVLPVSGANSTVQASSSMIGESNLPSSSPLNASVRDARYVVSRPASLPVD
DQRLQHYNQMRNMQSNMSAPGVLAATNRAGVRTLSSGNSTCIMGGINRGIPMARPGFQGVASPSMLNSGSMLSSGMVALS
NTLNIHSGVRSNQVNSMMRPRDGSLMTQPPQNQEAQRQMMVPEHQLQASQGNSQVVPPFGGLSSSLPNQSASPVTPYPLH
LQQSHPMSSQQPLMLSPHHPHLQGANHATNSQQQAYAFRLAKERHLQHRLLQQQHFSHSQPQHPISSSLQNSPKTTSQSS
APPVSVTSPTSVTPISRPHALPAHGHAWTAQTAGSSLTTQMSKQRPRQTGGQQLQPAGKHQPPQRQQSQSQQEAKLLNGV
GRGNMMMHQNLQIDPSLMNGFSNNKANQSAWKGVHATSLMQGHGLYSGSANSPAHLVKQAMEPHSSSPQQPESKSYSGRL
PLSTKHLQQEMPCNPDNSNQSPASLDASDTNSSQLSVPSSVVGSSNHHELVHQQAQWQPQPKLLNQKEATVQRVLQQNHV
VNSNPSKRLQADEFQAEQRSTCKTSKIGAITSMPQDCNNPTTVSGVSPLSANQWKGAEPLFDSIGAPPTSSAGSKPVPQV
SRGVSQRQPSGNSSPTGPDFSVNWQQKSSQLQPPSSVTQPQLQQQQQQLSPSQHSQQQGQVLQAGNSSLFARPNDCRLD

>CcARR2 |Locus_ID=BC332_02094 |Protein_ID=PHU30001.1 |Protein_len=670 |
chr01:156123343-156127931 |strand= +
MNIGSGSLVTVPSSTGSWKSGDVVSDQFPVGLRVLVVDDDPTCLRILEKMLRKCHYEVTKCNRAEIALSFLRENKNGFDI
VISDVHMPDMDGFKLLEHIGLEMDLPVIMMSADDSKDVVMKGVTHGAYDYLIKPVRIEALKNIWQHVVRKRKYEWKDNNF
DQSGSVEEGDRQQKPSEDVDYSSSANEGNWKNSKKRKEEEEEVEDRDDTSNLKKPRVVWSVELHQQFVQAVHQLGIDKAV
PKKILELMNVPGLTRENVASHLQKYRLYLRRLSGVSQHQNGLNNSFMGRPDATFCTISSLNGLDLQALAAAGQIPAQNLA
TLQAAALGKSATKSAISMPLDQRNFFSFENSKLRFPEGQQQLNNSNKQIDLLHGIPTTMESKQLANLHHPSQSFVGMNMQ
VNSTSQHNNSVLMRLSQSQPRTQLLNGANNGSQVSRLPLSMQQSLSPVLTQSGIVDNARGSVYNPVSQASSMVDFSVNQS
KELQSYNFSLGSNSLGMSSLTNRGMLQEEVNSDIKGSRGFPSNYDIFNELQPKSQNWGLQNDGSTFDTSLHPCMQGTQDV
SSQLLLQQGISSTHNSGQNRNAPIEKPMYSNGDEIGHSNTMCGSQLNSGGGNMLAVKAERFPDADYQNTNIREQFGRDDL
MSAFLRQQASVGPVETEFGFDGYTLDNLPV

>CcARR3 |Locus_ID=BC332_31526 |Protein_ID=PHU01739.1 |Protein_len=568 |
chr12:225454845-225458703 |strand= -
MATSGECLNSGGFSGEFPTFRLLVVDDDPSCLRVLECMLKKCHYEVTTCEHAEVALSLLRENRNNFHLIISDVHMPDMDG
FKLVELIVLEMNLPVIMMSVDDSRSVIMKAVIHGAHDYLVKPVSIEALKNIWQHVVRQNKDEWKGKILDRLGNVEGAEYS
DAAKEGRLKSSKKRNDKEENEIGERNDTNTVKKARVLWSPELHEKFIQAINQLGGDSKIPQKILKLMNVSGLEREHVASH
LQKYRLQLQKNRSGLESGFMGHSEVTIGTLLNNPDVQTLAPMDQHPVQNLTTHQGPTWVRPKSPIFIKEPTWGSPTTKSP
IFMQRNHLSFQNPVLRYQGPQQMNNSNKQVNLPCGIPTTEQFLRMNIPVARIFQPQLQVQNMLSESNKILSQNGIVDDTR
DSMYNQVPRTAVDFSLNQNIPLAGTNFLLSANSTVSATTEQVNIGYDNFSELCQQKAQDWGLQNVVGSTFDALMEQNGQN
VNAPIAEAVFSSRQEIGHVNPIDGPQVNSVKAAERYTDSSYQSTIFLEPFEQDNDLMSALLEQQHESSVGQVDSVFGHDE
DAFENLPV

>CcARR4 |Locus_ID=BC332_13151 |Protein_ID=PHU17456.1 |Protein_len=632 |
chr05:43213413-43221277 |strand= +
MASMSGVGATCKSEATVVADHFPVGLRVLVVDDDVVCLRIIEQMLRRCKYSVTTCTRATVALNLLRERRETFDIVLSDVH
MPDMDGFKLLELVGLEMDLPVIMMSGDGRTNLVMRGVQHGACDYLIKPIRDEELKNIWQHVVRKRYNSNKEHECSGSLDD
NDRYKRGSDDVECASSVVEGADGVIKPQKKKREAKEDDTEVENDDPGTTKKPRVVWSVELHQQFVSAVNQLGIDKAVPKR
ILELMNVPGLTRENVASHLQKFRLYLKRLSGVVQQQGGIPSTFCGPMEQNSELVSLGRFDIQALAASGQIPPETLTALHA
ELLGRSTSNLVLPAVEQQNLLQASLQQAKCMPVDQLVAYGQPLLKCPPSISNSKHPSQSILSAEDVHSGFGSQRAKNICM

VPSSNPTGIAAPNSNMFMAMMQHQQQWQKQQQLELQHRQSGPLESNYSINVQPSCVVLPSQSPGNFQVGDSPASISRANS
LSKSSVVDYGVLSPQSNNSSGVIQVLDRELRPECGSNRLSSGGSLSCSCSINADNSVGLQFHNSSSAFGSSKQLPGLNPN
HLGSPVPYNINSSQVLDQGHIRNPVVGKGASIPSRFAVDECESAMCNLNTAKSYLEDAKVKQEPNMNLMENC

>CcARR5 |Locus_ID=BC332_28216 |Protein_ID=PHU02965.1 |Protein_len=581 |
chr11:102358775-102362626 |strand= +
MMMENTSKNSGGFSTPRSDNNTFPAGLRVLVVDDDPTWLKILEKMLKKCSYQVTTCGLAREALYLLRERKDGFDIVISDV
NMPDMDGFKLLEHVGLEMDLPVIMMSVDGETSRVMKGVQHGACDYLLKPIRMKELRNIWQHVLRKKMQEARDVGNHELDQ
YDEVWMLNGAEILSGKKRKDFDNKHDEKEMSEPRGVDSSSMKKARVVWTVDLHQKFVKAVNQIGFDKVGPKKILDLMGIP
WLTRENVASHLQKYRLYLTRLQKENEVKASFCGVKHPDVSSKETSSSLSLQNPVDVCTDVTNDKYGGVSGGKAIVQNGKS
SICESKVKVVVSVPAAEPSSVVGDNFDSPAAVSKIGLNDSFGLVNTNVKNSKVPTTYCSTGEAPQPQYKQDFNQSANGSS
HQPLPVVSHQIPVDHTQETCFLSHVPSREERDRHPDTKNKPSFFKSKSEGVGKYSPIESTINLFQQVNQQANFHTLQQMA
STTWTGTSHNVVNGPQSSAGNLTSRSGSIVASAGEDMCGASILGECFLANNGLPNTEQFGYNDPQPISGVPTCLYDTLRF
DYEYPVDSLEGIVMDQGLFIV

>CcARR6 |Locus_ID=BC332_00798 |Protein_ID=PHU28705.1 |Protein_len=560 |
chr01:20167363-20172786 |strand= -
MICIEDELLGWKDFPKGLKVLLLDEDSNSAAEMKSRLEKMDYIVYTFCNESEALSAISSKSEGFHVAIVEVSAGDNDGVL
QFLESAKNLPTIMTSNIHSLSTMMKCIALGAVEFLQKPLSDDKLKNIWQHVVHKAFNARKDVSGPLEPVKESLLSMLQLQ
PEKGEPDDKSSNGTEPLIAVADNNTEQSSGCDKYPAPSTPQLKQGVRSVDDSDCHDHTIFSADQDSGEHDEDIILKEENG
SSPHQTMEADIATFSQINDCADNSDGSSPHQKTEADIATTSSQSKDCPDNSISHSAEPSKASGPHSSSGTKSNKKKVKVD
WTPELHKKFVQAVEQLGIDQAIPSRILDVMKVEGLTRHNIASHLQKYRMHRRQILPREVERRWPHPQPRDSVQRNYYPHK
PVMTFPPYHSNHVAPAGQCYPAWVPPASYPNGLQVWGSPYYPGWQPAETWHWKPHPGLLADTWGSPVMPPSFGSYPPYPQ
NAGMYQSHGMHNRYSMLEKSFDVHPAEEVIDKVVKEAITKPWLPLPLGLKPPSMEGVLDELSRQGISTVPPRINGSRCWR

>CcARR7 |Locus_ID=BC332_15933 |Protein_ID=PHU14728.1 |Protein_len=557 |
chr06:193757966-193762406 |strand= +
MVCTENDLLGWKDFPKGLRVLLLDKDSNSASDMRSRLEEMEYIVYAFCNETEALSAISSKSEVFHVAIVEVSAGNSDGGL
KFLEGAKDLPTIMVSNIHSISTMMKCIALGAVEFLQKPLSDDKLRNIWQHVVHKAFHSGGKNVAESLKPVKESLLSMLEL
QPVKREADSENSNEAEPLTSVLENQKESPPCCDKYPAPSTPQHKQGVRSVDDGDFQDHTILSNEQDSEVHEGDTKSVETT
CCDSVAETSILADSAGRLEVAITKDERDSAADQNMEDPIATCSRSNDYPADGSTRSAESNKASGLHSSSGTKANKKKMKV
DWTPELHKKFVKAVEKLGIDQAIPSRILELMKVEGLTRHNIASHLQKFRMQRRQILPKEDEKRWPRPQLRDSVQRTYYYP
HKPVMAFPTYHPNNAPTAGQFYPPWIPPGGYPNGAHMWGSPYYPGWQPPENWHWNPHSGLYADVWGCPVTPPSLGSCTPY
LQNASGIHNRYSIIQKSVDLHPAEEVIDKVVKEAIHKPWLPLPLGLKSPSTESVLDALSEQGISAVPSRINGSRRPH

>CcARR8 |Locus_ID=BC332_23287 |Protein_ID=PHU06798.1 |Protein_len=427 |
chr09:29910164-29922823 |strand= -
MAAPYYGMIDEVHVVLVDDDKEFVNNMVDLLKSYDYKEDTPYWTKLLALLCTYCDSQDVIDRAGSMIYNGKSRHIKWRHN
TIRELLSSEIITVDYVRSKDNVLIPLTKDPSRKGVKSSLKGMDLRPRTSHHCVMVVGTALDAMAMLSKGKQKIDVMILNI
HSSNTLSFHLLARVVALDIISLVVWDEQNELVVKKAFDEGAYLCLKKPFDKDITKYIWQFVVGEKLKREKARKGFENNKN
QMNKIVKRKNCTEWTEALHAKFMEAVQLLGEGRCYPKEILEVMNVPGLTRMQVASHLQKCRRNDWRSPKERKSLHRPLGQ
GSSSDFHESRSNGIVSSRNIRNEISNGYNLNVSMDNVTSYSSNATMYDTYVGNVAINGLEEENPNVQQYIGEPNISNMNN
PFQKLEPPSASLHNEQDNDFDQVYYDD

>CcARR9 |Locus_ID=BC332_23270 |Protein_ID=PHU06781.1 |Protein_len=290 |
chr09:27579625-27581268 |strand= -
MNKIVKRKNCTEWTETLHAKFMKAVQLLGEGRCYPKEILEVMNVPGLTRMQVASHLQKCRPNDWRSPEERKYLHGPLGQG
SSSDFHESRSSFKKFGTMPHLQIKEPNLNCNPDNTQRNSDLSFPNLSTNNTFAREESPFQQHFYRPQLHVQSPYLSNFNP
FSNPFLSIQNTISGGLQQQQQQRSLFGILYSQGLQGPIIKNNNYRSDVVINSGHHHTQSDYTLNSIVAQGATYSGSGIVS
SRNIRNEISNGYNLNVSMDNVTSYSSNATMYDTHVGNVAINELKEANPNF

>CcMYBH1 |Locus_ID=BC332_28265 |Protein_ID=PHU03014.1 |Protein_len=298 |
chr11:121715985-121724090 |strand= +
MGAPKQKWTSEEEAALKAGILKHGPGKWRTILKDPEFSGVLFLRSNVDLKDKWRNMTVMANGWSSREQTKLAVKKMKQAP
KQDGSPMTDTTAAESDEEAAEARPATTSSGSPQIHGSKKSMIRLDNLIMEAISSLKEPGGSNKTTIAEYIEAQYWAPPNF
KRLLSGKLKYLTATGKLIKMKRRYRIVPTSTPSDSRRNLSITLLDNKQRIFSKIDRDDLNMLTSQIDLELAKMKHMKPQE
AAAAAAQAVAEAEAAIVEAEEADREADAAEADAEAAQAFSVAANKTLHGRSAPRMVRH

>CcMYBH2 |Locus_ID=BC332_09595 |Protein_ID=PHU24488.1 |Protein_len=288 |
chr03:260830127-260836063 |strand= +
MGNPKVKWTSDEEDALKAGVAKHGTGRWKNILRDPDFASLLSNRSNIDLKDKWRNMSIYPAGQGSKDKSRVARARPMAID
ALVPSTAQNVTPIASAFEPSLAQSVPAITSAFENEAIDDPPKNSQEGLHLSNDMIFEALSSMKGSNGSDLGAIVGFIEQR

HEVPQHARRLLTTKLRRLVLQGKLEKVENCYKMKDATSETKIPTPKQKHVRSWPVPNSAVKVSCGTTEEAARIAAEKIVD
AEHKCFLEVEAIKDAEVTSKMAEEAETMLLLVKEIYEQCSRGGVVLLA

>CcMYBH3 |Locus_ID=BC332_18613 |Protein_ID=PHU11683.1 |Protein_len=280 |
chr07:104281258-104308863 |strand= -
MGNPKQKWTSEEEEALRAGVAKHGAGKWKNIQRDPEFNHLLYSRSNIDLKDKWRNLNVSANGQGPRDKSRTPKVKTNLDA
AAAPLPIMQAPVSSTLVPQDASADTVMEDSSKCALDGKNASKYNQMIYDALSSLKEPNGSDTSTLVNFIEQRQEVPQNFR
RLLSSRLRRLVQQDKLEKIENCYKIKKEVLEKTKIATPKQKHVGPRQFPNSVYLGDTAEEAAITAAYKIAEAENKSFVAA
EAAKEAERVSKMEEESDNSLQVYKDIFDKCSHGDIVLLMA

>CcMYBH4 |Locus_ID=BC332_03653 |Protein_ID=PHU25321.1 |Protein_len=293 |
chr02:91837168-91842324 |strand= +
MGAPKQKWTAEEEAALKAGVAKYGVGKWSTILKDPEFAVVLRSRSNVDLKDKWRNLHVMASGWGSRHRGRAVSKSVQPAP
KHDDNNLVSSVGENDIEVLDAKPLASSGEVLKDVGSKKPLTRLDDLILEAIVKLKEPRGSSRNAISSFIEERFAAPPHFE
KLLATNLKALTEKGRLVKVKHYYRIAPSRVSSDAKGGSFSSLMGGKLDSTVVEKNETRILTKAQIDADLEQMKSMSAEEA
AAAAAQAVAEAEAAIAEAERAARIAEECEAEAEAAQCFAEVASKALQHQTIHV

>CcMYBH5 |Locus_ID=BC332_32845 |Protein_ID=PHT98253.1 |Protein_len=1066 |
scaffolds:437968-458977 |strand= +
MSYYDQPPPVGVPPPQGYPPKDASYGYPPQGYPPQGYPPQGYPQQGYPQQAQQGYPQQGYPPPPYGAPQYAAPPPQKNSS
GSTDILTGWIFFLNSWMGAPKQKWTPDEEAALKAGVHKYGPGKWRTILKDPEFSKVLSSRSNVDLKDKWRNMSVIANGWG
SREKARLALKRMNQARKQDESSLAVITEAKSDEKMAEARLATTSIGSPQIGGSKRSIIRLDNLIMEAISNLKESGGSNKT
SIATYIEDQYWAPPNFKRLLSAKLKYLTATGRLTKVKRKYRIATIPVLSDKRRIPSIPLLESRQRISATVDQDDFKMLIK
AQVDLELTKMRSMTPQEAAAAAAQAVAEAEVSMAEAEDAARKADAAEAYAEAAQALAEAAMKALKERSSPRIPTLLLACK
YGKSGVCNSKGLPAMDGNLNDNAFLRARIKELERERDELHKDIEQLCMQQAGPSYIGMPTRMPLHRIAGLEQEAESLKKK
LSACTRENQNLQEELSEAYNIKGQLADLHSAEVSKNIEAEKQLKFFQGCVAAAFSERDHAVMEAEKAKEQEELMSRGLHE
LQQRIEELSCELLEEKILTATLQGDMDREERLDEIFKEVVHKFYEIRRQTLEDIEDVSWEDKCACLLHDSSEMWTFSNPG
ETSTSNYISSLEEQVETLRKSLDNLQYKLLVGLEIENHLKKKVRALEKQKILSEEKLRAQISAFRHYHSQHRVNITSFLD
EGFSHIKSAMNMVEEKLKDCSISERDLNSSQVDDLQFSELECQDVQIKNVDGSDLIFKRNQPSLTTTITVGNSDASKALA
VALNEKVETLLLLSQQEERHLLERNVNAALQKKVEELQRNLLQVTNEKVKALMELAQLNRDYQVLHEKVNEASRQGKSLG
EIAVKRTLQEKDGRLKNLLKTSYLRRWTGIQDSDVNDADTHRDSEANFADRRPTNNVDFARMKIENATLKESLESMEHLI
RAVRRLRLSLLKFKESAASEGMENCYSVSLDNIINEASQLKTALGSSLPLSWSAEADSGSVSKHVEEETDNGHSTRENMD
FVSAAGFEMVELLVFVAQLLKDYKCS

>CcSWI3A |Locus_ID=BC332_08722 |Protein_ID=PHU23615.1 |Protein_len=601 |
chr03:242779171-242784712 |strand= -
MHNYSSKPISIHFTKGYLSTSPHYIVNQFLVCAGEFVMDTSRSQDEPDHDLYTIPSYTSWFSWQSIHEVERLSLREFFDG
SSITRTPRIYKEYRDFIITSYREDPTRMLSFTEIRKALVGDVTVLHKVFTFLEKWGLINFDPSNAAAAAAAVGGVAEEED
KEDEKWRIRVEEGAPHGVRVVAAPHSLKPLIPVKEVVIGDGGGGGRVGGVVKVSPLASYSDVYGELVGVEKKESVVCESC
KEQCASGYYEYNKDASINLCEKCFKSGNYDKNKSTDEFKLMDGANPNANWTEAETLLLLESVLKHGDDWDLVTQNVKTKS
KLDCISKLIQLPFGDFMLGSIHRKLNFLDKNGEVSGVDQAQPAISESGETPGNQSHEQNQEHQQNGDAEDETPPLKKIRR
APISEASSFLMKQVARISGAVGSHITASAAEAAVTALCYENQCSTVIFDEDDDGLGSIADISETERASQVESAEGVEKPG
RSETEVDVSQRNTIPLTLRMRAATATAVGAAAAHAQLLADQEEREVEYLVSTLVEAQMKKLQRKMKHVEALNLMMEKQHG
QMKDLEESLVAERMDVLQKMFSAGVSRWKDHAPVKSQSSSV

>CcSWI3B |Locus_ID=BC332_13234 |Protein_ID=PHU17539.1 |Protein_len=511 |
chr05:67777395-67780617 |strand= -
MAESGEINPPAPKPNPETTSPPPPQTEPQTPLLPHPPPPPPQPGSSQAKRPQQPESSPNPTTPSATESKDKDVTPTTAIS
SRLAEPEFVYIPSYSRWFSWNSIHECEVRFLPEFFDGRSPSKNPKTYKYCRNTIIRRFRDNPTKKITFTEVRKTIVGDVG
SIRKVFDFLETWGLINYTANTSKSLIKWEEKESKSASASASHNADANGTSTDCTVPKKRVCSSCKTICRIACFASDKYDL
TLCARCYVSGDNRVGVNNSDFRRVEISEEAKTDWTDKETLLLLEALLHYGDDWKKVAAHVGGRSTKECVARFVKLPFGEQ
FMGPLESTEVDSEPKSETMHPQSKRMRLTPLADASNPIMAQAAFLSALAGKEVAELAAHAAVTALSEIGAGITKGNLGSL
LGGAEGQESDGTSNGYGKDIERAIVEARPQLEKEALDLERAVSDVAIETKEIEERIAHFEERDLQMEKEWQQLMQLKNLI
FIDQLTLLLNKASAPKTGETIGEEVIDVKAE

>CcSWI3C |Locus_ID=BC332_15732 |Protein_ID=PHU14527.1 |Protein_len=767 |
chr06:172876490-172894856 |strand= -
MKNQNDEDGFEDEEDEEEIEQQEMVDDLQNPNNSVDRSYTNSVVELVSESKERISEFPMVVRRAVTRPHSSVLNIVAIEK
AGQSGESKQNGGVVLENLSYGQLQALSAVPVDHHALLSDERGEGSGSGSYVITPPQILPGRGVVKHYGSAGRIHVVPMHA
DWFSPNTVHRLERQVVPHFFSGKSAEHTPEKYMECRNCIVAKYMESPVKHLSVDDCHGIVAGISADDVTRIARFLDHWGI
INYCAVPPKDEAPKDGTYLYEDSNNDICVPVAGLKSIDSLVQFDKPKCRLKAKDIYPELVRDCDDDSDFDNSIREVLSEI
KCNCCSRPVSLAYYQSQKEIDILLCLDCFHEGRFVAGHSSLDFVKVSSMKDYGDLDGDSWTDQETLLLLEGMQRYKENWN

QIAEHVGTKSKAQCILHFVRLPLDGASLDNIELPGASGPSSSSTGEDPNKSHSILNGNLAGPSTENFHPDSKFPFENCGN
PVMSLVAFLASSVGPRVAAACAHASLAALSKDDTLGARRNMTQMDGSTANNGTSVGRMPGKNGSPHGDVGNSHQLKDEKS
GGQGPHDTGGAPLSTVSVRAAAKAGLAAAAIKSKLFADHEEREIQRLSANIVNHQLKRLELKLKQFAEVETLLMKECEQL
ERTRQRFTAERSRMISQPGSIRVSRPMGVSGAGAAVVSNAGNSRQQQVVSAPQQQGFVSGYGNQQQMHPQMSFLQQQGMY
GFGPRLPLSAIHPSSSTPGMFNASASSQPALSHSMLRPVSGTKSGLG

>CcSWI3D |Locus_ID=BC332_22454 |Protein_ID=PHU10594.1 |Protein_len=943 |
chr08:191213095-191227220 |strand= +
MEEKRKDTGTPPPPSADTPPISADAPSVEPPTSRRRGGGHKRKASAIGSGASSTPPSTLSKRQKQSAVPFPPIHNGPLTR
ARQQPNHAAAAAAAAAGSPSGFGFTIESEVFPKAEAGVEEAVRVEKESNQVKEDLEALEAEIEAEIESIGSRDRNVHIVP
THAGWFSWTKVHPLEKRTMPSFFNEKLQSRTPEIYMEIRNWIMKKYHTNPNIQIELNDLSELSTGDLDVKQEVMEFLDYW
GLINYHPFPQTNSVMNVDIDGDKAARTDSLIDKLFRFESDETWTPVLPRSSVATPSVSSGFFPESAIAEELKSEGPAVEY
HCNSCSADCSRKRYHCQKEADFDLCSECFNNGKFGSGMSPSDFIVMEPGEVGSASGGKWTDQETLLLLEALELYKENWNE
IAEHVATKTKAQCILHFIEMPIEDRFLDTDAESNKLVKEKEDAVLSKDDTSASKDAPETTETKDDGNDDQLSSTVETSKP
ENVNGPIPQEEVGENCALNALRDAFTAVDFYPPPGERASFAEAGNPVMALAAFLVKLVEAKKVSASVRSSLKSISGNPSG
EQLALRHCFILEDPPDDGKTSSDSDRPANGSVDPEHKKDEDDNVEMQEEKLTSVIDEKSLSVGQDKETKGETNIDKKCEE
RDGENHEEKNEKELEVATHLVSTSDKSQEKSDTSKQSGAISTDKEGESASLKEPDDAGLAVGQTPSTTAESNVLTSKLEV
PPGFEKESVDEASMAIPSNSPDTPKDEDMMPTVQTKEPEQSMKSNIVLENGKNTGTVEVKDSVDGRKDPLKTKNDLDIDK
IKRAAVTALSAAAVKAKYLADQEEDQIRLLTTALIEKQLHKLESKITFFHDMDNVVMRVRELLERSKQRLVSERQQIINA
RRAGPQPVLANRPGMTLANSAPRLPTAMSSPRIPNSRPIMAGAPTPSSFMPTTVSGNSMQPSK

>CcRAP1 |Locus_ID=BC332_23251 |Protein_ID=PHU06762.1 |Protein_len=498 |
chr09:23718032-23741314 |strand= +
MNFDYIFNNNIAGDNEEPNREKKRGRKSSKEIQEEIHQTSVINQAVRRQTCTIWTDDLHAKFMEAIHQLGEGRCYPKEIL
EVMNVPGLTRTQISSHLQKCRSNSWRAPRKQKAICDSLSQASSSGTQQKNSFKNYGAMPRLPNLQCNLDQMERGSEFSFS
VPNINNIFARGKSLTQQQLYHPQPQVQPQAQNNVGGGLQQHGPSFGLLGSQGLQGPTIGNIIYKPGLTFNNSDHYTQSDY
NLNLNAAHDTTYSSSGIMSDTNIDAAINYYNLKVNLDNVTTYSRSSMISNTGVANSTINELGAANANFQKNIGESNEHDD
ELVQVHFDDQVRTLNFLTELRQDQLASISSSGKSSYFEDKFILYSVRMLSFPIELRKDQLASISRSGESVVANQIYKKCL
ITVLHKVMLADLIELDMVDFDLIIGMDCLQFYYASIDCCTYVVKFQFLDEPVFKWSEDLLEVPPDREIEFGIDLLPDTQP
TSIPPYYMAPTELKDLKE

>CcAPL1 |Locus_ID=BC332_07646 |Protein_ID=PHU22539.1 |Protein_len=382 |
chr03:124758023-124764010 |strand= -
MFHAKKPSTMNSHDRPMCVQGDSGLVLTTDPKPRLRWTVELHERFVDAVTQLGGPDKATPKTIMRVMGVKGLTLYHLKSH
LQKFRLGKQPHKEFNDHSVKDGDRATSLELQRNSASSSGMIGRNMNEMQMEVQRRLHEQLEVQRHLQLRIEAQGKYMQTI
LEKACQTLGGEENMALPARTFKGIGNQGGLIPDISAAFKEFGTPPLSFSSFQDLNICGGHDQQLELHAQSSMGERSSSFD
GFMNLGNTTDHNLTLGKKRPSPYSSNGGNSNGKSPFMWSDDFRLQELGVATAAALGPNNEDDHQIIQMERSCNPELDSVS
DLYESKPILQEEKKCDAKLERPSPRRAAQISSLSAQGGRSSVFGELLSWQDGKVTDGGERRE

>CcAPL2 |Locus_ID=BC332_15958 |Protein_ID=PHU14753.1 |Protein_len=233 |
chr06:195054353-195056773 |strand= +
MYENDGVVMTRDPKPRLRWTAELHDQFVDAVTKLGGPDKATPKSVLRLMGLKGLTLYHLKSHLQKYRLGQLAKKQNAAEA
NKENCGDSGQFSLHSSGPSTSSLSMNFMQGEVVPVADAVMCQIEVQKRLQEQLQVQQKLQMRIEAQGKYLQAILDKAQKK
LPNMNSPSAPEATTAQLTAFSSPLSNLLDYTVHGENRDNNIELLPTPNHIVEKEQKNDMNSINFDLNSRSSFV

>CcAPL3 |Locus_ID=BC332_00901 |Protein_ID=PHU28808.1 |Protein_len=152 |
chr01:23679239-23682102 |strand= -
MDRMYNGGGDGYENGVVMTRDPKPRLRWTADLHDRFVDAVTKLGGPDKATPKSVLRLMGLKGLTLYHLKSHLQKYRLGQQ
AKKQNAAEQNRENIAAESFGQFSLHSSGPSITSSSMNGMQGEAPISEQLWCQFEVQKRLQEQLEVQQKLQMR

>CcAPL4 |Locus_ID=BC332_31366 |Protein_ID=PHU01579.1 |Protein_len=291 |
chr12:222554639-222561207 |strand= -
MYSGLGMERNGGMGEYHNHHFHHNLQSSITGEMNNLVGDACLVLTADHRPRLRWTAELHERFVDAVTQLGGPDKATPKTI
MKAMGVKGLTLYHLKSHLQKYRIGKQSKEAAENYKDESCMAESQDTSSTASGSSKVVAQDINDCGYQVTEACRVQMEVQR
RLQEQLEVQHHVQLRIEAQGKYLQTILEKACKALNYTALESPELDTAREQLSELAIKGANANNCDGIVAVSSLPEVVTSF
ENQNASDMPATSLVSPTSIGVLKKRPRPLANGDALPVEDNMTPVQWMMTNS

>CcAPL5 |Locus_ID=BC332_06463 |Protein_ID=PHU21356.1 |Protein_len=292 |
chr03:5912615-5918150 |strand= +
MYSTLPIRSLGIEESGDFFNLQGCLSVDGTNLPGDSCVVLSTDPKPRLRWTTELHERFVDAVTQLGGPDKATPKTIMRTM
GVKGLTLYHLKSHLQKYRMGKQSAKEGTENSKDVSCPAESQDTGSSTSGSSRVIAQDINEGLQVTEALRVQMEVQRRLHE
QLEVQRRLQFRIEAQGKYLQSILEKACKAFNSQSLESNGLEMNGEDLPALATKVANDCEGIITVPSLPEIGSSIENRNAC

HLPARLGDCMLDDCLPSNGIGALKKRPRAFTNANGLPMESNTREVEWMMSNI

>CcAPL6 |Locus_ID=BC332_12550 |Protein_ID=PHU16855.1 |Protein_len=323 |
chr05:6131998-6137151 |strand= -
MYPRLIQSGQEDEYIQHHGCDVNVVGIHHQRVNGGDPCLVLTSDPKPRLRWTADLHERFVDAVTQLGGPSKATPKAIMRT
MGVKGLTLFHLKSHLQKYRLGKQSQKDLDEASKDGLTATYSLESPCSGGTPQQLPASDLNEGFEVKEALRAQMEVQSKLH
LQVEAEKHLQIRQDAEQRYITMLEKACKMLADQFIGDVVTENHQETYQGLGTKTQLSPLCNPNGLCPSDSADLIGVHGPE
EVSPRIHPQRTDCSTESCLTSHESPAGLPLEGSSPGGKKRGLSGDSTHASYVWGEADMRSSGVHAIQVNCFGITGSNVQN
VSN

>CcDIV1 |Locus_ID=BC332_32995 |Protein_ID=PHT98081.1 |Protein_len=256 |
scaffolds:171231-173554 |strand= -
MWIESYYQNTSLNSSAKTLSLFYIQTKKKITKSTMMYTTNNRWMIGDAANNSTAMIQSNSTWTRFEDKQFEQALVLYSEN
DVERWQKIANHVPGRTVEDVMMHYDTLVHDVFEIDSGRVEPPSYPDDDGGFGGDWDELDGGKLSQISFGGGASKKQEVER
KKGTPWTEEEHRLFLIGLDKYGKGDWRSISRNVVVTRTPTQVASHAQKYYLRQQSMKKERKRSSIHDITTAVDTKMVPPQ
NSLQNQGAYQNFNFPM

>CcDIV10-1 |Locus_ID=BC332_15633 |Protein_ID=PHU14428.1 |Protein_len=398 |
chr06:157986961-157989623 |strand= -
MSTDQICNSTFWTKEEEKVFETTLAIYFKGGDLLTKMEEALPCKSRDDIINHYKILIEDVDAIESGRVPLPNYPELPSHS
NQKSRFPKTNLARRKGVAWTVEEHRSFLRGLAIYGRGDWRSIARNCVITRTSMQVASHAQKYFNRLKAVNKENRRMSIHD
ITIVDAEVAGTSQVPYTEGMIWPACGGSQAVACTSNQNMLPPESTNAEQMIAVAGGESSGHTAAFGSGMNSMSDEDDFIL
HIDDLIVDPEDANESEILVDGGKSLLPSKQPCTAGSSGSNNHPIFRVGSELGALITKQMDEDYDFFSIFDDGDAPISHAM
VSNAAHSGMTSFAVASNSANNPPQNTVSAHSGRTSFAAASTGPNKAPLNMVSDHPQLPHVAPSSNCVVGEWLQFKYYH

>CcDIV10-2 |Locus_ID=BC332_15631 |Protein_ID=PHU14426.1 |Protein_len=400 |
chr06:157957125-157959752 |strand= -
MSTDRTCNSSFWTKEEDKIFETTLAIYFKGGDLLTKMEEALPWKSSDDIINHYKILIEDVEAIEFGRVPLPNYPELPSHS
NQKNRSSKTDLAWRKGVAWTVEEHRSFLRGLAIYGRGDWRSISRHCVITRTSTQVASHAQKYFNHLKAVNKENRRMSIHD
VTIVGADIAGTSQVPYTEGMILPACGGSQVVASTSNQSLLPQESTNAEQMLAVVGGESSGHSAAFVSGMNSMDPDEDDEF
IKHIDDLIVDPEDANESEFLVDGGRSLLASKQPCTAGSSGTNNHPIFIVGSDLEALITKQMNEDYDFTFILDDGEAPISH
AMLSNASHSGMASFAVASNNAYNPPQNTVSAHSRMTSFAAASIGPNNAPQNMVAAHPQSPHVAPSSNCVVGEWPQFKYYN

>CcDIV11 |Locus_ID=BC332_16553 |Protein_ID=PHU15348.1 |Protein_len=126 |
chr06:220155838-220156795 |strand= -
MVPNKTIDELKQHYQLLVDDVTAIEYGYVPIPNYTVEENSYGYSRVSRDDWQSNGNEFAETAQCPERRKGTPWTKEEHRL
FLLGLERYGKGDWRSISRNIVISRTPSQVASHAQKYFNRLKKNQHP

>CcDIV13-1 |Locus_ID=BC332_23075 |Protein_ID=PHU06586.1 |Protein_len=348 |
chr09:10863883-10868697 |strand= -
MVAHRGEPLEIDNWELGLQRDAGLSRRSTRIGQLSFWHPPRHGNRRDSLEITEGREEEEQIDTVYVDLAAERKVDIKGPK
DDTKSTFHSNTGSEIEHLSVGLSDYEGEGFDFDYEKYITDNTCLAAVDSVYQPDIYGCFCDIMNLIRFLNPLQELTLFML
EPFLVCQKEMRTDRTCNSSFWTLRRMEEELPGKSGDDIINYYNILLEDVEAIDSGRVPIPNYPELQSISNQNSEADVERQ
RGASWTELEHRSFLRGLDKYGRGDWKSISRYCVITRTPTQVASHSQKYFKHLKAVPKKNRRASILDITSADAEAAGTSQA
MPSTNNESIFLRQSTNAEQIIAVAGGVA

>CcDIV13-2 |Locus_ID=BC332_23074 |Protein_ID=PHU06585.1 |Protein_len=429 |
chr09:10846220-10847970 |strand= -
MLKLIRFLNPLQELSLFMFESFLVDPKKMSTDRTCNSSFWTKDEDKVLENTLAIYSDDKDLFTKMEEALPGKSRDDIINH
YNILIEDVEAIDSGRVPLPNYLELQSDSNQNSRADVEWRRGASWTELEHRSFLRGLDKYGRGDWKSISRNCVITRTPTQV
ASHAQKYFKRLQAVPKEKRRASILNITSADAEAAGTSQAVPSTKNEIMLPRESINAEQTIAVAEGESSGHDAVFVNGMDS
LNPDRNGEFIFSIDDIIMEEEDVNETGFLVDAGRSLLPREQPCAAASTGAYGHPITRIGSDLEALLTEPREEDNDINSIF
VVRKAPTSHEVLLNAAHSGISSYAAASFGAENAPESNVAAYSGMSSYADASSDAVNAPDNMVAAHSGMTNFAVTSRFASN
APQDTVAAHASQLPPFSLSSNFDVGDAPM

>CcDIV14 |Locus_ID=BC332_14807 |Protein_ID=PHU13602.1 |Protein_len=218 |
chr06:9374951-9376248 |strand= +
MENSPENNDVFIVSDDDGHDQQHHLVQQEDNHRPCRRRGTPWTKNEHQLFLMGLNRFKRGDWKSISRYYVVSKTPTQVAS
HAQKYFSRQDSKTPVERRRPSINDIQTVSLNLKTTSITTMGATYLINNNRFAYNNNHQAASSSIHFHYPSYTFSGPTFSN
NFNENFEISHLGETSGSNSSVMGKNNNNNKFNNLNRPVSPRPFLSTYLSSIRRNDPQI

>CcDIV2 |Locus_ID=BC332_07426 |Protein_ID=PHU22319.1 |Protein_len=292 |

chr03:73180846-73182030 |strand= +
METLFSSNSFLPNSSWMSKSIEWTKEENKQFESAIAIYDENTPNRWFKVAAHIPGKTVIDVMNQYKELVADVCDIEAGLV
PNPGYFGSSITLEFVDHCGLQTFRKRGSRSSDQERKKGVPWTEEEHRRFLMGLEKYGKGDWRNISKKMVISRTPTQVASH
AQKYYQRQISGGKDKRRPSIHDITTVHFTTDATSQNNLAYNINSLSKEKFYATSSSFQKFTTTTDIVNCWNASLLSEHVD
LMDFGSLYGSSPFVAYPYKIASQYWDEHGANSITLSGNPQLQLQSTRYQIWD

>CcDIV3 |Locus_ID=BC332_13887 |Protein_ID=PHU18192.1 |Protein_len=242 |
chr05:217772836-217773933 |strand= -
METLCPTSFVPNSNWTMQQSKSTTEWTKEENKKFESALAIYDETTPNRWSKVAEIIPGKSVLDVMMQYKELVADVSDIEA
GLVPNPRRFVSSFALEFADHRKIHTFSKRGKSYDQERKKGVPWTEEEHRRFLMGLEKYGKGDWRNISRNFVISKTPTQVA
SHAQKYYLRQLSGEKERKRPSIHDITTVNLTNNELLNNNNKLMCEVKSYMSPLQRSTSPADVLIGCWNNSFDGDLMAFGQ
FV

>CcDIV4 |Locus_ID=BC332_23536 |Protein_ID=PHU07047.1 |Protein_len=293 |
chr09:70770343-70772002 |strand= -
MNSEMGFLSPATYIKNSSWLFEENKGTRWTPEENKVFEEALALFDKDTPDKWYNVAAMIPGKTVNDVIKQYRELVDDISD
IEAGLIPVPGYTTSNSFTLEWVNNQDGFHGFKQFYGPSGKRGSSNRSSEHERKKGVPWTEEEHRQFLLGLKKYGKGDWRN
ISRNFVTTRTPTQVASHAQKYFIRQLSGGKDKRRSSIHDITVVNLTESKSTSPEDHKSAPTPQAQQHSQMNGMVKTLCEY
NSPNQESDITFNSSSGGGLITMPFRGAPSYELEHGMHNIALQGLQFGNYNTMC

>CcDIV5 |Locus_ID=BC332_17375 |Protein_ID=PHU16170.1 |Protein_len=306 |
chr06:244172554-244173998 |strand= -
MEILSPAPYLANPSWFLDEGKSTKWTPAENKAFENALALFDKDTPDRWQRVAEMVPGKSVGDVIRQYKELEDDVSRIEAG
LITIPGYSTSPFTLEWGNSHSFDGYKQTYVVGGKRPLSSRPPEQERKKGIPWTEEEHKLFLMGLKKYGKGDWRNISRNFV
VTRTPTQVASHAQKYFIRQLSGGKDKRRASIHDITTINLHDNQTSSPDYQKPTSPDQPAVISQQPKSAANAVHKMPFQWN
QANNGTAMGFNSAQGSLFMSPLYGVNSYGNNKMQSQSLQRGALHEPYFGSQSMNFQLQSAHQYHQA

>CcDIV6-1 |Locus_ID=BC332_28756 |Protein_ID=PHU03505.1 |Protein_len=361 |
chr11:235909003-235910919 |strand= +
MSLDRTCKRSSWTKEENKAFENALADYSGDRDLIQQIAAAVPGKSLEEVIEHYLVLVEDIKDIESGIVPLPKYRRMQSST
ERRKGIPWTAEEHRLFLQGLAKYGKGDWRAISRNFVLSRTPTQVASHAQKFYTRLNDKNNPKKRRSIHDVTSAANITEPS
QGPCGGRLQWPITNYVTDAFNTGMLSIPEPVTTRISTDAIEGASAADLEKIPLGAAVGNELNSSFPGMVEFVQSIEGHII
VPAEVPSGVCHGVDTKTSPSHSLQPSIAVGSGMYIPSFNNVRYDLTALMSKQLFGSSQVGPTVNSASLPLPTIDRIGVQD
CIASPSIPGSSSGGTYPCWNPSNIDDNTFDLVNLFPDGLGK

>CcDIV6-2 |Locus_ID=BC332_28750 |Protein_ID=PHU03499.1 |Protein_len=192 |
chr11:235389942-235391479 |strand= +
MSLDRTYKSSTLTKEEDKAFENALADYSGDGDLIQQIAAAVPGNLLQKFSNIILLFLQGLEKYGKGDWRDISRSFGLSRT
PTQVASHAQKFYTRLNDNNNPKKRRSIHDVTSAADIIEPSQGPCGGQLQRPITNYVTDAFDTGMLSIPEPVTTRISTDAI
EGASADDLEKIPLGAAVGNELNSSFPGMVEYV

>CcDIV7 |Locus_ID=BC332_14309 |Protein_ID=PHU18614.1 |Protein_len=189 |
chr05:233225693-233228706 |strand= +
MSIWSRNDDKLFENALVLYPENIADRFQLIADYVPGKSIDDIMAHYDDLVHDVFEIDSGRIEFPGYTDDDSGLEGHSGRV
ELEGDSGRVSAERKKGTPWTEEEHRLFLIGLEKYGKGDWRSISRNVVVSRTPTQVASHAQKYFIRQQAMKKERKRSSIHD
ITTAVDTNTVPPQSSLQNQRGTQNFNFAQ

>CcDIV8 |Locus_ID=BC332_25861 |Protein_ID=PHU05039.1 |Protein_len=282 |
chr10:167370402-167371420 |strand= -
MNSEMELFSPPQATYLRNSNWLFEENKGTKWTPEENKMFEDALALFDKETPDRWYYVASMIPGKTVDDVMKQYRELLEDI
TDIEAGLIPIPGYTTSSNSFTLEWINQDHEEGIFHDFNKQFYGPSGKRSTSNRYSEHERKKGVPWTEEEHRQFLLGLKKY
GKGDWRNISRNFVTTRTPTQVASHAQKYFIRQLSGGKDKRRSSIHDITTVNLTEMKSPSPENHISSSSPDKSVPSPQKAL
CEYNFPNQEAVIGFKTYGLKLHEHGMHSVILQGLQFGQYNTM

>CcDIV9 |Locus_ID=BC332_09843 |Protein_ID=PHU24736.1 |Protein_len=290 |
chr03:273459958-273460973 |strand= -
MSPSDWSREEEKAFENAIAMHWVENCEQQWEKFGLMVPTKTIDELKQHYQLLVDDVTAIEAGQVPIPNYKGEEASSSSTK
EGNLGYSAGSVDRRSNCGYSNGFSGTNHDPIGHGGKGNSRSEQERRKGIPWTEEEHRLFLLGLEKFGKGDWRSISRNFVI
SRTPTQVASHAQKYFIRLNSMNRDRRRSSIHDITSINNGDVSTHQAPITGQQVNTNPSNAAALGPSVKHRTQPNMHGMGM
YGAPMGHPVAAPSSHMASAVGTPVMMPHGHPPYVLPVAYPIPPPPPPMHQ

>CcEFM1 |Locus_ID=BC332_24443 |Protein_ID=PHU07954.1 |Protein_len=440 |

chr09:249856060-249859054 |strand= +
MNMTSSNSELSLECKPHQSYSMLLKSFGEKIDQTQNLEEFLARLEEERVKIDAFKRELPLCMQLLTNAMEASRQQLQSHR
ENNHIGHRPILEEFIPLKNNNNASVELGAEKIVANNTSTIVDNKANWMTSAQLWSPPSDHQETKQIQTSNNTSNKENDHH
IGFSISPKLALDNNSSCPSPTTLALASSNKQNESTIVEDHNKNLEGVNIQQDSQSIGGGTNSTSTSTQQQQPHRKARRCW
SPDLHRRFVNALQMLGGSQVATPKQIRELMKVDGLTNDEVKSHLQKYRLHTRRPSPSPQATTAPPHLVVLGGIWVPPEYA
AAAATHGGAPPAATFYGPHSTSHAPSPHYIAAPQAVTQEFYSTPQPLQSLHHLQHHQQMYHHGSHMYKPPSKSHSHSSPE
SDARGTGQHGDRSSESIEDGKSESCSSENGGERKVVVHKF

>CcEFM2 |Locus_ID=BC332_27315 |Protein_ID=PHU02064.1 |Protein_len=402 |
chr11:6571024-6574499 |strand= +
MMINNNNFNEKMQRCQQYIDALEEERRKIQVFSRELPLCLELVTQAIETYKQQLSGTTTEYNLNAQSDECTDDDEHTSSD
VPILEEFIPLKRTFSHEYEDQEDAHDEENESHKSKNNNSNNSTISKDNKSNTKSDWLRSVQLWNQTSDPTLKEVINFYYF
LTFNLFLFFLFFFIFKISRNIFFCDYPILGIYLSMYVIIQLSLALVRLEENKEEKDGQRKQRRCWSPELHRRFLQALQQL
GGSHVATPKQIRELMKVDGLTNDEVKSHLQKYRLHTRRPSPSSIHNNNNQQPPQFVVVGGIWVPPPDYASMAAVAPAASG
EASGVANSNGIYAPIATHAKGPLHDHASLGGTLKQLRQQHNEKPSRSSERDGSHSHSEGGGVHSSSPATSSSTHTTTASP
AY

>CcEFM3 |Locus_ID=BC332_22367 |Protein_ID=PHU10507.1 |Protein_len=407 |
chr08:189597824-189600185 |strand= -
MGSIPPELSLDCRPSFVPESVTDFLRKVSLIRHIPDKLAQIDDYISRLDDEMRKIEAFKRELPLSVLLVKDAIGTLREES
MQYRKSRKEPVLEEFIPLKKSLDEDTKAEITAKDKDSREKMSWMSSVQLWNSESHCEKTDEVNNKQPSKSELKVDTRKSL
EEEGHSSITEDPFRSYKTMNVGKAFSPFKGFSVAAVRKDNKDELPGVTGLSLHTPGIAKLREDTVTSGLNSKHNGSRGGL
SSAAGCQSNIRTGSLAQQQSARKQRRCWSPELHRRFVDALQQLGGSQVATPKQIRELMQVDGLTNDEVKSHLQKYRLHTR
RVPNSQTQPANKSGVALGNLWMSQGQYVESSKRSSSQSGSPPGPLHLAVSCGGTSTTVGDSMEEEDDLKSENHSWKNHVH
TSGKIDV

>CcETC3 |Locus_ID=BC332_24253 |Protein_ID=PHU07764.1 |Protein_len=86 |
chr09:236821040-236823376 |strand= +
MQGKAAYDTHLCGVPFPVQKESLGVTAFEANNEETSKLEFSEDEEFLVTKMFNLVGERWSLIAGRIPGRTAEEIEKYWNS
RNSTSQ

>CcGLK1-1 |Locus_ID=BC332_34438 |Protein_ID=PHT96636.1 |Protein_len=447 |
scaffolds:1797-5550 |strand= -
MLAVSPLSNTTARDDNTMESFAIGGGVDFPDFVGENLLDSIDFDDLFVGINDGEVLPDLEMDTEILAEFSVSSGDESDVN
NYSSSNKTTFISTATKNVERKDETERAASDVGSGLTSLNQGEEIVSTQKSEESTLQVKQNITPKESDKGKKSSKNNLPGK
RKVKVDWTPELHRRFVQAVEQLGVDKAVPSRILEIMGIDCLTRHNIASHLQKYRSHRKHLLAREAEAASWSQRRQLYGGA
AVVGGGGGKRDMTPWPAPTIGFPPPPTMAAPMPHHFRPLHVWGHPSVDQSYMHMWPKHLAPSPSPQHPSPAWAPPPHLHP
PPPSDPSFWHPHHQRVPNPLTPGTPYFPAPIAPTRYPGGHHPVPGIPPAAHAMYKVDHVRSTAPPTQPLPKPPCDFHPSK
ESIDAAIGDVLSKPWLPLPLGLKPPAVDSVLGELQRQGVPKIPPTCA

>CcGLK1-2 |Locus_ID=BC332_34411 |Protein_ID=PHT96663.1 |Protein_len=447 |
scaffolds:2358-6111 |strand= -
MLAVSPLSNTTARDDNTMESFAIGGGVDFPDFVGENLLDSIDFDDLFVGINDGEVLPDLEMDTEILAEFSVSSGDESDVN
NYSSSNKTTFISTATKNVERKDETERAASDVGSGLTSLNQGEEIVSTQKSEESTLQVKQNITPKESDKGKKSSKNNLPGK
RKVKVDWTPELHRRFVQAVEQLGVDKAVPSRILEIMGIDCLTRHNIASHLQKYRSHRKHLLAREAEAASWSQRRQLYGGA
AVVGGGGGKRDMTPWPAPTIGFPPPPTMAAPMPHHFRPLHVWGHPSVDQSYMHMWPKHLAPSPSPQHPSPAWAPPPHLHP
PPPSDPSFWHPHHQRVPNPLTPGTPYFPAPIAPTRYPGGHHPVPGIPPAAHAMYKVDHVRSTAPPTQPLPKPPCDFHPSK
ESIDAAIGDVLSKPWLPLPLGLKPPAVDSVLGELQRQGVPKIPPTCA

>CcGLK1-3 |Locus_ID=BC332_34315 |Protein_ID=PHT96759.1 |Protein_len=447 |
scaffolds:1284-5037 |strand= +
MLAVSPLSNTTARDDNTMESFAIGGGVDFPDFVGENLLDSIDFDDLFVGINDGEVLPDLEMDTEILAEFSVSSGDESDVN
NYSSSNKTTFISTATKNVERKDETERAASDVGSGLTSLNQGEEIVSTQKSEESTLQVKQNITPKESDKGKKSSKNNLPGK
RKVKVDWTPELHRRFVQAVEQLGVDKAVPSRILEIMGIDCLTRHNIASHLQKYRSHRKHLLAREAEAASWSQRRQLYGGA
AVVGGGGGKRDMTPWPAPTIGFPPPPTMAAPMPHHFRPLHVWGHPSVDQSYMHMWPKHLAPSPSPQHPSPAWAPPPHLHP
PPPSDPSFWHPHHQRVPNPLTPGTPYFPAPIAPTRYPGGHHPVPGIPPAAHAMYKVDHVRSTAPPTQPLPKPPCDFHPSK
ESIDAAIGDVLSKPWLPLPLGLKPPAVDSVLGELQRQGVPKIPPTCA

>CcGLK1-4 |Locus_ID=BC332_34226 |Protein_ID=PHT96848.1 |Protein_len=447 |
scaffolds:34599-38352 |strand= +
MLAVSPLSNTTARDDNTMESFAIGGGVDFPDFVGENLLDSIDFDDLFVGINDGEVLPDLEMDTEILAEFSVSSGDESDVN
NYSSSNKTTFISTATKNVERKDETERAASDVGSGLTSLNQGEEIVSTQKSEESTLQVKQNITPKESDKGKKSSKNNLPGK

RKVKVDWTPELHRRFVQAVEQLGVDKAVPSRILEIMGIDCLTRHNIASHLQKYRSHRKHLLAREAEAASWSQRRQLYGGA
AVVGGGGGKRDMTPWPAPTIGFPPPPTMAAPMPHHFRPLHVWGHPSVDQSYMHMWPKHLAPSPSPQHPSPAWAPPPHLHP
PPPSDPSFWHPHHQRVPNPLTPGTPYFPAPIAPTRYPGGHHPVPGIPPAAHAMYKVDHVRSTAPPTQPLPKPPCDFHPSK
ESIDAAIGDVLSKPWLPLPLGLKPPAVDSVLGELQRQGVPKIPPTCA

>CcGLK2 |Locus_ID=BC332_25081 |Protein_ID=PHU04259.1 |Protein_len=311 |
chr10:6978367-6984249 |strand= -
MLVVSTPLSYKNERGNYDLFQDFPDGNLIDTIDFDDFFEGINDGDLLQNLKILDEFDISKNNTTTNLNVKTKSKENDKSK
KSSSQIKNPEGKKKVKVDWTPELHRRFVKAVEKLGVDKAVPSRILELMATDGLTRHHIASHLQKYRAHRKHLLAREAEAA
SWTQRKQMYGGATAIGGGGKRVIMNPWSAPPTMGFPPMAHHIRPLHVWGHPYVNNSFWHPHYRVPNSLAPGTPCFPSPTR
FAAPLMVPGVPPPIASGQTPHLHLTKESIDAAIEDVLSKPQTPLPIGLKPPSIDSVLNELQCQGITKIPPT

>CcHHO1 |Locus_ID=BC332_05708 |Protein_ID=PHU27376.1 |Protein_len=449 |
chr02:161961278-161965535 |strand= -
MGSNSREMDIDLNFIYVPKIISDVLTEISVMDDISKKLIKLNQFIIALEEELRKIEAFKPIERLKEEALQYKGKDKKPVM
EKFISLKKGNSDESGRVKKSNDLNDKKNWMSSAQLWSTPVQYEQSYDLEKQGPFLHLNSGGVEEKNKEKEYQLGVHKLNS
DRGAFLPFQGQTLKDGKNGLPVKDLSLCMAVTVAEGQKGQNPIDLSVKNDNGTSNGCSFLQDKSQQRKQRRCWSPELHRR
FVDALHQLGGAQVATPKQIRDIMQVDGLTNDEVKSHLQKYRLHVRRVPVSGCSWSNTDEIGESSKTNGTQSGSPEGPLHF
TGSGKMTSDSQMQDAATSMGATNIATSSRTNAPPTMALTEKPEKFTGIDFKRWQQKMFFYLTTLCLQRFTSEDAPEVPEG
TSDKERFVIVEAWKHSNFLCRNYILSGLQDDLYNFYSGTKTSKELWGGT

>CcKAN1 |Locus_ID=BC332_00869 |Protein_ID=PHU28776.1 |Protein_len=290 |
chr01:22734254-22738844 |strand= -
MELFPAQPDLSLKISPPNIKPSSSSTNWKRKTTSSTEDENLDLGFWKRAMVESTNSMLNNNSNTSSCFELSLSNNPTKPN
NYSCDPNSSHHLQNYILQQNQRIITTRELGFLMQPIRGIPVYHQKPFDNTITTFPIPSSSNCSILNNINNNNTITSCAST
SPNFQQGGFLRSRFYSRFPGKRSTRAPRMRWTSTLHARFVHAVELLGGHERATPKSVLELMDVKDLTLAHVKSHLQMYRT
VKTTDRAASSGQSDTSEELIVETEEYLRKSEITIQQGRRNIHQNNDFQGL

>CcKAN2 |Locus_ID=BC332_15978 |Protein_ID=PHU14773.1 |Protein_len=418 |
chr06:195859709-195865647 |strand= +
MELFPAQPDLSLQISPPNSKASSTAWKSSMVTNNTNSTTNQDEMDLLFWKRALESRNSKSDNNSNNSSSLFELSLSNIPN
KPSPPHQLINTTSHFHPTIQNTHTNFIHQSLQQNQELSFLRPIRGIPVYHQNPPPLPSPSIPVSHHNYPIFAHQTFDNTS
TTAATLPIHSSGCCISSFNNNISNKTISNSSPIIPFHQSHHQAGLMRSRFLSRFPAKRTMRAPRMRWTSSLHARFVHAVE
LLGGHERATPKSVLELMDVKDLTLAHVKSHLQMYRTVKTTDRAAVPASSGQSEVFDNGSSGDNSEDLMPDMENSRKSADL
LSDQHAGRNNMHLQEKDYHGLWSNSSSRESWQLHGKHGDYPGNMAPLERTEKNQHKDMEANAKCLSYDRISGEVSSSSIT
ETSPKKPNLEFTLGRPSE

>CcKAN3 |Locus_ID=BC332_11999 |Protein_ID=PHU20848.1 |Protein_len=307 |
chr04:226904350-226907883 |strand= -
MRDTVLSMVPDLSLQISLPPCISKSDQFKEAIRFDSTTDSGSSASVGSDLSHENNGYFHLDKIKPFNLPLVPSYEPTLSL
GFGQVPTGTNHQYQHYQPQIYGRDFKRSSRMNISGVKRTARAPRMRWTSTLHAHFVHAVQLLGGHERATPKSVLELMNVK
DLTLAHVKSHLQMYRTVKSTDKATAKGQGENVFVNQHKGINNIGEYEKSGEEIDAVSQSSTMPQNMAHNMRDPSSFMPDQ
TNAWSHSNRERPFANYPHIYSNMGGIETANNSSSNDTLVNLEFTLGRPSWKMEQAGSSSNDLTLLKC

>CcKAN5 |Locus_ID=BC332_19106 |Protein_ID=PHU12176.1 |Protein_len=111 |
chr07:193411405-193412184 |strand= +
MPESSKKHKGETSEKVGPSHDLNRKPRFKWTVELHEHFVKAVNELGGPYEATPKNIVKLMDDEDITPDHIKSHLQIYQGR
SRSIKVQGVSCHPQVLIKARGALCHPQVSID

>CcKAN6 |Locus_ID=BC332_18917 |Protein_ID=PHU11987.1 |Protein_len=250 |
chr07:174661694-174670007 |strand= +
MMEYFSMMKKRSSNKIGVRQYKKSSVPRLRWTPELHELFIEAVQLLGGGHKATPKRIQQMMEVKGLTISHVKSHLQMYRN
LKERASPNIVSSVNNFLEERPQFSWSSPRHVCETLEHRPSKSSNQINPGSDQSPYKARVDAHSDQIAENNIYCCDYQEAQ
GSLSSGMTKEEEESGGPTSEICEFPEADGDYKRENNIWPLDDWVARCTKGSAEYSQSDSSTKFLDQQVLQSPKDNSHINL
DLSISSYFYC

>CcKUA1 |Locus_ID=BC332_00616 |Protein_ID=PHU28523.1 |Protein_len=343 |
chr01:12399830-12402577 |strand= -
MTRRCSHCSTNGHNSRTCPNRGVKLFGVRLTDGLIRKSASMGNLTHFGGGGGGGGGSSTGLNGVVHDSPGDTPDHPAVGG
AAADGYGSEDFVAGSSSSRERKKGVPWTEEEHRMFLLGLQKLGKGDWRGIARNYVITRTPTQVASHAQKYFIRQSNMSRR
KRRSSLFDIVADESGDTPMVSRDFLAEDPQAEMQSNNPLPPTPVVDEECESMGSAASANSIDGEHSLPIPESSQYPHPLV
YPAYVAPFFFPMPYPCWPGYTAEPAKAETHEVLKPTAVHSKSPINVDELVGMSKLSLGESIGNAKPPSLSLKLVEGSSRQS

AFHANPSSGSSGMNSSHSPIHAV

>CcKUA2 |Locus_ID=BC332_30669 |Protein_ID=PHU00882.1 |Protein_len=380 | chr12:150869443-150880452 |strand= +
MTRRCSHCNNNGHNSRTCPTRGGGGGATSSIGGGSAIGGGGVRLFGVRLTDGSIMKKSASMGNLSSLHYHSSSSAAASPN
HPGSPSSDVLREAVHLTDGYLSDDPVHASCSANRRIERKKGVPWTEEEHRLFLIGLQKLGKGDWRGISRNFVTSRTPTQV
ASHAQKYFIRQSSATRRKRRSSLFDIVADTAADASHPLPDEQFMLPPRAMESDKEQLAPSATKAIETDFADSLPSLDLSL
KSDFESMETVPSEPVQETKPNTATSEIPPAFPAFFPAYIPVPYPFWPSNAIPVPEDRGPEPSHHQILKPIPTVPKEPVNV
DELVGMSQLTLAEAGSGHFEPSPLSLKLTAEPSRQSAFHASTPGKSSEITKGETAPIQAL

>CcKUA3 |Locus_ID=BC332_24632 |Protein_ID=PHU08143.1 |Protein_len=240 | chr09:255398691-255401598 |strand= -
MGRKCSHCGYIGHNSRTCSTLKCAISGSNFIGGLKLFGVQLDTANSSSSSSSSSFSSHNLTKSFSLDCLSLTNSHIGHS
HLSSSLSPSPSINESCEKSTTSINNNGYLSDGIGVGCVGERKKGAPWTEDEHRKFLIGLEKLGKGDWRGISRNFVTTRTP
TQVASHAQKYFLRQSSINKKKRRSSLFDMARINNKFEDCQENSHWIYGSQNSHHNEVPNNKSISSNGNIDLELSLSNNIV

>CcKUA4 |Locus_ID=BC332_15336 |Protein_ID=PHU14131.1 |Protein_len=275 | chr06:105233532-105235753 |strand= +
MGSMTKDARKCSHCGHNSRTCNSKGVGGIKLFGVRIDDNYNSNSNNNNHGHGNIKIKMRKIGDYESIRRSKSLGNLEHAA
NYDNIGGVEAGYLSDGPIHSLRHRKKGTSWTEEEHRCFLIGLEKLRKGDWRGISKHYVPSRTPTQVASHAQKYFIRMSSI
DKKKRRPSVFDVYLKDLNPQIPSKSSPSVSNMVSEVSKDASSRVVQLFDTCQIKGQASKGVPCATRERPPLSPSPISRPF
GVRNLRHMPYMMGAPPNIHSVTPAKAGPTISWILP

>CcMYB1R-1 |Locus_ID=BC332_32578 |Protein_ID=PHT98499.1 |Protein_len=281 | scaffolds:544828-545986 |strand= -
MTITYGHSSFISAGELPPADVSGGEIMLFGVRVKLDPMRKSVSLNNLSQYEQPNSNDNNNSNGGNTNDSSSKVADEGYAS
ADDAVPHHSGSGRERKRGVPWTEGEHKLFLLGLQKVGKGDWRGISRNFVKTRTPTQVASHAQKYFLRRTNLNRRRRRSSL
FDITTDSVSVLPTEEAKNLQEAHVETSKINAFQVTSVPIKFAPLVLPAQMDKPIESPIQSYNASSIYLRPVPVVVPVSNQ
SSAIESSSLSLTLSLSSPSSSSTRYQVMSNFSNGESIISVA

>CcMYB1R-2 |Locus_ID=BC332_16646 |Protein_ID=PHU15441.1 |Protein_len=295 | chr06:222007861-222009975 |strand= +
MSSACGDESSATEVTGAGEIMLFGVRVKVDPMRKSVSLNNLSQYEQPNINNSGGDSKNESLKVAADEGYASADDAVQHQS
NCGRERKRGVPWTEEEHKLFLLGLQKVGKGDWRGISRNFVKTRTPTQVASHAQKYFLRRSNLNRRRRRSSLFDITTDSVS
AMPKVEGENKQEIPVLAPATLPTVETTKTNAFPVAPTVGPIMIPAQIDMSRDSPTLLRRNHGNSSMLVGAVPMFSLPNPS
PVIDLNANQNTTIEPSPLSLRLSLSLDQGQASSTRHLGYQVMSSFSNGESIISVA

>CcMYBC1 |Locus_ID=BC332_26707 |Protein_ID=PHU05885.1 |Protein_len=274 | chr10:222673866-222674690 |strand= -
MREEDSNWFAKWEEELPSPKELMPLSQTLITPSLAIAFHIQNPNTPNPRMSPLVHTPPSAEFESNSAELGGRAGSSGVGP
DEPARTPKRARLVWTPQLHKRFVDAVAQLGIKNAVPKTIMQLMSVDGLTRENVASHLQKYRLYLKRMQGFSSSSMSGAGV
DPATDHLFASSPVPAHFLHPGRGNSEHYMPFVPVAAIVGHAPQLQQQHRHLGPSPNGQFEVPFLSRQSQQQPVQRMGTSV
HSSSPVLPSYVEGLESATTANGRKVLTLFPTGDD

>CcMYBC2 |Locus_ID=BC332_27055 |Protein_ID=PHU06233.1 |Protein_len=290 | chr10:233421662-233422534 |strand= -
MREEDSNWFAKWEEELPKPEELMPLSQTLITPDLAIAFDIPNPTSPNPQSKQQQTPLVPSSQPNSSAEFESAELNGTGGG
DEPARTLKRPRLVWTPQLHKRFVDAVAHLGIKNAVPKTIMQLMSVDGLTRENVASHLQKYRLYLKRMQGLSNSGSGGSNA
QSLSGAGVDPAMDNLFASSPVPAHFLHPGRGNSDHFMPFVPVAPMQHHHHMGVVVGHHPQVQQQYRQFGSPANGHFEHPF
LSRQSQQQVQRMGTSVHNGSPVMSSYVEDVDSATAANGRKVLTLFPTGED

>CcMYBR1 |Locus_ID=BC332_13048 |Protein_ID=PHU17353.1 |Protein_len=300 | chr05:33253116-33257698 |strand= +
MGNCGRNGGVRQYIRSKVPRLRWTPDLHHCFVHAIEKLGGQDKATPKLVLQMMDVRGLTISHVKSHLQMYRSMKNDVNRQ
AERMSTQQRKHQSLEDHDCHYQKQEGVCVEQQKLLVYQHYPSFSSSPMERSDSTQYFNSILPTKRARIEVCSNQRIREAV
SNQYNSRNDYIEKNGVKGERAGFIWQQNSETNRQTAHPLCNSSSTTTLFFNPLKRSNEIQESEFFKVTKTQDSNAGGGGG
GEDNGECGLSLSLSLHRPSTQRSNASSIISEIGETISSYSAGSNVNQQSVSVDLNLSIAL

>CcMYBR10 |Locus_ID=BC332_32618 |Protein_ID=PHT98434.1 |Protein_len=628 | scaffolds:119352-121238 |strand= -
MDQGDEKEDMLRKLEKKSKKSNKNAATSGLGGESDGCEVKKKKKRKGGEHPGKCNKDNDEDVVKYIERKKNNKLKKSCLN
EPPLDFKISAPASASEMRDSEKVVETLGEISSGDVVDEIRRKKKSKKNIKTTNVVGVANTDIEAKTDDCDLKRKKEKKHG

KHSEKFNEDSDEVALRIVEGKKSRKLKKSRVAEQEKMHDTEDVQEDIVAEVDEGDISSPIKIEEKNRTDNGNIKKRKKVK
SGHSSEDPTHEIEEKSRTDNGNIKKRKKVKSGHSSEDPTHEIEEENRTDNGNIKKKKKVKSGHSSEDPTHEKSAKKVRFS
GHVQIFPSSDDPSDEKHEIEDENLLRGKRFSKLEDEIVKEAVHKYVDFHNLGEEGLKKILNSRSYPEIRGCWKEIGSAIP
YRPYTAVYYRAQILFRRSESRKWTEEEYEIVRKFQKEHGNNWKVLADELGKHRFHVKDTWRRIKRPNQNKGQWTQEEYQS
LFDLVNTDLRLRLSEEKRSKYGMLRDNIAWTAISDKLSTRTEANCCLKWYDQLTSPMVAKGEWADVDDYRLVDALFELDA
SCIEDVDWDNLLDHRPGEICRKRWNQMVLHIGKLKNSSFAEQIEVLAKRYRPDFAEVREAWDNKPVVP

>CcMYBR11 |Locus_ID=BC332_32616 |Protein_ID=PHT98432.1 |Protein_len=580 |
scaffolds:98923-104063 |strand= -
MERYLFKRRADEKERKKHGKHSEKFSEDCDEVALRIVEGKKSRKLKKSRVAEQEKMHDTEDVQEDIVAEVDEGDISSPIK
IEEKNRTDNGNIKKRKKVKSGHSSEDPTHEIEEKNRTDNGNIKKRKKVKSGHSSEDPTHEIEEXXXXXXXXXXXXXXXXX
XXXXXXXXXEEGLKKILNSRSYPEIRGCWKEIGSAIPYRPYTAVYYRAQILFRRSESRKWTEEEYEIVRKFQKEHGNNWK
VLADELGKHRFHVKDTWRRIKRPNQNKGQWTQEEYQSLFDLVNTDLRLRLSEEKRSKYGMLRDNIAWTAISDKLSTRTEA
NCCLKWYDQLTSPMVAKGEWADVDDYRLVDALFELDASCIEDVDWDNLLDHRPGEICRKRWNQMVLHIGKLKNSSFAEQI
EVLAKRYRPDFAEFSEDCDEVALRIVEGKKSRKLKKSRVAEQEKMHDTEDVQEDIVAEVDEGDISSPIKIEEKNRTDNGN
IKKRKKVKSGHSSEDPTHEIEEKNRTDNGNIKKRKKVKSGHSSEDPTHEIEEENRXQEQKHTIHWKNSRNMRNRRGILAD
LDQGKLSLKRNIRRAPDLVF

>CcMYBR12 |Locus_ID=BC332_30379 |Protein_ID=PHU00592.1 |Protein_len=1674 |
chr12:75409421-75417817 |strand= -
MPPEPLPWDRKDFFKERRHDRSEVLPRWREPASHHHYTSSRWLPDFRSRAPPPGHGKQGSWQMYPEELGHGFMTSRSNEK
IVEDESCRQSRGDAGKYGRNSSSSRENRSFGQRDWRGGGGAHSWEAASPSGSGSVRQNDATNNQRSLDVMEPHSEHVNTC
DQSHSRDQHNKSATANATASTGQRFERETSLGSIEWRPLKWTRSGSLSSRGSLSHSGSMGVDSNETKPELRLGNSKAVQS
LTGDATACVTSAAPSEETTSRKKPRLGWGEGLAKYEKKKVEGPEDNAVKVGTSISGDSVEPSHSQLLNQADRSPRVAVFP
DCQSPATPSSVACSSSPGLEDKQLVKATNIDQDVGNLCGSPNVVSQYYAEGSGFNLENWDLAQISNLNSSINKLLQSEDP
SSVDSGFMRSTAVNKLLVWKSDITKALEKTEAEIDSLENELKTLISGPKNTQLVPSASCSPPKDCNANSHEDQGATSNIA
SGPAPLVVDIPDDLVGEEGANIHENEQTEVKVEDIDSPGSATSKFVELPSEKDVAPVDVLKHGGIFISDDSKSRRLNVNM
CSFTEESAKSRSSDVKLCSFNEEKARDTLVCWERSQPTAGYSYSASNGILNGGEDALYNLILAANKDSARRAFEVFKNLL
PASKCSFDFSRTVRVSLLQIDHAVEERFAKRKQFQQFKEKIIALKFRVHQHLWKEDIRMLSVRRFRAKSQKKFDLSLRPV
QIGHQKHRATVRSRFSTTVGNLSLVPSSEILNFASRLLSELGAKVYRNTLRMPALILDQKERMMTRFISKNSLVEDPCAV
EEERGLINPWTSKEREIFIEKLATFGKDFRKIASFLDHKTTADCIEFYYKNHKSDCFERTRRKPDYSKQAKVCSANRYLV
ASSGKRWNREANSASLDILGAASAIAANVEDSIEIRPKGTSKYTVRMITEYKASRLNELERSNSLDVCHSERETVAVDVL
AGICGSLSSEAMSSCITSSVDPGEGNQEWKHQKVGSSTRLPRTPEVTQSMDDETCSDESCGEMDPTDWTDEEKSAFIQAV
SAYGKDFITVSRCVGTRSRDQCKIFFSKARKCLGLDKILPVPGNLERQDVNGGSDPDACSMETKLLCNGKSSLMLEDVSD
LCMKVGISNHNLTSSDEKDGAGELDSVDILQVNCDVDKQEVEFNRDCDQIGACIGNGRGDENMVIVSQEGVEIGGDASEN
GLLDIPCPNEVSAKHLGEEISSPEHDLKDRKAEIAEVGRSNCSLEDRKPNMVLSGNNSGLAAPRGGGLCPLNGFQNMTHL
ESDSECKLYLESNISVQSKQMPEGANADKLSEELENVCDEQCENATQSAELPLPSHSRLAQVESCQILGSYSVGESALTQ
FGDPGCRTSAALQEIQKVGRNLQLDTFSTTGCFLQKCNGTNRGGGSVSDVLIPNREQTAGSSSNLEKHCRNGDVKLFGQI
LSKPCPQANPSSNAHRSDGSNQQLKIGSDSFSASHILDGNSATAKFERNNFPCSETHQLRSFGFWDGNRIQTGFSSLPDS
AILLAKYPAAFGNYGISSTKMEQPTLHGAVKMAERNLNGVPGFPTNASGNNGVAAADYQVYRNRDVQPFTIEMKQRQEAF
SEMQRKNGFDVVSGVQQQARGVLVGRGGILQCTGVSDPVAAIKMHYAKAEQFSGQTASIIREDDSWRSKGDVSR

>CcMYBR13 |Locus_ID=BC332_17457 |Protein_ID=PHU16252.1 |Protein_len=464 |
chr06:245576455-245578945 |strand= +
MGIDTHISQWILEFILRQPLDDNILNDLINVLPLPNDNLNLKKALLVRKIESEIANGSVTEKILELMELIEELDHQEGII
RDSVATKAAYCAVAVECTVKFLKSERAGSDKGKYFEAVRRIWKRRINLIEKVENVGVLSEELWSWRDEIEAALWEDRCAE
NVIRRSKGVFAVDVVNAFVREAKERIGAPFLEVVAETYHTDEAVKSVFGEVNKGGASKKNGKEVCKGNALPRKKHVAFKR
TRGASGIRISDSIESEVEASGGQDDSSAEVKIAKKALKLSSLELRAMVKDPLPDALRFAETLCSVVRDNMGHQPAENNSD
MAPQPMVSSSRMAQAGGENREAQQNCHHAAASKPDLANRSSAAHTFEWDSFLDDINEGSPSGVNRVTLPSPKRTKVSPLK
KYEFKKITTRRKPVKWSALEEDTLRTGVQKYGIGNWKVILDAYRDIFTTRQSGDLKDKWRNMIS

>CcMYBR3 |Locus_ID=BC332_15220 |Protein_ID=PHU14015.1 |Protein_len=378 |
chr06:81614676-81616401 |strand= -
MEGGSSSTEESKLSPKNQIEFGEEEEEEDDLETEEKSKAQGTTRTCSSNSTVEENGKKTNSGSVRQYVRSKTPRLRWTPE
LHLRFAHAVERLGGQDRATPKLVLQLMNIKGLSIAHVKSHLQMYRSKKTDDPSQTVSTDGRTLLENGDHHIFNLTQLPRL
QGYNQTSSSSLRYENALWSRQPNSMYNSYNVNIGGGSRTSTRHGFASHINGYSSDGQLFPWNKPIDTEKKQNRPQFLQNQ
RFWPTQIGISSAKQNLLMTLQFNRNTVETATQFNRREQEMMTQVRANISLSSSRGKGWTSEEVAGTITSTSNKRKIQDSE
INLDLNLSLKATREDHDDHLKRLKAEQVGDLLSLSLYSSSTNDQENNGKKANTLDLTL

>CcMYBR4 |Locus_ID=BC332_24070 |Protein_ID=PHU07581.1 |Protein_len=278 |
chr09:211170957-211172475 |strand= -
MLEDSDETGCNSKTSSNFKKNEDETEENGSSKFKDGGSSSDSTLEESEKKPYVRPYVRSKMARLRWTPDLHLRFVHAMER

LGGQDRATPKLILQMMNTNGLSIAHVKSHLQMYRSKKMDGQSQGIGHHRKLFMEGGDPNIFNLIRFPMFPAYYQRLNSTF
RYGDASWNCHGNWMPTMGQTTLTGTGRGLYTTLNHLCTRIPSFPEIKDVLGSSRGNPSDDLTRLFNIAAKSQARAFDGNG
IASSPDLERAITPLKRKVSECDLDLNLHLGGYEVYRRC

>CcMYBR5 |Locus_ID=BC332_10237 |Protein_ID=PHU19086.1 |Protein_len=350 |
chr04:8011491-8017021 |strand= +
MEHGNGETSEEYYMEKVMSSIDLNEEVDHDHSKIQQIDDEAIEVISVESDKITNEENNSSSCGNEINIKNKMSKVRQYVR
SKLPRLRWTPQLHLSFVNAIHRLGGQERATPKLVLQMMNVRGLSIAHVKSHLQMYRSKKLGEFGQVLGHGHRANQRRNYL
YGDLSQRSFNPLQDFKMKNGAIVLARNFNDDDNINHHFQNSFARTQSRYMEWSSHQGTNNLLKMKILENHQICPIRQRHY
FLEEKKWPPFEFNKNKLNSQSLFHHTGPKWSCRYNNLDPKYKTPLRFEMKQDKKFILEKDYWLPDLQLGLSRSEENNMKE
KIIHHSNKVELSDINTMLSLSLPSYSSSAT

>CcMYBR6 |Locus_ID=BC332_04479 |Protein_ID=PHU26147.1 |Protein_len=382 |
chr02:137657966-137660949 |strand= +
MAENLVKQDHEEMMMIMENSSSSSIDLNEDNKGESEEEEEEEVIEVEDSSEKETERISNYNSCDVEKKNVRQYVRSKLPR
LRWTPELHRSFVQAIQRLGGQERATPKLVLQMMNVRGLSIAHVKSHLQMYRSKKLDESGQVLGRGNNRGMQGRGYFFRNH
LGGQRYNPIQDFKMKNGAIVLARNFNYDDHALKGRFRNSFSRPPYQSKGIISRYMQWSSDNQGGLCNTTKSLQGEDLLRM
KSWQTPRQAVIEENGIHEIVPIRSTQFLEQKKWPPFIPNQWEEKRADFCSAKNSHFLLQQNLGQPYSKWKYCRNNNALEQ
NLVTPFTFEMKQDKSPLEEKDWLPDLQLRLSRSTEDKNESNRRDQSDINTMLSLSLPTYSAT

>CcMYBR7 |Locus_ID=BC332_05115 |Protein_ID=PHU26783.1 |Protein_len=252 |
chr02:150879297-150881907 |strand= +
MMKENSSKNSPCNLSMRLGHVEQQILENSNKSSSGVRPYVRSKMPRLRWTQDLHRNFVHAVERLGGEDRATPKMVLQLMD
VKGLTISHVKSHLQMYRSMKHEQMMQAEAEAANGIKRNRMDGPDQLNYLNYYDGKALLDGHPNSKVTINYFNLIASTPNF
SPAQWKDMQEKMMGLEGQSKPACNMFRDFFNGCTTVQDGNYNIGECGSSLSNNRCSIIDTEEEAQDSSSTMSLEPSACLD
HGTNISLDLTLG

>CcMYBR8 |Locus_ID=BC332_11027 |Protein_ID=PHU19876.1 |Protein_len=150 |
chr04:142857940-142858392 |strand= -
MAMGRRNCTLCGGNGHNQRTCIEKGKSIKLFGVEITTSTVAAMSKIGRKIKKGNPWTEDEQIAFLKGLKFHGKGNWAKIA
KDFLPSRTSTQIASHAQKHFMRLDANSSERKYLKKSSVFDLHLEKTEDREHAIVPVETHHVPSFLSNYNR

>CcMYBR9 |Locus_ID=BC332_11026 |Protein_ID=PHU19875.1 |Protein_len=201 |
chr04:142716893-142718911 |strand= +
MFRKDYRRKIKKANPWTEDEQIAFLKGLKLHGKGNWANIAKDFLPNRTSTQIASHSQKHFMRLDVNSNERKHHKKSSVFD
IHLKKIEDIEHAIVPLENNQESLHVPIFLPNFNLTKRIQVPKVTPVTWVYMYPYHQYTSTSAKLLETIFCFLDFQDIKES
PILIRDPVIDFFVKGQAVQSAPLSSVIKVIGSLDKSIPCPG

>CcMYR1 |Locus_ID=BC332_26464 |Protein_ID=PHU05642.1 |Protein_len=415 |
chr10:214548398-214550730 |strand= -
MYHHHHQATNMHSSTRMSFPERHLFLQGGNANGDSGLVLSTDAKPRLKWTPDLHERFIEAVNQLGGADKATPKSVLKLMG
IQGLTLYHLKSHLQKYRLSKNHHGQANSSRDNKAAASMEKICESTGSPTSNPSIAPQPNNNIPISEAIQMQIEVQRRLHE
QLEVQRHLQLRIEAQGKYLQAVLEKAQETLGTQNLGTIGLEAAKVQLSDLVSKVSNQCLNSAFSEIQELSGFHTPQTQAT
QRLADCSLDSCLTSSEGPLRDLQEMHNNQVGLRTLNFGPCTEDIENQARLHRTDLRWRDDLKENRLFPKMDEDTEKEFAK
ETNWSNLSMNVGSQGENRNVNSSYVDGRLNGIDADIKLFHQAPIDRSDSTKPEKQVSPQEYKLPYFAPKLDLNTDDQTDA
ASSCKQLDLNGFSWN

>CcMYR2 |Locus_ID=BC332_26060 |Protein_ID=PHU05238.1 |Protein_len=414 |
chr10:188527457-188529757 |strand= +
MYHHHHQDNSMHPSTRMSIPERHLFLQGGNGNGDSGLVLSTDAKPRLKWTPDLHERFIEAVNQLGGADKATPKSVLKLMG
IQGLTLYHLKSHLQKYRLSKNLHGQANASGANKAAAGVERISENSAACISNPSMGPQPNKNIQISEAIQMQIEVQRRLHE
QLEVQRHLQLRIEAQGKYLQSVLEKAQETLGRQNMETVGLEAVKVQLSEFVSKASNQCLNSAFPDIKELSGFHSQRTQAT
QPTDRSIDSCLTSRDGSLRDNAMHDNPIGLRPFDFTPSMECKDIENDTRIQQTELRWCDNLKENRRLFSPVNEGREKAFT
RETNCNNLSISIGLQDEKLNGSMNYSDGNFSGTERDVKLFHQVAKRSESVPERQKSSQEYKLSYFEPKLDLNMHDETDAA
SSCKQFDLNGFSWG

>CcMYR3 |Locus_ID=BC332_27762 |Protein_ID=PHU02511.1 |Protein_len=339 |
chr11:27041239-27043111 |strand= +
MNLVLSSDAKPRLKWTPDLHQRFVDAVSQLGGPDKATPKSLMRVMNIHGLTLYHLKSHLQKYRLGKSQVTDQSFDEKKQE
GKLELCTIAHDDDTKENEISKSLNQRTYFDAESDQLSLGVSCDGSQNQMNESLQIARALQMQMDVQRKLHQQIEVQRHLQ
LRIEAQGKYLQSVLRKAQETLAGYGTSSVGVELAKAELSQLVSIVNMSCCPSTSLSAITEIDGSISKDTQTKNLKGGILS
SLESSLTSYESSVRKEDQNDTRNNKNTNCIGLSLNQETKERKRSRNNICDHDMCVEQPSGKRFLETIDLNSKCPNEYDEN

GPKVIDLNYKEVEQFNEYS

>CcPCL1 |Locus_ID=BC332_15022 |Protein_ID=PHU13817.1 |Protein_len=304 | chr06:31099385-31100299 |strand= -
MGEEVKITDGDAAFAGDDNRVLEWEDGLPSFDDLTPLSQPLIPPELASAFRITPEPAKTMTDVHRASESTFSSLRAGGGL
HMLSDKFNFNEGGRNENDEVDLTRDGSDSRKTRRIDPEMVPEEADSALRNEMNCGDDNSAKTLKRPRLVWTPQLHKRFVD
VVAHLGIKNAVPKTIMQLMNVEGLTRENVASHLQKYRLYLKRMQGLSNEGPSSSDHLFASTPVPQSLQQSGVGNGNGNSG
NGHSSNGHMPMPMPMPMMYPPQMVPMPMMGMSGHGYHHQYNMGQPRDWSGNKFGSYHHVAPGEK

>CcPCL2 |Locus_ID=BC332_17321 |Protein_ID=PHU16116.1 |Protein_len=293 | chr06:243275452-243276333 |strand= +
MGEEVVTKLTDYDSNGVNDDRLLWEAGLPSADDLTPLTLQLIPSELAHAFRISPESSKTMNDVNRASQNTFTSLQRWHSQ
DMASMNREETVTERDETDLVRQGSDPKKLRRVESGGTDEAADADSALCSEKFETVKRPRLVWTPQLHKRFIEVVAHLGIK
NAVPKTIMQLMNVGGLTRENVASHLQKYRLFTKRMQSNEEPSSSPDHLCTSRGATPQSLRESSESCHPHNTNGHMGMPTM
MPYQPQMMPMPMMMGMTNGGHVGMPVGYAGGPPAGFHHPYNFGYYHPVASNDN

>CcPHR1 |Locus_ID=BC332_07350 |Protein_ID=PHU22243.1 |Protein_len=461 | chr03:55690408-55697022 |strand= +
MEARPAVSVQNVVASQLSNCGASGTLSSSLCMFPTALGEKYPKFPDLQQASMEKELKQHPGTVASSLPSNSGAVGHMFSS
SSGFSADLHFSSVSTQEKHSGPTSFISQSTYSETSITFPHSGVLQSTASSQYLNENHEPWCIDPLPNFLDYSVDTPVQNS
QVACSDKQSDWQEWADLVINDEDALTSNWNDIMADTSIADLELKYQEGKQPLNFQMQQVQESEQIPAVPVENLVIAPASS
PASSAATKQRMRWTPELHEAFVEAVNKLGGSERATPKGVLKLMKVEGLTIYHVKSHLQKYRTARYKPEASEAGSSEKKQS
SLDDLSSLDLKTGIEITEALRLQMEVQKRLHEQLEIQRNLQLRIEEQGRYLQMMFEKQCKSMPGVDLAKGSSSTADDAFA
QLSDAVQNSPGTNEPGVSKFVTAKEVDEKQKLREREVLEDPETNITSPSDSPPLKRSKLDE

>CcPHR2 |Locus_ID=BC332_14656 |Protein_ID=PHU13451.1 |Protein_len=434 | chr06:3724639-3729428 |strand= -
MEARPALSVQREGAHPLSNCGAHSSLPVLRASLEEKYPKFPGPVLSSLPSNSGAVGHLFSSSSEFSTNLHFSSVLPHEEH
SASAPFIPQATNTEASVRLAASSQYVNENSEPWCIDPLSEYLDYSLNTPVKNTQLDCSNTGDCHIPPEDLSKQSDWPEWA
NLIMNDDDALSNWNEIMVDAGIVDAAEPKMQYQEQNQPSNFPAHQGQQLQQIPSASLETSGIVPASSTASGASSKQRMRW
TPELHEAFVEAVNKLGGSERATPKGVLKLMKVEGLTIYHVKSHLQKYRTARYKPEASEGSEKKESSIGDLSSLDLKTGIE
ITEALRLQMEVQKQLHEQLEIQRNLQLRIEEQGRCLQMMFEKQCKSMPGIDASAQPTDAVQSSSNKNDPGVSLVDHHEAG
DRTQRTVGEKQKENEKEDTSSSNSPPSKRAKVDE

>CcPHR3-1 |Locus_ID=BC332_14381 |Protein_ID=PHU18686.1 |Protein_len=448 | chr05:234637395-234640307 |strand= +
MNMRRALCIQRSSEAQNGNMGVSGAMSTSSNLIPSQAIPSASKSRTVGHLLSSTSEPHKDFNFSSTSSQESRQRKYPFIS
SEASAATCRSSLSSIHSTSLDNYPIGNDSNSSGKDACHDFINFPANDLVQNGQVESLAVVMPSDDQAKRFDWQEWADQLI
NDDDDRLGSSWSDILVDINPPEIKFLETSEVLACESHILPPPPSPAAAAAASSGQICPVGSPSSTTALAKPRMRWTPELH
EVFVEAISKLGGSEKATPKGVLKIMNVEGLTIYHVKSHLQKYRTARYKPEPSEGTPEKKPTSVIEMPSLDLKTTMGITEA
LRLQMEVQKQLHEQLEIQRKLQLRIEEQGKYLEMMFERTKDIGKDFKVSSSITDEHPSPSTEPKHSPPNDKSEALEKDPV
SLNDRSSSGGKSPDKKAREDHDTDGTDSCCSPPSKRARTEERSAVHES

>CcPHR3-2 |Locus_ID=BC332_14380 |Protein_ID=PHU18685.1 |Protein_len=392 | chr05:234617887-234620559 |strand= +
MGVSGATSTSSNLIPSQAIPSASKSRTVGHLLSLTSGPHKDFNFCSTSSQESRQHNYPFISSEASAATCRSSLSSIHSTS
FDNCPIGNDSNSSGKDAYHDFINFPTNDPVQNGQVESLAVVMPSDNQAKRSDWQEWADQLINDDDDGGLGSSWNDILVDI
SPPEIKFLETSEVLACESHILPSPAAVVAAASSGQSYPVGSPSSTTALTKPRKRWTPELHEVFVEAVSKLGGSEKATPKG
VLKIMNVEGLTIYHVKSHLQNCQIRYKPEPSEGTSEKKSTSVIEMPSLDLKTTMGITEALRLQMEAQKQLHEQLEIQRKL
QLRIEEQGKYLEMMFERTKDIGKDFKVSSSITDEHPSPSTEPKHSPPNDKSEALEKDPVSLNDRSITGQESS

>CcPHR4 |Locus_ID=BC332_14379 |Protein_ID=PHU18684.1 |Protein_len=99 | chr05:234614315-234615237 |strand= +
MGITEALRLQMEAQKQLHEQLEIQRKLQLRIEEQGKYLEMMFERTKDIGKDFKVSSSITDEHPSPSTEPKHSPPNDKSEA
LEKDPVSLNDRSITGQESS

>CcPHR5 |Locus_ID=BC332_14389 |Protein_ID=PHU18694.1 |Protein_len=405 | chr05:234698981-234707259 |strand= +
MVAFPASLVSSAFEIHGDCNLRIAGTSEKKSTSVIEMPSMDLKTTMGITEALRLQMEVQKQLHEQLEIQRKLQLPIEEQG
KYLKMMFERTKDIGKDLKVSSSITDEHPSPSTETKHSPPNDKSEALEKDPVSSNNCSSSSSGGKSPDKKVREDHDSDGGD
SCCSPPSKRARTEERRMTTPDDAETKVKVKDIMNAAQIHLVVATLLVTVTFAAGFTLPGGFESDHDSPNKGMAILTKKSA
FCAFVVTDAIAFVCSAGAVFSYFVMAANHRPKTAKELRVLMDIYRVATILQFLSMFAVVIAFITGEWVKRHEAFRDRLVR

GIRNNNLGMKYEDQFIPRMLLKWTEAAKYLRSRKTSAYITVNYYSYSHAEVGSLTTPASRKNNHNRAEYNERDAANDTDS
NGWTN

>CcPHR6 |Locus_ID=BC332_26869 |Protein_ID=PHU06047.1 |Protein_len=423 |
chr10:225668105-225672293 |strand= +
MSIQNYELPSDYNLEFPQMGFCFQPENDHQQLQQQQNLWPSNDSSSSSRSMISRIGSSPSGFFATERYLGLTQYEYQDNN
NNNSCSQLSKNHDPQVAAFTQQCGNGFLVTSSSARVDTDIPKISMPSFIRPLDSHGLYGNPFSNLSEKERVLLLKRGHGF
KPWKQPLAEMQGKAAYDTPLWWGPSPDTAHSGSVSAPGFPFMKVSNNTCGFNLVHIRQQSGSQSANSYNNSGCSGGSLSS
KTRIRWTQDLHDRFVECVNRLGGADKATPKAILKLMDSEGLTIFHVKSHLQKYRNAKFIPESTEGKTEKMDKQNNVTQID
GKTGMQIKEALHMQLEVQRRLHDQLEIQRKLQLRIEEQGEQLKKIFEQQQQTTRSLLETRNSSISSHADPFTPHEDEVFV
EESFDNSHFQSKISYNDMETTFV

>CcPHR7 |Locus_ID=BC332_26620 |Protein_ID=PHU05798.1 |Protein_len=263 |
chr10:220993713-220995148 |strand= +
MSSYDPQQCRNGFFEDSMVQSEPISLPSFISPEGPCRQYTFSDASEEERMLHLKNKLLGEFDASYRRHPALPFDGNQDYC
LCHDLCGSQLTCMQQQSSSPSLTCHNSASSGATQKPSKTRIRWTEDLHDRFLECANRLGGADKATPKQILNLMDSESLTL
DQVKSHLQKYRNAKHPESAGKSEKRNSLDTVTDIESKTATEITEALKMQLEVQRCLHEQLENQRTLQTRIEEQAKKLRMI
LDQQQNTNRTVLETRNSNVSSDN

>CcPHR8 |Locus_ID=BC332_16139 |Protein_ID=PHU14934.1 |Protein_len=332 |
chr06:203462411-203466083 |strand= -
MYQPKGVPTSSLAHNNVAVNNQSLDCAGGSMDPISGGTNNPSLASKQRLRWTNELHERFVDAVAQLGGPDRATPKGVLRV
MGVQGLTIYHVKSHLQKYRLAKYLPDSSSDGKNSDKKEPRDMLSSLDGSSGVEITTALKLQMEVQKRLHEQLEVQKQLQL
RIEAQGKYLKKIIEEQQRLSGVLSEVPGSGVTAPPTGDNGPESDNRTDPGTPAPSSESPHVDKPVKAHALTKSLSMDESF
SSHHEPLSPDSDFHETSPIESPNGERSSKKQRVGTSAAFTKDNMALPHQILESSLSSPYQQPHSAFMTRDQFNFTSGLSL
GIDDQKVSGSNI

>CcPHR9 |Locus_ID=BC332_23400 |Protein_ID=PHU06911.1 |Protein_len=80 |
chr09:47507492-47509477 |strand= -
MASNRSDCSSKERLKWTQELHDLFEKAVSQLGGPERATPKGILKVMGIPGLTIFHVKSHLQLASPKGKAYLKFCQTSALQ

>CcRAD1 |Locus_ID=BC332_32239 |Protein_ID=PHT98820.1 |Protein_len=102 |
scaffolds:433813-434481 |strand= +
MATNSTWSNQQNKLFENALAIYDTDTPDRWRKLAKAVGGKTEEEVKSHYEKLVEDIKHIESGNVPLPNYNNGGGGGGGR
SNNGNNIIMDHEQQRLKYLKLQ

>CcRAD2 |Locus_ID=BC332_22475 |Protein_ID=PHU10615.1 |Protein_len=99 |
chr08:191562318-191563676 |strand= -
MASSSLRSSSSSWTPQQNKLFERALAQFDKDTPDRWQNVARAVGGGKSAEEVKRHYEILLEDLRRIESGHVPFPTYTNNA
NNGVGANEEHRLLRYMNLH

>CcRAD3 |Locus_ID=BC332_31082 |Protein_ID=PHU01295.1 |Protein_len=89 |
chr12:212285385-212287135 |strand= +
MATWTTKQNKKFEDALALYDKDTPDRWHNIARYVGGKSAEEVKRHYELLEKDVMKIENDQVPLPNYRANASNGRSNYANE
QRLLQNLRL

>CcRAD4 |Locus_ID=BC332_33762 |Protein_ID=PHT97307.1 |Protein_len=92 |
scaffolds:351-1915 |strand= -
MATNWTTKQNKKFEEALVMYDKDNNGEKWHNIARHVGGKSVEEVRRHYDLLLRDITQIENGQVPLPNYRNASESNARGYA
NEQRLLKNLKLQ

>CcRAD5 |Locus_ID=BC332_32248 |Protein_ID=PHT98829.1 |Protein_len=92 |
scaffolds:522402-522680 |strand= -
MASWTARQNKKFKEALALYDRDTPDRWHNIARCVGGKSAAEVRRHYELLVTDIMQIENGQVPLPNYKAAENNNRGYANEL
RYPKSLLSIQPN

>CcRAD6 |Locus_ID=BC332_32247 |Protein_ID=PHT98828.1 |Protein_len=89 |
scaffolds:512698-513428 |strand= -
MASWTARQNKKFEEALALYDRDTPDRWHNIARCVGGKSAAEVRRHYELLVTDIMQIENGQVPLPNYKATENNNRGYANEL
RLLKNMKLQ

>CcRL1 |Locus_ID=BC332_22472 |Protein_ID=PHU10612.1 |Protein_len=87 |

chr08:191552066-191552329 |strand= +
MSSSQASQGSASSWTAKQNKAFERALAVYDKDTPDRWSNVAKAVGGNKTAEDVKRHYEVLIHDIMFIESGGVPFPNYKTT
RGRTNTN

>CcRL2 |Locus_ID=BC332_25561 |Protein_ID=PHU04739.1 |Protein_len=88 |
chr10:95640860-95641126 |strand= -
MSSMSSQHGSPGSWTAKQNKAFEKALAVYDKETRDRWSNVSKAVGGKTAEEVKRHYEILLRDVFYVETGRVPFPNYKTTG
GSHNSTSD

>CcRL3 |Locus_ID=BC332_05377 |Protein_ID=PHU27045.1 |Protein_len=78 |
chr02:156122441-156122677 |strand= -
MASNSLSAWTPRENKKFEQALAVYDKDTPDRWQNIARYVGGKSVEEVKHHYAILVEDLKHIESGDVPFPKYKSDGKSR

>CcRVE1 |Locus_ID=BC332_07817 |Protein_ID=PHU22710.1 |Protein_len=893 |
chr03:163501351-163508906 |strand= +
MTNGKRAEKNKDIMELKDDVLVWEIISRLPLKLAVQCKVLNKDIKGRISDPEFLRTWFRRQEDPSTELIYTLNGRHRSLH
KITLYPIPNTQMETPLSCDIEILASCNGLILIDFDRVTKYCVFNPLTGEHQLIPYPLMPSNRLHSMGFAVDYPDSDQYKL
VTISERVKYSHRFYKFSLLSSQQPGFWHEIQQETSSFRPFPEGNPPVYWCGSLYWLRCEGSVVAFDTRRQASRVIEARFI
PQYDFKYGNISTGNNMRLGTAQGVLTLACIFRRLIVLATYDNARSSWTVTHTVDNFISRMSGFVAGFPVWIDSKQVSFLM
ERPWVVTHYWDLYEYDTEINGYRKGAEWHSVGYPMYYFHPTLASVHQAPFKNVEADDLLYIAAKLNYIRGFIIEGTSQFQ
NQAEGAWQGASSVNSSKLEIVATQEKAPTCLANKNALKVRKPYTITKQREKWTEEEHQRFLEALKLYGRAWRQIEEYVGS
KTAIQIRSHAQKFFAKVARDSVNDGDESLNAIDIPPPRPKKKPLHPYPRKMVDSPVANKAVSGQPESSPSPNVSGRVSRS
PDSVLSAIGSGVSEYPVAEQQNSRFSPASCTTDVHTANIISAENDDESMTSNSSTVEEVHVELKPISASTSPIANSDMVI
PLEACNLSALPCYLGCTLTSGLYVPLQECDIVHRENSCNGEKLAVEAPSASIKLFGQTVFIPDANNLALRAPYNCNSLPS
KSTEKEIKISSEDVLHSFQANQANSQFMLAMVPGNMIPPASWLSHNMLENNPEITTVFPTTVSWWSWYQDLVNRSILSCD
QKAMETAVHCQGPKDEEPQREGSSTGSSIGSASEVDDGNRSSETAESKCTAKSYKWNNSKGFVPYKRCLAERDDKSSGAV
LEERESQRVRVCS

>CcRVE2 |Locus_ID=BC332_03406 |Protein_ID=PHU25074.1 |Protein_len=506 |
chr02:49791267-49800127 |strand= +
MQLMECRVGLDHGLTEIIVKTDTGSRQQLIYRDQSESDTTPPSRSRISLAVGARIQLEEQVTPAEEYAPKIRKPYTISKQ
RERWSEEEHKKFLEALRLHGRAWRRIEEHVGTKTAVQIRSHAQKFFSKVVRESNNGDASSVKSIEIPPPRPKRKPMHPYP
RKMATPVKSGTLAPGKLKSSTSPDLCLSEPEDQSPTTVLSALGSFAFGTVHSSKPSEHSSPVSSDVAENSGDVVLSEPSD
IILEERRSSPARGYASSNQDNQSCMKLDLFPEDNDFVKEGLDEASSTQCLKLFGKTVLVTDSYKPSSTCGEILLTDENDE
PASPTLPQSSVPTKYLRWGSECARSTSTIGLPAQSYYIPAPNGSSGPNQSTTSPLLPWGSSYVSDKFPCAKVHSPIPIKG
CPLFNETGLDDKETQKEGSSTASNAESADVESSGDTNLEIEAQSGNGNLVVVSVRVSVPPLFERRANFTKRVEGFVPYKR
CLAEGGISSSTITGEEREEQRTRLCL

>CcRVE3 |Locus_ID=BC332_26256 |Protein_ID=PHU05434.1 |Protein_len=287 |
chr10:205096625-205102246 |strand= +
MANNSSTPTDASGKKVRKPYTITKSRESWTEEEHDKFLEALQLFDRDWKKIEDFVGSKTVIQIRSHAQKYFLKVQKNGSI
AHVPPPRPKRKAAHPYPQKAPKNVLVPLQASMGYPSSMNSLPPGYPSWDDTSVLINSPTGGVMSSQDEYHLQRIQADIGS
KGATLIGNSSISGIRSSNRAAPNSELPEQSKLGSVPHGIPDFAEVYNFIGSVFDPDTRGHVQKLKEMDPINFETVLLLMR
NLTMNLASPDFEPIKNVLSTYDLNTKVVGLPTGGAVSNHNDLSCQTI

>CcRVE4 |Locus_ID=BC332_21524 |Protein_ID=PHU09664.1 |Protein_len=319 |
chr08:170384947-170392394 |strand= -
MVSVYPTLPGQDFQYMGDPMKTGLRGLHPVQSGTGLVMPDDPNKKTRKPYTITKSRESWTEQEHDKFLEALQLFDRDWKK
IEAFVGSKTVIQIRSHAQKYFLKVQKSGTSEHLPPPRPKRKAAHPYPQKAPKKVMSQVGSIQFQPPGALPEPGFGIGPDP
LSVPGNAINFSPWTYDNVPAISTTQMRKDDAQLTSGGVMQNCSSSSNESMPRNRKTLEANDQKESQKQMKVMPDFAEVYS
FIGSVFDPSTRDHLERLKKMDPIDIQTALMLMKNLSFNLSSPECEDHRRLLSSYGLGIEKDKMDLPIKTDFRGNAIQAV

>CcRVE5 |Locus_ID=BC332_15390 |Protein_ID=PHU14185.1 |Protein_len=301 |
chr06:114656963-114672905 |strand= +
MVMALPNLGSFSNPTATASPAGATASTSNPLSPSDDPSKKIRKPYTITKSRESWTEPEHDKFLEALQLFDRDWKKIEAFV
GSKTVIQIRSHAQKYFLKVQKSGTNEHLPPPRPKRKAAHPYPQKASKSAPAVSPVPTSFQASLPLPEPGFVKRPDSSFLP
SNQVTVVPVPSWTDNSVPPVSLSQIKKDNVRETGQLVANNRCCSSNESTPRSKSTVKVIERTQAPSLRVLPDFAQVYNFI
GSVFDPAVTGHLQILKKMDRIDVETVLLLMRNLSINLTSPDFEHDRQLLSSYDIDMEKKCK

>CcSRM1 |Locus_ID=BC332_03442 |Protein_ID=PHU25110.1 |Protein_len=392 |
chr02:60415030-60427475 |strand= -
MTIDESSSSIWSREQDKAFENALATYPEDSADRWEKIATDVPEKTLEEVKHHYDLLLEDVSRIESGSVPLPCYNSSSDSS

PSHGGDDGTGKKTGNSGNLNGESNHGGKSMRADQERRKGIAWTEDEHRSGSRLSYLCEASAGLSILAPPDELDDSFKLHF
PDMLESGKCSVLEWYDELLECLVDSYLVPVPIAGGRYPVELVEVHASLYRESDNLIMLYLRGFENRLFLLGLDKYGKGDW
RSISRNFVVTRTPTQVASHAQKYFIRLNSMNRDRRRSSIHDITSVNTGEVSVPPGPITGQANGSSAGSSSKPNKQQTPVR
ATPLGVSIYGETSIGQPIGGPFVSAVGTPVNLPPPTHMEYGVLPPIPGAVVPGAPKNVGPLTYPMPHTSMHR

>CcSRM2 |Locus_ID=BC332_12967 |Protein_ID=PHU17272.1 |Protein_len=316 |
chr05:26151885-26154239 |strand= -
MTLAKSRSSIWSREQDIAFENALATYPEDSADRWEKIAGDVSGKTLEEVKNHYEILVDDVSRIESGCVPLPCYNSSVDGS
SSHGGGERTGKKSGISGRLNGETNDGGKALRLEQDRRKGVAWTEDEHRLFLLGLEKYGKGDWRSISRNFVVTRTPTQVAS
HAQKYFIRLNSMNKDRRRTSIHDITSVNNGDVPVPQVPITGQTIGAGVAGSSGKSNKHSQTAPIAPVGVGIYGTTTIGQP
FNTTSASVVGTPVNLVPPTNKSCGVQPPIPEAVVSGAYNEAQIYTLVTFLKHKRTKSSEESYAYSCLVLSSSVVHE

>CcTRF1 |Locus_ID=BC332_22999 |Protein_ID=PHU06510.1 |Protein_len=515 |
chr09:6218453-6224925 |strand= +
QNSAFRSGLASLGVWFDEPETRDDHASISGALCFDEKRVLRGMERKGALYNIGSSIAPKRENDINHEVLDLKQSQFEAGK
VQAQNDLSTPCEDYLLDIEFAESITNLDYGSSRGLLDPILESQITLPSCDGTDSCGMPYSSGLVAQEAQSQNAMFGLTNS
ELHDLSHHKCESRILVEYNAVKFPTAFNPGDISNVDDVADFFAEAVSSEKMNDSMIDHSALTSNGISLMSNPTQNEGQDV
NCESFDVHESAVIGPPVTSQVINVDTPVTQKRLRKPTRRYIDESSDLNARRSRKKTEVSTSAPESKNKFLKVRCQKKSRA
ESMEMELFSEESSYKAIQVPFASLVNEECDERPASNAIQVFKPHKEDLHKEPMVVNPKDDAVAPKKLDQDGARRKHHRLW
TVSEVRKLIDGVAQYGVGRWSHIKKLYFSSSGHRTPVDLKDKWRNLLKACYLQKQSKITEKGKHNLSWRPLPKSVLHRVG
ELANMHPYPRNRRCESSRSPCASPSPEHFNNTNIQ

>CcTRF2 |Locus_ID=BC332_09582 |Protein_ID=PHU24475.1 |Protein_len=606 |
chr03:260635966-260641486 |strand= +
MDAIEAKTEVSVVPVTNTFGALEKEITDPIVYQLVRVDGDGRLVPATQDEIMVVEDLLEDDKCEPKLVPDTRQISESCIT
EGYLMARNLFQVPEEKSNVLVDAVPDLGNIDAPEKVAVHGLNPFSELSDFDQSVRTETCFLSQDASNEDMPSTSAAGSSW
KPDFSKLEGKICLDSLTVKELQETFKATFGRETSVKDKQWLKRRITMGLTNSCDFSCTTFIIRDNVVVKNGEEQIYHRVK
SRISADSEDGVANSDSIGSLGDRDNRINDDADLCGEDVSTSAFESCNVTKDLDTEQRTAKRARKPTKRYIEELSELESRE
SSEKLASPDKISRYRFACPQTHMRPTKNVRSNARPLVTREDSLGGSGIQIPFVSRIRRSRPRENFMPLLKLQPCGMDMVT
RKVTSAFDVSGPQEDDKRNNDLIKSSSSPGWTQQPLIAACEKDDHYSGMKIVELENDVEPNDCSEYNSDDNVVTVPTQKG
GMRRKHHRPWTIDEVVKLVEGVARYGAGRWSEIKRLAFASCSYRTSVDLKDKWRNLLKASFVQLPAEKGILNSRKQASVP
IPAAILRRVRELADMQGQFPPGLSTGKSSGHSSSDRSVHDARSGFL

>CcTRF3 |Locus_ID=BC332_29142 |Protein_ID=PHU03891.1 |Protein_len=562 |
chr11:248978922-248982420 |strand= -
MQNAIDPFEYNLNTSNESAMSFQQEPEQVVGAASVYFALEEEAIGVEQLLAEPEYDDILNTLLDFNTCAFSVPESYFKEF
SASDTVLTKSDCGALQRSHNEGDKTKEKFLDGLSNGDDENHCFISCEDYLLDIELEEDTPTLHDVTRDISCIESVNLENQ
LADSDGRDCGVHVLTLSGASTSSDHDATILDNFDCMSTDKLLKVFREMLGHQTPVSDNQWLKPHMIAGLENQEISDKNFS
FPKRSLDSSENQGVKVPPASCQNLLTISTAFASIFTFRRKPRVQQVKRRGRIQWNSFKSLSSVAGEIHLDCPDKWASKES
AEENVKCDGMKSGISEQYLKCKPSRRGFGRRYYRRGATVTSQGLGKRNILPTEDQLIRREGHLSKDCKANRAHDDNIYSA
EAQDDPSGTLSDQSSDDTSEDDWTIGCETRGTKQDRKRNNYWSTREVLKLVEGVSEYGVGRWSDIKKMFFPSSMRRSPAD
LKDKWRNLLRASCRRLQSRRGVDAKKKHRMRSIPQSVLNRVRELAVIYPHSKQRRSRISSTASRSFSSNVKFDNHLPVLA
YE

>CcTRF4 |Locus_ID=BC332_26430 |Protein_ID=PHU05608.1 |Protein_len=556 |
chr10:213500376-213503784 |strand= -
MQTAMDPFEYNWSTWNESATSFQQEPEQVVAAASVYFASEQEAIGVEQLLAEPEYDDIVNTLLDFNTCAFSGVPESYFKE
FSASDTILTKTDCGALQRSHNEGDKTKEKFLDGLSNGDDENHCFISCEDYLLDIELEEDTPTLHDVTRDISCVESVILEN
QLVDSDGRNCGIHVLTLSGASSSSDHDATIIDNFDCMSTDKLLMVSREMLGHETPVADNQWLKPHMIVGLENQEISEKNF
SFPKCSLDSSENQGANVPPASCRNLLTVSTAFASIFTFRRKPRVQHVKRREHIQWNSFKCLSSVAGEIQLDCPDKWDSKE
SAEENVKCCGMKPGISEQCLKCKPSRRGFGRRYYRRGATVTSQGLGKRNFQVGFDQLIRRERQLSKDCKANRAHDDNIHS
AEAKNDPSGTLSDQSSDDTSEDDWTIGCEPRGTNKDRKHRKYWSTREVLKLVEGVSEYGVGRWNDIKKMFFQSSMRRSPV
DLKDEWQNLLRASCRRLQSRRGVDAKKKRRMRSIPRTVLNHVRNLAVIYPYSMQRRSRISSAESLASSSNVKSDNH

>CcMYBR14 |Locus_ID=BC332_32768 |Protein_ID=PHT98299.1 |Protein_len=299 |
scaffolds:98862-101466 |strand= -
MGRYPCCNYDYSDLKKGPWTKDEDEKLIEYTYKNGHNNWRLVTKNAGLKRCGKSCRLRCNNYLKPGIKRGGFTKEEEQII
INLHSSFGNNANQDEGLIAWSSRIDGIECSLPNGYPSISSSLKVHSETFNKIETIQSRSACLMCNDEKHKSKNGPNSLEF
CQANESNSSANQVEGLNIAWSSRIDSIECSLPNDYPSISNSSKVHPETFDKIETIQSRSVWLLCNADKHKSRNDPNSSKG
IQANELNSNHVEGLVPSTSKSSNLNKNCISKFSELDDIDAWEIPQDDEAISFFWNTIFQ

>CcMYBR15 |Locus_ID=BC332_30589 |Protein_ID=PHU00802.1 |Protein_len=242 |

chr12:129732389-129733705 |strand= +
MGRSPSCDETGLKKGPWTPEEDQKLINHIKKHGHGSWRALPNKLAGLNRCGKSCRLRWTNYLRPDIKRGKFTQDEQKTIL
SLHAILGNNLPHLIALANLKELVEHHHSSWEGQQAAIAIRSLQTEVATLQYLHLLLQTPNMPANNTTISANIPEIEAYNN
LLNSMKADQIIIGNNSLNNIIPPPNSTSLQDSIPFSHLPELQASSSFHLSTSLHKKEIIKTPTTIYYFTMAASFFSGSCC
NK

>CcMYBR16 |Locus_ID=BC332_24180 |Protein_ID=PHU07691.1 |Protein_len=294 |
chr09:228494871-228507862 |strand= -
MNVDDIDNNNIVGDEEQDGEKNNDKNDVVSKGKYNLWKKRGRKSMKDINEGRSQSSAINNTVKRKDCIEWTTDLHFIFTK
AVKQLGEGRCYPAEILEVMNVPSLTKKQVVSHLQVQPGFYYGDHQDYGLNRHTQNGYNLDINAAHVPTYSVVVISVNPAC
RTRGICKTIIPLFGDLPSDGFVSLYLGHIHMVEYTFSVTFNLANRTFLPLVFGTFGRNSLKLYPSVDARPIEWPSAILAH
RGKGQIPNCLIPVRVRDIGASRQTSRSHPRWKRDTTTSHHRVYSQLAISSGLAR

>CcMYBR17 |Locus_ID=BC332_15032 |Protein_ID=PHU13827.1 |Protein_len=270 |
chr06:33697486-33699328 |strand= +
MEAVTAILGGGLRIGVVFQGKKVRDDNKTLFQAGISHEDKLDALGFTLEPSPSRASPPLAPEGRSRVLPCETAQPLTRCS
PAPTVIRNGIQQGTYNGFTDHPRTNLSNFIDSDHDSAPSPPDAFLEKNAADSRALVPVAAVDAEALAMVPMRKPKRSETA
QRRIRRPFSVSEVEALVQAVEKLGTGRWRDVKMRAFDNAKHRTYVDLKDKWKTLVHTARISPQQRRGEPVPQELLDRVLI
VHAYWSQQQARQQMKHHSETRLLLSDGCKG

>CcPHR10 |Locus_ID=BC332_14338 |Protein_ID=PHU18643.1 |Protein_len=104 |
chr05:233993199-233994296 |strand= -
MGVSGATSTSSNLIPSQAIPSASKSRTIQRKLQLRIEEQGKYLEMMFERTKDIGKDFKVSSSITDEHPSPSTEPKHSPPN
DKSEALEKDPVSLNDRSITGQESS

>CcRVE6 |Locus_ID=BC332_05079 |Protein_ID=PHU26747.1 |Protein_len=124 |
chr02:150335758-150336875 |strand= +
MFNSEMSLMVNNAIAPRDQSGESGVDSIHSSRNQISSDVGASSTRSIKLKEQVASADEYAPKVRKPYIITKQRERWTEEE
HKKFLEALKLYGRAWRRIEGRNEKRLMFIRDISWLSLHINGDLD

>CcMYBR18 |Locus_ID=BC332_00996 |Protein_ID=PHU28903.1 |Protein_len=120 |
chr01:29087811-29088173 |strand= -
MPQQWTIIAPILGRTSSQCIERYDKILDGEGWEREDDEKLLHLAKIMPQQWTIIASILGRTSSQCIECYDKLLDGENYDK
LLPWEIESHPDQSKPAPPDPINMDDDEKKNAFSCTADSRL

>CcA |Locus_ID=BC332_25948 |Protein_ID=PHU05126.1 |Protein_len=377 |
chr10:178193549-178210718 |strand= -
MVKSVMESGELSCGQSDVVISHLMNGRGKIYPTFIKNDRHVELHMLCVDSNNSRPILRVKVVRRSLKKASTSTLPPPPPP
VVDDTSMEYDCMSGDEWNNSEDYAEKECGGAEDRQFEPQGSHSFSDGTNLFVGQIFKDKNVYVILNIMNTAIIAKSSGVR
KGAWTEEEDFLLRKCIQNYGEGKWHLVPIRAGLNRCRKSCRLRWSLIAGRLPGRTANDVKNYWNSHLQKKLITAPHRQER
KYSTALKITKKNVLRPRPRTFSSSAKNNISWCTNKSTVITNTLDKDERDKEIGLNICQKLTSETSSTIDDGVHWWTSLLE
NCKEIEEDVAAVGIFEEKNKLVPSLLHDEINSLTMQQGQSDGWDDFSADIDLWNLLN

>CcBLIND |Locus_ID=BC332_29076 |Protein_ID=PHU03825.1 |Protein_len=315 |
chr11:247145324-247147354 |strand= +
MGRAPCCDKANVKRGPWSPDEDAKLKDFIHKFGTAGNWIALPQKAGLRRCGKSCRLRWLNYLRPNIKHGDFSDEEDRIIC
TLYANIGSRWSIIAAQLPGRTDNDIKNYWNTKLKKKLMGLIIPNNNNNNNFINIQKKSPHFLSTTTTTSFHQAQQNLGPL
ITGLEQPIISSAQQNILPNMMIMTNNYNFPNFGATNYNLQNYPKFEANCLQNYSQFGEVASCSSSDGSHCSQMSFGNKEI
NNNIKREEIMRFGQTIFEGINNQQFNLDYYGTMEQLATNCCTNGNNGSTSISSNNLLLYNDENCNKSNEIGMFYY

>CcMYB1 |Locus_ID=BC332_04984 |Protein_ID=PHU26652.1 |Protein_len=340 |
chr02:149207548-149209010 |strand= -
MGHHCCSKQKVKRGLWSPEEDEKLIKHITTHGHSCWSSVPKLAGLQRCGKSCRLRWINYLRPDLKRGCFSEQEERIIIDV
HRILGNKWAQIAKHLPGRTDNEVKNFWNSCIKKKLISQGFDPNTHNLLSRAAAAATPPHRNNKKKTNNNHTTSSVFTIET
SLSPSSTKELVSMDMIKASLAALSPFPHTNSSTTVLTMPQFQTSWSHLYESACNYYNHVPIKTNIVYQNHSTSTGTFGII
NENCMWGVTGFEPHHEFGPSNNIGHEEMQQGAQVQLQEEHCLEEVYKVNDLHEFNKHGRIAENVTFDDSSDFDFEFVDSE
LIPCGIYTNVNSIDQLAWDC

>CcMYB100 |Locus_ID=BC332_00785 |Protein_ID=PHU28692.1 |Protein_len=240 |
chr01:18958016-18958738 |strand= -
LKCLFCRWSLISEHLPGRTDNEIKNYWNSHLSRKVESLRIPSDEKLPQAVVELAKKGKQKQFIKQRCKRKTNASVLNSTS
ETAVPITPRLNIEKETPNTIGNGDNNAMELDRNDHQMLWHDDIVLMDDKDAEIDEDFIFNCLWNDEGENLELVEKNNNNN

NEIFGEDSYGTLNCWDWEYLGEILNNETLASTQEAGQENNGMSSVSIFDMNDNMPNCMNEEATVENIDLMQSDLVNWLLS

>CcMYB101 |Locus_ID=BC332_16135 |Protein_ID=PHU14930.1 |Protein_len=281 | chr06:203262885-203264828 |strand= -
MGRSPCCEKAHTNKGAWTKEEDQRLISYIRAHGEGCWRSLPKAAGLSRCGKSCRLRWINYLRPDLKRGNFTEEEDELIIK
LHSLLGNKWSVIAGRLPGRTDNEIKNYWNTHIKRKLISRGIDPQTHRSLNTTATISTTTAIPTTTTTTKNINMDFENVDQ
KPNIIIATSSSYDETKCSSGTTEETKPLDIIPKIPSSQVMINLELSIGLPLHTDHISSAESTASYNFLATVAPPPTVAVP
VVATASETVAKTVCLCWQIGYQGGTQYCGKCKNTTGFYRYY

>CcMYB102 |Locus_ID=BC332_33436 |Protein_ID=PHT97629.1 |Protein_len=269 | scaffolds:121483-122793 |strand= -
MGRSPCCEKAHTNKGAWTKEEDERLIAYIRTHGEGCWRSLPKAAGLLRCGKSCRLRWINYLRPDLKRGNFTEEEDELIIK
LHSLLGNKWSLIAGRLPGRTDNEIKNYWNTHIRRKLLSRGIDPTTHRPINEPSTTQKVTTISFAGGDHKTKDIEDQDYKM
INVKAESGLSQVEDEINSSPFREQFPDLNLELRISPPSRQNYQQSHHVQDLKQSPTRCFACSLGIQNSKDCNCSKNNNNN
NNNMGNIASYNFLGLRANGVLDYRTLETK

>CcMYB103 |Locus_ID=BC332_02487 |Protein_ID=PHU30394.1 |Protein_len=272 | chr01:193658210-193660316 |strand= +
MGRKPCCSKEGLKKGAWTAKEDMLLTEYIKEHGEGTGWRALPKKAGLLRCGKSCRLRWVNYLKPGLKRGTFTPEEDDLIV
RLYTLLGSRWSLIAGRIPGRTDNDIKNYWNTHLLKKLKAQGNEPRRHKSIRKSKEKEKASSQKKQTRNQKTKERKKKRKI
AENDSNIEEREQVAKKTEEQLQMQDFSQAVSENNYNFTGSSCSQDGTSSGASTSEINLEENNYNSGIYEKFQVIDELLLN
DQWTEYNNESHMMLKEIYEEYFQLVSKNSLSF

>CcMYB104 |Locus_ID=BC332_00057 |Protein_ID=PHU27964.1 |Protein_len=260 | chr01:682911-684231 |strand= -
MGRAPCCAKEGLRKGPWSSQEDSLLTNYINQHGEGHWRSLPLNAGLLRCGKSCRLRWVNYLRPGIKRGNFCEDEDDLIVR
LHSLLGNRWSLIAGRLPGRTDNEIKNYWNTHLVKKLKNTGIDPKPLKNFPRRKKTQKEGKKCKSQNSKGQSVQVEKIKVF
APKPIRISCVNSRDNSFENDTLSTSTCSSNSNFEEVDVRKEKENEVNFLARDLDFGELLKGDEFCDEFLMGESCNFSKCP
FSLDDNMVEKVYQEYLLLLS

>CcMYB105 |Locus_ID=BC332_20335 |Protein_ID=PHU08475.1 |Protein_len=277 | chr08:3613207-3615399 |strand= +
MGRAPCCAKEGLRKGPWSTKEDLLLTNYIKENGEGQWRNLPNKAGLLRCGKSCRLRWMNYLRPGIKRGNFSQDEDDLIIR
LHSLLGNRWSLISGRLPGRTDNEIKNYWNTHLIKKLKIAGIHPKVHKIFNKKEPKKKPKSDKPRKKQDKKKKNNKKKDQS
LVNNNNNNDTPQVVFVPKPIRLMRNYSVEMLSRPCQIFKFNMHASALLKSQNNAASSSDNSEELAENNKKNINIITKDDE
NFLFDEFCDFSNESMVDKVYEEYLQLISEKCYDPMLM

>CcMYB106 |Locus_ID=BC332_27082 |Protein_ID=PHU01831.1 |Protein_len=173 | chr11:1075349-1076543 |strand= +
MRKPGCDYKEEMHKGSWSKQEDQKLIDYITKHGEGCWRDLPKAAGLLRCGKSCRLRWMNYLNPNLKRGNFSEDEDDLIIK
LHALLGNRWSLIAGRLPGRTDNEVKNYWNSHLRRKLIKMGIDPKNHNISHYLRIKRLEYFQENSIKSENNAVISDATSSN
CVTSSLPDLNSLP

>CcMYB108 |Locus_ID=BC332_14002 |Protein_ID=PHU18307.1 |Protein_len=317 | chr05:223881846-223884303 |strand= +
MDHHQKIEVDSGRGGGRGGDYTKNNQEIREEDIVMDLRRGPWTVEEDFTLINFIAHHGEGRWNSLARCAGLKRTGKSCRL
RWLNYLRPDVRRGNITLEEQLLILELHSRWGNRWSKIAQHLPGRTDNEIKNYWRTRVQKHAKQLKCDVNSKQFKDTMKYL
WMPRLAERIQAAAASNPATASSSGPPTTTYIQNQEIQHSLPNMNHHMSDYTPIPFIEKTNSLNYSATSASSDNYSSDLTD
GCYNFPINQSNNQDYSQVNQSTNQMCYGESTINPTSYDFHHGFQGFQEVDQQNNTQWMDNPWNIEDVCFLQQLNNDM

>CcMYB11 |Locus_ID=BC332_27759 |Protein_ID=PHU02508.1 |Protein_len=419 | chr11:26930576-26932692 |strand= +
MGGASTSMGMFFADMNSLSINTPKSVDEGKGRKGSWDFQFLNKGGAVFSCCQNLDEVQNSEENNESIDLNASCYNEEKQI
TLHSTFNSNNSSSGGGNNGKETESGQSKLCARGHWRPAEDAQLKELVAIYGPQNWNLIAEKLEGRSGKSCRLRWFNQLDP
RINRRAFSEEEEERLMAAHRLYGNKWAMIARLFPGRTDNAVKNHWHVIMARKYREQSSAYRRRKMGQFVYRRSTLVVEEA
DSGKAQAPIISCQNSVNQFGATANTSVKINNDGPNEAAAATPSSTNNVTLLSPYSPFCAPPFDSLPAGHCGNEMMNILNQ
QGNKSWDRKMEITHHQHHHPTPPSNSLMMMNSMQQQSQYLQFPYVTTSRTQISSSPSAAQAEVLVKEEDRRSSSSNNNNN
SDNSTIAPPFIDFLGVGAT

>CcMYB110 |Locus_ID=BC332_18300 |Protein_ID=PHU11370.1 |Protein_len=214 | chr07:28014303-28015172 |strand= +
MKDKEVKTRMKRGFWKPEEDLILKNCVETHGEGNWATISEKSGLLRSGKSCRLRWKNYLRPNIKRGMMSEDEKDLIIRLH

KLLGNRWSLIAGRLPGRTDNEVKNFWNTHLNNKRSCKGKKKHVKSKEANTHSPQGKTQQCPAETVRNQEVATKTVLDSWI
EEMQDFNCSLLSPVMNNMPFLEDEPFIPILDDIVLLEAFTSTGKEAWSEIQTFL

>CcMYB111 |Locus_ID=BC332_18314 |Protein_ID=PHU11384.1 |Protein_len=277 |
chr07:30180883-30184740 |strand= -
MKVTEMRMLGWICGLTKKDSVINETIRDKVGVTSVKEKMQELRLRRFEHVMKRLTAERKSKSAMKDKEVKTRMKRGFWKP
EEDLILKNCVETHGEGNWATISEKSGLLRSGKSCRLRWKNYLRPNIKRGMMSEDEKDLIIRLHKLLGNRWSLIAGRLPGR
TDNEVKNFWNTHLYNKRSCKGKKKHVKSKEANTHSPQGKTQECPADTVSNQEVATKTVLDSWIEEMQDFNCSLLSPVMNS
MPFLEDEPFIPILDDIVLLEAFTSSGKEAWSEIQPFL

>CcMYB113 |Locus_ID=BC332_30875 |Protein_ID=PHU01088.1 |Protein_len=352 |
chr12:191929956-191931822 |strand= +
MGRKPCCSKEGLNKGAWTPIEDKILIDYIKVHGEGKWRNLPNRAGLKRCGKSCRLRWLNYLRPDIKRGNISPDEEDLIIR
LHKLLGNRWSLIAGRLPGRTDNEIKNYWNTNIGRKLQEAKGSGQPNRVTSSYRQRNRSSHAKSANPSPITQLQQNNQEQT
VQQDSNCLLQTDVGREGSSSSSSTCLVVCNKAIWCTKIFISPQNTDQSVNEITQIDNNDKAMTEENATTNIVTSSSSSLS
EQQQQPVSESYSPTNLSVEMENYNFNFMFGLDVDDPFLSELLSVPDLRDQILENSTISSTVDAGDEVGDCNISVKKERQR
SYFPPSSSQISIFSEGTQHNDLELWIDGFSSC

>CcMYB115 |Locus_ID=BC332_34193 |Protein_ID=PHT96881.1 |Protein_len=258 |
scaffolds:31973-33059 |strand= +
MNTPIICTTSLQVRKGAWNEEEDFLLRKCIEKYGEGKWHLVPARAGLNRCRKSCRLRWLNYLRPHIKRGDFDPDEVDLIL
RLHKLLGNRQFYVLDSPKFAGSSHLKVNDIRDLEWSLIAGRLPGRTANVVKNFWNTRLLRKVNIAPINNKIGDNINTKNE
IIRPQPRNFSSTMKNVSWCNNKSIINEANILENCYEIEEAIATGTRTPLCKNISSEKNCNEIDKTPCFLNGGGNAMQQGQ
SDGGWDEFSMDDIWNLLN

>CcMYB12 |Locus_ID=BC332_23833 |Protein_ID=PHU07344.1 |Protein_len=356 |
chr09:154409265-154416480 |strand= +
MENDTAEVQEVTVAVNGFYGGGDDEEVLITEDGSGKKNADRVKGPWSPEEDAILSRLVSNFGARNWSLIARGIPGRSGKS
CRLRWCNQLDPAVKRKPFTDDEDRIILQAHAIHGNKWASIARLLPGRTDNAIKNHWNSTLRRRCAELGRLRSDSSNVMED
VSVEKSKASSEETQSCGDVNSLKAIEGKDVSSEENQEDNLYEDKSQGDVQQSDAANDPHTLFRPVARVSAFSAYGSLNVP
ESQLQTPRLIPVQAPDLGISKLLEGAFTDRLVPHQCGHGCCGNGNQAINGSSLLGPEFVDYDEPPSFSSHELATLATEIS
NVAWCKSGLKNSSIEVIFNPTTRAVCGASHLPSRPF

>CcMYB13 |Locus_ID=BC332_13912 |Protein_ID=PHU18217.1 |Protein_len=398 |
chr05:220340289-220346172 |strand= -
MNENAEKSNFSAVAVTAAVENVSFEGGDAVLHGGGSEGNGGNASVDGNGGDDFVDDGGDVEMSESGRGNRVSSGRVKEPW
SREEDLVLMELIYKFGARNWSMIAQGIPGRSGKSCRLRWCNQLDPLLKRKPFTDEEDRIIINAHAVHGNKWASIAKLLPG
RTDNAIKNHWNSTLRRRFAEVWRVKPLCSVMLDDSIDNAKVLFEEIKSHGDFNQIKPLEMLEGSRAECMSSQSEDKTQIY
EIYDIPEKSPNFVSERSCSTSVENSDPSASRPTEKIGGFYVCNPSATSRIVPMQGPLIHTFSPDFDICKLLEGCVSAGET
TVPQGCGHGCCAASTNSSFKSSLLGPEFVEYEEHPPHLGHELASIATDLNNIAWIRSGLEKAGELRNSRHSSTQVGFG

>CcMYB14 |Locus_ID=BC332_31012 |Protein_ID=PHU01225.1 |Protein_len=242 |
chr12:206743102-206743830 |strand= +
MKTVKELIRVKGPWKKEEDELLQKLVEEHGAKNWSIIGKSIPGRSGKSCRLRWCNQLSPQVEHRPFTRDEDDKIIKARAE
FGNKWATIARLLTGRTDNSIKNHWNSTLKRKGLELSKDLAFENPQPPLKRSLSTGSGTNSGTPSGSDLSSGFARPDISTS
LSLSLPGFGSSEDSNTPIIQNCNFGPSSIEKQLFSPEFLRVVQDMIRKEVESYMSQYEEERVAMYTEAVKNNAINNIEIC
KD

>CcMYB16 |Locus_ID=BC332_11900 |Protein_ID=PHU20749.1 |Protein_len=326 |
chr04:224752264-224753244 |strand= -
MAARKDSDRIKGPWSPEEDELLQSLVEKHGPRNWTLISKSVPGRSGKSCRLRWCNQLSPQVEHRAFTPEEDETIIRAHAK
FGNKWATIARLLSGRTDNAIKNHWNSTLKRKCPSMSEDLSFDNPQPPLKRSASVGPSMMNPGSPSGSDLSDSGLSGSGSG
LDLPLPLVYRPVPRTGCIFPLPPPPAPSVPDPPTSLSLSLPGSVSVPGSGSFEKPRQSPQTPPLPPPQPPPFAAVEKPIP
PPAPVLSSTPPFMAQVPQTQQQSYEFCAPKSGEKQFFSPEFLSVLQAMIRKEVKSYMSGFEKNGFCLQTDAIRNAVIKRI
GISKIE

>CcMYB17 |Locus_ID=BC332_01622 |Protein_ID=PHU29529.1 |Protein_len=303 |
chr01:74523210-74526775 |strand= -
MSEIKKGPWKAEEDEVLIKHVKKYGPRDWSSIRSKGLLQRTGKSCRLRWVNKLRPNLKNGVKFSAEEERTVIELQAQFGN
KWARIATYMPGRTDNDVKNFWSSRQKRLAKILRNSPPQPNKPQKCIHREAPPATQKVPSVEAPKLNSSAEERPLSMSQDC
SSSYMNNFETTSMVPLRELVNSTSLPFEQELLPLEFTPNEKRTSIWPQFPLSFPQIPLQTTFDHPLGHQELPMKLEDTNF
LDYFDMGNVQVPLAPSCSGQERTSEIAMKREIMDYPLTPDSFIHDFPMDMFDYIDPLPSPSEW

>CcMYB19 |Locus_ID=BC332_15639 |Protein_ID=PHU14434.1 |Protein_len=505 | chr06:158086817-158091007 |strand= +
MSMTSESDDRMASQDGVDSPSVEEACGGGITGGGLPLKKGPWTSAEDAILMDYVTKHGEGNWNAVQKHSGLARCGKSCRL
RWANHLRPDLKKGAFTPEEERRIIELHAKMGNKWARMAAELPGRTDNEIKNYWNTRIKRRQRAGLPIYPPDISFQAISEN
KQNEELGAFSSTDSQYPDFLPMNNFEIPAVEFKRLEFNKQLCPPALLDIPNGGILDIPGRSLLAQGLNSAYYSRSFLSTM
PPAKRIRGAESLFSGLNGDCSPSKNEGSFPTCHQYQEDGSLLAQSMGFSSSFNQNLTSVHHPSSSGVIPGSHAPLNGKTS
SSEPLWAEKLELPSFQSQMASWGLSSSPLPSLESVDTMIQSPPTEHTESCNLSPRNSGLLDAVLYESQTMRASKSILHQE
NSGDVVDNSCPDLHETGWETYGDPISPLGHSAASVFSEYTPTSGSSPEEPQLVTMPGCKVKQEKFDFGPYDGKEDASDLI
FSRPDYLLESNCFGHMQKTARSIWH

>CcMYB20 |Locus_ID=BC332_01521 |Protein_ID=PHU29428.1 |Protein_len=509 | chr01:67429296-67432913 |strand= -
MSITSETDDMMTSKVDMDSPDEASGGESVPLKKGPWTTVEDAILVDYVMTNGEGNWNAVQRHSGLARCGKSCRLRWANHL
RPDLKKGAFTPEEERRILELHAKMGNKWARMAAELPGRTDNEIKNYWNTRIKRRQRAGLPVYPPDISFLANQNKQNEELG
AFSSVDAQNSNVLGINNFEIPAVEFKNLQLNHLLYPPPLGEIPAVSSFLAQGHRAPYGSTYLLSTMHPSKRIRGSESMFS
GSNGDLLLSSSQYHNGGSLLAQPLGFSSYNHHLTYDDDRSFSSVVHGGHACLNGNSSSSEPTWAMKLELPSLQNQTANWG
SPPSPLPSLESDDILIQSPPAGNSESGSLSPSNSGLLDAVLYESQTMKASNDNSHQGKETSGDAVNKGWESYGDPVSPLH
HFSASVFGEYTPVNGSSLHEFPSVATMPGCKIKKEIGDLAPLDENDDSLNQTIFSSPKTQHAKNSLALKEVISSGFFDDC
GWDCKQIRAVATSSGQACGCSSWDAMSAV

>CcMYB21 |Locus_ID=BC332_31637 |Protein_ID=PHT99442.1 |Protein_len=486 | scaffolds:6937567-6940075 |strand= +
MATNDRGMKNSGGKNGGGNRQVVKKGPWTTTEDTILMEYVRKHGEGNWNAVQRNSGLMRCGKSCRLRWANHLRPNLKKGA
FSPEEERIIIELHAKIGNKWARMAAQLPGRTDNEIKNYWNTRLKRRQRANLPIYPQELQSQNQPEHNQAQQSLISSAFDP
QRTTYNNPPSLSLLGILNPSSMRPPITHQFPLNGNKNFRNPPKGLSMTPPSSMQNSQFSSLPVSVSNNNFGHDLFNSTPM
PSFQQTYPDFSSTMRPFLGNIPSNQNGLMFDMGINSINYPSGQLSMPVTTSSSSENTSSGFGSSDANNYTNVGGLSRGNS
GLLEDLLEESQTLTRAMKIEERGKSLWENYGLVEKELGDHQDGAIFTEESVYSFAHGDDVTLYNNSESSSSNPNASPGTI
FKKEGSLKGTNQVDEDLMCLLDSFPLGVSVPDWNDHNSKIASDDYKAENLTEDSINSQVIITTNSGSKKHELGLEGCCWY
NMPSFC

>CcMYB22 |Locus_ID=BC332_24264 |Protein_ID=PHU07775.1 |Protein_len=479 | chr09:238380291-238383963 |strand= -
MAPNGGGVNKNNNGTSIGRNHNTRQVLKKGPWTTTEDAILMEYVKKHGEGNWNAVQRNSGLMRCGKSCRLRWANHLRPHL
KKGAFSIDEERLIVELHAKLGNKWARMAAQLPGRTDNEIKNYWNTRLKRRQRAGLPIYPQDIQPQNYQQDQNQQHSTNIP
SPFDNNHQNSNYNNNNSPLSLLDIFNPSTMKPSRNIIPHQYQFNNNNPTSSFLTNTNNINNQVKFFRDPRVSLSLTLASS
MKNSQLPSMIAPVPNNFSQGYSSSIPAPPLQHNYPNFTTTTRPFPGISSNPNGLILGMGTEQRSIQLSMPETTTTTTSSD
HADNNYVVERGLSRGNSGLLEDLLEESQTLTRGEKIEENCNVDNEDNKGKLVWEEYGLTEEAADAILTEESTYNFAHTDD
NNATQSKHSEDSSSLNSSSGITTKEASLELGNQVDDDIMRFLDNFPLGVPVPDWCDDQQNTSNAQSFECDQIQLSKSSS

>CcMYB23 |Locus_ID=BC332_06584 |Protein_ID=PHU21477.1 |Protein_len=260 | chr03:8824259-8825764 |strand= +
MVCEIISEQMGSNWGFIEEVWRKGPWTADEDRLLIEYVKLHGEGRWNSVARLTGLRRNGKSCRLRWVNYLRPDLKRGQIT
PNEEKIILELHARWGNRWSTIARSLPGRTDNEIKNYWRTHFKKKTKNSCDNSEKSRASLWKRQQFQLQQQQQQIKNQID
IKKVMSLPTMAQAKQEMAILYPNIYTIDQQDQVGLFYSMLNNSTSISQSEPSSSNEDIMWDDLWNLDDFYGHFINSTTYN
KTNTSPCLQPMASTTTPSFY

>CcMYB24 |Locus_ID=BC332_05804 |Protein_ID=PHU27472.1 |Protein_len=257 | chr02:163662294-163663398 |strand= -
MVAMMGWGANSDQQGWRKGPWTPEEDKMLSEYVKLHGEGRWSSVARCAGLNRTGKSCRLRWVNYLRPGLKRGHITPQEEG
IIIELHALWGNKWSTIARYLPGRTDNEIKNYWRTHFKKKEKASGKQDKRKIIRQKNQPQPNCTIATIDKKLSPQAEEVVI
MKSDDSNAYTNYYHKNIMDQDPVVELPPVTTCSDTSYAWTDTFLMDGLWGGLWNLDDHSLMQATNDKCKVANIQNQPANC
TYATDYAVNLHNGGFIF

>CcMYB25 |Locus_ID=BC332_14907 |Protein_ID=PHU13702.1 |Protein_len=238 | chr06:14503832-14505369 |strand= -
MAQEEMRKGPWTEQEDLQLAFYVNLFGDRRWDFLAKVSGLRRTGKSCRLRWVNYLHPGLKRGKMTPQEERLILELHSKWG
NRWSKIAMKLPGRTDNEIKNYWRTHMRKKAQDERKNKASTISPSSSFSNSSSFYSNSPDVDFTPIMENNERNFYDTGGVE
KKINDGGKMKVYSMDEIWKDIELSEESETMNNIKPIMSSPLWNYCPNNTLWMSDEEETKMFPQFYNCLDNQDNTFFAG

>CcMYB26 |Locus_ID=BC332_14908 |Protein_ID=PHU13703.1 |Protein_len=189 | chr06:14885819-14887203 |strand= +

MAQEEMRKGPWAEQEDLQLAFYVNLFGDRRWDFLAKVSGLRRTGKSCRLRWVNYLHPGLKRGKMTPQEERLILELHSKWG
NRWSKIAMKLPGRTDNEIKNYWRTHMRKKAQDERKNKASTISPSSSFSNSSSFYSNSPDVDFTPSTEDNERNIYDTGGFQ
KNVAENMKVYSMDEIWKDIELSEENETMC

>CcMYB27 |Locus_ID=BC332_17123 |Protein_ID=PHU15918.1 |Protein_len=228 |
chr06:231916922-231918274 |strand= -
MDRTRRSSASILCEDVWRSSLGFSGESFRFEVGWRERKNKLGLKRSGKSCRLRWVNYLNPGLKRGKMTPHEEQLVLQLHS
KYGNRWSKIAGKLPGRTDNEIKNYWRTHMRKLAQDEKKNNKASISPSSSNFSNCSSSSANSPAVDSSTEQNELLQVVEAN
KTYEEDQNMMVYSMDEIWKEVESSQEIETRNDLPVMASNTWDYCPDSLWMTHDHYYYDNTQDFSFFTG

>CcMYB28 |Locus_ID=BC332_22951 |Protein_ID=PHU06462.1 |Protein_len=206 |
chr09:5239565-5241556 |strand= +
MQEEKLRKGPWLDEEDERLAFVVSILGERHWDALAKASGLRRSGKSCRLRWMNYLRPNLKHGQITEDEENLIIKLQKQLG
NKWSKIAKQLPGRTDNEIKNYWRSHLRKKALIYEQECFGSNNTSGDSQPKLSFAKDDNCQSFDDGEEESSRLLDWMIPSW
TYEQSQMEHHMYLCKILNLCSCNYSSQDENISSTWDDASSSSIWEY

>CcMYB29 |Locus_ID=BC332_26333 |Protein_ID=PHU05511.1 |Protein_len=206 |
chr10:209309844-209310891 |strand= -
QLTMQEEELRRGQWLEEEDERLAMIVAISGERRWDALAKDSGLKRSGKSCRLRWLNYLHPNLKHGHITADEERLIIRLQK
QFGNKWSKIAKQLPGRTDNEIKNYWRSHLRKKAPVYEQECFEDNTSNSEQKSSISNCATVLNSSNSDDSFSEKGDCYSAD
SLANEVTLLDGIPSWSYEQSRMEHHMYFCSSNLCSCYAPWTSEDNN

>CcMYB3 |Locus_ID=BC332_04434 |Protein_ID=PHU26102.1 |Protein_len=345 |
chr02:136506858-136507895 |strand= +
MDRVKGPWSPEEDELLQQLVQKHGPRNWSLISKSIPGRSGKSCRLRWCNQLSPQVEHRAFTPEEDETIIRAHARFGNKWA
TIARLLNGRTDNAIKNHWNSTLKRKCCSLSADEGKELADQIFENHQPPLKRSVSAGSAMPVSGFHFSPGSPSGSDSDSSL
HVTSSSQSHVFKPVAKTGGIFPPPSIDISSPSIDPPTSLSLSLPGADLAELSSRSAESTQSKNPFQLSLPPPVQVPPPPP
PPPPPPQPQPVTAVPFEQQDKVFVPFSQELLRVMQEMIKTEVRNYMMGIEQKQQHPQQQHYQQQQQFQQQTHQLPSGLGL
GLCMQQASDGYRDRAANRMGISKFD

>CcMYB30 |Locus_ID=BC332_14493 |Protein_ID=PHU13288.1 |Protein_len=231 |
chr06:45830-46775 |strand= +
MEASMEMKKGPWSSDEDSKLIHSISLLGQGRWDSLARFAGLKRSGKSCRLRWLNYLRPNLRRGKITPQEQLLIILLHFRF
GNRWSKIAENLPGRSDNDVKNYWRTKVQKHAKELHCNVNNSHQFRHFLLYQWLPSLLAQQILPTTPPSSPNNTMTCPNAD
FISSPEDDNTRYNIWNNLSSFEDEINISHDYSIPTDDEYYCHNYWPTTPSQVLLPDEMLCLDDDMDLWLFP

>CcMYB31 |Locus_ID=BC332_19185 |Protein_ID=PHU12255.1 |Protein_len=199 |
chr07:199954179-199960897 |strand= -
MVRTPCYDENGMKKGTWTPEEDRKLTAYIAKYGSWNWRQLPKYAGLARCGKSCRLRWMNHLRPNVKRGNYTKEEDEIILN
LHAQLGNRWSAIAAHLPGRSDNEIKNHWHTKLKKRGTNYATNSSDESSKKCKNNTKKRYTESNTNKNTSHNNMQENIVLE
SSEWSPKESSSEELSSYSTTNYQQQHKVFQEEITSGSFW

>CcMYB32 |Locus_ID=BC332_03892 |Protein_ID=PHU25560.1 |Protein_len=196 |
chr02:114442162-114445894 |strand= -
MDKRPCNSQDAEVRKGPWTMEEDLILINYIANHGEGVWNSLAKSAGLKRTGKSCRLRWLNYLRPDVRRGNITPEEQLLIM
ELHAKWGNKWSKIAKHLPGRTDNEIKNYWRTRIQKHIKQPENVNGQGTSEQNIDHQEGSSSQMSSAGQVDTIETYSPTSY
NGNMDANFQAPNFLTETNDNMWSMEDIWSMQLLHGD

>CcMYB33 |Locus_ID=BC332_05936 |Protein_ID=PHU27604.1 |Protein_len=170 |
chr02:165584056-165586781 |strand= -
MDKRTCNSQDVEVRKGPWTMEEDLILINYIANHGEGVWNSLARSAGLKRTGKSCRLRWLNYLRPDVRRGNITPEEQLLIM
ELHAKWSKIAKHLPGRTDNEIKNYWRTRIQKHIKQAENMNRLSSDISDQHNNDQQASTSQASIGPPDTIETYSGNNNNNN
NIMGSNFHHQ

>CcMYB34 |Locus_ID=BC332_27190 |Protein_ID=PHU01939.1 |Protein_len=287 |
chr11:3303339-3304807 |strand= +
MSSNNNNNNLSSNEDELFELRRGPWTLEEDNLLIHYISTHGEGRWNALAKCAGLKRTGKSCRLRWLNYLKPDIKRGNLTP
QEQLLILELHSKWGNRWSKIAQHLPGRTDNEIKNYWRTRVQKQARQLKVDSNSKKFVEAIKNLWVPRLLEKMEQCSSSST
IDKKQNINNLSFPNLPLINNQESCTKDNKSSHDDDNTNTNSNNNTTWGSLGSSMNENFNICNNSMHQDDECYYVENNFNQ
QPQHFSYQEMSISECDEVAEADWFTNEGSLWNMDELWQFKKLGDVDI

>CcMYB35 |Locus_ID=BC332_09701 |Protein_ID=PHU24594.1 |Protein_len=293 |

chr03:262562871-262564270 |strand= -
MERDMSFLSKTSSSSSSDDDIELRRGPWTIEEDNLLVHYITNHGEGRWNMLAKHAGLKRTGKSCRLRWLNYLKPDVKRGN
LTPQEQLMILELHSKLGNRWSKIAHYLPGRTDNEIKNYWRTRVQKQARNLKIDANSAAFQEMIRYLWIPRLLQKIQSSSS
VMPSIQSPQSTPQDFNIEKNSTNTSSSSSCSSSHSSNIYNSTNIMSECPKIPPRDIGDSDLNSFVKAHFPVDDNSYDVDN
YGQLATGNYYNDVMDYDQMSGDQNKLTGDVLSDSFWSNMDEFFYQKQDMSHCR

>CcMYB36 |Locus_ID=BC332_31414 |Protein_ID=PHU01627.1 |Protein_len=292 |
chr12:224158300-224159991 |strand= +
MNHYSQYVDNNQKSDEETNNLDLKRGSWTIEEDFTLMNHIALHGEGRWNSLARSAGLKRTGKSCRLRWLNYLRPDVRRGN
ITLEEQLLILQLHSRWGNRWSKIAQHLPGRTDNEIKNYWRTRVQKHAKQLKCDVNSKQFKDTLHYLWIPRLVERIKASTN
SNNNQVNNIQQTNYDNIISNNNNNNNNNNNIVQNIMNNFKQENYSSTTTTSSENSFGSTQISDMSDNCYTNYPLNQGDYH
GEILINPTSDYFQHGIIDDNNHQQNMSQLFFDDVSDKFWNQEDMLFLQQQLN

>CcMYB37 |Locus_ID=BC332_31413 |Protein_ID=PHU01626.1 |Protein_len=269 |
chr12:224147657-224148659 |strand= +
MDKIRENNIVELKRGPWTVEEDFILVNYIAHHGEGRWNTLAHCAGLKRTGKSCRLRWLNYLRPDVRHGNITLEEQLLILQ
LHSRWGNRWSKIAQHLPGRTDNEIKNYWRTRVQKHAKTLKCDVNSKQFQDTLHYIWIPRLVERIQASKGQNNNSIRVAQQ
SVLRTGITELVPIQLANNSNNNNDNNNSNYLSASVTPENSSVATSSENSIGTTQVSHNQDYYQANQSDYHHQLWFEDNYQ
AMDQQNSIQLWMDNEDVFDNLCNIEDIWS

>CcMYB38 |Locus_ID=BC332_08081 |Protein_ID=PHU22974.1 |Protein_len=279 |
chr03:207146556-207148956 |strand= -
MGCKAAEKPKQKHKKGLWSPDEDDKLKNYIIKHGHGCWSSVPINAGLQRNGKSCRLRWINYLRPGLKRGAFSLEEEDTIL
TLHAMFGNKWSQIAQHLPGRTDNEIKNHWHSYLKKRVTKMAENEGDTKSDNTESSPSLKKLTPQNSSLDSFEHREGSLAD
SDQSIYPRESQKSNLPKVLFAEWLSLDQFNGQEFHNSGNFEPSTNNFGYNNSELHDIFMHSLPMNNGSYGNGVNQEVNNG
TVVDDIFQAQLKFEDPMSANGFEEFMSGEFNINDDVMYI

>CcMYB39 |Locus_ID=BC332_02119 |Protein_ID=PHU30026.1 |Protein_len=280 |
chr01:166163058-166167683 |strand= -
MGCKQMDKPKQKHKRGLWSPYEDHKLKDYILKHGHGCWASVPINSGLQRTGKSCRLRWINYLRPGLKRGTFSTEEEETIL
TLHGMLGNKWSQIAQHLPGRTDNEIKNHWHSYLKKSVSKMAKTYSENAEASSSSMKLVSKKSSNLGSFELVEGALLADID
QSTLHVQLPQENRECNLPKVLFSEWFSLDQFNVQDFKNTIKQHHSKNNYGCNDSVFQLDAFAQYGPLLAMNEAITYGNDI
NGGFNNMFQPPLKFDDQIFVNGFEEFISGEFNVNGGVMYI

>CcMYB40 |Locus_ID=BC332_02384 |Protein_ID=PHU30291.1 |Protein_len=366 |
chr01:185358207-185361437 |strand= +
MRKPEHNSSNPSPSTMMKEKEKEKGNKAKLRKGLWSPEEDEKLMNYMLTNGQGCWSDIARNAGLQRCGKSCRLRWINYLR
PDLKRGAFSPQEEELIVHLHSILGNRWSQIAARLPGRTDNEIKNFWNSTIKKRLKNNNNNNNHSNNSTPSPNASDSSSDP
RALVGGIFPMQGHDVNVMAAFCMDNSSSTTSGSSMQAMVPSNHFNPFPQLDNTSYDISGLVGPVNLGQFGCNGCDGGFLD
YGVEETYSMMGLESDEFSVPPLGGVHKSTMGENNNNNSHVNFSSNIVIGANDYMVEKKITNGNNNNNQHLMNMSGSSDQS
LKVEDYMVGFGNHNHWHGESLRIGEFDWEGLLANVSSLPYLDFQVE

>CcMYB41 |Locus_ID=BC332_28534 |Protein_ID=PHU03283.1 |Protein_len=357 |
chr11:213882044-213886319 |strand= -
MRKPEFSSSSSSGKNNNNNTNNNTNVKLRKGLWSPEEDEKLMHYMLTNGQGCWSDVARNAGLQRCGKSCRLRWINYLRPD
LKRGAFLPQEEEHIIHLHSLLGNRWSQIAARLPGRTDNEIKNFWNSTLKKRLKNSSSSSTPSPNASDSSSDHLSKDLNIG
VTQQRFMTMQKHNLMSINMDSTSSSSSSMALNTINIDPLPTLDHTLINMSNGFNISPSYLSTTQPCMVQGGNIVGTNNGG
SLFYGNNHGIFGGNLSMEGHELYVPPLENVSIDYNQNVENGNYVERSNHHQNNNYVNNMSSLNSTSHNFNSSSNIKVENF
GGIGNYWEGDELKVGEWDLEELMKDVSPFPFLDFQVE

>CcMYB42 |Locus_ID=BC332_25321 |Protein_ID=PHU04499.1 |Protein_len=331 |
chr10:35592237-35593896 |strand= -
MGHHCCSKQKVKRGLWSPEEDEKLIRHITTHGHGCWSSVPKLAGLQRCGKSCRLRWINYLRPDLRRGSFTEQEERTIIDV
HRILGNRWAQIAKHLPGRTDNEVKNFWNSCIKKKLIAQGLDPNTHNLLKNKSNNPTKKSDYTYHHQDSTSVFTIDTSTNK
EVISMDLKSTLATLSPNFPPSDNIITSSTYNYTPIVPNIEYQNPTFAWNSILDFASNCSLMESTTNINLSSYINENKMWN
GTGLEPTTLNPVNGQEEEMQVQLQGDQQFPIIQTKFCHDLEDVYKVNDHDQTVENTFDDNSNFDFEFMNSAFVPCGLYTN
DNSMDQLSWDC

>CcMYB43 |Locus_ID=BC332_09604 |Protein_ID=PHU24497.1 |Protein_len=381 |
chr03:260945914-260947886 |strand= +
MGHHTCCSKQKVKRGLWSPEEDEKLIKYITTYGHGCWSSVPKLAGLQRCGKSCRLRWINYLRPDLKRGSFSPQEAALIIE
LHSILGNRWAQIAKHLPGRTDNEVKNFWNSSIKKKLQLSHGTFSDLSIIFPNNNNLIANPNPNPSFDNSNSFNNPNVPIT

NSFMSSTNNSSPLIQQLDQMINFPNGDLNLINQVVPYSLPMSFDSMSNDPTWFNFNYPHHLQHDLEYNPSVIKQDNSNSM
FTSNISLNCTNGENFMDSTNADIKIHQDLMMAPLLLPKLSETIKGNEGGILESTPVASQYIHSMAISSLPCTIPTGYTMN
PHNNNNVHRVVHEMKQIESVHMSSSPSSSKSSASGSSSLSALSCSQFMMNPSGNLPTTWDS

>CcMYB44 |Locus_ID=BC332_00146 |Protein_ID=PHU28053.1 |Protein_len=338 |
chr01:2327678-2330911 |strand= -
MGHHSCCNQQKVKRGLWSPEEDEKLIRYISSHGYGCWSEVPEKAGLQRCGKSCRLRWINYLRPDIRRGRFTPEEEKLIIS
LHGAVGNRWAHIASHLPGRTDNEIKNYWNSWIKKKLKKPSKPSANTTSITDHHHQQSHQRSQLVNYNTITSQQDIFFTQD
LGTKSQVLLQDSTLFNSPSQLFFFDSGSLDSMTNAIINDATNRSTTTNNASLFQETSTLNSEFCWQVDQQQQQQLQVQTS
SYAIGMDSNYLPPLIESMVPPMEIPRNNNNNNNIMVEGQENNNQLDEWSSQVDTQQCCPSYLFWDQENGSIGGDHEINID
PTTSNNMGQILSSFPSSL

>CcMYB45 |Locus_ID=BC332_08924 |Protein_ID=PHU23817.1 |Protein_len=332 |
chr03:247287194-247288480 |strand= -
MGRHSCGVKQKLRKGLWSPEEDEKLSNYITNFGIGSWSSVPKQAGLQRCGKSCRLRWINYLRPDLKRGMFSQDEEDRILG
LHQVLGNRWAQIAAQLPGRTDNEIKNYWNSSLKKKLMKQGIDPNTHKPLRENQVIKDEENCTNKTSMFEVPFLNEMDQPF
TQLASSKDQYVSKQVFDLLFAHEFQSNENNSQTLAQYHDHHHQSEYEENPNYVFCSASSVSKLEHGQMTDTDFASSSTSR
MSSSNSSNMCSNQNTARIRTENSEALSWDVENKMESLFQYPYIGIKNEELRSSPSQERELYGHSSGDFMSNYPLSSLTEE
LSGANLDVFKQI

>CcMYB46 |Locus_ID=BC332_16679 |Protein_ID=PHU15474.1 |Protein_len=320 |
chr06:222540301-222541966 |strand= -
MGRHSVYVKEKLRRGLWSPDEDEKLYNYITRFGVGCWSSVPKLSGLQRCGKSCRLRWINYLRPDLKRGTFSLEEEDMIIT
LHEVLGNRWAQIAAKLPGRTDNEIKNYWNSYLKKKLIKQGIDPNTHKPLSENHQVRNDTNCTDKASMLLMSTFPNMSNSV
EIEQPFHFNKRIFNSEAITRQLTEVSRNQLVSKQVFDPLFLYDFQANMNPIGPYDGNQDFGICSMPNLTNFEHGHMTESD
FSDSSTSRMSTSNRTNTMIRHHSSAGIHQMNEMLENSQELTWDADNKIDSLFEYPYDGIKNEEDFSNNNNNNNNPLSSLL

>CcMYB47 |Locus_ID=BC332_22066 |Protein_ID=PHU10206.1 |Protein_len=420 |
chr08:183068022-183069543 |strand= -
MGRHSCCYKQKLRKGLWSPEEDEKLIKHITKFGHGCWSSVPKLAGLQRCGKSCRLRWINYLRPDLKRGTFSQDEENLIIE
LHAVLGNKWSQIAARLPGRTDNEIKNLWNSSIKKKLRQRGIDPNTHKPLSEVESEEKASANSNNKNNDKVSESSNNEFNF
VEAHENGFSTEKGINNMERYPLIDHQANNIAPPTHEFFTANCKSPDLSSYLSFHNYSPNTNILFNTKNSSADNMVSDHHQ
FNCSTLPNATFSTMSSNSILSTSPLARTFNINKFQNWEACTISSNGSNNSNGSSNSIELQSNCSFFDNSNAAAAAFTWGS
TSAAAAADGSGKQEREEIKWSEYMQTPFSLGVNNNTIQNHHHEISPHHQDLYDGETKSETQFMTHGTWLQNQTPQTSLQT
ADLYSNNNFQRLPAVFGQFS

>CcMYB48 |Locus_ID=BC332_25434 |Protein_ID=PHU04612.1 |Protein_len=222 |
chr10:61725009-61726632 |strand= +
MAQEEIMRRGPWTEEEDLQLVFYVKLFGDRRWDFLAKVSGLKRTGKSCRLRWVNYLNPDLKRGKMTPQEERVVLELHSKW
GNRWSRIARKIPGRTDNEIKNYWRTHMRKKAQEQRKKSSISPSSSFSNSSYSSSISHEENERNFYDTGGLELLKAEGEMK
SSDQEQGGESMKIYSMDEIWKDIELLEENEETTNKPIMGSISPLWDYSLDSLWKDFDWSSFR

>CcMYB49 |Locus_ID=BC332_11766 |Protein_ID=PHU20615.1 |Protein_len=330 |
chr04:220728386-220729916 |strand= +
MGRAPCCDKNNVKRGPWSPEEDAKLKSYIEQNGTGGNWIALPQKIGLKRCGKSCRLRWLNYLRPNIKHGGFSDEEDRIIC
SLYISIGSRWSIIAAQLPGRTDNDIKNYWNTKLKKKLFGKQRQKQGSRKGKEIKSMSSISTSNNLNQNPCWPELQPLPYS
NNEPRFINNDHTSIRKLLIKLGGKFSENDQLTNVVPNSHEYPIDNSSSMQLMYQDHINLISSAPIDHNVFNITAPNIDLY
NMDGAASNFTAEFEHMINTSQQKIDGLEFLYENLVIDKFTSTSGGNLDWESMNPYVLPLPPIVASSDGGFQQGVIFQQGA
LDELRYPMVQ

>CcMYB5-1 |Locus_ID=BC332_08856 |Protein_ID=PHU23749.1 |Protein_len=185 |
chr03:246216986-246219064 |strand= -
MGEAPSYCSLKNYDSKNEDHYKQIVGCFDEDFHDMKKDLWTEEEDRILIEAHAEIGNKWAEIAKRLTGRTENSIKNHWNA
TKRRQFTRRKCRTKWCSPSSILQNYIKSLNFEKGNNNSAPKIEPIDQQVPADNYDFGEFDLEDKLFDDLENVPNYCELAQ
LIMQDAEVNKEVDLMDFISPNPENV

>CcMYB5-2 |Locus_ID=BC332_08857 |Protein_ID=PHU23750.1 |Protein_len=228 |
chr03:246226646-246227429 |strand= -
MDFDPSSKQDVFCTIPTDYLHDFHHLDLTFSLAGGPLTFNTNPDHMIEMDSGYEHPLFDPLSNHGDNFSSMGQDFNFGYE
FKPFEQNGNASGSTLFMKNFESSTDMKSINFNVVRDESSCVTSTIENNGTNNVYNDKEIGGKKNKKLIKSNDIKSSSSSS
MRKSGRGTRKKSKSAKDQWTIEEDRILINLVEKFGDRKWSEIAKMLKGRIGKQCRERWNNHLRPDIKV

>CcMYB50 |Locus_ID=BC332_17980 |Protein_ID=PHU11050.1 |Protein_len=342 |
chr07:6396306-6398866 |strand= -
MGRAPCCDKANVKKGPWSPEEDAKLKAYIEENGTGNNWIALPQKIGLKRCGKSCRLRWLNYLRPNIKHGGFTEEEDNIIC
SLYISIGSRWSIIAAQLPGRTDNDIKNYWNTRLKKKLLGKRKQSQMNRLLLANGGQDLKELNGLEENPLLQNLSNSALER
LQLHMQLQTLTNPLSFYNNPSLWPKLTPLQQKMIQTLQVNGLNEDQPPLYAPINPSENHAQELGQKVGMNEFASTNFKVN
DQVEKSSPIDVLDTNVGQENTRDQIQIQGVQGFTQLEIDDLILSNKGSIGMNMNSEFDLAWWSNEFDTNRASASNSWGSS
SNILQNTHDEMYQDYALGYNLQ

>CcMYB51 |Locus_ID=BC332_22966 |Protein_ID=PHU06477.1 |Protein_len=274 |
chr09:5574868-5577256 |strand= +
MGRAPCCDKANVKKGPWSPEEDAKLKEYIDKFGTGGNWIALPQKAGLKRCGKSCRLRWLNYLRPNIKHGEFSDEEDRVIC
SLYANIGSRWSIIAAQLPGRTDNDIKNYWNTKLKKKLMGFVSSSHKIKPLNHHHYQHQIPTNYNYNNYSPQISLFQTSSL
LISSNYNTNTNNTTFPCYDINAPISNFLSNGASTSCTSGITTSTFQENNFVGPTATSSDESYGVSNLDFRTYMYNNGVIG
EEGEDQGNSKFLISGNNDGGGCYVDEQQNPLDYS

>CcMYB52 |Locus_ID=BC332_17052 |Protein_ID=PHU15847.1 |Protein_len=333 |
chr06:230366114-230369066 |strand= -
MGRAPCCDKNNVKRGPWSPEEDAKLKEFIDKYGTGGNWIALPQKAGLKRCGKSCRLRWLNYLRPNIKHGDFSDEEDRIIC
SLYANIGSRWSIIAAQLPGRTDNDIKNYWNTKLKKKLMGFIHTSSNQRIKSPNLFLPPTTFPPQIQSQAISNIFKDEYLE
PISSLQPNFMYHNNNMNFQLGTNNQYSYFLESLMNPMQASCSSSDGSCSQIPRSNFEETQKFTLDNYGNNGGTWADHDQK
PDGYFGNNHQNHSSQFQYDTSNIEEVKELISSSSSNGNGCNNNSNVLFFSDENKANIGAVPALTRGRGFDPEYGENSVGS
AATLMGPVKRNPD

>CcMYB53 |Locus_ID=BC332_05620 |Protein_ID=PHU27288.1 |Protein_len=330 |
chr02:160525726-160527115 |strand= +
MGRAPCCDKANVKRGPWSPEEDSKLKAYIEQHGTGGNWITLPQKVGLKRCGKSCRLRWLNYLRPNIKHGEFTDEEDTIIC
TLYMSIGSRWSIIAAQLPGRTDNDIKNYWNTRLKKKLLLCNKQRKDHRPRTGYNHNKVDMMKEQEDFFATHQTTNNAYSW
SPQQILFSTLIAPQNHDVAECSANSHNFQYSSTDQAYFSQDQLCQISSTNHLASMNPVNGATCNSSMMVSSGAYYPSNGF
CNNGLQEYNNYISVGLDHDVLNSTSTAHSQQVDKSVLEMVNSSTISTTSQDQSTSWEELSPLVNYPPSVFETVSPYYVFE
EPRYLGLLKQ

>CcMYB54 |Locus_ID=BC332_24646 |Protein_ID=PHU08157.1 |Protein_len=268 |
chr09:255698513-255701462 |strand= -
MGRAPCCDKTKVKRGPWSPEEDNILKNFLEKNGTGGNWIALPQKAGLRRCGKSCRLRWLNYLRPDIKHGGFTEEEDNIIL
NLYNQIGSRWSVIAANLQGRTDNDVKNYWNTKLKKKLLASQNTNIISTNNNINSSDFNHINYSRNYYGTLDYSNTFTSHM
DPNLTNYSNNCDQCPLLPSLVEIQENDATIQSSESCQILQKCATLKESDTHPSTFLQNPNNIGYNYYDDILENGFDFQEN
AIGGANPNSSYYNNILEIDDQLFKGFEN

>CcMYB55 |Locus_ID=BC332_07302 |Protein_ID=PHU22195.1 |Protein_len=100 |
chr03:51100152-51101088 |strand= +
MGRTPCCDKTKVKRGQWSPEEDEILKNYMLKHGTAGSWITLPHKAGLNRCSKSCRLRWLNYLRPDIKLGKFTQEEDNIIH
SLYSQLGSRLNLALQPLFIS

>CcMYB56 |Locus_ID=BC332_24914 |Protein_ID=PHU04092.1 |Protein_len=340 |
chr10:3706345-3710034 |strand= +
MGRIPCCEKDNVKRGQWTPEEDHKLSSYIAQHGTRNWRLIPKNAGLQRCGKSCRLRWTNYLRPDLKHGQFSEAEEQTIVT
LHSVLGNRWSVIAAQLPGRTDNDVKNHWNTKLKKRLSGMGIDPVTHKPFSHLISEIATTLSPPQVPHLAEAALGCFKDEM
LHLLTKKRIGFQFHQQFPNNAPSTSHVKNEENDTVEKIKYGLSRAIKGSDKNWETNGGATSSSFHDNGGYSNYNFANLLN
DEDGSPWNQSLCSGSTCTAGGELQQQQQQQRNKKTSNGNCEDSGKEVVTTRNGSTTTMFNSDCVLWDISSDDLMNPMVLR
GSSQGERKLTETPESFKKSL

>CcMYB57 |Locus_ID=BC332_10902 |Protein_ID=PHU19751.1 |Protein_len=332 |
chr04:104210982-104213348 |strand= -
MGRPPCCDKANVKRGPWTAEEDAKILAYVASHGIGNWTLVPQKAGLNRCGKSCRLRWTNYLRPDLKHDNFTSLEEECILE
LHKTIGSRWSLIAKRLPGRTDNDVKNYWNTKLKKKLVNMGIDPVTHKPFAQVFAEYGKISGLPIQNAKNHIHLPNNTTEK
NHVCMESKQFPFSNGTPHDKFVHKIMNDQLQENYTKNYTWDSKETLQTHSSLLISTSTSCNPTVHSYSPSFSSVQSQVHT
MPASTSTCNWNEFIFGDPFTSSGAEQKQETFLSNLLQKENPTCGEVTEVEEKQLVEEATCSSAMDSFVDTILANEKQMLM
DFPPLLDVYLDY

>CcMYB59 |Locus_ID=BC332_07716 |Protein_ID=PHU22609.1 |Protein_len=250 |
chr03:147200400-147201445 |strand= -
MEVIIIKKGAWSPDEDQKLKSYIMRYGIWNWNLMPKFAGLSRTGKSCRLRWMNYLRPEVKRGPFTIEETETVIKMYQELG

NRWAAIARRLPGRTDNEVKNFFHTHLKKHLGLKIDQNASVKLCKAVRRKRVLEKTKKSDKKNLAQEKPVLYLDISGTSSN
KSSFTISPSGSSSSSSIITIDQNQMTDIHVDQPNSIIDDMETTVLILESNPAISTHHDQSLDSLSIDSLYQFDTSSFWLH
LLNDAHRLIF

>CcMYB6 |Locus_ID=BC332_16696 |Protein_ID=PHU15491.1 |Protein_len=427 |
chr06:222783269-222786007 |strand= -
MELDQSNNQNLSIHQPFFMPNNNILKPEIDDDFCIDSKDYYLKDFEHLDLNFSNTTFNNFSQNFMIDDDTNGTTYHDPFD
PFSNIEDNFSSLGDFNFSSHELTPFDQENGESGGSKVMKNNFHDDEFMKPNLNFVNVPDDQSSCVTGDNNLSSSIKKKLG
GRGRSKKSSKSSKGQWTIEEDRLLIHLVEKFGIRKWSQIAQMLKGRIGKQCRERWHNHLRPDIKKDFWTEEEDRILIEAH
GEVGNKWAEIAKKLPGRTENSIKNHWNATKRRQFSRRKCRTKWPRPSSLLQNYIKSLNLEKGSTKKDTNPNGRMEYTGSV
AENRNENLANRPKRHANIAKVKVSPKLEAIEFCQDNNAGDFDLSEVPEFALGDNFFDENKSMDAFMEEINPNVGTSIMPC
NYEMPTLMMQGEVNKEIDLIEMISRVN

>CcMYB60-1 |Locus_ID=BC332_03169 |Protein_ID=PHU24837.1 |Protein_len=200 |
chr02:214943-215803 |strand= +
MFSKSPELWSHVVAGLSRTGKSCRLRWMNYLHPDVKRGPFTMEEVETVIKTYQELGSRWSAIATKLPGRTDTEVKNFFHT
HLKKHLGLKNYDAQLKTRKGHKQTQENEKENSTQGRLVLETINSSSSLSPDVCSPCSSSITCKENQMMDQFTNFSLVVDQ
QVSHVENTNIQSCFIPGGSTFSTFDQFDMNSFWFDVLGNF

>CcMYB60-2 |Locus_ID=BC332_07717 |Protein_ID=PHU22610.1 |Protein_len=197 |
chr03:147870740-147871891 |strand= +
MLRVPQQQQQKGLSRTGKSCRLRWMNYLHPDVKRGPFTMEEVETVIKTYQELGSRWSAIATKLPGRTDTEVKNFFHTHLK
KHLGLKNYDAQLKTRKGHKQTQENEKENSTQGRLVLETINSSSSLSPDVCSPCSSTITCKENQMTDQFTNFSLLVDQQVS
EGENTNIQPCFIPSGSTFSTFDQFDMNSFWFDVLGNF

>CcMYB61 |Locus_ID=BC332_19187 |Protein_ID=PHU12257.1 |Protein_len=229 |
chr07:200204999-200206373 |strand= +
MVRTPIIDKNGIKRGAWSEDEDNKLRAYVERFGHPNWRQLPKYAGLMRCGKSCRLRWMNHLRPGLKKGNYRLEEEQLIIK
LHKELGNRWSVIAANLPGRSDNDVKNHWHAHLKKRVRLTNCINNSSMSMEQFTESTQSGSSQLDERSASMNVVVDTSPTL
LPISFPEVSSSDLYSITDWIEEESMEQLPILSSMELLAADTFSWTNYPIDNFQTESFDHFWTQPFDTFF

>CcMYB63 |Locus_ID=BC332_19183 |Protein_ID=PHU12253.1 |Protein_len=247 |
chr07:199344861-199346752 |strand= -
MVRTPCCDENGRKKGTWTPEEDRKLAAYITEYGSWNWRQLPKYAGLERCGKSCRLRWLNYLRPNVKRGNYTEEEDEIILN
LHAQLGNKWSAIAAHLPGRSDNEIKNHWHTALKKRANNSGESSKKCNSKNNIRRSTSVANKNASPSTSHENIVLESSQWS
PKESSSEDFFSYSNTDYQEQHKVFQQEALDEITSGSFWTEPFLVESFNTTGTDFLAPPIDYCGLVCPPSPFIGHEFLSSF
DFDHFNY

>CcMYB64 |Locus_ID=BC332_19182 |Protein_ID=PHU12252.1 |Protein_len=137 |
chr07:199271585-199272584 |strand= -
MVRTPCHDEKGRKKGTWTPEEDRKLAAYITKYGSWNWRQLPKYAGLARCGKSCRLRWMNYLRPNVKRGNYTKEEDEIILN
LHAQLGNKWSAIAVHLPGRSDNEIKNHWHTALKKRANYVSTSSDVSSKKSNKKKNSG

>CcMYB65 |Locus_ID=BC332_19186 |Protein_ID=PHU12256.1 |Protein_len=248 |
chr07:199992922-199998903 |strand= -
LARCGKSCRLRWMNHLSPNVKRGNYTKEEDEIILNLHAQLGNKWSAIAAHLPGRSDNEIKNHWHTTLKKHATNNATNSSS
DKSSKKCSNNKNKNTKSNASKDASPNNNMHENVILESSEWSPKESLSDQELISSDSTSVDYQNDVFQEENLVEITSGSFW
TEPFVVESFNSPRIDFLGPPSIDYGLVCPPSPFIGEGFVSSFDLDDYNCGRIVEEAEDGKKQHTKKKEKTKKMNRKKKKK
KQTKKMWS

>CcMYB67-1 |Locus_ID=BC332_06786 |Protein_ID=PHU21679.1 |Protein_len=278 |
chr03:18458084-18460142 |strand= +
MVRAPCCEKMGLKKGPWTPEEDQILVSYIQANGHGNWRALPKLAGLLRCGKSCRLRWTNYLRPDIKRGNFTREEEDSIIQ
LHEMLGNRWSAIAARLPGRTDNEIKNVWHTHLKKRLKNYQAPENSKRHCKKNIDSKAPSTSQITLKSSHNFSNIKEGING
PDPNSPQLSSSEASTVTADSESVMTMGVTNGGDQMDSYENFIPEIDESFWTDDLLSTADNSSFDMAISGGEELQGQFPFY
DMKQESVELDVGAKLEDDMDFWYNVFIKSGDLLELPEF

>CcMYB67-2 |Locus_ID=BC332_10106 |Protein_ID=PHU18955.1 |Protein_len=110 |
chr04:4180528-4182538 |strand= +
MVRAPCCDKTGLRKGPWAFEEDQILISYIQKYGHGNWRALPKQAVVTSDSSSSIIAFGVTIDNTIAVKLEESDSSEYLTE
IDESFRTDDDMDFLFNFFIKAEDFSELPEL

>CcMYB68 |Locus_ID=BC332_15553 |Protein_ID=PHU14348.1 |Protein_len=280 | chr06:140486325-140491524 |strand= -
MGRAPCCEKMGLKKGPWSPQEDNILISYIQNNGHSNWRALPKLAGLLRCGKSCRLRWTNYLRPDIKRGNFSKEEEDTIIQ
LHENLGNRWSAIAARLSGRTDNEIKNVWHTHLKKKLKNYQPPQNSKRHFKTNHDSKKGPTTESSNNSDFSTSANNTNTHK
KHMKIAPNSPVLSSSEMSTVTLVVDDRQRVIIKEEKIESPLEYFPKIDESFWTEELSAEKNNDNDYVMAAGLDQDIQAQI
QLSSVKEENLDNTSTKMEEDMDFWYNVFIKTGDFLELPEF

>CcMYB69 |Locus_ID=BC332_17589 |Protein_ID=PHU16384.1 |Protein_len=283 | chr06:247867460-247869081 |strand= -
MGRTPCCEKLGLKRGPWSKEEDDLLINYINKNGHPNWRALPKLAGLLRCGKSCRLRWTNYLKPDIKRGNFTNEEEDTIIK
LHQVLGNRWSTIAARLPGRTDNEIKNIWHTRLKKKISKSHAQKIPELQEETSMETSKSDEQNLDTHLEPENSNLDIHLES
DNSKDNCEISSPKTNNEIQQQSSSSVHSSSSISSSSEDACSNTTATSPEPRDQFMLDNLVEIDDDFWSDVLWTTVDDSNY
NDNNFDLPLMEDNYQINPSLNDNWFWDDLFTRSNELMLELSEF

>CcMYB7 |Locus_ID=BC332_17369 |Protein_ID=PHU16164.1 |Protein_len=341 | chr06:244058071-244059809 |strand= +
KLLRLVKQFGVRKWAQIAEKMGARAGKQCRERWHNHLRPDIKKDTWSEEEELLLVELHKLLGNKWAEIAKRIPGRTENAI
KNHWNATKRRQNSRRNKNKKTQQADGTQCENKSHRSTVLQDYIRSKYFMNDDNSPLTATLYYGSGGTASGGGYSPSTTTF
IGSTSNATNATPTYSDDDSPYLITHQTYDEEMNFMQSLFGNSNNINGTKVMGTMETNVVTQDLLDKSSSSSSFGLNSSGE
IPLAECNVESYTTTKRVIADNNARNYIPDYSQYYPQDLYLSRLLDGSLVPNYSDCFECGSMNNQAGCSSSGGNIKEVDLM
EMMNSTNSQFSQPGTFNKTFF

>CcMYB70 |Locus_ID=BC332_14283 |Protein_ID=PHU18588.1 |Protein_len=147 | chr05:232898517-232898960 |strand= -
MFFFFRWSAIASRLPGRTDNEIKNFWHTHLKKRLEQHNIDLASTTTTKQNHEMTTNVTQNIDHHIISPNYYQDSQNMVTY
HPTYCHDHQEDFQENNSSSHDHTQSNKEEIMQYSTNYGHEDMTSFNNDMVFWYNVFMSSGNNPSEIS

>CcMYB71 |Locus_ID=BC332_24933 |Protein_ID=PHU04111.1 |Protein_len=225 | chr10:3918996-3919985 |strand= +
MGKGRRSCCDKSEVKKGPWTPEEDLKLISFIQKHGHGNWRALPKQAGLVRCGKSCRLRWINYLRPDVKRGNFTSQEEDTI
INLHRSFGNRWSKIASHLPGRTDNEIKNVWNTHLKKRLLLMKKEEHNSPSSTPTCSSTTLGFCSPTSSKANDQVIDFWEY
MLDTSNPTSINNLDHFDSYSKQDTTSTNTEHHQPQMADEYECQKWLTYLEIELGLTTNEAQHQIK

>CcMYB72 |Locus_ID=BC332_20160 |Protein_ID=PHU13230.1 |Protein_len=295 | chr07:233361071-233362589 |strand= -
MMGKGRAPCCDKSKVKKGPWSSEEDIRLIAFIQKHGHQNWRSLPKQAGLLRCGKSCRLRWINYLRPDVKRGNFTPQEEET
IIKLHQSFGNKWSRIASHLPGRTDNEIKNVWNTHLKKRLMKRLHGDKAKLDGTCANSQSSSSSTSVVSHTDDTSNEGKFK
NVDANMIIENATKEEEATKSCSPTSSYASNLSQVHEASSPNNVDMDWIFQFSEEPFPSTTARKVHDDDDEGIIEIPLECD
VDFWDMLDIMGPSPPPPPPPNSESKLDHHSEASCQGLSEFERQKWFTYFERELGV

>CcMYB73 |Locus_ID=BC332_21478 |Protein_ID=PHU09618.1 |Protein_len=310 | chr08:169412994-169415439 |strand= +
MGRTPCCDKNGLTRGPWTPEEDEKLVEFINKNGHGSWRSLPKLAGLLRCGKSCRLRWTNYLRPDIKRGPFSPEEQKLVIQ
LHGILGNRWAAIASQLPGRTDNEIKNLWNTHLRKRLLSMGIDPQTHEHHSDPNGLVRRPATSSLARHLAQWESARLEAEA
RLSRESQFLVPSSVGRSETDYFLRIWNSEIGESFRKFKKGEKSACHSPASQASSCTKYGSASGITTEVEIGVAGSPVTGS
NQNECMEWKIGQPYTEDFLQGADTCSSNAMDEDSSESALQLLLDFPSNNDMSFLGHSDSYSLYPFLSESY

>CcMYB74 |Locus_ID=BC332_00519 |Protein_ID=PHU28426.1 |Protein_len=277 | chr01:9886795-9888950 |strand= -
MGRQPCCDKLGVKKGPWTTEEDKKLINFILTNGQCCWRAVPKLAGLRRCGKSCRLRWTNYLRPDLKRGLLSDAEEKMVID
LHARLGNRWSKIAARLPGRTDNEIKNHWNTHIKKKLLKMGIDPVTHEPLIKEENPTDQPIQESDQNKNNDHQQVVVVTAG
ATSSELDNSSTSSSSASSSENSCNESKLVLDTFKENDPLLSCLLEADDAPPLIDSSWELPVKSFDNNNNMTSSWDDESFN
WLFDCQDFGIHDFGFDNCFNDVELEVFNTIYDMENKK

>CcMYB75 |Locus_ID=BC332_12501 |Protein_ID=PHU16806.1 |Protein_len=319 | chr05:4827474-4829916 |strand= -
MGRQPCCDKNGVKKGPWSSEEDKKLINFILNNGQCCWRSLPKLAGLLRCGKSCRLRWTNYLRPDVKRGLLSEYEEKMVID
LHAQLGNRWSKIASHLPGRTDNEIKNHWNTHIKKKLKKMGIDPITHKPITSDQPTYDQPIIQQEKQTLIAPPSQTDLNRQ
GNENTGTCPKNIKTTTTATTTTVAMPSSETNLDRQENKNVGTCPRHIKTTTTTTTVVTPQSETNLDRQKVPLIPPTEILI
PYESTTSSTSSFSSQSSNMILEDLKFLPNFDNWQSDFNMEINWTNDDFSSTLDYLLNDDDISDMNNTTISQDWSQVLEV

>CcMYB76 |Locus_ID=BC332_29469 |Protein_ID=PHT99681.1 |Protein_len=303 |

chr12:6595886-6597904 |strand= +
MGRQPCCDKVGLKKGPWTTEEDKKLIHFILNNGQCCWRAVPKLAGLLRCGKSCRLRWTNYLRPDLKRGLLSEYEEKMVID
LHAQLGNRWSKIASHLPGRTDNEIKNHWNTHIKKKLKKMGIDPVTHKSLSTITNDQTNILEQENQPIQQEKVEDQSTMIE
IKAEDDNNNSNNNNSNNDNNTMETSCANNFNSTIVEVNNNGFGIDEVPLIEPHEMLVQELSSTPSTSSSSSSFSSSSSSN
ILEDLKFLPSFDEWPLMENNNMGFGWEINNDFTSTLDFLLEDDHSDMMNNVTFDESWKFEQLL

>CcMYB77 |Locus_ID=BC332_06135 |Protein_ID=PHU27803.1 |Protein_len=255 |
chr02:168560272-168561366 |strand= -
MGRQPCCDRVGLKRGPWTIEEDHKLVHFILSNGIQCWRTVPKLAGLQRCGKSCRLRWINYLRPDLKRGALSEAEEDQIIQ
LHARLGNRWSKIASYFPGRTDNEIKNHWNTRIKKRLQLMGIDPLTHQPIDMEKYQHEPDQTEKCDKRNEPENFIENTNDV
SLPNNPHHNMLSDSFNVDLWNKSGNTMSTCYSPSISLEAEESINLSTTTSAATESSSNVAALTEEQDSLQQWMDSIFSCA
VNQLEEDLIFLRKYN

>CcMYB78 |Locus_ID=BC332_28218 |Protein_ID=PHU02967.1 |Protein_len=224 |
chr11:103211038-103212268 |strand= +
MGRQPCCDKIGLKRGPWKIEEDHKLMNFILNNGIQCWRLVPKLAGLMRCGKSCRLRWINYLRPDLKRGALTEAEEDIIIK
LHSHLGNRWSKIAAHFPGRTDNEIKNHWNTRIKKKLKFLGIDPLTHKPIEQNDDIKQQVSVEIDENVKELAEKTLDYPRE
QQNIQNSTSSSSLNESLDMGSSSEIFQNSHTLDMYNPGVEDPFQNWIGSPIHWDLFDNLEDNFL

>CcMYB79 |Locus_ID=BC332_10962 |Protein_ID=PHU19811.1 |Protein_len=347 |
chr04:124481192-124482690 |strand= -
MGRSPCCDENGLKKGPWTPEEDQKLVDYIDKHGHGSWRALPKLSGLNRCGKSCRLRWTNYLRPDIKRGKFSEEEEQTILH
LHSILGNKWSAIATHLPGRTDNEIKNFWNTHLKKKLIQMGYDPMTHRPRTDLFASLPNIIALANLLQHHPLEDQAIRLQA
EAAQIAKIQYLQFLLQSSNNNYNPIPPTTSSNTQYYNNLGDIGAFNLTNFVKENSTTPSLNLSNLENQTLFSNETGSQLL
HNPDNTQQVPFNFQTHLNNNNNNDMSDIDGNNFNFDLEDNSPSSPLNNTIIPSPPSSSHLPHLTEISISNNNQGDSSCNS
SNNVADQGTSSSYWPELFFEEHFMHDI

>CcMYB8 |Locus_ID=BC332_08524 |Protein_ID=PHU23417.1 |Protein_len=248 |
chr03:237317481-237320769 |strand= -
MCSRGHWRPHEDEKLKELVAKYGPHNWNAIAENLQGRSGKSCRLRWYNQLDPRINRSPFTEEEEERLLSSHRIHGNRWAM
IARLFPGRTDNAVKNHWHVIMSRRCRERSKIYSNRASAAAAAQKSTDQQDNINNIQDMMIRKRSSNNFIDHQQLLERYSN
CYQFSSYDHNSFYPTEFHFNHLPRHFKMDNQEKNEEVECYDFLQVNNTAGSNKSEVIDQEVEQKRDISPQSKKAPVRFID
FLSVGDSS

>CcMYB80 |Locus_ID=BC332_13875 |Protein_ID=PHU18180.1 |Protein_len=351 |
chr05:217058715-217061169 |strand= +
MGRSPCCDESGLKKGPWTPEEDQKLTNYINKNGHGSWRALPKHAGLNRCGKSCRLRWSNYLRPDIKRGKFSQEEEQTILN
LHAVLGNKWSAIATHLPGRTDNEIKNFWNTHLKKKLIQMGYDPMTHRPRTDIFNSLQHLIALANLKELIEHNHSWEEQVM
RLHYLQNLLQQPHNNNISTLSAIQNVEANYNLLNSLKDSQFLDSNSHLGNHIVHQIPSSLDQPIQNSIPFSHLTELHTPC
SFQTSLNKDKVPPQDIEFTVMSHEETSPASSWLPPLSPSLPPPPPPVSNNQRRKENSSEVVISSCLPEDMKVLDNFFQQD
SEQSLSNEGAPPSIWSDLPLEDSFFQGLDKF

>CcMYB81 |Locus_ID=BC332_00728 |Protein_ID=PHU28635.1 |Protein_len=299 |
chr01:16594255-16596304 |strand= -
MGRYPCCKLENDLKKGPWTSEEDEKLVEYIQENGHGNWQLVPKRAGLNRCGKSCRLRWTNYLRPDIKRGAFSNEEEETII
NLHSLLGNKWSRIAARLPGRTDNEIKNLWNTHLKKKLLKSGIDPVTHQPITDPNLLLSLSNLMNPFESALRLQAEFTEIA
KIHLIQNIIQVLNTPLAPLIPSLQENYPMQQFYNNILAPFDQILANEPSPISKNLSDNMVVNSSILENNMINNGYSKVCE
VDNSEYSSLPSLVTITPENSVPFDQKIDIINPSYDDFEALEKSLDDDFWKDISSTMQFV

>CcMYB82 |Locus_ID=BC332_00729 |Protein_ID=PHU28636.1 |Protein_len=215 |
chr01:16770190-16771372 |strand= -
MGRYPCFKVDEDLKKGPWTPDEDKKLMDYITNNGHTNWQLIPKKAGLNRCGKSCRLRWTNYLRPDIKRGAFSFEEEEIII
SLHSILGNKWSRIASHLPGRTDNEIKNFYNTHLRKKLLRLGIDPRTHKPISDLNFLINLSHQFPSKHNLFINNPLAYALK
LQAQVTEIAKLQQLLQDLYAPINTNIPLLPSNFQENNFATSSSHVITTNVSTNTH

>CcMYB83 |Locus_ID=BC332_19190 |Protein_ID=PHU12260.1 |Protein_len=337 |
chr07:200612980-200614206 |strand= +
MGRSPCCDKNGLKKGPWTPEEDNKLIQYIQLHGPGNWRTLPKNAGLQRCGKSCRLRWTNYLRPDIKRGRFSFDEEETIIQ
LHSVLGNKWSAIAARLPGRTDNEIKNYWNTHIRKRLLRNGIDPVTHSPRLDLLDLSSLLNSTQFNLSSLLGLQALINPEV
FRLATTLLASHNESSPELLLQKLQENNQMFSNSQLQNQEGSELLLQKLQQNQLLNAQVQNQPQFFQYQNQIQEIPNFTTT
QNVACSSSLSQPMQLLGHVDESYLMNGQMLPPQNYGYCNSDASDNSTFQSLNNNSSNQNFSLDNSVLSTPTEDEKESYCS
NLMKFEIPESLNFDDFM

>CcMYB84 |Locus_ID=BC332_25152 |Protein_ID=PHU04330.1 |Protein_len=329 | chr10:12669439-12671617 |strand= +
MGRTPCCDKNGLKKGPWTPEEDQKLIQYIQVHGPGNWRNLPKNAGLQRCGKSCRLRWTNYLRPDIRRGRFSFEEEETIIQ
LHSVLGNKWSAIAERLPGRTDNEIKNYWNTHIRKRLLRMGIDPVTHSPRLDFLDFSLLFNSTQLNLSSLLGLQALVNPEI
FKLANSILLSQTHENQELSLQNQNSQGPELLLQKLQQNQLLHNQSSIFQHNDQLHNQISKIPNYTPQNVSSSSSQSIQGN
MGSYLMNGQAFHENVISLQNYSYSASEVSENSTIQSLNNNNNNNNQNSQNFNFDTPLSGTEEEKESYCSNFNSFMKFEIP
ESLDFDDLL

>CcMYB85 |Locus_ID=BC332_04760 |Protein_ID=PHU26428.1 |Protein_len=381 | chr02:144150965-144152416 |strand= +
MGRAPCCDKNGLKKGPWTPEEDQKLIDYIQKHGYGNWRTLPKNAGLQRCGKSCRLRWTNYLRPDIKRGRFSFEEEETIIQ
LHSILGNKWSAIAARLPGRTDNEIKNYWNTHIRKRLLRMGIDPVTHSPRLDLLDLSSILNPSFYNNSSSLHQMNLSRLLG
HVQPLVNPEVLRLATSLLSSQRQNTNNSFMIPNNHQENQICQNQLPQMVQNSQIQAPIQDYPTCTTLSTTPCVPFSSHEA
QLMQPPTTNMEGFSSKLENFSNSTSCQVNDEWQLSNGMPTSNLTDDYFPLQNYGYYDPLPSDGSTLIQSNDSNNNNNFSF
QSTVLSNLSTPSSSPTPLDSNSTYFNNSSSTTTEDERDSYCSNMLNFDNIPNIWDTTNEFM

>CcMYB86 |Locus_ID=BC332_25248 |Protein_ID=PHU04426.1 |Protein_len=321 | chr10:22814177-22815897 |strand= +
MGRTPCCDKNGLKKGPWTTEEDQRLIDYIQKHGSGNWRTLPKNAGLQRCGKSCRLRWTNYLRPDIKRGKFSFEEEETIIH
LHSILGNKWSAIAGRLPGRTDNEIKNYWNTRIRKKLMRMGIDPVTHSPRLDLLDLNSIFNPSIYNSCQVINNNLSRLLGV
QSLVNPELLKLANSLLSSNHQNQNFLLGSNFQENQPFNSHVQNQLTSLCQANQIQNPIQNTSTCSDLNIQSVPFYSDTQV
MQQPNVEQLSSNVSTFSSQNSQQNEWKNFTFDSALPNLSTPSSSPTSLNSNSSTTSEEERESYCSMLNFDIPNILDVNEL
L

>CcMYB87 |Locus_ID=BC332_06008 |Protein_ID=PHU27676.1 |Protein_len=415 | chr02:166597434-166599474 |strand= -
MGRSPCCDKVGLKKGPWTPEEDQKLLAYIEEHGHGSWRALPAKAGLQRCGKSCRLRWTNYLRPDIKRGKFTLQEEQTIIQ
LHALLGNRWSAIATHLPKRTDNEIKNYWNTHLKKRLVKMGIDPVTHKPKNDALLSHDGQSKNAANLSHMAQWESARLEAE
ARLARQSKLRSSSFHSSITSQEFTAPSPSSPLSKPVVGPTRCLDVLKAWNGVWTKPMNDVSVANASAGISVTGLARDLES
PTSTLGYFENAQHISTSGIGGNSTILYEFVGNSSGSSEGGIMNNEESEEDWKGFGNSSTGHLPEYNKDGINENSLSFTSG
LQDLTLPMDTTWTAESLRSNTDQISPANFVETFTDLLLSNSGDGDLSEGGGTESDNGGEGSGSGNASENCEDNKNYWNSI
FNLVNNPSPSDSAMF

>CcMYB89 |Locus_ID=BC332_01852 |Protein_ID=PHU29759.1 |Protein_len=335 | chr01:102126900-102129773 |strand= +
MGRSPCCEKVGLKKGPWTPQEDQKLTYYIEKHGCGSWRALPTKAGLKRCGKSCRLRWINYLRPDIKRGKFSLQEEQTIIQ
LHALLGNRWSAIATHLANRTDNEIKNYWNTHLKKRLTKMGIDPNTHKPRSNFGSANLSHMAQWENARLEAEARLVRESKK
QQEEIILINNNINNYNNIHFGTNNLGTKNVLLPLQTKLPSLPCLDVLKAWEGGANWSNRPKIKKEKFFDNFPTSSTSLSN
NNLLLMVPSNNNNNNNILGAGLVNTCLVGAGSFLGNNINEISSYSSYPCLNDIQGFTQLESVVGSCQDDYDNNDNNYWNT
ILNSCTSLADGSPVF

>CcMYB9 |Locus_ID=BC332_26517 |Protein_ID=PHU05695.1 |Protein_len=344 | chr10:217396241-217398481 |strand= +
MNFLVPCVEQSPWSFNHEENLRKLKDEIEEENLVDMKKNNNNNNGGTKICVRGHWRPHEDSKLRELVAQYGPQNWNLIAE
KLDGRSGKSCRLRWFNQLDPRINRMAFSEEEEERLLTAHKMYGNKWSMIARLFPGRTDNAVKNHWHVIMARKHREQNSVY
RRRKSTSSNLQQIIAPNNNNNDSIIQIKSNNMQSDLTISSNNCNNNNIIVDDHQSASTCTDLSLTPSSSKPMFPPFLMTR
FTHHHHVEHQGFLDSNKGMLSRDEQEVKMDQKFLYGKEPIESYHHHNHQHYGCGNVSDTNSEISASESVANNMTNVKIY
GHQNEEEQDIKTIPFIDFLGVGAT

>CcMYB90 |Locus_ID=BC332_27735 |Protein_ID=PHU02484.1 |Protein_len=182 | chr11:25428714-25431671 |strand= +
MGRSKCCDKEGLKKGPWTHDEDQKLLSFIEKHGCGSWRSLPAKAGLQRCGKSCRLRWTNYLRPDIKRGKFSLQEERSIIH
LHALLGNRWSAIATYLPRRTDNEIKNYWNSRLKKRLTKMGIDPMTHKPKITNNLNGVASSKYVANLSHMAEWESARLEAE
ARLVQKSKDSKILFNNNKCQQH

>CcMYB91 |Locus_ID=BC332_27736 |Protein_ID=PHU02485.1 |Protein_len=345 | chr11:25454017-25455690 |strand= -
MGKSKCSDKEGLKKGPWTAEEDQKLLSFIDKHGCGSWRALPAKAGLQRCGKSCRLRWINYLRPDIKRGKFSLQEERTIIH
LHALLGNRWSAIATYLPSRTDNEIKNYWNSRLKKRLTKMGIDPMTHKPKVTNNLNGVASSKYVATLSHMAEWESARLEAE
ARLVRKSKDYEILFNNNNYNINRSTISQQLPYYQLPCLDILKAWQMSSTKLPTINDISAILLNNSRNNNLESSIPSTLHS
SETLFVNNNVPTTTTTTNVRDDHQIPPNLSTIESCFEDDQLQTDLPSFMQEFSGLFPEYYTQNSTNIQVDNLMGSCSGDF

EDNKLINWNNFPNYLVNSPIGSPVF

>CcMYB92 |Locus_ID=BC332_03833 |Protein_ID=PHU25501.1 |Protein_len=330 |
chr02:110224039-110226630 |strand= +
MGRLPCCDKVGVKKGPWTPEEDIMLVSYVQEHGPGNWRAVPTKTGLRRCSKSCRLRWTNYLRPGIKRGNFTDQEEKTIIQ
LQALFGNKWAAIASYLPERTDNDIKNYWNTHLKKKLKKFQLDQCSGSDDHLFYVENGLTHLSSSNSRGQWERTLQTDINM
AKKALHNALSLENSTSPLINIKQENPQVSSSTYASSTENISRLLQGWMRSSTSENSKTSSNNIATNDSSSCDGTPNAESK
GGMGLMEAFESLFGLESFESSSSDEFSQTTSPQAEATKVEIKKEENENVEAPAVPLSIILENWLLDENTLNIQAKDDLTT
FSFDETAPLF

>CcMYB93 |Locus_ID=BC332_06027 |Protein_ID=PHU27695.1 |Protein_len=304 |
chr02:166911270-166912677 |strand= -
MGRPPCCDKLGVKKGPWTPEEDIMLVSFVQEHGPGNWRTVPTHTGLRRCSKSCRLRWTNYLRPGIKRGSFTDQEEKMIIQ
LQALLGNKWAAIASYLPERTDNDIKNYWNTHLKKKLKKLEANDLYSSDGHCLSSIKSASRGQWERTLQADINTAKQALQN
ALSLDKSSPISEYTIKPINIDCYPSYIKQEAKMSTSTYASSAENIAKLLKQWTRSDSTNNSEQSKASSSTQFSCNTTNNT
NAMTEYSSSFESFEPSNSDQFSQATTPEAAGKFYGESKRELDDEVPLSVMLESWLFDENDNLLI

>CcMYB94 |Locus_ID=BC332_26534 |Protein_ID=PHU05712.1 |Protein_len=352 |
chr10:218195386-218197130 |strand= +
MNELIKNHLLLLFLSSNHLPLFKHLSICASSFSFQLSIHTSKTLHTKTTTTTTTPPPPXXXXKQTMGRPPCCDKIGIKKG
PWTPEEDIILVSYIQEHGPGNWRSVPTNTGLMRCSKSCRLRWTNYLRPGIKRGNFTPHEEGMIIHLQALLGNKWAAIASY
LPQRTDNDIKNYWNTHLKKKLKKLQTELDSHNLAPLLPNNNNNNINYDNHHQFSWQLSVNNNDKQGISSNSSSSSLYASS
TENISRLLEGWMRSSPNNPSRRNNIDHDHDDEMLHVAQKNDQDQEIVKSTCEENENSWIIPKEKSSNCESNGVVVVDHGK
TSPPPFTYLEKWLLEENGGQVEELMELPMIFT

>CcMYB95 |Locus_ID=BC332_09091 |Protein_ID=PHU23984.1 |Protein_len=334 |
chr03:252973028-252974354 |strand= -
MGRPPCCDKIGVKKGPWTPEEDIILVSYIQQHGPGNWRAVPGNTGLLRCSKSCRLRWTNYLRPGIKRGNFTEHEEKMIIH
LQALLGNRWAAIASYLPQRTDNDIKNYWNTHLRKKLKKIQGNDENSTGQEGATSSSSQSNFIKGQWERRLQTDIHMAKKA
LVEALSLDKSDSLANPISPVLAQQTVPGSSSYASSAENISRLLQNWMKNSPKSSQLSRSNSETTQSSLNNPSIGSGSGSS
SSPSEGTISGATPEGFDTLFSEGNAFTPENSGIFQVESKLNFPNSNSENGILFQEESQAPLTLLEKWLFDDAINAPTQED
EFIGMDLGESAGFF

>CcMYB96 |Locus_ID=BC332_33109 |Protein_ID=PHT97965.1 |Protein_len=370 |
scaffolds:252070-253948 |strand= -
MGRPPCCEKTGVKKGPWTPEEDIILVSYIQEHGPGNWRNIPTNTGLLRCSKSCRLRWTNYLRPGIKRGNFTEHEEKMIIH
LQALLGNRWAAIASYLPQRTDNDIKNYWNTHLKKKLNKKVESHGNDAHDHENNHQEGASTSSSSSQSRISKGQWERRLQT
DIHTAKQALCEALSLDASPNKTIPINPEQQQQPAVQASMTYASSAENIAKLLQNWMKNSPNSTSSSSSKITQQMSLNNNL
SVGALSSSSPSEGTVNVAPPQGLDSLFSFNSSNNSDVSQSVSIDEGGNLISTPENNNAGIFQVESKPILPDFKPESGIHQ
EESKQNLETQVPLTLLEKWLLDDANAQAEQEDQLMGIGMGMAMSETADLF

>CcMYB97 |Locus_ID=BC332_14191 |Protein_ID=PHU18496.1 |Protein_len=453 |
chr05:230830190-230832364 |strand= -
LLRCGKSCRLRWTNYLRPNLKRGKFTAEEDETIVKLHSTLGNRWSLIAQDLPGRTDNEIKNYWNSNLRRRLYSFRNFKEL
IKTTTNVPKIAVDGDSDESLRKQDTSTMSKEGSIESLLPEDNIVVDRGSTRKNRSLCVGSNEGATWSEGDSTESLLPKNC
TVDIGSMRKPYSSCPRGKDDVSWYRGGSTESLLLENIEINFACEENSIGSLLMENCSCPGGRDDTSWSEEGGSFKSLLLE
NIETNFSCPKGTCNATWSEGGSVEFFSLENCAVDLGSNRENKSDKGGSIEYLLTGNYTVDIGFNRETNSSSCPRGNAEAM
WYEEGSSIESSSTESYIVDLDFLTQSPTRKTEELAMQQHEHHAKSKIHEPNEEGEAKNIKDIIHDSPKQQLCQGICSIGE
QEQQIQGPHEQNEENEIVIQQKHNDCLHNFDNNNWSNINFDYFLEDEYGIWNH

>CcMYB98 |Locus_ID=BC332_02702 |Protein_ID=PHU30609.1 |Protein_len=450 |
chr01:210100644-210105740 |strand= +
MVSLFIGTGLITLPPHCHFNSNIIIRRERESYYWCWYANMGRAPCCEKVGLKRGRWTAEEDQILTNYILSNGEGSWRSLP
KNAGLLRCGKSCRLRWINYLRSDLKRGNITSQEENIIIKLHATLGNRWSLIAGHMPGRTDNEIKNYWNSHLSRKVESLRI
PSDEKLPQAVVDLAKKGALMPIKQSRKTSRISNMKKNKTSKNSNLAEPKEAKENSTSGPLSASAVSIPSTLNMEKEALSS
TISSWLDGKNMPASDSAMDSMQEDVADVAAPNPRVGSRESHSSLSSEDCDMEWLEEIMPDVVINDQDMDPNFILSSLENG
QGETPLNETGNNYCRTTHRIDERNKTVSSQEAVSSTNEKSRLLMEAANDGRLEWDWQDDHELVWPAMSWELETKQENIGS
SSIPSWPLWDTGTDLLQNFMNHEDPVEIDSMHIMSENDQNPSALAAWLLS

>CcMYB99 |Locus_ID=BC332_14511 |Protein_ID=PHU13306.1 |Protein_len=270 |
chr06:271656-275644 |strand= -
MGRTPCCEKVGLRKGRWTKEEDEILTNYILANGEGSWRSLPKNAGLLRCGKSCRLRWMNYLKTDLKRGNITSDEEAIIIK

LRATLGNRWSVIAEYLPGRTDNEIKNYWNSRLSRKVDSLRIPSDEKLPLAVVEMAKNKGIQEQIVKRQRRKRKADSNFMT
ETSVSTSSCYNNNNNNDDVVLMDDKDAELDQDFIFNCLWDCEGEDLEIVENNNNEVLSYDNYDQLDCWNWEQLDEIWANE
EATRQEIDNNDNVQNCMNQQQVPIENVVIW

>CcMYB122 |Locus_ID=BC332_25527 |Protein_ID=PHU04705.1 |Protein_len=282 |
chr10:86998929-87004403 |strand= +
MGRSPCCEKAHTNKGAWTKEEDERLIDYIRAHGEGCWRSLSKPAGLLRCGKSCRLRWINYLRPDLKRGNFTEEEDELIIK
LHSLLGNKWSLIAGRLPGRTNNEIKNYWNTHIRQKLLSRGIDPTIHRSINEPTPKVTTITFAAAADSTDDHHRDQKINIK
AEFDETSNEDNNEVPKMSCLPDLNLELRISPPHHQQLDHHHQRSNSLCFTCSLGIQNSKDCSCNNTNGIGCSNNIVSMNI
VTGISLCCLNAAVLPPNPSRHETEVWMGAFAVSDSINRFQRC

>CcMYB123 |Locus_ID=BC332_13322 |Protein_ID=PHU17627.1 |Protein_len=270 |
chr05:88778559-88782778 |strand= +
MKKLVEYIKKHGHGSWHSLPHLAGLARCGKSCRLRWTNYLRPDIKRGPFSHEVEKFVIQLHGMLGNRWAAIASQLPGRTD
NEIKNLWNTHLKKRLLSMGINPQTHEPSSAPNGLLVTPLTSLAARHMAQWESARLEAEAHLSRESQLLVPSSVESSGTDY
FLRIWNSEVGEAFRKFNKKKAGLVKVQPLKHLHQQNTKWKNGQPYKGDVFQGYDDTSSSSGLEDSSESALQLPFDFLSNN
DMSFLRHSDTYSLYPGFLSESSLKCSSTEQ

>CcMYB124 |Locus_ID=BC332_14051 |Protein_ID=PHU18356.1 |Protein_len=237 |
chr05:227335834-227337292 |strand= +
MGRWGIIEEEWRKGPWTVEEDKLLIEYVNLHGEGRWNSVARLAGLKRNGKSCRLRWVNYLRPDLKRGQITPYEERIILEL
HAIWGNRWSTIARSLPGRTDNEIKNYWRTHFKKKVKKSSTDNFEKTKARLLKRQQSQQKQLLNSQIDMKRFMSQNENKVP
SHVPKQDMAILYQNNSFHEQEQSGFFYSMNNGYAIVPEASSNEDIMWDIGLWNLDDFNVNLPVSAVESGFAIKEFKI
