# Supplementary figures and images for: Capsicum chinense MYB Transcription Factor Genes: Identification, Expression Analysis, and Their Conservation and Diversification With Other Solanaceae Genomes
Source: Front Plant Sci. 2021 Oct 13;12:721265. doi: 10.3389/fpls.2021.721265 (PMC8548648; doi:10.3389/fpls.2021.721265)

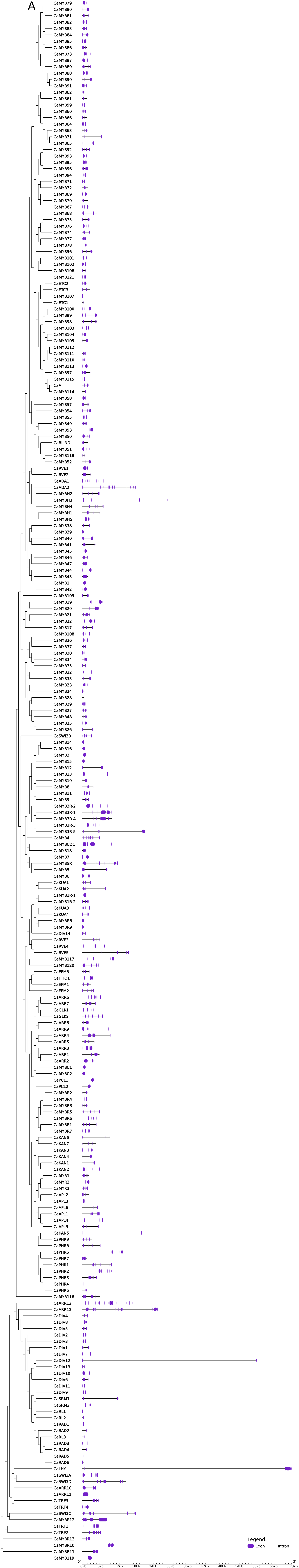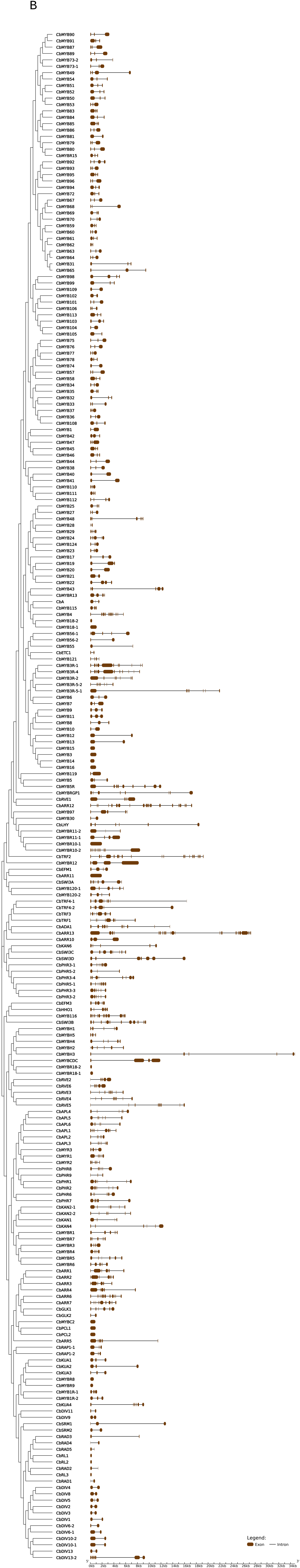

Supplement: Supplementary Figure 1 — Chromosomal distribution of MYB genes across 12 Capsicum chromosomes of (A) C. annuum and (B) C. baccatum. The capsaicinoid QTLs in C. annuum adapted from Han et al., 2018 are shown in shaded blocks. [file Data_Sheet_1.zip › Supplementary Figure S2.PDF]

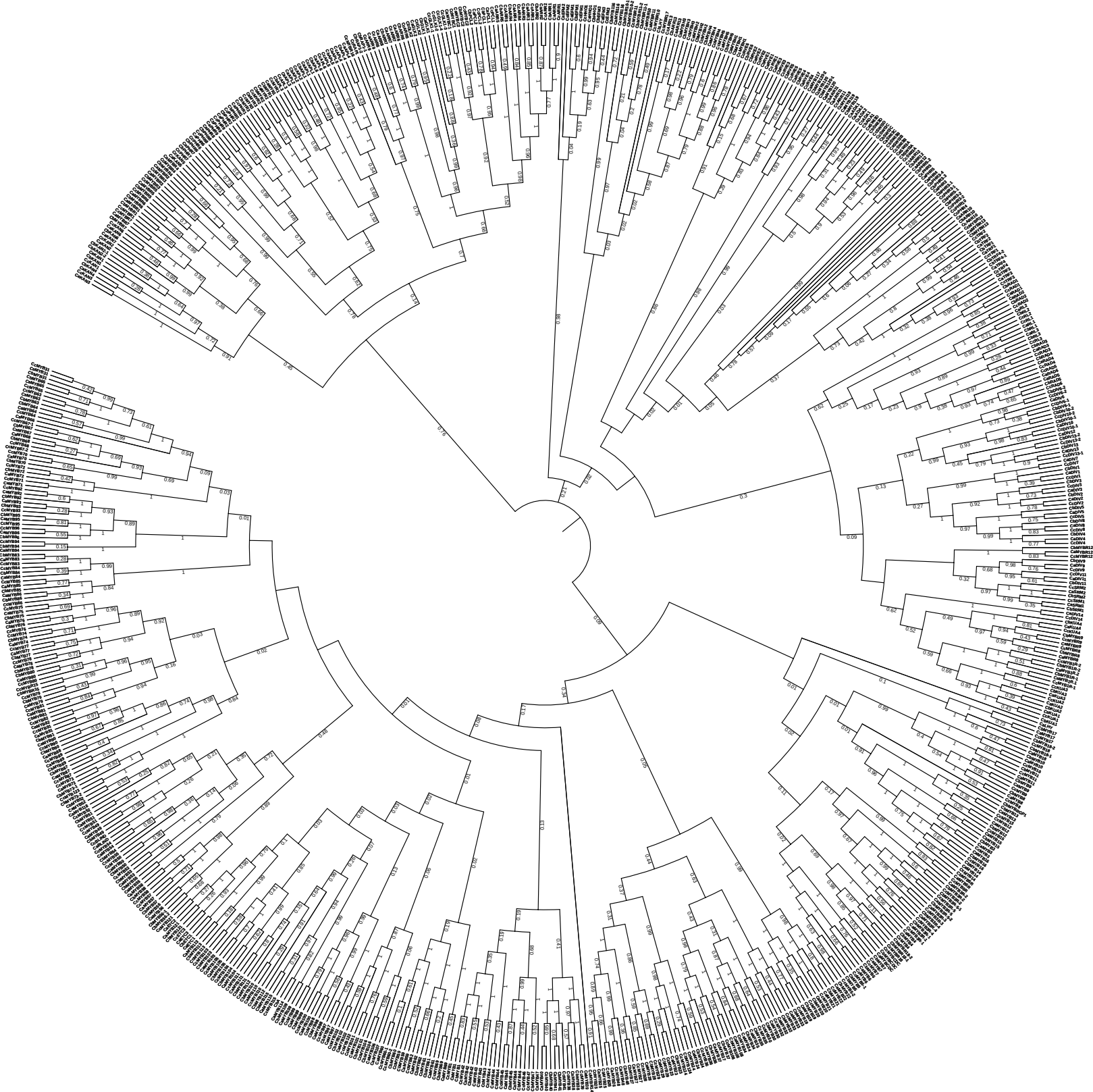

Supplement: Supplementary Figure 1 — Chromosomal distribution of MYB genes across 12 Capsicum chromosomes of (A) C. annuum and (B) C. baccatum. The capsaicinoid QTLs in C. annuum adapted from Han et al., 2018 are shown in shaded blocks. [file Data_Sheet_1.zip › Supplementary Figure S3.PDF]

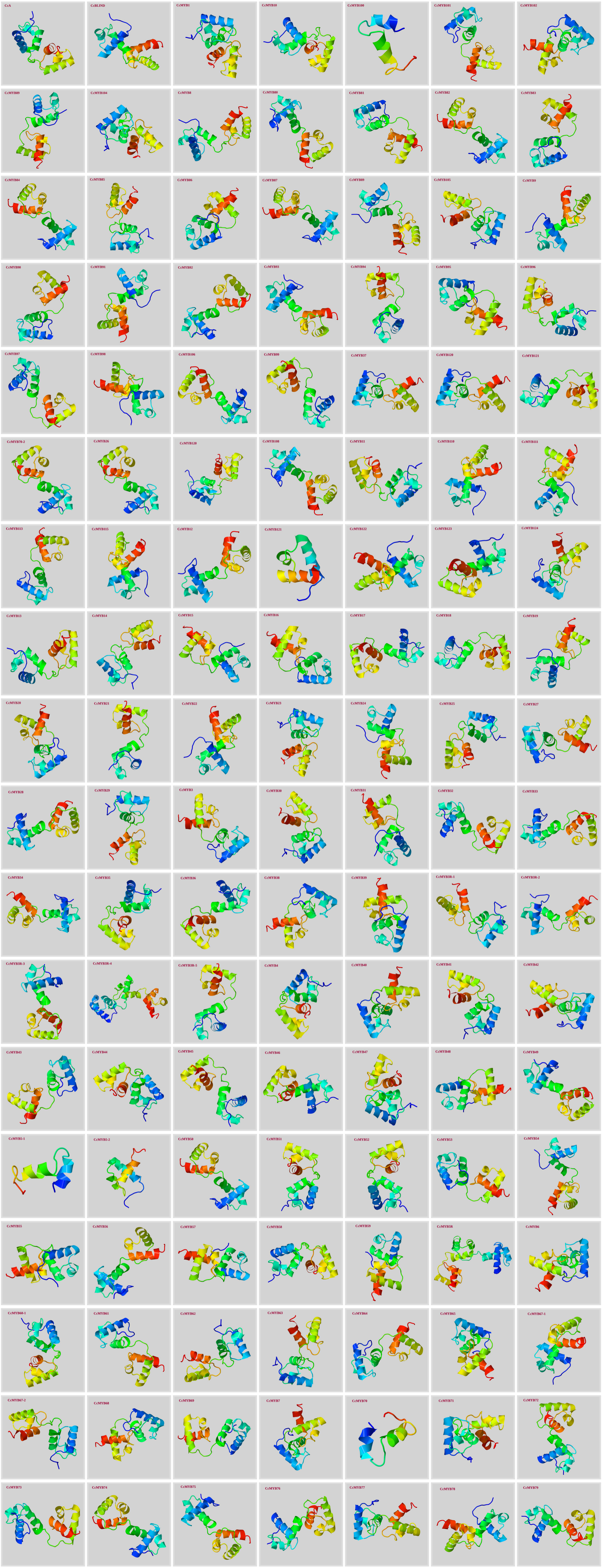

Supplement: Supplementary Figure 1 — Chromosomal distribution of MYB genes across 12 Capsicum chromosomes of (A) C. annuum and (B) C. baccatum. The capsaicinoid QTLs in C. annuum adapted from Han et al., 2018 are shown in shaded blocks. [file Data_Sheet_1.zip › Supplementary Figure S4.PDF]

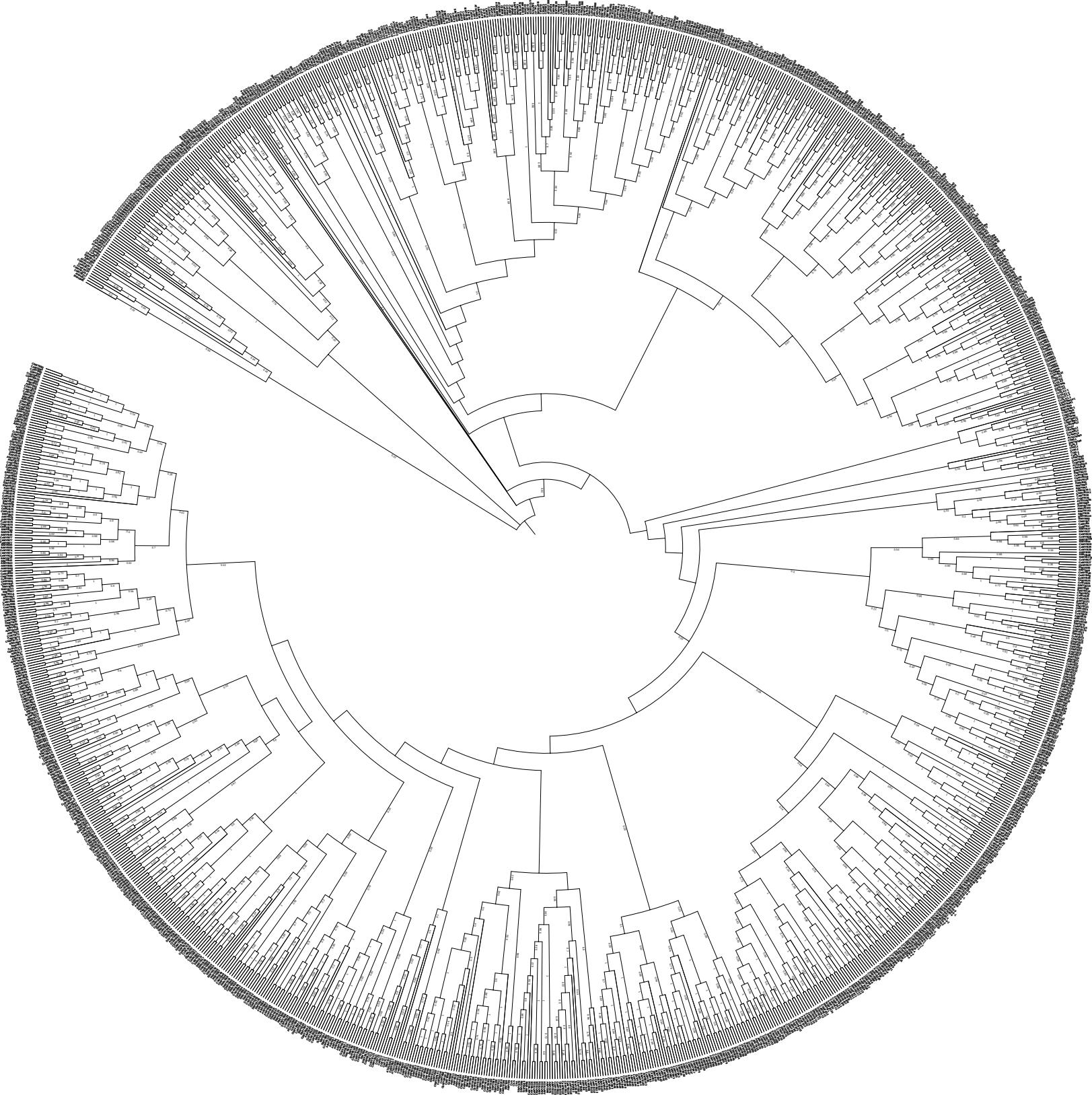

Supplement: Supplementary Figure 1 — Chromosomal distribution of MYB genes across 12 Capsicum chromosomes of (A) C. annuum and (B) C. baccatum. The capsaicinoid QTLs in C. annuum adapted from Han et al., 2018 are shown in shaded blocks. [file Data_Sheet_1.zip › Supplementary Figure S5.PDF]

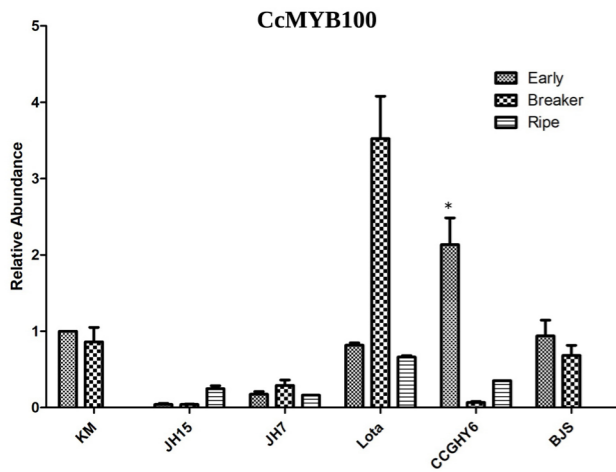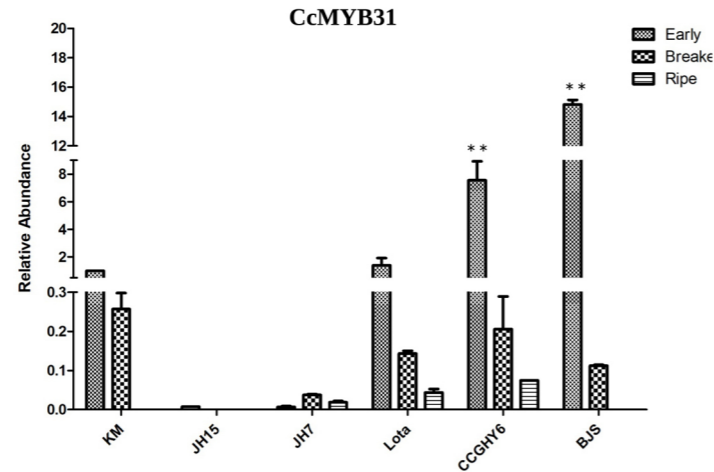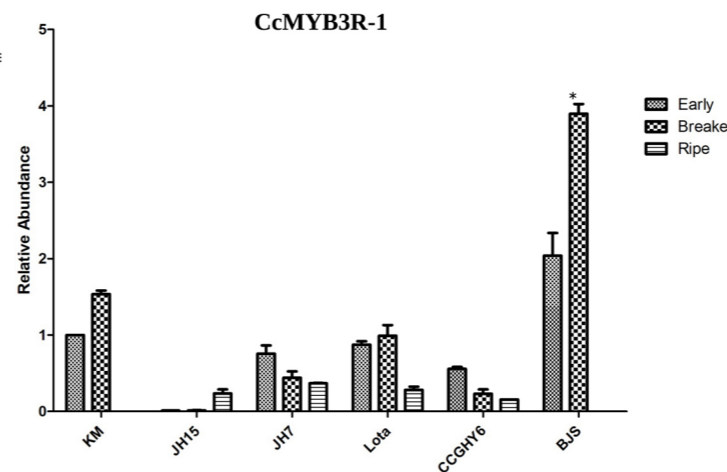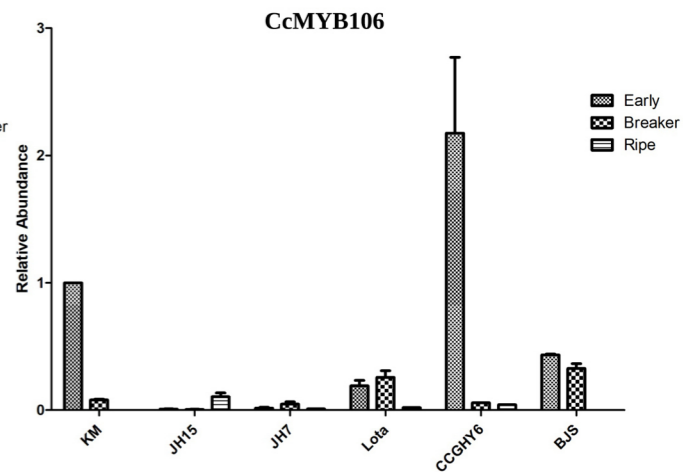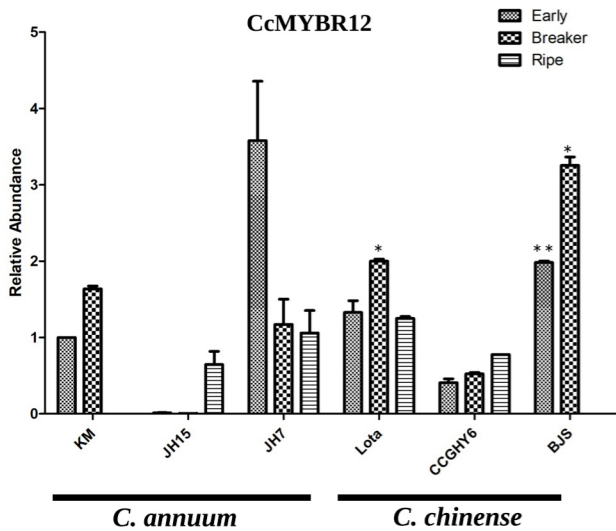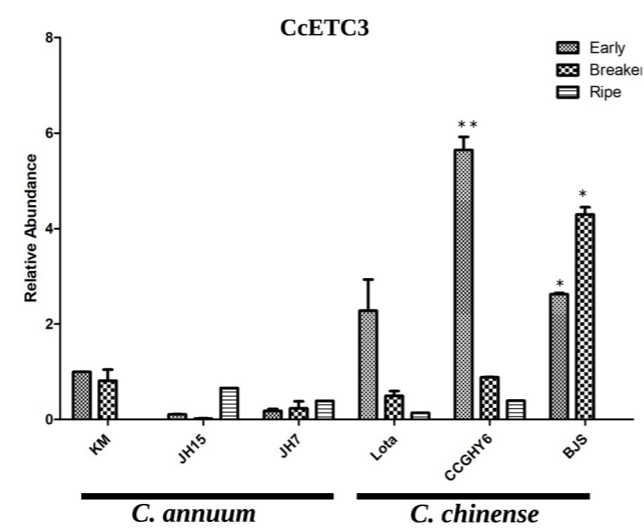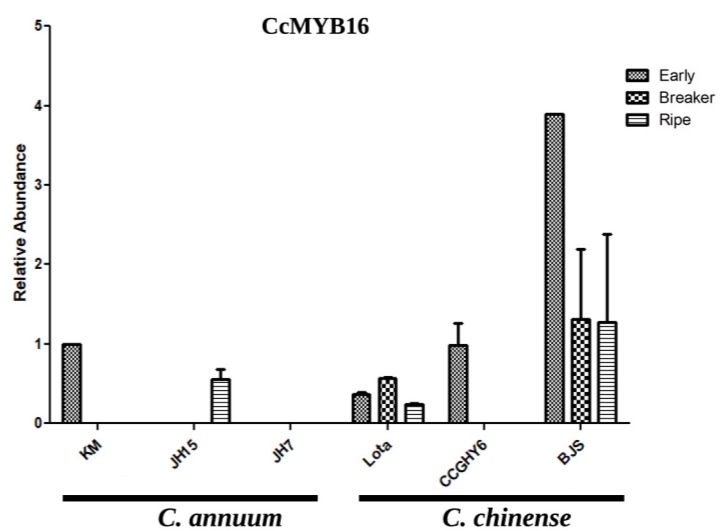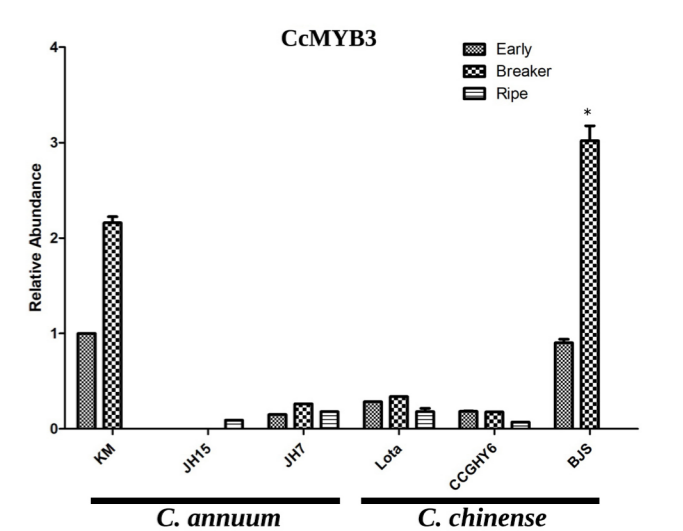

Supplement: Supplementary Figure 1 — Chromosomal distribution of MYB genes across 12 Capsicum chromosomes of (A) C. annuum and (B) C. baccatum. The capsaicinoid QTLs in C. annuum adapted from Han et al., 2018 are shown in shaded blocks. [file Data_Sheet_1.zip › Supplementary Figure S6.PDF]
